# High-throughput transcriptome sequencing and comparative analysis of *Escherichia coli* and *Schizosaccharomyces pombe* in respiratory and fermentative growth

**DOI:** 10.1371/journal.pone.0248513

**Published:** 2021-03-17

**Authors:** Joivier Vichi, Emmanuel Salazar, Verónica Jiménez Jacinto, Leticia Olvera Rodriguez, Ricardo Grande, Edgar Dantán-González, Enrique Morett, Armando Hernández-Mendoza

**Affiliations:** 1 Centro de Investigación en Dinámica Celular, Instituto de Investigación en Ciencias Básicas y Aplicadas, Universidad Autónoma del Estado de Morelos, Cuernavaca, Morelos, México; 2 Instituto de Biotecnología, Universidad Nacional Autónoma de México, Cuernavaca, Morelos, México; 3 Centro de Investigación en Biotecnología, Universidad Autónoma del Estado de Morelos, Cuernavaca, Morelos, México; University of Cambridge, UNITED KINGDOM

## Abstract

In spite of increased complexity in eukaryotes compared to prokaryotes, several basic metabolic and regulatory processes are conserved. Here we explored analogies in the eubacteria *Escherichia coli* and the unicellular fission yeast *Schizosaccharomyces pombe* transcriptomes under two carbon sources: 2% glucose; or a mix of 2% glycerol and 0.2% sodium acetate using the same growth media and growth phase. Overall, twelve RNA-seq libraries were constructed. A total of 593 and 860 genes were detected as differentially expressed for *E*. *coli* and *S*. *pombe*, respectively, with a log2 of the Fold Change ≥ 1 and False Discovery Rate ≤ 0.05. In aerobic glycolysis, most of the expressed genes were associated with cell proliferation in both organisms, including amino acid metabolism and glycolysis. In contrast in glycerol/acetate condition, genes related to flagellar assembly and membrane proteins were differentially expressed such as the general transcription factors *fliA*, *flhD*, *flhC*, and flagellum assembly genes were detected in *E*. *coli*, whereas in *S*. *pombe* genes for hexose transporters, integral membrane proteins, galactose metabolism, and ncRNAs related to cellular stress were overexpressed. In general, our study shows that a conserved "foraging behavior" response is observed in these eukaryotic and eubacterial organisms in gluconeogenic carbon sources.

## Introduction

During the past decade, the amount of biological data has dramatically increased due to the development of high throughput experimental biology such as massive parallel DNA sequencing technologies. These resources and the continuous improvement of bioinformatics tools allowed us to explore and reveal that universal principles in living systems, such as cell cycle, responses to environmental change, metabolic pathways, and gene regulation, are generally conserved in eukaryotes, eubacteria, and archaea [1–3]. For example, *Saccharomyces cerevisiae*, *Caenorhabditis elegans*, *Drosophila melanogaster*, and *Mus musculus*, among others, have been used as study models for human’s neurodegenerative diseases such as Alzheimer’s, Parkinson’s and Huntington’s, for cardiovascular and inflammatory diseases, diabetes, metabolic disorders, including the regulatory genetic elements that are conserved among all organisms [4–9]. In this evolutionary context, even an organism as simple as *Escherichia coli* can be compared with eukaryotes to delimit differences and similarities with other unicellular eukaryotic organisms, such as the yeasts *S*. *cerevisiae* and *Schizosaccharomyces pombe*. For instance, *E*. *coli*, *S*. *cerevisiae*, and *S*. *pombe*, are ideal model microorganisms to study the evolution between a prokaryote and a unicellular eukaryote because they carry out common processes such as the biosynthesis of amino acids, nucleotides and organic compounds, as well as metabolic events such as the Crabtree effect [10], which has been considered as an event analogous to the Warburg effect in tumor cells. Comparing the regulation of transcription and translation in these organisms can help us to understand the conservation and divergence of the biochemical mechanisms, protein, and protein interaction networks, transcriptional regulators, and gene regulatory networks [11].

Biological interaction networks are generally context-specific since they are based on structural, qualitative, and quantitative data obtained under controlled specific cell or tissue conditions, but it is evident that during evolution they were subjected to selective pressure. On the one hand, yeasts, such as *S*. *cerevisiae*, are capable of fermenting glucose to ethanol, even in the presence of oxygen, a phenomenon known as aerobic glycolysis. They also carry out an oxidative metabolism when glucose is low or when the carbon source is not fermentable, as is the case of succinate, ethanol, glycerol, and other compounds [12]. Glucose concentration reduction in the environment has a wide range of effects in regulatory networks, cell morphology, and life span increase induced by caloric restriction in yeast [10]. Similarly, *E*. *coli*, when grown in media with a high glucose concentration, accumulates acetyl Co-A and suppresses the expression of genes involved in the uptake of alternative sources of carbon. As in yeast, when glucose is scarce, *E*. *coli* can use other carbon sources such as glycerol, pyruvate, succinate, among others [13].

*E*. *coli* and *S*. *pombe* are of particular interest for the analysis of regulatory circuits conserved through evolution. Both species divide by binary fission and constitute models of symmetrical cell division. These organisms are considered models for the study of the caloric restriction in microorganisms, and when they grow in excess of glucose under aerobic conditions, they induce glucose-mediated aerobic acidogenesis (aerobic fermentation) or the Crabtree effect [10]. Besides, both organisms have common points in oxidative stress regulation [14], and mechanisms of post-transcriptional control of gene expression mediated by RNAse III [15,16]. These processes involve the participation of proteins such as Hfq *in E*. *coli* [17,18] and AGO1 in *S*. *pombe* [19,20].

RNA-seq has been used to obtain a representation of full transcripts, discover unknown genes and detect differential expression of both well-annotated and non-annotated regions of the genome and to infer regulatory networks of several cellular processes that were assumed exclusively to the dominion of proteins [21]. This shows that the transcriptomic profile presents more complexity and dynamism than the genome [22]. In both eukaryotes and prokaryotes, regulation of gene expression occurs by the combination and concerted action of transcription factors and ncRNAs, ensuring an accurate response to a given stimulus. At the transcriptional level, transcription factors bind to promoter regions and enhance the activation or repression of genes. At the post-transcriptional level, ncRNAs generally have a number of roles, such as suppressing the expression of genes by degradation or inhibition of their translation, or increasing the stability of the messenger and therefore its half-life [17,19]. The sRNAs in bacteria and the siRNAs/miRNAs in eukaryotes regulate gene expression at both transcriptional and post-transcriptional levels, interacting by complementarity with another nucleic acid molecule, or by altering the activity of a protein or protein complex.

On both organisms several transcriptomic analyses have been performed using glucose or glycerol as a carbon source operating the optimal conditions for each organism. However, there are no studies where both species were compared in the same growth media to reduce the variations that could influence the overall expression of the gene, making it difficult to compare results between the two organisms.

The present study aims to characterize the transcribed regions of the genome by RNA-seq obtained from the total RNA of *E*. *coli* MG1655 and *S*. *pombe* 972 h-, grown in similar conditions. In order to reduce the experimental variations, we generated RNA libraries of both organisms grown in the same modified YE medium to compare only the response to the carbon source, supplementing the media with 2% Glucose or a mixture of 2% Glycerol/0.2% Sodium acetate and collecting the samples at the same growth phase. This is the first report paralleling the growth of *E*. *coli* and *S*. *pombe* in the same growth media, comparing only the change of the carbon source. Data analysis showed a differential expression of protein-coding genes and ncRNAs. In glucose, we observed that enriched functional categories related to cell proliferation are a common response as expected, while glycerol catabolism, stress, cell differentiation and strong expression of ncRNAs are evident in gluconeogenic carbon source in both organisms, strengthening the idea that these molecules are essential for the regulation of genes involved in stress conditions. There is also evidence of gene expression in both organisms involving a "foraging behavior" phenotype when glucose is absent, a result that is supported by cell morphology observations. This research also serves as a framework for future research into functional comparative genomics between eubacteria and eukaryotes.

## Materials and methods

### Strains

*E*. *coli* K-12 MG1655 and *S*. *pombe* 972 h- were the strains used for this work (donated by Dra. Elena Hidalgo).

### Culture media and growth conditions

*E*. *coli* was maintained in Luria Bertani (LB) agar plates and *S*. *pombe* in Yeast Extract with Supplement (YES) agar plates. To compare both organisms, they were grown in Yeast Extract (YE; 5 g/L Yeast Extract, 3 g/L KH_2_PO_4_) medium under the two following conditions: the first condition used 20 g/L glucose as a carbon source while the second condition employed 20 g/L glycerol and 2 g/L sodium acetate; in the two conditions pH was adjusted to 7.2 for *E*. *coli* and 6 for *S*. *pombe*. Cells were incubated at 220 rpm, and an optimal temperature of 37°C for *E*. *coli* and 30°C for *S*. *pombe*. Growth started at an optical density of 0.05 at 600 nm in all experiments. Three independent biological replicates were performed for each condition.

For total RNA extraction, cultures were harvested at the middle log phase and after RNAlater (Ambion ®) was added according to manufacturer recommendation. Cultures were harvested by centrifugation at 6500 rpm for five minutes and stored at -70° C.

### RNA libraries

Total RNA was isolated with TRI Reagent®, followed by precipitation with isopropyl alcohol. DNA contamination was removed treating with DNase (Zymo Research) and RNA was concentrated using RNA Clean and Concentrator™ -5 columns (Zymo Research) following the manufacturer’s instructions. Ribosomal RNA depletion was performed with RiboMinus ™ Yeast/Bacteria Transcriptome Isolation Kit at 1 μg/μl of RNA concentration. Next-generation sequencing (NGS) libraries were prepared using Illumina TruSeq Stranded mRNA preparation kit as per the supplier’s instructions with dual indexed Illumina adapters. Paired-end (2 × 75 bp) sequencing multiplexing barcodes were added to the pooled libraries using the Illumina NextSeq 500 platform by the Unidad Universitaria de Secuenciación Masiva y Bioinformática (UUSMB)–UNAM (http://www.uusmb.unam.mx/).

### Sequencing data analysis

FASTQC available at http://www.bioinformatics.babraham.ac.uk/projects/fastqc/ was used for quality control analysis of all sequences. Trimmomatic v0.36 [23] was used to select a quality filtering score >20, removing 3’ end low-quality bases and trim adaptors. Forward and reverse-read mate pairs were aligned to the genome of *E*. *coli* MG1655 K-12 and *S*. *pombe* 972 h- using HISAT2 [24] for both organisms. Mapping parameters were set to align the best quality reads and report the best alignment, without accepting mismatches. SAMtools [25] was used to remove optical duplicates of PCR and sequence reads with a MAPQ < 30, with the objective to collect high quality alignments for estimating the abundance of unique transcripts resulting in 1.3 million on average in all libraries. The number of sequences reads that mapped to the genomes were tabulated from the resulting BAM alignment files using coverage BEDtools [25]. Gene with at least one count per million measurements in two replicates were analyzed for significant differential expression analysis.

The TMM method was used to normalize effective library size and the generalized linear model (GLM) likelihood ratio test was used to detect differentially expressed genes (DEGs), requiring a log2 of the Fold Change (log2FC) ≥ 1 and with a False Discovery Rate (FDR) of ≤0.05, using the EdgeR package in R [26].

An overview of the gene expression data has been deposited in NCBI’s Gene Expression **Omnibus (GEO) accessible through GEO Series accession number GSE138088.**

To review GEO accession GSE138088:

**Go to https://www.ncbi.nlm.nih.gov/geo/query/acc.cgi?acc=GSE138088**

Enter token mtejauqknbkjpol into the box

### Bioinformatic analyses

To compare the presence of orthologs among both species we used OrthoVenn software [27,28], using 4242 and 5132 protein coding sequences of *E*. *coli* and *S*. *pombe*, respectively (*E*. *coli* genome information is located in the NCBI link https://www.ncbi.nlm.nih.gov/genome/167?genome_assembly_id=161521 and *S*. *pombe* in https://www.ncbi.nlm.nih.gov/genome/14?genome_assembly_id=22534).

OrthoVenn2 generates protein clusters where each cluster consists of species orthologs or paralogs. The overlapping cluster means proteins from different species contained in the cluster. For instance, if cluster 1 has three A-species proteins and two B-species proteins, this means cluster 1 is an overlapping cluster for A and B, which also means shared cluster 1 for A and B. In addition, to compute the p values for GO terms being enriched in clusters overlapping, OrthoVenn uses hypergeometric distribution described in (https://orthovenn2.bioinfotoolkits.net/help). Pair-wise sequence similarities between all input protein sequences were calculated with an e-value cut-off of 1e−5. An inflation value (−I) of 1.5 was used to define orthologous cluster structure in *E*. *coli* and *S*. *pombe*.

To compare and complement the analysis obtained with OrthoVenn, we used the information of ortholog genes predicted by PANTHER phylogenetic trees stored in PANTHER database [29] that contains the complete sets of protein coding genes for 142 organisms, obtained from the definitive Reference Proteomes project at UniProt. For each gene, PANTHER also reports orthologs and paralogs based on the inferred speciation and gene duplication events in the phylogenetic tree (ftp://ftp.pantherdb.org/ortholog/16.0/RefGenomeOrthologs.tar.gz).

We used the Gene Set Enrichment Analysis (GSEA) to examine gene sets from metabolic pathways generated and curated in the KEGG database or gene sets generated from literature data to gain insight into the observed phenotypes of both species [30] using the log2 Ratio of Classes option as a metric and selecting the significantly enriched gene sets of each condition, according with the default values of FDR < 25% and at nominal p-value < 5%, as indicated by the program. We also introduce our data of genes with Log2FC values for a Functional Enrichment Analysis in STRING database (https://string-db.org) for subsequently assignment of enriched Gene Ontology (GO), KEGG pathways, and local network STRING (LNS) terms as they are designated in this database [31,32], and to cluster the DEGs into functional categories with statistical enrichment using a lenient FDR stringency < 25% as allowed by the software, since we are using two species from two different domains of life. In total, 4,125 genes in *E*. *coli* and 5,092 for *S*. *pombe* were mapped and used for this analysis. We used the same strategy, but in a subset of data for each species, combining only data from orthologs and co-orthologs genes obtained from OrthoVenn and the PANTHER database. The enriched GO, KEGG pathways, and (LNS) terms identified were separated by condition and introduced in Jvenn [33], an interactive Venn diagram viewer software to create diagrams.

### Correlation analysis

Orthologs data were obtained from PANTHER database that contains orthologs files predicted by PANTHER phylogenetic trees. RefGenomeOrthologs.txt file that contains orthologs among 12 Model Organism Databases from the reference genome project, was downloaded via FTP from the ftp://ftp.pantherdb.org/ortholog/15.0/ database and construction of orthologues pairs genes was created between *E*. *coli* and *S*. *pombe*. The Pearson correlation coefficient (r-values) was established as the index of association in a set of ortholog pairs using their log2FC. The r-values were determined with linear regression between log2FC value in *S*. *pombe* and *E*. *coli* ortholog pairs. The cut-off value was 0.0145. This number corresponds to the average r-value of non-orthologous randomly selected gene lists. Global analysis was carried out with a gene pair set with the same Enzyme Commission (EC) number. Specific analysis was carried out with a subset of pairs gene related to glycolysis/gluconeogenesis, tricarboxylic acid cycle and pentose phosphate pathway. Positive r-values correspond to gene sets with correlated expression, negative values to inverse correlated gene sets or negative linear relationship, and values close to zero to non-correlated.

## Results

### Orthologs conserved among *E*. *coli* and *S*. *pombe*

**Fig 1:**
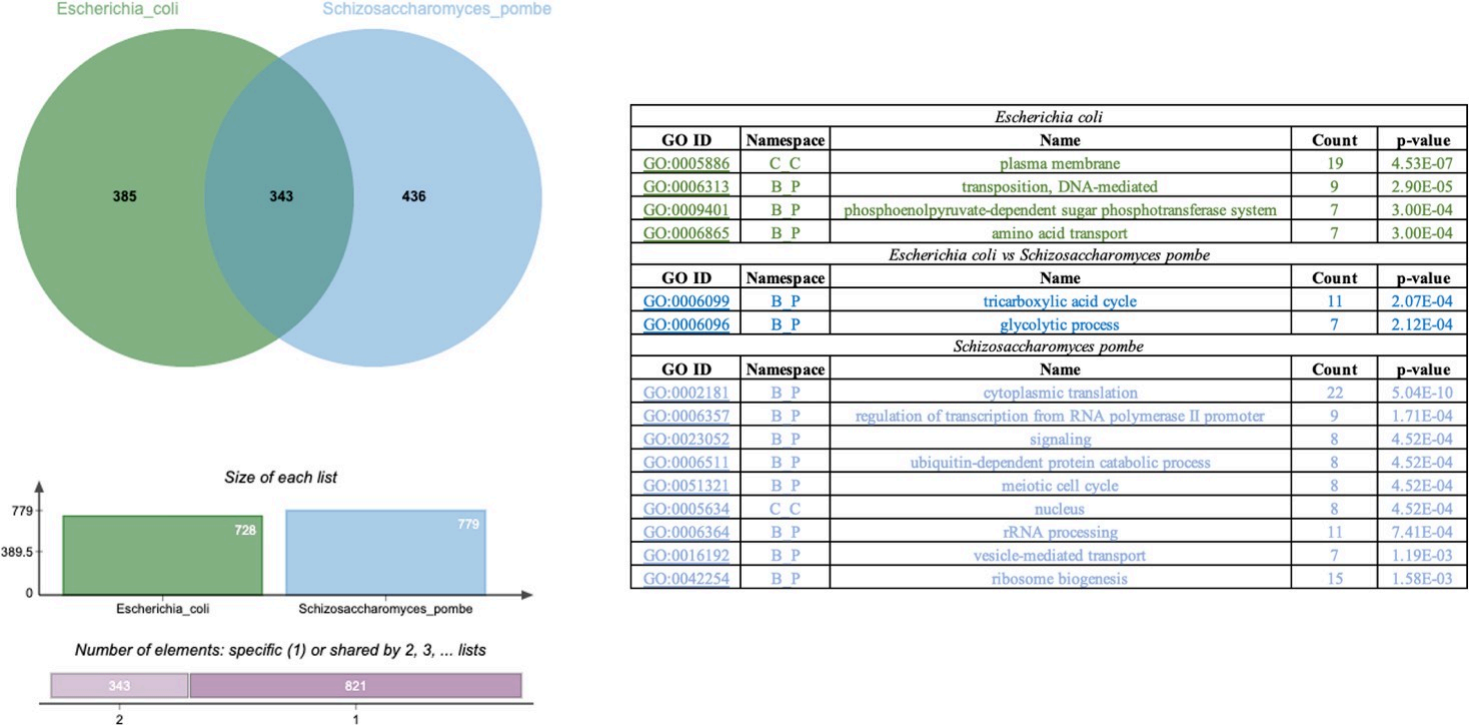
Analysis of ortholog and co_orthologs present in *Escherichia coli* and *Schizosaccharomyces pombe*. Venn diagram showing the distribution of shared orthologous clusters among *Escherichia coli* and *Schizosaccharomyces pombe*. Clusters of orthologs genes show that *E*. *coli* shares 343 clusters of orthologs genes with *S*. *pombe*, possessing 385 clusters that are only present in the bacteria and 436 in the fission yeast. Table shows the cluster of orthologs enriched in *E*. *coli*, *S*. *pombe* and in both organisms.

Despite *E*. *coli* and *S*. *pombe* belong to different domains of life, they have common processes such as binary fission division, positive Crabtree effect, regulation of oxidative stress and growth in gluconeogenic carbon sources such as glycerol. In order to identify ortholog genes from both genomes for classification within gene clusters, we applied an analysis with OrthoVenn software. The information gained from ortholog cluster comparisons can serve to better interpret the mechanisms underlying gene expression in a genomic context. Of the 4242 and 5132 protein coding sequences of *E*. *coli* and *S*. *pombe*, respectively, the software detects the presence of 718 direct orthologs, and 262 co_orthologs. The analysis showed that 1164 clusters, 916 orthologous clusters (at least contains two species) and 248 single-copy gene clusters (Fig 1) were formed based on the protein sequences of both species. The diagram shows that the two species share 343 gene clusters, indicating their preservation in the lineage after speciation, with the biological processes GO 0006099 (tricarboxylic acid cycle) and 0006096 (glycolytic process) being the enriched biological processes in both species. In addition, 436 clusters specific to *S*. *pombe* and 385 to *E*. *coli* were likely to be gene clusters within multiple genes or in-paralog clusters, indicating that in both species there could be a lineage-specific gene expansion in these gene families. Some of these lineage-specific clusters could potentially be involved in significant biological processes based on the annotation of these clusters (Fig 1). On the one hand, cytoplasmic translation, regulation of transcription from RNA polymerase II promoters, signaling, ubiquitin-dependent protein catabolic process, meiotic cell cycle, nucleus, rRNA processing, vesicle-mediated transport, and ribosome biogenesis were the enriched GO terms in *S*. *pombe* (Fig 1). Conversely, in *E*. *coli*, we found that the GO terms enriched were plasma membrane, transposition DNA-mediated, phosphoenolpyruvate-dependent sugar phosphotransferase system and amino acid transport (Fig 1).

### Whole-transcriptome RNA-seq analysis

To compare and understand the transcriptional response of both organisms to two different metabolic stages, the semi-defined medium YE was used with a source of carbon and glycolytic energy (2% glucose) or a source of gluconeogenic carbon (glycerol 2%/acetate 0.2%). This procedure allowed us to diminish the variations resulting from the use of culture media with different formulations and to infer parallel analogous growth responses. It was observed for *E*. *coli* in an adaptation phase in both carbon sources (S1A Fig) that was smaller than in *S*. *pombe* (S1B Fig). Similarly, in accordance with the literature, a difference in the generation time and length of the adaptation phase of *S*. *pombe* in glucose with respect to growth in glycerol was found (S1B Fig). For each carbon source, RNA was obtained from three biological replicas which constituted independent cultures of each microorganism. It is important to note that the same culture medium was used for the growth condition, and only the optimal growth temperature reported in the literature was adjusted, 37°C for *E*. *coli* (S1A Fig) and 30°C for *S*. *pombe* (S1B Fig).

Pearson correlations of raw read counts showed no biases in biological replicates. Correlation indices between replicates of the same growing conditions were higher than 0.94 and lower than 0.89 between different conditions. The RNA-seq of paired-end libraries averaged over 10.7 million reads for *E*. *coli* (range: 7.8 million to 13.6 million) and 6.8 million for *S*. *pombe* (range: 6.3 to 7.4 million readings). In total, 97% of paired reads mapped to the respective genomes.

For the 4,419 and 6,862 genes of *E*. *coli* and *S*. *pombe*, respectively, we performed a differential expression analysis between glucose and glycerol/acetate, of which 593 (13.41%) and 860 (12.54%) DEGs were detected for *E*. *coli* (S2A Fig) and *S*. *pombe* respectively (S2B Fig) (absolute log_2_FC > 1 and FDR < 0.05) (results are summarized in S1 and S2 Files). Although the volcano plot appears to be similar in the area corresponding to the two growth conditions for both organisms, a slightly higher number of DEGs were detected in *E*. *coli* and in *S*. *pombe* grown in glycerol/acetate (S2A and S2B Fig). In *E*. *coli*, a group of 330 genes were upregulated, and 263 were downregulated. In *S*. *pombe*, instead, 463 and 397 genes were upregulated and downregulated, respectively. A total of 321 ncRNA transcripts were differentially expressed in *S*. *pombe*. In *E*. *coli*, only five genes corresponding to sRNAs were significantly differentially expressed (S1 and S2 Files).

In *E*. *coli* grown in glucose the genes with the highest log2FC with respect to glycerol/acetate were *ydeS* (a putative fimbrial protein), *pyrB* (an aspartate carbamoyltransferase catalytic subunit), involved in pyrimidine biosynthesis, and *pyrI* (an aspartate carbamoyltransferase regulatory subunit) (S1 File). In *S*. *pombe*, the highly expressed genes, in addition to several tRNAs and ncRNAs, were SPAC821.09, a cell septum surface endo-1,3-beta-glucanase involved in cytokinesis, SPBPB10D8.02c, a predicted arylsulfatase, and an amino acid transmembrane transporter SPCC74.04 (S2 File).

The genes with the highest expression change between bacteria grown in glycerol/acetate compared to glucose were *glpT*, encoding the major uptake system for glycerol 3-phosphate, *flgB*, involved in rod section of the basal-body assembly of the flagellar motor, and *mglA*, coding for an ATP-binding component of a D-galactose/D-galactoside ABC transporter (S1 File). In *S*. *pombe*, some of the genes with the highest expression change in glycerol/acetate than in glucose have not yet been assigned a function, such as SPBC32H8.15 and SPCC794.16, but others, such as SPBC1348.14c, encodes a high-affinity Ght7 permease involved in the introduction of hexose into cells, and SPAC1F8.02c which is involved in the transport of transmembrane iron during the cellular response to iron starvation (S2 File). Interestingly, 54.2% of differentially expressed genes with higher expression in glycerol/acetate than glucose were non-coding RNAs.

### Functional enrichment analyses of GO, KEGG pathways, and LNS terms

**Fig 2:**
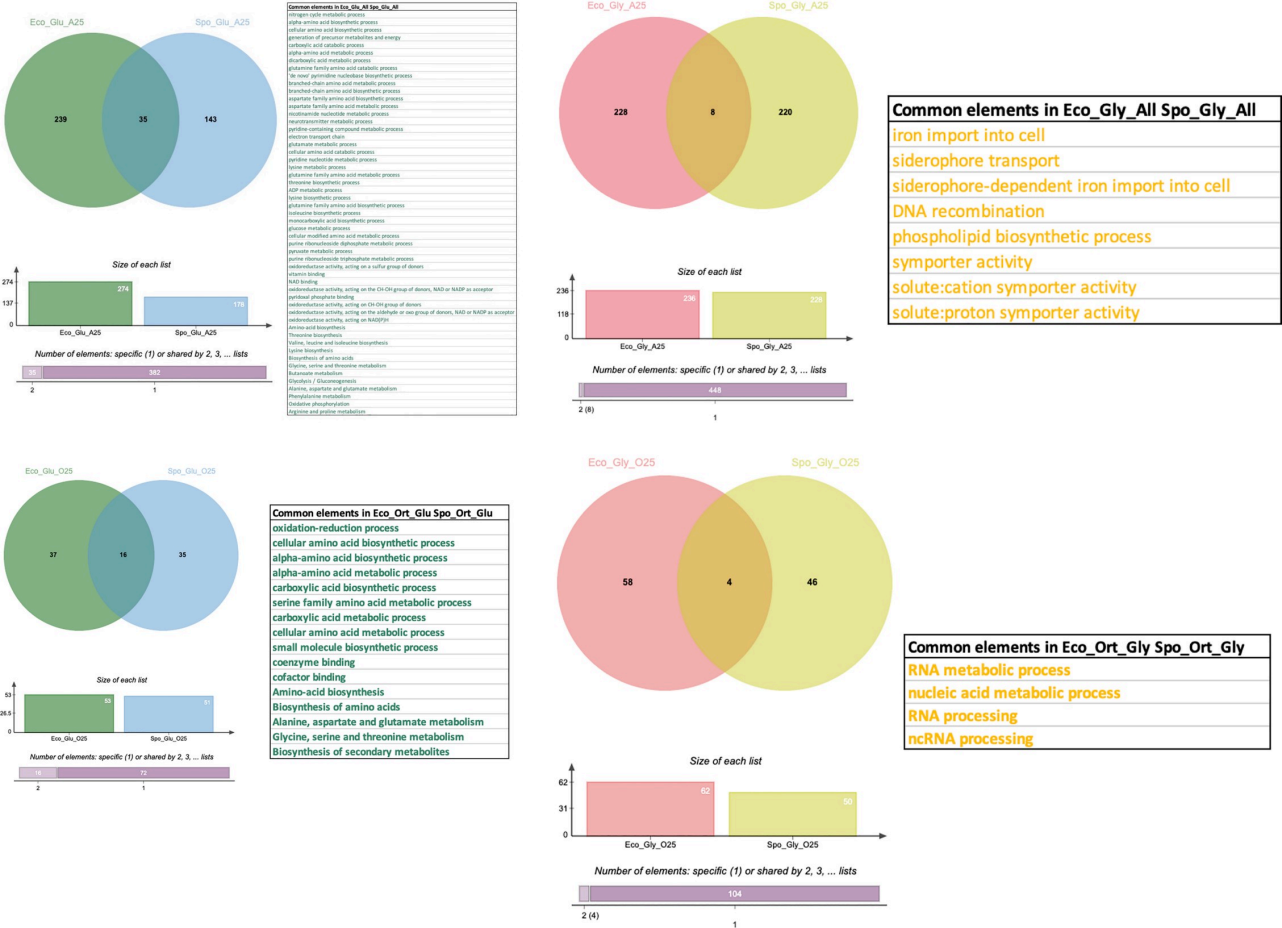
Functional gene set enrichment analysis of GO, KEGG pathways and local network STRING (LNS). DEGs data (RNA-seq) was analyzed for enriched gene sets of GO, KEGG pathways and local network STRING (LNS) terms using an FDR of 25% in the STRING database for all the genes of *E*. *coli* (Eco) (**a**) and *S*. *pombe* (Spo) (**b**) ranked by their Log2FC and separated by the condition where they were enriched (Eco_Glu_A25 and Spo_Glu_A25 in glucose and Eco_Gly_A25 and Spo_Gly_A25 in glycerol/acetate). (**c**) Same as in **a** but with a subset of ortholog and co_ortholog (O) genes from *E*. *coli*. (**d**) Same as in **b** but with a subset of ortholog and co_ortholog (O) genes from each *S*. *pombe*. (Eco_Glu_O25 and Spo_Glu_O25 in glucose and Eco_Gly_O25 and Spo_Gly_O25 in glycerol/acetate) Common terms in both conditions for *E*. *coli* and *S*. *pombe* are indicated in the tables.

Because two organisms belonging to different domains of life are contrasted and their gene expression varies, we used functional enrichment analysis as an alternative to compare absolute Log2FC values between the organisms. Analysis of enriched GO and KEGG pathways, and LNS terms in STRING shows that when analyzing all the genes in the experiments, 274 terms are enriched in glucose and 236 in glycerol/acetate in *E*. *coli* and 350 in glucose and 228 in glycerol/acetate in *S*. *pombe* (S1 and S2 Tables). For both organisms, we identified 53 shared enriched terms in glucose, most of them involved in amino acid metabolic, nitrogen cycle metabolic, carboxylic acid catabolic, oxidoreductase activity, Oxidative phosphorylation and Glycolysis/Gluconeogenesis processes, among others (Fig 2A). In glycerol/acetate we identified 8 common terms, most of them involved with iron import into cell, siderophore transport, DNA recombination, and symporter activity (Fig 2B). Since our previous analysis with OrthoVenn complemented with data of the PANTHER database indicates that only ~25% of the total genes from both species are orthologs and co-orthologs (S3 Table), we carried out the same analysis using this group of genes to discriminate between the conserved response of both organisms and the particular mechanisms evolved in *E*. *coli* or *S*. *pombe*. There were 115 terms enriched in *E*. *coli* (53 for glucose and 62 for glycerol) and 113 in *S*. *pombe* (62 in glucose and 51 in glycerol) when the expression levels of orthologs genes were analyzed (S2 Table). Once again, we identified the intersection of 16 terms in glucose for both organisms, conserving the oxidation-reduction, cellular amino acid metabolic, carboxylic acid metabolic, coenzyme binding, cofactor binding, and biosynthesis of secondary metabolites processes (Fig 2C). In glycerol/acetate, only 4 terms were shared in both organisms: RNA metabolic process, nucleic acid metabolic process, RNA processing, and ncRNA processing (Fig 2D). These results indicate that the core of orthologous and co-orthologous genes between both organisms share an adaptation to the use of glucose as a source of carbon by using amino acids and dicarboxylic acids for their metabolism. However, when both organisms were growing in glycerol/acetate rather than in glucose, we observed a conserved induction of orthologs involved in the processing of regulatory ncRNAs.

However, the detection of other enriched terms in both species indicates that each organism has also developed specific pathways to use each of the two carbon sources. On the one hand, the enrichment of GO terms associated with molecular function like ribosomal small subunit assembly, organic substance biosynthetic process, and alpha-amino acid metabolic process in glucose was higher than in glycerol/acetate (S1 Table). In this condition, GO terms and metabolic pathways associated with carbohydrate metabolism, formation of ribosomes, and protein and nucleotide synthesis were detected. This result revealed an enrichment of the genes involved in increasing biomass and cell proliferation in response to glucose levels in both organisms (S1 Table).

Also, when a source of gluconeogenic carbon was used for *E*. *coli* growth, the number of GO terms associated with cellular components like bacterial-type flagellum basal body, bacterial-type flagellum hook, and integral component of membrane was greater in glycerol/acetate than in glucose (S1 Table). GO terms related to the regulation of reproductive process, meiotic cell cycle, and the use of alternative carbon sources such as galactose, transport of hexoses, and transmembrane transport of ions have been enriched in *S*. *pombe*. It should be noted that in both organisms, none of the genes involved in a nutritional restriction response were overexpressed.

### Correlation of homologs expression associated with EC number in KEGG metabolic pathways

**Fig 3:**
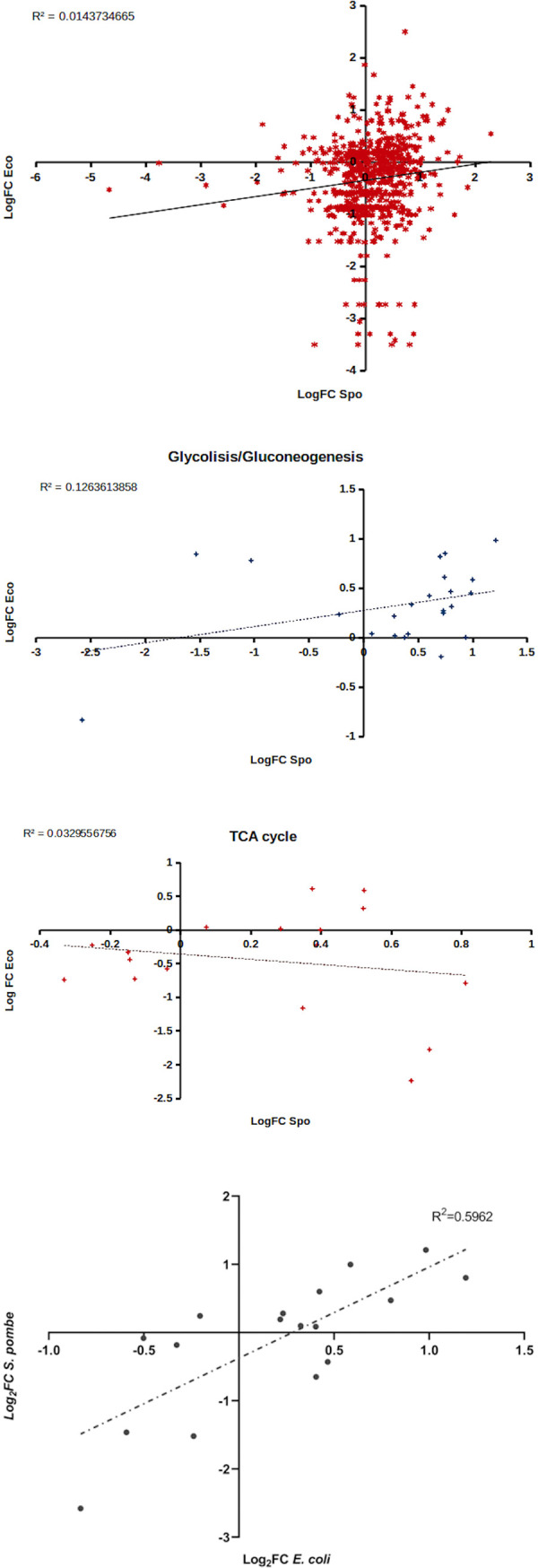
Relationship between Pearson’s correlation coefficient of log2 fold change of orthologs between *E*. *coli* and *S*. *pombe*. Each point (x, y) represents a gene orthologous pair formed by log_2_FC value in *E*. *coli* and *S*. *pombe*. (**a**) Representation of global orthologs gene pair. (**b**) Selected point in Glycolysis/Gluconeogenesis pathway. (**c**) Selected point in TCA cycle pathway. (**d**) Selected point in pentose phosphate pathway. Negative slope of the function implies negative correlation or negative association. A positive correlation (positive slope) indicates direct association.

PANTHER database has been used to determine the evolutionary-conserved gene expression response of homologs in both organisms [29]. A subset of data was created in the KEGG database using those homologs associated with an EC number. For this category, Pearson’s correlation coefficient showed a value of 0.1199 (Fig 3A). Significant differences were detected with a *p* value equal to 0.0049 when performing one sample t-test of this value with respect to the cut-off (0.0145). We reasoned at this point that there are pathways within this group that contribute more than others to the association of orthologs. The glycolysis/gluconeogenesis, tricarboxylic acid cycle, and the pentose phosphate pathways were analyzed to confirm this assumption. Linear regression showed r-values of 0.3555 (Fig 3B), -0.1815 (Fig 3C) and 0.7721 (Fig 3D) for these metabolic pathways respectively. These results showed a greater association in orthologs sets related to pentose phosphate pathway than glycolysis and very weak inverse association in tricarboxylic acid cycle pathway. These findings enable us to infer that in both organisms, the expression of the genes involved in processes of glucose transformation to obtain metabolite precursors is more correlated than the expression of the genes involved in energy production.

### Transcriptomic analysis and cellular phenotype indicate that glycerol growth induces a "foraging behavior"-like adaptation in both organisms

**Fig 4:**
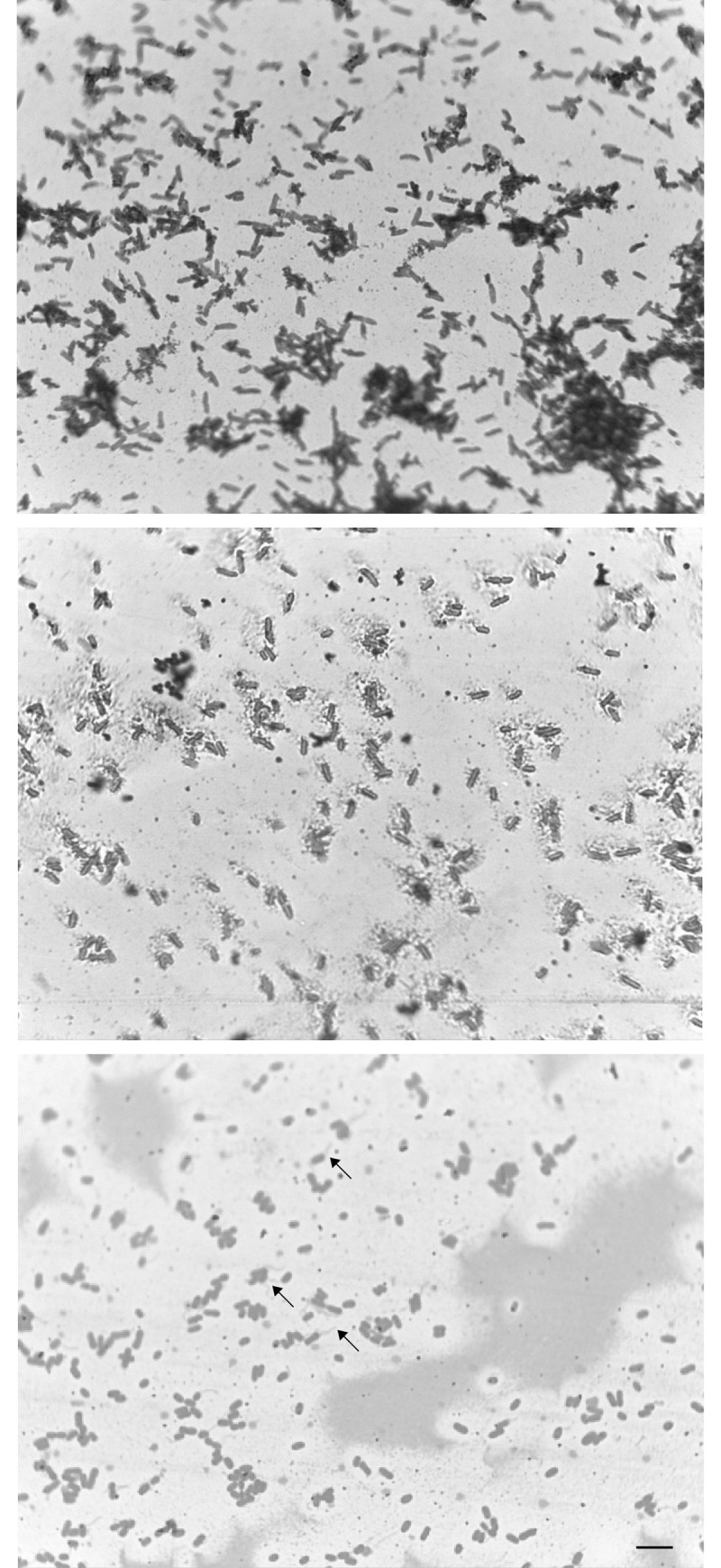
Light microscopic detection of flagella in *E*. *coli*. Bacteria were grown on YE medium supplemented with 2% glucose (**a**) or 2% glycerol and 0.2% sodium acetate (**b** and **c**), at 37° C for 2 h and stained with Leifson ’s stain. Cells growth in glycerol/acetate are shorter than in glucose and is evident in the peritrichous and polar flagella (arrows in c). Length bar, 5 μm.

Table 1 shows the results of the GSEA analysis. In *E*. *coli*, enriched gene sets in glycerol/acetate were lipopolysaccharide biosynthesis, carbohydrate transport, bacterial chemotaxis, and porins, all involved in changes in the composition of the membrane (Table 1). In addition, one of the clearest gene groups induced in the absence of glucose is that involved in flagellar assembly (*fli*, *flg* and *flh* regulons) [34], most of them displaying a log2FC < -1. One of the main transcriptional factors that showed differential expression in *E*. *coli* grown in glycerol/acetate compared to glucose was FliA which codes for σ^28^ sigma factor with an absolute value of log2FC of—2.5, which is required for the expression of transcription units associated with cell motility, signaling, and glycerol metabolism in anaerobic conditions. Based on this result, we compared the morphology of *E*. *coli* grown in both conditions by staining Liefson’s Method. Micrographies at 1000X clearly showed that in glycerol/acetate, the bacteria produced large flagella (Fig 4B and 4C) that were absent when grown in glucose (Fig 4A), supporting the bioinformatics analysis.

**Table 1:** GSEA analysis of KEGG metabolic pathways gene sets enriched in *E*. *coli* and *S*. *pombe* grown in glucose (GLU) and glycerol/acetate (GLY).

*Escherichia coli* GLU					
**RANKING**	**GS**	**ES**	**NES**	**NOM p-val**	**FDR q-val**
1	NITROGEN METABOLISM	47	0.74	2.6	0
2	**ALANINE ASPARTATE AND GLUTAMATE METABOLISM**	30	0.67	2.11	0
3	**ARGININE AND PROLINE METABOLISM**	43	0.52	1.77	0.005
4	**GLYCINE SERINE AND THREONINE METABOLISM**	32	0.54	1.73	0.002
5	SELENOCOMPOUND METABOLISM	17	0.61	1.7	0.016
6	AMINOBENZOATE DEGRADATION	10	0.69	1.69	0.023
7	**LYSINE BIOSYNTHESIS**	18	0.55	1.56	0.038
8	**BUTANOATE METABOLISM**	35	0.46	1.53	0.023
9	**CYSTEINE AND METHIONINE METABOLISM**	29	0.45	1.46	0.033
10	STARCH AND SUCROSE METABOLISM	32	0.45	1.46	0.044
11	GLYCOLYSIS_ GLUCONEOGENESIS	39	0.42	1.42	0.046
12	VALINE LEUCINE AND ISOLEUCINE BIOSYNTHESIS	20	0.47	1.37	0.093
13	GLUTATHIONE METABOLISM	18	0.49	1.36	0.099
***Schizosaccharomyces pombe* GLU**					
**RANKING**	**GS**	**ES**	**NES**	**NOM p-val**	**FDR q-val**
1	TRNA AND PROCESSING	197	0.9	3.66	0
2	AMINOACYL-TRNA BIOSYNTHESIS	201	0.87	3.61	0
3	RIBOSOME	206	0.72	2.98	0
4	60S RIBOSOMAL PROTEIN L10 AND ACIDIC	82	0.79	2.85	0
5	40S RIBOSOMAL PROTEIN	55	0.76	2.51	0
6	BIOSYNTHESIS OF AMINO ACIDS	98	0.62	2.31	0
7	5S RRNA	31	0.76	2.24	0
8	AMINOACID TRNA LIGASE	21	0.72	1.99	0
9	**ALANINE ASPARTATE AND GLUTAMATE METABOLISM**	26	0.68	1.97	0
10	**GLYCINE SERINE AND THREONINE METABOLISM**	25	0.68	1.92	0.004
11	BIOSYNTHESIS OF SECONDARY METABOLITES	209	0.46	1.91	0
12	**CYSTEINE AND METHIONINE METABOLISM**	29	0.63	1.84	0.004
13	RIBOSOME BIOGENESIS IN EUKARYOTES	108	0.49	1.82	0
14	TRANSLATIONS PROCESS	50	0.54	1.8	0
15	PURINE METABOLISM	83	0.5	1.79	0
16	2-OXOCARBOXYLIC ACID METABOLISM	28	0.6	1.77	0.004
17	**ARGININE AND PROLINE METABOLISM**	31	0.6	1.77	0.004
18	HEAT SHOCK PROTEINS	14	0.7	1.73	0.01
19	PYRIMIDINE METABOLISM	65	0.5	1.73	0.002
20	**LYSINE BIOSYNTHESIS**	13	0.71	1.72	0.01
21	PROTEASOME	34	0.57	1.72	0.008
22	PHENYLALANINE METABOLISM	10	0.75	1.69	0.017
23	PHENYLALANINE TYROSINE AND TRYPTOPHAN BIOSYNTHESIS	17	0.66	1.66	0.016
24	TYROSINE METABOLISM	19	0.62	1.64	0.013
25	SULFUR METABOLISM	15	0.64	1.6	0.028
26	WARBURG_ART	17	0.6	1.56	0.045
27	GLYOXYLATE AND DICARBOXYLATE METABOLISM	12	0.65	1.56	0.036
28	STEROID BIOSYNTHESIS	15	0.62	1.56	0.042
29	19S PROTEASOME	20	0.57	1.53	0.047
30	**BUTANOATE METABOLISM**	11	0.64	1.53	0.063
31	PROPANOATE METABOLISM	10	0.68	1.5	0.052
32	20S PROTEASOME	14	0.6	1.46	0.069
33	CHAPERONE RELATED PROTEINS	10	0.63	1.44	0.08
34	TERPENOID BACKBONE BIOSYNTHESIS	16	0.55	1.43	0.087
***Escherichia coli* GLY**					
**RANKING**	**GS**	**ES**	**NES**	**NOM p-val**	**FDR q-val**
1	FLAGELLAR ASSEMBLY	36	-0.77	-2.46	0
2	LIPOPOLYSACCHARIDE BIOSYNTHESIS	27	-0.72	-2.14	0
3	**GLYCEROPHOSPHOLIPID METABOLISM**	28	-0.6	-1.79	0
4	AMINOACYL-TRNA BIOSYNTHESIS	112	-0.46	-1.79	0
5	RNA POLYMERASE	11	-0.73	-1.76	0.002
6	AMINO SUGAR AND NUCLEOTIDE SUGAR METABOLISM	44	-0.5	-1.67	0.003
7	**CARBOHYDRATE TRANSPORT**	18	-0.56	-1.53	0.025
8	PORINS	10	-0.66	-1.52	0.043
9	THIAMINE METABOLISM	13	-0.59	-1.46	0.06
***Schizosaccharomyces pombe* GLY**					
**RANKING**	**GS**	**ES**	**NES**	**NOM p-val**	**FDR q-val**
1	WTF ELEMENT	20	-0.74	-2.04	0
2	**PLASMA MEMBRANE PROTEINS**	85	-0.53	-1.9	0
3	DUF999 FAMILY	10	-0.83	-1.86	0
4	GALACTOSE METABOLISM	16	-0.73	-1.85	0.004
5	RETROTRANSPOSABLE ELEMEN	12	-0.77	-1.8	0.006
6	PROTEIN MUG	30	-0.58	-1.69	0.006
7	**GLYCEROLIPID METABOLISM**	13	-0.67	-1.64	0.024
8	MEIOSIS PROTEINS	37	-0.51	-1.61	0.015
9	MATING TYPE	15	-0.62	-1.56	0.037
10	SPORE AND SPORE WALL	10	-0.65	-1.48	0.071

Gene Set Enrichment Analysis (GSEA) of enriched KEGG pathways upregulated in GLU (YE medium containing 2% glucose) and GLY (YE containing a mix of 2% glycerol and 0.2% sodium acetate) phenotypes. Gene sets in bold are those pathways similar in both conditions for the two organisms (FDR below 0.25 adjusted p-value < 0.05). Abbreviation: GS (Gene Set), ES (Enrichment score), NES (Normalized enrichment score), NOM p-val (Nominal p-value), FDR q-val (False Discovery Rate q- value).

**Fig 5:**
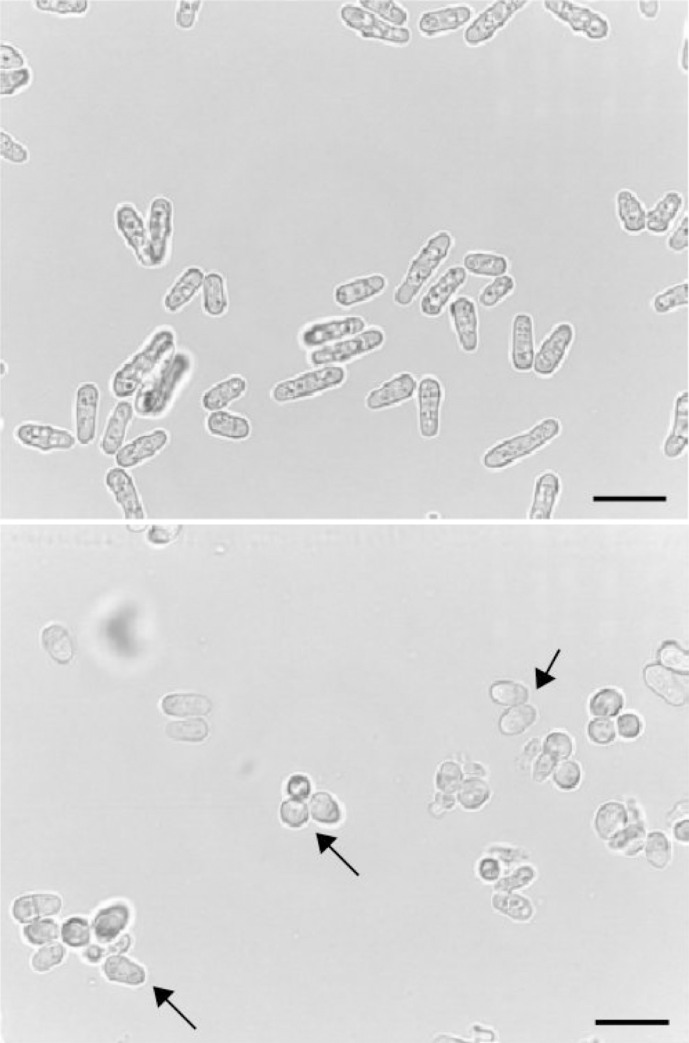
Microscopic analysis of cells cultured in YE medium with two different carbon sources. Wild-type *S*. *pombe* cells were incubated at 30°C to mid-log phase in YE containing 2% glucose (**a**) and YE medium containing 2% glycerol and 0.2% sodium acetate (**b**). Arrows in panel B mark cells displaying malformed and rounded shape. Length bar, 10 μm.

The GSEA analysis also showed that several groups of genes related to meiotic drivers (*wtf* element) [35], meiotic events and sporulation (protein mug) [36], mating type, membrane and cell wall, or like the Duf999 family involved in heterochromatin dynamics and fail-safe mechanisms to repair sub-telomeric double stranded breaks (DSB) [37], were enriched in fission yeast cultivated in glycerol/acetate (Table 1). Besides, the microscopic analysis of *S*. *pombe* grown in glycerol/acetate exhibited a change in cell morphology. Glucose-grown cells were larger and rod-shaped, the canonical structure described for fission yeast (Fig 5A). However, in glycerol/acetate, the cells were rounded, small, and some of them joined together, suggesting a change in cell wall morphology and integral membrane protein composition, in accordance with the transcriptome profile and the GSEA analysis (Fig 5B). These results are consistent with the observations previously described, showing a decrease in cell size and an increase in the generation time of *S*. *pombe* when the culture medium changes the carbon, nitrogen and even phosphate source [38]. In our study, the generation time in glycerol/acetate was approximately twice than in glucose.

Regarding central carbon metabolism, a conserved response was observed in both organisms. In bacteria, the *agaI*, *kbaY*, *agaD*, *agaB*, *ebgC*, *gatA*, *gatB*, *gatC*, and *gatD* genes, has been reported to be involved in the metabolism of glycerone-P, lactose, D-galactosamine 6-phosphate, tagatose and galactose, most of them subjected to Crp catabolite repression [39]. In our study, some of these genes were significantly differentially expressed in glycerol/acetate or with low levels of expression in glucose. Similarly, in fission yeast, some genes involved in galactose metabolism were overexpressed in glycerol/acetate condition, including GAL10 (UDP-glucose 4-epimerase/aldose 1-epimerase), GAL1 (galactokinase), MEL1 (alpha-galactosidase), GAL7 (galactose-1-phosphate uridylyltransferase), INV1 (external invertase beta-fructofuranosidase), and AGL1 (maltose alpha-glucosidase), most of which are also subjected to catabolite repression [40]. Moreover, we also observed the enrichment of gene sets related to the transport of hexoses in both organisms, the carbohydrate transport group in *E*. *coli* and the group of plasma membrane proteins in *S*. *pombe*. In both gene sets, hexose transporters with high affinity were overexpressed in glycerol/acetate (S1 File).

Together all of the above observations can be interpreted as a phenotype of "foraging behavior" as it stimulates the differentiation of cells and the use of alternative sources of nutrients, energy efficiency, and cell stress when both organisms are grown in glycerol/acetate.

### ncRNAs constitute a significant fraction of the genes differentially expressed in response to metabolic stress induced by glycerol growth in both organisms

In *E*. *coli* some ncRNAs are linked to the regulation of diverse biological processes like carbon metabolism as is the case of *cyaR*, which encodes an sRNA expressed under the action of CRP-cAMP and is subjected to catabolite repression [41]. In addition to *cyaR*, four other genes corresponding to sRNAs were up-regulated in glycerol/acetate: *rdlC* whose overexpression decreases swarming motility [42], *symR*, a very stable small RNA that is encoded on the opposite strand of the *symE* 5’ UTR and represses translation of *symE* involved in degradation and recycling of damaged RNA [43], *dsrA* that affects biosynthesis of capsular polysaccharide via increased production of the activator RcsA [44], and *oxyS*, which is involved in regulation of *fhlA* [45–47], *rpoS* [45,48,49], and *flhDC* [50]. The *flhDC* operon of *E*. *coli* encodes transcription factors that initiate flagellar synthesis, an energetically costly process that is tightly regulated (S1 File).

Interestingly, in *S*. *pombe*, the transcripts corresponding to ncRNA had a greater representation than metabolic and structural genes. We observed that 311 ncRNA genes were differentially expressed in glycerol/acetate compared to glucose, with 248 ncRNA genes induced and 63 repressed in glycerol/acetate. (S2 File). One of the ncRNAs detected in glycerol/acetate is the SPNCRNA.1324 gene, which is expressed antisense to *fbp1* that encodes fructose-1,6-bis-phosphatase and is transcriptionally repressed by glucose. Another interesting ncRNA expressed in glycerol/acetate is *sme2* (SPNCRNA.103), which has recently been shown to accumulate in the *sme2* chromosomal loci and mediates their robust meiosis pairing through the lncRNA–protein complexes assembled at specific chromosomal loci which mediate the recognition and subsequent pairing of homologous chromosomes [51].

## Discussion

Glucose has long been recognized as the preferred source of carbon for many microorganisms and defines a transcriptionally conserved response. Even so, many microorganisms may also use many other sources of carbon, including glycerol in the presence of external electron acceptors by means of respiratory metabolism [52,53]. Comparative expression profile studies have revealed coordinated gene expression evolution across eukaryote species, but it has been difficult to compare organisms classified into different kingdoms of life. This work aims to compare the behavior of two organisms, *E*. *coli* and *S*. *pombe*, using similar growth conditions in two different metabolic scenarios, to elucidate some of the conserved responses between prokaryotes and eukaryotes and the particular adaptations evolved to contend with different carbon sources.

Using RNA-seq technology, we compared the transcriptome of bacteria and fission yeast in two metabolic scenarios, growing organisms in the YE medium with glucose or glycerol/acetate as the principal carbon source. To decrease the variations, it is important to say that the only significant difference was the growth temperature. Our analyses showed that of the 4242 and 5132 proteins present in *E*. *coli* and *S*. *pombe*, respectively, 718 orthologous and 262 co-orthologs are conserved. The numbers in the Venn diagram (Fig 1) represent the number of orthologous clusters that *E*. *coli* shares with *S*. *pombe*. The diagram shows that 343 gene clusters are shared by both species, suggesting their conservation in the lineage after speciation. Additionally, it shows that there were 385 clusters specific to *E*. *coli* and 436 to *S*. *pombe*. We applied OrthoVenn clustering to identify gene clusters enriched in in both species and only the tricarboxylic and the glycolytic processes were enriched. The presence of specific clusters suggests that there might be a lineage specific gene expansion in these gene families in both species. Based on annotation of these clusters, some of these lineage specific clusters are involved in biological processes specific for each organism (e.g., bacterial-type flagellum hook for *E*. *coli* or negative regulation of ascospore formation for *S*. *pombe*).

Our comparison revealed that in both organisms, glucose promotes cell proliferation and biomass formation, while a gluconeogenic source, such as glycerol, activates typical respiratory metabolism genes, such as glyceraldehyde phosphate dehydrogenase, and induces changes in cell morphology. These common points reinforce the notion that the response to the same stimulus (growth condition) in phylogenetically distant species preserves a pattern where transcriptional response plays a central role.

In both organisms, a conserved response has been observed in central carbon metabolism. In bacteria, the expression of *agaI*, *kbaY*, *agaD*, *gatA*, *ebgC*, *agaB*, *gatB*, *gatC*, and *gatD* genes has been reported to be involved in the production of glycerone-P, lactose, D-galactosamine 6-phosphate, and tagatose, most of them subjected to Crp catabolite repression [39]. Similarly, some genes involved in galactose metabolism in fission yeast were overexpressed in glycerol/acetate, including GAL10 (UDP-glucose 4-epimerase/aldosis 1-epimerase), GAL1 (galactokinase), MEL1 (alpha-galactosidase), GAL7 (galactose-1-phosphate uridylyltransferase), INV1 (external invertase beta-fructofuranosidase), and AGL1 (maltose alpha-glucosidase), the majority of which are also subjected to catabolite repression [54]. Nonetheless, at the glucose concentrations used in the comparison study, the effect of catabolic repression is more pronounced in fission yeast than in *E*. *coli*.

The findings related to flagellum formation in *E*. *coli* are directly in line with previous studies where the responses to gluconeogenic carbon sources, such as glycerol, succinate, and glycine, correspond to the "foraging behavior” described by Liu and coworkers [55]. As carbon substrate quality decreases (defined by growth rate) *E*. *coli* systematically increases the number of genes expressed in a hierarchical manner and also increases their motility by production of flagella. In terms of the number of genes and the energy required for flagellar biosynthesis and functioning, this process is very expensive for the cellular economy and would trigger a high risk of exhausting the sole energy supply more rapidly. It seems that bacteria take a risk and actively seek out better conditions using the flagellar system [56]. The mechanistic and signal transduction models of the chemotactic responses in bacteria and archaea are based largely on studies with *E*. *coli* methylated chemotaxis proteins (MCPs). There are five MCPs in this organism: Tar (transducer for aspartate), Tap (transducer for peptides), Aer (the aerotaxis MCP), Tsr (transducer for serine), and Trg (transducer for ribose and galactose). Attractant or repellent binding to an MCP induces conformational changes that are transmitted via CheW to CheA, the histidine sensor kinase of the chemotaxis system [57]. In our data, only *trg* was differentially expressed with a log2FC of -1.193. This result is consistent with what was observed by Fitzgerald *et al* [34], where the induction of flagellar swarming by FliA, induces a complex transcriptional network involving many synchronized events, such as the expression of regulatory genes (*flhDC*), basal body and hook genes, *fliLMNOPQR* operon motor and hook-associated protein, the flagellar sigma factor σ^28^, and *trg*, all of which are significantly overexpressed in glycerol/acetate. A similar response has been detected in *Pectobacterium atrosepticum* when comparing surface swarming in an agar plate containing glycerol and glucose. In this species, swarming is promoted by H-NS2, by quorum sensing in the absence of glucose, via *hexY*, a small protein that is thought to bind and sequester FlhD_4_C_2_ complex. A mutant strain of *hexY* displayed a hyper-swarming phenotype [58].

In fission yeast, several groups of genes related to meiosis, membrane and cell wall were highly expressed in glycerol/acetate. Meiotically upregulated genes (*mug*) was another overexpressed group. These genes were discovered based on microarray expression analysis, and are induced during chromosome segregation. These proteins are associated with cellular stress or genes related to recognition and signaling, and prepare *S*. *pombe* for the diploid formation and meiosis. Phenotypes of several *mug* gene mutant strains include lethality, smaller cell, aberrant cells or reduction in mating [36]. In our analysis, members of the enriched *mug* proteins gene set included middle and early genes and most of them do not have an apparent phenotype except for *mug146* (many abnormal spores) and *mug168* (resisted deletion). These findings indicate that fission yeast cells are in a process of entry to mating-type switch, diploid formation, and meiosis cycle. However, *S*. *pombe* h- 972 is unable to form diploids because it has a deletion in the *mat2*-P gene.

Meiosis, although an event during sexual reproduction, is also induced in nitrogen deficient media, and the literature describes that it allows DNA repair, homologous recombination events, and adaptive survival advances [59]. Remarkably, several Duf999 family proteins, involved in rapidly and efficiently healing double-strands breaks near sub-telomeres and to maintain cell viability, were overexpressed in glycerol/acetate. Therefore, under certain assumptions, this can be interpreted as a “foraging behavior” response, since it stimulates cell differentiation and the use of alternative nutrient sources, energy efficiency, and cell stress.

An interesting feature observed in both species reveals that when grown in glycerol/acetate rather than glucose, there was a greater expression of ncRNAs genes. ncRNAs can regulate gene expression at post and transcriptional stages. More than 170 ncRNAs have been described in *E*. *coli* and some of those observed in this work have been validated in the literature in response to cellular stress, such as *ryhB* and *micF*. These sRNAs regulate expression of proteins linked to oxidative and membrane envelope stresses. The sRNA with the highest fold change in our study was *cyaR* (log2FC -2.37). The transcript of this gene is stabilized by Hfq, promoting degradation of the *ompX* [41,60], *yqaE*, *nadE*, and *luxS* mRNAs [41]. Its expression depends on the combinatorial effect of the σ ^E^ sigma factor and the two-component CpxAR system [61]. The other sRNAs that were over-expressed in glycerol/acetate were: *rdlC* (log2FC -1.19), whose overexpression decreases swarming motility [42]); *symR* (log2FC -1.14), a very stable small RNA that is encoded on the opposite strand of the *symE* 5’ UTR and represses translation of *symE* [43]; *dsrA* (log2FC -1.13), which affects biosynthesis of capsular polysaccharides via increased production of the activator RcsA [44], which showed a log2FC of -1.69; and *oxyS* (log2FC -1.08) that is involved in regulation of *fhlA* [45–47], *rpoS* [45,48,49], *flhDC* [50], and *fliA* (σ^F^ sigma factor). *fliA* regulates genes related to the expression of the flagellum and other transcriptional units, such as the *flhDC* operon (log2FC -2.77 and -2.13, respectively) of *E*. *coli*, which encodes transcription factors that initiate flagellar synthesis, an energetically costly process that is highly regulated and the *glpABD* operon, which facilitates the uptake of glycerol. Also, *fliA*, like σ^E^ sigma factor, competes for the binding sites of σ^70^ sigma factor [34]. Hence, we observed that most of the expression values for sRNAs and their regulated genes have the expected expression pattern.

For *S*. *pombe*, 1836 ncRNA genes were reported in the Pombase database. However, 5775 novel *lncRNAs* were recently reported by Atkinson *et al* [62]. based on RNA-seq results. The presence of Dicer and the expression of long ncRNAs in fission yeast indicates clear post-transcriptional gene regulation [63]. Most ncRNAs have not been identified as regulators or precursors for small ncRNAs [64], although some RNA-seq studies have revealed the presence of ncRNAS transcripts on vegetative development, meiotic division, and nutritionally restricted quiescent cells. Nevertheless, some of those found in glycerol/acetate are involved in the regulation of transcripts such as SPNCRNA.1324 (antisenses-*fbp1*) implicating gluconeogenesis [65], while others are specifically associated with cell stress, such as SPNCRNA.1164 [66]. Recently, it was stated that *sme2*, another ncRNA expressed in glycerol/acetate, forms lncRNA–protein complexes with Smp proteins assembled to tether homologs at different chromosomal loci [51]. To deepen our understanding of the role of these transcripts, we are currently analyzing two RNA-seq libraries specifically designed to evaluate the expression of ncRNAs in *S*. *pombe* under the same experimental conditions as in this study.

To conclude, transcriptome analyses carried out in this work comparing two different microorganisms in a similar environment changing only the carbon source, allowed us to infer that the metabolic response to glucose is usually maintained in two species that belong to separate life domains. Our results extend previous reports showing that analogous genes related to catabolite repression and associated with “foraging behavior” in both species are overexpressed when glucose is absent and another carbon source such as glycerol acetate is utilized. Interestingly, in this condition, the induction of the ncRNAs expression is preserved in both organisms, reinforcing the idea that these molecules are essential for regulating genes involved in stress conditions. This study serves as a framework for future research on functional comparative genomics between the three of domains of life: Eubacteria, Archaea, and Eukaryotes.

## Ethics approval

The authors have no ethical conflicts to disclose since neither human beings nor animals were involved in our research.

## Supporting information

S1 Fig Growth curves of E. coli and S. pombe in ME medium supplemented with glucose or glycerol/acetate. (**a**) Growth of *E*. *coli* MG1655 in YE medium with 2% (m/w) glucose (∙) and 2%/0.2% (m/w) glycerol acetate (**■**) while shaking to 250 rpm at 37°C. Error bars indicate one standard error of the mean of three biological replicates. (**b**)Growth of *S*. *pombe* 972 h^-^ in YE medium with 2% (m/w) glucose (∙) and 2%/0.2% (m/w) glycerol acetate (**■**) while shaking to 250 rpm at 30 C. Error bars indicate one standard error of the mean of three biological replicates.
(TIF)

S2 Fig Volcano plot of RNA-seq data in *E*. *coli* and *S*. *pombe*. The data for 4419 genes in *E*. *coli* (**a**) and 6862 genes in fission yeast (**b**) were plotted as log2 fold change versus the −log10 of the *p*-value. Thresholds are shown as dashed lines. Genes selected as significantly different are highlighted as red dots.
(TIF)

S1 Table STRING functional enrichment analysis of GO categories; KEGG pathways and LNS terms. STRING functional enrichment analysis of several biological terms of the STRING database using all gene expression profile ranked by Log2FC values and using a lenient FDR astringency (< 0.25) in *Schizosaccharomyces pombe* (a) and *Escherichia coli* (b) grown in glucose (GLUC) and glycerol/acetate (GLYC).
(XLSX)

S2 Table STRING functional enrichment analysis of GO categories; KEGG pathways and LNS terms. STRING functional enrichment analysis of several biological terms of the STRING database using exclusively the orthologs identified by OrthoVenn and PANTHER database in *S*. *pombe* and *E*. *coli*. Orthologs of *S*. *pombe* (a) and *E*. *coli* (b) grown in glucose (GLUC) and glycerol/acetate (GLYC) were ranked by Log2FC values for the analysis and using a lenient FDR astringency (< 0.25).
(XLSX)

S3 Table Ortholog clusters of *E*. *coli* and *S*. *pombe* identified by OrthoVenn and complemented with the information of ortholog genes predicted by PANTHER. (XLSX)

S1 File EdgeR analysis of *E*. *coli* MG1655 grown in YE medium with 2% (m/w) glucose (∙) and 2%/0.2% (m/w) glycerol acetate. (PDF)

S2 File EdgeR analysis of *S*. *pombe* grown in YE medium with 2% (m/w) glucose (∙) and 2%/0.2% (m/w) glycerol acetate. (PDF)
