# Supplementary figures and images for: High-throughput transcriptome sequencing and comparative analysis of Escherichia coli and Schizosaccharomyces pombe in respiratory and fermentative growth
Source: PLoS One. 2021 Mar 17;16(3):e0248513. doi: 10.1371/journal.pone.0248513 (PMC7968713; doi:10.1371/journal.pone.0248513)

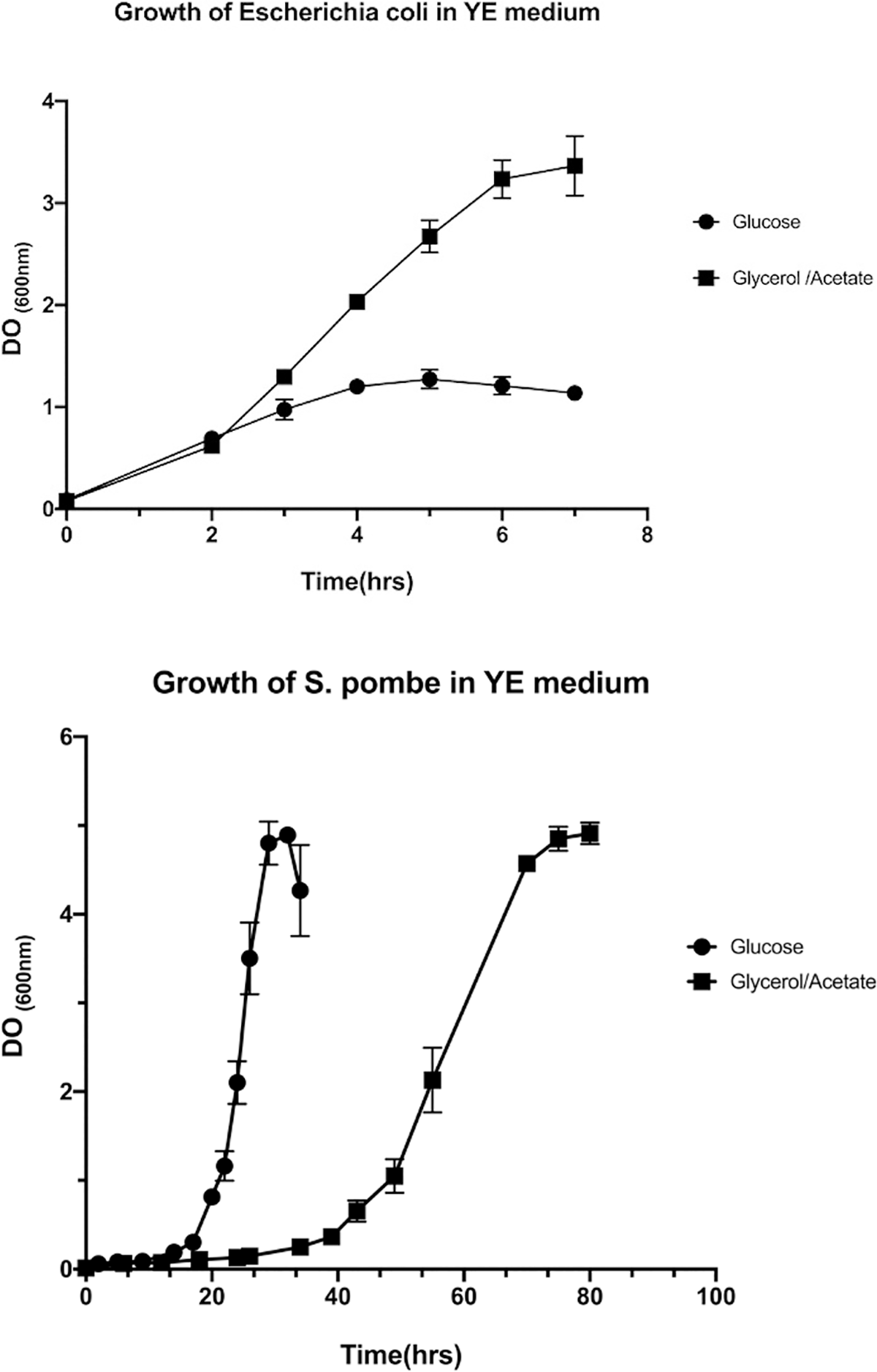

Supplement: S1 Fig — (a) Growth of E. coli MG1655 in YE medium with 2% (m/w) glucose (∙) and 2%/0.2% (m/w) glycerol acetate (■) while shaking to 250 rpm at 37°C. Error bars indicate one standard error of the mean of three biological replicates. (b)Growth of S. pombe 972 h- in YE medium with 2% (m/w) glucose (∙) and 2%/0.2% (m/w) glycerol acetate (■) while shaking to 250 rpm at 30 C. Error bars indicate one standard error of the mean of three biological replicates. (TIF) [file pone.0248513.s001.tif]

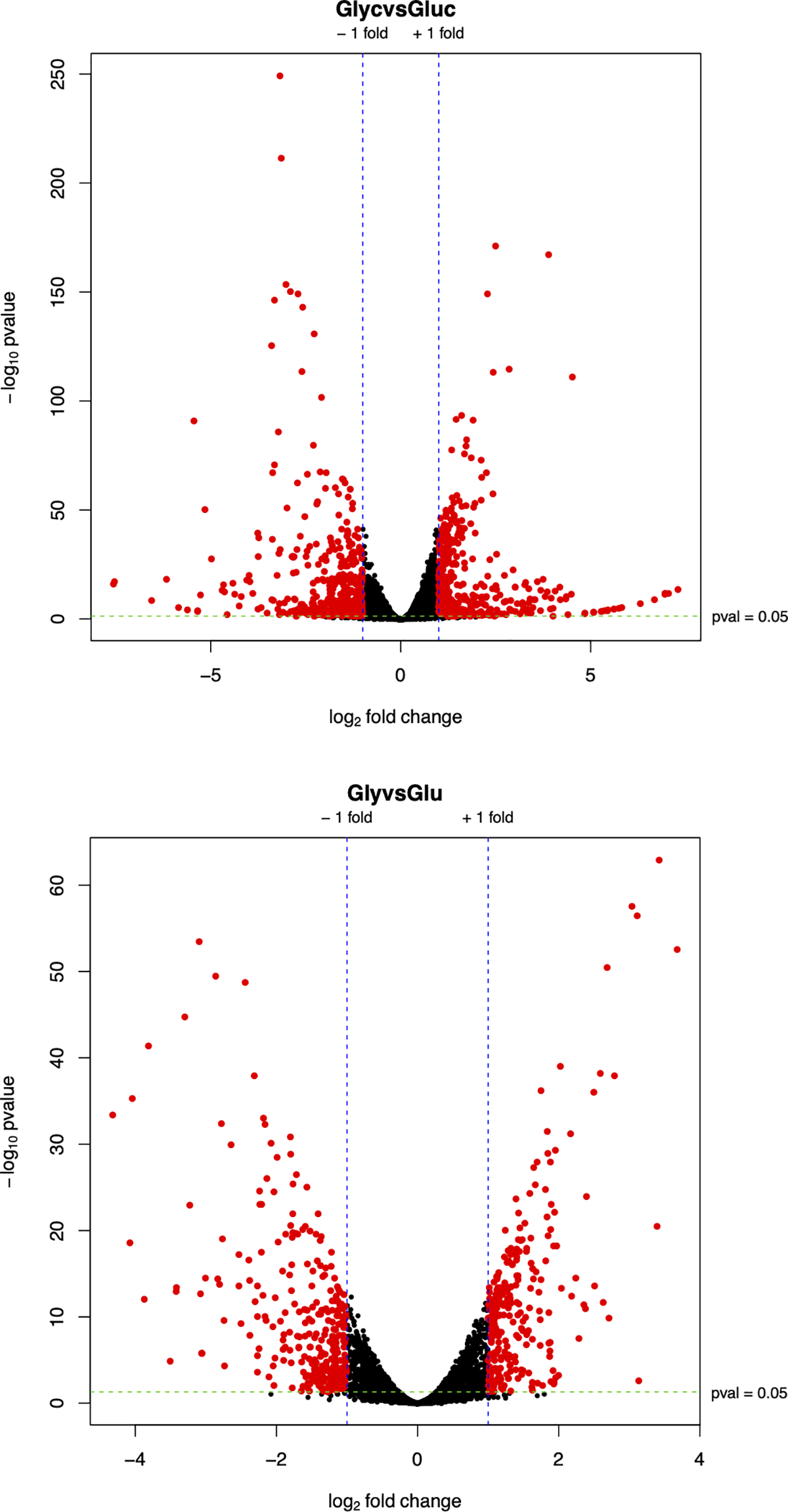

Supplement: S2 Fig — The data for 4419 genes in E. coli (a) and 6862 genes in fission yeast (b) were plotted as log2 fold change versus the −log10 of the p-value. Thresholds are shown as dashed lines. Genes selected as significantly different are highlighted as red dots. (TIF) [file pone.0248513.s002.tif]
