# Supplementary material for: High-throughput transcriptome sequencing and comparative analysis of Escherichia coli and Schizosaccharomyces pombe in respiratory and fermentative growth
Source: PLoS One. 2021 Mar 17;16(3):e0248513. doi: 10.1371/journal.pone.0248513 (PMC7968713; doi:10.1371/journal.pone.0248513)
Supplement: S1 File — (PDF) [file pone.0248513.s006.pdf]

ID Gene	Gene Name	Product	logFC	logCPM	PValue	FDR
b4245	pyrB	aspartate carbamoyltransferase catalytic subunit	3.681880281	8.47667086	2.77E-53	2.44E-50
b4244	pyrI	aspartate carbamoyltransferase, PyrI subunit	3.426041889	7.677821001	1.06E-63	4.68E-60
b0873	hcp	protein S-nitrosylase	3.395771905	6.516926166	3.29E-21	2.55E-19
b1503	ydeR	putative fimbrial protein YdeR	3.137467493	1.774759116	0.002282957	0.008512883
b3365	nirB	nitrite reductase catalytic subunit NirB	3.112886955	10.07369669	3.37E-57	4.96E-54
b2169	fruB	FruB - his299 phosphorylated	3.041481476	8.610235515	2.46E-58	5.43E-55
b3367	nirC	NIRC-MONOMER	2.792173286	6.979022425	1.19E-38	3.77E-36
b0872	hcr	G6456-MONOMER	2.714483614	5.703499001	1.36E-10	2.30E-09
b3366	nirD	nitrite reductase subunit NirD	2.687721087	7.122739882	3.12E-51	2.30E-48
b1466	narW	NarW, putative private chaperone for NarZ nitrate reductase subunit	2.631085213	4.548612648	1.81E-12	4.35E-11
b2168	fruK	1-phosphofructokinase	2.59015957	8.340064433	5.53E-39	2.03E-36
b2208	napF	ferredoxin-type protein	2.510323337	5.28471634	2.59E-14	8.47E-13
b0674	asnB	asparagine synthetase B	2.498495836	9.12655223	9.84E-37	2.71E-34
b1467	narY	nitrate reductase Z subunit β	2.396286623	6.034454524	1.04E-24	1.07E-22
b4070	nrfA	cytochrome csub552	2.381959541	6.819841215	1.12E-11	2.29E-10
b1746	astD	aldehyde dehydrogenase	2.359566067	5.763088585	3.40E-12	7.58E-11
b1465	narV	nitrate reductase Z subunit γ	2.287279119	4.163271905	2.82E-08	3.28E-07
b1747	astA	ARGSUCCTRAN-MONOMER	2.243167029	5.946250166	3.17E-15	1.16E-13
b1745	astB	N-succinylarginine dihydrolase	2.18623143	5.45637213	3.51E-13	9.34E-12
b0919	ycbJ	putative phosphotransferase YcbJ	2.168721056	7.358687492	5.25E-32	1.01E-29
b1751	ydjY	4Fe-4S ferredoxin-type domain-containing protein YdjY	2.040593307	5.660608804	4.34E-14	1.31E-12
b1593	ynfK	putative dethiobiotin synthetase	2.025506922	7.33573746	9.40E-40	3.77E-37
b0558	ybcV	DLP12 prophage; DUF1398 domain-containing protein YbcV	2.000684782	4.165592506	0.000548467	0.002411835
b2847	yqeI	putative transcriptional regulator YqeI	1.97356994	7.049986798	6.05E-19	3.34E-17
b1569	dicC	Qin prophage; DNA-binding transcriptional regulator for DicB	1.959269919	2.66633071	0.001035037	0.004211694
b3744	asnA	asparagine synthetase A	1.954233285	9.120652985	4.52E-30	7.39E-28
b3447	ggt	glutathione hydrolase, small subunit	1.943391614	6.735129808	7.43E-23	6.69E-21
b1690	ydiM	putative exporter YdiM	1.936304949	2.807957226	0.003646357	0.01270613
b1700	ydiT	ferredoxin-like protein YdiT	1.931908821	3.064693306	0.005973128	0.019491026
b1684	sufA	iron-sulfur cluster insertion protein SufA	1.930242531	6.537056096	5.17E-19	2.89E-17
b1674	ydhY	putative 4Fe-4S ferredoxin-like protein YdhY	1.922234462	3.906638365	0.000147044	0.000784056
b1468	narZ	nitrate reductase Z subunit α	1.896811602	7.361051282	8.34E-24	8.18E-22
b1488	ddpX	G6782-MONOMER	1.891563343	2.70775858	0.002741371	0.009961441
b4336	yjiN	DUF445 domain-containing protein YjiN	1.887366556	6.606338786	7.04E-21	5.18E-19
b3212	gltB	glutamate synthase subunit GltB	1.884502685	10.01215511	1.09E-28	1.55E-26
b1744	astE	SUCCGLUDESUCC-MONOMER	1.879882206	4.744188965	8.83E-08	9.35E-07
b0992	yccM	putative 4Fe-4S membrane protein	1.876818014	5.590705208	8.39E-07	7.44E-06
b0574	cusB	coppersilver export system membrane fusion protein	1.873530353	4.346750478	3.73E-06	2.88E-05
b0514	glxK	glycerate 2-kinase 2	1.864452426	5.357802009	3.29E-11	6.18E-10
b2207	napD	NapA signal peptide-binding chaperone NapD	1.864378137	4.691445753	9.49E-08	9.91E-07
b4154	frdA	fumarate reductase flavoprotein subunit	1.850588997	9.009560735	4.05E-20	2.63E-18
b2167	fruA	fructose-specific PTS multiphosphoryl transfer protein FruA	1.846915287	8.882386444	1.19E-29	1.87E-27
b1683	sufB	Fe-S cluster scaffold complex subunit SufB	1.841538183	8.19055404	3.15E-32	6.31E-30
b1895	uspC	universal stress protein C	1.836613102	6.512200736	2.43E-22	2.02E-20
b2130	yehY	glycine betaine ABC transporter membrane subunit YehY	1.81849188	6.180077907	3.02E-17	1.37E-15
b3417	malP	maltodextrin phosphorylase	1.813014822	8.543347648	1.80E-25	2.04E-23

b1455	yncH	DUF5445 domain-containing protein YncH	1.797181461	1.872777884	0.078401944	0.165145317
b1310	ycjN	putative ABC transporter periplasmic binding protein YcjN	1.774226646	2.495033026	0.007826539	0.024558756
b0451	amtB	ammoniaammonium transporter	1.752294996	7.74719289	5.51E-37	1.62E-34
b0894	dmsA	dimethyl sulfoxide reductase subunit A	1.750868294	7.706241541	4.32E-15	1.51E-13
b4071	nrfB	periplasmic nitrite reductase penta-heme c-type cytochrome	1.741192318	5.558985453	1.87E-07	1.85E-06
b1748	astC	succinylornithine transaminase	1.738486543	6.576164124	2.14E-10	3.52E-09
b2468	aegA	putative oxidoreductase Fe-S subunit AegA	1.730783371	6.783641349	1.85E-11	3.59E-10
b2141	yohJ	PF03788 family membrane protein YohJ	1.721274648	5.960995814	1.36E-13	3.80E-12
b1689	ydiL	DUF1870 domain-containing protein YdiL	1.704138247	2.911386157	0.004477686	0.015125466
b2166	psuK	putative pseudouridine kinase	1.698899828	1.650435053	0.124545499	0.23733237
b1668	ydhS	FADNAD(P) binding domain-containing protein YdhS	1.694834474	7.483407346	1.14E-28	1.58E-26
b2727	hypB	hydrogenase isoenzymes nickel incorporation protein HypB	1.681026306	7.317532799	5.27E-16	2.10E-14
b2903	gcvP	glycine decarboxylase	1.670523583	9.84323839	4.23E-26	5.04E-24
b1188	ycgB	PF04293 family protein YcgB	1.647895411	7.910449421	4.94E-28	6.61E-26
b1932	yedL	putative acetyltransferase YedL	1.64368232	6.18667534	3.58E-15	1.28E-13
b2206	napA	periplasmic nitrate reductase subunit NapA	1.632659986	7.305403858	2.65E-16	1.08E-14
b2848	yqeJ	protein YqeJ	1.630840592	6.275459922	4.02E-08	4.55E-07
b1311	ycjO	putative ABC transporter membrane subunit YcjO	1.628604033	2.308842654	0.027961259	0.072276906
b2339	yfcV	putative fimbrial protein YfcV	1.627775931	2.896432431	0.00347058	0.012228741
b1691	ydiN	putative transporter YdiN	1.621676368	2.620725332	0.011816215	0.03508097
b2142	yohK	PF04712 family membrane protein YohK	1.609079475	6.629458086	5.95E-17	2.63E-15
b1784	yeaH	DUF444 domain-containing protein YeaH	1.607713903	7.155843611	7.71E-20	4.80E-18
b1988	nac	DNA-binding transcriptional dual regulator Nac	1.602939692	6.437623635	4.60E-14	1.38E-12
b1619	hdhA	7-α-hydroxysteroid dehydrogenase	1.592641563	8.30639078	4.24E-25	4.46E-23
b2131	osmF	glycine betaine ABC transporter periplasmic binding protein OsmF	1.586950327	6.322375309	9.30E-12	1.94E-10
b1314	ycjR	putative ketohexose-3-epimerase YcjR	1.577035417	3.620467568	0.000297817	0.001429198
b1693	aroD	3-dehydroquinate dehydratase	1.57530603	5.658710238	3.84E-09	5.20E-08
b2302	yfcG	disulfide reductase	1.548867075	5.806091543	1.01E-08	1.31E-07
b2292	yfbS	putative transporter YfbS	1.546754244	7.370073064	9.98E-19	5.44E-17
b1217	chaB	putative cation transport regulator ChaB	1.54485828	5.036087491	1.46E-07	1.49E-06
b3213	gltD	glutamate synthase subunit GltD	1.544783479	8.976784573	2.47E-18	1.27E-16
b0450	glnK	uridylyl-[GlnK]	1.532204124	6.059219391	1.13E-10	1.94E-09
b2913	serA	phosphoglycerate dehydrogenase	1.521877298	9.198323516	1.30E-21	1.07E-19
b1793	yoaF	DUF333 domain-containing lipoprotein YoaF	1.49717536	5.849484455	3.07E-10	4.88E-09
b2203	napB	periplasmic nitrate reductase cytochrome cSUB550SUB protein	1.495301342	4.082962275	0.000144222	0.000769938
b1601	tqsA	G6859-MONOMER	1.491137316	5.74965665	3.93E-09	5.31E-08
b1449	curA	NADPH-dependent curcumindihydrocurcumin reductase	1.483892425	7.220447477	1.08E-19	6.46E-18
b4072	nrfC	putative menaquinol-cytochrome c reductase 4Fe-4S subunit	1.478261623	5.039783996	0.000417736	0.001893535
b2347	yfdC	inner membrane protein YfdC	1.474804074	6.055930634	2.14E-09	3.03E-08
b1882	cheY	CheY-acetylated	1.473212572	4.280046128	0.000136471	0.000734781
b1681	sufD	Fe-S cluster scaffold complex subunit SufD	1.461475081	7.789668811	1.26E-19	7.29E-18
b3158	yhbU	putative peptidase YhbU	1.458667587	7.321647522	2.91E-11	5.53E-10
b4051	qorA	putative quinone oxidoreductase 1	1.452561609	7.581687505	4.42E-21	3.37E-19
b0002	thrA	fused aspartate kinasehomoserine dehydrogenase 1	1.444061925	7.880453681	2.43E-18	1.26E-16
b3522	yhjD	putative transporter YhjD	1.437776613	6.621096598	3.11E-12	6.97E-11
b3416	malQ	4-α-glucanotransferase	1.432794097	8.319189565	8.41E-23	7.43E-21
b2129	yehX	glycine betaine ABC transporter ATP binding subunit YehX	1.432412197	6.170761395	2.32E-08	2.75E-07

b2662	gabT	4-aminobutyrate aminotransferase GabT	1.429668087	7.587827459	1.07E-19	6.46E-18
b4187	aidB	putative acyl-CoA dehydrogenase AidB	1.427308169	9.257818526	1.59E-15	6.02E-14
b1313	ycjQ	putative zinc-binding dehydrogenase YcjQ	1.424337431	3.555285871	0.001169483	0.004672639
b1223	narK	nitrate:nitrite antiporter NarK	1.424052831	8.815451158	4.80E-15	1.67E-13
b3073	patA	putrescine aminotransferase	1.420636527	7.084324981	5.48E-11	1.00E-09
b0077	ilvI	acetolactate synthase acetohydroxybutanoate synthase, catalytic subur	1.408013411	6.937216351	1.43E-18	7.61E-17
b1682	sufC	Fe-S cluster scaffold complex subunit SufC	1.40799292	6.977986838	1.85E-12	4.42E-11
b3524	yhjG	AsmA family protein YhjG	1.407516325	7.822611759	1.59E-15	6.02E-14
b0486	ybaT	putative transporter YbaT	1.406857632	8.82292374	1.11E-17	5.29E-16
b0078	ilvH	acetolactate synthase acetohydroxybutanoate synthase, regulatory sub	1.403629981	5.698861695	1.28E-08	1.61E-07
b2497	uraA	uracil:H+ symporter UraA	1.402329248	7.712455155	2.35E-17	1.09E-15
b1678	ldtE	L,D-transpeptidase LdtE	1.396482675	7.715346394	1.86E-24	1.86E-22
b1783	yeaG	protein kinase YeaG	1.39464714	8.925731185	1.99E-14	6.65E-13
b0621	dcuC	anaerobic C4-dicarboxylate transporter DcuC	1.393663355	6.29454307	0.000133869	0.000722532
b4364	yjjP	putative succinate exporter YjjP	1.389262468	4.978250132	1.46E-05	9.92E-05
b0643	ybeL	DUF1451 domain-containing protein YbeL	1.388001558	7.475968819	3.73E-18	1.85E-16
b0895	dmsB	dimethyl sulfoxide reductase subunit B	1.379682635	5.478084594	3.41E-06	2.67E-05
b1687	ydiJ	putative FAD-linked oxidoreductase	1.35353852	8.438424441	3.97E-10	6.22E-09
b1454	yncG	putative glutathione S-transferase YncG	1.349532905	5.249353187	4.37E-06	3.34E-05
b4269	ahr	aldehyde reductase, NADPH-dependent	1.346543299	7.875455169	7.52E-17	3.25E-15
b1004	wrbA	NAD(P)H:quinone oxidoreductase	1.34235536	8.506199584	8.19E-17	3.51E-15
b0925	ldtD	L,D-transpeptidase LdtD	1.329692133	8.757896539	3.07E-14	9.75E-13
b2661	gabD	NADP+-dependent succinate-semialdehyde dehydrogenase	1.32477653	7.964110619	1.10E-18	5.90E-17
b1955	yedP	putative mannosyl-3-phosphoglycerate phosphatase	1.324342482	6.93112086	2.26E-12	5.19E-11
b1615	uidC	outer membrane porin family protein UidC	1.321335462	2.724608136	0.035271254	0.087780488
b0287	yagU	inner membrane protein that contributes to acid resistance	1.320739643	7.411407542	5.65E-18	2.77E-16
b1587	ynfE	putative selenate reductase YnfE	1.319873475	5.755796362	0.00019786	0.001005239
b1648	ydhL	DUF1289 domain-containing protein YdhL	1.315976855	4.838100945	2.77E-05	0.000177207
b2157	yeiE	putative LysR-type transcriptional regulator YeiE	1.314075661	6.61385825	1.44E-12	3.50E-11
b2294	yfbU	UPF0304 family protein YfbU	1.313423849	7.099196548	6.31E-13	1.61E-11
b1752	ydjZ	DedA family protein YdjZ	1.310353896	5.255486685	4.88E-05	0.000298713
b1428	ydcK	putative enzyme YdcK	1.308445022	6.720326855	3.04E-13	8.18E-12
b4188	yjfN	protease activator	1.303166733	6.387821061	6.17E-11	1.11E-09
b2162	rihB	pyrimidine-specific ribonucleoside hydrolase RihB	1.302687621	2.155485917	0.061764959	0.136619387
b0572	cusC	coppersilver export system outer membrane channel	1.296867743	5.289143044	2.19E-06	1.78E-05
b3440	yhhX	putative oxidoreductase YhhX	1.290851218	8.042467182	2.98E-17	1.37E-15
b1443	ydcV	putative ABC transporter membrane subunit YdcV	1.287696231	4.204308945	0.001348732	0.005297733
b0573	cusF	coppersilver export system periplasmic binding protein	1.287302122	4.229980026	0.001230399	0.004889479
b4079	fdhF	formate dehydrogenase H	1.287064171	6.750296281	7.98E-12	1.68E-10
b4214	cysQ	EG10043-MONOMER	1.28471673	7.324577744	1.98E-18	1.04E-16
b3441	yhhY	N-acetyltransferase YhhY	1.282518183	5.529469777	6.26E-06	4.63E-05
b0972	hyaA	hydrogenase 1 small subunit	1.278470263	8.407668586	6.29E-13	1.61E-11
b2905	gcvT	GCVT-MONOMER	1.277624915	8.901897925	7.52E-14	2.20E-12
b4156	yjeM	putative transporter YjeM	1.270709972	7.330257264	4.50E-17	2.01E-15
b2301	yfcF	glutathione S-transferase YfcF	1.2696994	6.069413737	2.35E-07	2.31E-06
b4310	nanM	N-acetylneuraminate mutarotase	1.265966483	4.482989074	0.000482139	0.002150145
b2728	hypC	hydrogenase 3 maturation protein HypC	1.260930579	5.788551922	3.78E-06	2.91E-05

b0708	phr	deoxyribodipyrimidine photolyase (photoreactivation)	1.256609982	7.032719433	2.43E-14	8.07E-13
b2249	yfaY	IPR008135 CinA family protein YfaY	1.252808377	7.741853785	3.18E-14	9.97E-13
b0186	ldcC	lysine decarboxylase 2	1.249518271	8.493379101	9.73E-14	2.75E-12
b2010	dacD	D-alanyl-D-alanine carboxypeptidase DacD	1.249452097	6.565309782	5.83E-08	6.38E-07
b2356	yfdM	CPS-53 (KpLE1) prophage; putative methyltransferase	1.247898648	1.768677917	0.226400394	0.37144472
b2361	yfdR	CPS-53 (KpLE1) prophage; 5'-deoxynucleotidase	1.245730525	2.024549566	0.124268992	0.237201728
b2976	glcB	MALSYNG-MONOMER	1.245211731	7.847146001	9.07E-21	6.56E-19
b2552	hmp	nitric oxide dioxygenase	1.244465021	7.204000774	2.76E-14	8.85E-13
b2009	sbmC	DNA gyrase inhibitor	1.239758116	6.779908738	2.22E-12	5.16E-11
b1896	otsA	TREHALOSE6PSYN-MONOMER	1.236207758	8.091199991	1.05E-14	3.57E-13
b0495	ybbA	putative ABC transporter ATP-binding protein YbbA	1.235890825	5.889449189	9.43E-07	8.31E-06
b2904	gcvH	lipoyl-GcvH-protein	1.232559508	7.795169451	1.09E-17	5.24E-16
b1694	ydiF	putative acetate-CoA transferase	1.229284925	3.117297418	0.098198548	0.197605555
b1396	paaI	phenylacetyl-CoA thioesterase	1.228088166	3.546520745	0.008716772	0.026968848
b0789	clsB	cardiolipin synthase B	1.221193777	7.050863466	5.67E-11	1.03E-09
b0973	hyaB	hydrogenase 1 large subunit	1.220292002	8.803720253	4.57E-12	9.95E-11
b0871	poxB	pyruvate oxidase	1.212126094	9.054741774	3.91E-12	8.58E-11
b2246	yfaV	putative transporter YfaV	1.209452559	4.114540309	0.024370433	0.064274468
b2293	hxpA	hexitol phosphatase A	1.206834843	7.312188558	2.18E-13	5.97E-12
b2465	tktB	transketolase 2	1.192680476	9.173808548	2.78E-12	6.28E-11
b4209	ytfE	iron-sulfur cluster repair protein YtfE	1.189328592	6.594665369	5.99E-09	7.99E-08
b1698	ydiR	putative electron transfer flavoprotein subunit YdiR	1.189236155	3.782798837	0.015997949	0.044759789
b2004	cbeA	CP4-44 prophage; cytoskeleton bundling-enhancing antitoxin CbeA	1.18808459	4.019402909	0.006261672	0.020312478
b2111	yehD	putative fimbrial protein YehD	1.186975813	5.574374618	4.46E-06	3.39E-05
b1679	sufE	sulfur acceptor for SufS cysteine desulfurase	1.186525518	5.628896629	8.13E-06	5.86E-05
b1970	yedX	hydroxyisourate hydrolase transthyretin-related protein	1.184505986	4.471385297	0.001661449	0.006406375
b3485	yhhJ	ABC transporter family protein YhhJ	1.182221405	7.006154158	3.35E-14	1.04E-12
b1297	puuA	glutamate-putrescine ligase	1.178183954	5.706066064	7.64E-05	0.000448192
b1680	sufS	L-cysteine desulfurase	1.17312826	7.426021232	2.25E-12	5.19E-11
b1669	ydhT	uncharacterized protein YdhT	1.172973164	5.204445842	0.000881352	0.003674382
b1927	amyA	ALPHA-AMYL-CYTO-MONOMER	1.167084471	8.676737127	5.92E-11	1.07E-09
b1127	pepT	peptidase T	1.165926071	8.456373065	5.09E-14	1.52E-12
b4013	metA	homoserine O-succinyltransferase	1.157603491	6.441351358	5.54E-09	7.41E-08
b1800	dmlA	D-malate3-isopropylmalate dehydrogenase (decarboxylating)	1.157102633	4.817864369	0.001936297	0.007349568
b2838	lysA	diaminopimelate decarboxylase	1.155949696	8.353490683	7.76E-13	1.96E-11
b0715	abrB	putative regulator	1.152003014	7.304542305	6.69E-16	2.64E-14
b2924	mscS	small conductance mechanosensitive channel MscS	1.151730838	8.56068176	6.45E-11	1.15E-09
b1867	yecD	putative hydrolase	1.151396943	7.408084726	4.52E-13	1.18E-11
b4153	frdB	fumarate reductase iron-sulfur protein	1.151254111	7.410669776	8.39E-08	8.97E-07
b2250	yfaZ	putative porin YfaZ	1.148725288	6.623889155	1.43E-08	1.77E-07
b1799	dmlR	DNA-binding transcriptional regulator DmlR	1.147170947	6.148274138	4.20E-07	3.92E-06
b3486	rbbA	ribosome-associated ATPase	1.145694609	8.193086369	8.52E-14	2.44E-12
b2543	yphA	putative inner membrane protein	1.144321633	6.534077447	6.77E-09	8.98E-08
b0804	ybiX	PKHD-type hydroxylase YbiX	1.144225649	4.749604548	0.00134639	0.005293241
b1462	yddH	flavin reductase-like protein YddH	1.144009523	5.120571524	0.000290069	0.001397978
b2464	talA	transaldolase A	1.140777124	8.379019438	1.95E-12	4.56E-11
b0773	ybhB	putative kinase inhibitor	1.140683583	7.454491667	3.81E-14	1.17E-12

b1472	yddL	putative uncharacterized protein YddL	1.139174099	1.974250198	0.186026843	0.321582424
b1463	nhoA	arylamine N-acetyltransferase	1.137615996	6.215199623	7.97E-07	7.10E-06
b4337	mdtM	multidrug efflux pump bile salt:H+	1.136814913	5.117045258	0.000104318	0.000585215
b3713	yieF	chromate reductase	1.135654791	7.947477029	1.49E-15	5.71E-14
b1881	cheZ	chemotaxis protein CheZ	1.13332383	4.40995221	0.003061606	0.010962685
b2376	ypdI	colanic acid synthesis putative lipoprotein YpdI	1.131274071	4.191602429	0.001407689	0.005514593
b0801	hcxB	hydroxycarboxylate dehydrogenase B	1.128993672	8.048500603	4.03E-12	8.80E-11
b3548	yhjY	protein YhjY	1.128442512	7.256653201	4.04E-15	1.43E-13
b1845	ptrB	oligopeptidase B	1.126134501	7.604043556	5.39E-13	1.40E-11
b0004	thrC	THRESYN-MONOMER	1.12568287	7.401228655	7.20E-14	2.13E-12
b1399	paaX	DNA-binding transcriptional repressor PaaX	1.124693205	6.394233155	3.53E-09	4.81E-08
b4157	yjeN	protein YjeN	1.123867843	5.49640078	0.000126521	0.000687922
b1448	mnaT	L-amino acid N-acyltransferase	1.123689653	6.970433277	9.20E-12	1.93E-10
b3655	yicH	AsmA famly protein YicH	1.1221354	7.783062807	1.90E-15	7.13E-14
b1740	nadE	NAD synthetase, NHsub3sub-dependent	1.11235687	7.880118279	2.40E-13	6.55E-12
b4219	msrA	methionine sulfoxide reductase A	1.1098276	6.34127124	1.92E-06	1.59E-05
b0559	ybcW	DLP12 prophage; uncharacterized protein YbcW	1.108828643	4.465161277	0.002386763	0.008855091
b2729	hypD	Fe-(CN)sub2subCO cofactor assembly scaffold protein HypD	1.107132407	7.458561959	1.57E-09	2.24E-08
b0484	copA	soluble Cu+ chaperone	1.106580651	9.077063129	3.13E-11	5.91E-10
b0907	serC	phosphoserinephosphohydroxythreonine aminotransferase	1.103612303	9.015950188	5.96E-12	1.28E-10
b0064	araC	DNA-binding transcriptional dual regulator AraC	1.098959955	5.461751513	7.66E-05	0.000448392
b4190	yjfP	carboxylesterase	1.09775854	6.537226375	1.28E-08	1.61E-07
b4380	yjjI	DUF3029 domain-containing protein YjjI	1.096647546	6.827364952	1.16E-06	9.94E-06
b2000	flu	CP4-44 prophage; self recognizing antigen 43 (Ag43) autotransporter	1.092939591	10.2477167	1.67E-10	2.77E-09
b2137	yohF	putative oxidoreductase with NAD(P)-binding Rossmann-fold domain	1.090938515	6.142605714	1.04E-05	7.28E-05
b1780	yeaD	putative aldose 1-epimerase YeaD	1.088336217	7.125868847	5.92E-10	9.04E-09
b3745	viaA	protein ViaA	1.088330361	8.381685242	6.52E-12	1.38E-10
b2335	yfcR	putative fimbrial protein YfcR	1.087330608	3.578379375	0.062537184	0.138119893
b3491	yhiM	inner membrane protein with a role in acid resistance	1.087146733	8.342478994	1.17E-10	2.00E-09
b0795	ybhG	HlyD_D23 family protein YbhG	1.086380247	7.418074122	5.34E-10	8.21E-09
b3487	yhiI	putative membrane fusion protein YhiI	1.085175512	7.37056248	8.42E-15	2.88E-13
b1224	narG	nitrate reductase A subunit α	1.083813997	11.02512781	7.98E-13	1.99E-11
b2371	yfdE	acetyl-CoA:oxalate CoA-transferase	1.083423626	3.149848739	0.039086123	0.095287263
b0485	glsA	glutaminase 1	1.083395395	8.83343604	1.05E-10	1.82E-09
b2127	mlrA	DNA-binding transcriptional activator MlrA	1.082758766	6.616764072	1.13E-08	1.44E-07
b2109	yehB	putative fimbrial usher protein YehB	1.081178059	3.806359164	0.009831514	0.029894031
b1317	ycjU	β-phosphoglucomutase	1.079362297	2.413909002	0.198924103	0.33778843
b1338	abgA	p-aminobenzoyl-glutamate hydrolase subunit A	1.078792064	3.385947336	0.039548761	0.096008239
b2068	alkA	DNA-3-methyladenine glycosylase 2	1.078155764	5.842870265	3.59E-06	2.78E-05
b3432	glgB	GLYCOGEN-BRANCH-MONOMER	1.076629506	9.409540665	2.71E-10	4.35E-09
b1692	ydiB	shikimate dehydrogenase quinate dehydrogenase	1.073699564	4.054540781	0.008283186	0.025768359
b2418	pdxK	pyridoxal kinase I	1.071171916	7.324669745	1.86E-12	4.42E-11
b0496	ybbP	putative ABC transporter membrane subunit YbbP	1.069372235	6.498044684	1.36E-08	1.70E-07
b0794	ybhF	putative ABC exporter ATP binding subunit	1.064656956	7.66058359	1.96E-11	3.78E-10
b2660	lhgO	L-2-hydroxyglutarate oxidase	1.063611474	7.020690785	9.91E-09	1.29E-07
b0003	thrB	homoserine kinase	1.060930733	7.176831886	1.70E-09	2.42E-08
b1541	ydfZ	putative selenoprotein YdfZ	1.060659183	6.80833509	1.15E-09	1.69E-08

b1603	pntA	pyridine nucleotide transhydrogenase subunit α	1.060380708	8.499436646	5.62E-11	1.02E-09
b1440	ydcS	putative ABC transporter periplasmic binding protein polyhydroxybutyra	1.059325563	5.3255113	0.000187703	0.000959155
b0712	pxpC	5-oxoprolinase component C	1.058084081	8.204009859	1.06E-10	1.83E-09
b1868	yecE	DUF72 domain-containing protein YecE	1.05808312	6.976986891	2.25E-10	3.68E-09
b0325	yahK	aldehyde reductase, NADPH-dependent	1.058029538	8.174309824	1.05E-11	2.15E-10
b0581	ybdK	carboxylate-amine ligase	1.056639406	6.792928663	8.99E-09	1.18E-07
b2902	ygfF	putative NAD(P)-binding oxidoreductase with NAD(P)-binding Rossmann	1.047568166	6.50318325	4.16E-08	4.70E-07
b3746	ravA	regulatory ATPase RavA	1.047446669	8.056205203	9.21E-08	9.68E-07
b3431	glgX	EG10381-MONOMER	1.044838095	8.988544476	2.50E-09	3.48E-08
b4335	yjiM	putative dehydratase subunit	1.040185575	5.887288133	0.000169484	0.000885824
b0033	carB	carbamoyl phosphate synthetase subunit β	1.038578521	10.44899447	1.57E-08	1.91E-07
b1484	ddpD	putative D,D-dipeptide ABC transporter ATP-binding subunit DdpD	1.037960613	4.036122588	0.006463208	0.020889504
b1600	mdtJ	multidrugspermidine efflux pump membrane subunit MdtJ	1.037584185	5.505831147	0.000567152	0.002481642
b0707	ybgA	DUF1722 domain-containing protein YbgA	1.037114232	6.083635708	2.08E-06	1.71E-05
b0515	allE	(S)-ureidoglycine aminohydrolase	1.03639297	4.929467436	0.000792283	0.003334535
b0904	focA	formate channel FocA	1.03638558	8.762758719	2.48E-10	4.03E-09
b1646	sodC	superoxide dismutase (Cu-Zn)	1.033069778	6.336682887	2.65E-06	2.13E-05
b4330	yjiH	Gate family protein YjiH	1.029629416	4.545306261	0.004170807	0.01425241
b0710	ybgI	radiation resistance protein YbgI	1.029133135	8.097444481	1.92E-12	4.53E-11
b1339	abgR	putative LysR-type DNA-binding transcriptional regulator AbgR	1.028433164	4.42840375	0.001978082	0.007477081
b2226	yfaQ	tandem DUF2300 domain-containing protein YfaQ	1.022926541	4.892092709	0.000542725	0.002391346
b1151	beeE	e14 prophage; putative protein BeeE	1.022849985	2.493337908	0.131643447	0.248060529
b2266	elaB	tail anchored inner membrane protein	1.022064557	7.359627946	1.35E-13	3.80E-12
b4020	yjbB	putative inorganic phosphate export protein	1.021170289	7.657698518	7.11E-13	1.80E-11
b2382	ypdC	putative AraC-type DNA-binding transcriptional regulator YpdC	1.020215611	5.965743876	5.80E-05	0.000350302
b0974	hyaC	hydrogenase 1 cytochrome b subunit	1.020203212	7.395670887	1.30E-11	2.61E-10
b4566	topAI	KpLE2 phage-like element; toxin of the TopAI-YjhQ toxin-antitoxin syster	1.019095754	5.726833938	0.000131825	0.000714121
b3008	metC	cystathionine β-lyase L-cysteine desulfhydrase	1.018358395	7.429799627	3.99E-14	1.21E-12
b2373	oxc	oxalyl-CoA decarboxylase	1.015017162	3.177825322	0.043969408	0.104536854
b1567	ydfW	Qin prophage; uncharacterized protein YdfW	1.014508699	3.076632029	0.042244412	0.100979469
b2204	napH	ferredoxin-type protein NapH	1.012101039	4.783135748	0.002104238	0.007920043
b0507	gcl	glyoxylate carboligase	1.010373231	4.336443634	0.0085126	0.026411193
b1505	ydeT	fimbrial usher domain-containing protein YdeT	1.010231697	2.198338297	0.21692795	0.358971851
b0711	pxpB	5-oxoprolinase component B	1.0088818	7.851868443	3.84E-12	8.47E-11
b1441	ydcT	putative ABC transporter ATP-binding protein YdcT	1.008059627	4.234559789	0.008875207	0.027344062
b3429	glgA	glycogen synthase	1.003627406	8.865912774	1.32E-10	2.25E-09
b1392	paaE	phenylacetyl-CoA 1,2-epoxidase, reductase subunit	1.003349541	3.071391103	0.051299555	0.118085264
b1602	pntB	pyridine nucleotide transhydrogenase subunit β	1.002729349	8.413308721	1.34E-10	2.27E-09
b1469	narU	nitratenitrite transporter NarU	1.000922569	6.050331439	9.42E-06	6.69E-05
b2966	yqgA	DUF554 domain-containing protein YqgA	0.998544475	6.500632044	3.55E-06	2.76E-05
b1316	ycjT	kojibiose phosphorylase	0.997619909	5.105500118	0.012978686	0.037847357
b2306	hisP	lysinearginine	0.997612378	5.670885973	3.95E-05	0.0002454
b2019	hisG	ATP phosphoribosyltransferase	0.996830684	6.926224414	5.76E-07	5.28E-06
b4149	blc	outer membrane lipoprotein Blc	0.995408258	6.8272875	2.78E-09	3.83E-08
b2222	atoA	acetyl-CoA:acetoacetyl-CoA transferase subunit β	0.992416877	2.617079948	0.103507824	0.205839924
b2025	hisF	imidazole glycerol phosphate synthase subunit HisF	0.991085593	6.715666226	1.82E-05	0.000120738
b0977	hyaF	protein HyaF	0.99103571	7.332613753	6.98E-10	1.06E-08

b4024	lysC	aspartate kinase III	0.989555341	8.815898539	1.48E-09	2.14E-08
b1576	ydfD	Qin prophage; lysis protein	0.989176379	3.051845184	0.059650603	0.132759001
b3327	gspF	Type II secretion system protein GspF	0.98484925	5.974902145	0.000290244	0.001397978
b0007	yaaJ	putative transporter YaaJ	0.983938822	6.651587581	7.14E-07	6.42E-06
b2198	ccmD	heme trafficking system membrane protein CcmD	0.983669137	4.089026257	0.014118583	0.04044826
b2097	fbaB	fructose-bisphosphate aldolase class I	0.98271351	8.447775772	1.93E-09	2.74E-08
b0394	mak	EG11288-MONOMER	0.982681718	7.494255396	1.32E-11	2.64E-10
b0936	ssuA	aliphatic sulfonate ABC transporter periplasmic binding protein	0.982027135	3.982705108	0.012094562	0.035717386
b2177	yejA	putative oligopeptide ABC transporter periplasmic binding protein	0.9816093	7.333481865	6.58E-12	1.39E-10
b1003	yccJ	PF13993 family protein YccJ	0.981526907	7.178041575	1.74E-11	3.41E-10
b1985	yeeO	FMNFAD exporter	0.981352314	6.456278467	1.82E-06	1.51E-05
b1672	ydhW	uncharacterized protein YdhW	0.980811858	4.580567999	0.003716967	0.012931766
b0790	ybhP	endonucleaseexonuclease	0.976936516	6.359086447	3.47E-05	0.000217741
b0161	degP	periplasmic serine endoprotease DegP	0.974460902	9.187400512	1.01E-08	1.31E-07
b0034	caiF	DNA-binding transcriptional activator CaiF	0.973845094	6.196293166	1.08E-05	7.49E-05
b0074	leuA	2-isopropylmalate synthase	0.972492233	5.835646657	7.23E-05	0.000425899
b3159	yhbV	putative peptidase YhbV	0.968846385	6.886761236	7.77E-05	0.00045329
b3207	yrbL	protein kinase-like domain-containing protein YrbL	0.968564727	8.000691025	2.81E-07	2.73E-06
b0031	dapB	4-hydroxy-tetrahydrodipicolinate reductase	0.966024186	8.447347187	1.01E-08	1.31E-07
b2977	glcG	putative heme-binding protein GlcG	0.964706478	6.219443228	1.85E-05	0.000122737
b0889	lrp	DNA-binding transcriptional dual regulator Lrp	0.963885856	7.714930471	2.48E-12	5.68E-11
b3430	glgC	glucose-1-phosphate adenylyltransferase	0.963213836	8.809695084	2.24E-08	2.66E-07
b0304	rclA	putative pyridine nucleotide-disulfide oxidoreductase RclA	0.962022666	6.056561525	6.07E-05	0.000365327
b1673	ydhV	putative oxidoreductase YdhV	0.961593174	4.952639298	0.006014842	0.019583723
b0837	yliI	aldose sugar dehydrogenase YliI	0.959045511	5.840774602	0.000105554	0.000590645
b1699	ydiS	putative electron transfer flavoprotein-quinone oxidoreductase YdiS	0.955652854	4.483121255	0.021633089	0.05784984
b4126	yjdI	PF06902 family protein YjdI	0.953971713	6.462853665	1.62E-07	1.62E-06
b4073	nrfD	putative menaquinol-cytochrome c reductase subunit NrfD	0.953315744	4.924977026	0.004815098	0.01614173
b4127	yjdJ	putative N-acetyltransferase YjdJ	0.952862414	6.956739508	4.87E-10	7.52E-09
b4128	ghoS	antitoxin of the GhoTS toxin-antitoxin system	0.952687856	6.34309154	8.41E-06	6.06E-05
b2503	pdeF	cyclic di-GMP phosphodiesterase PdeF	0.95247057	6.198393982	0.001230362	0.004889479
b1686	menI	1,4-dihydroxy-2-naphthoyl-CoA hydrolase	0.952354641	6.179037237	2.58E-05	0.000165391
b4196	ulaD	3-keto-L-gulonate-6-phosphate decarboxylase UlaD	0.950856041	4.844372497	0.002463926	0.009065194
b1586	ynfD	DUF1161 domain-containing protein YnfD	0.950115924	4.180888416	0.025239256	0.066249296
b4061	pdeC	c-di-GMP phosphodiesterase PdeC	0.949424433	6.85364977	1.59E-07	1.59E-06
b2110	yehC	putative fimbrial chaperone YehC	0.947913705	2.526998644	0.131525406	0.247943924
b0709	dtpD	dipeptide:H+ symporter DtpD	0.946916242	5.955696276	4.45E-05	0.000273963
b2726	hypA	hydrogenase 3 nickel incorporation protein HypA	0.945245646	5.768042389	0.000189912	0.000968203
b2449	yffR	CPZ-55 prophage; uncharacterized protein YffR	0.943802862	5.548603134	0.000327258	0.001537068
b2458	eutD	phosphate acetyltransferase EutD	0.943774284	3.92215945	0.04876742	0.11314144
b0998	torD	trimethylamine-N-oxide reductase-specific chaperone	0.943501617	6.350113754	4.60E-06	3.48E-05
b1671	ydhX	putative 4Fe-4S ferredoxin-like protein YdhX	0.940180692	4.549363648	0.003276781	0.011601434
b0897	ycaC	putative hydrolase	0.939963572	7.498179598	1.21E-11	2.46E-10
b0803	ybiI	zinc finger domain-containing protein YbiI	0.939187636	5.106762123	0.000839631	0.00351705
b1757	ynjE	molybdopterin synthase sulfurtransferase	0.938127803	7.633996164	2.21E-09	3.11E-08
b4132	cadB	lysine:cadaverine antiporter	0.934983979	6.155128585	0.001391141	0.005454608
b1391	paaD	phenylacetate degradation protein	0.934832097	2.711843729	0.138453803	0.257595254

b2024	hisA	PRIBFAICARPISOM-MONOMER	0.9309404	6.561350801	3.48E-06	2.71E-05
b1887	cheW	chemotaxis protein CheW	0.929802639	4.407352813	0.012058425	0.035634503
b0777	bioC	malonyl-acyl carrier protein methyltransferase	0.929031728	4.192581862	0.01315831	0.038320541
b3098	yqjD	ribosome- and membrane-associated DUF883 domain-containing prote	0.927864798	7.83690106	2.48E-09	3.47E-08
b0219	yafV	2-oxoglutaramate amidase	0.924658792	7.280098534	1.04E-08	1.34E-07
b1068	yceM	putative oxidoreductase YceM	0.922280289	7.175577014	1.54E-08	1.89E-07
b0618	citC	CITC-MONOMER	0.922029485	4.871240287	0.006523805	0.021039153
b4225	chpB	endoribonuclease toxin ChpB	0.921799719	5.879684488	0.000198959	0.001007982
b1259	yciG	stress-induced bacterial acidophilic repeat motifs-containing protein Yci	0.917934343	4.165124264	0.018062435	0.049654825
b1900	araG	arabinose ABC transporter ATP binding subunit	0.912149984	5.05997818	0.002540017	0.009306369
b1227	narI	nitrate reductase A subunit γ	0.911328368	7.918740244	1.83E-10	3.03E-09
b0750	nadA	quinolinate synthase	0.910856248	6.508163125	2.91E-05	0.000185564
b4474	frlC	fructoselysine 3-epimerase	0.909702581	5.276434693	0.001232073	0.004891729
b0824	ybiY	putative pyruvate formate-lyase activating enzyme YbiY	0.909461413	3.479827399	0.057145949	0.128266072
b2787	gudD	D-glucarate dehydratase	0.908630406	6.679224545	3.74E-07	3.53E-06
b4683	yqeL	uncharacterized protein YqeL	0.906371085	5.104893278	0.013817519	0.039794095
b1792	yeaO	DUF488 domain-containing protein YeaO	0.905882569	6.326007101	5.60E-06	4.17E-05
b0047	kefC	K+ : H+	0.905269176	7.401712803	8.66E-08	9.19E-07
b0644	ybeQ	Sel1 repeat-containing protein YbeQ	0.905245346	6.099267198	3.12E-05	0.000197402
b3564	xylB	xylulokinase	0.904952311	7.256637977	1.05E-09	1.55E-08
b1739	osmE	osmotically-inducible lipoprotein OsmE	0.904754242	7.434009811	2.66E-10	4.28E-09
b0751	pnuC	PNUC-MONOMER	0.90446347	6.744972514	3.42E-05	0.0002154
b0823	ybiW	putative pyruvate formate lyase	0.903863273	5.740874714	0.000499537	0.002216538
b1573	ydfC	Qin prophage; uncharacterized protein YdfC	0.902798977	4.298271701	0.014612897	0.04151605
b1358	ydaT	Rac prophage; protein YdaT	0.902773951	3.36580819	0.070335183	0.151869043
b1502	ydeQ	putative fimbrial adhesin protein YdeQ	0.901727296	2.072159282	0.244414602	0.392968124
b3374	frlD	fructoselysine 6-kinase	0.8980311	5.667517093	0.000354983	0.001651475
b2366	dsdA	D-serine ammonia-lyase	0.895095412	6.169064739	0.000219229	0.001087525
b1963	yedR	putative inner membrane protein	0.894604145	6.422820364	2.97E-05	0.000188758
b0494	tesA	multifunctional acyl-CoA thioesterase I and protease I and lysophosphol	0.894295428	7.192937402	7.42E-07	6.63E-06
b0806	mcbA	DUF1471 domain-containing protein McbA	0.892790978	4.621421184	0.027582126	0.071380472
b2730	hypE	HypE-S-carboxamide	0.892299844	7.144775383	1.20E-06	1.03E-05
b4019	metH	cobalamin-dependent methionine synthase	0.892123123	9.0117669	8.43E-08	8.99E-07
b0616	citE	citrate lyase β subunit	0.891188318	2.961174027	0.166416147	0.296500117
b2385	ypdF	aminopeptidase	0.890475176	3.49004168	0.089907779	0.184882554
b1359	ydaU	Rac prophage; DUF1376 domain-containing protein YdaU	0.889178358	4.268121751	0.020345114	0.05490445
b1732	katE	catalase II	0.886801038	7.920638523	4.39E-07	4.08E-06
b1897	otsB	TREHALOSEPHOSPHASYN-MONOMER	0.884135839	7.388588891	3.19E-08	3.66E-07
b0754	aroG	3-deoxy-7-phosphoheptulonate synthase, Phe-sensitive	0.883304543	8.647688292	4.94E-08	5.47E-07
b0073	leuB	3-isopropylmalate dehydrogenase	0.88259303	5.375793897	0.004142175	0.014176514
b2381	ypdB	phosphorylated DNA-binding transcriptional activator YpdB	0.881963486	7.156012989	1.06E-08	1.36E-07
b4065	yjcE	putative transporter YjcE	0.880946689	8.079567496	7.09E-10	1.07E-08
b1647	ydhF	putative oxidoreductase	0.880104834	7.570384185	3.76E-08	4.29E-07
b2849	yqeK	protein YqeK	0.877802942	5.365890988	0.020574771	0.055490295
b1301	puuB	γ-glutamylputrescine oxidase	0.877801633	3.166346875	0.131358006	0.247734129
b1772	ydjH	putative sugar kinase YdjH	0.877237211	3.791733154	0.067172061	0.146090961
b4435	isrC	small RNA IsrC	0.875104703	5.470745886	0.002412078	0.008926509

b1182	hlyE	hemolysin E	0.874743592	4.504629484	0.014474217	0.0413329
b0622	pagP	Lipid IVsubAsub palmitoyltransferase	0.873587337	7.521783265	8.97E-08	9.47E-07
b3172	argG	argininosuccinate synthetase	0.873554923	8.185593065	3.53E-09	4.81E-08
b4152	frdC	fumarate reductase membrane protein FrdC	0.869217953	6.376236883	0.000682335	0.002924193
b3099	yqjE	inner membrane protein YqjE	0.866629689	7.988356267	2.70E-08	3.15E-07
b0844	ybjI	5-amino-6-(5-phospho-D-ribitylamino)uracil phosphatase	0.864107625	5.945387845	0.000651339	0.002816514
b0475	hemH	PROTOHEME-FERROCHELAT-MONOMER	0.863808413	6.909186567	5.72E-05	0.000346609
b3102	yqjG	glutathionyl-hydroquinone reductase YqjG	0.863343555	7.461512168	1.48E-08	1.84E-07
b1333	uspE	universal stress protein with a role cellular motility	0.863070161	8.852005954	3.79E-08	4.31E-07
b2372	yfdV	putative transport protein YfdV	0.861024707	2.560713917	0.210054558	0.351284423
b1753	ynjA	AhpD-like domain-containing protein YnjA	0.859724403	5.321906394	0.001504336	0.005851669
b4131	cadA	lysine decarboxylase 1	0.858634451	7.665297269	2.64E-08	3.10E-07
b3519	treF	cytoplasmic trehalase	0.857341043	8.236075435	6.37E-08	6.92E-07
b0480	ushA	5'-nucleotidase UDP-sugar hydrolase	0.856337394	8.303259617	4.34E-07	4.04E-06
b3023	ygiV	DNA-binding transcriptional repressor YgiV	0.855443888	7.380378795	1.32E-09	1.92E-08
b1843	yobB	putative carbon-nitrogen hydrolase family protein YobB	0.854439573	5.785335343	0.000567934	0.002482602
b0487	cueR	DNA-binding transcriptional dual regulator CueR	0.85412711	7.334413652	1.34E-09	1.94E-08
b2002	yeeS	CP4-44 prophage; RadC-like JAB domain-containing protein YeeS	0.853237148	4.651412372	0.012735357	0.037260836
b4551	yheV	DUF2387 domain-containing protein YheV	0.852831769	6.447204845	6.40E-05	0.000381682
b2023	hisH	imidazole glycerol phosphate synthase subunit HisH	0.852349759	6.337687639	0.000105909	0.000591887
b1050	yceK	DUF1375 domain-containing lipoprotein YceK	0.851695913	6.999239911	9.45E-08	9.89E-07
b3612	gpmM	2,3-bisphosphoglycerate-independent phosphoglycerate mutase	0.8512999	9.65972619	2.38E-07	2.34E-06
b0778	bioD	dethiobiotin synthetase	0.850952694	4.207246434	0.026731865	0.069587962
b1572	ydfB	Qin prophage; uncharacterized protein YdfB	0.849436659	4.276508646	0.018645799	0.051004462
b0479	fsr	FSR-MONOMER	0.849330328	5.732740558	0.00118752	0.004736136
b3060	ttdR	Dan transcriptional activator	0.848821445	6.253509649	0.002917086	0.01050484
b2384	ypdE	broad-specificity exoaminopeptidase	0.847018423	3.918282328	0.045768554	0.107719434
b0037	caiC	carnitine—CoA ligase	0.846704105	5.93537685	0.000865949	0.00362042
b0770	ybhI	putative tricarboxylate transporter	0.84630895	2.410478932	0.279364253	0.431558145
b0706	ybfD	H repeat-associated putative transposase YbfD	0.845774019	4.171191016	0.043412595	0.103380047
b2224	atoB	acetyl-CoA acetyltransferase	0.844587305	4.35177554	0.017494061	0.048333091
b0756	galM	galactose-1-epimerase	0.844342819	8.414609939	2.39E-08	2.81E-07
b1482	osmC	osmotically inducible peroxiredoxin OsmC	0.841669222	6.897481805	2.10E-06	1.72E-05
b0419	yajO	1-deoxyxylulose-5-phosphate synthase YajO	0.839352597	8.007915306	7.45E-10	1.12E-08
b1218	chaC	glutathione-specific γ-glutamylcyclotransferase	0.838943666	7.282613506	1.78E-07	1.78E-06
b0906	ycaP	DUF421 domain-containing protein YcaP	0.837434691	6.500389592	4.96E-05	0.000302883
b0160	dgt	dGTP triphosphohydrolase	0.836577226	7.125343442	3.33E-07	3.18E-06
b2200	ccmB	heme trafficking system membrane protein CcmB	0.834905184	5.324219893	0.002915635	0.01050484
b4398	creB	Phosphorylated DNA-binding transcriptional regulator CreB	0.834888665	6.805708943	1.30E-05	8.87E-05
b2837	galR	DNA-binding transcriptional dual regulator GalR	0.832594418	7.48612137	2.94E-10	4.69E-09
b1885	tap	methyl-accepting chemotaxis protein Tap	0.83256217	3.866788732	0.071392533	0.153231906
b4253	yjgL	protein YjgL	0.831838023	8.825850397	1.58E-07	1.59E-06
b2309	hisJ	histidine ABC transporter periplasmic binding protein	0.830384079	6.528040791	1.50E-05	0.000101437
b2202	napC	periplasmic nitrate reductase cytochrome c protein	0.82953252	6.138637596	9.91E-05	0.000564558
b1066	rimJ	EG10851-MONOMER	0.828140511	7.3209896	5.11E-07	4.70E-06
b4518	ymdF	stress-induced bacterial acidophilic repeat motifs-containing protein Yn	0.828010586	6.425092412	0.000315137	0.001488055
b3160	yhbW	putative luciferase-like monooxygenase	0.827873507	7.509778722	2.72E-06	2.17E-05

b4602	ynhF	stress response membrane protein YnhF	0.826228716	5.610849536	0.001864743	0.007091164
b2386	fryC	putative PTS enzyme IIC component FryC	0.826199345	3.228562906	0.154622991	0.28093025
b2446	yffO	CPZ-55 prophage; uncharacterized protein YffO	0.826025758	4.203122286	0.039306825	0.09556147
b3589	yiaY	L-threonine dehydrogenase	0.818807502	5.578071043	0.002781454	0.010090485
b4263	yjgR	DUF853 domain-containing protein YjgR	0.818499299	7.842766151	1.38E-07	1.42E-06
b4444	omrA	small regulatory RNA OmrA	0.815557261	5.908934475	0.005944424	0.01941171
b2715	ascF	β-glucoside specific PTS enzyme IIBC component	0.8150438	5.55050457	0.003673806	0.012791683
b2020	hisD	histidinalhistidinol dehydrogenase	0.814662971	7.004212034	1.18E-05	8.14E-05
b1341	dgcM	diguanylate cyclase DgcM	0.814324835	7.212627663	1.58E-07	1.59E-06
b3370	frlA	fructoselysinepsicoselysine transporter	0.814138541	4.970062754	0.0063446	0.020551292
b1051	msyB	acidic protein that suppresses heat sensitivity of a secY mutant	0.813442206	7.823418162	8.10E-08	8.70E-07
b0166	dapD	tetrahydrodipicolinate succinylase	0.809784218	8.769846886	6.63E-07	6.00E-06
b4077	gltP	glutamateaspartate : H+	0.809776275	7.548274134	2.19E-08	2.61E-07
b1521	uxaB	ALTRO-OXIDOREDUCT-MONOMER	0.809562994	4.66185735	0.039380453	0.095687781
b2665	kbp	K+ binding protein	0.809393127	7.696564091	1.11E-08	1.42E-07
b0976	hyaE	putative HyaA chaperone	0.807341206	6.690058814	6.50E-06	4.78E-05
b1261	trpB	tryptophan synthase subunit β	0.807025472	6.529328517	0.000167697	0.000878272
b0453	ybaY	PF09619 family lipoprotein YbaY	0.806780678	7.707437833	8.93E-10	1.33E-08
b4235	pmbA	metalloprotease PmbA	0.804896245	8.76418251	1.71E-06	1.43E-05
b2201	ccmA	heme trafficking system ATP-binding protein	0.804662636	6.463051305	0.000101425	0.000571163
b2788	gudX	glucarate dehydratase-related protein	0.804616664	5.263875405	0.00655669	0.021129771
b1195	ymgE	PF04226 family protein YmgE	0.80450401	4.120445052	0.03224167	0.081574197
b0036	caiD	CARNRACE-MONOMER	0.803588462	3.127618823	0.136614294	0.255140486
b2370	evgS	sensory histidine kinase EvgS - his721 phosphorylated	0.802950459	7.947331979	2.75E-08	3.20E-07
b1002	agp	glucose-1-phosphatase	0.800604131	7.040310861	2.79E-07	2.70E-06
b0975	hyaD	putative hydrogenase 1 maturation protease HyaD	0.800063992	7.271503002	1.14E-07	1.18E-06
b1249	clsA	cardiolipin synthase A	0.799879372	7.828464588	6.07E-09	8.07E-08
b3368	cysG	siroheme synthase	0.799026965	8.056328316	1.21E-06	1.03E-05
b0575	cusA	coppersilver export system RND permease	0.798826529	5.547920055	0.001691498	0.006505196
b0767	pgl	6PGLUCONOLACT-MONOMER	0.798410193	8.489001938	2.71E-06	2.17E-05
b2672	ygaM	DUF883 domain-containing protein YgaM	0.797907585	7.458302581	2.85E-08	3.29E-07
b0123	cueO	multicopper oxidase CueO	0.796096148	8.101278099	4.50E-07	4.17E-06
b4055	aphA	acid phosphatase phosphotransferase	0.795683854	6.256983814	0.002814945	0.01018687
b1886	tar	methyl-accepting chemotaxis protein Tar	0.795342839	4.330543081	0.04577161	0.107719434
b0587	fepE	polysaccharide co-polymerase family protein FepE	0.795071228	3.119399094	0.124856352	0.237501418
b2308	hisQ	lysinearginine	0.794329554	5.362647044	0.006932018	0.022161377
b0032	carA	carbamoyl phosphate synthetase subunit α	0.793623944	8.980473238	0.00021074	0.001054892
b0937	ssuE	NADPH-dependent FMN reductase	0.792090466	4.650862892	0.016573836	0.046103082
b3829	metE	HOMOCYSMET-MONOMER	0.791491232	9.737864225	3.18E-06	2.51E-05
b1303	pspF	DNA-binding transcriptional dual regulator PspF	0.791431179	5.99286234	0.000732407	0.003106224
b3361	fic	putative adenosine monophosphate—protein transferase Fic	0.79103785	7.484209713	2.13E-06	1.74E-05
b1067	yceH	DUF480 domain-containing protein YceH	0.787337535	7.272163665	1.73E-07	1.73E-06
b3588	aldB	aldehyde dehydrogenase B	0.778969196	5.42545973	0.007850553	0.024599142
b1360	ydaV	Rac prophage; putative ATP-binding protein YdaV	0.777897626	3.572200644	0.100419605	0.20100973
b1606	folM	dihydromonapterin reductase	0.776277334	6.511147821	2.04E-05	0.000134125
b1758	ynjF	putative phosphatidyl transferase, inner membrane protein	0.774779043	5.323679104	0.009605209	0.029306841
b0488	ybbJ	NfeD-like family protein	0.773212454	6.616390576	2.21E-05	0.000143443

b3244	tldD	metalloprotease TldD	0.772800986	9.19603752	6.78E-07	6.12E-06
b0752	zitB	Zn2+	0.770181292	6.762801437	2.12E-05	0.000138973
b4252	tabA	DUF386 domain-containing toxin-antitoxin biofilm protein TabA	0.766927168	8.25555244	2.70E-07	2.63E-06
b2377	yfdY	DUF2545 domain-containing protein YfdY	0.766304066	5.789334579	0.002267647	0.008462941
b0793	ybhS	putative ABC exporter membrane subunit YbhS	0.765144761	6.114745064	0.00060214	0.002626926
b1518	lsrG	(4S)-4-hydroxy-5-phosphonooxypentane-2,3-dione isomerase	0.763441995	3.392898616	0.214156108	0.355986151
b1256	ompW	outer membrane protein W	0.763308785	6.149979467	0.004398102	0.014936633
b4185	yjfM	DUF1190 domain-containing protein YjfM	0.758991308	2.733653486	0.288009753	0.441208556
b2247	rhmD	L-rhamnonate dehydratase	0.758988404	3.211597083	0.237969485	0.384989109
b2135	yohC	putative inner membrane protein	0.758905031	4.735516734	0.020066696	0.054319107
b1045	ymdB	2'-O-acetyl-ADP-ribose deacetylase, regulator of RNase III activity	0.757251083	6.429257848	0.000115162	0.000633968
b1754	ynjB	putative ABC transporter periplasmic binding protein YnjB	0.75701279	6.02902373	0.000423346	0.001909167
b0865	ybjP	DUF3828 domain-containing lipoprotein YbjP	0.754233711	6.819083165	7.27E-05	0.000427156
b4224	chpS	ChpS antitoxin of the ChpB-ChpS toxin-antitoxin system	0.754110465	5.420715355	0.006363858	0.020598557
b2916	argP	DNA-binding transcriptional dual regulator ArgP	0.754015128	7.122080166	1.34E-06	1.14E-05
b1697	ydiQ	putative electron transfer flavoprotein subunit YdiQ	0.753685917	4.132198713	0.090819903	0.186237749
b4565	sgcB	putative PTS enzyme IIB component SgcB	0.753036007	4.247116104	0.04230735	0.10107519
b3371	frlB	fructoselysine 6-phosphate deglycase	0.752864498	5.062981218	0.008740941	0.026998133
b1395	paaH	G6716-MONOMER	0.752677591	3.828560962	0.119569198	0.231128725
b0600	ybdL	methionine-oxo-acid transaminase, PLP-dependent	0.750876285	5.909860451	0.000659353	0.00284838
b3514	mdtF	multidrug efflux pump RND permease MdtF	0.750684936	10.15638597	3.56E-06	2.76E-05
b1802	yeaW	carnitine monooxygenase subunit YeaW	0.750682511	3.374311496	0.143150747	0.264550249
b2498	upp	uracil phosphoribosyltransferase	0.749987276	8.075868534	8.14E-07	7.23E-06
b3328	gspG	Type II secretion system protein GspG	0.749958184	5.021449761	0.041813092	0.1002742
b1913	uvrC	excision nuclease subunit C	0.749180945	9.075050737	1.47E-06	1.23E-05
b0713	pxpA	5-oxoprolinase component A	0.747213652	7.49095468	1.62E-05	0.000108468
b1589	ynfG	putative oxidoreductase YnfG	0.747098047	4.133503772	0.066505744	0.144927375
b1815	pdeD	putative c-di-GMP phosphodiesterase PdeD	0.746069514	5.881142019	0.002098289	0.007904391
b1492	gadC	XASA-MONOMER	0.745798485	9.459883523	4.46E-06	3.39E-05
b0863	artI	putative ABC transporter periplasmic binding protein ArtI	0.745167083	7.867006834	5.21E-08	5.75E-07
b0856	potH	putrescine ABC transporter membrane subunit PotH	0.74478652	4.325087956	0.039702674	0.096205984
b2989	yghU	disulfide reductase organic hydroperoxide reductase	0.743663598	7.347923881	3.67E-07	3.47E-06
b2664	csiR	DNA-binding transcriptional repressor CsiR	0.743430376	6.783854636	0.00013611	0.000733729
b1337	abgB	p-aminobenzoyl-glutamate hydrolase subunit B	0.741449511	4.708968426	0.021601067	0.0578008
b1776	ydjL	putative zinc-binding dehydrogenase YdjL	0.741149675	3.782838735	0.102677769	0.204660203
b1312	ycjP	putative ABC transporter membrane subunit YcjP	0.741018309	2.518927132	0.286383859	0.439633079
b3450	ugpC	sn-glycerol 3-phosphate ABC transporter ATP binding subunit	0.740516888	7.087990774	3.55E-05	0.000221875
b1342	zntB	Zn2+:H+	0.73859726	7.900325871	6.31E-08	6.90E-07
b1854	pykA	pyruvate kinase II	0.738544083	9.129837638	6.75E-06	4.94E-05
b3494	uspB	putative universal stress (ethanol tolerance) protein B	0.738109531	7.369931977	6.41E-08	6.95E-07
b4123	dcuB	anaerobic C4-dicarboxylate transporter DcuB	0.737719567	5.941450619	0.006166634	0.02001889
b1695	ydiO	putative acyl-CoA dehydrogenase	0.737692632	3.672363034	0.149155438	0.273358762
b0796	cecR	DNA-binding transcriptional dual regulator CecR	0.73749097	6.121804022	0.00071441	0.003044516
b3693	dgoK	DEHYDDEOXGALACTKIN-MONOMER	0.737134518	5.856855162	0.002853254	0.010297825
b2476	purC	phosphoribosylaminoimidazole-succinocarboxamide synthase	0.736440305	7.593808843	5.79E-07	5.30E-06
b0336	codB	CODB-MONOMER	0.736091724	6.79508173	1.79E-05	0.000119187
b1790	nimR	DNA-binding transcriptional repressor NimR	0.73604039	5.758430242	0.002114196	0.007950745

b2223	atoE	short chain fatty acid transporter	0.735367095	3.474755803	0.199600226	0.338545907
b1474	fdnG	formate dehydrogenase N subunit α	0.730930356	9.065157995	3.86E-05	0.000240572
b1400	paaY	2-hydroxycyclohepta-1,4,6-triene-1-carboxyl-CoA thioesterase	0.730443682	6.436562194	0.000402178	0.001834316
b4466	yghJ	putative lipoprotein YghJ	0.729824439	7.348605773	5.59E-06	4.17E-05
b0870	ltaE	low-specificity L-threonine aldolase	0.729170596	7.954805789	6.57E-07	5.96E-06
b4057	yjbR	PF04237 family protein YjbR	0.728402064	7.718552512	2.06E-08	2.47E-07
b1775	ydjK	putative transporter YdjK	0.728054018	3.438835725	0.176205631	0.309373241
b1300	puuC	γ-glutamyl-γ-aminobutyraldehyde dehydrogenase	0.727636241	3.803101691	0.125049175	0.237765766
b1931	yedK	putative SOS response-associated peptidase YedK	0.72564756	4.797148239	0.040709177	0.097999463
b4740	ymgM	protein YmgM	0.723795016	5.468985635	0.004878833	0.016305865
b3506	slp	starvation lipoprotein	0.723590566	8.665932377	5.18E-06	3.89E-05
b3597	yibH	inner membrane protein YibH	0.722934481	7.048185204	1.49E-05	0.000100803
b2334	yfcQ	putative fimbrial protein YfcQ	0.722495686	3.688201915	0.094668514	0.192077891
b4119	melA	α-galactosidase	0.722451901	5.88870794	0.001207559	0.004811709
b2336	yfcS	putative fimbrial chaperone YfcS	0.721761114	3.072245674	0.272072209	0.423703281
b0595	entB	holo EntB	0.721579441	3.867498381	0.122986538	0.235466421
b1278	pgpB	phosphatidylglycerophosphatase B	0.720356775	6.465201538	0.000158076	0.000835815
b0848	ybjM	putative inner membrane protein	0.719543426	6.963635863	4.20E-05	0.000260719
b3474	yhhT	putative transporter YhhT	0.717476428	7.479091775	4.92E-08	5.46E-07
b3025	qseB	Phosphorylated DNA-binding transcriptional activator QseB	0.715779195	6.578890039	6.59E-05	0.000392361
b3157	yhbT	SCP2 domain-containing protein YhbT	0.715000619	8.119300964	1.01E-06	8.84E-06
b1536	ydeI	BOF family protein YdeI	0.714607603	5.457506737	0.011292553	0.033641446
b1884	cheR	chemotaxis protein methyltransferase	0.713865557	3.282944007	0.268232638	0.420393005
b4324	uxuR	DNA-binding transcriptional repressor UxuR	0.713814615	6.872013481	1.19E-05	8.23E-05
b1015	putP	proline:Na+ symporter	0.713183068	6.910404589	1.58E-05	0.000106292
b0072	leuC	3-isopropylmalate dehydratase subunit LeuC	0.71222224	5.689781091	0.009228133	0.028273564
b1166	ariR	regulator of acid resistance, influenced by indole	0.711655573	5.821120578	0.003541625	0.012409742
b0788	ybhN	conserved inner membrane protein YbhN	0.710643585	5.8982555	0.004843479	0.016212252
b1201	dhaR	DNA-binding transcriptional dual regulator DhaR	0.71020193	6.812564192	0.00030946	0.001470683
b2001	yeeR	CP4-44 prophage; inner membrane protein YeeR	0.708844011	7.106239176	2.06E-05	0.000135413
b0781	moaA	GTP 3',8'-cyclase	0.708507589	8.401599098	8.05E-06	5.83E-05
b1725	yniA	putative kinase YniA	0.70847259	7.817885062	1.08E-07	1.13E-06
b2479	gcvR	putative transcriptional regulator GcvR	0.705606781	7.565923586	3.57E-07	3.39E-06
b4199	yjfY	DUF1471 domain-containing protein YjfY	0.705574222	5.127343421	0.054460343	0.123430397
b0999	cbpM	chaperone modulator CbpM	0.705448081	7.580797884	1.17E-07	1.21E-06
b1529	marC	inner membrane protein MarC	0.705251119	6.39616112	0.002209062	0.008258262
b0825	fsaA	fructose-6-phosphate aldolase 1	0.705030686	5.96044144	0.00150224	0.005848667
b3040	zupT	heavy metal divalent cation transporter ZupT	0.704986789	7.422422157	1.69E-06	1.41E-05
b3481	nikR	DNA-binding transcriptional repressor NikR	0.704822684	5.988994743	0.002213766	0.008268846
b0714	nei	endonuclease VIII	0.704705446	7.436503271	7.18E-07	6.44E-06
b1574	dicF	Qin prophage; small regulatory RNA DicF	0.703956062	3.357785225	0.137089238	0.25583059
b1226	narJ	nitrate reductase 1 molybdenum cofactor assembly chaperone	0.703792107	8.270630829	2.88E-06	2.28E-05
b2178	yejB	putative oligopeptide ABC transporter membrane subunit YejB	0.703219585	6.067715788	0.000975913	0.004004325
b0867	amiD	N-acetylmuramoyl-L-alanine amidase D	0.70301457	7.683806408	4.50E-06	3.42E-05
b4430	rydB	small regulatory RNA RydB	0.700691566	3.878952913	0.094792641	0.192241392
b3253	yhdH	acrylyl-CoA reductase	0.700426601	8.426148063	0.000100663	0.000569995
b1539	ydfG	3-hydroxy acid dehydrogenase	0.69888098	8.115136122	1.16E-06	9.94E-06

b3428	glgP	glycogen phosphorylase	0.696730601	9.603896859	9.91E-06	7.00E-05
b4220	tamA	translocation and assembly module subunit TamA	0.694938113	8.072566263	1.20E-06	1.03E-05
b3433	asd	aspartate-semialdehyde dehydrogenase	0.694677044	9.408433755	3.07E-05	0.000194891
b1962	yedJ	putative HD superfamily phosphohydrolase YedJ	0.69464948	7.403380357	6.78E-05	0.000402559
b1007	rutF	flavin reductase	0.694071576	2.995671607	0.220063443	0.363071787
b2459	eutT	putative ethanolamine utilization cobalamin adenosyltransferase	0.693928719	3.791857437	0.171987106	0.304094142
b0307	ykgF	putative amino acid dehydrogenase with NAD(P)-binding domain and fe	0.693692976	6.776443268	8.10E-05	0.000470459
b4605	ypaB	protein YpaB	0.691224651	3.102504129	0.24902929	0.398068181
b1971	msrP	periplasmic protein-L-methionine sulfoxide reductase catalytic subunit	0.690638946	6.292638115	0.001022798	0.004165732
b2659	csiD	PF08943 family protein CsiD	0.69041873	6.379775729	0.001851469	0.007052834
b0968	yccX	acylphosphatase	0.688254478	6.304669874	0.000373577	0.001727062
b4479	dgoR	DNA-binding transcriptional regulator DgoR	0.686737155	6.509463875	0.000171784	0.000894369
b0800	ybiB	nonspecific DNA-binding protein YbiB	0.683312319	7.793768706	0.000160617	0.000846864
b4054	tyrB	tyrosine aminotransferase	0.679395779	8.386826305	3.94E-06	3.02E-05
b1736	chbA	N,N'-diacetylchitobiose-specific PTS enzyme IIA component	0.679369893	5.111480103	0.01438451	0.041131874
b4738	ymgK	protein YmgK	0.676404032	2.555868245	0.282927361	0.435085241
b4755	yqhI	protein YqhI	0.675804495	4.235103326	0.122163105	0.23441868
b4350	hsdR	type I restriction enzyme EcoKI endonuclease component	0.669971962	8.529981548	0.00016954	0.000885824
b2357	yfdN	CPS-53 (KpLE1) prophage; protein YzyA	0.669203089	1.818619385	0.565002383	0.705055264
b1141	xisE	e14 prophage; putative excisionase	0.669129796	4.522657582	0.057805679	0.129286764
b1244	oppB	murein tripeptide ABC transporter oligopeptide ABC transporter inner r	0.669097295	8.786135336	0.000172982	0.000898972
b1595	ynfL	putative DNA-binding transcriptional regulator	0.668186054	5.060458575	0.024433799	0.064403117
b3472	dcrB	periplasmic bacteriophage sensitivity protein DcrB	0.667485551	8.293887957	3.34E-06	2.62E-05
b1444	patD	γ-aminobutyraldehyde dehydrogenase	0.667429868	6.562170732	0.00019124	0.000973844
b1225	narH	nitrate reductase A subunit β	0.667154598	9.608573754	2.93E-05	0.000186547
b0604	dsbG	protein sulfenic acid reductase and chaperone DsbG	0.666578857	6.642487442	0.000218316	0.001084214
b0350	mhpD	2-hydroxypentadienoate hydratase	0.666288587	7.397762593	1.07E-06	9.28E-06
b0040	caiT	L-carnitine:γ-butyrobetaine antiporter	0.666068642	3.636756593	0.222217594	0.365669279
b2454	eutJ	putative ethanolamine utilization chaperonin EutJ	0.662590516	3.347585621	0.258083466	0.408227169
b4334	yjiL	putative ATPase, activator of (R)-hydroxyglutaryl-CoA dehdratase	0.662522167	4.715457833	0.079646174	0.167366901
b2233	yfaL	putative autotransporter adhesin YfaL	0.661315223	7.86776763	0.000935875	0.003861576
b0516	allC	allantoate amidohydrolase	0.659721658	4.533203937	0.058345184	0.130229519
b0111	ampE	protein AmpE	0.657567692	7.026845172	1.02E-05	7.17E-05
b2199	ccmC	heme trafficking system membrane protein CcmC	0.657178834	5.920279478	0.010324985	0.031201101
b4467	glcF	glycolate dehydrogenase, putative iron-sulfur subunit	0.6568085	6.417546958	0.00047313	0.00211424
b0075	leuL	leu operon leader peptide	0.656779677	5.135342383	0.031924704	0.08105093
b2922	yggE	uncharacterized protein YggE	0.656220836	8.521689641	3.90E-05	0.000242534
b1000	cbpA	curved DNA-binding protein	0.654762936	9.120621871	9.01E-05	0.00051795
b0596	entA	2,3-dihydro-2,3-dihydroxybenzoate dehydrogenase	0.654610375	4.930657098	0.073381763	0.15673947
b0039	caiA	CROBETREDUCT-MONOMER	0.653608947	6.285748999	0.001087282	0.004387888
b0878	macA	ABC-type tripartite efflux pump membrane fusion protein	0.652809909	6.596120255	0.002496207	0.009168682
b0678	nagB	glucosamine-6-phosphate deaminase	0.651627866	7.538203767	2.02E-06	1.66E-05
b1500	safA	two-component system connector SafA	0.651026826	2.022256529	0.591018604	0.726522567
b0662	ubiF	OCTAPRENYL-METHYL-METHOXY-BENZOQ-OH-MON	0.650745519	7.74086853	3.70E-06	2.85E-05
b1588	ynfF	putative selenate reductase YnfF	0.64755405	6.15534828	0.003553225	0.012440513
b0351	mhpF	acetaldehyde dehydrogenase (acetylating) MhpF	0.646688913	7.189016682	3.44E-05	0.000216223
b0110	ampD	1,6-anhydro-N-acetylmuramoyl-L-alanine amidase	0.646368089	6.824509338	0.00040215	0.001834316

b3097	yqjC	DUF1090 domain-containing protein YqjC	0.646133043	8.257403097	1.19E-05	8.20E-05
b1033	ghrA	glyoxylatehydroxypyruvate reductase A	0.64578395	7.23820864	0.000114189	0.000629394
b2805	fucR	DNA-binding transcriptional activator FucR	0.645416147	6.050405127	0.008736391	0.026998133
b0808	ybiO	moderate conductance mechanosensitive channel	0.644289012	6.080105398	0.024120226	0.063728785
b1059	solA	SARCOX-MONOMER	0.642926915	7.332714703	3.99E-06	3.05E-05
b0908	aroA	AROA-MONOMER	0.640217868	8.374571822	4.76E-05	0.000292087
b4743	ynaL	protein YnaL	0.638037127	5.167793158	0.019256554	0.052421139
b4111	proP	osmolyte:H+ symporter ProP	0.637956928	9.313326715	0.000213856	0.001065659
b0038	caiB	γ-butyrobetainyl-CoA:carnitine CoA transferase	0.636590407	5.681924784	0.013623237	0.03938873
b2163	yeiL	putative DNA-binding transcriptional regulator YeiL	0.635403523	2.763796242	0.291578551	0.44466988
b0996	torC	cytochrome c menaquinol dehydrogenase TorC	0.634299215	3.985791184	0.141982424	0.262534421
b3369	yhfL	DUF4223 domain-containing lipoprotein YhfL	0.633291055	5.655368544	0.019962408	0.054069958
b2015	yeeY	putative DNA-binding transcriptional regulator YeeY	0.632928555	6.856172412	0.000199085	0.001007982
b2489	hyfI	hydrogenase 4 catalytic subunit HyfI	0.632862912	4.961929098	0.044020668	0.104602395
b0768	ybhD	putative DNA-binding transcriptional regulator YbhD	0.631896184	4.682616192	0.052766184	0.120697211
b2080	yegP	DUF1508 domain-containing protein YegP	0.627501262	6.702254047	0.00036398	0.001687995
b4392	slt	soluble lytic murein transglycosylase	0.627444697	8.452687267	9.99E-05	0.000567889
b4478	dgoD	GALACTONATE-DEHYDRATASE-MONOMER	0.627328247	5.602820866	0.014768441	0.041837052
b0197	metQ	L-methionineD-methionine ABC transporter membrane anchored bindin	0.626866695	8.279121164	1.42E-05	9.63E-05
b1393	paaF	putative 2,3-dehydroadipyl-CoA hydratase	0.62680922	3.249182729	0.245910841	0.394511761
b1724	ydiZ	protein YdiZ	0.626362326	6.519596783	0.002652847	0.009679604
b3100	yqjK	PF13997 family protein YqjK	0.624936877	7.455479246	0.000185096	0.000950229
b3330	gspI	Type II secretion system protein GspI	0.623976949	5.120562221	0.086903388	0.179877384
b2834	tas	putative NADP(H)-dependent aldo-keto reductase Tas	0.623775111	8.161526709	4.83E-05	0.000295897
b1688	ydiK	putative transporter YdiK	0.622283369	7.851124092	1.38E-06	1.17E-05
b1323	tyrR	DNA-binding transcriptional dual regulator TyrR	0.618296072	8.700096665	0.000102955	0.000579042
b1734	chbF	monoacetylchitobiose-6-phosphate hydrolase	0.618001651	4.540112742	0.115409271	0.224167149
b1485	ddpC	putative D,D-dipeptide ABC transporter membrane subunit DdpC	0.617759338	4.519228371	0.089887423	0.184882554
b2387	fryB	putative PTS enzyme IIB component FryB	0.617533759	2.313391638	0.442846842	0.601590402
b1914	uvrY	Phosphorylated DNA-binding transcriptional activator UvrY	0.617375201	8.219032218	2.22E-05	0.000144009
b1325	ycjG	L-Ala-DL-Glu epimerase	0.616548147	7.111806691	6.38E-05	0.000381135
b1844	exoX	exonuclease X	0.616338175	6.466504742	0.003089892	0.011037113
b1575	dicB	Qin prophage; cell division inhibition protein DicB	0.615575926	3.275525953	0.219247565	0.36199626
b0117	yacH	DUF3300 domain-containing protein YacH	0.615377414	6.047200455	0.012937359	0.037776746
b4726	yabR	protein YabR	0.615090574	2.262023759	0.487849219	0.640837341
b3103	yhaH	putative inner membrane protein	0.614082117	6.827881972	0.000949668	0.003914832
b2637	yfjT	CP4-57 prophage; uncharacterized protein YfjT	0.613748952	6.328230823	0.021040368	0.056543822
b1599	mdtI	multidrugspermidine efflux pump membrane subunit MdtI	0.613558718	6.505745754	0.002463866	0.009065194
b0982	etp	phosphotyrosine-protein phosphatase	0.613411872	4.186380626	0.086119118	0.178584355
b1245	oppC	murein tripeptide ABC transporter oligopeptide ABC transporter inner r	0.612331871	8.934759389	0.000175556	0.00090865
b2804	fucU	L-fucose mutarotase	0.61223632	5.821658622	0.008272981	0.025758259
b1378	ydbK	putative pyruvate-flavodoxin oxidoreductase	0.611140799	9.767382617	0.000228859	0.001131482
b0435	bolA	DNA-binding transcriptional dual regulator BolA	0.611121279	7.724843301	9.11E-06	6.51E-05
b1761	gdhA	glutamate dehydrogenase	0.610837993	8.324130024	6.29E-05	0.000376907
b1191	cvrA	putative K+:H+	0.610788572	7.326662109	9.95E-06	7.01E-05
b4056	yjbQ	UPF0047 protein YjbQ	0.61013865	8.07541905	2.50E-05	0.000160505
b1512	lsrR	DNA-binding transcriptional repressor LsrR	0.609709258	5.888714797	0.014593933	0.041488869

b1404	insI-2	IS30 transposase	0.609503404	8.182631958	4.72E-05	0.000290098
b4256	yjgM	putative acetyltransferase YjgM	0.608822874	6.594167412	0.000683136	0.002924193
b2663	gabP	GABP-MONOMER	0.608204039	6.271187335	0.015498325	0.043472111
b1568	ydfX	Qin prophage; uncharacterized protein YdfX	0.607585802	3.583846729	0.190417206	0.32813894
b4110	yjcZ	uncharacterized protein YjcZ	0.606491248	4.846324852	0.094920554	0.192299878
b2022	hisB	imidazoleglycerol-phosphate dehydratase histidinol-phosphatase	0.605670515	7.263546985	0.001493965	0.005821585
b4074	nrfE	putative cytochrome c-type biogenesis protein NrfE	0.604572446	5.176547946	0.041083152	0.098792002
b3003	yghA	NADP+-dependent aldehyde reductase	0.604454068	6.44783654	0.001983301	0.007490397
b2154	yeiG	S-formylglutathione hydrolase	0.603473914	7.272205768	7.91E-05	0.000460051
b0861	artM	L-arginine ABC transporter membrane subunit ArtM	0.603368421	7.266347274	2.17E-05	0.000141713
b2475	ypfJ	uncharacterized protein YpfJ	0.602991461	8.088883861	0.000100701	0.000569995
b4206	ytfB	cell division protein YtfB	0.602889975	7.638154044	7.13E-06	5.20E-05
b0076	leuO	DNA-binding transcriptional dual regulator LeuO	0.602593059	5.451334005	0.053405299	0.121744787
b4385	yjjJ	toxin YjjJ	0.601904191	7.419650503	0.000280658	0.001366157
b4301	sgcE	KpLE2 phage-like element; putative epimerase SgcE	0.600912439	4.510660532	0.136453664	0.25494834
b1511	lsrK	autoinducer-2 kinase	0.599888358	5.109893904	0.077652496	0.163801133
b0124	gcd	quinoprotein glucose dehydrogenase	0.599750076	9.447723769	0.002120661	0.007968272
b4445	omrB	small regulatory RNA OmrB	0.599070199	5.976091434	0.024070055	0.063634308
b3024	ygiW	BOF family protein YgiW	0.598802257	7.937416847	2.10E-05	0.00013766
b1483	ddpF	putative D,D-dipeptide ABC transporter ATP-binding subunit DdpF	0.598219713	5.245036428	0.02672696	0.069587962
b1661	cfa	cyclopropane fatty acyl phospholipid synthase	0.598193601	9.072909494	0.000177734	0.000917772
b1258	yciF	DUF892 domain-containing protein YciF	0.596946111	4.323590535	0.10285094	0.204820432
b1664	ydhQ	adhesin-like autotransporter YdhQ	0.595751511	8.677321361	0.000266855	0.001301837
b4141	yjeH	L-methioninebranched chain amino acid exporter	0.595169394	6.360181783	0.00236842	0.008801832
b2732	ygbA	protein YgbA	0.594317696	5.162680712	0.064032642	0.140439202
b3156	yhbS	putative acyltransferase with acyl-CoA N-acyltransferase domain	0.593654048	7.766433559	0.000140459	0.000754411
b2712	hypF	carbamoyl--[HypE] ligase	0.593621998	7.174155926	0.000617037	0.00268166
b1276	acnA	aconitate hydratase 1	0.591299129	9.127528585	0.000904661	0.003760903
b1614	ydgA	DUF945 domain-containing protein YdgA	0.590592548	9.391351193	0.000207083	0.0010425
b1247	oppF	murein tripeptide ABC transporter oligopeptide ABC transporter ATP bi	0.590039897	9.030122285	0.000212642	0.001062006
b2005	cbtA	CP4-44 prophage; cytoskeleton-binding toxin CbtA	0.58989555	5.182094604	0.046372787	0.108960007
b4251	bdcR	putative transcriptional regulator BdcR	0.589580729	5.680739942	0.027422623	0.071009315
b1620	malI	DNA-binding transcriptional repressor MalI	0.588994967	5.535088375	0.017573797	0.048492695
b3234	degQ	periplasmic serine endoprotease	0.588945734	8.280892324	0.000673735	0.00290199
b1167	ymgC	protein YmgC	0.58845036	4.552426929	0.093724776	0.190425627
b4122	fumB	fumarase B	0.587645378	6.53727569	0.008150439	0.025466518
b2279	nuoK	NADH:quinone oxidoreductase subunit K	0.587217868	7.313295784	5.51E-05	0.000334838
b3916	pfkA	6-phosphofructokinase I	0.586066336	10.01347825	9.88E-05	0.000563378
b3331	gspJ	Type II secretion system protein GspJ	0.585924506	5.120444621	0.053611575	0.122022156
b4248	yjgH	RutC family protein YjgH	0.584721708	5.189205988	0.053015263	0.121087629
b0155	clcA	chloride:H+ antiporter ClcA	0.584213339	8.612315356	0.000127737	0.000693674
b4284	insI-3	KpLE2 phage-like element; IS30 transposase	0.584133732	8.182541906	0.000101165	0.000570692
b2374	frc	formyl-CoA transferase	0.583377862	3.43491542	0.276172187	0.428425934
b4211	qorB	NAD(P)H:quinone oxidoreductase	0.583129163	5.553663675	0.037651186	0.092401326
b2359	yfdP	CPS-53 (KpLE1) prophage; protein YfdP	0.582053971	3.484618475	0.211219232	0.352697773
b4397	creA	PF05981 family protein CreA	0.581082385	6.705835623	0.001752274	0.006703889
b1733	chbG	chitin disaccharide deacetylase	0.580832813	5.339039323	0.046011221	0.108225648

b3068	mug	stationary phase mismatchuracil DNA glycosylase	0.58043721	6.719497211	0.005483021	0.018104482
b3354	yheU	UPF0270 family protein YheU	0.579489453	6.17454083	0.003918086	0.013588648
b2440	eutC	ethanolamine ammonia-lyase subunit β	0.578405863	5.990077733	0.008651486	0.026785632
b2494	bepA	β-barrel assembly-enhancing protease	0.577719811	8.597610552	0.000785995	0.00331123
b1832	msrC	free methionine-(R)-sulfoxide reductase	0.57719917	7.809935697	0.000213848	0.001065659
b0012	mbiA	uncharacterized protein MbiA	0.57626909	4.673687707	0.092671012	0.18889313
b2133	dld	quinone-dependent D-lactate dehydrogenase	0.57594251	9.075465121	0.00031183	0.001477179
b0646	djlB	putative chaperone	0.575809706	3.46676981	0.206599213	0.346819592
b0597	entH	proofreading thioesterase in enterobactin biosynthesis	0.575697456	4.422666967	0.097086557	0.196082868
b0997	torA	trimethylamine N-oxide reductase 1	0.574515106	5.116437939	0.098904692	0.198483733
b1394	paaG	putative ring 1,2-epoxyphenylacetyl-CoA isomerase (oxepin-CoA forming	0.57441438	3.428752628	0.247057382	0.395775886
b1906	yecH	DUF2492 domain-containing protein YecH	0.572663374	6.0644992	0.012968967	0.037844011
b3329	gspH	Type II secretion system protein GspH	0.572632143	5.050747117	0.101266042	0.202302975
b2803	fucK	L-fuculokinase	0.572391308	5.532717428	0.043203568	0.102937805
b2368	emrK	tripartite efflux pump membrane fusion protein EmrK	0.572278718	4.415613707	0.147837674	0.271281518
b0024	yaaY	DUF2575 domain-containing protein YaaY	0.572021244	5.704153005	0.022621845	0.060129707
b2434	ypeA	putative acetyltransferase YpeA	0.571612379	6.496896579	0.006468433	0.020891097
b2602	yfiL	DUF2799 domain-containing lipoprotein YfiL	0.571549017	6.525731371	0.029375855	0.075447586
b3719	yieL	putative hydrolase YieL	0.571148419	5.455344392	0.0527617	0.120697211
b3685	yidE	putative transport protein YidE	0.571058055	7.508326961	0.000108926	0.000606441
b3605	lldD	L-lactate dehydrogenase	0.570714375	6.675295553	0.003136952	0.011178081
b4221	tamB	translocation and assembly module subunit TamB	0.569820933	9.120211063	0.000276155	0.001345723
b2379	alaC	glutamate—pyruvate aminotransferase AlaC	0.569157823	7.535317944	0.000187158	0.000957478
b3459	panZ	acetyl-CoA-bound PanD maturation factor	0.566985239	6.678671668	0.001718518	0.006597616
b3591	selA	selenocysteine synthase	0.566404653	8.489056995	0.001157747	0.00464212
b0782	moaB	protein MoaB	0.565834218	7.718648318	0.000117534	0.000644612
b3026	qseC	sensory histidine kinase QseC - phosphorylated	0.565175513	7.001967974	0.002378922	0.008833424
b3105	yhaJ	DNA-binding transcriptional activator YhaJ	0.564859232	7.269024689	0.000336197	0.001577375
b2490	hyfJ	putative hydrogenase 4 assembly protein	0.564580955	4.464928985	0.151170621	0.276249293
b3513	mdtE	multidrug efflux pump membrane fusion protein MdtE	0.564577232	8.600475627	0.002350215	0.008748903
b3956	ppc	phosphoenolpyruvate carboxylase	0.564568323	10.40615952	0.000780571	0.003291521
b3045	insD-5	IS2 insertion element protein InsB	0.564276458	7.575247155	0.00021244	0.001062006
b2026	hisI	putative bifunctional phosphoribosyl-AMP cyclohydrolasephosphoribosyl	0.562413377	7.230661145	0.000141413	0.000757693
b0882	clpA	ATP-dependent Clp protease ATP-binding subunit ClpA	0.561543002	9.894929125	0.000288652	0.001394308
b1710	btuE	thioredoxinglutathione peroxidase	0.560413911	6.983584888	0.001017708	0.00414883
b0769	ybhH	putative isomerase YbhH	0.560365339	2.222838449	0.483383671	0.63705639
b3279	yrdA	protein YrdA	0.560152281	8.280190503	8.37E-05	0.0004842
b2901	bglA	6-phospho-β-glucosidase A	0.559457305	8.70947906	0.000477084	0.002129752
b2267	elaA	putative N-acetyltransferase ElaA	0.55845502	6.692054494	0.004340895	0.014765064
b4677	yobI	uncharacterized protein YobI	0.557646252	2.547866197	0.572880167	0.7118677
b2622	intA	CP4-57 prophage; integrase	0.557353925	8.17314887	0.000122997	0.000671236
b1555	ydfR	Qin prophage; protein YdfR	0.555927591	2.763736196	0.396675548	0.556200607
b4312	fimB	regulator for fimA	0.555490434	6.648868014	0.003071656	0.010989758
b0352	mhpE	MHPELY-MONOMER	0.554109353	7.130718399	0.00061945	0.002689155
b0518	fdrA	putative acyl-CoA synthetase FdrA	0.553539816	3.18514256	0.291406494	0.444561048
b3012	dkgA	methylglyoxal reductase DkgA	0.553029701	7.86632564	0.00011366	0.00062726
b1493	gadB	glutamate decarboxylase B	0.55236462	10.06575205	0.000376225	0.001733856

b3404	envZ	sensory histidine kinase EnvZ	0.55232614	7.848302317	6.85E-05	0.000406206
b3406	greB	transcription elongation factor GreB	0.551017749	6.731712981	0.001642648	0.00634496
b0563	tfaX	DLP12 prophage; protein TfaX	0.550387539	3.748885196	0.189601759	0.326988972
b1387	paaZ	oxepin-CoA hydrolase3-oxo-5,6-dehydrosuberyl-CoA semialdehyde dehy	0.549089686	3.281623384	0.337715758	0.496343233
b0995	torR	DNA-binding transcriptional dual regulator TorR	0.547874118	5.509399375	0.052245685	0.119888097
b2795	ppnN	nucleotide 5'-monophosphate nucleosidase	0.547456002	8.699234953	0.000978082	0.004009501
b4568	ytjA	DUF1328 domain-containing protein YtjA	0.545881259	6.66762409	0.003563345	0.012460113
b2957	ansB	L-asparaginase 2	0.544801505	6.577760028	0.010506351	0.031612188
b1981	shiA	SHIA-MONOMER	0.544302279	7.722651163	1.38E-05	9.41E-05
b2721	hycE	formate hydrogenlyase subunit HycE	0.543890097	4.816911502	0.103807646	0.205982363
b1302	puuE	4-aminobutyrate aminotransferase PuuE	0.543148588	3.905626144	0.192915355	0.331151358
b1849	purT	phosphoribosylglycinamide formyltransferase 2	0.542655562	5.403462706	0.063459855	0.139529511
b1773	ydjI	putative aldolase YdjI	0.542433855	4.030599007	0.24694674	0.395742234
b2063	yegH	inner membrane protein YegH	0.542020383	7.940139888	0.000124464	0.000678403
b2212	alkB	DNA oxidative demethylase	0.540811502	5.584236483	0.06939862	0.150046478
b3542	dppC	dipeptide ABC transporter membrane subunit DppC	0.540655446	5.863105447	0.056468995	0.127134428
b2488	hyfH	hydrogenase 4 component H	0.540523295	4.333598518	0.213301514	0.355234321
b0920	elyC	envelope biogenesis factor	0.539425576	5.950200327	0.045263897	0.106980785
b4015	aceA	isocitrate lyase	0.538910112	8.865654203	0.001164106	0.004659589
b4304	sgcC	putative PTS enzyme IIC component SgcC	0.538169346	4.274274163	0.157009446	0.284330068
b2767	ygcO	putative 4Fe-4S cluster-containing protein	0.536568543	4.629846734	0.172173305	0.30413805
b0776	bioF	8-amino-7-oxononanoate synthase	0.534532449	4.115749474	0.158859661	0.28732708
b0855	potG	putrescine ABC transporter ATP binding subunit	0.533219379	4.706031309	0.121031054	0.233239679
b0127	yadG	putative ABC transporter ATP-binding protein YadG	0.530638276	7.565937086	0.00010034	0.00056941
b4273	insD-6	KpLE2 phage-like element; IS2 insertion element protein InsB	0.530571599	7.896262265	0.000562439	0.002465909
b3408	feoA	ferrous iron transport protein A	0.52946645	6.290385373	0.006716595	0.021566376
b1025	dgcT	probable diguanylate cyclase DgcT	0.529207145	7.260553316	0.000928371	0.003837787
b4030	psiE	putative phosphate starvation-inducible protein	0.528117424	5.663379112	0.02699408	0.070105214
b0649	djlC	co-chaperone DjlC	0.527364234	5.713566394	0.03059036	0.078202918
b0154	hemL	glutamate-1-semialdehyde aminotransferase	0.527013922	8.806231619	0.001458724	0.005699351
b0209	yafD	endonucleaseexonuclease	0.526153511	8.528580841	0.000489416	0.002180394
b1486	ddpB	putative D,D-dipeptide ABC transporter membrane subunit DdpB	0.525955597	4.001275696	0.177722437	0.310996654
b2365	dsdX	DSDX-MONOMER	0.525953998	4.858113201	0.09462142	0.192070607
b4237	nrdG	anaerobic ribonucleoside-triphosphate reductase activating protein	0.525397698	5.507735172	0.085018677	0.176721968
b3355	prkB	putative phosphoribulokinase	0.524992683	7.687348427	5.77E-05	0.000348675
b2380	ypdA	sensory histidine kinase YbdA - his371 phosphorylated	0.523569339	7.368664785	0.000368266	0.001704291
b4477	dgoA	DEHYDDEOXPHOSGALACT-ALDOL-MONOMER	0.52280319	5.437786788	0.084644225	0.17610945
b1846	yebE	conserved inner membrane protein YebE	0.522748512	5.801296045	0.029435484	0.075556779
b4109	crfC	clamp-binding sister replication fork colocalization protein	0.522711504	5.70131949	0.069668295	0.150555812
b2457	eutM	putative ethanolamine catabolic microcompartment shell protein EutM	0.522186384	4.152939941	0.237339421	0.384372576
b0128	yadH	putative ABC transporter membrane subunit YadH	0.521247137	7.320820841	0.00028552	0.001383724
b0045	yaaU	putative transporter YaaU	0.520751198	3.7192044	0.259554463	0.409847266
b3136	agaS	putative galactosamine-6-phosphate deaminaseisomerase	0.519418689	5.921653048	0.212886002	0.354676112
b2790	yqcA	putative flavodoxin YqcA	0.518837524	6.866528269	0.001572844	0.006091321
b3774	ilvC	ketol-acid reductoisomerase (NADP+)	0.516245158	9.775168726	0.001866887	0.007093207
b1953	yodD	stress-induced protein	0.516231702	6.50818342	0.005876334	0.0192749
b0337	codA	cytosineisoguanine deaminase	0.516009252	8.290553844	0.00106526	0.004318753

b2407	xapA	xanthosine phosphorylase	0.51595706	3.140783531	0.491589019	0.643781134
b2452	eutH	putative ethanolamine permease EutH	0.513693987	4.113264081	0.339544352	0.498035985
b0866	ybjQ	putative heavy metal binding protein YbjQ	0.511660288	6.560520419	0.02247071	0.059836058
b2103	thiD	bifunctional hydroxymethylpyrimidine kinasephosphomethylpyrimidine l	0.510258696	8.117303852	0.000623767	0.002705238
b3437	gntK	D-gluconate kinase, thermostable	0.510127857	5.557428565	0.038298677	0.093626057
b0256	insI-1	IS30 transposase	0.508146627	8.309203647	0.000498731	0.002215188
b0828	iaaA	β cleavage product of IaaA	0.507071231	8.912064109	0.002401544	0.008901394
b4342	yjiT	putative uncharacterized protein YjiT	0.507023133	7.647185126	0.000141797	0.000757909
b4151	frdD	fumarate reductase membrane protein FrdD	0.506816554	6.240868212	0.060343525	0.133969612
b4390	nadR	DNA-binding transcriptional repressorNMN adenylyltransferase NadR	0.506563059	8.046064066	0.000954795	0.003928629
b0896	dmsC	dimethyl sulfoxide reductase subunit C	0.505540153	5.859951011	0.060261051	0.133896599
b2016	yeeZ	putative epimerase YeeZ	0.504432363	8.318054663	0.000740665	0.003138231
b0839	dacC	D-alanyl-D-alanine carboxypeptidase DacC	0.502737628	8.958176462	0.001734258	0.00664072
b0993	torS	sensory histidine kinase TorS	0.501762155	6.585499164	0.007687165	0.024254113
b1192	ldcA	murein L,D-carboxypeptidase	0.501663978	7.12793662	0.006992954	0.02234001
b4238	nrdD	anaerobic ribonucleoside-triphosphate reductase	0.501236007	8.113014883	0.009985464	0.030320374
b3653	gltS	glutamate:sodium symporter	0.500734368	7.63565648	8.91E-05	0.000513318
b3209	elbB	low activity glyoxalase ElbB	0.500700616	7.716747978	0.004018652	0.013844632
b2021	hisC	histidinol-phosphate aminotransferase	0.500485275	6.629261357	0.021601658	0.0578008
b1777	yeaC	DUF1315 domain-containing protein YeaC	0.500384785	6.53793642	0.012409022	0.036572652
b1847	yebF	secreted protein YebF	0.498654694	7.290380385	0.004245515	0.014472287
b0962	helD	DNA helicase IV	0.498240851	8.659251983	0.002798882	0.010137048
b3598	yibI	DUF3302 domain-containing protein YibI	0.497978999	5.761805392	0.036552181	0.090205635
b0284	paoC	aldehyde dehydrogenase: molybdenum cofactor-binding subunit	0.497886197	6.085725939	0.02426691	0.064039692
b3484	yhhI	putative transposase	0.497388853	5.928232253	0.032546621	0.082204422
b1087	yceF	m7GTP pyrophosphatase	0.496361593	7.353577931	0.003360323	0.011859172
b2383	fryA	putative PTS multiphosphoryl transfer protein FryA	0.495807174	5.249798141	0.099951185	0.200310704
b1908	yecA	UPF0149 family protein YecA	0.495426878	7.91156743	0.000404457	0.001840907
b1260	trpA	tryptophan synthase subunit α	0.495411616	5.941061601	0.043691856	0.103988974
b1489	dosP	oxygen-sensing c-di-GMP phosphodiesterase DosP	0.494853433	6.384717703	0.012594165	0.036970239
b4376	osmY	periplasmic chaperone OsmY	0.490953018	8.757698394	0.00404498	0.013919398
b4260	pepA	aminopeptidase AI	0.489321066	8.734969729	0.004592388	0.015477399
b0862	artQ	L-arginine ABC transporter membrane subunit ArtQ	0.489044553	7.055638552	0.004462493	0.015097245
b3451	ugpE	sn-glycerol 3-phosphate ABC transporter membrane subunit UgpE	0.488722833	5.48843537	0.114735457	0.223152883
b3676	yidH	conserved inner membrane protein YidH	0.48869914	5.652561109	0.054710875	0.123871032
b0199	metN	L-methionineD-methionine ABC transporter ATP binding subunit	0.488417744	7.95857718	0.001484739	0.005790743
b2333	yfcP	putative fimbrial protein YfcP	0.488072105	3.506521677	0.364612051	0.525553815
b1246	oppD	murein tripeptide ABC transporter oligopeptide ABC transporter ATP bi	0.486976496	9.186708797	0.003581593	0.012510075
b4192	ulaG	L-ascorbate-6-phosphate lactonase	0.485985454	5.655895739	0.056025976	0.126330278
b0042	fixB	putative electron transfer flavoprotein FixB	0.483948976	3.520349124	0.28165418	0.433729754
b2415	ptsH	HPr - phosphorylated	0.483618806	7.987500387	0.000766209	0.003237142
b0944	ycbF	putative fimbrial chaperone YcbF	0.483148716	5.686869844	0.080987883	0.16978229
b4016	aceK	isocitrate dehydrogenase kinasephosphatase	0.482053947	7.539689948	0.000423851	0.001909491
b1380	ldhA	D-lactate dehydrogenase	0.481683666	7.770760919	0.000989885	0.004054119
b4300	sgcR	KpLE2 phage-like element; putative DNA-binding transcriptional regulat	0.480860253	4.973620722	0.166067895	0.295999094
b2205	napG	ferredoxin-type protein NapG	0.479372315	4.85132605	0.151668962	0.27704529
b3356	yhfA	OsmC family protein YhfA	0.47932904	7.485830944	0.000348453	0.00162452

b0063	araB	ribulokinase	0.478986868	2.219886276	0.591420636	0.726522567
b0062	araA	L-arabinose isomerase	0.478986868	2.219886276	0.591420636	0.726522567
b0349	mhpC	2-hydroxy-6-ketonona-2,4-dienedioate hydrolase	0.478261404	6.672901816	0.004731619	0.01592233
b3678	yidJ	putative sulfatasephosphatase	0.477423457	5.908500494	0.034895921	0.087203225
b3718	yieK	putative glucosamine-6-phosphate deaminase YieK	0.474414253	5.561151662	0.085174073	0.176961663
b0716	ybgO	putative fimbrial protein YbgO	0.474255631	5.524990458	0.065828704	0.14366472
b1006	rutG	pyrimidine:H+ symporter	0.473930971	5.602920525	0.091862664	0.187765585
b0619	dpiB	sensory histidine kinase DpiB - phosphorylated	0.472500148	6.589298787	0.049877675	0.115414013
b2451	eutA	ethanolamine ammonia-lyase reactivase EutA	0.471154057	4.268609167	0.271183737	0.423127697
b2708	gutQ	D-arabinose 5-phosphate isomerase GutQ	0.470964875	7.105041085	0.010300734	0.031170486
b0481	ybaK	Cys-tRNAPro and Cys-tRNACys	0.470516789	7.082522929	0.004429408	0.015008316
b0243	proA	glutamate-5-semialdehyde dehydrogenase	0.470268906	8.852269352	0.002193499	0.008213995
b1356	racR	Rac prophage; DNA-binding transcriptional repressor RacR	0.469084353	7.660564112	0.000281416	0.001368339
b1889	motB	motility protein B	0.469016159	4.236506684	0.278045808	0.430370365
b1991	cobT	nicotinate-nucleotide—dimethylbenzimidazole phosphoribosyltra	0.467441212	7.545041186	0.001548405	0.006007214
b1852	zwf	GLU6PDEHYDROG-MONOMER	0.466768076	9.274920131	0.004293699	0.014615791
b2918	argK	methylmalonyl-CoA mutase-interacting GTPase YgfD	0.466624253	5.012661221	0.133475034	0.250549436
b4514	ybfQ	inactive transposase YbfQ	0.465248746	4.175496564	0.204123267	0.344233852
b4069	acs	acetyl-CoA synthetase (AMP-forming)	0.463615388	6.544377664	0.021397755	0.057359494
b1795	yeaQ	PF04226 family protein YeaQ	0.46158928	7.633899884	0.00087657	0.003658536
b1082	flgK	flagellar hook-filament junction protein 1	0.461256062	5.635280877	0.090862728	0.186239065
b2134	pbpG	peptidoglycan DD-endopeptidase PbpG	0.461208567	7.64087495	0.001281484	0.005068676
b1508	hipB	antitoxinDNA-binding transcriptional repressor HipB	0.460989239	5.033275134	0.141784605	0.262534421
b1923	fliC	flagellar filament structural protein	0.460602906	7.808068537	0.001096612	0.004417465
b0945	pyrD	dihydroorotate dehydrogenase, type 2	0.460538978	6.697253593	0.0214332	0.057419648
b0497	rhsD	protein RhsD	0.460132325	7.349813659	0.004020117	0.013844632
b0198	metI	L-methionineD-methionine ABC transporter membrane subunit	0.460054345	7.478858993	0.000548415	0.002411835
b4243	ridA	enamineimine deaminase, redox regulated chaperone	0.458980345	7.995402555	0.001839766	0.007014305
b3409	feoB	Fe2+ transporter FeoB	0.458649851	8.789142359	0.003615647	0.012619037
b1978	yeeJ	inverse autotransporter adhesin	0.458485696	8.165216104	0.002547193	0.009324923
b1160	iraM	anti-adaptor protein IraM, inhibitor of σS proteolysis	0.457837501	5.279470447	0.094952148	0.192299878
b3573	ysaA	putative electron transport protein YsaA	0.457085165	6.756066151	0.006068288	0.019728638
b3689	yidR	DUF3748 domain-containing galacturonate catabolism protein YidR	0.45705971	8.09303728	0.00244393	0.009006638
b3521	yhjC	putative DNA-binding transcriptional regulator YhjC	0.456758585	5.315746344	0.092734266	0.188934834
b1888	cheA	chemotaxis protein CheA	0.456085175	4.75897066	0.174084656	0.306575093
b2398	yfeC	putative DNA-binding transcriptional regulator YfeC	0.453863947	6.600538674	0.012538336	0.036842447
b1442	ydcU	putative ABC transporter membrane subunit YdcU	0.452841505	4.589405655	0.182426508	0.317216634
b1840	yebZ	putative inner membrane protein	0.452341065	7.951621901	0.007644207	0.024175626
b1478	adhP	ethanol dehydrogenase alcohol dehydrogenase	0.45044026	7.510636013	0.00424419	0.014472287
b1851	edd	PGLUCONDEHYDRAT-MONOMER	0.450133387	7.818306177	0.002946414	0.010567357
b4379	yjjW	putative glycyl-radical enzyme activating enzyme YjjW	0.449110668	5.61445585	0.103741559	0.205943788
b3153	yhbO	proteinnucleic acid deglycase 2	0.44849832	7.152150971	0.007142854	0.022769458
b2298	yfcC	putative transporter YfcC	0.4468498	5.888696031	0.11259528	0.219764882
b3771	ilvD	dihydroxy-acid dehydratase	0.446197733	9.795791588	0.007833365	0.024562717
b0043	fixC	putative oxidoreductase FixC	0.444637091	3.560086838	0.407318324	0.566575425
b3682	glvB	putative PTS enzyme II component GlvB	0.444407365	5.020219256	0.155472897	0.282056813
b3654	xanP	YICE-MONOMER	0.444192703	6.690875201	0.017669784	0.048696691

b2469	narQ	sensory histidine kinase NarQ - phosphorylated	0.444042853	6.979322105	0.014523838	0.041369512
b2682	ygaZ	L-valine exporter subunit YgaZ	0.443047323	7.113667776	0.008265198	0.025752189
b2842	kduD	putative 2-keto-3-deoxy-D-gluconate dehydrogenase	0.442530612	5.893200685	0.106774602	0.211016055
b2580	ung	uracil-DNA glycosylase	0.442465889	7.416369842	0.002598085	0.009487629
b2545	yphC	putative zinc-binding dehydrogenase YphC	0.441827078	5.695158557	0.075820955	0.160809866
b1243	oppA	oligopeptide ABC transporter periplasmic binding protein	0.441748687	10.18289456	0.007296971	0.023177068
b0851	nfsA	NADPH-dependent nitroreductase NfsA	0.441591767	7.561669756	0.001937689	0.007349568
b3379	php	putative hydrolase	0.441483727	5.282869459	0.202208477	0.341657262
b3438	gntR	DNA-binding transcriptional repressor GntR	0.440219096	8.328643674	0.002182405	0.008179386
b0963	mgsA	methylglyoxal synthase	0.439432268	7.809886358	0.000933262	0.0038544
b1034	ycdX	zinc-binding phosphatase	0.439303255	7.828848693	0.002530706	0.009279958
b4332	yjiJ	putative transporter YjiJ	0.438710362	6.071426186	0.063195713	0.139156645
b3517	gadA	glutamate decarboxylase A	0.43862312	10.16303759	0.005285979	0.017533882
b4673	ymjD	uncharacterized protein YmjD	0.438551799	2.894030206	0.531387421	0.678448659
b3530	bcsC	cellulose biosynthesis protein BcsC	0.435341239	8.503719134	0.012347966	0.036417013
b0926	ycbK	DUF882 domain-containing protein YcbK	0.434035976	8.122576431	0.005561951	0.018325383
b0846	rcdA	DNA-binding transcriptional regulator RcdA	0.43395866	4.633724537	0.206447915	0.346697431
b3210	arcB	sensory histidine kinase ArcB - his292 phosphorylated	0.433776629	9.021740887	0.007025051	0.022426321
b1596	ynfM	putative transporter YnfM	0.432993212	7.099213283	0.007578476	0.024019361
b2766	ygcN	putative oxidoreductase with FADNAD(P)-binding domain	0.432414112	6.187052076	0.076633002	0.162115335
b2542	hcaD	putative 3-phenylpropionatecinnamate dioxygenase ferredoxin reductas	0.432306331	5.830483717	0.073121141	0.156334061
b3541	dppD	dipeptide ABC transporter ATP binding subunit DppD	0.432223563	6.18755338	0.098112269	0.197521965
b0854	potF	putrescine ABC transporter periplasmic binding protein	0.430856572	6.099035905	0.041908121	0.100447531
b0189	rof	modulator of Rho-dependent transcription termination	0.430372659	7.316151825	0.002570449	0.009402265
b3518	ccp	cytochrome c peroxidase	0.429035101	6.549310459	0.025109814	0.065948738
b3376	yhfS	putative aminotransferase YhfS	0.427841959	5.601709808	0.137157646	0.25583059
b2427	murR	DNA-binding transcriptional dual regulator MurR	0.426974217	5.498064456	0.152966253	0.278723073
b2925	fbaA	fructose-bisphosphate aldolase class II	0.426325769	9.749384695	0.004944533	0.016488
b3350	kefB	KEFB-MONOMER	0.426144806	7.302831488	0.037925293	0.092948508
b2633	yfjQ	CP4-57 prophage; DUF932 domain-containing protein YfjQ	0.425560939	6.139010585	0.124669634	0.237398697
b4377	yjjU	putative patatin-like phospholipase YjjU	0.425070612	8.938091107	0.009796244	0.029807317
b0783	moaC	cyclic pyranopterin monophosphate synthase	0.424566736	7.53346608	0.007718223	0.024305247
b4116	adiY	DNA-binding transcriptional activator AdiY	0.423066006	7.77194919	0.001276636	0.00505502
b4025	pgi	glucose-6-phosphate isomerase	0.422884803	10.24863983	0.005585349	0.018388752
b1035	ycdY	chaperone protein YcdY	0.422567715	7.527931978	0.004099682	0.014074726
b3128	garD	GALACTARDEHYDRA-MONOMER	0.422407426	6.254562735	0.18712694	0.323099508
b3291	mscL	large conductance mechanosensitive channel	0.42117472	7.857041321	0.001147939	0.004607411
b0940	elfC	putative fimbrial usher protein ElfC	0.420403137	5.822595104	0.063081254	0.13897392
b1554	rrrQ	Qin prophage; putative lysozyme	0.42034277	3.244231756	0.473792962	0.628544449
b4601	ydgU	uncharacterized protein YdgU	0.420218341	6.94736707	0.017559886	0.048484612
b0291	ecpC	putative fimbrial usher protein EcpC	0.419630876	6.402986071	0.037786345	0.092681507
b0506	allR	DNA-binding transcriptional repressor AllR	0.419331466	8.176554965	0.002787398	0.010103746
b3125	garR	TSA-REDUCT-MONOMER	0.415563746	6.245356172	0.094917055	0.192299878
b3267	yhdV	lipoprotein YhdV	0.415553981	7.623158356	0.024229435	0.063979041
b4559	ghoT	toxin of the GhoTS toxin-antitoxin system	0.41298382	5.901263932	0.161091541	0.289582081
b4472	yhdP	outer membrane permeability factor YhdP	0.412302595	9.160308365	0.00880735	0.027153947
b1221	narL	Phosphorylated DNA-binding transcriptional dual regulator NarL	0.412007991	7.425197982	0.007417437	0.023542763

b1010	rutC	putative aminoacrylate peracid reductase	0.41088604	2.445495429	0.534197706	0.680356089
b3155	yhbQ	DNA damage response nuclease YhbQ	0.410723661	6.91066943	0.070618858	0.152171249
b4148	gdx	guanidinium exporter	0.410379068	6.846247413	0.011289354	0.033641446
b2643	yfjX	CP4-57 prophage; putative antirestriction protein	0.410290025	5.922862919	0.134414787	0.251992054
b4021	pepE	peptidase E	0.409504642	6.896072917	0.027192774	0.070496829
b0438	clpX	ATP-dependent Clp protease ATP-binding subunit ClpX	0.409371002	9.52891237	0.008156761	0.025468245
b3755	yieP	putative transcriptional regulator YieP	0.409240647	8.433993464	0.005209879	0.01730746
b2564	pdxJ	pyridoxine 5'-phosphate synthase	0.408320366	8.270424353	0.004018694	0.013844632
b0384	psiF	PsiF family protein	0.408083104	6.809313342	0.021040829	0.056543822
b3679	yidK	putative transporter YidK	0.408048467	5.429133258	0.171550285	0.303443313
b3208	mtgA	peptidoglycan glycosyltransferase MtgA	0.407928975	7.561013248	0.024873136	0.065482942
b4037	malM	maltose regulon periplasmic protein	0.407867393	6.045708319	0.132633959	0.249182523
b0753	ybgS	PF13985 family protein YbgS	0.407759211	7.306968483	0.003163613	0.011245854
b2551	glyA	serine hydroxymethyltransferase	0.407295251	9.492050338	0.009948433	0.030228721
b1755	ynjC	putative ABC transporter membrane subunit YnjC	0.407246144	5.793230145	0.1051579	0.208380668
b2914	rpiA	ribose-5-phosphate isomerase A	0.405701606	8.322665618	0.004762211	0.016000882
b1883	cheB	CheB-Pasp	0.405653283	3.744468779	0.519885697	0.66904994
b4403	yjtD	putative rRNA methyltransferase	0.405461999	6.788567138	0.020100097	0.054371004
b0044	fixX	putative ferredoxin FixX	0.404564997	2.961603977	0.542438409	0.685422317
b4158	yjeO	conserved inner membrane protein YjeO	0.404170549	5.736830768	0.137840261	0.256778377
b0164	yaeI	phosphodiesterase YaeI	0.40413504	6.180422445	0.057945913	0.129534788
b2029	gnd	6-phosphogluconate dehydrogenase, decarboxylating	0.403834264	10.11370105	0.009465447	0.028940407
b3656	yicI	α-D-xyloside xylohydrolase	0.403695074	6.692685583	0.026974586	0.070095819
b0425	panE	2-dehydropantoate 2-reductase	0.403318221	7.971545272	0.002432564	0.008972238
b4014	aceB	MALATE-SYNTHASE	0.40318787	9.349368705	0.014776847	0.041837052
b0876	ybjD	DUF2813 domain-containing protein YbjD	0.403175577	7.516680257	0.003879006	0.013463688
b4539	yoeB	ribosome-dependent mRNA interferase toxin YoeB	0.40264787	6.05112233	0.081866944	0.171381013
b0869	ybjT	putative NAD(P)-dependent oxidoreductase YbjT	0.401523023	7.710232056	0.005243866	0.01740727
b1326	mpaA	murein tripeptide amidase A	0.401181455	7.264953654	0.014912634	0.042114861
b1956	dgcQ	putative diguanylate cyclase DgcQ	0.400848494	7.820137699	0.010310429	0.031178455
b1389	paaB	phenylacetyl-CoA 1,2-epoxidase subunit B	0.400365601	2.270942773	0.736227903	0.834693559
b2259	pmrD	signal transduction protein PmrD	0.400195392	6.318455184	0.048766947	0.11314144
b0192	nlpE	lipoprotein NlpE	0.39997989	8.002316272	0.003259348	0.011548975
b2941	yqgD	DUF2684 domain-containing protein YqgD	0.399821864	5.925632335	0.08661059	0.179392113
b1446	ydcY	DUF2526 domain-containing protein YdcY	0.399791562	6.798533735	0.014539699	0.041387988
b4378	yjjV	putative DNase YjjV	0.399722158	8.394859185	0.007822461	0.024558756
b3455	livG	branched chain amino acidphenylalanine ABC transporter ATP binding s	0.398758899	5.370564445	0.141985597	0.262534421
b1219	ychN	DsrEF sulfur relay family protein YchN	0.39853431	6.973336075	0.018164183	0.049903464
b2818	argA	N-acetylglutamate synthase	0.398396962	6.481196473	0.053474136	0.121820593
b2636	yfjS	CP4-57 prophage; inner membrane lipoprotein YfjS	0.398075103	5.836548847	0.195565796	0.334033102
b1418	cybB	superoxide oxidase	0.3979899	6.375021495	0.091637136	0.187391365
b3770	ilvE	branched-chain-amino-acid aminotransferase	0.397713101	9.574423932	0.026634346	0.06941596
b0879	macB	ABC-type tripartite efflux pump ATP bindingmembrane subunit	0.39699779	6.918267807	0.027152067	0.070432653
b1083	flgL	flagellar hook-filament junction protein 2	0.396235991	5.683823448	0.101754863	0.203187572
b3053	glnE	fused glutamine synthetase deadenylaseglutamine synthetase adenylyl	0.396104447	9.275169417	0.011283647	0.033641446
b2369	evgA	Phosphorylated DNA-binding transcriptional activator EvgA	0.395960873	7.760565274	0.001601049	0.006195121
b0306	ykgE	putative lactate utilization oxidoreductase YkgE	0.395590906	5.764667851	0.126301533	0.239837105

b0785	moaE	molybdopterin synthase catalytic subunit		0.395348141	7.416922111	0.008466927	0.026287961
b1390	paaC	phenylacetyl-CoA 1,2-epoxidase, structural subunit		0.395194078	2.266788524	0.598768057	0.733507484
b4193	ulaA	L-ascorbate specific PTS enzyme IIC component		0.394090178	4.483882498	0.324877158	0.482615293
b1419	ydcA	protein YdcA		0.39316087	4.960519255	0.197169018	0.336230674
b1982	amn	AMP nucleosidase		0.392880744	7.945601293	0.005926834	0.019411701
b0237	pepD	peptidase D		0.392660331	9.646503013	0.013986241	0.040158441
b4223	yzfA		0	0.392126311	7.064770091	0.010693181	0.032137777
b0353	mhpT	3-hydroxyphenylpropionate3-hydroxycinnamate:H+		0.391916455	5.893672036	0.13713882	0.25583059
b0227	yafL	NlpCP60 family protein YafL		0.391432458	5.892212149	0.219883709	0.362910869
b2007	yeeX	DUF496 domain-containing protein YeeX		0.390898974	7.922528209	0.004381459	0.014891565
b0733	cydA	cytochrome bd-I ubiquinol oxidase subunit I		0.389240638	9.87383908	0.014494039	0.0413329
b3754	hsrA	putative transporter HsrA		0.388465079	7.651429005	0.012844333	0.037529933
b4144	yjeI	DUF4156 domain-containing lipoprotein YjeI		0.388385488	7.492274989	0.004917618	0.016410644
b2439	eutL	putative structural protein, ethanolamine utilization microcompartment		0.387961972	5.581702905	0.192347355	0.330561919
b3712	yieE	putative phosphopantetheinyl transferase		0.387506759	7.811177814	0.009196152	0.028195146
b4062	soxS	DNA-binding transcriptional dual regulator SoxS		0.387444841	6.113319685	0.079918643	0.167779748
b2520	yfhM	αsub2sub-macroglobulin		0.387427096	9.700550469	0.011836282	0.035095491
b1750	ydjX	DedA family protein YdjX		0.387191019	5.950999552	0.090321341	0.185473824
b2283	nuoG	NADH:quinone oxidoreductase subunit G		0.387032885	10.5870639	0.015669312	0.043895947
b4756	yqiD	protein YqiD		0.386432527	6.421696034	0.074248188	0.158207409
b0838	gstB	glutathione S-transferase GstB		0.385605262	7.198535769	0.012497783	0.036760634
b1477	yddM	putative DNA-binding transcriptional regulator YddM		0.385087465	5.658266284	0.12288449	0.235385082
b4265	idnT	L-idonate5-ketogluconate		0.384919962	5.36858108	0.147500585	0.27096274
b1327	ycjY	putative hydrolase YcjY		0.384199393	5.183995721	0.162819435	0.292107242
b4035	malK	maltose ABC transporter ATP binding subunit		0.384125394	4.754318697	0.352858038	0.513300902
b0424	yajL	proteinnucleic acid deglycase 3		0.38301415	7.217338837	0.010898833	0.032578433
b1129	phoQ	sensory histidine kinase PhoQ		0.382794016	8.733962235	0.020231645	0.054631629
b0599	hcxA	hydroxycarboxylate dehydrogenase A		0.381509598	6.469399231	0.05862899	0.130796863
b4662	sgrT	putative inhibitor of the PtsG glucose transporter		0.380578423	5.760661023	0.148382781	0.272168666
b4046	zur	DNA-binding transcriptional repressor Zur		0.380242552	7.116060775	0.018397181	0.050480768
b0242	proB	glutamate 5-kinase		0.379698492	8.714540601	0.014472712	0.0413329
b0145	dksA	RNA polymerase-binding transcription factor DksA		0.379635381	8.199442514	0.007045478	0.022475277
b0942	ycbU	putative fimbrial protein YcbU		0.379448394	4.001468081	0.36384295	0.525299747
b1774	ydjJ	putative zinc-binding dehydrogenase YdjJ		0.379291243	3.59574869	0.603742264	0.737520981
b0491	fetB	putative iron ABC exporter membrane subunit FetB		0.379080145	7.056955337	0.019258684	0.052421139
b1797	yeaR	DUF1971 domain-containing protein YeaR		0.378299842	7.16300012	0.02950465	0.075646358
b0786	ybhL	Bax1-I family protein YbhL		0.378273678	8.026027433	0.004427658	0.015008316
b1709	btuD	vitamin Bsub12sub ABC transporter ATP binding subunit		0.377489471	7.22610349	0.033822607	0.084893014
b4036	lamB	maltose outer membrane channel phage lambda receptor protein		0.376294179	5.757625434	0.150887794	0.275960899
b1108	ycfP	UPF0227 protein YcfP		0.37601465	7.941395213	0.005942081	0.01941171
b0864	artP	L-arginine ABC transporter ATP binding subunit		0.375981105	7.726447746	0.007189577	0.022885352
b0627	tatE	twin arginine protein translocation system - TatE protein		0.375924737	7.131789824	0.010116968	0.030656426
b0927	gloC	hydroxyacylglutathione hydrolase GloC		0.375553879	8.605392672	0.019000175	0.051909512
b2687	luxS	S-ribosylhomocysteine lyase		0.374321458	8.496878796	0.019723366	0.053520997
b2487	hyfG	hydrogenase 4 catalytic subunit HyfG		0.372634073	5.76673617	0.169692463	0.30147323
b3609	secB	SecB chaperone		0.372492032	8.462667872	0.017221177	0.047728498
b0020	nhaR	DNA-binding transcriptional activator NhaR		0.372435989	8.15500576	0.00763153	0.024170162

b2179	yejE	putative oligopeptide ABC transporter membrane subunit YejE	0.371680308	6.498808271	0.052310124	0.119973609
b2461	eutP	putative ethanolamine utilization acetate kinase EutP	0.371438218	3.56516272	0.619037914	0.750014378
b0903	pflB	pyruvate formate-lyase	0.371322443	10.8437463	0.013665367	0.03945886
b0046	kefF	regulator of KefC-mediated potassium transport and quinone oxidoredu	0.371122752	6.055900737	0.106901802	0.211147296
b3772	ilvA	threonine deaminase	0.370959171	9.566058393	0.035760567	0.088798034
b3721	bglB	6-phospho-β-glucosidase B	0.369895723	5.245007999	0.199546513	0.338545907
b4585	chiX	small regulatory RNA ChiX	0.368467485	5.45224434	0.165406604	0.295297274
b2315	folC	bifunctional folylpolyglutamate synthetase dihydrofolate synthetase	0.367645634	8.466317719	0.036203658	0.089646186
b2559	tadA	tRNA adenosine34 deaminase	0.367458079	7.866995602	0.004013813	0.013844632
b0959	sxy	transcriptional coactivator for CRP	0.3664788	6.509172698	0.039240283	0.09545226
b3515	gadW	DNA-binding transcriptional dual regulator GadW	0.366341044	8.980438624	0.021927156	0.058529862
b2416	ptsI	PTS enzyme I	0.366178054	10.1001387	0.022604639	0.06012017
b0375	iprA	putative DNA-binding transcriptional regulator IprA	0.365983412	6.27465764	0.181801178	0.316503234
b3332	gspK	Type II secretion system protein GspK	0.365759619	5.88398433	0.109915637	0.215530484
b1834	yebT	intermembrane transport protein YebT	0.36546995	8.184849076	0.01516544	0.042701158
b1841	yobA	CopC domain-containing protein	0.363208092	6.893114283	0.061125076	0.135475507
b2836	aas	fused 2-acylglycerophospho-ethanolamine acyltransferaseacyl-acyl carri	0.362693559	8.154385032	0.011461645	0.034076205
b1178	pliG	inhibitor of g-type lysozyme	0.362515897	5.140800523	0.241661153	0.389818776
b0941	elfG	putative fimbrial-like adhesin protein	0.361796423	5.00166562	0.228267896	0.373675477
b1969	hprR	phosphorylated DNA-binding transcriptional dual regulator HprR	0.361545539	5.972583447	0.121117879	0.233264112
b2278	nuoL	NADH:quinone oxidoreductase subunit L	0.361426046	9.606706566	0.023477424	0.062179261
b2990	hybG	hydrogenase 2 accessory protein	0.359616339	6.18123599	0.08164661	0.171000845
b0677	nagA	N-acetylglucosamine-6-phosphate deacetylase	0.358543431	8.284123985	0.018421776	0.050491171
b1992	cobS	COBS-MONOMER	0.358300992	6.996704201	0.040181901	0.096994584
b0935	ssuD	FMNHsub2sub-dependent alkanesulfonate monooxygenase	0.358220463	3.453926231	0.448712584	0.606537289
b1548	nohA	Qin prophage; putative prophage DNA-packaging protein NohA	0.358166237	3.26184786	0.550111072	0.691481346
b4465	yggP	putative zinc-binding dehydrogenase YggP	0.358149317	4.345522404	0.399435662	0.558779609
b4544	arnE	undecaprenyl-phosphate-α-L-Ara4N flippase - ArnE subunit	0.358079709	5.464352937	0.169839381	0.301504169
b3555	yiaG	putative DNA-binding transcriptional regulator YiaG	0.357655489	7.332244823	0.013265568	0.038594176
b1402	insD-2	IS2 insertion element protein InsB	0.357058283	8.078086882	0.01401729	0.040212042
b2702	srlA	sorbitol-specific PTS enzyme IICsub2sub component	0.35692654	4.212725673	0.545335628	0.687559902
b4242	mgtA	Mg2+ importing P-type ATPase	0.35667537	9.324025391	0.064401176	0.141037297
b2504	yfgG	protein YfgG	0.356216063	6.552826471	0.058838022	0.131196903
b2474	tmcA	tRNAMet cytidine acetyltransferase	0.356004289	8.370331932	0.018396621	0.050480768
b1662	ribC	riboflavin synthase	0.355403317	7.855886524	0.009519837	0.029086562
b1120	cobB	protein-lysine deacetylasedesuccinylase	0.355288334	7.502387519	0.007194995	0.022886097
b2107	rcnB	periplasmic protein involved in nickelcobalt export	0.355146203	6.711120748	0.122289839	0.234437533
b1760	ynjH	DUF1496 domain-containing protein YnjH	0.353912076	4.852400477	0.265976872	0.418044816
b0437	clpP	ATP-dependent Clp protease proteolytic subunit	0.353683247	8.572608313	0.026831908	0.069807234
b1542	ydfI	putative oxidoreductase YdfI	0.351561195	4.828558706	0.252865776	0.401883782
b3608	gpsA	glycerol-3-phosphate dehydrogenase	0.351336298	8.899392301	0.035201631	0.087656628
b1809	yoaB	RutC family protein YoaB	0.350928693	7.19931039	0.033080937	0.08331565
b1241	adhE	AdhE monomer	0.349753242	10.05196504	0.037937307	0.092948508
b0700	rhsC	rhs element protein RhsC	0.348542975	7.748213803	0.006793255	0.021796671
b4272	insC-6	KpLE2 phage-like element; IS2 insertion element repressor InsA	0.347674811	6.812303488	0.063388854	0.139442846
b1842	holE	DNA polymerase III subunit θ	0.346557448	5.769952366	0.153324993	0.279261487
b3657	yicJ	putative xyloside transporter YicJ	0.346503914	5.491126252	0.212562537	0.354404684

b3039	ygiD	4,5-DOPA dioxygenase extradiol	0.34627483	6.711700338	0.047355663	0.110680388
b2395	pdeA	putative c-di-GMP phosphodiesterase PdeA	0.346124925	7.180738713	0.029728944	0.076177183
b4718	gadF	small regulatory RNA GadF	0.346113887	6.475304737	0.059684185	0.132759001
b1008	rutE	putative malonic semialdehyde reductase	0.345636603	2.774870628	0.819982225	0.895592815
b0112	aroP	aromatic amino acid:H+ symporter AroP	0.345229158	7.663856635	0.007750905	0.024373393
b3888	yiiD	putative acetyltransferase YiiD	0.344951082	9.608812913	0.031432267	0.079938627
b3132	kbaZ	tagatose-1,6-bisphosphate aldolase 1 subunit KbaZ	0.343947534	5.915478162	0.386372983	0.547093239
b2782	mazF	endoribonuclease toxin MazF	0.343294359	7.048103049	0.032869405	0.082939392
b2284	nuoF	NADH:quinone oxidoreductase subunit F	0.343197204	9.577009477	0.036318001	0.08977826
b3052	hldE	fused heptose 7-phosphate kinaseheptose 1-phosphate adenyltransfera	0.343163738	8.792623969	0.032438558	0.081978381
b2781	mazG	nucleoside triphosphate pyrophosphohydrolase	0.343155065	7.753613229	0.013273863	0.038594176
b0827	moeA	molybdopterin molybdotransferase	0.34163836	8.577856927	0.049982957	0.115597043
b0561	tfaD	DLP12 prophage; putative tail fiber assembly protein TfaD	0.341356207	4.195874419	0.328622058	0.485889613
b1315	ycjS	putative oxidoreductase YcjS	0.341132283	4.691683485	0.320824295	0.477880993
b1346	xisR	Rac prophage; excisionase	0.341111077	1.302712531	1	1
b2282	nuoH	NADH:quinone oxidoreductase subunit H	0.341060521	9.0088871	0.064301393	0.140888661
b4215	ytfI	protein YtfI	0.340779316	5.527692144	0.31839104	0.475057938
b3335	gspO	Type II secretion system prepilin peptidase	0.340077451	6.930473067	0.033648113	0.084503083
b0493	ybbO	NADP+-dependent aldehyde reductase	0.33987304	7.066247795	0.055609309	0.125518967
b1640	anmK	anhydro-N-acetylmuramic acid kinase	0.339795728	7.413609985	0.018435281	0.050491171
b4402	yjjY	protein YjjY	0.339308648	7.425279233	0.015555113	0.043603697
b3677	yidI	putative inner membrane protein	0.339267732	5.637661968	0.163566198	0.293316313
b3054	ygiF	inorganic triphosphatase	0.338766576	8.356857186	0.028582111	0.073709123
b1309	ycjM	glucosylglycerate phosphorylase	0.338742217	4.140070204	0.395210626	0.555490625
b2711	norW	EG12450-MONOMER	0.338306963	5.192401777	0.337990571	0.49658182
b1248	yciU	DUF440 domain-containing protein YciU	0.337341235	6.877178004	0.031242667	0.079593984
b2431	yfeX	porphyrinogen peroxidase	0.337256245	8.585314761	0.034879983	0.087203225
b3202	rpoN	RNA polymerase, sigma 54 (sigma N) factor	0.33642318	9.477891063	0.027625801	0.071451617
b2491	hyfR	DNA-binding transcriptional activator HyfR	0.335876899	5.833447975	0.177345243	0.310706051
b2003	yeeT	CP4-44 prophage; DUF987 domain-containing protein YeeT	0.335499783	3.774675143	0.520118413	0.669091723
b2560	pgpC	phosphatidylglycerophosphatase C	0.335374715	8.382432905	0.021988263	0.058622089
b2755	cas1	multifunctional nuclease Cas1	0.335172025	5.598146185	0.201913695	0.341591986
b1779	gapA	glyceraldehyde-3-phosphate dehydrogenase A	0.33494928	9.961370963	0.029254406	0.07522318
b0652	gltL	glutamateaspartate ABC transporter ATP binding subunit	0.334776419	7.368340292	0.025299772	0.066368683
b0829	gsiA	glutathione ABC transporter ATP binding subunit GsiA	0.334684256	9.477422832	0.031146487	0.079394769
b2438	eutK	putative structural protein, ethanolamine utilization microcompartment	0.334559152	5.413827728	0.218500265	0.361032437
b2883	guaD	G7502-MONOMER	0.33447835	6.350953381	0.112513925	0.21970322
b1517	lsrF	3-hydroxy-2,4-pentadione 5-phosphate thiolase	0.334466019	4.043053698	0.392943956	0.55352386
b2992	hybE	hydrogenase 2-specific chaperone	0.334310255	7.25435924	0.019960878	0.054069958
b4230	ytfT	galactofuranose ABC transporter putative membrane subunit YtfT	0.334302898	3.844457616	0.481496909	0.635328408
b0513	ybbY	putative purine transporter	0.333536044	4.601182326	0.327963654	0.485078571
b2280	nuoJ	NADH:quinone oxidoreductase subunit J	0.332518298	8.291777476	0.036448938	0.090051518
b2072	pphC	protein-serinethreonine phosphatase PphC	0.332260435	3.317812135	0.555240811	0.695232042
b1957	yodC	protein YodC	0.331951716	6.2594123	0.16063888	0.289123789
b2757	casD	type I-E CRISPR system Cascade subunit CasD	0.331463605	5.005064732	0.388528045	0.549439884
b3439	yhhW	quercetin 2,3-dioxygenase	0.33105242	7.399278758	0.020982267	0.056520263
b4394	yjjX	ITPaseXTPase	0.330888289	6.596281106	0.070379176	0.151869043

b2448	yffQ	CPZ-55 prophage; uncharacterized protein YffQ	0.330882709	3.90659853	0.494412485	0.646381735
b0520	ylbF	DUF2877 domain-containing protein YlbF	0.330533924	3.914132253	0.419269907	0.57872702
b2079	baeR	Phosphorylated DNA-binding transcriptional activator BaeR	0.329740819	6.966351203	0.048099366	0.112136822
b2644	yfjY	CP4-57 prophage; RadC-like JAB domain-containing protein YfjY	0.329534281	5.74845564	0.296239991	0.449604523
b4554	yibT	protein YibT	0.328897677	6.702261396	0.088921771	0.183624706
b4135	yjdC	putative DNA-binding transcriptional regulator YjdC	0.328528898	7.852978333	0.019481311	0.052961814
b3847	pepQ	Xaa-Pro dipeptidase	0.328107375	10.08875372	0.028307551	0.073129222
b4603	rseX	small regulatory RNA RseX	0.327998111	3.035359121	0.646846956	0.771012233
b0857	potI	putrescine ABC transporter membrane subunit PotI	0.327249	4.231939566	0.484023784	0.637699148
b1168	pdeG	putative c-di-GMP phosphodiesterase PdeG	0.327107999	5.142506312	0.256016631	0.405711925
b2225	yfaP	DUF2135 domain-containing protein YfaP	0.326831421	4.67118488	0.295566477	0.449030406
b1447	ydcZ	putative inner membrane protein	0.325873725	6.554375723	0.076528714	0.162011728
b1202	ycgV	putative autotransporter adhesin YcgV	0.32563433	6.931656017	0.057251131	0.12836451
b4624	ryjB	small RNA RyjB	0.324666075	5.033640061	0.366404975	0.527102627
b1403	insC-2	IS2 insertion element repressor InsA	0.324470496	6.796692712	0.092783297	0.188947534
b3386	rpe	RIBULP3EPIM-MONOMER	0.324446454	7.837925081	0.022851563	0.060703761
b4008	gltV	tRNA-Glu(UUC)	0.323322131	8.864757149	0.114956033	0.223385072
b1180	ycgM	putative isomerasehydrolase	0.322947646	6.529882286	0.097981279	0.197348242
b1085	yceQ	DUF2655 domain-containing protein YceQ	0.32264018	7.516504018	0.019078066	0.052025733
b2706	gutM	DNA-binding transcriptional activator GutM	0.32237758	4.81474826	0.529867163	0.677095087
b2630	rnlA	CP4-57 prophage; RNase LS, toxin of the RnlAB toxin-antitoxin system	0.322294088	8.836162682	0.035814516	0.088882005
b2709	norR	DNA-binding transcriptional dual regulator NorR	0.321909391	6.945362487	0.139580357	0.259047489
b2645	yfjZ	CP4-57 prophage; putative antitoxin of the YpjF-YfjZ toxin-antitoxin syst	0.321694085	5.726254751	0.341180818	0.500050729
b3150	yraP	divisome-associated lipoprotein YraP	0.32100325	7.689511237	0.021042242	0.056543822
b2997	hybO	hydrogenase 2 small subunit	0.32091975	9.038118422	0.042140528	0.100895028
b0775	bioB	biotin synthase	0.320808683	4.305271081	0.396479316	0.556200607
b2993	hybD	putative hydrogenase 2 maturation protease	0.320641267	7.531897994	0.015380409	0.043223746
b0482	ybaP	TraB family protein YbaP	0.319870836	8.15004911	0.031111295	0.079394769
b1299	puuR	DNA-binding transcriptional repressor PuuR	0.319603106	5.495623529	0.221555797	0.364958877
b2783	mazE	antitoxin of the MazF-MazE toxin-antitoxin system MazE	0.319151977	6.956710361	0.048293195	0.112277228
b2271	yfbL	putative peptidase YfbL	0.319113245	2.737469894	0.613543263	0.745607902
b1136	icd	isocitrate dehydrogenase	0.318822231	9.860119468	0.050077264	0.115693941
b1597	asr	acid shock protein	0.318268188	8.548708893	0.047195554	0.110540249
b2677	proV	glycine betaine ABC transporter ATP binding subunit ProV	0.317939066	5.757689004	0.286906872	0.440129895
b0994	torT	TorT-TMAO	0.317261367	5.264438556	0.260982468	0.411539518
b4399	creC	sensory histidine kinase CreC	0.31696483	6.435489447	0.103668532	0.205891395
b0845	ybjJ	inner membrane protein YbjJ	0.3149312	5.341053485	0.25287274	0.401883782
b2926	pgk	PGK	0.314119368	9.753582068	0.039555829	0.096008239
b2927	epd	D-erythrose-4-phosphate dehydrogenase	0.313241417	9.055596326	0.052964554	0.121034423
b4303	sgcQ	KpLE2 phage-like element; putative nucleoside triphosphatase	0.313098023	6.823760005	0.055675542	0.12560425
b3642	pyrE	orotate phosphoribosyltransferase	0.312963243	6.171062194	0.144370261	0.266135575
b1670	ydhU	putative cytochrome YdhU	0.311260643	4.859856549	0.306047654	0.461003205
b3038	ygiC	putative acid—amine ligase YgiC	0.311140529	8.132260253	0.025021796	0.06576743
b3233	yhcB	DUF1043 domain-containing protein YhcB	0.310470531	8.233108239	0.060456489	0.134128341
b1179	ycgL	PF05166 family protein YcgL	0.310084892	6.592718661	0.093239417	0.189723746
b3942	katG	hydroperoxidase I	0.309517236	10.37142816	0.030391614	0.077763991
b3691	dgoT	putative D-galactonate transporter	0.309488531	5.455320681	0.254551839	0.403966471

b2668	ygaP	thiosulfate sulfurtransferase YgaP	0.308932555	6.318644024	0.234817559	0.381427344
b3152	yraR	putative nucleoside-diphosphate-sugar epimerase	0.308716445	6.897571333	0.110090328	0.215734042
b1813	nudL	putative NUDIX hydrolase with low 3-phosphohydroxypyruvate phosphat	0.308037846	6.500114421	0.154233611	0.280453621
b2245	yfaU	2-keto-3-deoxy-L-rhamnonate aldolase	0.307982234	5.030256255	0.385289545	0.546637241
b0970	yccA	modulator of FtsH protease	0.30787892	7.815049098	0.01658249	0.046103082
b2756	casE	pre-CRISPR RNA endonuclease	0.307784308	5.036851279	0.321659119	0.478801419
b1480	sra	30S ribosomal subunit protein S22	0.307408467	6.592138046	0.097807427	0.197312505
b3448	yhhA	DUF2756 domain-containing protein YhhA	0.307129591	7.324273934	0.028457823	0.073474438
b4191	ulaR	DNA-binding transcriptional repressor UlaR	0.305547891	6.461572193	0.107290348	0.211656338
b2615	nadK	NAD kinase	0.304950193	7.662626685	0.049098328	0.113789563
b0932	pepN	aminopeptidase N	0.304615714	10.09558674	0.056663325	0.127441967
b3407	yhgF	putative RNA-binding protein YhgF	0.304496892	9.215514479	0.079103816	0.166385587
b0163	yaeH	DUF3461 domain-containing protein YaeH	0.304473864	7.199899202	0.056398076	0.127039544
b3495	uspA	universal stress global stress response regulator	0.304366026	7.794002626	0.028910548	0.074469119
b4180	rlmB	23S rRNA 2'-O-ribose G2251 methyltransferase	0.304260696	7.247940932	0.034093614	0.085427528
b2417	crr	Enzyme IIAGlc	0.304107561	8.525944387	0.056799302	0.127617771
b3863	polA	DNA polymerase I	0.303879497	10.16726037	0.035009668	0.087326375
b1349	recT	Rac prophage; recombinase, DNA renaturation	0.302530981	4.092750246	0.438175176	0.597265639
b4297	yjhG	KpLE2 phage-like element; D-xylonate dehydratase	0.302166114	6.501983938	0.093377507	0.18980741
b1796	yoaG	DUF1869 domain-containing protein YoaG	0.302010556	6.561591401	0.121202236	0.233264112
b3384	trpS	tryptophan—tRNA ligase	0.301998201	8.989796053	0.078162208	0.164797589
b0483	ybaQ	putative DNA-binding transcriptional regulator YbaQ	0.301184482	6.702291352	0.089795577	0.184882554
b0329	yahO	DUF1471 domain-containing protein YahO	0.301124718	7.417102279	0.050063867	0.115693941
b1298	puuD	γ-glutamyl-γ-aminobutyrate hydrolase	0.301077275	5.073865623	0.373899929	0.535268543
b4742	ymjE	protein YmjE	0.300954293	2.822966517	0.647952138	0.771704529
b2196	ccmF	holocytochrome c synthetase membrane subunit CcmF	0.300558626	6.114157227	0.169226113	0.300899432
b1997	insC-3	CP4-44 prophage; IS2 insertion element repressor InsA	0.300525941	5.14325606	0.32885833	0.486076173
b3616	tdh	threonine dehydrogenase	0.300446741	9.14930628	0.069100734	0.149695654
b0361	insD-1	IS2 element protein	0.300441173	8.138916587	0.03243	0.081978381
b1130	phoP	Phosphorylated DNA-binding transcriptional dual regulator PhoP	0.30026279	8.482974304	0.052395743	0.120045255
b2923	argO	L-arginine exporter	0.300102256	7.022039341	0.05826397	0.130114024
b2478	dapA	4-hydroxy-tetrahydrodipicolinate synthase	0.300087495	9.135217346	0.060104376	0.133615721
b4577	sgrS	small regulatory RNA SgrS	0.300041396	5.849924299	0.226792656	0.371673932
b1791	nimT	2-nitroimidazole exporter	0.299739967	5.20599117	0.30854477	0.46413123
b2994	hybC	hydrogenase 2 large subunit	0.29964811	8.964103362	0.059480806	0.132496346
b3221	yhcH	DUF386 domain-containing protein YhcH	0.298946124	7.220822881	0.178511032	0.31212919
b2432	yfeY	DUF1131 domain-containing lipoprotein YfeY	0.298918136	7.545684406	0.057277029	0.12836451
b2743	pcm	L-isoaspartate protein carboxylmethyltransferase type II	0.29886633	8.131702885	0.060880173	0.135000484
b1464	yddE	PF02567 family protein YddE	0.298698519	7.238095727	0.047319124	0.110653567
b0830	gsiB	glutathione ABC transporter periplasmic binding protein	0.298612463	8.956252633	0.06476826	0.141700628
b1190	dadX	alanine racemase 2	0.298361317	8.58609317	0.135915965	0.254158824
b0648	ybeU	DUF1266 domain-containing protein YbeU	0.298129234	3.738330183	0.542181963	0.685422317
b2194	ccmH	holocytochrome c synthetase - thiol:disulfide oxidoreductase CcmH	0.297896583	5.827186892	0.205939273	0.345974845
b3362	yhfG	DUF2559 domain-containing protein YhfG	0.297675034	6.488597659	0.138852858	0.258120154
b4139	aspA	aspartate ammonia-lyase	0.297508823	9.318628789	0.10539552	0.208757837
b1507	hipA	phosphorylated serinethreonine kinase HipA	0.296589211	6.995263073	0.107730955	0.212209704
b1376	uspF	nucleotide binding filament protein	0.29594037	6.563259807	0.127318161	0.24155981

b4406	yaeP	UPF0253 family protein YaeP	0.293813012	7.28621901	0.057473198	0.128738798
b1046	clsC	cardiolipin synthase C	0.293471566	7.256385112	0.098416111	0.197862992
b4529	ydbJ	DUF333 domain-containing protein YdbJ	0.293178561	6.978780215	0.089901762	0.184882554
b4075	nrfF	putative formate-dependent nitrite reductase complex subunit NrfF	0.292522185	4.688908077	0.456452299	0.613212915
b0035	caiE	putative transferase CaiE	0.29217805	5.325251233	0.396706098	0.556200607
b2113	mrp	P-loop NTPase family protein Mrp	0.292117619	9.286920946	0.071116697	0.152788428
b2683	ygaH	L-valine exporter subunit YgaH	0.291693629	6.406692582	0.207324792	0.347509095
b2731	fhlA	DNA-binding transcriptional activator FhlA	0.291623233	7.6839365	0.040256784	0.09712224
b2329	aroC	chorismate synthase	0.291369912	8.324563908	0.085392593	0.177332218
b1057	yceJ	putative cytochrome b561	0.290451345	3.417053758	0.582931836	0.719699121
b2257	arnT	G7170-MONOMER	0.290340359	6.191431674	0.21373283	0.355416363
b0167	glnD	PII uridylyltransferase uridylyl removing enzyme	0.289056351	8.963928041	0.08386307	0.174813717
b0893	serS	serine—tRNA ligase	0.288622909	9.588549966	0.072397106	0.155011262
b0780	ybhK	putative transferase YbhK	0.288543004	7.750564166	0.075165479	0.159853366
b3336	bfr	bacterioferritin	0.288520092	8.611518736	0.073432366	0.15677171
b2741	rpoS	RNA polymerase, sigma S (sigma 38) factor	0.288437679	9.103944244	0.08334644	0.174147909
b3124	garK	glycerate 2-kinase 1	0.288095001	6.615772482	0.150996791	0.276045892
b3226	nanR	DNA-binding transcriptional dual regulator NanR	0.287184709	7.701267119	0.113532268	0.221300204
b2819	recD	exodeoxyribonuclease V subunit RecD	0.286645687	7.990366426	0.051498646	0.118358418
b2456	eutN	putative ethanolamine catabolic microcompartment shell protein EutN	0.28658139	2.957225799	0.84013619	0.906673497
b1235	rssB	RssB-P	0.286549726	7.68464087	0.048157275	0.112197556
b4085	alsE	D-allulose-6-phosphate 3-epimerase	0.286375511	5.084883796	0.30214957	0.455753451
b0322	yahH	putative uncharacterized protein YahH	0.284135817	5.572716456	0.331121064	0.488276385
b2285	nuoE	NADH:quinone oxidoreductase subunit E	0.28298896	8.67570262	0.082296211	0.172197996
b2546	yphD	putative ABC transporter membrane subunit YphD	0.282957897	4.825697945	0.447384217	0.605742858
b4717	micL	small regulatory RNA MicL-S	0.282550652	4.472905993	0.422417223	0.582520111
b0505	allA	ureidoglycolate lyase	0.282120732	3.876864383	0.519934393	0.66904994
b2480	bcp	EG10108-MONOMER	0.28177209	7.895409873	0.053920146	0.122520557
b3500	gor	glutathione reductase (NADPH)	0.281682748	8.686764371	0.102815393	0.204820432
b2276	nuoN	NADH:quinone oxidoreductase subunit N	0.281424802	8.986009817	0.074985051	0.159546505
b3133	agaV	N-acetyl-D-galactosamine specific PTS enzyme IIB component	0.280473805	5.59760401	0.555928115	0.695895273
b3139	agaC	galactosamine-specific PTS enzyme IIC component	0.280393095	5.622735266	0.541966845	0.685414958
b0812	dps	DNA protection during starvation protein	0.280101755	8.862340497	0.07927242	0.166660826
b1641	slyB	outer membrane lipoprotein SlyB	0.279609767	9.061761879	0.080081256	0.168041229
b3044	insC-5	IS2 insertion element repressor InsA	0.27916702	6.66080747	0.145355473	0.267839906
b2262	menB	1,4-dihydroxy-2-naphthoyl-CoA synthase	0.27810791	8.358076746	0.092439102	0.188507453
b3477	nikB	Ni(2+) ABC transporter membrane subunit NikB	0.277969575	5.945735495	0.215911483	0.357557838
b2082	ogrK	prophage P2 late control protein OgrK	0.277895557	4.399089551	0.437617627	0.596689877
b3511	hdeD	acid-resistance membrane protein	0.277732187	8.991511298	0.08328278	0.174097288
b2346	mlaA	intermembrane phospholipid transport system - outer membrane lipopı	0.277333268	7.848134402	0.045674932	0.107664082
b4143	groL	chaperonin GroEL	0.276624471	10.40689041	0.063899688	0.140217258
b3284	smg	DUF494 domain-containing protein Smg	0.276528686	8.208650844	0.068863561	0.149255092
b0283	paoD	molybdenum cofactor insertion chaperone for PaoABC	0.275162462	5.750540506	0.271109419	0.423127697
b3585	yiaU	putative LysR-type DNA-binding transcriptional regulator YiaU	0.274350445	6.642437645	0.123444132	0.236137713
b3412	bioH	pimeloyl-acyl carrier protein methyl ester esterase	0.273958329	6.892186596	0.154490359	0.28080483
b4663	azuC	uncharacterized protein AzuC	0.273629638	6.201383346	0.269943785	0.422175633
b2835	lplT	lysophospholipid transporter	0.273410751	7.106452788	0.072313901	0.154908235

b2738	ygbL	putative 3-oxo-tetronate 4-phosphate decarboxylase YgbL	0.273385036	4.591258477	0.513683545	0.664492486
b0210	yafE	putative S-adenosylmethionine-dependent methyltransferase	0.273268749	7.925324759	0.079073959	0.166385587
b0755	gpmA	2,3-bisphosphoglycerate-dependent phosphoglycerate mutase	0.272915206	9.086889649	0.12392723	0.23693232
b3421	rtcB	RNA-splicing ligase	0.272819371	5.988424116	0.215172822	0.35687326
b1104	ycfL	DUF1425 domain-containing protein YcfL	0.272776891	7.496687574	0.038115814	0.093263186
b3647	ligB	DNA ligase B	0.272467323	6.115688116	0.213629845	0.355416363
b1343	dbpA	ATP-dependent RNA helicase DbpA	0.2721383	6.720279115	0.13052741	0.24669457
b1571	ydfA	Qin prophage; DUF1391 domain-containing protein YdfA	0.271650435	4.224950673	0.469072807	0.624346229
b1165	ymgA	putative two-component system connector protein YmgA	0.27118236	6.12635258	0.224869564	0.369070307
b4667	ibsA	toxic peptide IbsA	0.270984546	4.915695581	0.465586901	0.621580335
b2861	insC-4	IS2 insertion element repressor InsA	0.269768126	6.954279825	0.097959641	0.197348242
b1014	putA	fused DNA-binding transcriptional repressor proline dehydrogenase	0.268923888	8.800276357	0.139455149	0.259047489
b3468	yhhN	conserved inner membrane enzyme YhhN	0.268630603	6.930375301	0.152854952	0.278635265
b2325	yfcL	PF08891 family protein YfcL	0.268447646	7.069770928	0.102022817	0.203630533
b2521	sseA	3-mercaptopyruvate sulfurtransferase	0.267477559	7.70403	0.052395189	0.120045255
b2412	zipA	cell division protein ZipA	0.267174451	8.979671036	0.095898307	0.193949165
b3766	ilvL	ilvXGMEDA operon leader peptide	0.266173795	9.348677258	0.199254242	0.338218946
b3349	slyD	FKBP-type peptidyl-prolyl cis-trans isomerase SlyD	0.265961558	8.951516577	0.097928117	0.197348242
b4222	ytfP	γ-glutamylamine cyclotransferase family protein YtfP	0.265628301	7.438849892	0.047397851	0.110720376
b2893	dsbC	protein disulfide isomerase DsbC	0.265193349	8.293849698	0.100729607	0.201504855
b1406	pdxI	pyridoxine 4-dehydrogenase	0.264627135	5.679932423	0.356729571	0.517568536
b4162	orn	oligoribonuclease	0.264312054	7.490475139	0.051898443	0.119153213
b4599	mgtS	small protein MgtS	0.263809787	6.952724741	0.135650265	0.253769458
b2258	arnF	undecaprenyl-phosphate-α-L-Ara4N flippase - ArnF subunit	0.262893726	6.241447152	0.235147254	0.38163904
b3380	yhfW	putative mutase YhfW	0.262459462	5.128325262	0.496775596	0.648145435
b2314	dedD	cell division protein DedD	0.262188019	7.94648092	0.06376932	0.140126449
b1388	paaA	phenylacetyl-CoA 1,2-epoxidase, monooxygenase subunit	0.261994025	2.544664836	0.696783104	0.807434537
b0650	hscC	chaperone protein HscC	0.261743998	4.898427247	0.423676978	0.58370901
b3383	yhfZ	putative DNA-binding transcriptional regulator YhfZ	0.261442446	6.382652097	0.192903341	0.331151358
b3222	nanK	N-acetylmannosamine kinase	0.260919172	7.195280026	0.247330813	0.3960702
b3848	yigZ	IMPACT family member YigZ	0.260768888	9.566108774	0.138045488	0.256943858
b3809	dapF	diaminopimelate epimerase	0.260653806	9.896812248	0.100827832	0.20161
b0187	yaeR	VOC domain-containing protein YaeR	0.260037988	7.061927128	0.091231426	0.18682131
b3617	kbl	2-amino-3-ketobutyrate CoA ligase	0.259446709	9.192718625	0.138009663	0.256943858
b4436	sibA	small RNA SibA	0.259355938	5.261733669	0.423987508	0.583734012
b4302	sgcA	putative PTS enzyme IIA component SgcA	0.259280145	4.578829466	0.533086183	0.679831166
b1726	yniB	uncharacterized protein YniB	0.258497588	7.1524246	0.083713252	0.174666354
b1412	azoR	FMN dependent NADH:quinone oxidoreductase	0.258367393	6.504147691	0.170185786	0.30163398
b1519	tam	trans-aconitate 2-methyltransferase	0.25819569	6.547277379	0.202167112	0.341657262
b1556	essQ	Qin prophage; putative S lysis protein	0.257719008	3.098659289	0.6883547	0.801728018
b2277	nuoM	NADH:quinone oxidoreductase subunit M	0.25688144	9.130568302	0.11293548	0.220331483
b1433	ydcO	putative transport protein YdcO	0.25669314	5.56580667	0.342807896	0.501656235
b3203	hpf	ribosome hibernation-promoting factor	0.256662701	8.079047228	0.089011717	0.183724512
b4093	phnO	aminoalkylphosphonate N-acetyltransferase	0.25573034	6.20047801	0.24568156	0.394287199
b1771	ydjG	NADH-dependent methylglyoxal reductase	0.255716647	4.348842582	0.560185669	0.700015421
b1650	nemA	N-ethylmaleimide reductase	0.255166143	7.667043588	0.075833781	0.160809866
b3794	rffM	UDPMANACATRANS-MONOMER	0.254774631	9.466611102	0.17288634	0.304951335

b3697	yidA	sugar phosphatase YidA	0.253788215	7.841291208	0.069389741	0.150046478
b1475	fdnH	formate dehydrogenase N subunit β	0.253477601	6.621932434	0.160496096	0.289123789
b3661	nlpA	lipoprotein-28	0.253337241	7.891222496	0.056543088	0.127236357
b3498	prlC	oligopeptidase A	0.252824102	9.226048053	0.128348814	0.243201722
b2331	smrB	putative endonuclease SmrB	0.252678218	7.139448719	0.104179671	0.206534911
b0289	ecpE	putative fimbrial chaperone EcpE	0.251894731	6.182623099	0.242677724	0.390879803
b4329	yjiG	Gate family protein YjiG	0.251334839	5.801850092	0.283224232	0.435085241
b0213	yafS	putative S-adenosyl-L-methionine-dependent methyltransferase	0.251296289	7.524219206	0.107763116	0.212209704
b0377	sbmA	peptide antibioticpeptide nucleic acid transporter	0.250554568	7.84388613	0.084563691	0.176024844
b2829	ptsP	PtsP-phosphorylated	0.250126093	9.321355546	0.134334867	0.25194921
b2784	relA	GDPGTP pyrophosphokinase	0.249215994	9.465373689	0.124694808	0.237398697
b2988	gss	fused glutathionylspermidine amidase glutathionylspermidine syntheta	0.248873191	10.03008161	0.107440668	0.211763638
b0153	fhuB	iron(III) hydroxamate ABC transporter membrane subunit	0.248810109	6.645712178	0.193647276	0.332020475
b0273	argF	CP4-6 prophage; ornithine carbamoyltransferase ArgF	0.248621935	6.719976746	0.195763656	0.3342214
b1993	cobU	cobinamide-P guanylyltransferase cobinamide kinase	0.248285286	6.779008655	0.165948967	0.295906579
b2779	eno	enolase	0.247736731	10.15884703	0.106190024	0.210082042
b0811	glnH	L-glutamine ABC transporter periplasmic binding protein	0.247359417	8.617838904	0.159804402	0.288327108
b2425	cysP	thiosulfatesulfate ABC transporter periplasmic binding protein CysP	0.246823816	9.169708445	0.13086166	0.247114163
b0611	rna	RNase I	0.246321128	7.755681405	0.076941481	0.162612081
b4124	dcuR	DcuR	0.245402877	6.661389053	0.185744255	0.32146644
b2450	yffS	CPZ-55 prophage; uncharacterized protein YffS	0.245386522	7.064522427	0.162453682	0.291676701
b2433	yfeZ	putative inner membrane protein	0.243811861	6.116940129	0.290883066	0.444069412
b2635	ypjK	CP4-57 prophage; uncharacterized protein YpjK	0.243340732	5.762203515	0.464581453	0.620801185
b1140	intE	e14 prophage; putative integrase	0.243266769	6.054719797	0.257094818	0.406982296
b4090	rpiB	allose-6-phosphate isomerase ribose-5-phosphate isomerase B	0.243130139	5.207875235	0.422574593	0.582520111
b1438	hicB	antitoxin of the HicA-HicB toxin-antitoxin system	0.242990752	5.901380722	0.295031023	0.448386219
b0041	fixA	putative electron transfer flavoprotein FixA	0.241916725	3.399470043	0.620079205	0.751061562
b0261	mmuM	MMUM-MONOMER	0.240767253	6.351741894	0.287298824	0.440425107
b2099	yegU	putative ADP-ribosylglycohydrolase YegU	0.240649782	5.068824124	0.507504521	0.657965852
b1271	yciK	putative oxidoreductase	0.240611952	6.495199355	0.183629992	0.31875737
b0704	ybfC	uncharacterized protein YbfC	0.239924293	4.958018374	0.410406504	0.569793935
b1324	tpx	lipid hydroperoxide peroxidase	0.239782464	8.429271837	0.109937633	0.215530484
b2539	hcaF	putative 3-phenylpropionatecinnamate dioxygenase subunit β	0.239213489	5.380314141	0.438594504	0.597652696
b0001	thrL	thr operon leader peptide	0.238606749	6.578254884	0.241611861	0.389818776
b0983	gfcE	putative exopolysaccharide export lipoprotein GfcE	0.238302217	5.530556218	0.36973886	0.530688253
b2821	ptrA	protease 3	0.238154187	8.686934027	0.134165015	0.251737586
b0489	qmcA	PHB domain-containing protein	0.238032664	6.760041258	0.203991659	0.34414336
b2296	ackA	acetate kinase	0.23784589	8.745783795	0.143715424	0.265482676
b4029	yjbH	YjbH family protein	0.237141499	6.07642391	0.416507403	0.575549353
b1594	mlc	DNA-binding transcriptional repressor Mlc	0.236621745	7.400694115	0.129671274	0.245391631
b2628	abpA	CP4-57 prophage; anti-bacteriophage protein	0.235998315	8.585857164	0.135611203	0.253769458
b1874	cutC	protein CutC	0.235954778	7.518626061	0.074013108	0.157782652
b0191	arfB	peptidyl-tRNA hydrolase, ribosome rescue factor	0.235836259	7.462472161	0.07258679	0.155342064
b2453	eutG	putative alcohol dehydrogenase EutG	0.235115458	3.498755178	0.715560006	0.817809326
b1590	ynfH	putative menaquinol dehydrogenase	0.23485679	6.427211096	0.232716218	0.378431714
b3910	yiiM	2-amino-6-N-hydroxylaminopurine resistance protein	0.234656192	9.362193465	0.177033986	0.310283863
b3688	yidQ	DUF1375 domain-containing putative lipoprotein YidQ	0.234100974	7.782526009	0.102474669	0.204439975

b4702	mgtL	leader peptide MgtL	0.233871297	6.04326214	0.338829728	0.497318234
b2631	rnlB	CP4-57 prophage; antitoxin RnlB	0.233615192	7.656898654	0.097237199	0.196297318
b0360	insC-1	IS2 element protein InsA	0.233407122	6.961893649	0.141321957	0.261828132
b0831	gsiC	glutathione ABC transporter membrane subunit GsiC	0.233388823	7.649101565	0.114854656	0.223286353
b3138	agaB	galactosamine-specific PTS enzyme IIB component	0.233061701	5.582503149	0.646530695	0.770843375
b3149	diaA	DnaA initiator-associating factor for replication initiation	0.23279056	7.846540449	0.104124876	0.206519014
b3960	argH	ARGSUCCINLYA-MONOMER	0.232709696	9.367759872	0.198350142	0.337351216
b1972	msrQ	periplasmic protein-L-methionine sulfoxide reductase heme binding sub	0.232597657	5.148690134	0.417010183	0.576063817
b0660	ybeZ	PhoH-like protein	0.232401221	8.787843414	0.183673556	0.31875737
b3781	trxA	oxidized thioredoxin	0.232243815	9.814958712	0.182343495	0.317197214
b4383	deoB	phosphopentomutase	0.231537904	9.417268849	0.15925545	0.287807127
b0318	yahD	ankyrin repeat-containing protein YahD	0.23123842	5.287937941	0.450348765	0.607668032
b2540	hcaC	putative 3-phenylpropionatecinnamate dioxygenase ferredoxin subunit	0.231049539	5.114590117	0.479776076	0.633436416
b1643	ydhI	DUF1656 domain-containing protein YdhI	0.23084862	6.33127747	0.250400235	0.399349347
b4604	yojO	uncharacterized protein YojO	0.230634592	2.58041433	0.887116942	0.935239177
b4669	ilvX	uncharacterized protein IlvX	0.230514686	9.224407175	0.282946117	0.435085241
b2544	yphB	putative aldose 1-epimerase YphB	0.230292479	5.93172298	0.386051117	0.546812859
b2739	ygbM	putative 2-oxo-tetronate isomerase YgbM	0.229435914	4.844564804	0.513941768	0.664631782
b4548	ypjJ	CP4-57 prophage; DUF987 domain-containing protein YpjJ	0.229400598	5.63472997	0.507594947	0.657965852
b3581	sgbH	3-keto-L-gulonate-6-phosphate decarboxylase SgbH	0.228794561	4.301096193	0.584169074	0.720420799
b2777	queE	putative 7-carboxy-7-deazaguanine synthase QueE	0.228312428	7.97169465	0.092303162	0.188317219
b0508	hyi	hydroxypyruvate isomerase	0.227607295	2.706728174	0.701890045	0.811003546
b1353	sieB	Rac prophage; phage superinfection exclusion protein	0.227487644	3.548160631	0.627445755	0.756464503
b1905	ftnA	ferritin iron storage protein	0.227332927	7.54822092	0.142000498	0.262534421
b2624	alpA	CP4-57 prophage; DNA-binding transcriptional activator AlpA	0.227051666	5.709469254	0.504695615	0.655747834
b2320	pdxB	erythronate-4-phosphate dehydrogenase	0.227027747	8.472609874	0.186029787	0.321582424
b3131	agaR	DNA-binding transcriptional repressor AgaR	0.226477329	8.160140739	0.126053291	0.23946871
b0826	moeB	molybdopterin-synthase adenylyltransferase	0.225801155	8.05789963	0.138526588	0.257622109
b3348	slyX	protein SlyX	0.225701774	6.435551835	0.238644466	0.385939677
b4396	rob	DNA-binding transcriptional dual regulator Rob	0.22536548	8.860209201	0.177560876	0.31096044
b4589	ylcI	DLP12 prophage; DUF3950 domain-containing protein YlcI	0.22529893	3.059357333	0.796933121	0.879506691
b2563	acpS	holo-[acyl-carrier-protein] synthase	0.225008756	6.755600578	0.196427057	0.335094844
b4737	ymdG	protein YmdG	0.22473055	2.509491604	0.879125309	0.93064781
b1836	yebV	DUF1480 domain-containing protein YebV	0.224667123	7.337824814	0.151845053	0.277137622
b4305	sgcX	KpLE2 phage-like element; putative endoglucanase with Zn-dependent e	0.224295768	5.728804304	0.358765854	0.519923096
b4121	yjdF	conserved inner membrane protein YjdF	0.224117079	5.807908371	0.334092892	0.491592524
b2942	metK	methionine adenosyltransferase	0.223962374	9.168424224	0.159077164	0.287602654
b3238	yhcN	DUF1471 domain-containing stress-induced protein YhcN	0.223835173	7.939261099	0.205118987	0.345385328
b3887	dtd	D-aminoacyl-tRNA deacylase	0.223677053	9.478804977	0.180371463	0.314758898
b0509	glxR	tartronate semialdehyde reductase 2	0.223535276	3.078807824	0.739328216	0.836742905
b2281	nuoI	NADH:quinone oxidoreductase subunit I	0.223529709	8.259351233	0.172218601	0.30413805
b2547	yphE	putative ABC transporter ATP-binding protein YphE	0.223392338	5.116916005	0.533418295	0.680013716
b4512	ybdD	PF04328 family protein YbdD	0.223207815	5.390206015	0.409983294	0.569385417
b4004	zraR	Phosphorylated DNA-binding transcriptional activator ZraR	0.222825936	9.35064746	0.215391078	0.356963817
b4470	cyuA	putative L-cysteine desulfidase CyuA	0.222628247	5.957232116	0.49136365	0.643781134
b0006	yaaA	peroxide stress resistance protein YaaA	0.222561519	7.221633875	0.179389965	0.313293787
b3405	ompR	OmpR-Phosphorylated	0.222177367	7.643842781	0.080464494	0.168765197

b3503	arsC	EG12237-MONOMER	0.221718043	5.300799084	0.441465066	0.600452331
b4468	glcE	glycolate dehydrogenase, putative FAD-binding subunit	0.220386503	4.335692341	0.583385216	0.71980689
b0129	yadI	putative PTS enzyme IIA component YadI	0.220353138	6.188091584	0.325596861	0.48335916
b3211	yhcC	radical SAM family oxidoreductase YhcC	0.220336209	6.628075906	0.44144216	0.600452331
b4009	rrlE	23S ribosomal RNA	0.21966229	11.61061147	0.135195602	0.253240808
b2737	ygbK	putative 3-oxo-tetronate kinase YgbK	0.219154376	5.272471479	0.49773291	0.648993148
b1479	maeA	malate dehydrogenase, NAD-requiring	0.218870103	9.824738859	0.170060862	0.30163398
b3915	fieF	Zn2+	0.218623347	9.480600821	0.175952131	0.309247077
b2675	nrdE	ribonucleoside-diphosphate reductase 2 subunit α	0.218494455	6.332123233	0.340798422	0.49970941
b1525	sad	NAD+-dependent succinate semialdehyde dehydrogenase	0.217939297	6.614760437	0.242034543	0.390136002
b2477	bamC	outer membrane protein assembly factor BamC	0.217565014	9.107791143	0.173598359	0.305962777
b2768	ygcP	putative anti-terminator regulatory protein	0.217497023	4.836203225	0.524935792	0.672740645
b2248	yfaX	putative DNA-binding transcriptional regulator YfaX	0.217131617	3.648311497	0.72584035	0.826136929
b3769	ilvM	acetolactate synthase II subunit IlvM	0.21711318	9.257971601	0.294287848	0.447564881
b0688	pgm	PHOSPHOGLUCMUT-MONOMER	0.21701033	9.453575484	0.192673722	0.330993962
b2707	srlR	DNA-binding transcriptional repressor SrlR	0.216902401	7.166534384	0.17011472	0.30163398
b1177	ycgJ	PF05666 family protein YcgJ	0.216072501	6.696355821	0.204369023	0.344385205
b3662	nepI	purine ribonucleoside exporter	0.215949256	6.683363239	0.20311651	0.342958165
b4295	yjhU	KpLE2 phage-like element; putative DNA-binding transcriptional regulat	0.215401078	8.28739142	0.159568369	0.288029755
b1611	fumC	fumarase C	0.215221363	7.980129713	0.16477923	0.294534535
b0695	kdpD	sensory histidine kinase KdpD	0.215174602	8.000766485	0.183264946	0.318423745
b4060	yjcB	uncharacterized protein YjcB	0.214712411	7.401155617	0.159574356	0.288029755
b4023	yjbD	DUF3811 domain-containing protein YjbD	0.214447419	6.325398499	0.300818562	0.454833545
b3478	nikC	Ni(2+) ABC transporter membrane subunit NikC	0.214282466	5.813859169	0.407255998	0.566575425
b0011	yaaW	putative enzyme-specific chaperone YaaW	0.214265347	5.098492816	0.527037825	0.674455651
b2235	nrdB	ribonucleoside-diphosphate reductase 1 subunit β	0.214183314	8.646913159	0.290611027	0.444069412
b0880	cspD	DNA replication inhibitor	0.213835938	6.983814743	0.176375817	0.309373241
b2195	ccmG	holocytochrome c synthetase - thiol:disulfide oxidoreductase CcmG	0.213468674	4.396230714	0.589539916	0.725221156
b1902	ftnB	putative ferritin-like protein	0.212810832	6.116646313	0.382076358	0.543624596
b3590	selB	selenocysteyl-tRNA-specific translation elongation factor	0.212605742	8.802625061	0.219066611	0.361832805
b1872	torZ	trimethylamine N-oxide reductase 2	0.211681587	6.234842952	0.298957467	0.452950314
b3850	hemG	protoporphyrinogen oxidase	0.211585494	9.569801868	0.241835044	0.3899568
b2705	srlD	sorbitol-6-phosphate 2-dehydrogenase	0.211508043	5.301231906	0.598413122	0.733276141
b0146	sfsA	putative DNA-binding transcriptional regulator of maltose metabolism	0.210678621	8.232391575	0.149397494	0.27368877
b3610	grxC	oxidized glutaredoxin 3	0.210319028	7.728375632	0.153929518	0.280114316
b0133	panC	pantothenate synthetase	0.210167625	8.751361542	0.186358315	0.321898263
b1112	bhsA	DUF1471 domain-containing multiple stress resistance outer membran	0.20980997	5.522195968	0.443425157	0.602005556
b2676	nrdF	ribonucleoside-diphosphate reductase 2 subunit β	0.209569395	5.76314768	0.494908528	0.646838706
b2704	srlB	sorbitol-specific PTS enzyme IIA component	0.209244595	4.159908102	0.687043176	0.801099932
b4165	glyY	tRNA-Gly(GCC)	0.209047802	6.183599744	0.315040963	0.471813382
b2502	ppx	exopolyphosphatase	0.208429791	8.595662546	0.19799084	0.336981325
b3021	mqsA	antitoxin of the MqsRA toxin-antitoxin system DNA-binding transcriptic	0.208255722	6.36556876	0.267187761	0.419357635
b4003	zraS	sensory histidine kinase ZraS - phosphorylated	0.208159086	9.074991654	0.316266912	0.473317002
b3660	yicL	putative inner membrane protein	0.207674216	7.246667281	0.161024597	0.289581912
b2307	hisM	lysinearginine	0.20676914	5.257115814	0.535331478	0.681120599
b3998	nfi	endonuclease V	0.206731149	9.267604519	0.275641539	0.427753039
b3811	xerC	site-specific tyrosine recombinase	0.206730271	9.856590327	0.195577705	0.334033102

b0888	trxB	thioredoxin reductase	0.206256383	8.891974021	0.224048162	0.367907882
b4411	ecnB	bacteriolytic entericidin B lipoprotein	0.206122024	7.344476367	0.159949198	0.288470469
b3997	hemE	uroporphyrinogen decarboxylase	0.205135873	9.487154485	0.235248105	0.38163904
b2215	ompC	outer membrane porin C	0.203422299	10.27169321	0.181391343	0.316038981
b4449	sraG	small regulatory RNA SraG	0.203169992	7.318744614	0.308901002	0.46450883
b4699	fnrS	small regulatory RNA FnrS	0.202819764	6.023696655	0.381196276	0.542722205
b1837	yebW	DUF1482 domain-containing protein YebW	0.202503563	6.116797703	0.347999407	0.507570988
b0492	cnoX	chaperedoxin	0.202064297	8.170055957	0.204305944	0.344385205
b4159	mscM	miniconductance mechanosensitive channel MscM	0.201896913	7.88307451	0.16163642	0.290327418
b2930	yggF	fructose 1,6-bisphosphatase YggF	0.201724957	4.804551842	0.603937361	0.737520981
b0719	ybgD	putative fimbrial protein YbgD	0.201176082	3.011860307	0.723726512	0.825433363
b0850	ybjC	DUF1418 domain-containing protein YbjC	0.200594993	6.120260138	0.371090237	0.532132953
b1234	rssA	putative patatin-like phospholipase RssA	0.200199743	7.647924755	0.13121354	0.247567428
b1707	ydiV	anti-FlhDC factor	0.199668353	7.493243238	0.182080223	0.316864085
b2632	yfjP	CP4-57 prophage; putative GTP-binding protein YfjP	0.199329696	6.017861735	0.521845528	0.670141945
b1431	ydcL	DUF3313 domain-containing lipoprotein YdcL	0.198900973	7.257520868	0.186203776	0.321757209
b2646	ypjF	CP4-57 prophage; toxin of the YpjF-YfjZ toxin-antitoxin system	0.198713377	5.892050856	0.525991904	0.673703004
b1150	ymfR	e14 prophage; IPR020297 domain-containing protein YmfR	0.198707818	1.812810432	1	1
b3812	yigB	5-amino-6-(5-phospho-D-ribitylamino)uracil phosphatase	0.198704468	9.627227391	0.24900258	0.398068181
b4189	bsmA	DUF1471 domain-containing putative lipoprotein BsmA	0.198092695	6.17130845	0.351100679	0.51175619
b3823	rhtC	L-threonine exporter	0.197466565	9.485707155	0.278107962	0.430370365
b2962	yggX	putative Fe2+-trafficking protein	0.197383919	6.952950833	0.21029205	0.351548429
b3382	yhfY	PRD domain-containing protein YhfY	0.19703	5.350559724	0.475324705	0.630020193
b3512	gadE	DNA-binding transcriptional activator GadE	0.196716056	8.974237112	0.216496642	0.358392454
b0081	mraZ	DNA-binding transcriptional repressor MraZ	0.196150512	8.017242459	0.183437721	0.31859856
b1598	ydgD	putative serine protease YdgD	0.195976139	9.095162829	0.246245021	0.394904384
b4572	ylbE	DUF1116 domain-containing protein YlbE	0.195525036	3.880935179	0.659043407	0.780074166
b2894	xerD	site-specific recombinase	0.195314497	8.168567561	0.224056625	0.367907882
b4475	rtcA	RNA 3'-terminal phosphate cyclase	0.195280538	6.666287008	0.27950489	0.431624376
b3540	dppF	dipeptide ABC transporter ATP binding subunit DppF	0.195042083	6.38454011	0.375432631	0.535898826
b4173	hflX	ribosome rescue factor HflX	0.194889986	9.401370742	0.224077983	0.367907882
b1708	nlpC	NlpCP60 family lipoprotein NlpC	0.194348934	7.191744266	0.191205024	0.32923954
b1103	hinT	purine nucleoside phosphoramidase	0.193981814	7.652976326	0.144166954	0.26598291
b1461	pptA	putative 4-oxalocrotonate tautomerase (4-OT)	0.19380349	5.218046957	0.536367656	0.681849468
b3826	yigL	phosphosugar phosphatase YigL	0.193626522	9.636484853	0.258158608	0.408227169
b4331	kptA	RNA 2'-phosphotransferase	0.193362632	5.893906674	0.414981438	0.574339513
b0134	panB	3-methyl-2-oxobutanoate hydroxymethyltransferase	0.192801666	8.397503723	0.227252129	0.372288738
b3395	hofM	DNA utilization protein HofM	0.192725459	5.512046784	0.456395217	0.613212915
b2621	ssrA	tmRNA	0.191525595	8.895455307	0.231143646	0.376846085
b4012	yjaB	peptidyl-lysine N-acetyltransferase YjaB	0.190608399	5.99666912	0.392515272	0.55330617
b3206	npr	Npr phosphorylated	0.190166067	7.689719435	0.270214704	0.422265926
b2817	amiC	N-acetylmuramoyl-L-alanine amidase C	0.189959549	8.193799264	0.19469503	0.333428456
b0311	betA	choline dehydrogenase	0.189417903	7.966320781	0.198500815	0.337397267
b1621	malX	PTS enzyme IIBC component MalX	0.189122735	6.51254134	0.291172053	0.444356936
b3569	xylR	DNA-binding transcriptional dual regulator XylR	0.188280133	6.78521384	0.26774624	0.419928828
b1826	mgrB	PhoQ kinase inhibitor	0.188116568	7.236922922	0.270936301	0.423127697
b4130	dtpC	dipeptidetripeptide:H+	0.187842991	6.463871044	0.323575954	0.481006005

b2132	bglX	β-D-glucoside glucohydrolase, periplasmic	0.187464059	8.451622827	0.251679605	0.400944309
b2462	eutS	putative ethanolamine catabolic microcompartment shell protein EutS	0.187430415	3.090735517	0.881362	0.931580855
b4408	csrB	small regulatory RNA CsrB	0.187423101	8.190294083	0.213612957	0.355416363
b3385	gph	GPH-MONOMER	0.187068839	8.075992903	0.288615002	0.441829139
b0474	adk	adenylate kinase	0.186510706	8.601562273	0.313280856	0.46981487
b2919	scpB	methylmalonyl-CoA decarboxylase	0.186354718	4.482030096	0.62998896	0.758188604
b3698	yidB	DUF937 domain-containing protein YidB	0.186218176	6.351270988	0.362461466	0.523990626
b0694	kdpE	KdpE-Phosphorylated	0.185776194	5.561306271	0.579186748	0.716889938
b1592	clcB	putative chloride:H+ antiporter ClcB	0.184788373	6.230933372	0.414506805	0.573927748
b1275	cysB	DNA-binding transcriptional dual regulator CysB	0.184446253	7.989327717	0.170178083	0.30163398
b4174	hflK	regulator of FtsH protease	0.182944301	9.38016858	0.261966198	0.412475308
b1677	lpp	murein lipoprotein	0.182903567	8.218563754	0.22873643	0.374165001
b1272	sohB	S49 peptidase family protein	0.182703777	8.537514711	0.234540954	0.381118259
b3761	trpT	tRNA-Trp(CCA)	0.182673423	9.336341874	0.387753254	0.548519903
b1675	fumD	fumarase D	0.182505842	5.877886182	0.535695605	0.681272288
b0316	yahB	putative LysR-type DNA-binding transcriptional regulator YahB	0.182330157	6.586269203	0.300557917	0.454595137
b2996	hybA	hydrogenase 2 iron-sulfur protein	0.182228639	8.832375232	0.249577441	0.398511538
b3970	rrlB	23S ribosomal RNA	0.181870738	12.01731532	0.213666115	0.355416363
b4558	yifL	putative lipoprotein YifL	0.181663024	9.477220543	0.317995973	0.474628878
b1823	cspC	stress protein, member of the CspA family	0.181267218	8.177767712	0.228702617	0.374165001
b2437	eutR	putative AraC-type transcriptional regulator EutR	0.180602194	6.101380471	0.472642279	0.627808796
b2938	speA	arginine decarboxylase, biosynthetic	0.180239708	9.793313844	0.267192079	0.419357635
b1958	yedI	conserved inner membrane protein YedI	0.178403268	7.276236003	0.283168163	0.435085241
b3810	yigA	DUF484 domain-containing protein YigA	0.178218895	9.78032403	0.27028845	0.422265926
b0308	ykgG	DUF162 domain-containing lactate utilization protein YkgG	0.177897261	6.318915937	0.385803317	0.546637241
b0171	pyrH	UMP kinase	0.177494946	8.78621571	0.301231785	0.454990876
b2736	ygbJ	putative L-threonate dehydrogenase	0.177023229	4.872320489	0.629487484	0.758188604
b1106	thiK	thiamine kinase	0.176674561	7.738036479	0.244763629	0.393386029
b4287	fecE	ferric citrate ABC transporter ATP binding subunit	0.176520068	5.470067132	0.530963648	0.678299915
b3759	rrfC	5S ribosomal RNA	0.17639972	9.65361681	0.356664153	0.517568536
b0860	artJ	L-arginine ABC transporter periplasmic binding protein	0.17454951	6.216991704	0.458207077	0.614888828
b2470	acrD	multidrug efflux pump RND permease AcrD	0.174160693	7.125849184	0.242905667	0.390879803
b1585	ynfC	UPF0257 family lipoprotein YnfC	0.174059517	6.918103829	0.279660305	0.431713373
b2822	recC	exodeoxyribonuclease V subunit RecC	0.173897612	8.971438241	0.301026642	0.454990876
b3859	srkA	stress response kinase A	0.173882485	9.410290298	0.292733294	0.44612271
b1411	ynbD	phosphatase domain-containing protein YnbD	0.172773706	5.076717117	0.55366868	0.694096501
b3681	glvG	putative inactive phospho-glucosidase	0.172712321	5.576133565	0.484160259	0.637699148
b4616	istR	small regulatory RNA IstR-1	0.172455289	5.223239485	0.580997126	0.717912765
b1064	grxB	oxidized glutaredoxin 2	0.172453686	7.253162099	0.270145188	0.422265926
b0881	clpS	specificity factor for ClpA-ClpP chaperone-protease complex	0.172055233	7.330426318	0.274834157	0.427100599
b1591	dmsD	redox enzyme maturation protein DmsD	0.171992392	6.689104181	0.323041015	0.480372543
b0534	sfmF	putative fimbrial protein SfmF	0.171634755	2.702703124	0.781043627	0.868716449
b3860	dsbA	thiol:disulfide oxidoreductase - DsbAsubreducedsub	0.171388122	9.5221132	0.292263377	0.445560362
b2286	nuoC	NADH:quinone oxidoreductase subunit CD	0.171289584	10.14660944	0.31012004	0.465707475
b3791	wecE	dTDP-4-dehydro-6-deoxy-D-glucose transaminase	0.171129364	9.827511873	0.295658299	0.449030406
b3583	sgbE	L-ribulose-5-phosphate 4-epimerase SgbE	0.170876629	4.715371645	0.663922068	0.783119405
b1833	yebS	intermembrane transport protein YebS	0.170860004	7.297850994	0.257470823	0.407431427

b0222	gmhA	D-sedoheptulose 7-phosphate isomerase	0.170329215	7.880462577	0.22915769	0.37471526
b3333	gspL	Type II secretion system protein GspL	0.17025603	6.753914755	0.35665641	0.517568536
b4596	yciZ	UPF0509 protein YciZ	0.170147336	4.999511524	0.614764076	0.746431608
b3792	wzxE	lipid III flippase	0.16980886	9.66702672	0.322003856	0.479153025
b2740	ygbN	putative transporter YgbN	0.169708722	5.708753926	0.586726892	0.722767642
b1967	hchA	proteinnucleic acid deglycase 1	0.169707149	6.863681073	0.452596912	0.609769718
b4213	cpdB	2'3' cyclic nucleotide phosphodiesterase3' nucleotidase	0.169431172	7.266800357	0.261583403	0.412166569
b2696	csrA	carbon storage regulator	0.169231902	7.754680545	0.23739888	0.384372576
b0832	gsiD	glutathione ABC transporter membrane subunit GsiD	0.168722444	7.536644575	0.260999015	0.411539518
b2495	yfgD	putative oxidoreductase YfgD	0.168690807	7.479527025	0.293377765	0.446642356
b2710	norV	anaerobic nitric oxide reductase flavorubredoxin	0.168575123	5.71030386	0.486664508	0.640042837
b0525	ppiB	peptidyl-prolyl cis-trans isomerase B	0.168328199	8.732708947	0.301192369	0.454990876
b3885	yihX	α-D-glucose-1-phosphate phosphatase	0.167853348	9.28078444	0.352137055	0.512758938
b1267	yciO	putative RNA-binding protein YciO	0.16777807	7.340039908	0.324725954	0.482553042
b3996	nudC	NADH pyrophosphatase	0.166858534	9.409972177	0.346444295	0.505971406
b4682	yqcG	cell envelope stress response protein YqcG	0.166384359	3.300892417	0.833071182	0.9034658
b3353	yheT	putative hydrolase YheT	0.166262584	7.352751662	0.303086432	0.45685442
b1340	smrA	DNA endonuclease SmrA	0.165957034	5.731132067	0.485148244	0.63842906
b3201	lptB	lipopolysaccharide transport system ATP binding protein	0.165689466	8.511379335	0.283050111	0.435085241
b4167	nnr	NAD(P)HX epimerase NAD(P)HX dehydratase	0.165622167	8.444167102	0.27253344	0.423972916
b3793	wzyE	enterobacterial common antigen polymerase	0.165330171	9.427537124	0.375404937	0.535898826
b2892	recJ	ssDNA-specific exonuclease RecJ	0.165082991	8.937273306	0.302139054	0.455753451
b1850	eda	KHGKDPG aldolase	0.164918342	7.948236311	0.269014352	0.421319037
b4309	nanS	N-acetyl-9-O	0.16371253	3.91783545	0.710993556	0.815334168
b3453	ugpB	sn-glycerol 3-phosphate ABC transporter periplasmic binding protein	0.163076941	6.151445535	0.618395086	0.749441204
b1119	nagK	N-acetyl-D-glucosamine kinase	0.162627943	7.655901443	0.22391166	0.367907882
b4401	arcA	Phosphorylated DNA-binding transcriptional dual regulator ArcA	0.162624619	9.033399132	0.331075603	0.488276385
b2221	atoD	acetyl-CoA:acetoacetyl-CoA transferase subunit α	0.162413667	3.72307159	0.750201996	0.844287997
b2501	ppk	polyphosphate kinase	0.162057277	9.119594791	0.327601014	0.485078571
b4179	rnr	RNase R	0.161789414	9.652313231	0.322278785	0.479400552
b2958	yggN	putative EcfF	0.161481741	7.619675772	0.242828931	0.390879803
b2345	yfdF	protein YfdF	0.161472393	6.184361312	0.445359335	0.604074182
b3999	yjaG	DUF416 domain-containing protein YjaG	0.16087789	9.462367006	0.368142682	0.529085267
b3758	rrlC	23S ribosomal RNA	0.160841483	12.08896467	0.275334069	0.427726923
b1062	pyrC	dihydroorotase	0.160631204	7.485019062	0.411859105	0.571148104
b2303	folX	dihydroneopterin triphosphate 2'-epimerase	0.16048126	7.079989777	0.393045821	0.55352386
b0136	yadK	putative fimbrial protein YadK	0.160169757	4.879416987	0.65883537	0.78003705
b3796	argX	tRNA-Arg(CCG)	0.160025153	9.403664793	0.406913989	0.566575425
b1009	rutD	putative aminoacrylate hydrolase	0.160010121	2.255717137	1	1
b4450	arcZ	small regulatory RNA ArcZ	0.159931861	6.745847172	0.59528077	0.730451528
b0258	ykfC	CP4-6 prophage; PF00078 domain-containing protein YkfC	0.159744097	6.992906507	0.326870231	0.484272507
b4704	arrS	small regulatory RNA ArrS	0.158869639	7.309875696	0.300158943	0.45430296
b4294	insA-7	KpLE2 phage-like element; IS1 repressor protein InsA	0.158493187	6.111619986	0.46379952	0.620319565
b0814	ompX	outer membrane protein X	0.158063165	9.237618374	0.318537121	0.475115335
b0019	nhaA	Na+:H+	0.15767657	8.744377224	0.345104046	0.504461774
b2289	lrhA	DNA-binding transcriptional dual regulator LrhA	0.157342121	7.142989117	0.277604256	0.430043084
b2011	sbcB	exodeoxyribonuclease I	0.156650094	8.001477099	0.314964679	0.471813382

b0734	cydB	cytochrome bd-I ubiquinol oxidase subunit II	0.156274846	9.449078393	0.365547346	0.526358008
b2995	hybB	hydrogenase 2 membrane subunit	0.155332518	8.31343534	0.294258375	0.447564881
b3827	bioP	biotin transporter	0.155166745	9.286152225	0.430846537	0.589870667
b3411	rpnA	recombination-promoting nuclease RpnA	0.15504049	6.46508541	0.401490239	0.560818625
b1961	dcm	DNA-cytosine methyltransferase	0.154936918	7.886345703	0.364132505	0.525323588
b4261	lptF	lipopolysaccharide transport system protein LptF	0.154888295	8.547405341	0.320280502	0.477232
b2017	yefM	YefM antitoxin of the YoeB-YefM toxin-antitoxin pair and DNA binding t	0.154887589	6.163116581	0.47298921	0.627855491
b2261	menC	O-SUCCINYLBENZOATE-COA-SYN-MONOMER	0.154790276	8.191798419	0.333660502	0.491200772
b3204	ptsN	PtsN-phosphorylated	0.154726272	8.175806313	0.280601217	0.432486402
b0172	frr	ribosome-recycling factor	0.154288763	8.809423947	0.325196456	0.482927128
b1515	lsrD	Autoinducer-2 ABC transporter membrane subunit LsrD	0.154265765	3.428158243	0.847358233	0.910471305
b3790	rffC	TDPFUCACTRANS-MONOMER	0.15423776	9.572642468	0.398625391	0.557999715
b3760	aspT	tRNA-Asp(GUC)	0.153737852	9.4633515	0.452017632	0.609361175
b2626	yfjJ	CP4-57 prophage; protein YfjJ	0.153640814	5.891888319	0.645277653	0.769907321
b3800	aslB	putative anaerobic sulfatase maturation enzyme AslB	0.15357683	9.228655498	0.448823856	0.606537289
b4142	groS	cochaperonin GroES	0.153255083	8.186054888	0.356985524	0.51776974
b0260	mmuP	B0260-MONOMER	0.152822241	5.790150644	0.568697468	0.708864857
b3664	adeQ	adenine transporter	0.152747791	5.084298598	0.627218455	0.756397017
b1450	mcbR	DNA-binding transcriptional dual regulator McbR	0.152381118	6.581357228	0.449602116	0.607141452
b1712	ihfA	integration host factor subunit α	0.152322734	7.907920329	0.268501508	0.420665067
b3886	yihY	putative inner membrane protein	0.152262736	9.444337879	0.373863985	0.535268543
b2455	eutE	acetaldehyde dehydrogenase (acetylating) EutE	0.15189284	3.869598975	0.804507349	0.884437238
b4481	rffT	G7800-MONOMER	0.151759556	9.490925994	0.411836746	0.571148104
b3849	trkH	TRKH-MONOMER	0.151530967	9.783637417	0.371106991	0.532132953
b2471	yffB	putative reductase YffB	0.151464629	6.973810309	0.38731678	0.548078072
b1056	yceI	protein YceI	0.151096792	5.268408783	0.635644123	0.761979039
b2018	hisL	his operon leader peptide	0.1508253	5.65458252	0.670496858	0.787298837
b3797	hisR	tRNA-His(GUG)	0.150428099	9.491651027	0.431650469	0.590559907
b1105	lpoB	outer membrane lipoprotein - activator of MrcB activity	0.149698763	7.795807339	0.300498635	0.454595137
b2104	thiM	hydroxyethylthiazole kinase	0.149607159	4.33040054	0.71359578	0.817044961
b1538	dcp	peptidyl-dipeptidase Dcp	0.149493038	9.377461457	0.365648927	0.526358008
b2326	epmC	EF-P-Lys34 hydroxylase	0.149485416	7.713632932	0.358823482	0.519923096
b2058	wcaB	putative colanic acid biosynthesis acyl transferase WcaB	0.149108569	3.503804984	0.818209593	0.894075162
b1618	uidR	DNA-binding transcriptional repressor UidR	0.148967416	7.54382197	0.327897584	0.485078571
b2332	yfcO	DUF2544 domain-containing protein YfcO	0.148860619	6.823483286	0.424281415	0.583734012
b4384	deoD	purine nucleoside phosphorylase	0.148839274	8.387239593	0.376036352	0.536587102
b1232	purU	formyltetrahydrofolate deformylase	0.147797871	8.44627229	0.369329534	0.530273136
b0784	moaD	MoaD-S	0.147589042	6.660620881	0.514188006	0.664755504
b4547	ypfN	UPF0370 protein YpfN	0.147469775	6.742817803	0.372956145	0.534263913
b1990	ldtA	L,D-transpeptidase LdtA	0.14744976	7.904488307	0.296035948	0.44944935
b4010	rrfE	5S ribosomal RNA	0.147041542	7.566603185	0.286832152	0.440129895
b2098	yegT	putative transporter YegT	0.146498264	4.371316939	0.68751249	0.801099932
b2717	hycI	hydrogenase 3 maturation protease	0.146481108	6.468940805	0.498924257	0.649587318
b3789	rffH	dTDP-glucose pyrophosphorylase 2	0.146291481	9.61381051	0.396905612	0.556207331
b3565	xylA	xylose isomerase	0.146176968	6.419737122	0.498718213	0.649587318
b3593	rhsA	rhs element protein RhsA	0.146051665	7.952695119	0.287822005	0.441073986
b1604	ydgH	DUF1471 domain-containing protein YdgH	0.145902605	8.067032537	0.406634339	0.566516442

b2100	yegV	putative sugar kinase YegV	0.145310958	5.856912934	0.614967075	0.746431608
b3808	yzcX	protein YzcX	0.145191487	9.429712272	0.447412788	0.605742858
b2647	ypjA	adhesin-like autotransporter YpjA	0.144824641	7.960462308	0.299670038	0.453874174
b0391	ppnP	nucleoside phosphorylase PpnP	0.142913215	6.525021873	0.506720013	0.657666542
b1487	ddpA	putative D,D-dipeptide ABC transporter periplasmic binding protein	0.142430251	4.453419596	0.696833108	0.807434537
b0951	pqiB	intermembrane transport protein PqiB	0.142031901	8.432053187	0.379512692	0.541023099
b1856	mepM	EG10013-MONOMER	0.141941351	7.786022351	0.409874407	0.569385417
b2008	yeeA	putative transporter YeeA	0.141612837	6.687568898	0.448279232	0.606537289
b2830	rppH	RNA pyrophosphohydrolase	0.141514736	8.087691338	0.301479573	0.45520941
b0095	ftsZ	cell division protein FtsZ	0.141244087	9.59312029	0.396275354	0.556200607
b2473	ypfH	esterase YpfH	0.140007431	7.813388906	0.334975197	0.492643403
b1146	ymfT	e14 prophage; putative DNA-binding transcriptional regulator YmfT	0.13916639	3.98735154	0.711202387	0.815361864
b0659	ybeY	endoribonuclease YbeY	0.139113007	7.701181057	0.352821227	0.513300902
b2754	cas2	CRISPR-associated endoribonuclease Cas2	0.138745583	4.729868141	0.692018957	0.803862598
b4217	ytfK	DUF1107 domain-containing protein YtfK	0.138690322	7.801318838	0.366365853	0.527102627
b4000	hupA	DNA-binding protein HU-α	0.138297788	9.521375889	0.434292505	0.593461229
b1128	roxA	ribosomal protein-arginine oxygenase	0.138032663	8.661774274	0.402913504	0.562397445
b3413	yhgH	DNA utilization protein YhgH	0.137999981	6.865035258	0.407085721	0.566575425
b3883	yihV	6-deoxy-6-sulfofructose kinase	0.13741571	9.060478123	0.480029049	0.633580942
b3378	yhfU	DUF2620 domain-containing protein YhfU	0.137302063	5.123212358	0.771556146	0.86271327
b3571	malS	ALPHA-AMYL-PERI-MONOMER	0.135255652	6.01075709	0.54537594	0.687559902
b3806	cyaA	ADENYLATECYC-MONOMER	0.135105067	10.7167902	0.342162243	0.50104355
b0026	ileS	isoleucine—tRNA ligase	0.134260269	10.44198599	0.392713668	0.553409143
b1107	nagZ	β-N-acetylhexosaminidase	0.133676569	8.59517349	0.415345993	0.574483885
b0655	gltI	glutamateaspartate ABC transporter periplasmic binding protein	0.133639341	8.420021538	0.425166312	0.584223239
b0091	murC	UDP-N-acetylmuramate—L-alanine ligase	0.133405254	8.961779978	0.444856844	0.603763592
b0082	rsmH	16S rRNA m4C1402 methyltransferase	0.133218901	8.609546196	0.396439232	0.556200607
b1336	abgT	p-aminobenzoyl glutamate:H+	0.13272035	4.837992657	0.709706102	0.814492446
b3145	yraK	putative fimbrial adhesin YraK	0.132643373	7.052300494	0.545745731	0.68782969
b1184	umuC	DNA polymerase V catalytic protein	0.132461046	6.532123674	0.516415467	0.666871575
b3357	crp	DNA-binding transcriptional dual regulator CRP	0.132148725	8.996517586	0.401488429	0.560818625
b3410	feoC	ferrous iron transport protein FeoC	0.132116399	6.506033339	0.466968405	0.622671551
b1189	dadA	D-amino acid dehydrogenase	0.132032105	8.952584601	0.498258636	0.649486826
b1296	puuP	B1296-MONOMER	0.131745711	4.964067446	0.66790891	0.78593226
b3137	kbaY	tagatose-1,6-bisphosphate aldolase 1 subunit KbaY	0.131297872	5.756014464	0.780284048	0.86818399
b4457	csrC	small regulatory RNA CsrC	0.130839646	9.51153467	0.465983129	0.621921256
b0137	yadL	putative fimbrial protein YadL	0.130563076	4.766390121	0.758131258	0.850738488
b0212	gloB	hydroxyacylglutathione hydrolase GloB	0.130515532	7.51667159	0.382730966	0.544114802
b0761	modE	DNA-binding transcriptional dual regulator ModE	0.130277347	7.238206205	0.390359199	0.551676013
b2831	mutH	DNA mismatch repair protein MutH	0.129622558	5.708722305	0.637921841	0.764087067
b2523	pepB	aminopeptidase B	0.129012704	9.056779395	0.435039286	0.593724404
b3958	argC	N-ACETYLGLUTPREDUCT-MONOMER	0.128843503	9.146090878	0.51703316	0.666871575
b2634	yfjR	CP4-57 prophage; putative DNA-binding transcriptional regulator YfjR	0.128824489	6.010361088	0.666817729	0.785066739
b4386	lplA	lipoate—protein ligase A	0.12881061	6.594892868	0.487395339	0.640622335
b0378	yaiW	surface-exposed outer membrane lipoprotein	0.127308945	8.037918875	0.391667032	0.552816479
b3391	hofQ	DNA utilization protein HofQ	0.127168557	5.651040122	0.645005568	0.7698887
b2556	glrK	sensory histidine kinase GlrK - phosphorylated	0.12697057	7.536672267	0.368510968	0.529442214

b4442	micA	small regulatory RNA MicA	0.126908425	6.953070993	0.490308064	0.643110547
b2524	iscX	accessory iron-sulfur cluster assembly protein IscX	0.126821377	7.424134852	0.364614119	0.525553815
b1175	minD	Z-ring positioning protein MinD	0.125904441	8.564401542	0.442288449	0.601016775
b3482	rhsB	rhs element protein RhsB	0.125834252	8.00186587	0.375280579	0.535898826
b0125	hpt	hypoxanthine phosphoribosyltransferase	0.124845979	8.052288432	0.416348821	0.575510346
b3799	proM	tRNA-Pro(UGG)	0.124451993	9.272769631	0.542999885	0.685935478
b3140	agaD	galactosamine-specific PTS enzyme IID component	0.124367961	5.695674977	0.795156005	0.878258153
b0093	ftsQ	cell division protein FtsQ	0.124353161	8.652460953	0.45395216	0.611194631
b3798	leuT	tRNA-Leu(CAG)	0.124352823	9.445704495	0.514586893	0.665076444
b0452	tesB	acyl-CoA thioesterase II	0.124287957	7.095184302	0.541820954	0.685414958
b2911	ssrS	6S RNA	0.124070176	8.207682205	0.41933131	0.57872702
b1149	ymfN	e14 prophage; chimeric replication proteinphage terminase YmfN	0.123978809	4.211893179	0.743778409	0.839667487
b1785	cdgI	putative c-di-GMP binding protein CdgI	0.123934037	5.444205138	0.658398085	0.77993763
b0476	aes	acetylesterase	0.123523606	5.962487457	0.607979946	0.740836324
b0204	rrlH	23S ribosomal RNA	0.123479547	11.87747395	0.409785929	0.569385417
b4483	tatD	3' → 5' ssDNARNA exonuclease TatD	0.123249607	9.534059973	0.491260873	0.643781134
b1645	ydhK	putative transporter YdhK	0.123206202	6.702246525	0.485806358	0.639104609
b0676	nagC	DNA-binding transcriptional dual regulator NagC	0.123203141	8.430807503	0.450313989	0.607668032
b3473	yhhS	putative transporter YhhS	0.123053099	7.029805394	0.475754704	0.63013175
b0877	ybjX	DUF535 domain-containing protein YbjX	0.12269593	8.606018353	0.434308772	0.593461229
b1094	acpP	octanoyl-ACP	0.122572453	8.440895106	0.448414744	0.606537289
b0521	ybcF	putative carbamate kinase	0.122548995	4.380527776	0.765769166	0.85721878
b1183	umuD	DNA polymerase V protein UmuD	0.122223464	5.017670382	0.67847369	0.793920313
b2769	ygcQ	putative flavoprotein	0.122090367	4.599036798	0.724187087	0.825532143
b0933	ssuB	aliphatic sulfonate ABC transporter ATP binding subunit	0.121583822	6.184817867	0.607850806	0.740836324
b0234	yafP	putative acyltransferase with acyl-CoA N-acyltransferase domain	0.121346626	5.734591026	0.690999007	0.803043068
b1115	ycfT	inner membrane protein YcfT	0.120701755	5.130675904	0.667330805	0.785461344
b2601	aroF	3-deoxy-7-phosphoheptulonate synthase, Tyr-sensitive	0.120624573	7.825679861	0.399870805	0.559034073
b1153	ymfQ	e14 prophage; DUF2313 domain-containing protein YmfQ	0.120169499	2.64429767	1	1
b2070	yegI	protein kinase YegI	0.119814898	5.519605528	0.669774516	0.78665988
b2426	ucpA	putative oxidoreductase	0.119757729	7.536767647	0.407989659	0.567330502
b3586	yiaV	putative membrane fusion protein	0.119518907	5.41875992	0.669056698	0.786025898
b2679	proX	glycine betaine ABC transporter periplasmic binding protein ProX	0.119470287	6.333479744	0.635625112	0.761979039
b2599	pheA	fused chorismate mutaseprephenate dehydratase	0.119383906	8.076796163	0.392243446	0.553276298
b0092	ddlB	D-alanine—D-alanine ligase B	0.119094444	8.519784489	0.483217327	0.637027321
b0965	yccU	putative CoA-binding protein with NAD(P)-binding Rossmann-fold doma	0.118919596	5.997665766	0.651182049	0.774298074
b1501	ydeP	putative oxidoreductase YdeP	0.118875086	4.999120101	0.742816934	0.839530442
b4485	ytfR	galactofuranose ABC transporter putative ATP binding subunit	0.118835882	4.802126279	0.730592198	0.829833947
b3144	yraJ	putative fimbrial usher protein YraJ	0.118741491	7.534778262	0.510271521	0.66070864
b2553	glnB	uridylyl-[protein PII]	0.118468074	7.421472897	0.399101097	0.55848854
b0052	pdxA	4-hydroxythreonine-4-phosphate dehydrogenase	0.118407992	8.277349257	0.42998968	0.589305848
b4175	hflC	regulator of FtsH protease	0.118407604	8.851310721	0.475917519	0.63013175
b3056	cca	fused tRNA nucleotidyltransferase 2',3'-cyclic phosphodiesterase	0.118370853	8.518165832	0.452492537	0.609769718
b3782	rhoL	rho operon leader peptide	0.118294161	9.499228343	0.538216935	0.682696747
b3683	glvC	putative PTS enzyme II component GlvC	0.118168569	5.226893589	0.690402137	0.802560672
b1285	pdeR	cyclic di-GMP phosphodiesterase PdeR	0.117713498	7.812875095	0.46827836	0.623845643
b0557	borD	DLP12 prophage; prophage lipoprotein BorD	0.117304313	4.016784741	0.758438661	0.850738488

b3923	uspD	universal stress protein D	0.117246021	9.605336636	0.488547349	0.641182089
b3034	nudF	ADP-sugar pyrophosphatase	0.116990597	7.746174095	0.439396085	0.598375607
b0228	rayT	REP-associated tyrosine transposase	0.116382406	5.751961584	0.700000363	0.809618092
b3959	argB	acetylglutamate kinase	0.116341951	9.166559551	0.540071469	0.683801416
b3275	rrlD	23S ribosomal RNA	0.116230641	11.90397943	0.437525245	0.596689877
b0030	rihC	ribonucleoside hydrolase RihC	0.116084083	5.350281054	0.732605101	0.831324288
b2678	proW	glycine betaine ABC transporter membrane subunit ProW	0.11595154	6.050681651	0.645032458	0.7698887
b0440	hupB	DNA-binding protein HU-β	0.115852844	8.071590007	0.424566432	0.583944174
b1952	dsrB	protein DsrB	0.115782811	6.541349071	0.538321834	0.682696747
b3143	yraI	putative fimbrial chaperone YraI	0.11525559	5.716593675	0.810035418	0.888523322
b4107	yjdN	PF06983 family protein YjdN	0.114755995	4.931031343	0.806475709	0.885484918
b2572	rseA	anti-sigma-E factor RseA	0.114709275	9.052922567	0.458571559	0.615190955
b0448	mdlA	ABC transporter family protein MdlA	0.114301857	7.041625366	0.46399289	0.620390251
b3035	tolC	outer membrane channel TolC	0.114061844	9.599320306	0.469384306	0.624572548
b3638	yicR	RadC-like JAB domain-containing protein YicR	0.113359333	6.223277802	0.631279146	0.759220221
b3480	nikE	Ni(2+) ABC transporter ATP binding subunit NikE	0.112865505	5.844155344	0.638376837	0.764424663
b2236	yfaE	ferredoxin-like diferric-tyrosyl radical cofactor maintenance protein YfaI	0.112559651	6.574005908	0.635056454	0.761979039
b3675	yidG	putative inner membrane protein	0.112292734	6.326720378	0.570069159	0.709572974
b4076	nrfG	putative formate-dependent nitrite reductase complex subunit NrfG	0.111680871	5.195124688	0.692068343	0.803862598
b0131	panD	PanD β cleavage product	0.111161437	7.735530307	0.407119491	0.566575425
b2666	yqaE	Pmp3 family protein YqaE	0.110960576	6.31293377	0.696972371	0.807434537
b1824	yobF	DUF2527 domain-containing protein YobF	0.110243313	7.859876611	0.463528477	0.620319565
b1284	yciT	putative DNA-binding transcriptional regulator YciT	0.110231881	6.281605596	0.587522258	0.723343773
b0013	yaaI	DUF2541 domain-containing protein YaaI	0.110144215	3.75208874	0.934320166	0.967180195
b2720	hycF	formate hydrogenlyase subunit HycF	0.109664868	3.894489084	0.857947734	0.916393629
b3858	yihD	DUF1040 domain-containing protein YihD	0.109509737	9.078116463	0.547847779	0.6893832
b1560	ydfU	Qin prophage; protein YdfU	0.109481712	3.832629458	0.792428089	0.876175811
b3029	ygiN	putative quinol monooxygenase YgiN	0.10924769	7.944384263	0.463708084	0.620319565
b3554	yiaF	DUF3053 domain-containing protein YiaF	0.109198182	8.941030607	0.496789162	0.648145435
b4172	hfq	RNA-binding protein Hfq	0.108752328	8.284005166	0.503970531	0.655191371
b2810	csdA	cysteine sulfinate desulfinase	0.107411341	8.172757472	0.449220675	0.606887785
b2959	yggL	protein YggL	0.107299221	7.916085379	0.475333468	0.630020193
b0178	skp	periplasmic chaperone Skp	0.107148741	8.966674046	0.521821353	0.670141945
b0220	ivy	periplasmic chaperone, inhibitor of vertebrate C-type lysozyme	0.106032459	8.418012732	0.498826776	0.649587318
b2882	xanQ	YGFO-MONOMER	0.10598516	5.81969462	0.674892316	0.790777488
b3867	hemN	coproporphyrinogen III dehydrogenase	0.10584141	9.901011168	0.475555252	0.630124981
b2589	rrlG	23S ribosomal RNA	0.10559817	11.89996761	0.478250516	0.631989234
b3788	rffG	dTDP-glucose 4,6-dehydratase 2	0.104755816	9.747590886	0.544368328	0.687074376
b4171	miaA	tRNA dimethylallyltransferase	0.104369781	8.756513577	0.540559027	0.684222507
b3087	ygjR	putative oxidoreductase YgjR	0.104213546	7.421344176	0.569817478	0.70945972
b0285	paoB	aldehyde dehydrogenase, FAD-binding subunit	0.104134616	5.493921487	0.714564349	0.81767738
b3592	yibF	glutathione transferase-like protein YibF	0.104087625	6.074393888	0.662956816	0.782817423
b3853	alaT	L-alanyl-tRNAalaT	0.103741354	9.513558263	0.579134821	0.716889938
b2625	yfjI	CP4-57 prophage; protein YfjI	0.103376818	6.188128012	0.73663014	0.834693559
b0083	ftsL	cell division protein FtsL	0.103304646	7.532368932	0.434688785	0.593478394
b4460	araH	arabinose ABC transporter membrane subunit	0.102871496	5.29899478	0.745967028	0.841515338
b0586	entF	seryl-[EntF peptidyl-carrier protein]	0.102743355	5.959166458	0.70482726	0.812055501

b4707	esrE	small RNA EsrE	0.101879572	9.460876689	0.579348613	0.716889938
b3347	fkpA	peptidyl-prolyl cis-trans isomerase FkpA	0.101286071	9.037919706	0.519163857	0.668448069
b0086	murF	UDP-NACMURALGLDAPAALIG-MONOMER	0.101020378	8.993332874	0.537912162	0.682696747
b2573	rpoE	RNA polymerase sigma E factor	0.100879198	8.875775535	0.521373608	0.670141945
b0293	ecpA	common pilus major subunit	0.100699615	5.773940078	0.710976801	0.815334168
b4081	mdtO	putative multidrug efflux pump subunit MdtO	0.10017138	5.528162546	0.674667559	0.790777488
b2809	ygdI	DUF903 domain-containing lipoprotein YgdI	0.099654424	7.444317531	0.501731102	0.652664353
b3831	udp	uridine phosphorylase	0.099437187	9.828948862	0.552116563	0.693123738
b0094	ftsA	cell division protein FtsA	0.09926773	9.250326943	0.539537106	0.683320804
b0498	ybbC	PF15631 family protein YbbC	0.099148472	4.742825142	0.760230274	0.852531537
b1622	malY	negative regulator of MalT activitycystathionine β-lyase	0.098958932	7.302479199	0.511312729	0.661813457
b3510	hdeA	periplasmic acid stress chaperone HdeA	0.098904796	8.801499852	0.555205531	0.695232042
b3795	yifK	putative transporter YifK	0.098873604	9.754852386	0.557942593	0.697823384
b3785	wzzE	enterobacterial common antigen polysaccharide co-polymerase	0.09871067	9.7213236	0.57171049	0.71098847
b1145	ymfK	e14 prophage; putative repressor protein YmfK	0.098692672	7.329938932	0.526980372	0.674455651
b3835	ubiB	ubiquinone biosynthesis protein UbiB	0.098608676	9.73506005	0.557040411	0.697073328
b3720	bglH	carbohydrate-specific outer membrane porin, cryptic	0.098343807	5.304303768	0.747016156	0.841990383
b4094	phnN	EG10723-MONOMER	0.098050284	5.11774686	0.784467356	0.870863308
b3980	tufB	translation elongation factor Tu 2	0.09754987	10.84001065	0.504675979	0.655747834
b1656	sodB	superoxide dismutase (Fe)	0.095665384	9.022944048	0.57102979	0.710368138
b3852	ileT	tRNA-Ile(GAU)	0.095588697	9.486672266	0.604048894	0.737520981
b2360	yfdQ	CPS-53 (KpLE1) prophage; protein YfdQ	0.094763111	2.766307346	0.900629845	0.943364357
b0462	acrB	multidrug efflux pump RND permease AcrB	0.094339673	10.03376952	0.547915209	0.6893832
b3229	sspA	stringent starvation protein A	0.093105188	8.878052484	0.569571593	0.70945972
b0344	lacZ	β-galactosidase	0.092685946	5.136743328	0.786568679	0.871494332
b4473	smf	protein Smf	0.092439122	7.720776408	0.534213458	0.680356089
b4045	yjbJ	putative stress response protein	0.092180299	7.75137488	0.479311046	0.633092678
b4706	iroK	protein IroK	0.092047222	4.459137695	0.814919314	0.891444195
b3198	kdsC	3-deoxy-D-manno-octulosonate 8-phosphate phosphatase KdsC	0.091802798	8.390222854	0.538278034	0.682696747
b3961	oxyR	DNA-binding transcriptional dual regulator OxyR	0.091764067	9.582838056	0.572304934	0.711353121
b0463	acrA	multidrug efflux pump membrane fusion lipoprotein AcrA	0.091662651	9.001256771	0.576377769	0.714805576
b4456	glmZ	small regulatory RNA GlmZ	0.091359695	9.507141218	0.633933219	0.761264884
b4058	uvrA	excision nuclease subunit A	0.091338264	9.747083772	0.582445694	0.719300067
b3037	ygiB	DUF1190 domain-containing protein YgiB	0.091142452	8.006922004	0.570622296	0.710061284
b1765	ydjA	putative oxidoreductase	0.090770538	6.963063138	0.692853041	0.804350822
b3338	chiA	endochitinase	0.089220576	7.194155081	0.574408943	0.713365818
b2304	yfcH	putative NAD-dependent epimerase YfcH	0.089164312	7.829042629	0.521821743	0.670141945
b0946	zapC	cell division protein ZapC	0.088474475	6.643363096	0.601278706	0.736378776
b3123	rnpB	RNase P catalytic RNA component	0.088438247	8.671706344	0.603286328	0.737520981
b0608	ybdR	putative Zn2+-dependent alcohol dehydrogenase YbdR	0.087474449	5.725074363	0.71957463	0.821474656
b4150	ampC	β-lactamase	0.08707679	6.846898774	0.627709674	0.756576088
b3951	pflD	putative formate acetyltransferase 2	0.086842281	9.104690079	0.656344591	0.77896811
b2330	prmB	50S ribosomal subunit protein L3 N5	0.086794373	8.218338386	0.624275949	0.754134876
b3532	bcsB	cellulose synthase periplasmic subunit	0.08678711	6.233613348	0.68453219	0.798892313
b1268	yciQ	DUF2207 domain-containing protein YciQ	0.086603904	7.016338344	0.603994169	0.737520981
b1778	msrB	methionine sulfoxide reductase B	0.086212425	6.838962505	0.749156326	0.843541235
b3909	kdgT	2-dehydro-3-deoxy-D-gluconate:H+ symporter	0.086078371	9.068636345	0.657180981	0.7796908

b3200	lptA	lipopolysaccharide transport system protein LptA	0.08576702	8.6387392	0.585795962	0.722023778
b0934	ssuC	aliphatic sulfonate ABC transporter membrane subunit	0.085337025	3.439252246	1	1
b0615	citF	citrate lyase α subunit	0.085309591	2.911682706	1	1
b4212	ytfH	putative transcriptional regulator YtfH	0.085217754	5.608384885	0.782325983	0.869579359
b0917	ycaR	UPF0434 family protein YcaR	0.085127431	6.409537901	0.703737806	0.811881876
b2998	yghW	DUF2623 domain-containing protein YghW	0.08512729	7.068066539	0.620581617	0.751266202
b2549	yphG	DUF5107 domain-containing protein YphG	0.085069063	5.492146613	0.741250316	0.838488379
b3141	agaI	putative deaminase AgaI	0.084624048	5.772742779	0.853098909	0.912788886
b3552	yiaD	PF13488 family lipoprotein YiaD	0.084571765	8.226606714	0.624659398	0.754134876
b0605	ahpC	alkyl hydroperoxide reductase, AhpC component	0.08441236	9.229345524	0.603988937	0.737520981
b4249	bdcA	c-di-GMP-binding biofilm dispersal mediator protein	0.084210502	5.342119835	0.773936652	0.864702507
b1654	grxD	glutaredoxin 4	0.083317872	7.638419675	0.561409142	0.701165873
b1838	pphA	phosphoprotein phosphatase 1	0.082864208	6.539242244	0.676882233	0.792478138
b2548	yphF	putative ABC transporter periplasmic binding protein YphF	0.082806332	4.677095571	0.839674717	0.906618703
b3551	bisC	biotin sulfoxide reductase	0.082776625	8.081543538	0.612515506	0.744768922
b2105	rcnR	DNA-binding transcriptional repressor RcnR	0.082648584	5.450658754	0.761137375	0.852898861
b4001	yjaH	DUF1481 domain-containing protein YjaH	0.082422757	9.202145979	0.67342203	0.789893269
b0084	ftsI	peptidoglycan DD-transpeptidase FtsI	0.081423355	9.082660024	0.607090813	0.740416006
b0957	ompA	outer membrane porin A	0.081207322	10.22725354	0.605093631	0.738592309
b2673	nrdH	glutaredoxin-like protein	0.080931669	5.765598765	0.781352892	0.868716449
b2667	ygaV	putative DNA-binding transcriptional regulator YgaV	0.080655796	6.016167319	0.779373188	0.867703583
b3889	yiiE	putative DNA-binding transcriptional regulator YiiE	0.080171576	8.993136863	0.68513365	0.799343916
b2780	pyrG	CTP synthetase	0.080105435	9.853486068	0.657761235	0.7796908
b1613	manA	MANNPISOM-MONOMER	0.079825005	8.530007489	0.627934541	0.756640556
b4484	cpxP	periplasmic protein CpxP	0.079763277	9.387467655	0.662321846	0.782695651
b4022	rluF	23S rRNA pseudouridine2604 and tRNATyr	0.079429776	7.079756894	0.622991903	0.75294532
b3476	nikA	Ni(2+) ABC transporter periplasmic binding protein	0.079349712	6.748102109	0.719697388	0.821474656
b0757	galK	GALACTOKIN-MONOMER	0.079218182	7.605774921	0.583508235	0.71980689
b3580	lyxK	L-xylulose kinase	0.079055564	4.97386608	0.831357226	0.903127722
b3836	tatA	twin arginine protein translocation system - TatA protein	0.078236927	9.602509068	0.658013119	0.7796908
b3854	rrlA	23S ribosomal RNA	0.077646473	11.96456864	0.607889375	0.740836324
b1547	ydfN	Qin prophage; putative side tail fibre assembly protein	0.077101145	4.518056628	0.841264875	0.906724036
b3922	yiiS	DUF406 domain-containing protein YiiS	0.077100495	9.35542077	0.661048658	0.781818865
b3976	thrU	tRNA-Thr(UGU)	0.077037697	9.20525125	0.705071641	0.812055501
b2703	srlE	sorbitol-specific PTS enzyme IIBCsub1sub component	0.076937934	4.516466793	0.885645451	0.934391134
b2929	fumE	fumarase E	0.076884033	7.226452062	0.5881472	0.723911316
b3866	yihI	Der GTPase-activating protein YihI	0.076585369	9.495466292	0.641264165	0.766349056
b3905	rhaS	DNA-binding transcriptional activator RhaS	0.076543059	9.009426053	0.698660112	0.808541126
b3339	tufA	translation elongation factor Tu 1	0.076475545	10.40388533	0.622583527	0.75286184
b4437	sibB	small RNA SibB	0.076226159	5.735360516	0.834924556	0.903964913
b3228	sspB	ClpXP protease specificity-enhancing factor	0.076214331	8.474987549	0.638664128	0.764561314
b2597	raiA	stationary phase translation inhibitor and ribosome stability factor	0.076181223	8.553381108	0.664862864	0.783601053
b2193	narP	Phosphorylated DNA-binding transcriptional dual regulator NarP	0.076170468	7.42965888	0.571850522	0.71098847
b2414	cysK	O-acetylserine sulfhydrylase A	0.075711144	9.964495285	0.640530494	0.766172347
b1410	ynbC	hydrolasemethyltransferase domain-containing protein YnbC	0.075658047	4.118006182	0.849208186	0.911340336
b1383	ydbL	DUF1318 domain-containing protein YdbL	0.075302776	5.886864409	0.708830977	0.813911252
b3465	rsmD	16S rRNA m2G966 methyltransferase	0.075022786	7.499216162	0.624418343	0.754134876

b4259	holC	DNA polymerase III subunit χ	0.074845453	7.000515015	0.688924968	0.801899218
b2496	hda	inibitor of reinitiation of DNA replication	0.074499893	7.717424068	0.622752308	0.75286184
b3364	tsgA	putative transporter TsgA	0.074192736	6.989020125	0.66528691	0.783682419
b1651	gloA	glyoxalase I	0.074163182	6.874789719	0.684365051	0.798892313
b0565	ompT	outer membrane protease VII (outer membrane protein 3b)	0.074109166	9.607922467	0.647360303	0.7712077
b3205	rapZ	RNase adaptor protein	0.073762934	8.903669007	0.645396828	0.769907321
b2067	dgcE	putative diguanylate cyclase DgcE	0.073346646	7.926097626	0.621276907	0.751695682
b1093	fabG	3-oxoacyl-[acyl-carrier-protein] reductase FabG	0.072973823	8.918368211	0.687481209	0.801099932
b1781	yeaE	methylglyoxal reductase YeaE	0.072824594	6.168835291	0.795306024	0.878258153
b1220	ychO	putative invasin YchO	0.072774722	6.954740246	0.658801858	0.78003705
b0548	ninE	DLP12 prophage; NinE family prophage protein	0.072747687	2.100909425	1	1
b0953	rmf	ribosome modulation factor	0.072652837	7.118880224	0.635485089	0.761979039
b2328	mepA	peptidoglycan DD-endopeptidasepeptidoglycan LD-endopeptidase	0.07227719	7.50612718	0.629953435	0.758188604
b2860	insD-4	IS2 insertion element protein InsB	0.071898459	7.47849599	0.593431085	0.728789497
b3516	gadX	DNA-binding transcriptional dual regulator GadX	0.070565524	9.58742617	0.652715328	0.775703411
b0679	nagE	N-acetylglucosamine-specific PTS enzyme IIABC component	0.07014164	7.892769362	0.626548797	0.755795884
b1534	ydeE	dipeptide exporter	0.070016621	5.87167554	0.78266586	0.869738176
b4082	mdtN	putative multidrug efflux pump membrane fusion protein	0.069898785	5.303299345	0.811296669	0.889243991
b2287	nuoB	NADH:quinone oxidoreductase subunit B	0.06971589	8.877645382	0.72521055	0.825776508
b3838	tatB	twin arginine protein translocation system - TatB protein	0.069223664	9.699383013	0.682309321	0.797774272
b2595	bamD	outer membrane protein assembly factor BamD	0.069139196	9.070286006	0.663404211	0.78308656
b3971	rrfB	5S ribosomal RNA	0.068971726	9.421806623	0.713089456	0.816677029
b2898	ygfZ	folate-binding protein	0.068826432	8.562483433	0.688414703	0.801728018
b3266	acrF	multidrug efflux pump RND permease AcrF	0.068488505	7.357576624	0.763433175	0.855254369
b1623	add	adenosine deaminase	0.067073638	7.406369332	0.664356015	0.783421957
b3607	cysE	serine acetyltransferase	0.066743116	8.449627616	0.669019201	0.786025898
b3968	rrsB	16S ribosomal RNA	0.066125186	11.31407742	0.662723055	0.782750746
b4597	rydC	small regulatory RNA RydC	0.065950327	4.004386891	0.889379876	0.936245149
b1706	ydiU	UPF0061 family protein YdiU	0.065697469	8.153283259	0.704436064	0.812055501
b1749	xthA	exodeoxyribonuclease III	0.065557468	7.3429512	0.689481875	0.802252555
b0088	murD	UDP-N-acetylmuramoyl-L-alanine—D-glutamate ligase	0.06554591	8.943543889	0.693388165	0.804760449
b0201	rrsH	16S ribosomal RNA	0.065050068	11.01318816	0.668844602	0.786025898
b3941	metF	5,10-methylenetetrahydrofolate reductase	0.06433095	9.167214888	0.735206511	0.834002247
b1490	dosC	diguanylate cyclase DosC	0.064163326	6.613844308	0.726279153	0.826210374
b2447	yffP	CPZ-55 prophage; uncharacterized protein YffP	0.063933964	4.537482393	0.83136479	0.903127722
b3667	uhpC	UhpC-glucose-6-P	0.063807853	5.69449147	0.788087286	0.87203142
b3454	livF	branched chain amino acidphenylalanine ABC transporter ATP binding s	0.063657696	5.778292571	0.80221147	0.883012625
b0335	prpE	propionyl-CoA synthetase	0.062546485	5.807790735	0.804896407	0.88464467
b4195	ulaC	L-ascorbate specific PTS enzyme IIA component	0.062184503	3.733759086	0.87760589	0.930283314
b1766	sppA	protease IV, a signal peptide peptidase	0.06206525	8.587439812	0.732657487	0.831324288
b0223	yafJ	putative glutamine amidotransferase YafJ	0.061880923	8.179523893	0.668221637	0.786025898
b2180	yejF	putative oligopeptide ABC transporter ATP binding subunit	0.061844488	8.36129543	0.683268293	0.798404222
b2362	yfdS	CPS-53 (KpLE1) prophage; protein YfdS	0.061704304	2.149331801	1	1
b3851	rrsA	16S ribosomal RNA	0.061680309	11.27141118	0.683246733	0.798404222
b3197	kdsD	D-arabinose 5-phosphate isomerase KdsD	0.061604742	8.702196698	0.697409091	0.80751669
b3351	kefG	protein required for KefB activity	0.061542275	6.314364507	0.872677593	0.926839446
b3844	fre	flavin reductase	0.061288238	10.04314101	0.699391963	0.809175973

b2940	yqgC	protein YqgC	0.061128567	6.718012952	0.703937445	0.811881876
b0471	ybaB	putative nucleoid-associated protein YbaB	0.060417448	7.716334179	0.746865967	0.841990383
b2312	purF	amidophosphoribosyltransferase	0.060179118	8.020487195	0.711864811	0.815697675
b2840	ygeA	amino acid racemase	0.059932921	6.434326753	0.785856041	0.871309498
b3963	fabR	DNA-binding transcriptional repressor FabR	0.059644233	9.819712806	0.705675928	0.812131564
b3784	rfe	UDP-N-acetylglucosamine—undecaprenyl-phosphate N	0.059615695	9.887473365	0.717422188	0.819712861
b3816	corA	Ni2+	0.059331394	9.609698846	0.742932148	0.839530442
b2910	zapA	cell division protein ZapA	0.059164927	8.288919165	0.715241511	0.81771444
b3672	ivbL	ilvBN operon leader peptide	0.058408222	6.081871436	0.822910033	0.897534072
b0807	rlmF	23S rRNA m6A1618 methyltransferase	0.058351447	6.144274985	0.840113902	0.906673497
b3953	frwD	putative PTS enzyme IIB component FrwD	0.058331813	9.039541568	0.765425476	0.857051351
b3030	parE	DNA topoisomerase IV subunit B	0.058009805	9.267666633	0.731031079	0.830118883
b3729	glmS	L-glutamine—D-fructose-6-phosphate aminotransferase	0.05792976	10.3201742	0.70580947	0.812131564
b3825	pldB	lysophospholipase L2	0.056450938	9.777729017	0.728279929	0.827846521
b4118	melR	DNA-binding transcriptional dual regulator MelR	0.056331652	6.3917006	0.800657743	0.882182165
b4716	cpxQ	small regulatory RNA CpxQ	0.056045651	9.049800002	0.775892009	0.866134822
b3449	ugpQ	glycerophosphodiester phosphodiesterase	0.055559534	7.829995492	0.723282268	0.825139848
b0090	murG	NACGLCTRANS-MONOMER	0.055337413	8.600743064	0.72620484	0.826210374
b1368	ynaA	Rac prophage; putative prophage tail length tape measure domain-cont	0.055188542	4.009522651	0.943011868	0.971848132
b2674	nrdI	dimanganese-tyrosyl radical cofactor maintenance flavodoxin NrdI	0.054783708	5.712491811	0.845415946	0.909702998
b3377	yhfT	uncharacterized protein YhfT	0.054703546	5.513573232	0.926181933	0.960722162
b4387	ytjB	protein Smp	0.054460712	6.39065233	0.833042966	0.9034658
b3834	ubiJ	ubiquinone biosynthesis accessory factor UbiJ	0.054454149	9.650241829	0.747649961	0.842489683
b0085	murE	UDP-NACMURALGLDAPLIG-MONOMER	0.054400535	9.398315545	0.742637065	0.839530442
b0215	dnaQ	DNA polymerase III subunit ε	0.054378385	7.999044213	0.685281251	0.799343916
b2554	glrR	Pasp56 DNA-binding transcriptional activator GlrR	0.054297614	7.574051632	0.688660938	0.80180328
b4515	cydX	cytochrome bd-I ubiquinol oxidase subunit CydX	0.054272242	6.888766064	0.77165452	0.86271327
b2327	yfcA	conserved inner membrane protein YfcA	0.053979717	7.703865686	0.729480985	0.82899834
b1535	dgcZ	diguanylate cyclase DgcZ	0.053512064	6.307614583	0.779263452	0.867703583
b1581	rspA	mandelate racemasemuconate lactonizing enzyme family protein RspA	0.052738855	4.148068418	0.901838224	0.943734477
b1372	stfR	Rac prophage; putative membrane protein	0.052667185	5.993808857	0.838117508	0.905530346
b3874	yihN	putative transporter YihN	0.052038772	9.045230374	0.791717769	0.875829103
b0952	pqiC	intermembrane transport lipoprotein PqiC	0.051541786	7.23574353	0.701270296	0.810689567
b0071	leuD	3-isopropylmalate dehydratase subunit LeuD	0.051501022	6.043514186	0.837659208	0.905530346
b1713	pheT	phenylalanine—tRNA ligase subunit β	0.05063968	10.06717495	0.765235649	0.857051351
b0396	araJ	putative transport protein AraJ	0.050601493	6.792073081	0.822452866	0.897534072
b4262	lptG	lipopolysaccharide transport system protein LptG	0.050586339	8.235407636	0.729918023	0.829281542
b3271	yhdZ	putative ABC transporter ATP-binding subunit YhdZ	0.050219298	7.265765744	0.835012628	0.903964913
b0321	yahG	DUF1116 domain-containing protein YahG	0.049502212	5.424704764	0.858068237	0.916393629
b0061	araD	L-ribulose-5-phosphate 4-epimerase AraD	0.049487072	3.623496276	1	1
b1685	ydiH	protein YdiH	0.049230653	5.28746804	0.879425292	0.93064781
b3239	yhcO	putative barnase inhibitor	0.049132148	7.643376091	0.779837187	0.867905516
b3890	yiiF	protein YiiF	0.048718843	9.065510513	0.799281534	0.881105611
b1328	pgrR	DNA-binding transcriptional repressor PgrR	0.048517774	6.716957733	0.825636037	0.899823032
b0791	ybhQ	putative inner membrane protein	0.048229103	6.195710879	0.847573514	0.910471305
b2744	umpG	broad specificity 5'(3')-nucleotidase and polyphosphatase	0.048168589	8.445568451	0.79626855	0.879101187
b3142	yraH	putative fimbrial protein YraH	0.04803059	6.078426138	0.885713907	0.934391134

b3550	yiaC	peptidyl-lysine N-acetyltransferase YiaC	0.047931138	6.602226728	0.800254049	0.881957471
b3906	rhaR	DNA-binding transcriptional activator RhaR	0.047496384	9.043890593	0.806491057	0.885484918
b4355	tsr	methyl-accepting chemotaxis protein Tsr	0.047449328	5.992400123	0.84116158	0.906724036
b4147	efp	protein chain elongation factor EF-P, β-lysyl-Lys34	0.047163896	8.921727799	0.783388057	0.870321659
b0760	modF	ABC family protein ModF	0.046884792	7.416864132	0.794752604	0.878086273
b3873	yihM	putative TIM barrel domain-containing protein YihM	0.046042989	9.148280647	0.798137405	0.880283947
b3949	frwC	putative PTS enzyme IIC component FrwC	0.045819433	9.026955565	0.814262349	0.891167147
b2290	alaA	glutamate—pyruvate aminotransferase AlaA	0.045599873	8.894016354	0.792119572	0.876054086
b0290	ecpD	fimbrial adhesin EcpD	0.045591373	7.045173884	0.748721787	0.843267013
b3921	yiiR	DUF805 domain-containing protein YiiR	0.045327501	9.150416254	0.813005158	0.890453429
b3509	hdeB	periplasmic acid stress chaperone HdeB	0.045201543	8.794584976	0.792693316	0.876249623
b0449	mdlB	ABC transporter family protein MdlB	0.044920985	7.308160629	0.76842935	0.859825201
b2078	baeS	sensory histidine kinase BaeS - phosphorylated	0.044715215	6.318422603	0.861389323	0.918829153
b3764	maoP	macrodomain Ori protein	0.044701198	9.749938793	0.803054595	0.883500134
b2896	ygfX	protein YgfX	0.043519595	7.516196068	0.760828327	0.852768993
b3176	glmM	phosphoglucosamine mutase	0.042338658	9.275400961	0.786952926	0.871494332
b0323	yahI	carbamate kinase-like protein YahI	0.042093101	5.30840096	0.890071676	0.936303657
b2043	wcaM	putative colanic acid biosynthesis protein WcaM	0.041984636	5.50125522	0.870891562	0.925833432
b2375	yfdX	protein YfdX	0.041417929	2.644331222	1	1
b2770	ygcR	putative flavoprotein	0.041287087	4.233113602	0.949410834	0.975255661
b3652	recG	ATP-dependent DNA helicase RecG	0.04128697	8.300286146	0.801820387	0.882802247
b0549	ybcO	DLP12 prophage; putative nuclease YbcO	0.04125778	2.013436804	1	1
b4594	ymgJ	uncharacterized protein YmgJ	0.0411024	3.988566794	0.948215912	0.974708556
b3422	rtcR	DNA-binding transcriptional activator RtcR	0.041099788	6.520801406	0.840521058	0.906724036
b2118	yehI	DUF4132 domain-containing protein YehI	0.041000472	6.115640652	0.895596579	0.940770615
b3690	cbrA	colicin M resistance protein	0.040866102	5.419817683	0.940390984	0.97027955
b4703	pmrR	putative bitopic inner membrane protein	0.040290822	6.478430375	0.829496411	0.902248498
b3964	yijD	conserved inner membrane protein YijD	0.039853408	9.555486271	0.811718828	0.889485884
b2512	bamB	outer membrane protein assembly factor BamB	0.039697851	9.113151729	0.810992176	0.88913098
b3986	rplL	50S ribosomal subunit protein L7	0.039570037	9.766577729	0.808296106	0.886835812
b0080	cra	DNA-binding transcriptional dual regulator Cra	0.039487733	8.334214553	0.801751823	0.882802247
b0116	lpd	lipoamide dehydrogenase	0.039385091	10.21852973	0.806663724	0.885484918
b3952	pflC	putative pyruvate formate-lyase 2 activating enzyme PflC	0.038887374	9.054426087	0.838577575	0.905655576
b2220	atoC	phosphorylated DNA-binding transcriptional activator AtoC	0.038749659	5.403537209	0.926558317	0.960722162
b3950	frwB	putative PTS enzyme IIB component FrwB	0.038612528	9.023457409	0.841769257	0.906724036
b3283	yrdD	putative DNA topoisomerase	0.038226613	8.394726977	0.82747232	0.900712597
b0912	ihfB	integration host factor subunit β	0.03801847	7.696299512	0.779077702	0.867703583
b3619	rfaD	ADP-L-glycero-D-mannoheptose 6-epimerase	0.037741046	8.978339712	0.822545202	0.897534072
b3984	rplA	50S ribosomal subunit protein L1	0.037739171	10.30493246	0.802675918	0.883303634
b0653	gltK	glutamateaspartate ABC transporter membrane subunit GltK	0.037724395	7.016039049	0.874895906	0.928748599
b1476	fdnI	formate dehydrogenase N subunit γ	0.03743416	6.113732658	0.887659203	0.93524666
b3164	pnp	polynucleotide phosphorylase	0.037305594	10.5134482	0.828058904	0.901128928
b0327	yahM	uncharacterized protein YahM	0.03709686	5.811339875	0.86794951	0.924040773
b2158	yeiH	conserved inner membrane protein YeiH	0.0368915	6.536250516	0.847539519	0.910471305
b3535	yhjR	PF10945 family protein YhjR	0.036821661	7.423258862	0.77945921	0.867703583
b0177	bamA	outer membrane protein assembly factor BamA	0.036425592	10.47221531	0.831245897	0.903127722
b4690	eyeA	CP4-6 prophage; small RNA EyeA	0.036294918	5.671427417	0.885137938	0.934267857

b2516	rodZ	transmembrane component of cytoskeleton	0.035679563	8.850762943	0.833991179	0.903964913
b2388	glk	glucokinase	0.035442851	8.377917327	0.827160349	0.900656027
b0524	lpxH	EG12666-MONOMER	0.035414138	7.379584123	0.818336971	0.894075162
b3179	rlmE	23S rRNA 2'-O-ribose U2552 methyltransferase	0.035351535	8.660060635	0.835169395	0.903964913
b2716	ascB	6-phospho-β-glucosidase AscB	0.034884592	6.53892966	0.838184913	0.905530346
b4452	gadY	small regulatory RNA GadY	0.034502927	6.655022574	0.841826007	0.906724036
b2555	qseG	outer membrane lipoprotein QseG	0.034378037	7.090706869	0.812526179	0.890149648
b0205	rrfH	5S ribosomal RNA	0.034288858	8.072381311	0.831572708	0.903127722
b3022	mqsR	mRNA interferase toxin of the MqsR-MqsA toxin-antitoxin system	0.034051889	6.165837464	0.889868773	0.936303657
b3884	csqR	DNA-binding transcriptional dual regulator CsqR	0.033760445	9.251520168	0.851780491	0.912326751
b4007	rrsE	16S ribosomal RNA	0.03373952	10.67236728	0.831941211	0.903127722
b0938	elfA	putative laminin-binding fimbrial subunit	0.033362465	5.557133532	0.894094325	0.939639715
b4532	hicA	toxin of the HicA-HicB toxin-antitoxin system	0.033303304	4.644262099	0.956877525	0.980372845
b3872	yihL	putative transcriptional regulator YihL	0.032663838	9.152384559	0.857297937	0.916014131
b1434	sutR	DNA-binding transcriptional dual regulator SutR	0.032656192	5.771710736	0.877060703	0.930283314
b2441	eutB	ethanolamine ammonia-lyase subunit α	0.03230393	8.157116723	0.836152851	0.904585846
b2112	yehE	DUF2574 domain-containing protein YehE	0.032105122	6.032771147	0.887675594	0.93524666
b1460	ydcC	H repeat-associated putative transposase YdcC	0.031956768	4.049784225	0.897019037	0.940921608
b1756	ynjD	putative ABC transporter ATP-binding protein YnjD	0.031276308	5.46182116	0.945309723	0.973307469
b3247	rng	RNase G	0.030896796	9.043558443	0.848094882	0.910588254
b3787	wecC	UDP-N-acetyl-D-mannosamine dehydrogenase	0.030892792	9.857179381	0.852131454	0.912481292
b4533	ynfO	Qin prophage; DUF3950 domain-containing protein YnfO	0.030596805	2.511456161	1	1
b2525	fdx	oxidized ferredoxin	0.030532521	8.159555208	0.85886032	0.91701773
b3702	dnaA	chromosomal replication initiator protein DnaA	0.029544092	9.009810794	0.860137942	0.917937881
b2947	gshB	glutathione synthetase	0.02910638	8.736393038	0.865237052	0.921645591
b3864	spf	small regulatory RNA Spot 42	0.028627734	9.043421666	0.879415616	0.93064781
b3855	rrfA	5S ribosomal RNA	0.02784058	9.322672318	0.877568817	0.930283314
b2343	yfcZ	DUF406 domain-containing protein YfcZ	0.027347935	7.745217704	0.909690337	0.949027136
b2991	hybF	hydrogenase maturation protein HybF	0.027309997	6.326491877	0.896917738	0.940921608
b3278	rrsD	16S ribosomal RNA	0.026956113	10.88956663	0.864320755	0.921288299
b2541	hcaB	2,3-dihydroxy-2,3-dihydrophenylpropionate dehydrogenase	0.026878218	5.174593528	0.944121376	0.972537535
b2789	gudP	galactarateglucarate	0.026630078	4.305635698	0.956615452	0.980372845
b3839	tatC	twin arginine protein translocation system - TatC protein	0.025403374	9.680579759	0.878021309	0.930429396
b2897	sdhE	FAD assembly factor	0.025274539	7.199464642	0.89370089	0.939449864
b1176	minC	Z-ring positioning protein MinC	0.024929029	8.211969844	0.886082232	0.934556391
b1429	tehA	tellurite resistance protein	0.024574736	6.633688726	0.888087926	0.93524666
b3977	tyrU	tRNA-Tyr(GUA)	0.024177957	9.294035115	0.896849125	0.940921608
b3786	wecB	UDP-N-acetylglucosamine 2-epimerase	0.024114133	9.874140482	0.881022842	0.931580855
b3979	thrT	tRNA-Thr(GGU)	0.023898145	9.208742163	0.898533009	0.941888482
b3556	cspA	cold shock protein CspA	0.023041624	8.34580286	0.892676139	0.938596131
b2579	grcA	stress-induced alternate pyruvate formate-lyase subunit	0.022822352	8.799809177	0.896057594	0.940921608
b3715	yieH	6-phosphogluconate phosphatase	0.022651186	6.713994206	0.898658029	0.941888482
b2519	pbpC	peptidoglycan glycosyltransferase PbpC	0.0225859	7.219677173	0.910099208	0.949229389
b1279	lapA	lipopolysaccharide assembly protein A	0.021793651	6.758932354	0.946449692	0.973799905
b3214	gltF	periplasmic protein GltF	0.021693001	6.886855307	0.935450176	0.967259236
b4592	appX	small protein AppX	0.021402833	6.331181597	0.957833727	0.980372845
b0918	kdsB	3-deoxy-manno-octulosonate cytidylyltransferase	0.021018669	8.212221774	0.916789654	0.953958838

b3479	nikD	Ni(2+) ABC transporter ATP binding subunit NikD	0.020743895	6.168586679	0.922589084	0.957506067
b3978	glyT	tRNA-Gly(UCC)	0.020349777	9.204127695	0.912624043	0.950964161
b1996	insD-3	CP4-44 prophage; IS2 insertion element protein InsB	0.020230956	7.033429712	0.904892516	0.945138504
b0607	uspG	universal stress protein G	0.019756212	6.804452727	0.916794417	0.953958838
b3985	rplJ	50S ribosomal subunit protein L10	0.018951897	9.98703481	0.902144327	0.943831091
b0320	yahF	putative acyl-CoA synthetase YahF	0.018808443	5.20174743	0.941345556	0.970810705
b0114	aceE	pyruvate dehydrogenase E1 component	0.018217375	11.08940325	0.910917013	0.949857963
b2917	scpA	methylmalonyl-CoA mutase	0.018097538	5.864233167	0.918505197	0.955513771
b0473	htpG	chaperone protein HtpG	0.016230093	9.602388551	0.930515022	0.964145464
b3663	yicN	DUF1198 domain-containing protein YicN	0.015893782	5.562646109	0.974869422	0.991944802
b2176	pdeN	putative c-di-GMP phosphodiesterase PdeN	0.015559814	7.042559955	0.922384547	0.957506067
b1095	fabF	β-ketoacyl-[acyl carrier protein] synthase II	0.015337772	9.761585685	0.931624867	0.96484255
b0821	ybiU	DUF1479 domain-containing protein YbiU	0.015051365	7.30317427	0.913872393	0.951815668
b3600	mtlD	mannitol-1-phosphate 5-dehydrogenase	0.014496057	8.729631708	0.944346009	0.972542017
b0633	rlpA	rare lipoprotein RlpA	0.014486209	8.557504324	0.948920561	0.975205838
b2933	cmtA	mannitol-specific PTS enzyme IICB component CmtA	0.014053882	4.188755474	1	1
b1714	pheS	phenylalanine—tRNA ligase subunit α	0.013865976	8.769719153	0.955647229	0.980065625
b0139	htrE	putative fimbrial usher protein HtrE	0.013559823	5.544654361	0.95557356	0.980065625
b2297	pta	phosphate acetyltransferase	0.013227626	9.293140865	0.945593266	0.97337241
b0418	pgpA	phosphatidylglycerophosphatase A	0.012813632	7.526547779	0.908749793	0.948269992
b3326	gspE	Type II secretion system protein GspE	0.012756997	5.974607662	0.955257313	0.980065625
b0339	cynT	carbonic anhydrase 1	0.012705632	5.762862246	0.974372555	0.99166778
b2776	ygcE	putative sugar kinase YgcE	0.012489206	5.328878684	0.939567091	0.96965608
b3896	yiiG	DUF3829 domain-containing lipoprotein YiiG	0.012030607	9.065116595	0.943938092	0.972537535
b2323	fabB	β-ketoacyl-[acyl carrier protein] synthase I	0.01183613	9.937999236	0.952060784	0.977113305
b1273	yciN	protein YciN	0.011424274	7.132477503	0.968776376	0.988022107
b4207	fklB	FKBP-type peptidyl-prolyl cis-trans isomerase FklB	0.011340166	7.528773656	0.949353911	0.975255661
b2012	yeeD	putative sulfurtransferase YeeD	0.010932885	8.175014681	0.956156151	0.980132205
b3601	mtlR	transcriptional repressor MtlR	0.010091688	7.782558161	0.950016375	0.975650685
b0233	yafO	ribosome-dependent mRNA interferase toxin YafO	0.009918294	6.382042558	0.933929148	0.967002155
b3969	gltT	tRNA-Glu(UUC)	0.009692381	9.466277619	0.957400887	0.980372845
b1236	galU	UTP—glucose-1-phosphate uridylyltransferase	0.009405612	8.751263042	0.965840009	0.985938414
b3496	dtpB	dipeptidetripeptide:H+	0.009208539	8.392612552	0.957760728	0.980372845
b2697	alaS	alanine—tRNA ligaseDNA-binding transcriptional repressor	0.00918662	10.6815213	0.96023403	0.981826261
b2881	xdhD	fused putative xanthinehypoxanthine oxidase: molybdopterin-binding su	0.00868236	6.461223248	0.981191734	0.996578303
b3199	lptC	lipopolysaccharide transport system protein LptC	0.008641595	8.572635001	0.960254993	0.981826261
b4393	trpR	DNA-binding transcriptional repressor TrpR	0.008241971	6.589732369	1	1
b0051	rsmA	16S rRNA m6sub2	0.008173993	8.609453937	0.972885475	0.990382609
b2260	menE	o-succinylbenzoate—CoA ligase	0.007980244	8.11177055	0.963490005	0.984438277
b1759	nudG	5-hydroxy-CTP diphosphatase	0.007779162	5.255138227	1	1
b3983	rplK	50S ribosomal subunit protein L11	0.006890736	9.882764712	0.963470644	0.984438277
b2472	dapE	succinyl-diaminopimelate desuccinylase	0.006828838	8.396732458	0.984013805	0.999176851
b3928	zapB	cell division factor ZapB	0.006486959	9.707567681	0.96739391	0.987296374
b1209	lolB	outer membrane lipoprotein LolB	0.005358437	7.820867643	1	1
b3981	secE	Sec translocon subunit SecE	0.005182347	9.519709566	0.970649206	0.989703521
b0376	ampH	peptidoglycan DD-carboxypeptidasepeptidoglycan DD-endopeptidase	0.005100064	7.973871982	1	1
b2742	nlpD	murein hydrolase activator NlpD	0.005039183	9.442132041	0.989462397	1

b0185	accA	acetyl-CoA carboxyltransferase subunit α	0.004928514	8.957089829	0.981228966	0.996578303
b0978	appC	cytochrome bd-II ubiquinol oxidase subunit I	0.00469705	9.682703202	0.98689631	1
b2877	mocA	molybdenum cofactor cytidylyltransferase	0.004632582	5.35791646	1	1
b3599	mtlA	mannitol-specific PTS enzyme IICBA component	0.003454294	8.879001914	0.991296534	1
b0584	fepA	ferric enterobactin outer membrane transporter	0.003420542	5.325400495	1	1
b1430	tehB	tellurite methyltransferase	0.003231879	6.771070411	1	1
b2820	recB	exodeoxyribonuclease V subunit RecB	0.002745502	9.346697647	0.993173383	1
b0512	allB	allantoinase	0.002678269	5.368292526	1	1
b1973	zinT	metal-binding protein ZinT	0.002379848	5.653749753	0.976054047	0.99292134
b4226	ppa	inorganic pyrophosphatase	0.002023844	8.803796737	1	1
b1566	flxA	Qin prophage; protein FlxA	0.001962688	3.495962368	1	1
b2064	asmA	putative assembly protein AsmA	0.001911609	8.637629075	0.994090754	1
b1916	sdiA	DNA-binding transcriptional dual regulator SdiA	0.001910679	6.929790043	1	1
b1676	pykF	pyruvate kinase I	0.00177438	9.727322192	1	1
b1266	yciV	RNAssDNA 5'→3' exonuclease	0.001468724	7.171320731	1	1
b3918	cdh	CDPDIGLYPYPHOSPHA-MONOMER	0.001257957	9.252260904	0.988692068	1
b0115	aceF	AceF-dihydrolipoate	0.000881606	10.41664173	1	1
b3290	trkA	NAD-binding component of Trk potassium transporters	0.000612278	8.496490674	1	1
b0533	sfmH	putative fimbrial adhesin protein SfmH	0.00060971	2.512014451	1	1
b3094	exuR	DNA-binding transcriptional repressor ExuR	-0.000601636	8.097971609	1	1
b1114	mfd	transcription-repair coupling factor	-0.000972298	9.533539521	0.991786045	1
b0200	gmhB	D-glycero-β-D-manno	-0.001308558	7.981309159	0.976603834	0.993063132
b4216	ytfJ	protein YtfJ	-0.002232882	5.938937669	1	1
b1274	topA	DNA topoisomerase 1	-0.002260149	9.399171573	0.986009154	1
b2305	rpnB	recombination-promoting nuclease RpnB	-0.002840056	6.104539918	1	1
b1377	ompN	outer membrane porin N	-0.003255514	5.225369975	1	1
b0317	yahC	uncharacterized protein YahC	-0.00355662	5.158681197	1	1
b0623	cspE	transcription antiterminator and regulator of RNA stability	-0.004087165	8.079600703	0.965213377	0.985684606
b3320	rplC	50S ribosomal subunit protein L3	-0.004617994	9.51728242	0.972069865	0.99000887
b3936	rpmE	50S ribosomal subunit protein L31	-0.005051563	9.50072091	0.985984237	1
b0053	surA	peptidyl-prolyl cis-trans isomerase SurA	-0.005330825	9.331529363	0.968528803	0.987997843
b3281	aroE	AROE-MONOMER	-0.00534188	8.782011742	0.971735841	0.99000887
b1208	ispE	4-(cytidine 5'-diphospho)-2-C-methyl-D-erythritol kinase	-0.005394554	8.183004163	0.96256225	0.983957475
b0457	pdeB	c-di-GMP phosphodiesterase PdeB	-0.0054951	8.070351875	0.963701525	0.984438277
b3531	bcsZ	endo-1,4-D-glucanase	-0.005576023	5.743122795	1	1
b1532	marB	multiple antibiotic resistance protein	-0.006210131	6.485399433	0.979621234	0.995632539
b4059	ssb	ssDNA-binding protein	-0.006222471	8.236969889	0.971540625	0.99000887
b2218	rcsC	sensory histidine kinase RcsC - asp875 phosphorylated	-0.006398422	8.538018705	0.972574358	0.990294232
b1471	yddK	leucine-rich repeat domain-containing protein YddK	-0.006817043	2.620672179	1	1
b2013	yeeE	inner membrane protein YeeE	-0.007110839	9.571989247	0.959181039	0.981182643
b2610	ffh	signal recognition particle protein component	-0.007466805	9.219270299	0.958184651	0.980390552
b3334	gspM	Type II secretion system protein GspM	-0.007544235	5.768128088	1	1
b1041	csgB	curlin, minor subunit	-0.007783225	1.914953663	1	1
b4668	ibsB	putative toxic peptide IbsB	-0.00826859	5.375268242	0.976643288	0.993063132
b4382	deoA	thymidine phosphorylase	-0.008546172	8.487069543	0.947186354	0.974028876
b3670	ilvN	acetohydroxy acid synthase I subunit IlvN	-0.009368394	5.712225271	1	1
b3575	yiaK	2,3-diketo-L-gulonate reductase	-0.009470561	4.337399797	1	1

b3611	yibN	putative sulfurtransferase YibN	-0.009590112	7.816023611	0.934619595	0.967259236
b2745	truD	tRNA pseudouridine13 synthase	-0.009729406	8.750899589	0.947083101	0.974028876
b3701	dnaN	β sliding clamp	-0.00986649	8.634701784	0.946399906	0.973799905
b2591	rrsG	16S ribosomal RNA	-0.011130704	10.8709239	0.940850416	0.970526773
b0702	ybfB	uncharacterized protein YbfB	-0.011550457	4.667272562	1	1
b4471	tdcG	L-serine deaminase III	-0.011677682	5.747282355	0.980908053	0.996578303
b4033	malF	maltose ABC transporter membrane subunit MalF	-0.011847571	5.336800565	1	1
b4096	phnL	methylphosphonate degradation complex subunit PhnL	-0.012087707	5.144843274	1	1
b3163	nlpI	lipoprotein NlpI	-0.01223861	9.492017515	0.936186677	0.967397542
b1473	yddG	aromatic amino acid exporter	-0.012656296	4.579395034	1	1
b1413	hrpA	ATP-dependent RNA helicase HrpA	-0.014017712	9.404967529	0.935349174	0.967259236
b3861	yihF	DUF945 domain-containing protein YihF	-0.014098091	8.984954119	0.957562203	0.980372845
b0820	ybiT	ABC transporter ATP-binding protein YbiT	-0.014534247	8.399055155	0.934948432	0.967259236
b3982	nusG	transcription termination factor NusG	-0.014621921	9.698119353	0.936283057	0.967397542
b1912	pgsA	PHOSPHAGLYPSYN-MONOMER	-0.016177511	7.501586425	0.889790944	0.936303657
b0101	yacG	DNA gyrase inhibitor YacG	-0.016532885	6.780778457	0.920866031	0.956617301
b2318	truA	tRNA pseudouridine38-40 synthase	-0.01670477	8.213648788	0.908332317	0.94813158
b3833	ubiE	bifunctional 2-octaprenyl-6-methoxy-1,4-benzoquinone methylase and S	-0.016763575	9.747364775	0.927755372	0.961511729
b0087	mraY	PHOSNACMURPENTATRANS-MONOMER	-0.016854988	8.514355006	0.908402397	0.94813158
b4351	mrr	methylated adenine and cytosine restriction protein	-0.017435708	7.566543682	0.927215475	0.961177816
b0193	yaeF	peptidase C92 family protein YaeF	-0.018085332	6.966716771	0.985445766	1
b3340	fusA	elongation factor G	-0.018570973	11.03405241	0.903922403	0.944969447
b1288	fabI	enoyl-[acyl-carrier-protein] reductase	-0.018758803	9.209396986	0.901288365	0.943606387
b4527	ydaF	Rac prophage; DUF1391 domain-containing protein YdaF	-0.019094657	2.579125182	1	1
b0658	ybeX	CorC-HlyC family protein YbeX	-0.019173859	8.900343446	0.901811534	0.943734477
b0403	malZ	maltodextrin glucosidase	-0.01971432	7.179053285	0.904302585	0.944969447
b3523	yhjE	putative transporter YhjE	-0.020177703	5.463789043	1	1
b2878	ygfK	putative oxidoreductase, Fe-S subunit	-0.020220613	6.004076627	0.955892027	0.980089015
b0522	purK	5-(carboxyamino)imidazole ribonucleotide synthase	-0.020787917	6.336081439	0.920195582	0.956581
b1853	yebK	DNA-binding transcriptional repressor YebK	-0.020802921	7.699854507	0.918875021	0.955673314
b0981	etk	protein-tyrosine kinase Etk	-0.020810589	7.305821096	0.888219535	0.93524666
b1011	rutB	peroxyureidoacrylateureidoacrylate amidohydrolase	-0.020843708	2.270137916	1	1
b2515	ispG	(emEem)-4-hydroxy-3-methylbut-2-enyl-diphosphate synthase (flavodox	-0.021122055	9.005460872	0.892100903	0.938214742
b0687	seqA	SeqA, negative modulator of initiation of replication	-0.021224196	8.286727593	0.881177475	0.931580855
b0333	prpC	2-methylcitrate synthase	-0.021472178	5.121708242	1	1
b3646	yicG	conserved inner membrane protein YicG	-0.021614712	6.446652126	0.90124086	0.943606387
b2522	sseB	protein SseB	-0.0217634	8.150563088	0.878375928	0.930429396
b2122	yehQ	SWIM zinc finger domains-containing protein YehQ	-0.021957989	4.924770592	0.965368117	0.985684606
b0428	cyoE	HEMEOSYN-MONOMER	-0.022219399	9.291787993	0.888100958	0.93524666
b1280	lapB	lipopolysaccharide assembly protein B	-0.02238839	7.82333797	0.885138849	0.934267857
b3252	csrD	regulator of CsrB and CsrC decay	-0.022435441	8.84678447	0.898794151	0.941888482
b1723	pfkB	6-phosphofructokinase II	-0.02244644	7.100702477	0.869693721	0.92545138
b0025	ribF	bifunctional riboflavin kinase FMN adenylyltransferase	-0.022904052	8.595171531	0.88048565	0.931323465
b3013	yqhG	DUF3828 domain-containing protein YqhG	-0.022907594	5.685112019	1	1
b0439	lon	Lon protease	-0.02298628	10.10540863	0.876929926	0.930283314
b3821	pldA	outer membrane phospholipase A	-0.023293937	9.614768786	0.904132632	0.944969447
b1084	rne	ribonuclease E	-0.023451652	10.2581429	0.878889173	0.93064781

b3215	yhcA	putative fimbrial chaperone YhcA	-0.023620731	6.602318756	0.970933739	0.989765056
b0102	zapD	cell division factor ZapD	-0.023854975	7.958816088	0.860126723	0.917937881
b0276	yagJ	CP4-6 prophage; protein YagJ	-0.023912013	7.144733089	0.920614421	0.956581
b1735	chbR	DNA-binding transcriptional dual regulator ChbR	-0.024332016	5.839159176	0.938777276	0.969193898
b4736	yliM	protein YliM	-0.024526705	7.106149645	0.849578165	0.91151582
b1546	tfaQ	Qin prophage; putative tail fiber assembly protein TfaQ	-0.024707897	4.776715993	1	1
b4689	rzoQ	Qin prophage; putative lipoprotein RzoQ	-0.024857291	3.033619883	1	1
b3323	gspA	Type II secretion system protein GspA	-0.02549627	5.09563537	0.938899729	0.969193898
b3674	yidF	uncharacterized protein YidF	-0.025567866	7.251524753	0.8782004	0.930429396
b2963	mltC	membrane-bound lytic murein transglycosylase C	-0.025854218	7.300823961	0.899295317	0.942190039
b0620	dpiA	Pasp DNA-binding transcriptional dual regulator DpiA	-0.02587899	6.259977288	0.941744146	0.970994957
b3317	rplB	50S ribosomal subunit protein L2	-0.025921288	9.794734078	0.870673741	0.925833432
b4115	adiC	arginine:agmatine antiporter	-0.025990551	8.225861416	0.870811422	0.925833432
b2581	yfiF	putative methyltransferase	-0.026204922	8.331890736	0.856092685	0.915390943
b1857	znuA	Zn2+ ABC transporter periplasmic binding protein	-0.026277672	7.435912257	0.850975189	0.912326751
b3502	arsB	arseniteantimonite:H+	-0.026529958	5.748603284	0.977502129	0.993707552
b3280	yrdB	DUF1488 domain-containing protein YrdB	-0.027436047	7.656890169	0.830016725	0.902592079
b0089	ftsW	essential cell division protein FtsW	-0.027499877	8.901835417	0.862646846	0.919948267
b0684	fldA	oxidized flavodoxin 1	-0.027705654	8.430046997	0.846555947	0.910313936
b1423	ydcJ	DUF1338 domain-containing protein YdcJ	-0.027825686	6.112629009	0.913845628	0.951815668
b0446	cof	HMP-PP phosphatase	-0.028589921	6.388340369	0.920439778	0.956581
b1233	ychJ	NTF2-like domain-containing protein YchJ	-0.028796171	7.319872024	0.864774471	0.921549431
b2603	yfiR	DUF4154 domain-containing protein YfiR	-0.028986328	7.446413715	0.903944014	0.944969447
b3917	sbp	sulfatethiosulfate ABC transporter periplasmic binding protein Sbp	-0.029055325	9.330615449	0.882725981	0.932799236
b4414	tff	putative small RNA T44	-0.029098262	7.339506006	0.831880504	0.903127722
b2909	ygfB	UPF0149 family protein YgfB	-0.029160238	8.375792596	0.841540605	0.906724036
b4125	dcuS	sensory histidine kinase DcuS - phosphorylated	-0.029255037	7.154204093	0.914597216	0.952345922
b4374	yjjG	pyrimidine 5'-nucleotidase YjjG	-0.029923028	7.676320102	0.837038446	0.905322082
b0764	modB	molybdate ABC transporter membrane subunit	-0.030001887	7.256347481	0.834842325	0.903964913
b4120	melB	melibiose:H+	-0.030356747	6.71437671	0.919226202	0.955813397
b1801	yeaV	putative transporter YeaV	-0.03039221	3.637669552	1	1
b0490	fetA	putative iron ABC exporter ATP-binding subunit FetA	-0.030437362	7.149995367	0.853044357	0.912788886
b3177	folP	dihydropteroate synthase	-0.030505741	8.024978834	0.823324918	0.897534072
b2310	argT	lysinearginine	-0.030560851	5.767388549	0.935770423	0.967320631
b3973	birA	DNA-binding transcriptional repressorbiotin-[acetyl-CoA-carboxylase] lig	-0.030897646	9.537119814	0.873467664	0.927455445
b0346	mhpR	DNA-binding transcriptional activator MhpR	-0.030965849	6.886326327	0.870343245	0.925833432
b0426	yajQ	nucleotide binding protein	-0.031181284	8.551288605	0.848624048	0.910934883
b2557	purL	FGAMSYN-MONOMER	-0.031639083	8.335283428	0.840626068	0.906724036
b0181	lpxA	UDP-N-acetylglucosamine acyltransferase	-0.03178436	8.843923359	0.838256517	0.905530346
b1215	kdsA	3-deoxy-D-manno-octulosonate 8-phosphate synthase	-0.032132311	8.843688457	0.835595288	0.904204215
b2217	rcsB	Pasp56 RcsB	-0.032603289	8.680715035	0.83268803	0.9034658
b1928	yedD	lipoprotein YedD	-0.032946329	6.714808157	0.845103892	0.909588903
b2906	ubiI	2-octaprenylphenol hydroxylase	-0.033001348	8.432534061	0.826609542	0.900439459
b3549	tag	3-methyl-adenine DNA glycosylase I, constitutive	-0.033049455	7.152143785	0.814001547	0.891102611
b2814	metZ	tRNA-initiator Met(CAU)	-0.033149818	6.521455926	0.894794947	0.940152235
b2574	nadB	L-aspartate oxidase	-0.033277453	6.279026436	0.926379973	0.960722162
b1398	paaK	phenylacetate-CoA ligase	-0.033618382	5.859389564	0.884150057	0.933857058

b3939	metB	O-succinylhomoserine(thiol)-lyase	-0.033703196	9.408044068	0.853245692	0.912788886
b0054	lptD	lipopolysaccharide assembly protein LptD	-0.034324865	10.1668852	0.820131767	0.895592815
b2584	patZ	peptidyl-lysine N-acetyltransferase	-0.035088644	8.086737989	0.806501072	0.885484918
b1839	yebY	DUF2511 domain-containing protein YebY	-0.035539518	7.28291641	0.787014342	0.871494332
b1812	pabB	aminodeoxychorismate synthase subunit 1	-0.035980962	7.193923613	0.801056803	0.882401642
b3620	waaF	ADP-heptose—LPS heptosyltransferase 2	-0.036072707	8.745390972	0.818126774	0.894075162
b2853	ygeI	protein YgeI	-0.036299413	4.96863075	0.968078345	0.987766557
b3753	rbsR	DNA-binding transcriptional dual regulator RbsR	-0.036455726	7.275562267	0.826431431	0.900439459
b0664	glnX	tRNA-Gln(CUG)	-0.036515582	6.405997832	0.841333625	0.906724036
b3442	yhhZ	putative endonuclease YhhZ	-0.036866248	4.757785373	1	1
b3649	rpoZ	RNA polymerase subunit ω	-0.037065781	7.94095653	0.777404537	0.866954017
b3101	yqjF	DoxX family protein	-0.037509128	5.775865668	0.921257599	0.956798941
b3644	yicC	UPF0701 family protein YicC	-0.037570453	8.540146556	0.80705765	0.885697123
b3537	bcsF	putative cellulose biosynthesis protein BcsF	-0.038298366	6.219579147	0.896957888	0.940921608
b3297	rpsK	30S ribosomal subunit protein S11	-0.038575736	8.998660167	0.813564157	0.890844681
b1636	pdxY	pyridoxal kinase 2	-0.038843971	8.350993575	0.786385133	0.871494332
b3832	rmuC	putative recombination limiting protein RmuC	-0.039045766	9.596664538	0.842345631	0.907062429
b4064	ghxP	guaninehypoxanthine transporter GhxP	-0.039522407	6.948762972	0.846808456	0.910313936
b0188	tilS	G6096-MONOMER	-0.039856922	8.156121865	0.787207111	0.871494332
b0294	ecpR	DNA-binding transcriptional dual regulator MatA	-0.0404179	5.119560281	0.971993863	0.99000887
b0179	lpxD	UDP-3-O-(3-hydroxymyristoyl)glucosamine N	-0.040693753	9.668366478	0.798543436	0.880511805
b3684	yidP	putative DNA-binding transcriptional regulator YidP	-0.040758456	5.242919965	0.904837972	0.945138504
b0250	ykfB	CP4-6 prophage; protein YkfB	-0.040830863	6.170809615	0.856891164	0.915801135
b0203	alaV	tRNA-Ala(UGC)	-0.041002721	8.135065217	0.861334682	0.918829153
b3299	rpmJ	50S ribosomal subunit protein L36	-0.041476147	7.933076202	0.753890728	0.847575137
b1174	minE	Z-ring positioning protein MinE	-0.041525859	7.067446068	0.777555622	0.866954017
b4163	glyV	tRNA-Gly(GCC)	-0.041620672	7.007547882	0.805212778	0.884772129
b0369	hemB	porphobilinogen synthase	-0.041671892	8.485850885	0.778525188	0.867703583
b0699	ybfA	DUF2517 domain-containing protein YbfA	-0.041876019	7.19687717	0.785087039	0.871113163
b0014	dnaK	chaperone protein DnaK	-0.042368319	10.66649463	0.785348713	0.871184565
b0472	recR	DNA repair protein RecR	-0.043719295	7.889332677	0.751490259	0.845306877
b1788	yoaI	protein YoaI	-0.04431466	4.646046591	0.916470759	0.953958838
b1811	yoaH	UFP0181 family protein YoaH	-0.044378435	6.443437966	0.871615109	0.926156608
b2535	csiE	stationary phase inducible protein CsiE	-0.044777607	7.245867083	0.803686952	0.883759107
b4183	yjfK	DUF2491 domain-containing protein YjfK	-0.045568241	3.347021188	1	1
b2928	yggC	P-loop NTPase domain-containing protein YggC	-0.045591106	7.194258001	0.803690331	0.883759107
b3732	atpD	ATP synthase Fsub1sub complex subunit β	-0.0456101	10.0966204	0.772511347	0.863452556
b0732	mngB	α-mannosidase	-0.04568284	6.991286291	0.797034263	0.879506691
b3375	frlR	putative DNA-binding transcriptional regulator FrlR	-0.046202704	6.689495977	0.84492797	0.909588903
b2979	glcD	glycolate dehydrogenase, putative FAD-linked subunit	-0.046697081	4.792415266	0.924437813	0.959199282
b0152	fhuD	iron(III) hydroxamate ABC transporter periplasmic binding protein	-0.046701401	6.13700858	0.875241927	0.928892574
b0441	ppiD	periplasmic folding chaperone	-0.046772961	9.440113854	0.757546375	0.850602046
b3345	tusD	sulfurtransferase complex subunit TusD	-0.047006204	7.698628417	0.714672823	0.81767738
b2627	abpB	CP4-57 prophage; putative helicase YfjK	-0.048002102	8.303669611	0.743691379	0.839667487
b3282	tsaC	threonylcarbamoyl-AMP synthase	-0.049642569	8.38591855	0.738972389	0.83655464
b3296	rpsD	30S ribosomal subunit protein S4	-0.049775234	9.51928013	0.760523713	0.85264403
b4138	dcuA	C4-dicarboxylate transporter DcuA	-0.050102298	8.132230908	0.75008946	0.844287997

b1322	ycjF	conserved inner membrane protein YcjF	-0.050526373	6.819815368	0.756106177	0.849633182
b3318	rplW	50S ribosomal subunit protein L23	-0.051209535	8.609275933	0.756634638	0.849794436
b3269	yhdX	putative ABC transporter membrane subunit YhdX	-0.051412872	6.975243682	0.885173827	0.934267857
b2895	fldB	oxidized flavodoxin 2	-0.051431609	7.993402407	0.700140855	0.809618092
b1092	fabD	[acyl-carrier-protein] S-malonyltransferase	-0.051444053	9.069145927	0.755655657	0.849343108
b1332	ynaJ	DUF2534 domain-containing protein YnaJ	-0.051827185	7.308964757	0.69510644	0.806410277
b3578	yiaN	2,3-diketo-L-gulonate:Na+ symporter - membrane subunit	-0.051882298	4.414026982	0.95794529	0.980372845
b4590	ybfK	uncharacterized protein YbfK	-0.052188533	1.758620389	1	1
b3381	yhfX	putative PLP-binding protein YhfX	-0.052347543	4.790405607	0.898473046	0.941888482
b4609	ryfD	small regulatory RNA RyfD	-0.052493815	7.342450008	0.74396299	0.839667487
b0528	ybcJ	putative RNA-binding protein YbcJ	-0.052779299	6.056252533	0.846671162	0.910313936
b0692	potE	putrescine:H+ symporter	-0.053267809	5.88250319	0.851723492	0.912326751
b3458	livK	L-leucineL-phenylalanine ABC transporter periplasmic binding protein	-0.05364286	6.21065653	0.828683153	0.901586033
b3223	nanE	putative N-acetylmannosamine-6-phosphate epimerase	-0.053897727	6.826487072	0.865282214	0.921645591
b1197	treA	periplasmic trehalase	-0.053952634	7.083770164	0.738269896	0.835973735
b0271	yagH	CP4-6 prophage; putative xylosidasearabinosidase	-0.054392957	6.010559877	0.877353064	0.930283314
b1563	relE	Qin prophage; mRNA interferase toxin RelE	-0.054475647	7.088949783	0.714762659	0.81767738
b3321	rpsJ	30S ribosomal subunit protein S10	-0.054496372	8.709577469	0.744004351	0.839667487
b0430	cyoC	cytochrome bosub3	-0.055205857	8.998668807	0.746126364	0.841515338
b2695	serV	tRNA-Ser(GCU)	-0.05538276	7.421540038	0.706182942	0.812316446
b0277	yagK	CP4-6 prophage; uncharacterized protein YagK	-0.056023701	6.267592609	0.911489916	0.950006605
b0478	ybaL	putative transporter YbaL	-0.056551507	7.706122312	0.708260769	0.813468078
b3287	def	peptide deformylase	-0.056565613	8.618931865	0.711625562	0.815635217
b3243	aaeR	LysR-type transcriptional regulator AaeR	-0.056569694	7.749444822	0.777597416	0.866954017
b2389	yfeO	putative transport protein YfeO	-0.056624499	7.909200482	0.702247795	0.811204609
b3295	rpoA	RNA polymerase subunit α	-0.056944741	10.05799304	0.723951171	0.825476348
b0027	lspA	lipoprotein signal peptidase	-0.057223214	7.770721473	0.676084155	0.791753726
b4113	basR	Phosphorylated DNA-binding transcriptional dual regulator BasR	-0.057266283	6.902676197	0.768487031	0.859825201
b1582	ynfA	conserved inner membrane protein YnfA	-0.057701841	5.184479754	0.851115969	0.912326751
b1262	trpC	fused indole-3-glycerol phosphate synthasephosphoribosylanthranilate i	-0.058168546	6.258746401	0.821410246	0.896767121
b1154	ycfK	e14 prophage; protein StfP	-0.058551118	3.006530538	1	1
b1111	comR	DNA-binding transcriptional repressor ComR	-0.058588428	6.681633195	0.72447985	0.825652695
b2197	ccmE	periplasmic heme chaperone	-0.058916229	5.936622435	0.81087421	0.88913098
b2760	casA	type I-E CRISPR system Cascade subunit CasA	-0.05901044	5.159230198	0.848073463	0.910588254
b3232	zapE	cell division protein ZapE	-0.059426389	7.771854857	0.727050444	0.826665454
b1830	prc	tail-specific protease	-0.05943064	9.923941603	0.703628112	0.811881876
b0096	lpxC	UDPACYLGLCNACDEACETYL-MONOMER	-0.059677888	9.397160439	0.704855318	0.812055501
b1181	ycgN	PF03693 family protein YcgN	-0.059738038	6.415119054	0.79776798	0.88009636
b2538	hcaE	putative 3-phenylpropionatecinnamate dioxygenase subunit α	-0.059828351	6.717113165	0.77518524	0.865564703
b2617	bamE	outer membrane protein assembly factor BamE	-0.06022275	8.106399582	0.681807657	0.797398889
b3270	yhdY	putative ABC transporter membrane subunit YhdY	-0.060546467	6.829112883	0.871145842	0.925880811
b1764	selD	selenide, water dikinase	-0.060550678	9.001176144	0.704910551	0.812055501
b0126	can	carbonic anhydrase 2	-0.060585305	8.999604818	0.70346868	0.811881876
b1222	narX	sensory histidine kinase NarX	-0.06070936	7.850770033	0.63288601	0.760738289
b2907	ubiH	OCTAPRENYL-METHOXYPHENOL-OH-MONOMER	-0.061303424	8.795084072	0.696050782	0.807002155
b2811	csdE	sulfur acceptor protein CsdE	-0.061308345	7.367889905	0.66478373	0.783601053
b4728	yadW	protein YadW	-0.06213222	6.842604585	0.758219913	0.850738488

b3637	rpmB	50S ribosomal subunit protein L28	-0.062249439	8.342977174	0.678458555	0.793920313
b3303	rpsE	30S ribosomal subunit protein S5	-0.062489785	9.207834622	0.709183908	0.814104772
b2908	pepP	proline aminopeptidase P II	-0.062604423	9.299251866	0.689812415	0.802297633
b0836	bssR	regulator of biofilm formation	-0.062630022	7.050031902	0.719011841	0.82111673
b3130	yhaV	ribosome-dependent mRNA interferase toxin YhaV	-0.063091894	7.628360975	0.739746003	0.83700118
b3309	rplX	50S ribosomal subunit protein L24	-0.063704846	8.632285609	0.705191572	0.812055501
b2341	fadJ	3-hydroxyacyl-CoA dehydrogenase FadJ	-0.063973061	6.965458706	0.703127245	0.811881876
b3241	aaeA	aromatic carboxylic acid efflux pump membrane fusion protein	-0.064115345	7.505746497	0.781179444	0.868716449
b0023	rpsT	30S ribosomal subunit protein S20	-0.064341017	8.333799009	0.67477352	0.790777488
b3988	rpoC	RNA polymerase subunit β'	-0.064521463	11.75790089	0.64638194	0.770843375
b3313	rplP	50S ribosomal subunit protein L16	-0.064919102	8.931216474	0.703934422	0.811881876
b3178	ftsH	ATP-dependent zinc metalloprotease FtsH	-0.064940502	10.17429674	0.662021924	0.782550681
b0887	cydD	glutathioneL-cysteine ABC exporter subunit CydD	-0.065042278	8.103205914	0.663624306	0.78308656
b0202	ileV	tRNA-Ile(GAU)	-0.065073598	8.073879615	0.75118781	0.845182003
b3308	rplE	50S ribosomal subunit protein L5	-0.065209613	9.303161151	0.701434688	0.810689567
b4031	xylE	D-xylose:H+ symporter	-0.065380709	5.406238968	0.879875046	0.93090063
b3093	exuT	hexuronate transporter	-0.065842836	5.863305854	0.868486403	0.924389458
b2237	inaA	putative lipopolysaccharide kinase InaA	-0.06592484	7.599967362	0.615191913	0.746431608
b3319	rplD	50S ribosomal subunit protein L4	-0.06608589	9.402224601	0.692851461	0.804350822
b4166	queG	epoxyqueuosine reductase	-0.066295302	8.402250297	0.668926438	0.786025898
b1379	hslJ	lipoprotein implicated in Novobiocin resistance	-0.066377733	7.760845803	0.672482459	0.789000813
b4389	radA	DNA recombination protein	-0.066557991	7.909198902	0.665263928	0.783682419
b1058	yceO	DUF2770 domain-containing protein YceO	-0.066928845	2.755207228	1	1
b3346	yheO	putative DNA-binding transcriptional regulator YheO	-0.06705259	8.509532279	0.668369134	0.786025898
b0943	ycbV	putative fimbrial protein YcbV	-0.067331222	4.347871239	0.951953565	0.977113305
b0638	cobC	putative adenosylcobalamin phosphataseα-ribazole phosphatase	-0.067678476	6.790209714	0.698286283	0.808320383
b2507	guaA	GMP synthetase	-0.067770305	10.00533698	0.671692931	0.788377053
b0343	lacY	lactose permease	-0.068106235	5.304066759	0.904172223	0.944969447
b0578	nfsB	NAD(P)H nitroreductase NfsB	-0.068258212	7.905079176	0.617849665	0.748985797
b3574	yiaJ	DNA-binding transcriptional repressor YiaJ	-0.068509154	7.193897396	0.725016612	0.825776508
b1382	ynbE	lipoprotein YnbE	-0.068762934	5.372799746	0.815503384	0.891862135
b3538	bcsG	cellulose phosphoethanolamine transferase	-0.06879087	7.855780351	0.683390612	0.798404222
b1949	fliQ	flagellar biosynthesis protein FliQ	-0.069352358	3.729786968	0.937170177	0.968029961
b1719	thrS	threonine—tRNA ligase	-0.069549951	10.37630924	0.662020266	0.782550681
b3310	rplN	50S ribosomal subunit protein L14	-0.069674313	8.792490564	0.690187146	0.802522057
b2086	yegS	lipid kinase	-0.070082007	7.454114347	0.621261677	0.751695682
b0050	apaG	DUF525 domain-containing protein ApaG	-0.070308176	7.441383105	0.648955678	0.772482966
b4164	glyX	tRNA-Gly(GCC)	-0.070852038	6.965068152	0.657919304	0.7796908
b1825	yebO	uncharacterized protein YebO	-0.071428012	8.413085995	0.64137254	0.766349056
b4417	rybB	small regulatory RNA RybB	-0.071670476	4.093860153	0.947334087	0.974028876
b3871	bipA	ribosome-dependent GTPase, ribosome assembly factor	-0.071749222	10.73377549	0.616428667	0.747673782
b3161	mtr	tryptophan:H+ symporter Mtr	-0.071922983	7.154866859	0.69717387	0.807456096
b4610	ygdT	hypothetical protein YgdT	-0.072201664	4.670153205	0.937333654	0.968029961
b3324	gspC	Type II secretion system protein GspC	-0.072301404	5.994644312	0.814750296	0.891444195
b1789	yeaL	conserved inner membrane protein YeaL	-0.072440076	5.539072807	0.793385107	0.876794805
b0979	appB	cytochrome bd-II ubiquinol oxidase subunit II	-0.072752129	9.211686511	0.649381301	0.772781252
b1930	yedF	putative sulfurtransferase YedF	-0.073670077	5.595211178	0.785851793	0.871309498

b4725	rseD	rpoE leader peptide	-0.073835664	7.353750533	0.633371686	0.760907753
b2816	metV	tRNA-initiator Met(CAU)	-0.074173182	6.041854603	0.817539568	0.893867557
b2950	yggR	Type IIIV secretion system family protein	-0.07426454	4.964234687	0.864202256	0.921288299
b3461	rpoH	RNA polymerase, sigma 32 (sigma H) factor	-0.074323672	9.032483663	0.630058113	0.758188604
b2288	nuoA	NADH:quinone oxidoreductase subunit A	-0.075105626	8.347595001	0.642231358	0.767167599
b1617	uidA	β-glucuronidase	-0.075294474	4.919753937	0.851618494	0.912326751
b3778	rep	ATP-dependent DNA helicase Rep	-0.075344867	9.72745701	0.6840075	0.798702225
b3733	atpG	ATP synthase Fsub1sub complex subunit γ	-0.075551372	9.44119069	0.63018128	0.758188604
b2823	ppdC	prepilin-type N-terminal cleavagemethylation domain-containing protei	-0.075627715	5.25422048	0.846278397	0.910313936
b3298	rpsM	30S ribosomal subunit protein S13	-0.076477022	8.867963147	0.65981519	0.780778361
b3116	tdcC	threonineserine:H+	-0.077091115	5.464648018	0.856450065	0.915551341
b3192	mlaC	intermembrane phospholipid transport system - periplasmic binding prc	-0.077136894	8.567346072	0.612892644	0.745022309
b0057	yabQ	protein YabQ	-0.077350503	5.206668393	0.837660111	0.905530346
b1308	pspE	thiosulfate sulfurtransferase PspE	-0.077922144	6.516304966	0.674121565	0.790503775
b3306	rpsH	30S ribosomal subunit protein S8	-0.077941506	8.885533718	0.65089045	0.774159842
b2363	yfdT	CPS-53 (KpLE1) prophage; protein YfdT	-0.078478048	3.961730699	0.952104516	0.977113305
b3737	atpE	ATP synthase Fsubosub complex subunit c	-0.07891358	8.123184524	0.583209797	0.71980689
b3305	rplF	50S ribosomal subunit protein L6	-0.079308522	9.252081029	0.647243901	0.7712077
b2513	yfgM	ancillary SecYEG translocon subunit	-0.07976108	8.348112193	0.612457891	0.744768922
b0282	yagP	putative LysR family substrate binding domain-containing protein YagP	-0.080071457	5.77612332	0.832666188	0.9034658
b4129	lysU	lysine—tRNA ligaseApsub4	-0.080672545	10.07197961	0.603640058	0.737520981
b4028	yjbG	capsule biosynthesis GfcC family protein YjbG	-0.080907372	4.98824755	0.911479301	0.950006605
b4182	yjfJ	PspA family protein YjfJ	-0.081042076	2.858042647	0.896779849	0.940921608
b4546	ypeB	PF12843 family protein YpeB	-0.081367038	6.677778644	0.68359856	0.798435885
b3300	secY	Sec translocon subunit SecY	-0.081405878	10.42581723	0.609230002	0.741795494
b2598	pheL	phe operon leader peptide	-0.08148142	6.780666838	0.777365325	0.866954017
b2746	ispF	2-C-methyl-D-erythritol 2,4-cyclodiphosphate synthase	-0.081841276	7.718163658	0.546972531	0.688786003
b0190	yaeQ	uncharacterized protein YaeQ	-0.081846002	7.609875875	0.568288228	0.708554794
b0466	ybaM	DUF2496 domain-containing protein YbaM	-0.082116152	5.7089172	0.74356948	0.839667487
b1716	rplT	50S ribosomal subunit protein L20	-0.082157086	8.659714112	0.622560297	0.75286184
b0464	acrR	DNA-binding transcriptional repressor AcrR	-0.082161816	7.44661472	0.6411839	0.766349056
b0303	rclB	DUF1471 domain-containing protein RclB	-0.082204821	5.016300461	0.883421552	0.933310877
b3736	atpF	ATP synthase Fsubosub complex subunit b	-0.082763254	8.808687642	0.61208901	0.744660507
b4296	yjhF	KpLE2 phage-like element; putative transporter YjhF	-0.082796366	5.487784712	0.77760768	0.866954017
b1717	rpmI	50S ribosomal subunit protein L35	-0.083262555	8.07578894	0.575665415	0.714323443
b3466	yhhL	DUF1145 domain-containing protein YhhL	-0.083883785	6.300530551	0.736761223	0.834693559
b2605	yfiB	lipoprotein YfiB	-0.084326907	6.959294284	0.70763972	0.812966267
b0675	umpH	UMP phosphatase	-0.084368274	7.641549039	0.57607093	0.714625781
b3314	rpsC	30S ribosomal subunit protein S3	-0.084431072	9.578463586	0.614710197	0.746431608
b1096	pabC	aminodeoxychorismate lyase	-0.085101838	6.945701365	0.63550538	0.761979039
b3388	damX	cell division protein DamX	-0.08537267	9.581827097	0.594188811	0.729314317
b2424	cysU	sulfatethiosulfate ABC transporter inner membrane subunit CysU	-0.085488417	8.599862537	0.610502377	0.742934949
b4413	sokC	antisense RNA blocking mokC and hokC translation	-0.085531916	3.708620334	0.935491945	0.967259236
b0338	cynR	DNA-binding transcriptional dual regulator CynR	-0.086761636	6.179097062	0.788039965	0.87203142
b1101	ptsG	EIIBCglc - phosphorylated	-0.086895873	9.428626494	0.603277757	0.737520981
b4409	blr	β-lactam resistance protein	-0.087342086	6.638825816	0.660111258	0.780919401
b3311	rpsQ	30S ribosomal subunit protein S17	-0.087679618	8.325081955	0.577051202	0.71543978

b3301	rplO	50S ribosomal subunit protein L15	-0.088381829	9.035363731	0.59691958	0.731868018
b3943	yijE	cystine exporter	-0.088462519	9.341560391	0.637859414	0.764087067
b1855	lpxM	myristoyl-acyl carrier protein-dependent acyltransferase	-0.088646042	7.991559124	0.531966111	0.678794907
b0119	yacL	UPF0231 family protein YacL	-0.089596377	7.630054445	0.501457386	0.652500548
b0467	priC	primosomal replication protein N''	-0.090083639	6.521294147	0.624566257	0.754134876
b2244	rpnE	inactive recombination-promoting nuclease-like protein RpnE	-0.090100847	5.499355439	0.752112947	0.845791813
b3294	rplQ	50S ribosomal subunit protein L17	-0.090156034	8.630743987	0.584384043	0.720484654
b0924	mukB	chromosome partitioning protein MukB	-0.091108987	10.37732079	0.552689117	0.693340678
b1901	araF	arabinose ABC transporter periplasmic binding protein	-0.091200467	3.881839185	0.887153267	0.935239177
b3706	mnmE	5-carboxymethylaminomethyluridine-tRNA synthase GTPase subunit	-0.091342359	8.399185391	0.596622596	0.731868018
b3533	bcsA	cellulose synthase catalytic subunit	-0.091581803	6.900982442	0.649857705	0.773139792
b3082	higA	antitoxinDNA-binding transcriptional repressor HigA	-0.091722227	5.678411407	0.834246107	0.903964913
b3292	zntR	DNA-binding transcriptional activator ZntR	-0.091876914	7.146552837	0.54234106	0.685422317
b2719	hycG	formate hydrogenlyase subunit HycG	-0.091970159	4.907771163	0.827216351	0.900656027
b0159	mtn	5'-methylthioadenosineS-adenosylhomocysteine nucleosidase	-0.092281222	8.025342264	0.492913808	0.645186617
b0641	lptE	lipopolysaccharide assembly protein LptE	-0.092604402	8.412210593	0.538611464	0.682726936
b0680	glnS	glutamine—tRNA ligase	-0.093216934	9.792822351	0.55020928	0.691481346
b1738	chbB	N,N'-diacetylchitobiose-specific PTS enzyme IIB component	-0.093483294	5.979187418	0.71529177	0.81771444
b4194	ulaB	L-ascorbate specific PTS enzyme IIB component	-0.093953561	3.522931425	0.931406169	0.964842383
b1193	emtA	lytic murein transglycosylase E	-0.093966187	6.972434232	0.59371525	0.728935714
b1659	ydhB	putative LysR family DNA-binding transcriptional regulator YdhB	-0.094822133	6.449554094	0.654489782	0.777184613
b2231	gyrA	DNA gyrase subunit A	-0.095514283	10.25513528	0.552144097	0.693123738
b2514	hisS	histidine—tRNA ligase	-0.095832583	9.412614929	0.559586141	0.699482676
b3148	yraN	UPF0102 family protein YraN	-0.096164658	7.369768774	0.573073012	0.711906963
b3731	atpC	ATP synthase Fsub1sub complex subunit ε	-0.096345487	8.520670021	0.533537375	0.680013716
b2594	rluD	23S rRNA pseudouridine19111915	-0.097084983	8.361135283	0.523558696	0.671170628
b2264	menD	2-succinyl-5-enolpyruvyl-6-hydroxy-3-cyclohexene-1-carboxylate synthas	-0.097196512	8.945026931	0.547134704	0.68879376
b1053	mdtG	efflux pump MdtG	-0.097234828	6.724464698	0.580018212	0.717104566
b3526	kdgK	2-dehydro-3-deoxygluconokinase	-0.097294194	7.630620185	0.489467943	0.642199397
b1718	infC	translation initiation factor IF-3	-0.097469242	9.102823479	0.564167863	0.704212924
b0763	modA	molybdate ABC transporter periplasmic binding protein	-0.097815772	8.837077906	0.537332828	0.68246396
b3734	atpA	ATP synthase Fsub1sub complex subunit α	-0.098256607	10.31902713	0.546927512	0.688786003
b2152	yeiB	DUF418 domain-containing protein YeiB	-0.098277155	7.139281262	0.579645168	0.716889938
b2793	syd	SecY-interacting protein	-0.09849518	6.746220816	0.632148258	0.760058431
b0852	rimK	ribosomal protein S6 modification protein	-0.099570003	7.678478795	0.448659813	0.606537289
b4356	lgoT	YJIZ-MONOMER	-0.099926731	6.121028894	0.68050859	0.796090467
b3508	yhiD	inner membrane protein YhiD	-0.0999848	8.870640117	0.523166693	0.671170628
b3168	infB	translation initiation factor IF-2	-0.1004956	10.74518094	0.531912774	0.678794907
b2813	mltA	membrane-bound lytic murein transglycosylase A	-0.100553075	7.613529346	0.531158812	0.678352952
b2411	ligA	DNA ligase	-0.100828604	9.164273417	0.560329444	0.700015421
b0654	gltJ	glutamateaspartate ABC transporter membrane subunit GltJ	-0.101238259	7.179162841	0.554583619	0.694917708
b0928	aspC	aspartate aminotransferase	-0.10143024	8.969207762	0.535047609	0.681120599
b2364	dsdC	DNA-binding transcriptional dual regulator DsdC	-0.101565466	5.392722891	0.74268043	0.839530442
b0672	leuW	tRNA-Leu(UAG)	-0.101785592	7.43672606	0.460505297	0.617222491
b3315	rplV	50S ribosomal subunit protein L22	-0.102018607	8.676586939	0.562466867	0.70228824
b1089	rpmF	50S ribosomal subunit protein L32	-0.102267489	7.873730564	0.472194868	0.627555792
b0431	cyoB	cytochrome bosub3	-0.102375735	10.59537644	0.527491066	0.674840062

b2354	yfdK	CPS-53 (KpLE1) prophage; putative tail fiber assembly protein YfdK	-0.102564527	4.570990796	0.823332501	0.897534072
b0151	fhuC	iron(III) hydroxamate ABC transporter ATP binding subunit	-0.102688874	6.240447994	0.652451432	0.775598565
b2265	menF	isochorismate synthase MenF	-0.102939475	8.856349791	0.537469352	0.68246396
b3553	ghrB	glyoxylate reductase	-0.103163017	8.073043068	0.469687128	0.624598997
b3966	btuB	cobalamincobinamide outer membrane transporter	-0.103549576	9.807396731	0.522501987	0.670789844
b4170	mutL	DNA mismatch repair protein MutL	-0.103860384	8.903051635	0.520456942	0.669332188
b1624	ydgJ	putative oxidoreductase YdgJ	-0.103901089	8.058165297	0.510309504	0.66070864
b3457	livH	branched chain amino acidphenylalanine ABC transporter membrane su	-0.104728991	5.016540562	0.758411918	0.850738488
b3307	rpsN	30S ribosomal subunit protein S14	-0.10474489	8.66135003	0.552788038	0.693340678
b3602	yibL	DUF2810 domain-containing protein YibL	-0.104803921	7.065084599	0.544317339	0.687074376
b1860	ruvB	Holliday junction branch migration complex subunit RuvB	-0.105560296	7.679445289	0.434177148	0.593461229
b2076	mdtC	multidrug efflux pump RND permease subunit MdtC	-0.105699186	7.100041162	0.549986871	0.691481346
b1829	htpX	zinc dependent endoprotease	-0.106026227	8.453803995	0.488089443	0.640962193
b2699	recA	DNA recombinationrepair protein RecA	-0.106232254	9.236532754	0.502086058	0.65293371
b2688	gshA	glutamate—cysteine ligase	-0.106373169	9.465629836	0.509483998	0.660026952
b2607	trmD	tRNA m1G37 methyltransferase	-0.106381538	9.637167169	0.518150603	0.667727645
b3387	dam	DNA adenine methyltransferase	-0.106424285	8.296606791	0.476397931	0.63013175
b2865	ygeR	LysM domain-containing putative peptidase lipoprotein YgeR	-0.106444331	7.712047412	0.460490067	0.617222491
b0429	cyoD	cytochrome bosub3	-0.106642317	8.293764328	0.507279598	0.657943428
b1827	kdgR	DNA-binding transcriptional repressor KdgR	-0.106897715	8.098123995	0.443419739	0.602005556
b3813	uvrD	ssDNA translocase and dsDNA helicase - DNA helicase II	-0.10719448	10.23514079	0.484869315	0.638252243
b4395	ytjC	putative phosphatase	-0.107663915	7.195952621	0.464536905	0.620801185
b3220	yhcG	DUF1016 domain-containing protein YhcG	-0.108387666	7.230769162	0.710473101	0.815160795
b2159	nfo	endonuclease IV	-0.108420585	7.445445616	0.468456937	0.623845643
b3302	rpmD	50S ribosomal subunit protein L30	-0.109119341	8.070033143	0.466787548	0.622618436
b2604	dgcN	diguanylate cyclase DgcN	-0.10925562	7.547068676	0.529247142	0.676731564
b2435	amiA	N-acetylmuramoyl-L-alanine amidase A	-0.109923901	7.531023544	0.459621949	0.61641279
b3668	uhpB	sensory histidine kinase UhpB - phosphorylated	-0.110067613	6.765655008	0.554674972	0.694917708
b0176	rseP	intramembrane zinc metalloprotease	-0.110386113	9.17490592	0.518473169	0.667948363
b3989	yjaZ	protein YjaZ	-0.110454753	9.336917199	0.606951632	0.740416006
b0281	intF	CP4-6 prophage; putative phage integrase	-0.110493996	8.241538879	0.476702162	0.63013175
b3402	yhgE	putative transport protein YhgE	-0.110614802	7.053885232	0.546895253	0.688786003
b4613	dinQ	UV inducible membrane toxin DinQ	-0.110749651	6.457357691	0.587304282	0.7232771
b3895	fdhD	sulfurtransferase for molybdenum cofactor sulfuration	-0.111013899	9.538448352	0.516825048	0.666871575
b3940	metL	fused aspartate kinasehomoserine dehydrogenase 2	-0.111221453	9.914783318	0.472811834	0.627808796
b0673	metT	tRNA-Met(CAU)	-0.111288721	6.791620071	0.543312799	0.686134441
b4043	lexA	DNA-binding transcriptional repressor LexA	-0.111965069	7.997475826	0.4120287	0.571148104
b4041	plsB	glycerol-3-phosphate 1-O-acyltransferase	-0.112069165	9.508583819	0.494042943	0.646089926
b0104	guaC	GMP reductase	-0.112196989	7.906531107	0.428824046	0.588151029
b3543	dppB	dipeptide ABC transporter membrane subunit DppB	-0.112889908	5.783064223	0.695357628	0.806410277
b2153	folE	GTP cyclohydrolase 1	-0.113313959	8.229258766	0.431491979	0.590526066
b0766	ybhA	pyridoxal phosphatefructose-1,6-bisphosphate phosphatase	-0.113546275	7.301037342	0.424233538	0.583734012
b0810	glnP	L-glutamine ABC transporter membrane subunit	-0.11360698	7.696361599	0.452926858	0.61002809
b3735	atpH	ATP synthase Fsub1sub complex subunit δ	-0.114141482	8.787523066	0.493293073	0.645322699
b3415	gntT	high-affinity gluconate transporter	-0.114238776	6.848680061	0.523508796	0.671170628
b2263	menH	2-succinyl-6-hydroxy-2,4-cyclohexadiene-1-carboxylate synthase	-0.114250014	7.629308366	0.428564278	0.587977404
b2319	usg	putative semialdehyde dehydrogenase Usg	-0.114550199	8.34286423	0.468555414	0.623845643

b2614	grpE	nucleotide exchange factor GrpE	-0.114964397	8.683254771	0.495223975	0.646868003
b2748	ftsB	cell division protein FtsB	-0.115720271	7.4927231	0.451525779	0.608900508
b3730	glmU	fused N-acetylglucosamine-1-phosphate uridyltransferase and glucosan	-0.116137648	9.743056943	0.461773857	0.618734925
b4391	ettA	energy-dependent translational throttle protein EttA	-0.116357889	9.28368303	0.487669035	0.640791306
b2571	rseB	anti-sigma factor stabilizing protein RseB	-0.116488031	9.084986783	0.454807689	0.61181473
b3536	bcsE	c-di-GMP-binding protein BcsE	-0.116637077	9.131072272	0.456819316	0.613212915
b3507	dctR	putative DNA-binding transcriptional regulator DctR	-0.116744245	8.325118548	0.466764142	0.622618436
b0334	prpD	G6199-MONOMER	-0.116941047	5.150303905	0.783941024	0.870716885
b1091	fabH	β-ketoacyl-[acyl carrier protein] synthase III	-0.117618145	9.144538792	0.474453749	0.629231992
b3325	gspD	Type II secretion system protein GspD	-0.118181124	6.906841553	0.578746419	0.716889938
b0342	lacA	galactoside O-acetyltransferase	-0.118306507	5.028290893	0.784732385	0.870938532
b3843	ubiD	3-octaprenyl-4-hydroxybenzoate decarboxylase	-0.118674951	10.32162625	0.455283678	0.612268486
b4426	mcaS	small regulatory RNA McaS	-0.119027923	3.831030555	0.942644802	0.971696661
b3019	parC	DNA topoisomerase IV subunit A	-0.119299853	9.323272113	0.467851433	0.623472405
b0841	ybjG	undecaprenyl pyrophosphate phosphatase	-0.12008305	7.259873278	0.488451593	0.641182089
b3463	ftsE	cell division protein FtsE	-0.120104919	8.275673257	0.402334538	0.561766914
b4328	iadA	isoaspartyl dipeptidase	-0.120223403	8.464417458	0.425568795	0.584577927
b2429	murP	N-acetylmuramic acid-specific PTS enzyme IICB component anhydro-N-	-0.120238378	6.077680184	0.662584331	0.782750746
b3444	insA-6	IS1 protein InsA	-0.120487121	7.017982952	0.451537999	0.608900508
b2890	lysS	lysine—tRNA ligase, constitutive	-0.120491714	9.829289287	0.438875332	0.597850846
b4525	ymjC	putative uncharacterized protein YmjC	-0.121018199	3.900482946	0.823094524	0.897534072
b3987	rpoB	RNA polymerase subunit β	-0.121604641	11.70398081	0.39671024	0.556200607
b3304	rplR	50S ribosomal subunit protein L18	-0.121637599	8.785199131	0.491646134	0.643781134
b4679	yohP	uncharacterized membrane protein YohP	-0.121647471	3.955745835	0.834350598	0.903964913
b2268	rbn	ribonuclease BN	-0.121668212	6.824445634	0.495710077	0.647311443
b3559	glyS	glycine—tRNA ligase subunit β	-0.122201446	10.14550092	0.42480765	0.584093981
b1118	lolE	lipoprotein release complex - inner membrane subunit	-0.122288897	8.184025637	0.398408484	0.557872964
b4092	phnP	5-phospho-α-D-ribosyl 1,2-cyclic phosphate phosphodiesterase	-0.122513915	6.785558772	0.463482936	0.620319565
b2397	alaW	tRNA-Ala(GGC)	-0.122991051	6.547881495	0.519046247	0.668448069
b3195	mlaF	intermembrane phospholipid transport system, ATP binding subunit Mla	-0.123047251	8.808814303	0.44611277	0.604538945
b2794	queF	7-cyano-7-deazaguanine reductase	-0.123797724	7.808141085	0.361756365	0.523142598
b1787	yeaK	mischarged aminoacyl-tRNA deacylase	-0.123845728	7.178569674	0.396966999	0.556207331
b3972	murB	UDP-N-acetylenolpyruvoylglucosamine reductase	-0.123974035	9.844837031	0.462742326	0.619844468
b2714	ascG	DNA-binding transcriptional repressor AscG	-0.124012293	6.945318286	0.491694221	0.643781134
b4255	rraB	ribonuclease E inhibitor protein B	-0.126726037	8.241853184	0.374740581	0.535898826
b3216	yhcD	putative fimbrial usher protein YhcD	-0.127556598	8.130685965	0.523091798	0.671170628
b3129	prlF	antitoxin PrlF	-0.127612184	7.0964351	0.544972172	0.687443469
b0130	yadE	putative polysaccharide deacetylase lipoprotein	-0.127906539	7.378388556	0.380698646	0.542363511
b4431	rprA	small regulatory RNA RprA	-0.127909065	3.774888222	0.774053717	0.864702507
b0741	pal	peptidoglycan-associated outer membrane lipoprotein Pal	-0.128750013	9.02085443	0.434724604	0.593478394
b1545	pinQ	Qin prophage; putative site-specific recombinase	-0.128862914	4.731551896	0.784158059	0.870738891
b3020	ygiS	putative deoxycholate binding periplasmic protein	-0.129396631	8.149537714	0.368132394	0.529085267
b3360	pabA	aminodeoxychorismate synthase subunit 2	-0.129641509	6.765549759	0.506818246	0.657666542
b2874	yqeA	putative amino acid kinase YqeA	-0.129992686	5.452705069	0.736588158	0.834693559
b3196	yrbG	putative transport protein YrbG	-0.130205505	8.099837693	0.395202451	0.555490625
b3146	rsmI	16S rRNA 2'-O-ribose C1402 methyltransferase	-0.130507554	7.287489918	0.430303914	0.589448271
b1013	rutR	DNA-binding transcriptional dual regulator RutR	-0.130627689	6.27960513	0.574897194	0.713771404

b4168	tsaE	N6-L-threonylcarbamoyladenine synthase, TsaE subunit	-0.130847375	7.18267905	0.415287158	0.574483885
b2747	ispD	2-C-methyl-D-erythritol 4-phosphate cytidylyltransferase	-0.130994079	8.10606622	0.38480345	0.546272422
b4254	argI	ornithine carbamoyltransferase ArgI	-0.131259363	5.248803703	0.671772248	0.788377053
b0208	yafC	putative LysR family transcriptional regulator YafC	-0.13172399	7.204094851	0.442264323	0.601016775
b3456	livM	branched chain amino acidphenylalanine ABC transporter membrane su	-0.131842519	5.41438451	0.732152801	0.831178867
b0022	insA-1	IS1 protein InsA	-0.131884172	7.254126123	0.360327635	0.521744794
b2939	yqgB	acid stress response protein YqgB	-0.131914924	5.79881042	0.620242459	0.751061562
b0029	ispH	1-hydroxy-2-methyl-2-(E)-butenyl 4-diphosphate reductase	-0.13194546	8.275342189	0.375022986	0.535898826
b0686	ybfF	esterase	-0.131976492	6.271843465	0.538758923	0.682726936
b4323	uxuB	MANNONOXIDOREDUCT-MONOMER	-0.132597661	5.811918909	0.579387755	0.716889938
b0156	erpA	iron-sulfur cluster insertion protein ErpA	-0.132945842	8.215774189	0.359362365	0.520533084
b2889	idi	isopentenyl-diphosphate Δ-isomerase	-0.133221346	6.709451316	0.433892636	0.593443614
b2160	yeiI	putative sugar kinase YeiI	-0.133654713	7.352221361	0.38038903	0.542097342
b0902	pflA	pyruvate formate-lyase activating enzyme	-0.134185719	8.077131364	0.319410398	0.476256977
b2295	yfbV	UPF0208 membrane protein YfbV	-0.134495751	6.562516029	0.476487371	0.63013175
b2585	pssA	phosphatidylserine synthase	-0.134740705	8.908994922	0.392510473	0.55330617
b2815	metW	tRNA-initiator Met(CAU)	-0.13504024	6.955893075	0.44508877	0.603892723
b3783	rho	transcription termination factor Rho	-0.135315219	10.23247773	0.390500727	0.551699427
b4727	yacM	protein YacM	-0.135501878	5.552624242	0.675802393	0.79163374
b3714	adeP	adenine:H+ symporter	-0.135596239	8.0092581	0.332917997	0.49059845
b3469	zntA	Zn2+	-0.135740134	8.744542584	0.412818325	0.572063058
b2400	gltX	glutamate—tRNA ligase	-0.136017295	10.03826604	0.394577725	0.555115645
b4751	yoaL	protein YoaL	-0.136203788	4.116302279	0.717596876	0.819712861
b3604	lldR	DNA-binding transcriptional dual regulator LldR	-0.1362111	6.350159168	0.536704137	0.682080819
b2946	rsmE	16S rRNA m3U1498 methyltransferase	-0.136254928	7.827419112	0.352133391	0.512758938
b0292	ecpB	putative fimbrial chaperone EcpB	-0.136324359	5.246903198	0.736317014	0.834693559
b4288	fecD	ferric citrate ABC transporter membrane subunit FecD	-0.13666574	4.842107547	0.706338128	0.812316446
b2340	sixA	G7211-MONOMER	-0.137213677	7.50153283	0.302379018	0.455943772
b0169	rpsB	30S ribosomal subunit protein S2	-0.137256042	9.489270394	0.40886913	0.568374435
b0455	ffs	signal recognition particle 4.5S RNA	-0.137322938	6.77087276	0.44165938	0.600531617
b0206	aspU	tRNA-Asp(GUC)	-0.137426416	7.232312674	0.532529316	0.67931723
b4373	rimI	protein N-acetyltransferase RimI	-0.137500969	7.404873636	0.363467526	0.524929385
b2085	yegR	uncharacterized protein YegR	-0.138069316	4.364372845	0.756469872	0.849794436
b0266	yagB	CP4-6 prophage; orphan antitoxin YagB	-0.138119732	6.076857305	0.589765289	0.72529631
b2526	hscA	iron-sulfur cluster biosynthesis chaperone HscA	-0.138171912	9.787409723	0.406125811	0.566165284
b2006	yeeW	CP4-44 prophage; putative uncharacterized protein YeeW	-0.138338184	5.105282195	0.689591947	0.802252555
b1637	tyrS	tyrosine—tRNA ligase	-0.139112158	9.46190454	0.369000158	0.529972576
b0168	map	methionine aminopeptidase	-0.139295065	8.941139967	0.384585133	0.546138102
b0911	rpsA	30S ribosomal subunit protein S1	-0.139329622	10.60667063	0.398309371	0.557872964
b2575	yfiC	tRNA m6A37 methyltransferase	-0.139464484	6.534841775	0.544799722	0.687422341
b0642	leuS	leucine—tRNA ligase	-0.139719676	10.54131829	0.37542084	0.535898826
b3520	yhjB	putative DNA-binding transcriptional regulator YhjB	-0.140128065	5.895746863	0.609949443	0.74246672
b3312	rpmC	50S ribosomal subunit protein L29	-0.140762075	8.145038549	0.401528352	0.560818625
b2686	emrB	multidrug efflux pump membrane subunit EmrB	-0.140820245	7.677147482	0.315957061	0.473024899
b0132	rpnC	recombination-promoting nuclease RpnC	-0.141195607	7.147049065	0.387253154	0.548078072
b2409	yfeR	putative LysR-type DNA-binding transcriptional regulator YfeR	-0.141243528	6.042126839	0.639899914	0.76583305
b0028	fkpB	peptidyl-prolyl cis-trans isomerase FkpB	-0.141342633	7.629416239	0.272214189	0.42377491

b0296	ykgM	putative ribosomal protein	-0.141416586	5.986617078	0.630249644	0.758188604
b2270	yfbK	IPR002035DUF3520 domain-containing protein YfbK	-0.141531159	5.205185538	0.725235104	0.825776508
b0564	appY	DNA-binding transcriptional activator AppY	-0.141924674	8.954093244	0.417676334	0.57680357
b2593	pgeF	polyphenol oxidase YfiH	-0.142134849	7.618954126	0.298871242	0.452950314
b2482	hyfB	hydrogenase 4 component B	-0.142192845	4.87833589	0.723279804	0.825139848
b3237	argR	DNA-binding transcriptional dual regulator ArgR	-0.142202129	8.103468845	0.40661769	0.566516442
b0950	pqiA	intermembrane transport protein PqiA	-0.142207653	7.934875526	0.338685274	0.497271529
b2436	hemF	coproporphyrinogen III oxidase	-0.142298304	6.945117321	0.392230974	0.553276298
b4277	yjgZ	KpLE2 phage-like element; uncharacterized protein YjgZ	-0.142655809	3.340533056	0.856045785	0.915390943
b0792	ybhR	putative ABC exporter membrane subunit YbhR	-0.144034683	5.99859281	0.525719692	0.67354975
b1381	ydbH	PF11739 family protein YdbH	-0.144053784	7.378365174	0.327813176	0.485078571
b0740	tolB	Tol-Pal system periplasmic protein TolB	-0.14464952	9.810870914	0.371272205	0.532197008
b0632	dacA	D-alanyl-D-alanine carboxypeptidase DacA	-0.145233763	8.538607682	0.395553744	0.555637855
b4698	mgrR	small regulatory RNA MgrR	-0.145289565	5.493527103	0.633132999	0.760828032
b4545	ypdJ	CPS-53 (KpLE1) prophage; putative uncharacterized protein YpdJ	-0.145411485	3.488406654	0.866875195	0.92311963
b2510	yfgJ	zinc ribbon domain-containing protein YfgJ	-0.146130899	6.723956595	0.414553927	0.573927748
b2827	thyA	thymidylate synthase	-0.146192611	8.028354692	0.271566661	0.423127697
b0762	acrZ	multidrug efflux pump accessory protein AcrZ	-0.146408525	6.845068371	0.383193635	0.544512359
b0194	proS	proline—tRNA ligase	-0.147191321	9.9962283	0.360580865	0.521784503
b3092	uxaC	D-glucoronateD-galacturonate isomerase	-0.147319065	6.01821584	0.625347959	0.75473331
b4202	rpsR	30S ribosomal subunit protein S18	-0.147569396	8.110596085	0.326543203	0.4839504
b3227	dcuD	putative transporter DcuD	-0.14838	7.051041474	0.538425838	0.682696747
b0103	coaE	EG12312-MONOMER	-0.148913501	7.624189445	0.271606771	0.423127697
b2751	cysN	sulfate adenylyltransferase subunit 1	-0.149049297	9.977788175	0.333552686	0.491200772
b2826	ppdA	prepilin-type N-terminal cleavagemethylation domain-containing protei	-0.149259355	4.49648171	0.779145113	0.867703583
b1117	lolD	lipoprotein release complex - ATP binding subunit	-0.149657463	7.257789525	0.355590892	0.516425589
b4749	ynfR	Qin prophage; protein YnfR	-0.150198722	3.169100067	0.921810921	0.957148452
b0244	thrW	tRNA-Thr(CGU)	-0.150483039	6.984140001	0.456706934	0.613212915
b3186	rplU	50S ribosomal subunit protein L21	-0.151048305	8.689477589	0.367891929	0.529069338
b0305	rclR	DNA-binding transcriptional activator RclR	-0.151166712	5.350606053	0.657700611	0.7796908
b3316	rpsS	30S ribosomal subunit protein S19	-0.151376077	8.493507115	0.385560035	0.546637241
b3165	rpsO	30S ribosomal subunit protein S15	-0.151500455	8.329038447	0.329227622	0.486459154
b0098	secA	protein translocation ATPase	-0.152078623	10.21182215	0.31386783	0.470535303
b4279	yjhB	putative sialic acid transporter	-0.15216179	6.522346475	0.539026349	0.682869822
b2735	ygbI	putative DeoR-type DNA-binding transcriptional regulator YgbI	-0.152790479	6.934685795	0.371985778	0.533046806
b1816	yoaE	putative inner membrane protein	-0.152868801	6.343114039	0.471635462	0.627001073
b2806	rlmM	23S rRNA 2'-O-ribose C2498 methyltransferase	-0.153538055	7.86026461	0.261253587	0.411793855
b2775	yqcE	putative transport protein YqcE	-0.153572037	4.60225171	0.787148492	0.871494332
b0966	hspQ	heat shock protein, hemimethylated DNA-binding protein	-0.15363227	7.281132259	0.26960883	0.421801199
b2213	ada	DNA-binding transcriptional dual regulator DNA repair protein Ada	-0.153657957	7.106298426	0.326081414	0.483742108
b2430	yfeW	penicillin binding protein 4B	-0.153763068	6.389724706	0.495222195	0.646868003
b4306	yjhP	KpLE2 phage-like element; putative methyltransferase YjhP	-0.154322062	3.678036657	0.727053671	0.826665454
b3525	pdeH	c-di-GMP phosphodiesterase PdeH	-0.154577233	4.497677595	0.695210416	0.806410277
b3231	rplM	50S ribosomal subunit protein L13	-0.154672975	9.128308409	0.338619961	0.497271529
b3560	glyQ	glycine—tRNA ligase subunit α	-0.154902175	9.161262604	0.32997072	0.486950817
b1564	relB	Qin prophage; antitoxinDNA-binding transcriptional repressor RelB	-0.154939989	6.752858873	0.351398847	0.512021752
b2771	ygcS	putative transporter YgcS	-0.155471392	4.395862119	0.768997899	0.8601788

b3265	acrE	multidrug efflux pump membrane fusion lipoprotein AcrE	-0.155546615	6.727718156	0.655645464	0.778347599
b0216	aspV	tRNA-Asp(GUC)	-0.155712175	6.964494974	0.535759317	0.681272288
b4538	yoeF	CP4-44 prophage; putative uncharacterized protein YoeF	-0.155834082	5.287193272	0.589221017	0.725030878
b2042	galF	UTP:glucose-1-phosphate uridylyltransferase, low activity	-0.156362225	9.130366961	0.317922792	0.474628878
b4258	valS	valine—tRNA ligase	-0.156401632	10.1777335	0.342397756	0.501222179
b0162	cdaR	DNA-binding transcriptional activator CdaR	-0.156867085	6.472758442	0.465462148	0.621580335
b3193	mlaD	intermembrane phospholipid transport system, substrate binding protei	-0.158555611	8.258431377	0.277866572	0.430298462
b2694	argV	tRNA-Arg(ACG)	-0.158877698	7.443484124	0.297976445	0.451929236
b3648	gmk	guanylate kinase	-0.159013271	8.603512491	0.30771273	0.463195262
b0347	mhpA	putative 3-(3-hydroxyphenyl)propanoate3-hydroxycinnamate hydroxylas	-0.159448367	5.118105072	0.648200589	0.771792233
b0743	lysT	tRNA-Lys(UUU)	-0.160140189	7.081158901	0.321061331	0.478072775
b0759	galE	UDP-glucose 4-epimerase	-0.160290803	7.700158085	0.246631862	0.395381144
b0389	yaiA	protein YaiA	-0.160294626	7.05991956	0.341735869	0.500585223
b3033	yqiB	DUF1249 domain-containing protein YqiB	-0.160759334	7.760534983	0.248638005	0.397874879
b3031	yqiA	esterase YqiA	-0.160763809	8.128019889	0.248606012	0.397874879
b0947	ycbX	6-N-hydroxylaminopurine resistance protein	-0.160786308	7.495214432	0.281571512	0.433729754
b0121	speE	spermidine synthase	-0.160839427	7.907074127	0.2900739	0.443752946
b4146	epmB	lysine 2,3-aminomutase	-0.161103342	8.102049062	0.269473445	0.421801199
b0182	lpxB	lipid A disaccharide synthase	-0.161162695	8.440765836	0.280574379	0.432486402
b0055	djlA	co-chaperone protein DjlA	-0.161347092	7.980067218	0.240293477	0.388179912
b3191	mlaB	intermembrane phospholipid transport system protein MlaB	-0.161427798	7.738714565	0.26489748	0.41664495
b1667	ydhR	putative monooxygenase YdhR	-0.161841768	7.30825006	0.345930758	0.505388583
b0639	nadD	NICONUCADENYLYLTRAN-MONOMER	-0.162425738	7.806575463	0.267128029	0.419357635
b0354	yaiL	DUF2058 domain-containing protein YaiL	-0.162744122	7.327053357	0.24542773	0.394023065
b1875	yecM	putative metal-binding enzyme YecM	-0.163289769	6.249998257	0.456792866	0.613212915
b2592	clpB	ClpB chaperone	-0.164329988	10.49442478	0.306942054	0.462192759
b2136	yohD	DedA family protein YohD	-0.164493797	6.935442248	0.363461954	0.524929385
b3187	ispB	all-trans-octaprenyl-diphosphate synthase	-0.164903451	8.875720241	0.313095903	0.469697049
b4227	ytfQ	galactofuranose ABC transporter periplasmic binding protein	-0.16531822	5.539450442	0.567656741	0.707967376
b2609	rpsP	30S ribosomal subunit protein S16	-0.165384855	8.360217073	0.307898609	0.463317095
b0886	cydC	glutathioneL-cysteine ABC exporter subunit CydC	-0.165590263	8.285654881	0.29829414	0.452255711
b3230	rpsI	30S ribosomal subunit protein S9	-0.166126414	8.851398144	0.316473791	0.473317002
b1550	gnsB	Qin prophage; protein GnsB	-0.166638672	7.387032641	0.258544979	0.408691758
b3699	gyrB	DNA gyrase subunit B	-0.166944012	10.22660058	0.280453709	0.432486402
b3967	murI	glutamate racemase	-0.167237813	9.654916945	0.314649591	0.471547163
b3180	yhbY	ribosome assembly factor YhbY	-0.167597346	8.299349275	0.310096082	0.465707475
b3147	lpoA	outer membrane lipoprotein - activator of MrcA activity	-0.167742719	8.886417505	0.327814759	0.485078571
b4088	alsB	D-allose ABC transporter periplasmic binding protein	-0.168351208	4.853661564	0.616811028	0.747932075
b4506	ykgO	putative ribosomal protein	-0.16880294	5.591277978	0.641250175	0.766349056
b4161	rsgA	ribosome small subunit-dependent GTPase A	-0.169117824	8.653377089	0.278647763	0.430903633
b0180	fabZ	3-hydroxy-acyl-[acyl-carrier-protein] dehydratase	-0.169117894	8.443596173	0.268063313	0.42027682
b4447	sibD	small RNA SibD	-0.169343575	6.616965683	0.496937535	0.64814748
b2128	yehW	glycine betaine ABC transporter membrane subunit YehW	-0.169386143	6.555241597	0.381351109	0.542767616
b2981	yghO	putative DNA-binding transcriptional regulator YghO	-0.170081061	6.507211655	0.463786484	0.620319565
b2608	rimM	ribosome maturation factor RimM	-0.170163811	9.235167372	0.347437449	0.507086393
b2075	mdtB	multidrug efflux pump RND permease subunit MdtB	-0.170391533	7.490917979	0.353520713	0.514095503
b3240	aaeB	aromatic carboxylic acid efflux pump subunit AaeB	-0.170432673	7.246898656	0.3894218	0.550527457

b3908	sodA	superoxide dismutase (Mn)	-0.170539898	9.687714885	0.279310778	0.431558145
b4186	yjfC	putative acid—amine ligase YjfC	-0.172045519	3.952940217	0.737639737	0.835474458
b3757	gltU	tRNA-Glu(UUC)	-0.172132217	8.174795019	0.317245418	0.474277028
b2232	ubiG	bifunctional 3-demethylubiquinone-8 3-O	-0.172704233	6.919592796	0.29077774	0.444069412
b2316	accD	acetyl-CoA carboxyltransferase subunit β	-0.172720468	9.177182008	0.28344932	0.435279564
b3071	nfeR	DNA-binding transcriptional repressor NfeR	-0.173415849	6.687182495	0.390757894	0.55188615
b2069	yegD	HSP70 family protein YegD	-0.174150854	4.711528655	0.654421459	0.777184613
b3009	yghB	DedA family protein YghB	-0.174539743	7.631890003	0.212849421	0.354676112
b4595	yciY	uncharacterized protein YciY	-0.174797948	7.167317508	0.271486648	0.423127697
b2182	bcr	multidrug efflux pump Bcr	-0.175996246	8.240102292	0.226604285	0.371641128
b2606	rplS	50S ribosomal subunit protein L19	-0.176044872	8.526538063	0.272406495	0.423924806
b3194	mlaE	intermembrane phospholipid transport system, integral membrane sub	-0.17638378	8.597527209	0.278964064	0.431241716
b4502	yeiW	PF03692 putative metal-chelating domain-containing protein YeiW	-0.176419198	5.024498496	0.636980296	0.763373509
b1162	bluR	DNA-binding transcriptional repressor BluR	-0.176557624	7.360520675	0.245102911	0.393787973
b0665	glnV	tRNA-Gln(CUG)	-0.176923886	7.030973848	0.259313883	0.40961388
b1583	ynfB	DUF1283 domain-containing protein YnfB	-0.177635534	7.496272355	0.185440725	0.321066981
b1968	hprS	sensory histidine kinase HprS - phosphorylated	-0.177826357	7.02017794	0.342973416	0.501732151
b0267	yagA	CP4-6 prophage; integrase core domain-containing protein YagA	-0.178363551	5.197598799	0.657892219	0.7796908
b1159	mcrA	e14 prophage; 5-methylcytosine-specific restriction enzyme McrA	-0.179217334	5.973555479	0.429075548	0.588313212
b4664	ibsD	putative toxic peptide IbsD	-0.17968965	6.352394984	0.405778126	0.566038049
b1909	leuZ	tRNA-Leu(UAA)	-0.179717111	6.910056959	0.312385536	0.468790666
b1960	vsr	DNA mismatch endonuclease of the very short patch (VSP) mismatch re	-0.179755865	6.105499944	0.430066465	0.589305848
b0272	xynR	CP4-6 prophage; DNA-binding transcriptional repressor XynR	-0.179922972	6.623881604	0.354149644	0.514501704
b2528	iscA	iron-sulfur cluster insertion protein IscA	-0.180275346	8.102261029	0.184104854	0.319254882
b4701	sokX	small RNA SokX	-0.180329523	4.065545942	0.71218525	0.815853108
b0324	yahJ	putative deaminase with metallo-dependent hydrolase domain	-0.180401058	7.531440914	0.171405391	0.303308538
b3248	yhdE	nucleoside triphosphate pyrophosphatase YhdE	-0.180413525	8.493148318	0.258642061	0.408698891
b4234	yjgA	DUF615 domain-containing protein YjgA	-0.180758934	7.738373826	0.242938659	0.390879803
b4155	epmA	EF-P-lysine lysyltransferase	-0.180761265	6.827061018	0.393884383	0.554351148
b3166	truB	tRNA pseudouridine55 synthase	-0.180876797	8.654559501	0.254175343	0.403518209
b0270	yagG	putative D-xylonate transporter YagG	-0.181099424	5.39019043	0.565629411	0.705638274
b4443	gcvB	small regulatory RNA GcvB	-0.181305666	7.53832547	0.23090062	0.376595088
b0735	ybgE	PF09600 family protein YbgE	-0.18153953	6.989928401	0.27717786	0.429533258
b2121	yehP	VWA domain-containing protein YehP	-0.181876045	3.327086718	0.851634261	0.912326751
b4418	sraB	small RNA SraB	-0.181900275	6.577920298	0.317330542	0.474277028
b1782	mipA	scaffolding protein that interacts with murein polymerase and murein h	-0.182040042	7.886409043	0.205838177	0.345936639
b0579	ybdF	PF04237 family protein YbdF	-0.182948392	5.477188327	0.550101854	0.691481346
b3032	cpdA	cAMP phosphodiesterase	-0.183025301	8.617529192	0.235570164	0.381947217
b3341	rpsG	30S ribosomal subunit protein S7	-0.183298983	9.116733628	0.303199563	0.456868966
b2161	nupX	putative nucleoside transporter	-0.183816339	4.233564244	0.687426238	0.801099932
b2299	yfcD	putative Nudix hydrolase	-0.184097486	7.92478191	0.211814715	0.35342478
b2758	casC	type I-E CRISPR system Cascade subunit CasC	-0.184152561	5.200464275	0.635506784	0.761979039
b1200	dhaK	dihydroxyacetone kinase subunit K	-0.184917395	8.860534723	0.256646844	0.406564698
b2685	emrA	multidrug efflux pump membrane fusion protein EmrA	-0.185266182	7.592051779	0.154886802	0.281178138
b3255	accB	biotin carboxyl carrier protein	-0.185573705	8.830416614	0.25191835	0.400944309
b2065	dcd	dCTP deaminase	-0.185723633	7.851597577	0.174306056	0.306842598
b4053	alr	alanine racemase 1	-0.186081306	8.619883398	0.23529365	0.38163904

b3636	rpmG	50S ribosomal subunit protein L33		-0.186360105	7.545304952	0.184938847	0.320411999
b0330	prpR	DNA-binding transcriptional dual regulator PrpR		-0.18732694	5.899116146	0.43649079	0.595521273
b1109	ndh	NADH:quinone oxidoreductase II		-0.187685091	8.229976923	0.250463951	0.399349347
b0670	glnU	tRNA-Gln(UUG)		-0.187831739	7.324138632	0.176410568	0.309373241
b2422	cysA	sulfatethiosulfate ABC transporter ATP binding subunit		-0.1888592	9.18211395	0.230644821	0.376589085
b1557	cspB	Qin prophage; cold shock-like protein CspB		-0.189069927	8.08143813	0.210616555	0.351957643
b3175	secG	Sec translocon subunit SecG		-0.189121858	8.563239482	0.230776426	0.376595088
b3235	degS	serine endoprotease		-0.189270927	8.120103828	0.249355377	0.398301009
b3036	ygiA		0	-0.18932652	6.718181779	0.293109311	0.446476436
b3747	kup	K+:H+		-0.190212742	8.018839333	0.176382727	0.309373241
b0279	yagM	CP4-6 prophage; protein YagM		-0.190414192	5.694510205	0.615235475	0.746431608
b3577	yiaM	2,3-diketo-L-gulonate:Na+ symporter - membrane subunit		-0.190484021	4.020951184	0.725336648	0.825776508
b0504	allS	DNA-binding transcriptional activator AllS		-0.191053314	3.639551479	0.831028651	0.903127722
b4429	sokB	putative small regulatory RNA SokB		-0.191078036	4.515252688	0.608671384	0.741319768
b2348	argW	tRNA-Arg(CCU)		-0.191967911	4.637312549	0.602082027	0.737158111
b2537	hcaR	DNA-binding transcriptional dual regulator HcaR		-0.1920067	5.627422795	0.508524391	0.658977161
b2936	loiP	metalloprotease LoiP		-0.192123623	8.327067481	0.19759497	0.33656705
b3680	yidL	putative DNA-binding transcriptional regulator YidL		-0.19213353	5.022102744	0.536935623	0.68217864
b0984	gfcD	putative lipoprotein GfcD		-0.192253733	5.895835468	0.526680845	0.674389771
b1373	tfaR	Rac prophage; putative tail fiber assembly protein TfaR		-0.192738269	4.709577743	0.705658203	0.812131564
b3919	tpiA	triose-phosphate isomerase		-0.193688719	9.944644709	0.273370606	0.424975783
b1831	proQ	RNA chaperone ProQ		-0.193697856	9.03421938	0.243788359	0.392104045
b1060	bssS	regulator of biofilm formation		-0.194272793	7.283708632	0.170925206	0.302701478
b1361	ydaW	Rac prophage; putative uncharacterized protein YdaW		-0.195200328	4.894419016	0.654129824	0.777175235
b0184	dnaE	DNA polymerase III subunit α		-0.195607183	9.665148355	0.237414273	0.384372576
b3256	accC	biotin carboxylase		-0.195799229	9.824415104	0.203133771	0.342958165
b4672	ymiB	putative protein YmiB		-0.196000604	4.301489445	0.663716853	0.78308656
b0345	lacI	DNA-binding transcriptional repressor LacI		-0.196013769	7.731882428	0.12435667	0.237266507
b0980	appA	periplasmic phosphoanhydride phosphatasemultiple inositol-polyphospl		-0.196045429	8.911483221	0.211308962	0.352714203
b0401	brnQ	branched chain amino acid transporter BrnQ		-0.196342651	8.214931235	0.16946019	0.30119434
b2752	cysD	sulfate adenylyltransferase subunit 2		-0.19675424	9.306826709	0.201937731	0.341591986
b3566	xylF	xylose ABC transporter periplasmic binding protein		-0.197023943	4.29076975	0.70676339	0.812593846
b1610	tus	DNA replication terminus site-binding protein		-0.197073945	6.926917263	0.222146345	0.365669279
b0387	yaiI	DUF188 domain-containing protein YaiI		-0.197110052	7.011302522	0.208255302	0.348671657
b0070	setA	B0070-MONOMER		-0.197396896	7.262227892	0.230904395	0.376595088
b0683	fur	DNA-binding transcriptional dual regulator Fur		-0.197651292	8.538147502	0.210769163	0.3520794
b3167	rbfA	30S ribosome binding factor		-0.19784776	7.733362765	0.132302929	0.248796457
b1741	cho	endonuclease of nucleotide excision repair		-0.19798951	6.430184578	0.31770558	0.474516284
b2642	yfjW	CP4-57 prophage; uncharacterized protein YfjW		-0.197992608	8.79070186	0.259895435	0.41023895
b1210	hemA	glutamyl-tRNA reductase		-0.198184519	8.543574405	0.201611839	0.34130677
b2209	eco	serine protease inhibitor ecotin		-0.198635376	7.448442661	0.157998019	0.285885759
b3711	yidZ	putative LysR-type transcriptional regulator YidZ		-0.198751964	7.184661574	0.189970632	0.327497205
b2291	yfbR	dCMP phosphohydrolase		-0.199042065	6.219897544	0.399780337	0.559034073
b1638	pdxH	pyridoxal 5-phosphate synthase		-0.199600532	8.45881343	0.227799111	0.373046393
b4177	purA	adenylosuccinate synthetase		-0.199697718	9.348254353	0.214545175	0.356230517
b0689	ybfP	lipoprotein YbfP		-0.200751252	5.787948465	0.422607455	0.582520111
b3041	ribB	3,4-dihydroxy-2-butanone-4-phosphate synthase		-0.20148487	8.515814747	0.194816806	0.333490421

b3723	bglG	transcriptional antiterminator BglG	-0.201600844	5.245743166	0.581924897	0.71885798
b0021	insB-1	IS1 protein InsB	-0.202169757	7.672212858	0.131813043	0.248274141
b1729	tcyP	cystinesulfocysteine:cation symporter	-0.202243715	9.749821155	0.191077526	0.329148372
b4233	mpl	UDP-N-acetylmuramate—L-alanyl-γ-D-glutamyl-meso	-0.202859832	9.3049852	0.206862533	0.347102504
b2460	eutQ	putative ethanolamine utilization acetate kinase EutQ	-0.203044584	3.490516456	0.834053675	0.903964913
b4729	ykgV	protein YkgV	-0.203045504	5.235031243	0.608106646	0.740836324
b0385	dgcC	diguanylate cyclase DgcC	-0.203155536	5.661148383	0.50511419	0.656098602
b2875	yqeB	XdhC-CoxI family protein YqeB	-0.203235893	6.174617712	0.482089159	0.635729879
b3185	rpmA	50S ribosomal subunit protein L27	-0.203398467	8.332869242	0.203611299	0.343632984
b4018	iclR	DNA-binding transcriptional repressor IclR	-0.203593972	8.762042671	0.205420821	0.345629926
b1514	lsrC	Autoinducer-2 ABC transporter membrane subunit LsrC	-0.203720365	3.028753937	0.905223106	0.945260173
b4178	nsrR	DNA-binding transcriptional repressor NsrR	-0.20381469	7.789634399	0.113717709	0.221563851
b1871	cmoB	tRNA U34 carboxymethyltransferase	-0.203956968	7.840873883	0.146254953	0.269048175
b2529	iscU	scaffold protein for iron-sulfur cluster assembly	-0.20447742	8.41085604	0.173722903	0.306060102
b2891	prfB	peptide chain release factor RF2	-0.204545097	9.374666534	0.2070036	0.347102504
b1207	prs	PRPPSYN-MONOMER	-0.204561572	9.172206889	0.253357622	0.402364712
b3390	aroK	shikimate kinase 1	-0.205009665	8.409851659	0.198360987	0.337351216
b4063	soxR	DNA-binding transcriptional dual regulator SoxR	-0.205103041	5.848505382	0.454718284	0.61181473
b2961	mutY	adenine DNA glycosylase	-0.205160333	7.529597712	0.165678976	0.295663976
b2077	mdtD	putative multidrug efflux pump MdtD	-0.206020969	5.621326002	0.521750409	0.670141945
b1194	ycgR	flagellar brake protein YcgR	-0.206163216	5.199419486	0.476464239	0.63013175
b4419	ldrA	small toxic polypeptide LdrA	-0.206260058	6.107721241	0.365487417	0.526358008
b3067	rpoD	RNA polymerase, sigma 70 (sigma D) factor	-0.20626287	9.872464416	0.165760782	0.295690446
b1211	prfA	peptide chain release factor RF1	-0.206566529	8.385720745	0.184989849	0.320411999
b0765	modC	molybdate ABC transporter ATP binding subunit	-0.207601693	6.732283652	0.267543821	0.419760472
b2638	yfjU	CP4-57 prophage; ArsC family protein YfjU	-0.207630829	6.615900328	0.282115412	0.434137171
b3342	rpsL	30S ribosomal subunit protein S12	-0.207752247	8.667129392	0.249718159	0.398592073
b3389	aroB	AROB-MONOMER	-0.208063107	9.118299121	0.238970811	0.386184527
b2219	atoS	sensory histidine kinase AtoS	-0.208181807	6.235966314	0.329712711	0.486950817
b3169	nusA	transcription terminationantitermination protein NusA	-0.208314136	9.796012718	0.194595201	0.333428456
b1975	serU	tRNA-Ser(CGA)	-0.208329854	5.070478224	0.534268304	0.680356089
b0598	cstA	carbon starvation protein A	-0.208679034	6.71733055	0.251908201	0.400944309
b3219	yhcF	DUF1120 domain-containing protein YhcF	-0.208679145	7.59710507	0.360435248	0.521744794
b2508	guaB	inosine 5'-monophosphate dehydrogenase	-0.209526212	9.836787579	0.197545439	0.33656705
b0843	ybjH	uncharacterized protein YbjH	-0.209556453	5.619495922	0.439696472	0.598600038
b1065	mdtH	multidrug efflux pump MdtH	-0.209729714	7.336111322	0.14383585	0.26559401
b1657	ydhP	putative transporter YdhP	-0.21023288	6.747599627	0.231750822	0.377417883
b2405	xapR	DNA-binding transcriptional activator XapR	-0.210778952	6.214085563	0.382790617	0.544114802
b3392	hofP	DNA utilization protein HofP	-0.210966014	4.626265546	0.575186188	0.713929441
b2843	kduI	5-dehydro-4-deoxy-D-glucuronate isomerase	-0.211529276	5.813242094	0.51690461	0.666871575
b2600	tyrA	fused chorismate mutaseprephenate dehydrogenase	-0.211545351	7.907205345	0.120250598	0.232039506
b3106	yhaK	bicupin-related protein	-0.211939331	5.311404682	0.58020525	0.717134988
b0930	asnS	asparagine—tRNA ligase	-0.212169739	9.428267408	0.188417264	0.325073162
b0407	yajC	Sec translocon accessory complex subunit YajC	-0.212223046	7.9054101	0.132939626	0.249650552
b3154	yhbP	putative FMN binding protein YhbP	-0.212573868	6.28875543	0.411968687	0.571148104
b3072	aer	aerotaxis sensor receptor, flavoprotein	-0.212606679	5.977329345	0.51539633	0.665927655
b0840	deoR	DNA-binding transcriptional repressor DeoR	-0.212919994	7.377959343	0.164071505	0.294103002

b2399	yfeD	putative DNA-binding transcriptional regulator YfeD	-0.21349254	6.758234732	0.241020622	0.38921216
b1904	yecR	lipoprotein YecR	-0.213552946	4.147251507	0.578268917	0.716547086
b3501	arsR	DNA-binding transcriptional repressor ArsR	-0.214016441	4.962871176	0.569162657	0.709244463
b3352	yheS	ABC transporter ATP-binding protein YheS	-0.214258957	8.469245025	0.198751137	0.337624575
b3344	tusC	sulfurtransferase complex subunit TusC	-0.214342984	7.167979078	0.197938977	0.336981325
b2300	yfcE	phosphodiesterase YfcE	-0.215408385	7.787902078	0.170919095	0.302701478
b1814	sdaA	L-serine deaminase I	-0.216003417	8.815701042	0.172140843	0.30413805
b0537	intD	DLP12 prophage; putative integrase	-0.216089711	8.174204249	0.119403121	0.231009981
b3114	tdcE	activated 2-ketobutyrate formate-lyase pyruvate formate-lyase 4	-0.21631428	5.704691257	0.517577096	0.667378178
b4203	rplI	50S ribosomal subunit protein L9	-0.216598248	8.494454706	0.157613993	0.285307823
b2317	dedA	DedA family protein DedA	-0.217337518	7.883732888	0.164607319	0.294477675
b3568	xylH	xylose ABC transporter membrane subunit	-0.218111584	5.080217292	0.481763479	0.635490218
b4278	insG	KpLE2 phage-like element; IS4 putative transposase	-0.218132927	7.404072405	0.124552458	0.23733237
b1616	uidB	glucuronide:H+ symporter	-0.219268791	3.69624518	0.748613839	0.843267013
b2937	speB	agmatinase	-0.219629075	8.299733966	0.150768504	0.275857001
b1540	ydfH	DNA-binding transcriptional repressor YdfH	-0.220598775	7.572161205	0.107084042	0.211343784
b0231	dinB	DNA polymerase IV	-0.220743759	7.251097762	0.206989099	0.347102504
b2550	yphH	putative DNA-binding transcriptional regulator, NAGC-like	-0.221395618	6.276040461	0.394679051	0.555115645
b4433	sdsR	small regulatory RNA SdsR	-0.221716616	6.785646598	0.251808952	0.400944309
b3394	hofN	DNA utilization protein HofN	-0.221759973	4.663674375	0.559224017	0.69922799
b2378	lpxP	PALMITOTRANS-MONOMER	-0.221784694	8.25508987	0.15379606	0.280003961
b3065	rpsU	30S ribosomal subunit protein S21	-0.221792573	8.219836861	0.196107328	0.334678722
b1533	eamA	cysteineO	-0.221950669	6.802651558	0.255682889	0.40532853
b3399	yrfG	purine nucleotidase	-0.222303913	7.662127451	0.082498955	0.172540449
b1894	insA-5	IS1 protein InsA	-0.222678757	7.246602364	0.145874533	0.268572169
b3274	rrfD	5S ribosomal RNA	-0.222837922	8.913559938	0.290854429	0.444069412
b4080	mdtP	putative multidrug efflux pump outer membrane channel	-0.223249087	4.593776524	0.625496984	0.75473331
b3403	pck	PEPCARBOXYKIN-MONOMER	-0.223375446	8.881853239	0.151779806	0.277133104
b1607	ydgC	GlpM family protein	-0.223408377	5.319861224	0.45406985	0.611194631
b1270	btuR	cobinamidecobalamin adenosyltransferase	-0.223815129	6.368077336	0.316373229	0.473317002
b4084	alsK	EG11956-MONOMER	-0.223948042	5.422421994	0.498774452	0.649587318
b0696	kdpC	K+ transporting P-type ATPase subunit KdpC	-0.224771727	5.368687489	0.447656303	0.60588675
b2764	cysJ	sulfite reductase, flavoprotein subunit	-0.224951486	10.2278331	0.147711446	0.271162593
b2532	trmJ	tRNA Cm32Um32 methyltransferase	-0.225093363	8.394510954	0.120539167	0.232393198
b1863	ruvC	crossover junction endodeoxyribonuclease RuvC	-0.225135762	7.480934268	0.106216206	0.210082042
b0195	trmO	tRNA m6t6	-0.225255701	7.137489025	0.155963858	0.282668487
b0923	mukE	chromosome partitioning protein MukE	-0.22543783	8.057342797	0.122226909	0.23441868
b2825	ppdB	prepilin-type N-terminal cleavagemethylation domain-containing protei	-0.22547441	4.7638686	0.640303967	0.766108947
b2102	yegX	putative hydrolase	-0.226777621	6.686609319	0.287104012	0.440279337
b3462	ftsX	cell division protein FtsX	-0.2268879	8.539572846	0.13231576	0.248796457
b1113	ldtC	L,D-transpeptidase LdtC	-0.227059642	4.540782364	0.596932443	0.731868018
b1357	ydaS	Rac prophage; toxin YdaS	-0.227153096	4.555146534	0.569677081	0.70945972
b2402	valX	tRNA-Val(UAC)	-0.227382283	6.908911117	0.181240458	0.315900759
b0286	paoA	aldehyde dehydrogenase, Fe-S subunit	-0.227463848	5.51490108	0.499679176	0.650378408
b3236	mdh	malate dehydrogenase	-0.227480202	8.970302257	0.191717536	0.32986474
b0183	rnhB	RNase HII	-0.227660449	7.579267137	0.116598424	0.226377327
b4501	torI	CPS-53 (KpLE1) prophage; prophage CPS-53 recombination directionality	-0.228960008	5.849780385	0.38578992	0.546637241

b0348	mhpB	3-carboxyethylcatechol 2,3-dioxygenase	-0.229115344	4.996594871	0.529276125	0.676731564
b1737	chbC	N,N'-diacetylchitobiose-specific PTS enzyme IIC component	-0.229682565	5.160450951	0.469639121	0.624598997
b0628	lipA	lipoyl synthase	-0.230917842	8.813557286	0.144286323	0.266091946
b2972	pppA	prepilin peptidase	-0.231131163	5.07783127	0.493310104	0.645322699
b0147	thpR	RNA 2',3'-cyclic phosphodiesterase	-0.231193275	6.469233333	0.253137509	0.402159806
b4246	pyrL	pyrBI operon leader peptide	-0.231971556	4.330686788	0.606158433	0.739687529
b0170	tsf	protein chain elongation factor EF-Ts	-0.232424436	9.426230766	0.145509142	0.268011206
b4200	rpsF	30S ribosomal subunit protein S6	-0.23265038	8.55184604	0.181069575	0.315753382
b2565	recO	DNA repair protein RecO	-0.232823056	7.859191177	0.092140618	0.188207383
b2143	cdd	cytidinedeoxycytidine deaminase	-0.233001368	8.223290038	0.174727632	0.307462134
b1994	insH-6	CP4-44 prophage; IS5 transposase and trans-activator	-0.233162908	8.647481368	0.130917333	0.247114163
b1584	speG	spermidine N-acetyltransferase	-0.233359361	7.709429717	0.065703766	0.14346297
b1531	marA	DNA-binding transcriptional dual regulator MarA	-0.233752377	7.672282023	0.091284745	0.186843833
b2570	rseC	protein RseC	-0.234026187	7.822480703	0.070764571	0.152254183
b3613	envC	murein hydrolase activator EnvC	-0.234316853	8.69222428	0.146862638	0.269941111
b2164	psuT	YEIM-MONOMER	-0.234371625	4.80570392	0.551794803	0.693123738
b2410	yfeH	putative solute:Na+ symporter	-0.235018871	7.452711979	0.110525231	0.216394189
b3262	yhdJ	DNA adenine methyltransferase	-0.235141709	6.88516575	0.446859051	0.60536444
b1061	dinI	DNA damage-inducible protein I	-0.235341841	6.386908299	0.207412671	0.347524456
b2899	yqfA	hemolysin-III family protein YqfA	-0.236394763	7.831955002	0.071167356	0.152822896
b3418	malT	DNA-binding transcriptional activator MalT	-0.23655266	9.244202148	0.15044862	0.275500065
b1869	yecN	putative inner membrane protein	-0.236553401	7.0582352	0.122222223	0.23441868
b4432	ryeA	small RNA RyeA	-0.236608018	7.115787024	0.194882285	0.333490421
b3288	fmt	10-formyltetrahydrofolate:L-methionyl-tRNAfMet N	-0.237084114	9.087565734	0.132532585	0.249098069
b1043	csgC	inhibitor of CsgA amyloid formation	-0.237394914	2.441155404	0.872175149	0.926528701
b0138	yadM	putative fimbrial protein YadM	-0.237543999	4.876488682	0.602796047	0.737520981
b3576	yiaL	DUF386 domain-containing protein YiaL	-0.237636905	4.01359792	0.614874668	0.746431608
b2948	yqgE	DUF179 domain-containing protein YqgE	-0.237982042	7.683942346	0.067780184	0.147340972
b2960	trmI	tRNA m7G46 methyltransferase	-0.23837353	7.705401937	0.098364726	0.197849779
b4381	deoC	DEOXYRIBOSE-P-ALD-MONOMER	-0.238495733	7.827416433	0.103417686	0.205763445
b3289	rsmB	16S rRNA m5C967 methyltransferase	-0.238498035	9.043887109	0.119522526	0.231128725
b2762	cysH	phosphoadenosine phosphosulfate reductase	-0.240043106	8.95128365	0.139505235	0.259047489
b2873	hyuA	phenylhydantoinase	-0.240165594	5.455230443	0.484332086	0.637735211
b1329	mppA	murein tripeptide ABC transporter periplasmic binding protein	-0.241068149	7.472347944	0.070347572	0.151869043
b0717	ybgP	putative fimbrial chaperone YbgP	-0.241098972	4.693583496	0.577593093	0.715910586
b3017	ftsP	cell division protein required during stress conditions	-0.241308975	8.54021842	0.114398897	0.222694503
b0140	yadV	putative fimbrial chaperone YadV	-0.2414614	5.003857165	0.553705069	0.694096501
b0106	hofC	inner membrane protein HofC	-0.241496913	6.315006723	0.290173218	0.443752946
b0049	apaH	diadenosine tetraphosphatase	-0.242191595	7.957515258	0.094164396	0.191230823
b4208	cycA	serinealanine	-0.243030007	8.178551876	0.116722369	0.226518355
b1407	ydbD	DUF2773 domain-containing protein YdbD	-0.243148504	4.504191618	0.516944448	0.666871575
b2530	iscS	cysteine desulfurase	-0.243161978	9.757984588	0.137294834	0.255978333
b2210	mqo	EG12069-MONOMER	-0.243735092	9.24570498	0.237824035	0.384894836
b0107	hofB	T2SSE family protein	-0.245264527	6.591430566	0.20795059	0.348293572
b2749	ygbE	conserved inner membrane protein YgbE	-0.245663368	7.515360419	0.067040939	0.145877648
b1206	dauA	aerobic C4-dicarboxylate transporter DauA	-0.245917125	7.417724733	0.108389234	0.213157447
b4517	gnsA	putative phosphatidylethanolamine synthesis regulator GnsA	-0.246382357	5.853226217	0.332622398	0.490326506

b1865	nudB	dihydroneopterin triphosphate diphosphatase	-0.246396531	7.850620351	0.109037726	0.214146601
b4598	yncL	uncharacterized protein YncL	-0.246885707	6.59084047	0.175283709	0.308194972
b2785	rlmD	23S rRNA m5U1939 methyltransferase	-0.246973069	8.060520894	0.068629348	0.148893646
b1703	ppsR	posphoenolpyruvate synthetase regulatory protein	-0.247474675	6.209578488	0.275556992	0.427753039
b4611	sibE	small RNA SibE	-0.249010142	6.604397984	0.300122643	0.45430296
b0388	aroL	shikimate kinase 2	-0.249030489	7.596542219	0.055443864	0.125209545
b4298	yjhH	KpLE2 phage-like element; putative 2-dehydro-3-deoxy-D-pentonate ald	-0.24993167	5.060563858	0.476184722	0.63013175
b0432	cyoA	cytochrome bosub3	-0.250070065	9.56764228	0.1548132	0.28116013
b0436	tig	trigger factor	-0.250306088	9.832907339	0.113208703	0.220766971
b4160	psd	phosphatidylserine decarboxylase proenzyme	-0.250357314	8.278634277	0.130866239	0.247114163
b1427	rimL	ribosomal-protein-L12-serine acetyltransferase	-0.250413467	7.059956029	0.126949392	0.2409637
b1653	lhr	putative ATP-dependent helicase Lhr	-0.250691151	7.738862676	0.090622318	0.185918928
b4537	yecJ	DUF2766 domain-containing protein YecJ	-0.250732357	6.278880622	0.349348792	0.509370844
b2691	argQ	tRNA-Arg(ACG)	-0.250896292	7.032037095	0.23203148	0.377735613
b3464	ftsY	signal recognition particle receptor	-0.252727049	9.180243252	0.121152712	0.233264112
b4752	yecU	protein YecU	-0.253089923	4.69023527	0.529634582	0.676993828
b0847	ybjL	putative transport protein YbjL	-0.253200084	8.106216959	0.098461891	0.197864928
b3189	murA	UDPNACETYLGLUCOSAMENOLPYRTRANS-MONOMER	-0.253882262	8.929045908	0.10867601	0.21353119
b0232	yafN	antitoxin YafN	-0.254107853	6.37137548	0.294539487	0.447793332
b0900	ycaN	putative LysR-type transcriptional regulator YcaN	-0.254598072	5.380081187	0.424975337	0.584142625
b0805	fiu	putative iron siderophore outer membrane transporter	-0.255156216	5.18727099	0.434515568	0.593478394
b1562	hokD	Qin prophage; toxic protein HokD	-0.255295729	6.893676027	0.125869265	0.239222043
b0682	chiQ	lipoprotein ChiQ	-0.2555201	4.978756879	0.395323113	0.555490625
b2984	yghR	putative ATP-binding protein YghR	-0.255981243	3.946703608	0.714888944	0.81767738
b2763	cysI	sulfite reductase, hemoprotein subunit	-0.256771449	10.15014302	0.109325425	0.214520779
b4169	amiB	N-acetylmuramoyl-L-alanine amidase B	-0.2568784	8.478355059	0.106254405	0.210082042
b2486	hyfF	hydrogenase 4 component F	-0.257104095	5.066667936	0.422156132	0.582443538
b4140	fxsA	protein FxsA	-0.257421985	6.258060446	0.364947399	0.525862522
b0144	gluQ	glutamyl-Q tRNAAsp synthetase	-0.257562029	7.148441373	0.10693666	0.211147296
b1866	aspS	aspartate—tRNA ligase	-0.257794866	9.682609232	0.100370302	0.20100973
b3259	prmA	methyltransferase for 50S ribosomal subunit protein L11	-0.257829995	8.145808292	0.107885925	0.212356825
b0239	frsA	fermentation-respiration switch protein	-0.258010213	8.408537298	0.098610123	0.197982579
b3544	dppA	dipeptide ABC transporter periplasmic binding protein	-0.258021991	8.360107747	0.172574073	0.304643955
b1910	cysT	tRNA-Cys(GCA)	-0.258818134	6.957824526	0.122890739	0.235385082
b0640	holA	DNA polymerase III subunit δ	-0.258872102	8.336715937	0.084383727	0.175816024
b4423	ldrC	small toxic polypeptide LdrC	-0.259629929	5.896959859	0.271349733	0.423127697
b3091	uxaA	ALTRODEHYDRAT-MONOMER	-0.259854192	6.236703068	0.325871511	0.483604276
b1023	pgaB	poly-β-1,6-N-acetyl-D-glucosamine N	-0.260013199	4.275168781	0.579682237	0.716889938
b1088	yceD	DUF177 domain-containing protein YceD	-0.26029627	8.914762853	0.13003444	0.245868117
b0319	yahE	DUF2877 domain-containing protein YahE	-0.260654818	4.935957906	0.506917156	0.657666542
b3445	insB-6	IS1 protein InsB	-0.261156549	7.082556648	0.09003819	0.18506453
b1665	valV	tRNA-Val(GAC)	-0.261160366	5.637596165	0.352752152	0.513300902
b0331	prpB	2-methylisocitrate lyase	-0.261168158	5.205337768	0.383336165	0.544539629
b3724	phoU	negative regulator of the pho regulon	-0.261199229	7.651711397	0.049875431	0.115414013
b1032	serX	tRNA-Ser(GGA)	-0.261242768	5.950242877	0.252817761	0.401883782
b3363	ppiA	peptidyl-prolyl cis-trans isomerase A	-0.261257258	7.608142236	0.070516137	0.152090252
b0312	betB	betaine aldehyde dehydrogenase	-0.261895514	8.311356282	0.089231183	0.184005453

b3110	cyuP	putative DL-serine transporter	-0.262026229	6.020304944	0.476565875	0.63013175
b4027	yjbF	lipoprotein YjbF	-0.263278157	5.13080731	0.505304225	0.656152398
b1293	sapB	putrescine ABC exporter membrane subunit SapB	-0.263441595	6.760942405	0.176444353	0.309373241
b2165	psuG	pseudouridine-5'-phosphate glycosidase	-0.264595305	2.221271696	0.707220547	0.812709236
b2396	alaX	tRNA-Ala(GGC)	-0.264687023	6.099106861	0.275434992	0.427733201
b2071	yegJ	DUF2314 domain-containing protein YegJ	-0.264993415	3.149600025	0.774217217	0.864702507
b2183	rsuA	16S rRNA pseudouridine516 synthase	-0.265067637	8.188335764	0.066183847	0.144297127
b2588	rrfG	5S ribosomal RNA	-0.265658884	8.105683024	0.160544591	0.289123789
b0744	valT	tRNA-Val(UAC)	-0.266105081	7.220010443	0.071546796	0.153428762
b3497	rsmJ	16S rRNA m2G1516 methyltransferase	-0.266318359	7.221096282	0.089881173	0.184882554
b1890	motA	motility protein A	-0.266463606	4.329110338	0.523456378	0.671170628
b4039	ubiC	CHORPYRLY-MONOMER	-0.266735568	7.246691876	0.127711341	0.242097712
b4264	idnR	DNA-binding transcriptional dual regulator IdnR	-0.267699061	6.473484202	0.18290609	0.31792535
b1810	yoaC	DUF1889 domain-containing protein YoaC	-0.26838861	7.379198475	0.070707143	0.152216641
b0420	dxs	1-deoxy-D-xylulose-5-phosphate synthase	-0.268423277	9.054647775	0.087027052	0.180048938
b0624	crcB	F- channel	-0.268463856	7.005556839	0.097829462	0.197312505
b1187	fadR	DNA-binding transcriptional dual regulator FadR	-0.268752067	7.858825704	0.057744595	0.129215603
b1277	ribA	GTP cyclohydrolase 2	-0.26890085	7.67199817	0.040026061	0.096724171
b0956	matP	macrodomain Ter protein	-0.270323762	6.590181883	0.164271435	0.294144711
b0745	lysW	tRNA-Lys(UUU)	-0.270819632	7.197390169	0.103157555	0.205338415
b4349	hsdM	type I restriction enzyme EcoKI methyltransferase component	-0.271131856	8.144902486	0.107351026	0.211681455
b0834	dgcI	putative diguanylate cyclase DgcI	-0.272224992	7.299007295	0.078318966	0.165049276
b0328	yahN	putative amino acid exporter	-0.272461239	5.534292245	0.406033852	0.566165284
b4719	sdsN	small regulatory RNA SdsN	-0.27252876	5.983909864	0.252689615	0.401883782
b1097	mltG	endolytic murein transglycosylase	-0.27295682	7.235755002	0.132305007	0.248796457
b3700	recF	DNA repair protein RecF	-0.273612941	8.113468754	0.068798688	0.149187724
b2722	hycD	formate hydrogenlyase subunit HycD	-0.273733867	4.003194874	0.597664334	0.73256192
b0415	ribE	6,7-dimethyl-8-ribityllumazine synthase	-0.273901611	8.034917845	0.053413285	0.121744787
b0890	ftsK	cell division DNA translocase FtsK	-0.274790377	10.18232533	0.070712645	0.152216641
b1001	yccE	uncharacterized protein YccE	-0.275328306	6.030648403	0.335663177	0.493490819
b0577	ybdG	miniconductance mechanosensitive channel YbdG	-0.275392842	7.137836736	0.114476247	0.222746862
b3665	adeD	adenine deaminase	-0.27617327	6.680609317	0.12148187	0.233496933
b3924	fpr	flavodoxinferredoxin-NADP+	-0.276173799	9.484769875	0.174915415	0.307669943
b3738	atpB	ATP synthase Fsubosub complex subunit a	-0.277056594	9.163901882	0.092164069	0.188207383
b1608	rstA	Phosphorylated DNA-binding transcriptional regulator RstA	-0.277426	8.46976007	0.063889817	0.140217258
b2511	der	50S ribosomal subunit stability factor	-0.277786725	9.126904474	0.091157017	0.18675556
b1634	dtpA	dipeptidetripeptide:H+	-0.278626929	8.261119413	0.099541607	0.199601617
b4040	ubiA	4OHBENZOATE-OCTAPRENYLTRANSFER-MONOMER	-0.278659012	7.786012639	0.039585092	0.096021046
b2175	mepS	peptidoglycan DD-endopeptidasepeptidoglycan LD-carboxypeptidase	-0.27927652	8.694357531	0.090607819	0.185918928
b3722	bglF	β-glucoside specific PTS enzyme II BglG kinase	-0.279507813	7.089094219	0.095374404	0.192977998
b1424	opgD	glucan biosynthesis protein D	-0.279544061	8.052765609	0.049526129	0.114720808
b0301	rclC	reactive chlorine species resistance protein C	-0.279994899	5.160323022	0.487061263	0.640373876
b2467	nudK	GDP-mannose hydrolase	-0.280402402	7.242848481	0.07399435	0.157782652
b1828	yebQ	putative transporter YebQ	-0.280550964	5.67915074	0.276489161	0.428766999
b0606	ahpF	alkyl hydroperoxide reductase, AhpF component	-0.281746772	9.96835171	0.071553753	0.153428762
b3756	rrsC	16S ribosomal RNA	-0.282411046	10.58642969	0.129147209	0.24460064
b4201	priB	primosomal replication protein N	-0.282490588	8.304891934	0.100436572	0.20100973

b0779	uvrB	excision nuclease subunit B	-0.283759048	9.335096153	0.075476962	0.160226505
b3242	aaeX	DUF1656 domain-containing protein AaeX	-0.283778654	7.068397767	0.220726328	0.364029412
b3911	cpxA	sensory histidine kinase CpxA - phosphorylated	-0.283991065	9.562461808	0.152050624	0.277398142
b1230	tyrV	tRNA-Tyr(GUA)	-0.284163065	5.669348075	0.265871414	0.418027882
b0399	phoB	Phosphorylated DNA-binding transcriptional dual regulator PhoB	-0.284368571	6.505169392	0.171207897	0.30308054
b1291	sapD	putrescine ABC exporter ATP binding protein SapD	-0.284832723	7.325468635	0.053722745	0.122197794
b1911	glyW	tRNA-Gly(GCC)	-0.285082768	6.944867232	0.110389136	0.216223617
b1893	insB-5	IS1 protein InsB	-0.285555828	7.436258726	0.046608707	0.109281701
b0746	valZ	tRNA-Val(UAC)	-0.285841673	7.133214074	0.061399364	0.135946936
b0275	insA-3	IS1 protein InsA	-0.286116237	7.120993034	0.057685793	0.12914948
b0614	citX	G6340-MONOMER	-0.286781445	4.556986753	0.425689249	0.584577927
b2949	yqgF	ribonuclease H-like domain containing nuclease	-0.287523454	7.100833339	0.05444985	0.123430397
b4606	ypfM	uncharacterized protein YpfM	-0.287822412	6.621938923	0.139657894	0.259071262
b3218	insH-10	IS5 transposase and trans-activator	-0.287995252	6.939331218	0.289780793	0.443460035
b2275	yfbP	uncharacterized protein YfbP	-0.288802864	6.067254672	0.176285234	0.309373241
b4461	yfjD	putative inner membrane protein	-0.289356862	8.185957679	0.046787573	0.109642852
b2499	purM	phosphoribosylformylglycinamide cyclo-ligase	-0.289540987	6.490880465	0.217037416	0.35901843
b0288	ykgJ	putative zinc- or iron-chelating domain-containing protein YkgJ	-0.289556944	6.503748872	0.215228411	0.35687326
b4067	actP	acetateglycolate:cation symporter	-0.290095285	5.517044724	0.263200381	0.414270831
b1345	intR	Rac prophage; putative integrase	-0.290364931	5.951308929	0.202110669	0.341657262
b2342	fadI	3-ketoacyl-CoA thiolase FadI	-0.290733032	6.457363088	0.18421714	0.319324174
b2807	ygdD	conserved inner membrane protein YgdD	-0.290951527	6.626042643	0.112442148	0.21970322
b0409	secF	Sec translocon accessory complex subunit SecF	-0.29095908	8.723914628	0.073245038	0.156523158
b0048	folA	dihydrofolate reductase	-0.291631367	7.794156383	0.042225975	0.100979469
b4476	gntU	low-affinity gluconate transporter	-0.293612659	6.111831309	0.185933844	0.321582424
b1926	fliT	flagellar biosynthesis protein FliT	-0.293742523	4.064443517	0.479372327	0.633092678
b2812	tcdA	tRNA threonylcarbamoyladenosine dehydratase	-0.293999303	7.495419488	0.042500827	0.101407523
b3261	fis	DNA-binding transcriptional dual regulator Fis	-0.294604801	8.21038174	0.054792618	0.123992522
b1330	ynaI	small conductance mechanosensitive channel YnaI	-0.294801498	6.908102447	0.065613091	0.143335871
b3460	livJ	branched chain amino acidphenylalanine ABC transporter periplasmic bi	-0.295385913	6.146473174	0.159405485	0.287960399
b4133	cadC	DNA-binding transcriptional activator CadC	-0.295866297	7.493621474	0.164211897	0.294144711
b4712	agrA	small RNA AgrA	-0.29625825	4.977142848	0.467327412	0.622962115
b1098	tmk	DTMPKI-MONOMER	-0.29641495	7.327903182	0.051393343	0.118239504
b4464	ghxQ	guaninehypoxanthine transporter GhxQ	-0.296643567	5.490575362	0.38532431	0.546637241
b3224	nanT	N-acetylneuraminate:H+	-0.296644568	6.744265603	0.293586784	0.446806498
b2839	lysR	DNA-binding transcriptional dual regulator LysR	-0.297689759	5.916608741	0.347576681	0.507121958
b3273	thrV	tRNA-Thr(GGU)	-0.298108953	8.411790633	0.140308461	0.260168775
b0954	fabA	β-hydroxyacyl-acyl carrier protein dehydrataseisomerase	-0.298306517	8.349627812	0.047255556	0.110622099
b2423	cysW	sulfatethiosulfate ABC transporter inner membrane subunit CysW	-0.298505678	8.725141244	0.061231716	0.135643766
b2778	ygcG	protein YgcG	-0.298645768	4.432145442	0.557185	0.697073328
b4034	malE	maltose ABC transporter periplasmic binding protein	-0.299216587	5.22497516	0.309227216	0.464841048
b1526	yneJ	putative LysR-type DNA-binding transcriptional regulator YneJ	-0.300002387	5.817328317	0.245317495	0.393989357
b4105	phnD	phosphonate ABC transporter periplasmic binding protein	-0.300135509	4.057161403	0.552345538	0.693179519
b0097	secM	SecA translation regulator	-0.300495119	7.869556747	0.022278132	0.059359054
b2693	argY	tRNA-Arg(ACG)	-0.300634662	7.498437299	0.066863181	0.145634408
b2611	ypjD	cytochrome c assembly family protein	-0.300729013	7.450791236	0.086021912	0.178471212
b4446	sibC	small RNA SibC	-0.300823209	6.410429895	0.188325025	0.325041041

b3358	yhfK	putative transporter YhfK	-0.30117651	8.292815146	0.038625711	0.094268941
b3659	setC	putative arabinose exporter	-0.301356754	5.045376492	0.363222813	0.524919385
b3492	yhiN	putative oxidoreductase YhiN	-0.302460208	7.293865334	0.061903441	0.136857132
b2956	yggM	DUF1202 domain-containing protein YggM	-0.302991358	6.149629859	0.221620555	0.364958877
b2833	ygdR	DUF903 domain-containing lipoprotein YgdR	-0.303228545	7.385659001	0.036091959	0.08946996
b3113	tdcF	putative enamineimine deaminase	-0.303981471	4.905678959	0.541272639	0.684929407
b0175	cdsA	CDP-diglyceride synthetase	-0.304239783	8.287041011	0.045183242	0.106847356
b2229	yfaT	DUF1175 domain-containing protein YfaT	-0.304472791	3.486774987	0.707231911	0.812709236
b4032	malG	maltose ABC transporter membrane subunit MalG	-0.304783394	4.949389757	0.33000155	0.486950817
b3182	dacB	peptidoglycan DD-endopeptidase DacB	-0.304843607	7.539519822	0.117663935	0.227944832
b0891	lolA	outer membrane lipoprotein carrier protein	-0.305736641	8.23912381	0.05498966	0.124374666
b0408	secD	Sec translocon accessory complex subunit SecD	-0.306366842	9.559971745	0.050183517	0.115878781
b1321	ycjX	DUF463 domain-containing protein YcjX	-0.306412679	7.335465559	0.051257082	0.118049044
b2406	xapB	XAPB-MONOMER	-0.306516912	5.381247971	0.326373593	0.483861455
b1214	ychA	transglutaminase-likeTPR repeat-containing protein	-0.306864242	8.432503433	0.044920385	0.106282689
b0148	hrpB	putative ATP-dependent RNA helicase HrpB	-0.308541058	8.547391309	0.05361789	0.122022156
b2403	valY	tRNA-Val(UAC)	-0.309279467	7.324656359	0.036158241	0.089583971
b1042	csgA	curlin, major subunit	-0.309472871	3.713348746	0.644613519	0.769804892
b3822	recQ	ATP-dependent DNA helicase RecQ	-0.309534894	9.829016435	0.07528685	0.159971742
b0594	entE	2,3-dihydroxybenzoate-AMP ligase	-0.310195098	4.177125142	0.456313223	0.613212915
b4369	leuP	tRNA-Leu(CAG)	-0.31034797	7.022563972	0.059688928	0.132759001
b1666	valW	tRNA-Val(GAC)	-0.31125803	5.938743015	0.204780774	0.344947394
b3614	yibQ	divergent polysaccharide deacetylase domain-containing protein YibQ	-0.312087015	7.902757197	0.03194957	0.081067444
b1370	insH-5	Rac prophage; IS5 transposase and trans-activator	-0.312377217	6.362796577	0.124082383	0.236947975
b1644	ydhJ	putative membrane fusion protein YdhJ	-0.31449574	6.009123737	0.205266992	0.345502771
b1516	lsrB	Autoinducer-2 ABC transporter periplasmic binding protein	-0.317337981	4.334328428	0.424051598	0.583734012
b2689	yqaA	DedA family protein YqaA	-0.317959209	7.412892546	0.052684475	0.12064417
b0066	thiQ	thiamin ABC transporter ATP binding subunit	-0.318652132	5.915244618	0.231333338	0.377016126
b0381	ddlA	D-alanine—D-alanine ligase A	-0.320245526	8.503960945	0.048517937	0.112740365
b2313	cvpA	colicin V production protein	-0.321035499	6.543696013	0.102607561	0.204612638
b2428	murQ	N-acetylmuramic acid 6-phosphate etherase	-0.321274219	5.894455469	0.205499181	0.345630051
b1609	rstB	sensory histidine kinase RstB - phosphorylated	-0.321439157	8.789761004	0.039150398	0.095391285
b4011	yjaA	stress response protein	-0.322809393	6.171223471	0.232622131	0.378431714
b2445	yffN	CPZ-55 prophage; uncharacterized protein YffN	-0.322933469	4.167136231	0.427198465	0.586468041
b3080	ygjK	glycoside hydrolase	-0.32336017	5.593861326	0.377335584	0.538093218
b4441	glmY	small regulatory RNA GlmY	-0.324304408	6.363099649	0.173466715	0.305852854
b4415	hokE	protein HokE	-0.324566522	3.4076166	0.666698187	0.785066739
b1635	gstA	glutathione S-transferase GstA	-0.325059652	6.884305249	0.054059564	0.122774164
b1102	fhuE	ferric coprogenferric rhodotorulic acid outer membrane transporter	-0.325599185	5.974705736	0.179134499	0.313062549
b0141	yadN	putative fimbrial protein YadN	-0.32593795	4.827888648	0.428149124	0.58759042
b3251	mreB	dynamic cytoskeletal protein MreB	-0.32597639	9.440264802	0.039822135	0.096336836
b0612	citT	B0612-MONOMER	-0.326220339	4.609442236	0.374285634	0.535647026
b4311	nanC	N-acetylneuraminic acid outer membrane channel	-0.326424037	2.634627738	0.591368984	0.726522567
b1879	flhA	flagellar biosynthesis protein FlhA	-0.326428735	5.430011094	0.279852548	0.431859141
b0008	talB	transaldolase B	-0.327012648	9.420663893	0.046492494	0.109125127
b3174	leuU	tRNA-Leu(GAG)	-0.327125746	6.919396506	0.193321606	0.33171974
b1920	tcyJ	cystine ABC transporter periplasmic binding protein	-0.327827926	8.93835731	0.042361191	0.101149085

b3010	yqhC	DNA-binding transcriptional activator YqhC	-0.328319883	6.73555424	0.085561933	0.177600345
b4757	ysdD	protein YsdD	-0.328879577	4.761038389	0.357675698	0.518600396
b0547	ybcN	DLP12 prophage; DNA base-flipping protein	-0.329096575	2.758643388	0.746213488	0.841515338
b3173	yhbX	putative hydrolase YhbX	-0.329459685	6.825226001	0.201089151	0.340678666
b3272	rrfF	5S ribosomal RNA	-0.329642306	8.403675079	0.116828897	0.226625474
b2690	yqaB	fructose-1-phosphatase	-0.330003502	7.53467823	0.031354205	0.07978606
b4047	yjbL	uncharacterized protein YjbL	-0.330447943	4.942994668	0.464852116	0.620974915
b3671	ilvB	acetohydroxy acid synthase I subunit IlvB	-0.330476703	7.268820434	0.046155283	0.108506696
b2088	insE-5	IS3 element protein InsE	-0.330683117	6.370536351	0.103623955	0.205891395
b0729	sucD	succinyl-CoA synthetase subunit α	-0.331380781	9.045827724	0.055405777	0.125209545
b1537	ydeJ	PF02464 family protein YdeJ	-0.33141733	6.061376222	0.131877767	0.248290124
b3546	eptB	Kdo2-lipid A phosphoethanolamine 7''-transferase	-0.332677465	8.260100573	0.035007551	0.087326375
b2186	yejK	nucleoid-associated protein YejK	-0.333253706	8.230864803	0.038590876	0.094236016
b0540	insE-3	DLP12 prophage; IS3 element protein InsE	-0.333612472	6.426523115	0.099552154	0.199601617
b2786	barA	sensory histidine kinase BarA - his816 phosphorylated	-0.334209716	8.067205524	0.03174105	0.080652556
b4290	fecB	ferric citrate ABC transporter periplasmic binding protein	-0.334398133	5.84170964	0.160708857	0.289131868
b1116	lolC	lipoprotein release complex - inner membrane subunit	-0.335635564	8.004775235	0.013119762	0.038233498
b0651	rihA	pyrimidine-specific ribonucleoside hydrolase RihA	-0.336582913	6.889924541	0.068433304	0.148541316
b4724	ynfQ	Qin prophage; protein YnfQ	-0.337147802	4.642288322	0.43087948	0.589870667
b1495	yddB	putative TonB-dependent receptor	-0.337627252	4.463809139	0.517874889	0.667567192
b3188	sfsB	putative transcriptional regulator SfsB	-0.337722186	7.086380202	0.144105475	0.265980633
b3545	proK	tRNA-Pro(CGG)	-0.337734446	5.196918759	0.370653933	0.531828766
b0444	queC	7-cyano-7-deazaguanine synthase	-0.338497993	7.868836814	0.008790757	0.027121729
b3634	coaD	pantetheine-phosphate adenylyltransferase	-0.338879325	7.183095553	0.026969207	0.070095819
b1864	yebC	putative transcriptional regulator YebC	-0.339386652	8.609385986	0.03998186	0.096670269
b2569	lepA	elongation factor 4	-0.339844104	9.509510318	0.03560973	0.088473247
b3716	cbrB	putative inner membrane protein	-0.340174213	6.819113057	0.036257388	0.089728905
b2534	yfhR	putative peptidase	-0.341595085	5.518464615	0.237158161	0.384372576
b3151	yraQ	permease family protein YraQ	-0.341780924	7.715715664	0.014935236	0.042114861
b1385	feaB	phenylacetaldehyde dehydrogenase	-0.34192587	6.550164055	0.070622625	0.152171249
b0720	gltA	citrate synthase	-0.341974353	8.932547588	0.059345351	0.132261345
b4670	yjeV	uncharacterized protein YjeV	-0.342212781	4.833316911	0.374635627	0.535898826
b0386	proC	pyrroline-5-carboxylate reductase	-0.34303272	6.967671117	0.044091553	0.104658176
b1862	yebB	putative papain-like amidase YebB	-0.343649928	5.456037368	0.217605163	0.359688055
b3743	asnC	DNA-binding transcriptional dual regulator AsnC	-0.343864657	6.614045161	0.086628309	0.179392113
b4086	alsC	D-allose ABC transporter membrane subunit	-0.343932009	5.598951289	0.226752707	0.371673932
b2750	cysC	adenylyl-sulfate kinase	-0.344693658	8.470548964	0.030835935	0.078785099
b0813	rhtA	L-threonineL-homoserine exporter	-0.34510748	5.052390873	0.229536397	0.375195554
b0120	speD	S-adenosylmethionine decarboxylase proenzyme	-0.345186416	7.676940199	0.021171047	0.056820774
b2401	valU	tRNA-Val(UAC)	-0.345834269	5.676072851	0.181084386	0.315753382
b0922	mukF	chromosome partitioning protein MukF	-0.346089777	8.618552763	0.044290299	0.10490433
b3190	ibaG	acid stress protein IbaG	-0.347469618	7.583102827	0.021136649	0.056762958
b3803	hemX	PF04375 family protein HemX	-0.348200846	9.370028074	0.077594991	0.163758071
b1642	slyA	DNA-binding transcriptional dual regulator SlyA	-0.348788601	8.066392729	0.010897246	0.032578433
b4687	shoB	toxic peptide ShoB	-0.35022204	6.199282929	0.238745511	0.385961711
b1929	yedE	putative selenium transporter YedE	-0.350337319	7.374006773	0.047316285	0.110653567
b3225	nanA	N-acetylneuraminate lyase	-0.350899247	6.853762579	0.209451414	0.350456647

b3049	glgS	surface composition regulator	-0.351007693	6.603035522	0.079781098	0.16757067
b1286	rnb	RNase II	-0.351189032	9.54104409	0.032939497	0.083015879
b0969	tusE	sulfur transfer protein TusE	-0.353210785	6.495161874	0.05058932	0.116693755
b0400	phoR	sensory histidine kinase PhoR	-0.35375831	6.923351743	0.040056744	0.096745364
b0523	purE	N5	-0.354096882	5.622986168	0.215255604	0.35687326
b4723	ymcF	protein YmcF	-0.354605237	6.65898849	0.083792477	0.174749073
b1216	chaA	Na+	-0.35492264	6.06781816	0.156311397	0.283182117
b3398	igaA	inner membrane protein - inhibits the Rcs signaling pathway	-0.354972948	7.955702032	0.007514496	0.023833693
b4197	ulaE	L-ribulose-5-phosphate 3-epimerase UlaE	-0.355020017	4.981733614	0.296391259	0.449679522
b0747	lysY	tRNA-Lys(UUU)	-0.355097709	6.824779254	0.034071715	0.085421136
b4198	ulaF	L-ribulose-5-phosphate 4-epimerase UlaF	-0.356020085	4.716335069	0.347423849	0.507086393
b1012	rutA	pyrimidine oxygenase	-0.356042506	2.056598189	0.852811476	0.912788886
b0913	ycaI	conserved inner membrane protein YcaI	-0.356595144	6.935782825	0.034900711	0.087203225
b3621	waaC	ADP-heptose:LPS heptosyltransferase 1	-0.356914156	8.316636433	0.013278495	0.038594176
b1199	dhaL	dihydroxyacetone kinase subunit L	-0.357475699	8.169399998	0.023419834	0.062063967
b2700	pncC	NMN aminohydrolase	-0.35783916	6.98353013	0.078629524	0.165545707
b0056	yabP	putative uncharacterized protein YabP	-0.358038194	7.040397599	0.021755946	0.05814316
b4400	creD	putative inner membrane protein	-0.358118621	5.606748822	0.281936715	0.434013457
b1983	yeeN	putative transcriptional regulator YeeN	-0.358209751	8.719249527	0.044189193	0.104833577
b2887	ygfT	fused putative oxidoreductase, Fe-S subunit and nucleotide-binding sub	-0.358356441	5.314422197	0.364216422	0.525323588
b4370	leuQ	tRNA-Leu(CAG)	-0.358390036	6.760308622	0.047669146	0.111236406
b3957	argE	acetylornithine deacetylase	-0.358724236	9.049650349	0.072153768	0.154640236
b2101	yegW	putative DNA-binding transcriptional regulator YegW	-0.359072896	6.421240417	0.058116149	0.129849593
b3343	tusB	sulfurtransferase complex subunit TusB	-0.360671813	7.051776152	0.039741566	0.096234484
b2173	yeiR	zinc-binding GTPase YeiR	-0.360818673	7.014035749	0.041416446	0.099485097
b2413	cysZ	EG10003-MONOMER	-0.360893878	7.243869177	0.035918102	0.089089
b1036	ycdZ	putative inner membrane protein	-0.361131439	7.195649374	0.019283684	0.052456848
b3359	argD	N-acetylornithine aminotransferase	-0.362185286	7.037582424	0.032875184	0.082939392
b0742	cpoB	cell division coordinator CpoB	-0.36327938	9.052281481	0.037479251	0.09218434
b1432	insQ	putative insertion element transposase InsQ	-0.36389112	7.341174134	0.010381354	0.031342065
b1292	sapC	putrescine ABC exporter membrane protein SapC	-0.364173561	6.462337357	0.050905908	0.117362706
b4134	pheU	tRNA-Phe(GAA)	-0.364616807	5.87869561	0.153983566	0.280114316
b1164	ycgZ	putative two-component system connector protein YcgZ	-0.365322341	5.830897633	0.195565035	0.334033102
b3717	cbrC	UPF0167 family colicin E2 tolerance protein CbrC	-0.365373362	6.662614849	0.042662907	0.101704499
b3467	yhhM	DUF2500 domain-containing protein YhhM	-0.365978729	6.314746658	0.076980623	0.162616962
b1263	trpD	anthranilate synthase subunit TrpD	-0.366175751	5.423207236	0.241283755	0.389494617
b1870	cmoA	carboxy-S-adenosyl-L-methionine synthase	-0.367054258	7.591676879	0.006896338	0.022063284
b3742	mioC	flavoprotein MioC	-0.369056849	7.974660203	0.005430821	0.017973818
b1915	yecF	DUF2594 domain-containing protein YecF	-0.370030561	5.369605532	0.168892342	0.300426949
b1027	insE-4	IS3 element protein InsE	-0.370347692	4.920272536	0.280650859	0.432486402
b4181	yjfI	DUF2170 domain-containing protein YjfI	-0.370870419	3.303026752	0.511712708	0.662137047
b0390	aroM	protein AroM	-0.371539663	6.822097249	0.051748582	0.118870962
b1612	fumA	fumarase A	-0.371622114	8.809165916	0.045439375	0.107190903
b4102	phnF	putative transcriptional regulator PhnF	-0.371839164	4.205862791	0.329806223	0.486950817
b0748	lysZ	tRNA-Lys(UUU)	-0.372160484	6.690300826	0.051463854	0.118340061
b4510	rzoD	DLP12 prophage; putative prophage lysis lipoprotein RzoD	-0.373125673	3.151622655	0.495892775	0.647358546
b4516	insA-4	IS1 protein InsA	-0.37337335	5.998215712	0.091571502	0.18734392

b0460	hha	haemolysin expression modulating protein	-0.374538161	7.589085317	0.004133385	0.014157405
b3650	spoT	bifunctional (p)ppGpp synthasehydrolase SpoT	-0.375108913	9.671804003	0.012542287	0.036842447
b0798	ybiA	N-glycosidase YbiA	-0.37620223	5.34320776	0.214997887	0.356847997
b3651	trmH	EG10967-MONOMER	-0.376721775	7.727974367	0.005735597	0.018841264
b1290	sapF	putrescine ABC exporter ATP binding protein SapF	-0.377160569	6.422675498	0.073949216	0.157782652
b0511	ybbW	putative allantoin transporter	-0.377977757	2.182020803	0.850543899	0.912326751
b1350	recE	Rac prophage; exonuclease VIII, ds DNA exonuclease, 5' -- 3' specific	-0.378582233	5.641012231	0.178420956	0.312095294
b0265	insA-2	IS1 protein InsA	-0.378851569	7.078888012	0.018616184	0.050955022
b0298	insE-1	IS3 element protein InsE	-0.379048921	6.573595194	0.032047897	0.081223574
b4715	ytiA	uncharacterized protein YtiA	-0.379118427	4.567769291	0.310737269	0.466475703
b4145	yjeJ	protein YjeJ	-0.379454646	6.406679386	0.054203462	0.123037678
b3260	dusB	tRNA-dihydrouridine synthase B	-0.379875838	9.159707983	0.013736948	0.039613734
b4734	yldA	protein YldA	-0.379908068	4.102420499	0.413664685	0.573056035
b4567	yjjZ	DUF1435 domain-containing protein YjjZ	-0.380844169	5.074456208	0.249133257	0.398090239
b0909	ycaL	periplasmic protease YcaL	-0.381255903	4.711972568	0.250086758	0.399036154
b1763	topB	DNA topoisomerase III	-0.381955879	9.097889319	0.014720522	0.041741235
b1649	nemR	DNA-binding transcriptional repressor NemR	-0.382192057	6.486191746	0.0603546	0.133969612
b2920	scpC	G7517-MONOMER	-0.382344444	6.15474669	0.076547331	0.162011728
b2587	kgtP	α-ketoglutarate:H+ symporter	-0.382712507	9.546875013	0.045449914	0.107190903
b0958	sulA	cell division inhibitor SulA	-0.38336696	6.968547763	0.021772409	0.058151956
b0588	fepC	ferric enterobactin ABC transporter ATP binding subunit	-0.383488376	3.720394182	0.472720687	0.627808796
b0634	mrdB	SEDS family protein MrdB	-0.384582696	8.015663944	0.009168728	0.028130601
b0601	ybdM	ParB-like nuclease domain-containing protein YbdM	-0.385205543	5.203376305	0.215863007	0.357557838
b1711	btuC	vitamin Bsub12sub ABC transporter membrane subunit	-0.385616724	6.754612426	0.031981454	0.081101735
b1044	ymdA	uncharacterized protein YmdA	-0.385646181	4.132966866	0.353693566	0.514174803
b4709	insA9	IS1 repressor TnpA	-0.386289769	7.333802261	0.013839733	0.039832086
b0758	galT	galactose-1-phosphate uridylyltransferase	-0.386767848	7.267138404	0.015410648	0.043281176
b1705	ydiE	PF10636 family protein YdiE	-0.38815438	5.562164433	0.1484577	0.272193001
b3710	mdtL	efflux pump MdtL	-0.388277892	6.134767605	0.093250403	0.189723746
b4099	phnI	carbon-phosphorus lyase core complex subunit PhnI	-0.388750524	4.164860533	0.341257723	0.500050729
b1049	opgH	osmoregulated periplasmic glucans (OPGs) biosynthesis protein H	-0.389018739	9.525359854	0.022551313	0.060014495
b3293	yhdN	DUF1992 domain-containing protein YhdN	-0.389117554	7.061541811	0.018434163	0.050491171
b1925	fliS	flagellar biosynthesis protein FliS	-0.389284575	4.168758179	0.317551311	0.474446375
b0566	envY	DNA-binding transcriptional activator EnvY	-0.389791934	4.311921288	0.380921752	0.542506302
b2900	yqfB	PUA-like domain-containing protein YqfB	-0.390223483	7.372954363	0.017781233	0.048942734
b2568	lepB	signal peptidase I	-0.390293789	8.579064756	0.016688417	0.046368383
b3434	yhgN	putative inner membrane protein	-0.390751833	6.502594152	0.064204249	0.140745661
b2723	hycC	formate hydrogenlyase subunit HycC	-0.390846539	4.100148229	0.37517966	0.535898826
b3527	yhjJ	peptidase M16 family protein YhjJ	-0.391546594	8.590785531	0.012418694	0.03657674
b0211	mltD	membrane-bound lytic murein transglycosylase D	-0.391559181	8.586897232	0.01078973	0.03233989
b2155	cirA	EG10155-MONOMER	-0.391774017	6.086394603	0.200824035	0.340360121
b1048	opgG	osmoregulated periplasmic glucans (OPGs) biosynthesis protein G	-0.391870594	9.145024815	0.01760496	0.048548344
b2421	cysM	cysteine synthase B	-0.392157379	9.024856574	0.013542523	0.039232439
b4420	rdlA	putative antisense RNA regulator of LdrA	-0.392970818	5.455950385	0.194651425	0.333428456
b4368	leuV	tRNA-Leu(CAG)	-0.394079109	6.322191011	0.046454462	0.109093857
b1924	fliD	flagellar filament capping protein	-0.394337609	4.827717577	0.309706955	0.465403746
b0410	yajD	HNH nuclease family protein YajD	-0.395561826	7.128686222	0.014934616	0.042114861

b1494	pqqL	putative zinc peptidase	-0.395802283	5.687177678	0.177691052	0.310996654
b4688	ykgS	CP4-6 prophage; protein YkgS	-0.396475694	6.246911329	0.114100251	0.22221112
b2759	casB	type I-E CRISPR system Cascade subunit CasB	-0.396555941	4.413562365	0.288161641	0.441288118
b3596	yibG	tetratricopeptide-like domain-containing protein YibG	-0.396775235	4.980025464	0.290312001	0.443811455
b2324	mnmC	fused 5-methylaminomethyl-2-thiouridine-forming methyltransferase a	-0.396788977	8.478573322	0.023044963	0.061180705
b0456	ybaA	DUF1428 domain-containing protein YbaA	-0.397243049	5.076316148	0.184085087	0.319254882
b1238	tdk	thymidinedeoxyuridine kinase	-0.397527972	7.509904602	0.013581008	0.039318131
b1242	ychE	putative inner membrane protein	-0.397571753	6.789885368	0.023823582	0.06302044
b1237	hns	DNA-binding transcriptional dual regulator H-NS	-0.397895718	8.232859818	0.009752444	0.029694511
b0809	glnQ	L-glutamine ABC transporter ATP binding subunit	-0.398552932	7.879039697	0.011871754	0.035177043
b2493	yfgO	putative transporter YfgO	-0.398775529	7.287345931	0.014792217	0.041837052
b0248	yafX	CP4-6 prophage; protein YafX	-0.399015707	6.5464504	0.038022952	0.093106675
b0310	ykgH	uncharacterized protein YkgH	-0.399550375	5.534114575	0.205745086	0.345911864
b1024	pgaA	partially deacetylated poly-β-1,6-N-acetyl-D-glucosamine outer m	-0.39980075	5.48522957	0.230350935	0.376248383
b3606	trmL	tRNA (cytidineuridine-2'-O	-0.400095199	6.652102318	0.02535113	0.066424474
b2234	nrdA	ribonucleoside-diphosphate reductase 1 subunit α	-0.400166347	9.198729329	0.066986425	0.1458309
b2349	intS	CPS-53 (KpLE1) prophage; prophage CPS-53 integrase	-0.400791946	6.778737666	0.048105808	0.112136822
b1100	ycfH	putative metal-dependent hydrolase YcfH	-0.400981879	7.955945114	0.002677669	0.009739627
b0010	satP	acetatesuccinate:H+	-0.401744393	6.942001413	0.020663676	0.05569605
b4722	idlP	iraD leader peptide	-0.402380539	6.705976324	0.025509306	0.06679928
b1212	prmC	EG12424-MONOMER	-0.403503515	7.523518949	0.004967079	0.016525739
b0470	dnaX	DNA polymerase III subunit γ	-0.404484762	9.02117553	0.011752068	0.034916139
b2620	smpB	SsrA-binding protein	-0.404712339	7.758863595	0.003336991	0.01179569
b0693	speF	ornithine decarboxylase, degradative	-0.405910441	5.724459794	0.108668078	0.21353119
b1762	ynjI	DUF1266 domain-containing protein YnjI	-0.406094166	5.931493779	0.146673615	0.26970596
b2652	ileY	tRNA-Ile(CAU)	-0.406184524	5.380391529	0.250895027	0.399892254
b4745	yncO	protein YncO	-0.406626517	5.313920568	0.233983159	0.380351858
b0668	glnW	tRNA-Gln(UUG)	-0.406814828	7.090765975	0.008399737	0.026097705
b1742	ves	HutD family protein Ves	-0.40693293	4.497327674	0.255115746	0.404574719
b0060	polB	DNA polymerase II	-0.407240731	8.028213348	0.005642213	0.018562125
b3257	yhdT	DUF997 domain-containing protein YhdT	-0.407639783	6.900050644	0.160627032	0.289123789
b2500	purN	phosphoribosylglycinamide formyltransferase 1	-0.407735597	6.646928967	0.033603929	0.084455387
b3725	pstB	phosphate ABC transporter ATP binding subunit	-0.407756489	8.088436244	0.003934169	0.013633718
b0009	mog	molybdopterin adenylyltransferase	-0.407954709	7.666003989	0.001620136	0.006263485
b2616	recN	DNA repair protein RecN	-0.407970015	8.38520388	0.013482631	0.03908458
b1347	rcbA	Rac prophage; double-strand break reduction protein	-0.408151323	3.226118907	0.586018518	0.722096499
b3170	rimP	ribosome maturation factor RimP	-0.408833281	8.294161324	0.01089383	0.032578433
b1331	insH-4	IS5 transposase and trans-activator	-0.408866615	9.440719312	0.020122813	0.054371004
b3582	sgbU	putative L-xylulose 5-phosphate 3-epimerase	-0.409256248	4.580653487	0.271227094	0.423127697
b0849	grxA	oxidized glutaredoxin 1	-0.40928414	7.500150413	0.005983201	0.019495079
b0730	mngR	DNA-binding transcriptional repressor MngR	-0.409507362	6.211756627	0.063290021	0.139294837
b1134	nudJ	phosphatase NudJ	-0.409683211	6.433846952	0.04218937	0.100957218
b3666	uhpT	hexose-6-phosphate:phosphate antiporter	-0.409819208	5.627358597	0.150762662	0.275857001
b0442	ybaV	helix-hairpin-helix 3 family protein	-0.410074402	5.434128921	0.161155871	0.289582081
b1861	ruvA	Holliday junction branch migration complex subunit RuvA	-0.410289715	7.276454846	0.011411646	0.033950415
b2392	mntH	Mn2+	-0.411607058	7.665935987	0.003251843	0.011531636
b2982	insH-9	IS5 transposase and trans-activator	-0.411758041	9.473088857	0.018957635	0.05182536

b4333	yjiK	uncharacterized protein YjiK	-0.412950267	5.82993701	0.101075167	0.202013066
b2174	lpxT	Kdosub2sub-lipid A phosphotransferase	-0.413129465	6.816793695	0.016082807	0.04496871
b1344	ttcA	tRNA cytosine32 2-sulfurtransferase TtcA	-0.413863506	7.919676621	0.00683214	0.021889621
b4675	yoaJ	uncharacterized protein YoaJ	-0.414119862	5.142106279	0.212468926	0.354382436
b0884	infA	translation initiation factor IF-1	-0.414810711	7.759657507	0.005455593	0.018028773
b2096	gatY	tagatose-1,6-bisphosphate aldolase 2 subunit GatY	-0.414904743	9.506149749	0.013900034	0.039979578
b0414	ribD	fused diaminohydroxyphosphoribosylaminopyrimidine deaminase 5-am	-0.416461432	8.576401213	0.008030208	0.025126414
b2828	lgt	EG12128-MONOMER	-0.417375956	7.910465913	0.001375485	0.005398013
b2114	metG	methionine—tRNA ligase	-0.418141133	8.902005138	0.012618169	0.036991512
b1663	mdtK	multidrug efflux pump MdtK	-0.418726898	7.50625422	0.004949047	0.016490598
b0174	ispU	undecaprenyl diphosphate synthase	-0.419448367	8.536160376	0.008248756	0.025719107
b2935	tktA	transketolase 1	-0.420971821	10.2288154	0.008921087	0.027427995
b4184	yjfL	DUF350 domain-containing inner membrane protein YjfL	-0.42100429	2.412246411	0.535320963	0.681120599
b3055	ygiM	putative signal transduction protein (SH3 domain)	-0.42160799	7.463495388	0.004839875	0.016212252
b4459	ryjA	small RNA RyjA	-0.421634625	6.286167915	0.069251928	0.149947645
b2800	fucA	L-fuculose-phosphate aldolase	-0.421865159	4.306365762	0.261902787	0.412475308
b0393	rdgC	nucleoid-associated protein RdgC	-0.421947638	7.820829442	0.00352596	0.012374503
b2718	hycH	formate hydrogenlyase assembly protein	-0.42213032	5.177487651	0.155498592	0.282056813
b0960	yccS	putative transporter YccS	-0.422314544	8.101673944	0.002201511	0.008237011
b2463	maeB	malate dehydrogenase	-0.422365216	9.144693011	0.014571196	0.041450921
b1205	ychH	stress-induced protein	-0.423187432	6.391212869	0.062963649	0.13878408
b4615	yibV	PF15596 family protein YibV	-0.424131	4.489613785	0.343159317	0.501837822
b1374	pinR	Rac prophage; putative site-specific recombinase	-0.424695102	4.722475762	0.223428188	0.367387505
b2619	ratA	ribosome association toxin RatA	-0.425167592	7.399165223	0.007638102	0.024173635
b0416	nusB	transcription antitermination protein NusB	-0.425325482	7.910500278	0.001088743	0.00438977
b4348	hsdS	type I restriction enzyme EcoKI specificity protein	-0.425390667	7.315644403	0.010771398	0.032306877
b1520	yneE	conserved inner membrane protein YneE	-0.425678231	7.041199674	0.01379668	0.039760015
b0461	tomB	protein that modulates Hha toxicity	-0.425712976	7.761199842	0.001080913	0.004366177
b3802	hemY	protein HemY	-0.426236465	9.351777373	0.039215423	0.09545226
b4711	insH21	IS5 transposase and trans-activator	-0.426514633	9.453369317	0.01529033	0.043025371
b4388	serB	PSERPHOSPHA-MONOMER	-0.426563905	7.278383933	0.026184907	0.068446633
b4280	yjhC	KpLE2 phage-like element; putative oxidoreductase YjhC	-0.428276861	7.351230805	0.031128999	0.079394769
b0383	phoA	alkaline phosphatase	-0.42839417	6.648456575	0.034870205	0.087203225
b1696	ydiP	putative DNA-binding transcriptional regulator YdiP	-0.428530043	5.520202777	0.117651757	0.227944832
b0397	sbcC	ATP dependent, structure specific DNA nuclease - SbcC subunit	-0.429113529	7.891690863	0.003358091	0.011859172
b4042	dgkA	diacylglycerol kinase	-0.429689513	6.742439859	0.019091983	0.052031548
b2492	focB	formate channel FocB	-0.430629143	6.176441702	0.04119747	0.099012973
b1818	manY	mannose-specific PTS enzyme IIC component	-0.430848599	8.553246931	0.005292859	0.017543523
b4136	dsbD	thiol-disulfide exchange protein DsbDsubreducedsub	-0.431553494	7.589833899	0.000818274	0.003437373
b1817	manX	mannose-specific PTS enzyme IIAB component	-0.432643846	8.899748547	0.007652991	0.024186081
b1255	yciC	putative inner membrane protein	-0.432706612	7.680365302	0.002009002	0.007580979
b4535	yniD	uncharacterized protein YniD	-0.432818872	4.613399652	0.214512687	0.356230517
b3126	garL	α-dehydro-β-deoxy-D-glucarate aldolase	-0.433121604	5.861746836	0.221558584	0.364958877
b0986	gfcB	lipoprotein GfcB	-0.433903683	3.826010629	0.345182111	0.504461774
b2921	ygfI	putative LysR-type DNA-binding transcriptional regulator YgfI	-0.434115658	6.620758309	0.026082505	0.068219349
b1186	nhaB	Na+:H+	-0.43414727	7.243403116	0.011952667	0.035369321
b1626	ydgK	conserved inner membrane protein YdgK	-0.434575072	7.377029611	0.001450995	0.005679206

b4428	hokB	toxin HokB	-0.434747212	5.93106139	0.052789652	0.120697211
b2692	argZ	tRNA-Arg(ACG)	-0.434858643	7.422779678	0.028877002	0.074426131
b1250	kch	K+ channel Kch	-0.434899023	8.327496632	0.003992406	0.013803034
b4078	yjcO	Sel1 repeat-containing protein YjcO	-0.435753218	6.86876572	0.010703282	0.032146251
b0413	nrdR	NrdR transcriptional repressor	-0.435789273	7.460411917	0.001040876	0.004231553
b0447	decR	DNA-binding transcriptional activator DecR	-0.435818893	5.914360249	0.11980126	0.2313747
b1848	yebG	DNA damage-inducible protein YebG	-0.437534878	6.565458079	0.019545717	0.053104209
b1397	paaJ	β-ketoadipyl-CoA thiolase	-0.438036163	5.943261402	0.074408705	0.158472953
b0404	acpH	EG11095-MONOMER	-0.438672727	6.191669222	0.031749523	0.080652556
b2485	hyfE	hydrogenase 4 component E	-0.438844071	4.142313779	0.382560016	0.544114802
b3561	wecH	O-acetyltransferase WecH	-0.439558094	5.298017612	0.21443959	0.356230517
b3483	yhhH	PF15631 family protein YhhH	-0.439733645	5.776537513	0.124020519	0.23693232
b2484	hyfD	hydrogenase 4 component D	-0.440052702	4.490187515	0.232685279	0.378431714
b3493	pitA	metal phosphate:H+ symporter PitA	-0.44006812	9.083161437	0.005939531	0.01941171
b3051	yqiK	protein YqiK	-0.440244538	5.59523557	0.072916086	0.155971182
b2216	rcsD	RcsD-Phis	-0.440349989	9.317815981	0.009628389	0.029357276
b0883	serW	tRNA-Ser(GGA)	-0.4404578	6.217123146	0.040405439	0.097374462
b0728	sucC	succinyl-CoA synthetase subunit β	-0.441175732	9.493453075	0.014789315	0.041837052
b1386	tynA	copper-containing amine oxidase	-0.442709363	5.490112258	0.146078727	0.268836006
b1528	ydeA	YDEA-MONOMER	-0.443859584	7.628098645	0.015481986	0.04345389
b2444	yffM	CPZ-55 prophage; uncharacterized protein YffM	-0.443876945	3.923922422	0.363965192	0.525304454
b3945	gldA	L-1,2-propanediol dehydrogenase glycerol dehydrogenase	-0.444592985	8.887729844	0.050396646	0.116310086
b1873	torY	cytochrome c quinol dehydrogenase TorY	-0.444695777	4.340299998	0.229660191	0.375258973
b3276	alaU	tRNA-Ala(UGC)	-0.445261611	8.070495135	0.028567616	0.073709123
b4052	dnaB	replicative DNA helicase	-0.445451017	8.888442816	0.010946338	0.032698298
b4422	rdlB	small regulatory antisense RNA RdlB	-0.44587566	4.837231374	0.209480202	0.350456647
b1281	pyrF	orotidine-5'-phosphate decarboxylase	-0.446264409	7.463877192	0.045365304	0.107163092
b4440	ryfA	small regulatory RNA RyfA	-0.446885382	7.335559313	0.005884182	0.019286313
b0015	dnaJ	chaperone protein DnaJ	-0.44754525	8.930780294	0.005859172	0.019232897
b2184	radD	putative DNA repair helicase RadD	-0.448043666	6.618749414	0.035571422	0.088427832
b0583	entD	phosphopantetheinyl transferase EntD	-0.44813954	4.904946802	0.179186651	0.313062549
b3830	ysgA	putative dienelactone hydrolase	-0.448660608	9.07494511	0.040458705	0.097449636
b1355	ydaG	Rac prophage; uncharacterized protein YdaG	-0.448738212	3.177129041	0.445902371	0.604439352
b2713	hydN	putative electron transport protein HydN	-0.450656628	5.484442456	0.120464185	0.232350099
b0774	bioA	adenosylmethionine-8-amino-7-oxononanoate aminotransferase	-0.451980563	4.20254541	0.293167653	0.446476436
b1142	ymfH	e14 prophage; putative uncharacterized protein YmfH	-0.45206275	4.331306585	0.319763287	0.476622185
b4270	leuX	tRNA-Leu(CAA)	-0.452149577	5.607503189	0.08907423	0.183767629
b0990	cspG	cold shock protein CspG	-0.452263787	7.694165939	0.002950887	0.010574809
b0551	ybcQ	DLP12 prophage; putative antitermination protein	-0.453301712	4.193441235	0.26377603	0.415028928
b4307	yjhQ	KpLE2 phage-like element; putative acetyltransferase TopAI antitoxin Yj	-0.453387717	3.675971977	0.361335878	0.522705734
b3162	deaD	ATP-dependent RNA helicase DeaD	-0.453464067	9.803396399	0.003162754	0.011245854
b1570	dicA	DNA-binding transcriptional dual regulator DicA	-0.454177323	6.867044667	0.010094795	0.030610246
b0536	argU	tRNA-Arg(UCU)	-0.454215307	5.98817334	0.057238365	0.12836451
b0454	atl	DNA base-flipping protein	-0.456140599	5.200934678	0.141935826	0.262534421
b1907	tyrP	TYRP-MONOMER	-0.45642528	7.298397361	0.003564467	0.012460113
b0122	yacC	putative lipoprotein YacC	-0.456936344	6.770749555	0.021032422	0.056543822
b3263	yhdU	DUF2556 domain-containing protein YhdU	-0.457069368	6.918721911	0.121625327	0.23367094

b0556	rzpD	DLP12 prophage; putative prophage endopeptidase RzpD	-0.457486415	3.691147801	0.30180388	0.455543292
b4117	adiA	arginine decarboxylase, degradative	-0.457889281	9.377420212	0.004447656	0.01505859
b2867	xdhB	putative xanthine dehydrogenase FAD-binding subunit XdhB	-0.458250232	5.05995779	0.195215152	0.333930606
b0245	ykfI	CP4-6 prophage; toxin of the YkfI-YafW toxin-antitoxin system	-0.458623183	5.960634852	0.083512003	0.174328838
b4327	hypT	DNA-binding transcriptional dual regulator HypT	-0.459103806	7.572882859	0.001268099	0.005025726
b3488	yhiJ	DUF4049 domain-containing protein YhiJ	-0.460941599	6.229411366	0.031085916	0.079377859
b1553	rzpQ	Qin prophage; DUF2514 domain-containing protein RzpQ	-0.460977409	3.335017222	0.516676173	0.666871575
b0526	cysS	cysteine—tRNA ligase	-0.461368293	8.153249832	0.004111546	0.014104486
b4666	ibsE	toxic peptide IbsE	-0.462061992	6.279447581	0.08988422	0.184882554
b4710	insB9	IS1 transposase B	-0.46209594	7.652544623	0.000564937	0.002474404
b3570	bax	putative glycoside hydrolase Bax	-0.463696037	7.764304533	0.001659157	0.006403128
b3277	ileU	tRNA-Ile(GAU)	-0.463893877	7.985609176	0.027110674	0.070366623
b4361	dnaC	DNA replication protein DnaC	-0.463953389	6.827327862	0.005692561	0.018713818
b2256	arnD	putative 4-deoxy-4-formamido-L-arabinose-phosphoundecaprenol defor	-0.463959164	6.801843698	0.013713915	0.039573161
b3868	glnG	NtrC-phosphorylated monomer	-0.465692515	9.171633649	0.036869	0.090885892
b0421	ispA	geranyl diphosphatefarnesyl diphosphate synthase	-0.465846394	8.3454796	0.002588192	0.009459326
b1335	ogt	methylated-DNA—[protein]-cysteine S-methyltransferase	-0.466007913	6.47959896	0.011166919	0.033334649
b3805	hemC	OHMETHYLBILANESYN-MONOMER	-0.467152824	9.419608456	0.026387766	0.068854603
b2684	mprA	DNA-binding transcriptional repressor MprA	-0.467748379	7.630608932	0.000683283	0.002924193
b1090	plsX	putative phosphate acyltransferase	-0.468232472	8.939843621	0.006877385	0.022018602
b0529	folD	bifunctional 5,10-methylene-tetrahydrofolate dehydrogenase 5,10-meth	-0.46934939	7.953108204	0.003206628	0.011389593
b0340	cynS	cyanase	-0.470076284	5.718939104	0.086157344	0.178584355
b1198	dhaM	dihydroxyacetone kinase subunit M	-0.470932708	9.256196983	0.005934038	0.01941171
b0988	insB-4	IS1 protein InsB	-0.471016191	7.022336062	0.002656051	0.009683289
b0280	yagN	CP4-6 prophage; protein YagN	-0.47172469	6.83151542	0.023338459	0.061885463
b1522	dgcF	putative diguanylate cyclase DgcF	-0.471966315	6.170124497	0.084553912	0.176024844
b2120	yehM	uncharacterized protein YehM	-0.472099477	4.706242989	0.200466815	0.339885172
b2566	era	30S ribosomal subunit maturation GTPase Era	-0.472858525	8.903070338	0.003509467	0.012336224
b3658	selC	tRNA-Sec(UCA)	-0.473059038	5.65288572	0.112504387	0.21970322
b2527	hscB	co-chaperone for [Fe-S] cluster biosynthesis	-0.473492576	8.034629651	0.001013204	0.004134285
b0629	ybeF	putative LysR-type DNA-binding transcriptional regulator YbeF	-0.47349437	6.028651477	0.048959675	0.113527819
b2404	lysV	tRNA-Lys(UUU)	-0.473600849	6.757308421	0.008744582	0.026998133
b1658	purR	DNA-binding transcriptional repressor PurR	-0.473820545	8.112411404	0.000631317	0.002735294
b3057	bacA	undecaprenyl pyrophosphate phosphatase	-0.474698221	7.691292422	0.000385737	0.001772145
b3088	alx	putative membrane-bound redox modulator Alx	-0.475266861	5.277409625	0.234949003	0.381500496
b4637	uof	RyhB-regulated fur leader peptide	-0.475311314	6.824609594	0.006156128	0.019999488
b2352	yfdI	CPS-53 (KpLE1) prophage; serotype specific glucosyl transferase	-0.475515767	8.028251096	0.003295032	0.011656703
b0560	nohD	DLP12 prophage; putative DNA-packaging protein NohD	-0.475642804	3.586501764	0.449683477	0.607141452
b0443	fadM	thioesterase III	-0.475801184	5.262814568	0.181653624	0.316371104
b2954	rdgB	dITPXTP pyrophosphatase	-0.475907104	7.157505636	0.003493224	0.01228891
b3584	yiaT	outer membrane protein YiaT	-0.476663163	5.126487124	0.155924631	0.282668487
b1070	flgN	flagellar biosynthesis protein FlgN	-0.477972527	5.338992206	0.119628285	0.231141741
b3322	gspB	putative general secretion pathway protein B	-0.478594254	5.311985766	0.195503937	0.334033102
b1768	pncA	nicotinamidase	-0.478599338	7.204194973	0.002423904	0.008962761
b0802	ybiJ	DUF1471 domain-containing protein YbiJ	-0.479240839	6.25743612	0.024496913	0.064530949
b3250	mreC	cell shape determining protein MreC	-0.479758668	8.890435594	0.003863809	0.013421492
b0221	fadE	acyl-CoA dehydrogenase	-0.480710894	6.578962767	0.012662722	0.037097491

b0214	rnhA	ribonuclease HI	-0.48245582	7.375515039	0.000422492	0.001907262
b2670	alaE	G7399-MONOMER	-0.483115903	8.00778546	0.001758099	0.006720353
b2027	wzzB	regulator of length of O-antigen component of lipopolysaccharide chain	-0.483392447	9.27560634	0.004248264	0.014472287
b1806	yeaY	Slp family lipoprotein YeaY	-0.483454622	7.396328751	0.000821881	0.003449243
b4340	yjiR	fused putative DNA-binding transcriptional regulatorputative aminotran	-0.484328267	6.553217488	0.023771007	0.062919063
b3505	insH-11	IS5 transposase and trans-activator	-0.484391023	6.452868001	0.021976681	0.058622089
b2851	ygeG	TPR repeat-containing putative chaperone YgeG	-0.484511404	4.240391806	0.326183524	0.483742108
b2108	yehA	putative fimbrial adhesin YehA	-0.484560913	3.734141383	0.339494711	0.498035985
b0991	ymcE	protein YmcE	-0.484578258	5.581396539	0.0754861	0.160226505
b1282	yciH	putative translation factor	-0.484692326	6.463551352	0.025332305	0.066414564
b4358	lgoD	L-galactonate oxidoreductase	-0.485537112	4.703782482	0.192020147	0.330128096
b3870	glnA	adenylyl-[glutamine synthetase]	-0.485708637	10.09787674	0.016929993	0.046950954
b2036	glf	GALPMUT-MONOMER	-0.486760112	9.79641925	0.001833724	0.006997314
b2871	ygeX	2,3-diaminopropionate ammonia-lyase	-0.487541479	5.647756512	0.098595792	0.197982579
b4608	ohsC	small regulatory RNA OhsC	-0.487860006	4.707378231	0.248897165	0.398068181
b1334	fnr	DNA-binding transcriptional dual regulator FNR	-0.488002057	8.451334894	0.002854935	0.010297825
b0967	rlmI	23S rRNA m5C1962 methyltransferase	-0.488127653	8.213996403	0.004872995	0.016298691
b2955	yggW	putative coproporphyrinogen-III oxidase-like protein YggW	-0.489007058	7.767413576	0.001715155	0.006590433
b2192	insH-8	IS5 transposase and trans-activator	-0.489082806	9.047305571	0.009341141	0.028599956
b2841	araE	arabinose:H+ symporter	-0.48988065	7.00087352	0.001812912	0.00692388
b4522	ymiA	uncharacterized protein YmiA	-0.49006786	5.836456806	0.041506911	0.099648186
b3740	rsmG	16S rRNA m7G527 methyltransferase	-0.49115813	8.13818049	0.000650984	0.002816514
b0916	ycaQ	winged helix DNA-binding domain-containing protein YcaQ	-0.491381492	6.237663109	0.029251324	0.07522318
b3249	mreD	cell shape determining protein MreD	-0.491959049	8.165963392	0.000901647	0.003752452
b2561	yfhH	putative DNA-binding transcriptional regulator YfhH	-0.492202272	6.431092294	0.023261374	0.061718128
b1625	cnu	H-NS- and StpA-binding protein	-0.493783686	6.622295792	0.016344956	0.045517787
b4362	dnaT	primosomal protein DnaT	-0.493872193	6.70217101	0.014938136	0.042114861
b3579	yiaO	2,3-diketo-L-gulonate:Na+ symporter - periplasmic binding protein	-0.495007521	4.199404254	0.278355897	0.430603114
b2189	proL	tRNA-Pro(GGG)	-0.495643293	5.018565919	0.167868025	0.298845698
b3529	pdeK	putative c-di-GMP phosphodiesterase PdeK	-0.496411821	7.984996267	0.000925139	0.003831603
b0382	iraP	anti-adaptor protein for σS stabilization	-0.496430178	6.911425542	0.014139222	0.040456685
b2311	ubiX	flavin prenyltransferase	-0.496893968	5.329416023	0.119889818	0.231444489
b4095	phnM	RPnTP hydrolase	-0.49725215	4.475781812	0.164294645	0.294144711
b0636	rlmH	23S rRNA m3Ψ1915 methyltransferase	-0.497919926	7.145002224	0.001569499	0.006083703
b4523	yciX	uncharacterized protein YciX	-0.500433628	6.434149032	0.01448937	0.0413329
b2987	pitB	metal phosphate:H+ symporter PitB	-0.50046125	5.286652615	0.108228994	0.21293717
b4322	uxuA	MANNONDEHYDRAT-MONOMER	-0.501671721	5.942939463	0.029276145	0.075235263
b3669	uhpA	UhpA-Phosphorylated	-0.501843907	7.442625961	0.000683526	0.002924193
b3752	rbsK	ribokinase	-0.502045518	8.498748582	0.001131815	0.004546828
b1948	fliP	flagellar biosynthesis protein FliP	-0.50276144	4.43408443	0.242306533	0.390431876
b0373	insE-2	IS3 element protein InsE	-0.503243011	6.562234733	0.007735612	0.024342641
b1715	pheM	pheST-ihfA operon leader peptide	-0.504180094	5.210989606	0.077457013	0.163545055
b3084	rlmG	23S rRNA m2G1835 methyltransferase	-0.504506483	6.167382892	0.033609992	0.084455387
b3739	atpI	ATP synthase accessory factor	-0.505105877	8.142677239	0.000326147	0.001533479
b0149	mrcB	peptidoglycan glycosyltransferase peptidoglycan DD-transpeptidase Mr	-0.505128418	9.287284529	0.001222634	0.004867386
b3048	yqiI	putative fimbrial protein YqiI	-0.505168128	5.406616215	0.09637997	0.194834051
b4700	sokE	small RNA SokE	-0.505279489	3.871997026	0.334149301	0.491592524

b2171	yeiP	elongation factor P family protein	-0.505679377	7.829152147	0.001453897	0.005685522
b0787	ybhM	Bax1-I family protein YbhM	-0.50694618	5.461738678	0.118545699	0.229451671
b0058	rluA	23S rRNA pseudouridine746 and tRNA pseudouridine32	-0.507462812	6.935158462	0.003637273	0.012684487
b1543	ydfJ	putative transporter YdfJ	-0.507714635	3.747540488	0.391378369	0.552585705
b3393	hofO	DNA utilization protein HofO	-0.509516998	4.41806047	0.217272374	0.359272484
b4044	dinF	DNA damage-inducible protein F	-0.509527965	7.358322374	0.000727508	0.003094364
b2864	glyU	tRNA-Gly(CCC)	-0.509642924	5.952649221	0.095130463	0.192572671
b1822	rlmA	23S rRNA m1G745 methyltransferase	-0.509727848	7.541438852	0.000160741	0.000846864
b4372	holD	DNA polymerase III subunit ψ	-0.510455359	7.077570371	0.001322286	0.005212403
b4049	dusA	tRNA-dihydrouridine synthase A	-0.510506549	6.977799911	0.003224498	0.011443858
b1436	yncJ	protein YncJ	-0.511387911	4.409331056	0.197265933	0.33626606
b2046	wzxC	G7097-MONOMER	-0.511780225	4.202083396	0.2546407	0.403966471
b2724	hycB	formate hydrogenlyase subunit HycB	-0.512147921	3.567732928	0.411623845	0.571148104
b2577	yfiE	putative LysR-type DNA-binding transcriptional regulator YfiE	-0.514188564	7.058547526	0.006492647	0.020953974
b4550	arfA	alternative ribosome-rescue factor A	-0.514209459	6.822573838	0.017356679	0.048013621
b2863	ygeQ	protein YgeQ	-0.514408345	6.033288172	0.05432509	0.123250397
b3780	rhlB	ATP-dependent RNA helicase RhlB	-0.514873808	9.96860568	0.007694057	0.024254113
b3929	rraA	ribonuclease E inhibitor protein A	-0.51673194	9.289584967	0.016136543	0.045052364
b0718	ybgQ	putative fimbrial usher protein YbgQ	-0.518451945	4.509749268	0.136275471	0.254723202
b2951	yggS	PLP homeostasis protein	-0.518570005	7.056034484	0.001282381	0.005068676
b0477	gsk	inosineguanosine kinase	-0.519197069	7.086652356	0.004149935	0.014192072
b2802	fucI	L-fucose isomerase	-0.520054893	5.687081946	0.045715591	0.107702419
b0196	rcsF	outer membrane lipoprotein RcsF	-0.520345918	7.342814017	0.000236517	0.001168035
b1123	potD	spermidine preferential ABC transporter periplasmic binding protein	-0.521271852	8.25093536	0.000490845	0.002184556
b4410	ecnA	entericidin A lipoprotein, antidote to entericidin B	-0.52131173	6.405745676	0.048667785	0.113029074
b1727	hxpB	hexitol phosphatase B	-0.521647435	6.641779713	0.002923602	0.01051116
b2536	hcaT	putative 3-phenylpropionate transporter	-0.521654431	6.380383292	0.009536831	0.029118331
b1660	ydhC	putative transporter YdhC	-0.521705751	5.691950745	0.053821935	0.122360372
b4542	yohO	UPF0387 family protein YohO	-0.521782262	5.127823278	0.087974178	0.18176573
b2170	setB	B2170-MONOMER	-0.521913242	5.847550347	0.037552794	0.092277787
b1453	ansP	ANSP-MONOMER	-0.522450092	5.699336108	0.03689951	0.09090917
b0158	btuF	vitamin Bsub12sub ABC transporter periplasmic binding protein	-0.522648691	6.772543264	0.010221554	0.0309521
b1037	csgG	curli secretion channel	-0.523025786	5.552995625	0.053256866	0.121576557
b0142	folK	H2PTERIDINEPYROPHOSPHOKIN-MONOMER	-0.523147628	6.435820262	0.019706842	0.053509045
b0259	insH-1	CP4-6 prophage; IS5 transposase and trans-activator	-0.523355923	8.331727496	0.000314968	0.001488055
b0109	nadC	quinolinate phosphoribosyltransferase (decarboxylating)	-0.523657994	7.963505749	0.000132414	0.000716433
b0069	sgrR	DNA-binding transcriptional dual regulator SgrR	-0.52371989	6.788138787	0.010511009	0.031612188
b1213	ychQ	SirB family protein YchQ	-0.524223795	6.706064342	0.004236976	0.014467322
b0630	lipB	lipoyl(octanoyl) transferase	-0.52437863	7.507082825	0.000452449	0.002030045
b1204	pth	peptidyl-tRNA hydrolase	-0.526902589	6.836687219	0.017317421	0.047935056
b4210	ytfF	inner membrane protein YtfF	-0.527078986	6.164680223	0.01670305	0.046379852
b1808	yoaA	putative 5' to 3' DNA helicase implicated in DNA repair	-0.527849656	7.907465247	0.000771718	0.003257298
b0931	pncB	NICOTINATEPRIBOSYLTRANS-MONOMER	-0.528170264	8.137517247	0.000252919	0.001239329
b3567	xylG	xylose ABC transporter ATP binding subunit	-0.530345405	5.409926076	0.090306093	0.185473824
b0173	dxr	1-deoxy-D-xylulose 5-phosphate reductoisomerase	-0.531701551	8.745975466	0.000609473	0.002653909
b3011	yqhD	NADPH-dependent aldehyde reductase YqhD	-0.532435488	8.478353312	0.002403276	0.008901394
b0099	mutT	8-oxo-dGTP diphosphatase	-0.533606155	6.252397807	0.015887875	0.044480004

b4137	cutA	copper binding protein CutA	-0.533699061	6.107373443	0.013633936	0.039393865
b0274	insB-3	IS1 protein InsB	-0.534314976	7.22902924	0.000202011	0.001019289
b4536	yobH	protein YobH	-0.535931544	6.678101442	0.007696492	0.024254113
b3001	gpr	L-glyceraldehyde 3-phosphate reductase	-0.536164236	6.727094807	0.004783003	0.016058523
b1254	yciB	inner membrane protein	-0.536451212	7.092130773	0.001507264	0.005857895
b3475	acpT	holo-[acyl carrier protein] synthase 2	-0.537395028	7.483459507	5.95E-05	0.00035812
b3728	pstS	phosphate ABC transporter periplasmic binding protein	-0.537813624	7.90227174	9.88E-05	0.000563378
b1986	asnU	tRNA-Asn(GUU)	-0.538052302	5.982753247	0.027252011	0.070608938
b1143	ymfI	e14 prophage; uncharacterized protein YmfI	-0.539505005	7.030982523	0.00284225	0.01026885
b2586	yfiM	protein YfiM	-0.539835465	7.620600341	0.000838016	0.003513619
b3401	hslO	molecular chaperone Hsp33	-0.540233859	8.086961443	0.000106523	0.000594561
b2074	mdtA	multidrug efflux pump membrane fusion protein MdtA	-0.541883885	6.612271017	0.035540976	0.088401921
b0313	betI	DNA-binding transcriptional repressor BetI	-0.542073465	7.447952914	0.000713926	0.003044516
b3703	rpmH	50S ribosomal subunit protein L34	-0.542866	7.448711211	0.000362652	0.001683604
b2968	yghD	putative type II secretion system M-type protein	-0.544335376	5.198921154	0.123994057	0.23693232
b1498	ydeN	putative sulfatase	-0.54441339	4.773378286	0.223263863	0.367254082
b3075	ebgR	DNA-binding transcriptional repressor EbgR	-0.544662649	7.242960488	0.000509528	0.002256336
b0380	yaiZ	DUF2754 domain-containing protein YaiZ	-0.54518238	5.709302338	0.056393158	0.127039544
b0637	rsfS	ribosomal silencing factor RsfS	-0.545507735	7.219116976	0.000338957	0.001585271
b0018	mokC	regulatory protein MokC	-0.546786853	5.547948877	0.047616115	0.111171416
b1942	fliJ	flagellar biosynthesis protein FliJ	-0.549957021	3.927332637	0.231749913	0.377417883
b0833	pdeI	putative c-di-GMP phosphodiesterase PdeI	-0.550457915	8.859649477	0.000876723	0.003658536
b3181	greA	transcription elongation factor GreA	-0.55098234	7.962363352	0.000189432	0.000966868
b3643	rph	truncated RNase PH	-0.552186869	7.284894992	0.001338412	0.005271268
b0341	cynX	CYNX-MONOMER	-0.553581816	5.634997139	0.043907706	0.104446402
b1257	yciE	DUF892 domain-containing protein YciE	-0.553685321	5.077127766	0.074749595	0.15912221
b3587	yiaW	DUF3302 domain-containing protein YiaW	-0.555127728	4.127334028	0.256895107	0.406812014
b2420	yfeS	PF05406 family protein YfeS	-0.555331014	7.965946349	7.71E-05	0.000450543
b3912	cpxR	Phosphorylated DNA-binding transcriptional dual regulator CpxR	-0.555568302	9.306814656	0.011823439	0.03508097
b0067	thiP	thiamin ABC transporter membrane subunit	-0.555776372	5.689030551	0.044240326	0.104898518
b3804	hemD	UROGENIIISYN-MONOMER	-0.558658546	9.236620666	0.008211275	0.025620339
b4412	hokC	protein HokC	-0.558752248	5.4178741	0.044074068	0.104658176
b1989	asnV	tRNA-Asn(GUU)	-0.559263847	5.724257961	0.034901042	0.087203225
b0949	uup	ATP-binding protein with possible role in replication	-0.56036486	9.251312476	0.000693258	0.002962957
b0631	ybeD	DUF493 domain-containing protein YbeD	-0.560877643	6.949617923	0.000387208	0.00177705
b0468	ybaN	conserved inner membrane protein YbaN	-0.561277857	6.217987361	0.008139242	0.025449544
b2669	stpA	DNA-binding transcriptional repressor StpA with RNA chaperone activity	-0.561303897	6.358951664	0.024945767	0.065635019
b4313	fimE	regulator for fimA	-0.561364499	5.114161835	0.122143674	0.23441868
b1251	yciI	protein YciI	-0.562421959	6.335673325	0.006443915	0.020842405
b3018	plsC	1-acylglycerol-3-phosphate O-acyltransferase	-0.563896128	7.822482469	1.20E-05	8.26E-05
b2509	xseA	exodeoxyribonuclease VII subunit XseA	-0.564119027	8.614234677	0.00035952	0.00167082
b2172	yeiQ	putative dehydrogenase, NAD-dependent	-0.564366264	5.940606979	0.030143697	0.077195141
b3641	slmA	nucleoid occlusion factor SlmA	-0.566106386	7.197836861	0.000421977	0.001906886
b1497	ydeM	putative anaerobic sulfatase maturation enzyme YdeM	-0.566217834	3.766330011	0.27320502	0.424867969
b0118	acnB	AcnB	-0.566702858	10.45610035	0.001462295	0.005708253
b2272	yfbM	DUF1877 domain-containing protein YfbM	-0.566739149	3.737942261	0.376619758	0.537245956
b2531	iscR	DNA-binding transcriptional dual regulator IscR	-0.568439925	8.167390383	0.000141698	0.000757909

b4714	ralA	Rac prophage; antitoxin RalA	-0.568805855	3.160844744	0.393259442	0.553648099
b0398	sbcD	ATP dependent, structure specific DNA nuclease - SbcD subunit	-0.56929399	7.358443155	0.000207845	0.001043955
b4612	yrhD	uncharacterized protein YrhD	-0.56952662	4.486767246	0.147541207	0.27096274
b3645	dinD	DNA damage-inducible protein D	-0.569956781	6.364231853	0.004581283	0.015451768
b0108	ppdD	prepilin-type N-terminal cleavagemethylation domain-containing protei	-0.570125378	4.459244752	0.198540906	0.337397267
b1496	yddA	ABC transporter family protein YddA	-0.570420254	2.997212178	0.634013812	0.761264884
b2734	pphB	phosphoprotein phosphatase 2	-0.572052209	5.009808106	0.112046269	0.219080724
b0278	yagL	CP4-6 prophage; resolvase-like catalytic domain-containing protein YagI	-0.574292849	6.407637133	0.01773691	0.048851191
b0543	emrE	multidrugbetaine	-0.575737204	7.161964124	0.000203161	0.001023923
b0842	mdfA	multidrug efflux pump MdfA Na+	-0.576585119	6.460473759	0.003128445	0.011156773
b0727	sucB	SucB-dihydrolipoate	-0.576875735	9.606865756	0.001310439	0.005174943
b4555	yicS	uncharacterized protein YicS	-0.577449985	5.593239858	0.029454193	0.075560872
b3264	envR	DNA-binding transcriptional repressor EnvR	-0.578527143	6.662506065	0.045418818	0.107190903
b0602	ybdN	putative PAPS reductaseDUF3440 domain-containing protein YbdN	-0.578991252	4.335620373	0.127492978	0.241787586
b3042	ubiK	ubiquinone biosynthesis accessory factor UbiK	-0.580388001	6.938952564	0.000237633	0.001172235
b0422	xseB	exodeoxyribonuclease VII subunit XseB	-0.580700303	6.935371091	0.000420197	0.001900789
b2030	insH-7	IS5 transposase and trans-activator	-0.581887653	7.638285535	1.63E-05	0.000109028
b3709	tnaB	TNAB-MONOMER	-0.581943062	5.424897545	0.044283564	0.10490433
b2483	hyfC	hydrogenase 4 component C	-0.583521809	4.190446141	0.160452434	0.289123789
b3452	ugpA	sn-glycerol 3-phosphate ABC transporter membrane subunit UgpA	-0.583889318	4.616447293	0.135117526	0.253201986
b3563	yiaB	conserved inner membrane protein YiaB	-0.584086361	4.679310569	0.16532339	0.29526811
b4289	fecC	ferric citrate ABC transporter membrane subunit FecC	-0.584368795	4.866140628	0.056781075	0.127617771
b2558	mltF	membrane-bound lytic murein transglycosylase F	-0.584574817	7.223418829	0.000286187	0.001385434
b0799	dinG	ATP-dependent DNA helicase DinG	-0.586291866	8.042443375	0.000436755	0.001963619
b4089	alsR	DNA-binding transcriptional repressor AlsR	-0.587069898	6.959288181	0.001731783	0.006636999
b1530	marR	DNA-binding transcriptional repressor MarR	-0.587209799	6.404820073	0.007179354	0.022869298
b0666	metU	tRNA-Met(CAU)	-0.589573659	6.754922647	0.000729055	0.003097957
b4685	yrbN	uncharacterized protein YrbN	-0.589690882	6.858905185	0.002161139	0.00811346
b1976	mtfA	Mlc titration factor	-0.589885036	6.933307679	0.002428926	0.008966312
b0225	yafQ	ribosome-dependent mRNA interferase toxin YafQ	-0.590070836	6.589091331	0.003525963	0.012374503
b3258	panF	PANF-MONOMER	-0.590170371	7.713621599	0.000288334	0.001394296
b3047	yqiH	putative fimbrial chaperone YqiH	-0.590722082	4.217996253	0.137609367	0.25645646
b2505	yfgH	lipoprotein YfgH	-0.591317051	4.54647626	0.169753669	0.30147323
b4268	idnK	D-gluconate kinase, thermosensitive	-0.591516114	4.425570511	0.087668615	0.181291305
b1099	holB	DNA polymerase III subunit δ'	-0.591964767	7.795632037	0.000241749	0.001191208
b2230	yfaA	DUF2138 domain-containing protein YfaA	-0.593150401	5.964167397	0.03132188	0.07974977
b1819	manZ	mannose-specific PTS enzyme IID component	-0.59354019	8.506493741	9.01E-05	0.00051795
b1155	tfaP	e14 prophage; putative tail fiber assembly protein TfaP	-0.593734238	4.222945122	0.124849766	0.237501418
b4665	ibsC	toxic peptide IbsC	-0.594135624	6.128103382	0.014842819	0.041953295
b1513	lsrA	Autoinducer-2 ABC transporter ATP binding subunit	-0.596108369	4.017444932	0.162825804	0.292107242
b1228	ychS	putative uncharacterized protein YchS	-0.596177853	4.425814409	0.116934892	0.226731465
b1148	ymfM	e14 prophage; uncharacterized protein YmfM	-0.59646153	4.311853032	0.16502509	0.294854622
b4341	yjiS	DUF1127 domain-containing protein YjiS	-0.597256773	2.446853632	0.491262623	0.643781134
b3066	dnaG	DNA primase	-0.597418328	9.336287191	0.001109166	0.004463964
b4427	micC	small regulatory RNA MicC	-0.597952318	2.503050101	0.4237344	0.58370901
b4425	rttR	small RNA RttR	-0.597994302	5.259928534	0.048273752	0.112277228
b2824	ygdB	DUF2509 domain-containing protein YgdB	-0.598752617	4.866554385	0.123416531	0.236137713

b1605	ydgI	putative arginine:ornithine antiporter	-0.599423532	6.104141787	0.010745954	0.032252472
b1984	asnW	tRNA-Asn(GUU)	-0.600100323	6.011590475	0.01195124	0.035369321
b1348	ralR	Rac prophage; endodeoxyribonuclease toxin RalR	-0.603103002	2.855707005	0.440257936	0.599179651
b2953	yggU	DUF167 domain-containing protein YggU	-0.605194236	6.394361967	0.001687125	0.006499699
b2188	yejM	putative cardiolipin transport protein	-0.60540993	8.565684215	0.000112424	0.000622773
b3400	hslR	heat shock protein Hsp15	-0.606070603	7.347347577	2.22E-05	0.000144009
b2443	yffL	CPZ-55 prophage; uncharacterized protein YffL	-0.606211492	7.485793332	2.20E-05	0.000143443
b2975	glcA	glycolatelactate:H+	-0.606365395	5.080466019	0.121417531	0.233496933
b0135	yadC	fimbrial tip-adhesin YadC	-0.607185112	5.980612821	0.013955735	0.040113654
b2044	wcaL	putative colanic biosynthesis glycosyl transferase	-0.607763008	3.932573189	0.260033398	0.410310026
b2590	gltW	tRNA-Glu(UUC)	-0.608125651	7.907908894	0.000953198	0.003925717
b4314	fimA	type 1 fimbriae major subunit	-0.60823481	8.387240106	0.001010833	0.004129793
b3028	mdaB	NADPH:quinone oxidoreductase MdaB	-0.608862761	7.855504669	6.74E-06	4.94E-05
b0868	ybjS	putative NAD(P)H-binding oxidoreductase with NAD(P)-binding Rossmar	-0.608884493	7.408107344	0.000155641	0.000824916
b2618	yfjF	putative component of the Rsx system	-0.608903758	7.051087291	0.000183192	0.00094243
b3572	avtA	valine—pyruvate aminotransferase	-0.609136485	8.214959264	9.63E-05	0.000550863
b2872	ygeY	putative peptidase YgeY	-0.609527739	5.11416201	0.081039912	0.169810731
b0465	mscK	potassium dependent, small conductance mechanosensitive channel	-0.609705065	9.377296477	0.000100896	0.000570363
b3064	tsaD	N6-L-threonylcarbamoyladenine synthase, TsaD subunit	-0.610544326	8.493641216	0.0005108	0.0022597
b2321	flk	putative flagella assembly protein	-0.610652352	7.333834033	0.000305933	0.001457057
b0738	tolR	Tol-Pal system protein TolR	-0.611653191	8.020238193	0.000115756	0.000636444
b0739	tolA	Tol-Pal system protein TolA	-0.612162439	9.222987028	0.000249175	0.001225062
b2578	eamB	cysteineO	-0.612673743	6.180335079	0.012251933	0.036157945
b2725	hycA	regulator of the transcriptional regulator FhlA	-0.612877321	3.870020982	0.276860382	0.429191919
b4087	alsA	D-allose ABC transporter ATP binding subunit	-0.612908215	5.097326313	0.048285669	0.112277228
b2367	emrY	tripartite efflux pump membrane subunit EmrY	-0.613052636	4.096717034	0.16476949	0.294534535
b0571	cusR	Pasp DNA-binding transcriptional activator CusR	-0.613108995	6.506839353	0.004805185	0.016120739
b0402	proY	putative transporter ProY	-0.613610302	7.834642154	3.09E-06	2.45E-05
b1977	asnT	tRNA-Asn(GUU)	-0.613731511	6.124861555	0.009143901	0.028073938
b1559	ydfT	Qin prophage; putative antitermination protein Q	-0.617822657	6.258688736	0.007821374	0.024558756
b4360	yjjA	DUF2501 domain-containing protein YjjA	-0.618779292	6.824805358	0.00038875	0.001780427
b0207	dkgB	methylglyoxal reductase DkgB	-0.620385686	7.67223243	5.56E-05	0.00033788
b1439	ydcR	fused putative DNA-binding transcriptional regulatorputative aminotran	-0.620737085	6.938527373	0.000609527	0.002653909
b4365	yjjQ	DNA-binding transcriptional repressor YjjQ	-0.620808374	2.780553227	0.445579058	0.604186591
b2041	rfbB	dTDP-glucose 4,6-dehydratase 1	-0.621754429	9.269936912	0.000210664	0.001054892
b3938	metJ	DNA-binding transcriptional repressor MetJ	-0.621818372	9.131483265	0.004475903	0.015125466
b0822	ybiV	sugar phosphatase	-0.622361217	7.180163984	0.000176528	0.000912613
b0299	insF-1	IS3 element protein InsF	-0.622532927	7.414830788	2.43E-05	0.000156571
b0910	cmk	cytidylate kinase	-0.62301791	8.193918072	0.000385275	0.001771863
b0921	cmoM	tRNA bcmob5	-0.623393802	7.894667828	3.29E-06	2.59E-05
b0065	yabI	DedA family protein YabI	-0.625805269	6.97903383	0.000388408	0.001780427
b0295	ykgL	uncharacterized protein YkgL	-0.626178703	5.995633873	0.013331318	0.038722215
b2251	nudI	pyrimidine deoxynucleotide diphosphatase NudI	-0.629045992	5.537274494	0.028947229	0.074520126
b0874	lysO	L-lysine exporter	-0.629258852	4.947141311	0.06281984	0.13853626
b3894	fdoG	formate dehydrogenase O subunit α	-0.630437479	10.45171566	0.001075061	0.0043505
b4746	yddY	protein YddY	-0.63155062	4.351947223	0.124416058	0.237277277
b1561	rem	Qin prophage; protein Rem	-0.631607196	5.235931725	0.026245451	0.0685643

b2965	speC	ornithine decarboxylase, biosynthetic	-0.631776674	8.11803583	0.000404238	0.001840907
b0892	rarA	recombination factor	-0.632325682	8.388687471	0.000197301	0.001003553
b3640	dut	deoxyuridine triphosphatase	-0.632589585	7.347245205	3.69E-05	0.000230403
b4753	yqfH	protein YqfH	-0.633752264	4.855535576	0.166704007	0.29689318
b1040	csgD	DNA-binding transcriptional dual regulator CsgD	-0.635366948	4.517440366	0.135407278	0.253529743
b4218	ytfL	putative inner membrane protein	-0.635600481	7.363100424	7.71E-05	0.000450543
b2765	queD	6-carboxy-5,6,7,8-tetrahydropterin synthase	-0.636725654	6.129646478	0.003095765	0.011049155
b4098	phnJ	carbon-phosphorus lyase core complex subunit PhnJ	-0.637209236	4.155460585	0.103549371	0.205839924
b3027	ygiZ	conserved inner membrane protein YgiZ	-0.637548059	4.366336473	0.121475603	0.233496933
b1457	ydcD	uncharacterized protein YdcD	-0.637632641	6.2298231	0.01045755	0.031536942
b0143	pcnB	poly(A) polymerase I	-0.638282624	8.930791027	9.31E-05	0.00053467
b3696	yidX	putative lipoprotein YidX	-0.638587209	7.283088693	0.000439033	0.00197185
b0427	yajR	putative major facilitator superfamily transport protein YajR	-0.640443537	7.868861024	3.08E-05	0.00019532
b2576	srmB	ATP-dependent RNA helicase SrmB	-0.640703205	7.889394222	1.21E-05	8.34E-05
b0016	insL-1	IS186IS421 transposase	-0.641568494	8.745426755	6.95E-05	0.000410403
b4509	ylcG	DLP12 prophage; uncharacterized protein YlcG	-0.641864717	2.186151371	0.603389643	0.737520981
b2761	cas3	CRISPR-associated endonucleasehelicase Cas3	-0.64270182	6.475696976	0.002776216	0.010079764
b4371	rsmC	16S rRNA m2G1207 methyltransferase	-0.643290593	7.793192713	2.16E-06	1.76E-05
b4671	ykgR	uncharacterized membrane protein YkgR	-0.644004768	5.586637485	0.040930637	0.09847889
b0971	serT	tRNA-Ser(UGA)	-0.64516708	5.446368445	0.017797603	0.04895727
b3865	yihA	GTP-binding protein YihA	-0.645297366	9.137115603	0.001077294	0.004355544
b4017	arpA	regulator of acetyl CoA synthetase	-0.645400961	5.851593451	0.015310513	0.043054723
b0264	insB-2	IS1 protein InsB	-0.645491395	6.730669852	0.000173075	0.000898972
b2952	yggT	uncharacterized protein YggT	-0.645694711	7.346843791	1.59E-05	0.000106623
b0541	insF-3	DLP12 prophage; IS3 element protein InsF	-0.647218129	7.713935814	2.85E-05	0.000182191
b3096	mzrA	modulator protein MzrA	-0.647992541	6.842390181	0.000753045	0.003187627
b2945	endA	DNA-specific endonuclease I	-0.647993666	5.360742468	0.040361028	0.097320555
b4112	basS	sensory histidine kinase BasS - phosphorylated	-0.648278487	7.659462294	5.23E-06	3.92E-05
b1144	ymfJ	e14 prophage; uncharacterized protein YmfJ	-0.648358361	6.305476695	0.004053275	0.013932117
b0502	ylbG	putative DNA-binding transcriptional regulator YlbG	-0.649065989	5.683717684	0.025651333	0.06713138
b0226	dinJ	antitoxinDNA-binding transcriptional repressor DinJ	-0.652108483	7.087218836	0.000309833	0.001470873
b3171	metY	tRNA-initiator Met(CAU)	-0.652276802	6.785871809	0.003757274	0.013061704
b0915	lpxK	TETRAACYLDISACC4KIN-MONOMER	-0.652508818	7.458647376	1.72E-05	0.000114595
b0657	lnt	apolipoprotein N-acyltransferase	-0.652662457	8.406300549	1.96E-05	0.000129287
b3741	mnmG	5-carboxymethylaminomethyluridine-tRNA synthase subunit MnmG	-0.653293045	9.0914731	0.000456899	0.002047928
b2039	rfbA	dTDP-glucose pyrophosphorylase	-0.657404663	9.105287877	0.000104725	0.000586751
b0417	thiL	thiamine monophosphate kinase	-0.657878177	7.947221378	1.70E-06	1.42E-05
b1408	ynbA	inner membrane protein YnbA	-0.659846777	2.253322941	0.551874396	0.693123738
b1821	mntP	G6999-MONOMER	-0.660974217	5.814310656	0.014641014	0.041542466
b3076	ebgA	evolved β-D-galactosidase subunit α	-0.662305878	5.577715104	0.130025899	0.245868117
b4048	yjbM	uncharacterized protein YjbM	-0.662466742	5.756143968	0.037622021	0.092381102
b4618	tisB	membrane-depolarizing toxin TisB	-0.663467947	5.8149542	0.01261378	0.036991512
b3925	glpX	fructose-1,6-bisphosphatase 2	-0.664996712	9.079051959	0.003478886	0.012248231
b0445	ybaE	putative protein YbaE	-0.666426149	6.314281617	0.006621271	0.021284878
b1289	ycjD	DUF559 domain-containing protein YcjD	-0.666767559	4.663882378	0.083408635	0.174195423
b2623	yfjH	CP4-57 prophage; protein YfjH	-0.667786514	7.56745265	1.14E-05	7.92E-05
b0613	citG	G6339-MONOMER	-0.667823736	3.056368469	0.269576987	0.421801199

b3891	fdhE	formate dehydrogenase formation protein	-0.667873039	9.446677046	0.000918855	0.003809149
b3727	pstC	phosphate ABC transporter membrane subunit PstC	-0.668468699	7.270541492	6.63E-06	4.87E-05
b0530	sfmA	putative fimbrial protein SfmA	-0.669943125	3.362696531	0.191299933	0.329274544
b1022	pgaC	poly-N-acetyl-D-glucosamine synthase subunit PgaC	-0.670468207	3.250993812	0.19186298	0.329986387
b4106	phnC	phosphonate ABC transporter ATP binding subunit	-0.670904307	4.117349792	0.110968812	0.217166358
b2351	yfdH	CPS-53 (KpLE1) prophage; bactoprenol glucosyl transferase	-0.673496535	8.940003238	0.000164168	0.000863885
b1139	lit	e14 prophage; cell death peptidase Lit	-0.673863706	6.482620782	0.002525127	0.009267196
b0819	ldtB	L,D-transpeptidase LdtB	-0.674969857	8.659932529	0.000113027	0.000624548
b0262	afuC	CP4-6 prophage; ABC transporter ATP-binding protein AfuC	-0.675904252	5.433013813	0.036919624	0.09090917
b2506	yfgI	nalidixic acid resistance protein YfgI	-0.676159358	5.415305095	0.035200385	0.087656628
b2798	ygdG	flap endonuclease	-0.676356481	5.991413775	0.00452243	0.015264931
b1921	fliZ	DNA-binding transcriptional regulator FliZ	-0.677286616	5.262500105	0.021265868	0.057040588
b4066	yjcF	pentapeptide repeat-containing protein YjcF	-0.678317599	5.67898658	0.01201912	0.03554214
b3632	waaQ	lipopolysaccharide core heptosyltransferase 3	-0.678614851	7.766408193	5.62E-05	0.000340551
b2880	ygfM	putative oxidoreductase	-0.679411697	4.94938443	0.064541797	0.141275178
b2671	ygaC	protein YgaC	-0.679656528	7.13281902	6.55E-05	0.000390059
b1039	csgE	curli transport specificity factor	-0.679778521	2.510000449	0.473453896	0.628283424
b2912	fau	putative 5-formyltetrahydrofolate cyclo-ligase	-0.681662081	7.5414785	1.13E-06	9.80E-06
b1919	dcyD	D-cysteine desulfhydrase	-0.681904813	8.273740335	1.04E-05	7.28E-05
b1420	mokB	putative regulatory protein MokB	-0.683170475	5.722704845	0.006580057	0.021189608
b0661	miaB	isopentenyl-adenosine A37 tRNA methylthiolase	-0.683437221	8.768240101	0.00021565	0.001073388
b0582	insL-2	IS186IS421 transposase	-0.684585896	8.682468529	1.96E-05	0.000129594
b4453	ldrD	small toxic polypeptide LdrD	-0.685636301	6.433493339	0.000517299	0.002281596
b1283	osmB	osmotically-inducible lipoprotein OsmB	-0.687181647	7.754979032	1.72E-06	1.43E-05
b4553	ysaB	putative lipoprotein YsaB	-0.688612921	6.086315254	0.001940327	0.007353257
b1552	cspI	Qin prophage; cold shock protein CspI	-0.688862213	5.909888161	0.006289425	0.020387526
b1987	cbl	DNA-binding transcriptional activator Cbl	-0.691855256	7.47694538	4.77E-07	4.42E-06
b2095	gatZ	tagatose-1,6-bisphosphate aldolase 2 subunit GatZ	-0.691963786	10.02724659	3.60E-05	0.000224777
b2419	yfeK	DUF5329 domain-containing protein YfeK	-0.693903796	6.396008169	0.000378876	0.00174425
b2629	yfjM	CP4-57 prophage; protein YfjM	-0.694143269	5.891590235	0.016142296	0.045052364
b4620	yjbT	PF17089 family protein YjbT	-0.695200175	4.870665884	0.039236074	0.09545226
b1950	fliR	flagellar biosynthesis protein FliR	-0.695966126	4.699519307	0.037248911	0.091668864
b0545	ybcL	DLP12 prophage; periplasmic protein YbcL	-0.699722172	5.651803474	0.009751261	0.029694511
b4451	ryhB	small regulatory RNA RyhB	-0.699924905	4.921395357	0.044395658	0.105097497
b0955	ycbZ	putative ATP-dependent protease YcbZ	-0.700536067	8.880990003	7.92E-06	5.74E-05
b3869	glnL	sensory histidine kinase NtrB	-0.701048695	9.04779311	0.002671198	0.009730479
b1859	znuB	Zn2+ ABC transporter membrane subunit	-0.701239043	6.903289281	2.39E-05	0.000154088
b1798	leuE	leucine exporter	-0.701757847	5.850580103	0.002832116	0.010240615
b1631	rsxG	SoxR [2Fe-2S] reducing system protein RsxG	-0.702597207	6.672327888	0.000201607	0.001018415
b4375	prfC	peptide chain release factor RF3	-0.705017571	9.46569332	6.38E-05	0.000381135
b1020	phoH	ATP-binding protein PhoH	-0.705061208	5.590780914	0.007211719	0.022922777
b1974	yodB	putative cytochrome	-0.705837831	5.771092622	0.003991316	0.013803034
b3005	exbD	Ton complex subunit ExbD	-0.707347388	7.303145711	6.29E-06	4.65E-05
b2866	xdhA	putative xanthine dehydrogenase molybdenum-binding subunit XdhA	-0.707533336	6.323587209	0.002942088	0.010560422
b0224	ldtF	L,D-transpeptidase domain-containing protein LdtF	-0.709302117	8.488200972	2.24E-06	1.81E-05
b0617	citD	citrate lyase acyl carrier protein	-0.710394713	2.433972025	0.333172852	0.490810191
b1804	rnd	RNase D	-0.711478735	8.441176578	9.34E-06	6.66E-05

b2886	ygfS	putative oxidoreductase, 4Fe-4S ferredoxin-type subunit	-0.712094575	4.878105373	0.164614021	0.294477675
b0853	ybjN	protein YbjN	-0.712250193	7.679715628	3.85E-08	4.37E-07
b2983	yghQ	putative transport protein YghQ	-0.712738516	5.766802189	0.005516118	0.018187946
b2985	yghS	putative ATP-binding protein YghS	-0.712866054	4.067584107	0.168415101	0.299698779
b0635	mrdA	peptidoglycan DD-transpeptidase MrdA	-0.713240914	8.72286065	3.35E-05	0.000211091
b4713	agrB	small RNA AgrB	-0.713565529	4.8607228	0.048261454	0.112277228
b3414	nfuA	iron-sulfur cluster carrier protein NfuA	-0.716709797	8.178920733	1.01E-06	8.86E-06
b4231	yjfF	galactofuranose ABC transporter putative membrane subunit YjtF	-0.717324356	5.37981231	0.013593792	0.039329352
b0885	aat	leucylphenylalanyl-tRNA—protein transferase	-0.71744795	6.497891714	0.00049249	0.002189671
b1203	ychF	redox-responsive ATPase YchF	-0.717627652	8.548905581	2.60E-05	0.00016642
b2876	yqeC	uncharacterized protein YqeC	-0.718590797	5.110916545	0.071112656	0.152788428
b3006	exbB	Ton complex subunit ExbB	-0.71926994	8.253674477	4.55E-06	3.45E-05
b3726	pstA	phosphate ABC transporter membrane subunit PstA	-0.72045318	7.32243096	3.51E-07	3.34E-06
b3631	waaG	lipopolysaccharide glucosyltransferase I	-0.720726696	8.085520631	3.32E-07	3.18E-06
b2394	insL-3	putative IS186IS421 transposase	-0.722806709	8.698185023	8.08E-06	5.84E-05
b4520	ymgF	inner membrane protein that interacts with cell division proteins	-0.723506389	4.300630659	0.068401774	0.148541316
b4676	yoaK	uncharacterized membrane protein YoaK	-0.725413094	5.852909945	0.002678122	0.009739627
b0771	ybhJ	putative hydratase YbhJ	-0.725987418	6.067269451	0.004410785	0.014968191
b2442	intZ	CPZ-55 prophage; putative phage integrase IntZ	-0.726479273	7.537069163	9.13E-07	8.08E-06
b4005	purD	GLYCRIBONUCSYN-MONOMER	-0.726565814	8.859876144	0.003081342	0.011015484
b2187	yejL	DUF1414 domain-containing protein YejL	-0.727355314	6.586759316	0.000167581	0.000878272
b4528	rzoR	Rac prophage; putative lipoprotein	-0.727891183	4.201119237	0.069284982	0.149947645
b4326	iraD	anti-adaptor protein IraD, inhibitor of σS proteolysis	-0.729983511	7.574772704	5.44E-06	4.07E-05
b3965	trmA	tRNA m5U54 methyltransferase	-0.73025121	9.439602016	0.000154273	0.000818649
b2255	arnA	fused UDP-4-amino-4-deoxy-L-arabinose formyltransferaseUDP-glucuro	-0.730732299	8.185601564	0.000175546	0.00090865
b0372	insF-2	IS3 element protein InsF	-0.732775373	7.750084046	5.87E-06	4.35E-05
b0068	thiB	thiamin ABC transporter periplasmic binding protein	-0.733697603	6.065825153	0.000797209	0.003352076
b3095	yqjA	DedA family protein YqjA	-0.733845079	7.352011166	5.54E-06	4.14E-05
b2390	ypeC	DUF2502 domain-containing protein YpeC	-0.734279791	6.660396909	0.0001982	0.001005808
b2808	gcvA	DNA-binding transcriptional dual regulator GcvA	-0.734556935	7.609340807	1.57E-07	1.59E-06
b0985	gfcC	capsule biosynthesis GfcC family protein	-0.734895928	4.140457775	0.063794827	0.140126449
b1147	ymfL	e14 prophage; uncharacterized protein YmfL	-0.735401502	5.229087243	0.036501668	0.090131356
b0948	rlmL	fused 23S rRNA m2G2445 methyltransferase and 23S rRNA m7	-0.735742522	8.915818021	0.000290371	0.001397978
b1524	glsB	glutaminase 2	-0.736212879	7.2504496	1.31E-05	8.97E-05
b1264	trpE	anthranilate synthase subunit TrpE	-0.736310145	4.313457222	0.054645607	0.123786739
b2567	rnc	RNase III	-0.736652906	8.586281395	1.11E-05	7.67E-05
b3931	hslU	ATPase component of the HslVU protease	-0.737476593	9.824200986	0.000406428	0.001846846
b0726	sucA	subunit of E1(0) component of 2-oxoglutarate dehydrogenase	-0.738117112	10.66173542	2.13E-05	0.000139058
b0772	ybhC	outer membrane lipoprotein YbhC	-0.738304429	8.783440533	9.93E-06	7.00E-05
b2038	rfbC	DTDPDEHYDRHAMEPIM-MONOMER	-0.739067742	8.408155042	3.45E-06	2.69E-05
b3127	garP	galactarateglucarate	-0.740472153	5.250813517	0.087980377	0.18176573
b3539	yhjV	putative transporter YhjV	-0.74083216	6.34064952	0.000156896	0.000830572
b0645	ybeR	DUF1266 domain-containing protein YbeR	-0.74352548	2.375298869	0.415656745	0.574733645
b1137	ymfD	e14 prophage; putative SAM-dependent methyltransferase	-0.743900025	6.562068928	0.000963715	0.003961642
b1352	kilR	Rac prophage; inhibitor of FtsZ, killing protein	-0.744539437	3.068351964	0.221546182	0.364958877
b2040	rfbD	DTDPDEHYRHAMREDUCT-MONOMER	-0.74460914	8.885851716	9.33E-05	0.000534875
b0469	apt	adenine phosphoribosyltransferase	-0.7452106	7.604164772	4.40E-08	4.95E-07

b4352	yjiA	P-loop guanosine triphosphatase YjiA	-0.746288517	7.842938319	1.23E-05	8.42E-05
b3705	yidC	membrane protein insertase YidC	-0.74634597	9.588674401	3.63E-06	2.81E-05
b2733	mutS	DNA mismatch repair protein MutS	-0.746751978	8.683349089	5.39E-05	0.00032836
b2753	iap	alkaline phosphatase isozyme conversion protein	-0.748200712	8.558278133	8.78E-06	6.28E-05
b4068	yjcH	conserved inner membrane protein YjcH	-0.749374133	4.158853932	0.109197185	0.214364416
b0569	nfrB	bacteriophage N4 receptor, inner membrane subunit	-0.749652126	7.113708346	2.50E-06	2.00E-05
b0238	gpt	xanthine-guanine phsophoribosyltransferase	-0.750061172	7.907806919	1.10E-07	1.14E-06
b2481	hyfA	hydrogenase 4 component A	-0.750720881	3.703017905	0.172760649	0.304851425
b1121	ycfZ	putative inner membrane protein	-0.751288999	4.785415439	0.033133194	0.083399686
b1918	tcyL	cystine ABC transporter membrane subunit	-0.752611754	7.33836688	2.44E-07	2.40E-06
b1054	lpxL	LAUROYLACYLTRAN-MONOMER	-0.753500256	5.879879798	0.001543524	0.005993544
b1156	tfaE	e14 prophage; putative tail fiber assembly protein TfaE	-0.755295267	4.227636951	0.062684633	0.138332127
b1306	pspC	phage shock protein C	-0.758173534	5.910487494	0.004127349	0.014147708
b0326	yahL	uncharacterized protein YahL	-0.758201055	5.299427531	0.01504667	0.042393777
b4346	mcrB	5-methylcytosine-specific restriction enzyme subunit McrB	-0.758940761	7.599446439	1.52E-08	1.87E-07
b1253	yciA	acyl-CoA thioesterase	-0.759818093	7.072312513	1.75E-06	1.45E-05
b4100	phnH	carbon-phosphorus lyase core complex subunit PhnH	-0.761055695	4.29505392	0.070840536	0.152343384
b1580	rspB	putative zinc-binding dehydrogenase RspB	-0.761556941	5.34274414	0.057128428	0.128266072
b2792	yqcC	DUF446 domain-containing protein YqcC	-0.762542486	5.441961212	0.00769038	0.024254113
b4101	phnG	carbon-phosphorus lyase core complex subunit PhnG	-0.762902149	4.093410928	0.062468193	0.138036573
b3893	fdoH	formate dehydrogenase O subunit β	-0.763178011	9.235016121	0.000462347	0.002068146
b2089	insF-5	IS3 element protein InsF	-0.764675089	7.76747625	2.80E-06	2.23E-05
b1131	purB	adenylosuccinate lyase	-0.767025978	7.806125401	3.61E-09	4.90E-08
b2408	yfeN	conserved outer membrane protein YfeN	-0.767242386	4.684065468	0.036660461	0.09042231
b3779	gpp	guanosine-5'-triphosphate,3'-diphosphate phosphatase	-0.768274168	9.626490073	0.000406975	0.001846846
b0817	mntR	DNA-binding transcriptional dual regulator MntR	-0.768734575	7.594573795	0.000120059	0.000656014
b1940	fliH	flagellar biosynthesis protein FliH	-0.76915782	4.760325407	0.066136795	0.144265785
b1086	rluC	23S rRNA pseudouridine9552504	-0.770033678	8.098943131	1.60E-06	1.34E-05
b2799	fucO	L-1,2-propanediol oxidoreductase	-0.770427811	5.897355199	0.002368189	0.008801832
b0059	rapA	RNA polymerase-binding ATPase and RNAP recycling factor	-0.770646932	8.542314582	9.91E-06	7.00E-05
b1320	ycjW	putative LacI-type DNA-binding transcriptional regulator YcjW	-0.7719442	5.60332229	0.010815675	0.032395662
b1876	argS	arginine—tRNA ligase	-0.774557841	9.16748923	8.47E-06	6.08E-05
b0737	tolQ	Tol-Pal system protein TolQ	-0.77655072	8.567667269	3.54E-05	0.000221455
b4526	ydaE	Rac prophage; zinc-binding protein	-0.777223214	2.375056061	0.403753915	0.563392394
b3115	tdcD	propionate kinase	-0.777463117	4.870979673	0.139586405	0.259047489
b0423	thiI	tRNA uridine 4-sulfurtransferase	-0.777815557	8.803153199	1.39E-06	1.18E-05
b3547	yhjX	putative pyruvate transporter	-0.778198415	9.158566446	7.51E-05	0.000440988
b4678	yoeI	uncharacterized protein YoeI	-0.780519255	5.433360579	0.011331948	0.033736043
b1069	murJ	lipid II flippase MurJ	-0.78202218	7.054562907	7.78E-07	6.94E-06
b1196	ycgY	uncharacterized protein YcgY	-0.782532432	4.210505598	0.096541324	0.195070912
b2045	wcaK	putative colanic acid biosynthesis pyruvyl transferase WcaK	-0.78333214	4.790243566	0.033551761	0.084405143
b2156	lysP	LYSP-MONOMER	-0.784922098	8.298240034	3.62E-07	3.43E-06
b1363	trkG	Rac prophage; K+ transporter TrkG	-0.785643625	8.302133824	2.08E-07	2.06E-06
b0736	ybgC	esterasethioesterase	-0.785988476	7.945086915	4.80E-06	3.62E-05
b2845	yqeG	putative transporter YqeG	-0.787648599	7.473093983	1.07E-06	9.28E-06
b2214	ftp	FAD:protein FMN transferase	-0.787735297	7.241401638	2.38E-06	1.92E-05
b0724	sdhB	succinate:quinone oxidoreductase, iron-sulfur cluster binding protein	-0.788709888	8.460033444	1.65E-05	0.000110233

b4321	gntP	fructuronate transporter	-0.791087495	4.756842778	0.026346326	0.068787124
b0363	yaiP	putative glucosyltransferase	-0.791566195	4.700093832	0.038547172	0.094181387
b3446	yrhB	putative heat shock chaperone	-0.794401377	5.517691354	0.008594574	0.0266468
b3424	glpG	EG10397-MONOMER	-0.796217742	7.928718221	1.02E-06	8.89E-06
b1252	tonB	Ton complex subunit TonB	-0.798247414	7.463409148	7.31E-07	6.55E-06
b1878	flhE	flagellar protein	-0.799881431	3.907224722	0.06812865	0.148025585
b2967	pheV	tRNA-Phe(GAA)	-0.80179624	5.932449047	0.00232191	0.00865083
b3074	ygjH	putative tRNA-binding protein YgjH	-0.80283283	4.591960902	0.180154783	0.314505087
b3857	mobA	molybdenum cofactor guanylyltransferase	-0.804899344	8.71087881	0.000731747	0.003106224
b3807	cyaY	frataxin CyaY	-0.805191301	9.079494179	0.000597829	0.002610697
b3828	metR	DNA-binding transcriptional dual regulator MetR	-0.807154265	8.837132089	0.002476568	0.009104118
b2852	ygeH	putative transcriptional regulator YgeH	-0.807403863	5.628975251	0.017396112	0.04809257
b2033	wbbJ	putative acyl transferase	-0.809154587	8.72970492	4.64E-06	3.50E-05
b0503	selU	EG11768-MONOMER	-0.809929944	7.866144472	9.35E-08	9.81E-07
b3824	rhtB	L-homoserineL-homoserine lactone	-0.810807232	8.955776134	0.001047221	0.004250684
b4754	yqfI	protein YqfI	-0.811344779	6.295883753	0.000311126	0.001475423
b0898	ycaD	putative transporter YcaD	-0.815159454	9.005779409	2.68E-07	2.63E-06
b4421	ldrB	small toxic polypeptide LdrB	-0.816391579	5.285464587	0.008907392	0.027409799
b0005	yaaX	DUF2502 domain-containing protein YaaX	-0.816651362	6.11470216	0.000207348	0.001042643
b4439	micF	small regulatory RNA MicF	-0.816665577	5.880310198	0.000926346	0.003833008
b4363	yjjB	putative succinate exporter YjjB	-0.816894115	2.741984954	0.201614509	0.34130677
b4315	fimI	putative fimbrial protein FimI	-0.819056425	7.419468115	0.001011168	0.004129793
b1770	ydjF	putative DNA-binding transcriptional regulator YdjF	-0.819167325	6.59425647	1.15E-05	7.95E-05
b4732	ykiD	protein YkiD	-0.822025915	4.669875939	0.129197892	0.24460064
b2649	ypjB	DUF5508 domain-containing protein YpjB	-0.822838804	5.575791237	0.012441576	0.036619707
b4006	purH	bifunctional AICAR transformylaseIMP cyclohydrolase	-0.823378802	8.768079692	0.002041115	0.007695577
b4480	hdfR	DNA-binding transcriptional dual regulator HdfR	-0.823993507	9.042185874	0.001047507	0.004250684
b1630	rsxD	SoxR [2Fe-2S] reducing system protein RsxD	-0.824723775	7.286887922	4.86E-07	4.48E-06
b1943	fliK	flagellar hook-length control protein	-0.825511444	4.775463539	0.03499069	0.087326375
b1231	tyrT	tRNA-Tyr(GUA)	-0.82600723	6.02414996	0.000316491	0.001492852
b3635	mutM	DNA-formamidopyrimidine glycosylase	-0.827526148	7.198106726	4.62E-08	5.15E-07
b4083	yjcS	linear primary-alkylsulfatase	-0.828762568	4.325452192	0.033853181	0.084921475
b3856	mobB	molybdopterin-guanine dinucleotide biosynthesis adaptor protein	-0.828841245	8.681441798	0.000617116	0.00268166
b4232	fbp	fructose-1,6-bisphosphatase 1	-0.832432274	8.381934893	6.54E-07	5.94E-06
b1294	sapA	putative periplasmic binding protein SapA	-0.833755016	7.864717909	2.47E-08	2.90E-07
b3626	waaJ	UDP-glucose:(glucosyl)LPS α-1,2-glucosyltransferase	-0.834230121	8.6737997	9.54E-07	8.39E-06
b1229	tpr	protamine-like protein	-0.834441622	4.493999358	0.035150069	0.087627078
b2014	plaP	YEEF-MONOMER	-0.835535904	8.194763545	1.42E-05	9.63E-05
b2073	yegL	IPR002035 domain-containing protein YegL	-0.836017208	3.721764324	0.092291955	0.188317219
b0459	maa	maltose O-acetyltransferase	-0.837470065	6.873043355	3.12E-07	2.98E-06
b0914	msbA	ATP-binding lipopolysaccharide transport protein	-0.83822208	8.838409191	1.24E-06	1.06E-05
b3104	yhaI	putative inner membrane protein	-0.838987396	5.020043932	0.105852243	0.209568454
b0150	fhuA	ferrichrome outer membrane transporterphage receptor	-0.840484551	8.148953178	1.11E-06	9.61E-06
b2144	sanA	DUF218 domain-containing protein SanA	-0.841036665	6.166498676	9.51E-05	0.000544438
b3630	waaP	lipopolysaccharide core heptose (I) kinase	-0.842447329	7.71724398	7.49E-11	1.32E-09
b2066	udk	uridinecytidine kinase	-0.843735223	7.822591908	1.25E-08	1.59E-07
b0858	ybjO	putative inner membrane protein	-0.843755097	6.258379553	0.000368138	0.001704291

b1305	pspB	phage shock protein B	-0.844774012	5.798668653	0.000675043	0.002904791
b1523	yneG	DUF4186 domain-containing protein YneG	-0.846188019	6.718413166	5.90E-06	4.37E-05
b1185	dsbB	protein thiol:quinone oxidoreductase DsbBsubreducedsub	-0.846241304	6.916811235	9.29E-07	8.21E-06
b3892	fdoI	formate dehydrogenase O subunit γ	-0.850416116	9.138598674	0.000218041	0.001084068
b0987	gfcA	threonine-rich inner membrane protein GfcA	-0.851022524	3.894711602	0.076247708	0.161532453
b4621	yjbS	uncharacterized protein YjbS	-0.851651111	4.352102814	0.030401053	0.077763991
b1629	rsxC	SoxR [2Fe-2S] reducing system protein RsxC	-0.853331005	8.668640336	3.21E-06	2.53E-05
b2034	wbbI	β-1,6-galactofuranosyltransferase	-0.856563427	9.249482522	2.96E-07	2.85E-06
b3002	yqhA	uncharacterized protein YqhA	-0.859366642	7.333909323	2.23E-06	1.81E-05
b2037	rfbX	polyisoprenol-linked O-antigen repeat unit flippase	-0.860230248	9.413595133	1.54E-07	1.56E-06
b1414	ydcF	DUF218 domain-containing protein YdcF	-0.862289654	7.109751997	4.38E-08	4.93E-07
b3629	waaS	lipopolysaccharide core biosynthesis protein WaaS	-0.862860036	8.211979252	6.96E-09	9.21E-08
b3845	fadA	3-ketoacyl-CoA thiolase	-0.863924616	8.970741592	0.000765808	0.003237142
b3937	yiiX	putative lipid binding hydrolase	-0.86432283	8.856525424	0.000302316	0.001442947
b3090	ygjV	inner membrane protein	-0.864673805	6.345223044	0.000187112	0.000957478
b0797	rhlE	ATP-dependent RNA helicase RhlE	-0.864706786	7.867542506	4.26E-09	5.73E-08
b3499	rlmJ	23S rRNA m6A2030 methyltransferase	-0.864861199	7.404881994	2.02E-08	2.42E-07
b4750	ynfS	Qin prophage; protein YnfS	-0.865059973	5.926383329	0.000720542	0.00306769
b1704	aroH	3-deoxy-7-phosphoheptulonate synthase, Trp-sensitive	-0.865656788	8.268346629	3.53E-08	4.05E-07
b2322	yfcJ	putative transporter YfcJ	-0.866678142	6.111810265	0.000337509	0.001581851
b3107	yhaL	uncharacterized protein YhaL	-0.868011362	4.880516407	0.065265224	0.142717169
b4108	yjdM	zinc ribbon domain-containing protein YjdM	-0.869992251	5.55818288	0.005327532	0.017645202
b1731	cedA	cell division modulator	-0.870948652	4.735940671	0.013989509	0.040158441
b3622	waaL	O-antigen ligase	-0.871901718	9.057358625	7.12E-08	7.69E-07
b3628	waaB	UDP-D-galactose:(glucosyl)lipopolysaccharide-1,6-D-galactosyltransfera	-0.871943704	8.680162988	1.44E-07	1.47E-06
b3396	mrcA	peptidoglycan glycosyltransferase peptidoglycan DD-transpeptidase Mr	-0.872000408	8.339029369	2.46E-06	1.98E-05
b4747	yneP	protein YneP	-0.875133942	4.167491466	0.046566835	0.109241539
b4487	yjdP	protein YjdP	-0.87634625	6.873071094	2.37E-05	0.000153587
b3932	hslV	peptidase component of the HslVU protease	-0.878795393	9.216481142	0.00035104	0.001634854
b3920	yiiQ	DUF1454 domain-containing protein YiiQ	-0.879273926	8.786237219	0.000514984	0.002273656
b3955	eptC	phosphoethanolamine transferase EptC	-0.882046294	9.668941428	1.06E-05	7.41E-05
b4291	fecA	ferric citrate outer membrane transporter	-0.882970387	6.002667702	0.001691319	0.006505196
b4731	ykiC	protein YkiC	-0.883755296	3.986423703	0.266445019	0.418631587
b0749	lysQ	tRNA-Lys(UUU)	-0.884557878	5.364956098	0.001723452	0.006610808
b4176	yjeT	DUF2065 domain-containing protein YjeT	-0.885968338	5.530431758	0.002166021	0.008124881
b4454	rdlD	antisense regulatory RNA RdlD	-0.886272818	5.891841227	0.000183363	0.00094243
b4730	yahV	protein YahV	-0.886325835	5.266485198	0.0035357	0.01239882
b2035	wbbH	putative O-antigen polymerase	-0.886716925	9.207437636	1.92E-07	1.90E-06
b3627	waaO	UDP-D-glucose:(glucosyl)LPS α-1,3-glucosyltransferase	-0.889700508	8.683647833	1.09E-07	1.13E-06
b3594	yibA	putative lyase containing HEAT-repeat	-0.890003183	6.793789178	1.96E-06	1.62E-05
b0157	yadS	conserved inner membrane protein YadS	-0.890983282	6.754001871	1.09E-06	9.50E-06
b4097	phnK	carbon-phosphorus lyase subunit PhnK	-0.8915052	4.509484006	0.014501622	0.0413329
b0961	yccF	conserved inner membrane protein YccF	-0.892123051	5.672474263	0.001237563	0.00490911
b1835	rsmF	16S rRNA m5C1407 methyltransferase	-0.892343092	6.670262788	7.52E-06	5.47E-05
b0550	rusA	DLP12 prophage; crossover junction endodeoxyribonuclease RusA	-0.893832193	2.139600756	0.353808166	0.514174803
b0592	fepB	ferric enterobactin ABC transporter periplasmic binding protein	-0.894952122	5.100484422	0.013369413	0.038781838
b3639	dfp	fused 4'-phosphopantothenoylcysteine decarboxylase and phosphopantc	-0.89501669	8.101912775	7.71E-10	1.15E-08

b4686	yshB	uncharacterized protein YshB	-0.895353714	8.853738477	0.000679065	0.002919253
b2844	yqeF	putative acyltransferase	-0.89594371	6.607581858	6.09E-05	0.000365603
b0546	ybcM	DLP12 prophage; putative DNA-binding transcriptional regulator	-0.897249555	7.331012996	1.53E-07	1.56E-06
b1365	ynaK	Rac prophage; ParB-like nuclease domain-containing protein YnaK	-0.897827386	4.476779458	0.012725726	0.037257348
b2185	rplY	50S ribosomal subunit protein L25	-0.898481819	7.494419844	3.11E-10	4.93E-09
b4463	ygcU	putative FAD-containing dehydrogenase	-0.898863895	4.868731835	0.007870095	0.024642886
b1445	ortT	orphan toxin OrtT	-0.899183199	5.423078151	0.00489711	0.016354569
b3562	yiaA	conserved inner membrane protein YiaA	-0.900954282	4.985704283	0.016169827	0.04509778
b3085	ygjP	putative metal-dependent hydrolase	-0.902482538	6.211446922	0.000104149	0.000585011
b2145	yeiS	DUF2542 domain-containing protein YeiS	-0.90313218	5.059716504	0.010026444	0.030423883
b3089	sstT	YGJU-MONOMER	-0.905335257	7.755802525	4.03E-10	6.29E-09
b2934	cmtB	mannitol-specific PTS enzyme IIA component CmtB	-0.907264281	3.912775771	0.111852802	0.218799344
b1565	ydfV	Qin prophage; protein YdfV	-0.913445624	5.135412796	0.006599043	0.021235259
b0576	pheP	PHEP-MONOMER	-0.91389704	6.335962408	7.64E-06	5.55E-05
b1880	flhB	flagellar biosynthesis protein FlhB	-0.915816677	4.401874246	0.039758029	0.096234484
b0610	rnk	nucleoside diphosphate kinase regulator	-0.917876229	6.634605732	1.97E-06	1.63E-05
b3934	cytR	DNA-binding transcriptional repressor CytR	-0.918830904	9.14548339	8.31E-05	0.000481508
b3935	priA	primosome factor N'	-0.918980258	9.098111917	0.00010818	0.00060305
b0315	pdeL	DNA-binding transcriptional activatorc-di-GMP phosphodiesterase PdeL	-0.919628504	6.749130158	2.77E-06	2.20E-05
b0458	ylaC	putative inner membrane protein	-0.919727092	6.316985209	1.55E-05	0.00010444
b3183	obgE	GTPase ObgE	-0.919813416	9.067739513	2.33E-08	2.75E-07
b2181	yejG	protein YejG	-0.920243686	7.130948168	6.33E-08	6.90E-07
b2791	truC	tRNA pseudouridine65 synthase	-0.92308634	6.565298815	1.04E-05	7.28E-05
b3933	ftsN	cell division protein FtsN	-0.926396943	9.277774276	1.93E-05	0.000127449
b2562	yfhL	putative 4Fe-4S cluster-containing protein YfhL	-0.92867169	7.061401225	4.48E-08	5.02E-07
b1527	yneK	protein YneK	-0.929030274	4.272888515	0.016143338	0.045052364
b3058	folB	dihydroneopterin aldolase	-0.929687083	6.469176244	7.80E-05	0.000454045
b1269	rluB	23S rRNA pseudouridine2605 synthase	-0.92976962	7.93863091	1.22E-10	2.07E-09
b2273	yfbN	uncharacterized protein YfbN	-0.930852211	5.317028438	0.005486704	0.018104482
b1132	hflD	lysogenization regulator	-0.932962085	7.402953228	1.45E-08	1.80E-07
b0251	yafY	CP4-6 prophage; inner membrane lipoprotein YafY	-0.933286974	4.937396626	0.009418028	0.02881538
b3069	ileX	tRNA-Ile(CAU)	-0.934455525	4.926888607	0.093334383	0.18980714
b0567	ybcH	DUF4434 domain-containing protein YbcH	-0.934565849	6.137454741	4.44E-05	0.000273676
b0532	sfmD	putative fimbrial usher protein SfmD	-0.936205548	3.961560787	0.041650892	0.099939504
b0555	rrrD	DLP12 prophage; lysozyme	-0.93650896	4.817298038	0.014634884	0.041542466
b1491	yddW	putative lipoprotein YddW	-0.938825223	7.708325901	4.33E-13	1.14E-11
b0269	yagF	CP4-6 prophage; D-xylonate dehydratase	-0.941110627	6.682169961	5.06E-05	0.00030883
b1917	tcyN	cystine ABC transporter ATP binding subunit	-0.946489302	7.642185162	1.22E-11	2.46E-10
b3801	aslA	ARYLSULFAT-MONOMER	-0.946813693	8.765127184	0.000669023	0.00288451
b1767	ansA	L-asparaginase 1	-0.947552153	7.576861218	4.78E-11	8.76E-10
b3995	rsd	regulator of sigma D	-0.949639926	8.764949793	0.000418273	0.001894026
b3751	rbsB	ribose ABC transporter periplasmic binding protein	-0.950697402	8.324829698	5.63E-08	6.18E-07
b0570	cusS	sensory histidine kinase CusS - phosphorylated	-0.951563786	7.031275244	1.04E-06	9.04E-06
b2650	ypjC	DUF5507 domain-containing protein YpjC	-0.952588782	5.214121182	0.010511142	0.031612188
b0964	yccT	DUF2057 domain-containing protein YccT	-0.95363614	4.828447009	0.006624105	0.021284878
b4622	ytcA	putative lipoprotein YtcA	-0.954958178	3.990678918	0.038817263	0.094684098
b4266	idnO	5-keto-D-gluconate 5-reductase	-0.955162551	4.14885583	0.025025885	0.06576743

b1481	bdm	biofilm-dependent modulation protein	-0.955288185	4.825107054	0.008908961	0.027409799
b0939	elfD	putative fimbrial chaperone ElfD	-0.955827932	3.063230518	0.118117671	0.228723473
b0412	yajI	putative lipoprotein YajI	-0.95728221	6.032128357	6.72E-05	0.000399233
b4588	ylcH	DLP12 prophage; uncharacterized protein YlcH	-0.961076451	3.172509179	0.152479406	0.278065501
b3842	rfaH	transcription antiterminator RfaH	-0.962327916	8.97981657	0.000150674	0.000801474
b3773	ilvY	DNA-binding transcriptional dual regulator IlvY	-0.962505841	8.892107477	0.001165323	0.004660235
b3625	waaY	lipopolysaccharide core heptose (II) kinase	-0.962724601	8.063750358	2.75E-09	3.80E-08
b3470	tusA	sulfur transfer protein TusA	-0.965079196	6.760094202	1.70E-08	2.06E-07
b2350	yfdG	CPS-53 (KpLE1) prophage; putative bactoprenol-linked glucose transloca	-0.96657197	7.310123953	2.64E-09	3.67E-08
b3946	fsaB	fructose-6-phosphate aldolase 2	-0.966909895	8.634752922	0.000551746	0.00242384
b1133	mnmA	tRNA-specific 2-thiouridylase	-0.967282165	8.660857015	1.43E-07	1.47E-06
b2846	yqeH	putative LuxR family transcriptional regulator YqeH	-0.967369344	6.746799278	2.73E-06	2.17E-05
b3898	frvX	peptidase M42 family protein FrvX	-0.967451281	8.631953783	0.000514918	0.002273656
b3954	yijO	putative DNA-binding transcriptional regulator YijO	-0.968124241	8.728334693	0.000259175	0.001267172
b0406	tgt	tRNA-guanine transglycosylase	-0.969859542	8.862174572	7.95E-08	8.56E-07
b1307	pspD	phage shock protein D	-0.971868773	5.251041404	0.006810339	0.021835619
b1803	yeaX	carnitine monooxygenase subunit YeaX	-0.97353661	5.39646079	0.00100337	0.00410554
b3397	nudE	ADP-sugar diphosphatase NudE	-0.978421054	7.610478378	3.85E-11	7.16E-10
b0252	yafZ	CP4-6 prophage; DUF932 domain-containing protein YafZ	-0.98140715	6.098330603	9.42E-06	6.69E-05
b0905	ycaO	ribosomal protein S12 methylthiotransferase accessory factor YcaO	-0.982381082	7.143391566	1.57E-08	1.91E-07
b3846	fadB	dodecenoyl-CoA δ-isomerase, enoyl-CoA hydratase, 3-hydroxybuty	-0.983192826	8.798548337	0.00068274	0.002924193
b3818	yigG	inner membrane protein	-0.98466687	8.764711918	0.000407017	0.001846846
b4691	sroH	small RNA SroH	-0.984798174	8.76527845	0.000131044	0.000710759
b3618	htrL	protein HtrL	-0.986832178	8.379581507	2.29E-09	3.21E-08
b2701	mltB	membrane-bound lytic murein transglycosylase B	-0.988234831	6.970052002	3.95E-07	3.71E-06
b2126	btsS	high-affinity pyruvate receptor	-0.990886963	7.14057335	8.19E-10	1.22E-08
b3994	thiC	phosphomethylpyrimidine synthase	-0.992860419	8.54478697	0.000392562	0.001796023
b0568	nfrA	bacteriophage N4 receptor, outer membrane protein	-0.993063803	8.090501226	4.69E-12	1.02E-10
b3817	yigF	DUF2628 domain-containing protein YigF	-0.993143985	8.802049927	0.000250524	0.001230329
b4482	yigE	DUF2233 domain-containing protein YigE	-0.99455462	8.888337414	0.000396124	0.001810444
b0818	ybiR	putative transporter YbiR	-0.994929883	7.28791029	1.29E-09	1.89E-08
b2868	xdhC	putative xanthine dehydrogenase iron-sulfur-binding subunit XdhC	-0.99624864	4.776574217	0.026631299	0.06941596
b1805	fadD	long-chain-fatty-acid—CoA ligase	-0.998342118	7.186910944	9.68E-11	1.69E-09
b1701	fadK	short chain acyl-CoA synthetase	-0.99910238	5.579747606	0.000307723	0.001464008
b3880	yihS	sulfoquinovose isomerase	-0.999556747	8.636376793	0.000374708	0.001729116
b3337	bfd	bacterioferritin-associated ferredoxin	-1.002507915	6.028605579	0.000169974	0.000887041
b2980	glcC	DNA-binding transcriptional dual regulator GlcC	-1.010645286	6.056429684	0.000247924	0.001220274
b3775	ppiC	peptidyl-prolyl cis-trans isomerase C	-1.014141958	8.93650413	0.000301135	0.001438866
b4317	fimD	type I fimbriae usher protein	-1.014782672	7.879318261	8.21E-05	0.000476434
b0314	betT	BETT-MONOMER	-1.015250933	7.727934745	1.07E-10	1.84E-09
b1807	tsaB	N6-L-threonylcarbamoyladenine synthase, TsaB subunit	-1.015666828	7.380192929	4.68E-11	8.63E-10
b1628	rsxB	SoxR [2Fe-2S] reducing system protein RsxB	-1.021083297	6.909923452	3.59E-08	4.11E-07
b3930	menA	DMK-MONOMER	-1.022919388	9.000692902	4.27E-05	0.000264335
b2106	rcnA	Ni2+	-1.023005922	6.037776989	1.46E-05	9.89E-05
b4241	treR	DNA-binding transcriptional repressor TreR	-1.024412811	6.637347148	1.09E-05	7.60E-05
b1728	ydjM	putative inner membrane protein regulated by LexA	-1.025139581	4.956410427	0.003987903	0.013803034
b3765	yifB	putative magnesium chelatase YifB	-1.026087785	8.849904335	0.000290678	0.001397978

b3704	rnpA	RNase P protein component	-1.02736783	7.720841632	5.22E-12	1.12E-10
b2971	yghG	lipoprotein YghG	-1.029003079	4.169971471	0.06172495	0.136599325
b1639	mliC	inhibitor of c-type lysozyme, putative lipoprotein	-1.029527686	6.638587786	8.14E-08	8.73E-07
b1499	ydeO	DNA-binding transcriptional dual regulator YdeO	-1.030183607	3.610148072	0.055424597	0.125209545
b4680	ypdK	uncharacterized membrane protein YpdK	-1.031079738	6.289851086	2.19E-06	1.78E-05
b1435	rlhA	23S rRNA 5-hydroxycytidine C2501 synthase	-1.031296515	8.418857976	1.57E-09	2.24E-08
b3707	tnaC	tnaAB operon leader peptide	-1.032571446	5.22367739	0.006041933	0.019657431
b0731	mngA	HRSA-MONOMER	-1.033659743	3.463354865	0.051207862	0.11799724
b4720	ytiC	protein YtiC	-1.034860994	5.675374908	0.00115869	0.00464212
b2517	rlmN	23S rRNA m2A2503 methyltransferase	-1.03600316	8.447695132	1.05E-09	1.56E-08
b2801	fucP	L-fucose:H+ symporter	-1.037252131	4.867402818	0.012790074	0.037396144
b3184	yhbE	inner membrane protein YhbE	-1.038668543	8.809828958	1.55E-10	2.59E-09
b0268	yagE	CP4-6 prophage; putative 2-dehydro-3-deoxygluconate aldolase	-1.039499787	6.12723089	9.92E-05	0.000564641
b2032	wbbK	putative lipopolysaccharide biosynthesis protein	-1.039550224	9.067905554	4.86E-10	7.52E-09
b1655	mepH	G6892-MONOMER	-1.039871412	6.760483016	2.72E-07	2.65E-06
b3633	waaA	KDO transferase	-1.042172091	7.922679589	2.59E-13	7.00E-12
b3819	rarD	putative transporter RarD	-1.042290206	8.92815844	8.75E-05	0.000505663
b1632	rsxE	SoxR [2Fe-2S] reducing system protein RsxE	-1.043941933	6.797668691	1.57E-09	2.24E-08
b4353	yjiX	PF04328 family protein YjiX	-1.04428689	6.135302467	9.39E-06	6.69E-05
b1786	dgcJ	putative diguanylate cyclase DgcJ	-1.045559176	7.755009925	1.22E-09	1.79E-08
b1633	nth	endonuclease III	-1.047573862	6.562179234	5.51E-08	6.07E-07
b3878	yihQ	sulfoquinovosidase	-1.04858611	8.56634563	0.000699573	0.002987053
b4359	opgB	phosphoglycerol transferase II	-1.05318436	8.763138243	1.66E-10	2.76E-09
b1124	potC	spermidine preferential ABC transporter membrane subunit PotC	-1.053357626	7.247909385	1.23E-08	1.56E-07
b3947	ptsA	putative PTS multiphosphoryl transfer protein PtsA	-1.053556669	8.618750992	0.000178157	0.000918883
b4320	fimH	type 1 fimbriae D-mannose specific adhesin	-1.05414873	6.44381988	0.000186318	0.000955395
b3425	glpE	thiosulfate sulfurtransferase GlpE	-1.056894818	6.730473595	6.26E-05	0.000375375
b2059	wcaA	putative colanic acid biosynthesis glycosyl transferase WcaA	-1.057558201	4.206359718	0.019024246	0.051943134
b3962	sthA	soluble pyridine nucleotide transhydrogenase	-1.057919758	9.892661679	8.76E-05	0.000505663
b2466	ypfG	DUF1176 domain-containing protein YpfG	-1.058163139	7.487860196	1.02E-11	2.10E-10
b3877	yihP	putative 2,3-dihydroxypropane-1-sulfonate export protein	-1.05836345	8.574993492	0.000283551	0.001377201
b3882	yihU	3-sulfolactaldehyde reductase	-1.061322533	8.551784879	0.000299238	0.001432904
b2869	ygeV	putative σ54-dependent transcriptional regulator YgeV	-1.063401004	6.882239002	0.000159412	0.00084187
b3899	frvB	putative PTS enzyme IIBC component FrvB	-1.066411828	8.560907261	0.000299075	0.001432904
b1071	flgM	anti-sigma factor for FliA (σ28)	-1.0669493	4.202957483	0.014127116	0.04044826
b3897	frvR	putative transcriptional regulator FrvR	-1.071046408	8.604217944	0.000165391	0.000868252
b3944	yijF	DUF1287 domain-containing protein YijF	-1.073521558	8.656451411	0.000141408	0.000757693
b3907	rhaT	rhamnoselyxose:H+	-1.074879293	8.586018976	0.000223531	0.00110638
b0603	ybdO	putative LysR family DNA-binding transcriptional regulator YbdO	-1.077305938	5.127854827	0.002428411	0.008966312
b3624	waaZ	lipopolysaccharide core biosynthesis protein WaaZ	-1.078892424	7.992487988	2.78E-12	6.28E-11
b3881	yihT	6-deoxy-6-sulfofructose-1-phosphate aldolase	-1.082999785	8.57265082	0.000257888	0.001262281
b3876	yihO	putative sulfoquinovose transporter	-1.084692795	8.569671061	0.00029401	0.001412466
b4458	oxyS	small regulatory RNA OxyS	-1.086392877	8.643829765	0.000112947	0.000624548
b3901	rhaM	L-rhamnose mutarotase	-1.089530908	8.536092386	0.000305205	0.001455163
b3879	yihR	putative aldose 1-epimerase YihR	-1.091470014	8.53640423	0.000346425	0.001616772
b0368	tauD	taurine dioxygenase	-1.09252368	6.045379559	1.04E-05	7.28E-05
b1794	dgcP	diguanylate cyclase DgcP	-1.096564062	7.761400343	1.38E-13	3.83E-12

b2124	yehS	DUF1456 domain-containing protein YehS	-1.097472731	7.011266033	5.80E-10	8.89E-09
b0656	insH-3	IS5 transposase and trans-activator	-1.097842547	7.182402533	0.000374805	0.001729116
b0358	yaiO	outer membrane protein YaiO	-1.098894798	4.46361631	0.019799999	0.053695943
b1287	yciW	putative oxidoreductase	-1.100061686	7.715570356	3.45E-12	7.66E-11
b2964	nupG	nucleoside:H+ symporter NupG	-1.104107817	6.838178294	1.33E-08	1.67E-07
b1941	fliI	flagellum-specific ATP synthase FliI	-1.104515267	4.764727914	0.008287898	0.025768359
b4114	eptA	phosphoethanolamine transferase EptA	-1.105140522	7.262696869	6.95E-11	1.23E-09
b1937	fliE	flagellar basal-body protein FliE	-1.10691805	3.762104196	0.016214257	0.045193148
b3900	frvA	putative PTS enzyme IIA component FrvA	-1.10942997	8.53521028	0.000338618	0.001585271
b0580	ybdJ	DUF1158 domain-containing protein YbdJ	-1.113681079	5.083961833	0.000918829	0.003809149
b3471	yhhQ	putative queuosine precursor transporter	-1.113801423	5.744156505	0.000965333	0.0039646
b0405	queA	EG10812-MONOMER	-1.114900583	7.815300032	7.51E-14	2.20E-12
b3902	rhaD	rhamnulose-1-phosphate aldolase	-1.117984813	8.517980575	0.000313694	0.001484413
b1947	fliO	flagellar biosynthesis protein FliO	-1.118467813	4.269408385	0.01635135	0.045517787
b4469	ygiQ	radical SAM superfamily protein YgiQ	-1.120233469	8.441829415	6.25E-10	9.49E-09
b3903	rhaA	L-rhamnose isomerase	-1.123421719	8.511644017	0.00032543	0.001531741
b3875	ompL	putative outer membrane porin L	-1.123434929	8.527510256	0.000284901	0.001382238
b3904	rhaB	RHAMNULOKIN-MONOMER	-1.124063521	8.512077489	0.000323451	0.001524052
b4345	mcrC	5-methylcytosine-specific restriction enzyme subunit McrC	-1.125454743	6.914802805	3.78E-11	7.07E-10
b3063	ttdT	YGJE-MONOMER	-1.127942128	6.523842224	1.20E-06	1.03E-05
b3061	ttdA	L(+)-tartrate dehydratase subunit α	-1.129308091	4.474668306	0.075293608	0.159971742
b0835	rimO	ribosomal protein S12 methylthiotransferase RimO	-1.129616158	7.297052355	1.76E-11	3.41E-10
b1939	fliG	flagellar motor switch protein FliG	-1.129658929	5.597452276	0.000424746	0.001911575
b2533	suhB	inositol-phosphate phosphatase	-1.130259804	7.947842407	1.38E-12	3.38E-11
b1722	ydiY	acid-inducible putative outer membrane protein YdiY	-1.133938175	8.074752886	3.14E-14	9.91E-13
b0517	allD	ureidoglycolate dehydrogenase	-1.134138775	2.68366773	0.076757005	0.162299893
b1954	dsrA	small regulatory RNA DsrA	-1.137858359	4.266841824	0.01038583	0.031342065
b1366	ydaY	Rac prophage; putative uncharacterized protein YdaY	-1.138689904	4.426739379	0.004625888	0.015578409
b1627	rsxA	SoxR [2Fe-2S] reducing system protein RsxA	-1.139107931	6.516585742	1.95E-08	2.35E-07
b2094	gatA	galactitol-specific PTS enzyme IIA component	-1.139915355	8.362105672	6.63E-11	1.18E-09
b4721	ytiD	protein YtiD	-1.142766434	6.832515925	4.80E-07	4.43E-06
b4625	symR	small regulatory RNA SymR	-1.143184845	5.212884429	0.000125507	0.000683248
b4705	mntS	small protein MntS	-1.146029362	5.76812841	6.95E-05	0.000410403
b2147	preA	NAD-dependent dihydropyrimidine dehydrogenase subunit PreA	-1.150427684	6.745060307	9.20E-08	9.68E-07
b2943	galP	galactose:H+ symporter	-1.151106984	7.322800412	3.53E-13	9.34E-12
b4357	lgoR	putative DNA-binding transcriptional regulator LgoR	-1.152065504	4.38683541	0.00545391	0.018028773
b3014	yqhH	lipoprotein YqhH	-1.155581801	4.471538764	0.001862086	0.007087162
b2879	ssnA	putative aminohydrolase	-1.157278404	4.616122847	0.032177123	0.081457567
b2254	arnC	G7167-MONOMER	-1.158991664	6.438551485	1.42E-07	1.46E-06
b0723	sdhA	succinate:quinone oxidoreductase, FAD binding protein	-1.160414494	9.689759315	2.77E-09	3.82E-08
b1451	yncD	putative TonB-dependent outer membrane receptor	-1.162032959	7.737146212	0.00011687	0.00064177
b3820	yigI	putative thioesterase YigI	-1.162055287	9.053910166	2.44E-05	0.000157262
b0591	entS	YBDA-MONOMER	-1.16372089	4.592691209	0.003375696	0.011903913
b4557	yidD	membrane protein insertion efficiency factor	-1.163846874	7.166761057	1.10E-12	2.74E-11
b0815	opgE	G6418-MONOMER	-1.165604384	7.606056854	1.24E-12	3.06E-11
b0552	insH-2	DLP12 prophage; IS5 transposase and trans-activator	-1.166040641	6.915487186	3.13E-05	0.000197526
b4741	ymiC	protein YmiC	-1.166182112	5.563760362	0.00017076	0.000890089

b1047	opgC	protein required for succinyl modification of osmoregulated periplasmic	-1.168513081	6.753752333	3.12E-08	3.59E-07
b0249	ykfF	CP4-6 prophage; protein YkfF	-1.168816286	5.269064581	0.000859535	0.00359701
b2146	preT	NAD-dependent dihydropyrimidine dehydrogenase subunit PreT	-1.169317156	6.480896531	3.98E-06	3.04E-05
b3862	yihG	putative acyltransferase YihG	-1.171012819	8.755015853	6.37E-06	4.70E-05
b3050	yqiJ	putative inner membrane protein	-1.171491182	5.376323065	0.0002102	0.001054582
b3974	coaA	pantothenate kinase	-1.171706253	9.167347498	1.40E-06	1.18E-05
b0875	aqpZ	water channel AqpZ	-1.172823858	5.754437819	2.90E-05	0.000184888
b1743	spy	ATP-independent periplasmic chaperone	-1.175242195	7.672929554	3.25E-15	1.17E-13
b0589	fepG	ferric enterobactin ABC transporter membrane subunit FepG	-1.175296827	3.780263065	0.020116994	0.054371004
b0901	ycaK	putative NAD(P)H-dependent oxidoreductase YcaK	-1.180470247	6.234950587	2.35E-07	2.31E-06
b1820	yobD	conserved inner membrane protein YobD	-1.183652584	5.790986997	5.14E-06	3.87E-05
b3070	nfeF	NADPH-dependent ferric-chelate reductase	-1.1847707	5.716330477	0.00025272	0.001239329
b2274	yfbO	uncharacterized protein YfbO	-1.185945085	4.38419804	0.005978963	0.019495079
b1135	rluE	23S rRNA pseudouridine2457 synthase	-1.186675611	6.629254331	2.49E-09	3.48E-08
b1019	efeB	heme-containing peroxidasedeferrochelatase	-1.189399338	6.351321318	6.86E-07	6.18E-06
b1426	ydcH	protein YdcH	-1.194730303	6.542708498	1.47E-07	1.49E-06
b1421	trg	methyl-accepting chemotaxis protein Trg	-1.195196192	6.223616493	8.48E-08	9.03E-07
b0246	yafW	antitoxin of the YkfI-YafW toxin-antitoxin pair	-1.196538111	5.349061037	6.90E-05	0.000408294
b0113	pdhR	DNA-binding transcriptional dual regulator PdhR	-1.196876251	7.918288623	6.18E-07	5.64E-06
b4424	rdlC	small regulatory antisense RNA RdlC	-1.198229758	4.266909203	0.004963636	0.016525739
b0253	ykfA	CP4-6 prophage; putative GTP-binding protein YkfA	-1.199803416	6.310688791	2.84E-08	3.29E-07
b2944	yggI	protein YggI	-1.202458935	5.517829166	8.55E-06	6.13E-05
b3558	insK	IS150 conserved protein InsB	-1.203473399	6.216529544	1.74E-08	2.11E-07
b2253	arnB	G7166-MONOMER	-1.203777321	7.072160421	3.61E-10	5.67E-09
b3623	waaU	putative ADP-heptose:LPS heptosyltransferase 4	-1.205840222	8.416584611	1.38E-12	3.38E-11
b3673	emrD	multidrug efflux pump EmrD	-1.207629433	8.125866868	1.54E-11	3.03E-10
b2048	cpsG	PHOSMANMUT-MONOMER	-1.211408559	4.895757521	0.001341777	0.005279808
b3748	rbsD	D-ribose pyranase	-1.217011677	7.657582571	2.81E-18	1.43E-16
b4316	fimC	type 1 fimbriae periplasmic chaperone	-1.218350333	7.106551258	7.38E-06	5.38E-05
b2057	wcaC	putative colanic acid biosynthesis glycosyl transferase WcaC	-1.218452813	4.314581626	0.001971382	0.00745814
b3991	thiG	1-deoxy-D-xylulose 5-phosphate:thiol sulfurtransferase	-1.219458341	8.387581376	0.000148407	0.000790369
b0433	ampG	muropeptide:H+ symporter	-1.221879015	8.016887324	1.26E-16	5.27E-15
b1319	ompG	outer membrane porin G	-1.227928496	2.288914592	0.140533433	0.260476535
b1125	potB	spermidine preferential ABC transporter membrane subunit PotB	-1.22851642	7.433409254	7.93E-14	2.30E-12
b2049	cpsB	MANNPGUANYLTRANGDP-MONOMER	-1.229970784	4.867981951	0.000343386	0.001604286
b1158	pinE	e14 prophage; site-specific DNA recombinase	-1.230079493	3.436470611	0.032084864	0.081270611
b0217	yafT	lipoprotein YafT	-1.233633717	5.85273195	0.004054988	0.013932117
b1769	ydjE	putative transporter YdjE	-1.236933651	4.573606625	0.00112681	0.004530845
b3990	thiH	2-iminoacetate synthase	-1.238160977	8.408353802	0.000136742	0.00073534
b2125	btsR	Phosphorylated DNA-binding transcriptional activator BtsR	-1.241242103	6.577399093	6.93E-08	7.50E-07
b2774	ygcW	putative deoxygluconate dehydrogenase	-1.245326083	3.462858433	0.062695943	0.138332127
b1409	ynbB	putative CDP-diglyceride synthase	-1.247944691	1.572983008	0.385717857	0.546637241
b4733	ylcJ	protein YlcJ	-1.249362659	3.041178444	0.053315076	0.121646544
b2151	galS	DNA-binding transcriptional dual regulator GalS	-1.249633528	4.797277538	0.000181487	0.000934965
b0531	sfmC	putative fimbrial chaperone SfmC	-1.252041833	2.767770288	0.065309346	0.142742952
b0859	rlmC	23S rRNA m5U747 methyltransferase	-1.261153253	6.74096502	2.63E-10	4.25E-09
b4050	pspG	phage shock protein G	-1.265242399	5.955481932	0.000101212	0.000570692

b3079	ygjJ	protein YgjJ	-1.265496675	4.563814428	0.041932294	0.100450938
b1351	racC	Rac prophage; protein RacC	-1.266924223	3.692300787	0.013350098	0.038751271
b1026	insF-4	IS3 element protein InsF	-1.267070231	6.967514501	2.65E-11	5.06E-10
b3062	ttdB	L(+)-tartrate dehydratase subunit β	-1.273512162	4.445505529	0.042515362	0.101407523
b3992	thiF	ThiS adenylyltransferase	-1.274188412	8.369820481	0.000112015	0.000621289
b3993	thiE	thiamine phosphate synthase	-1.279660798	8.382155207	0.000117959	0.000646142
b4407	thiS	sulfur carrier protein ThiS	-1.286662233	8.347053674	0.0001514	0.000804368
b4257	yjgN	conserved inner membrane protein YjgN	-1.287814748	5.510660925	1.66E-05	0.000110989
b2269	elaD	protease ElaD	-1.290708773	3.937763528	0.032943108	0.083015879
b4026	yjbE	uncharacterized protein YjbE	-1.294494476	6.049819701	3.51E-05	0.000219819
b3603	lldP	lactateglycolate:H+	-1.294922338	6.607497776	9.57E-12	1.98E-10
b2518	ndk	nucleoside diphosphate kinase	-1.298519629	7.515293702	1.92E-16	7.91E-15
b2344	fadL	long-chain fatty acid outer membrane channel bacteriophage T2 recept	-1.299212901	6.431649822	2.96E-09	4.06E-08
b4416	rybA	small RNA RybA	-1.306177731	6.102150286	2.10E-06	1.72E-05
b3083	higB	ribosome-dependent mRNA interferase toxin HigB	-1.308603556	5.886739968	0.001177958	0.004702248
b1138	ymfE	e14 prophage; uncharacterized protein YmfE	-1.314012119	6.339749617	1.13E-08	1.44E-07
b4511	ybdZ	enterobactin biosynthesis protein YbdZ	-1.314129723	2.820006296	0.037559045	0.092277787
b1063	yceB	putative lipid-binding lipoprotein YceB	-1.314524105	7.354034617	1.33E-15	5.15E-14
b3081	fadH	DIENOYLCOAREDUCT-MONOMER	-1.323649381	5.110793416	0.003143683	0.011193034
b4002	zraP	zinc responsive, periplasmic protein with chaperone activity	-1.331026317	8.403114713	7.05E-05	0.000415494
b3117	tdcB	catabolic threonine dehydratase	-1.335786707	4.521970534	0.038129117	0.093263186
b1110	ycfJ	PF05433 family protein YcfJ	-1.339773714	7.189683772	2.10E-15	7.78E-14
b2119	yehL	putative AAA+ MoxR family ATPase YehL	-1.342335869	3.426961407	0.01905133	0.051984932
b0697	kdpB	K+ transporting P-type ATPase subunit KdpB	-1.34712853	5.604195707	2.38E-06	1.92E-05
b3043	ygiL	putative fimbrial protein YgiL	-1.347211709	4.856146293	4.24E-05	0.000262604
b4318	fimF	type 1 fimbriae minor subunit FimF	-1.347856773	5.417282656	0.000503911	0.002233701
b3059	plsY	putative glycerol-3-phosphate acyltransferase	-1.349837221	6.807888074	1.73E-11	3.39E-10
b4655	ythA	KpLE2 phage-like element; uncharacterized protein YthA	-1.356853062	2.876855146	0.075937021	0.160951487
b3557	insJ	insertion element IS150 protein InsA	-1.356985476	5.787523841	1.15E-06	9.94E-06
b0899	ycaM	putative transporter YcaM	-1.359592504	6.186162938	4.84E-08	5.39E-07
b4354	btsT	pyruvate:H+ symporter	-1.360896284	9.503932088	1.46E-11	2.90E-10
b2243	glpC	anaerobic glycerol-3-phosphate dehydrogenase subunit C	-1.361280764	5.049371751	0.000200769	0.001015346
b1304	pspA	phage shock protein A	-1.363441608	7.926652914	4.57E-20	2.93E-18
b4267	idnD	IDONDEHYD-MONOMER	-1.363992895	3.259723634	0.014096638	0.040413414
b1021	pgaD	poly-N-acetyl-D-glucosamine synthase subunit PgaD	-1.365956542	4.645754268	0.000119178	0.000652008
b4319	fimG	type 1 fimbriae minor subunit FimG	-1.366418535	5.492224966	0.00016532	0.000868252
b3086	ygjQ	DUF218 domain-containing protein YgjQ	-1.368917679	5.082125608	0.002934814	0.010542884
b1652	rnt	RNase T	-1.370704647	6.393784905	4.63E-11	8.58E-10
b2093	gatB	galactitol-specific PTS enzyme IIB component	-1.372281319	7.723010543	9.85E-17	4.14E-15
b0367	tauC	taurine ABC transporter membrane subunit	-1.373571035	5.732383475	3.98E-06	3.04E-05
b1384	feaR	DNA-binding transcriptional activator FeaR	-1.375129257	4.854444108	0.000109223	0.000607328
b3750	rbsC	ribose ABC transporter membrane subunit	-1.378859203	7.756496842	1.20E-19	7.05E-18
b0585	fes	ferric enterobactin esterase	-1.387738593	3.819331738	0.004758752	0.016000882
b0989	cspH	CspA family protein CspH	-1.390789882	4.69536391	0.000133093	0.000719226
b4684	yqfG	uncharacterized protein YqfG	-1.392856044	4.78462169	0.001067856	0.004325307
b3120	yhaB	protein YhaB	-1.394485446	4.904413658	0.009057969	0.02782946
b2870	ygeW	putative carbamoyltransferase YgeW	-1.394903067	4.794940186	0.000300755	0.001438604

b2123	yehR	DUF1307 domain-containing lipoprotein YehR	-1.396589734	5.513986832	3.82E-07	3.60E-06
b4293	fecI	RNA polymerase sigma factor FecI	-1.398684475	6.352884472	4.69E-11	8.63E-10
b1126	potA	spermidine preferential ABC transporter ATP binding subunit	-1.407179007	7.909914093	1.10E-22	9.33E-21
b3118	tdcA	DNA-binding transcriptional activator TdcA	-1.415791233	5.083307206	0.001965635	0.007442776
b0929	ompF	outer membrane porin F	-1.417850917	9.055948017	3.25E-17	1.46E-15
b3427	yzgL	putative uncharacterized protein YzgL	-1.4291519	6.505046069	1.93E-12	4.53E-11
b1295	ymjA	DUF2543 domain-containing protein YmjA	-1.432613585	5.913442646	8.30E-09	1.09E-07
b1029	ycdU	uncharacterized protein YcdU	-1.434280641	4.781181917	2.12E-05	0.000138973
b2986	yghT	putative ATP-binding protein YghT	-1.437829926	4.16481773	0.002921679	0.01051116
b1858	znuC	Zn2+ ABC transporter ATP binding subunit	-1.438258555	7.365470331	2.43E-20	1.65E-18
b3121	yhaC	uncharacterized protein YhaC	-1.450153957	5.633323809	5.83E-05	0.000351366
b1081	flgJ	putative peptidoglycan hydrolase FlgJ	-1.45226115	4.679511684	0.000174746	0.000906586
b3926	glpK	glycerol kinase	-1.453835315	9.360589738	2.31E-08	2.74E-07
b4455	hokA	small toxic polypeptide	-1.455021131	5.547663185	8.47E-06	6.08E-05
b0535	fimZ	putative LuxR family transcriptional regulator FimZ	-1.458944712	4.649284204	0.000110872	0.000615726
b1946	fliN	flagellar motor switch protein FliN	-1.46970302	4.661922574	0.000461839	0.00206797
b1452	yncE	PQQ-like domain-containing protein YncE	-1.482388078	7.003895332	4.58E-16	1.84E-14
b3615	waaH	EG11266-MONOMER	-1.487035815	6.819253573	6.21E-12	1.32E-10
b0247	ykfG	CP4-6 prophage; RadC-like JAB domain-containing protein YkfG	-1.493773181	4.98873399	2.38E-05	0.000153778
b0554	essD	DLP12 prophage; putative phage lysis protein	-1.496260873	4.088169545	0.000662561	0.00285944
b2047	wcaJ	undecaprenyl-phosphate glucose phosphotransferase	-1.496860472	4.33668522	0.001316745	0.005195202
b1959	yedA	putative transporter YedA	-1.49778849	6.603290668	2.68E-14	8.64E-13
b1163	bluF	blue light-responsive regulator of BluR	-1.497930039	6.603713724	2.81E-10	4.50E-09
b2115	yehF	putative protein YehF	-1.517408613	5.327289358	2.84E-07	2.74E-06
b3927	glpF	glycerol facilitator	-1.522190224	8.916732057	2.72E-07	2.65E-06
b4347	symE	toxic protein SymE	-1.522306434	3.175652262	0.005052534	0.016797391
b1415	aldA	aldehyde dehydrogenase A	-1.523816264	7.20879779	1.13E-20	8.06E-19
b1265	trpL	trp operon leader peptide	-1.530160037	3.17215684	0.039604604	0.096021046
b4593	ymgI	uncharacterized protein YmgI	-1.534367984	5.096167662	6.93E-06	5.06E-05
b4292	fecR	regulator for fec operon, periplasmic	-1.534891167	5.929344614	8.91E-09	1.17E-07
b2239	glpQ	glycerophosphoryl diester phosphodiesterase	-1.538843827	8.510355133	1.46E-10	2.45E-09
b4541	yehK	uncharacterized protein YehK	-1.550863312	2.001375843	0.193438705	0.331791718
b1551	ynfN	Qin prophage; protein YnfN	-1.552457171	3.927428456	0.004012528	0.013844632
b4325	yjiC	uncharacterized protein YjiC	-1.553549675	3.852298761	0.000555225	0.002436696
b4299	yjhI	KpLE2 phage-like element; putative DNA-binding transcriptional regulat	-1.554853955	3.529753258	0.017258891	0.047803014
b2583	yfiP	DTW domain-containing protein YfiP	-1.560242882	6.751415172	6.96E-17	3.04E-15
b1055	yceA	UPF0176 protein YceA	-1.562565545	8.354974342	1.49E-11	2.94E-10
b0434	yajG	putative lipoprotein YajG	-1.567260132	7.734906263	8.85E-26	1.03E-23
b1549	ydfO	Qin prophage; DUF1398 domain-containing protein YdfO	-1.581731952	3.837840226	0.000906664	0.003765684
b2698	recX	RecA inhibitor RecX	-1.588652704	5.878036891	4.74E-10	7.36E-09
b2832	ygdQ	UPF0053 inner membrane protein YgdQ	-1.589568802	7.606903148	3.19E-21	2.51E-19
b0366	tauB	taurine ABC transporter ATP binding subunit	-1.591743626	6.173962957	1.57E-08	1.91E-07
b1161	ycgX	DUF1398 domain-containing protein YcgX	-1.599463724	5.011874802	3.35E-06	2.62E-05
b0379	yaiY	inner membrane protein	-1.600716679	6.099041865	1.25E-11	2.51E-10
b1122	ymfA	putative inner membrane protein	-1.613394049	4.219082243	0.000262106	0.001280086
b3078	ygjI	putative transporter YgjI	-1.623921576	4.673521165	0.010477809	0.031576468
b1730	ydjO	protein YdjO	-1.624949779	7.314180416	6.32E-21	4.73E-19

b0681	chiP	chitobiose outer membrane channel	-1.641347035	5.099497532	1.65E-05	0.000110233
b3119	tdcR	DNA-binding transcriptional activator TdcR	-1.642859841	4.274948959	0.036279061	0.089732244
b4748	ynfT	Qin prophage; protein YnfT	-1.668542145	5.858361687	1.85E-08	2.23E-07
b0365	tauA	taurine ABC transporter periplasmic binding protein	-1.675057902	6.247978991	2.52E-11	4.84E-10
b0241	phoE	outer membrane porin PhoE	-1.681084915	5.387422292	4.01E-07	3.76E-06
b3708	tnaA	tryptophanase	-1.68299978	6.122580495	3.09E-07	2.97E-06
b1951	rcsA	DNA-binding transcriptional activator RcsA	-1.698095369	7.245548569	2.60E-20	1.74E-18
b4204	yjfZ	protein YjfZ	-1.699360625	5.349642386	1.51E-08	1.87E-07
b3749	rbsA	ribose ABC transporter ATP binding subunit	-1.7128592	8.342702255	2.94E-27	3.82E-25
b2028	ugd	UDP-glucose 6-dehydrogenase	-1.719496917	7.970393245	2.19E-20	1.51E-18
b1558	cspF	Qin prophage; cold shock protein CspF	-1.739748776	6.257405872	2.79E-12	6.28E-11
b2582	trxC	oxidized thioredoxin 2	-1.763882706	7.155955235	4.00E-26	4.90E-24
b2393	nupC	nucleoside:H+ symporter NupC	-1.76485873	8.046889917	1.57E-20	1.10E-18
b3687	ibpA	small heat shock protein IbpA	-1.764910137	7.298526264	1.00E-22	8.69E-21
b0722	sdhD	succinate:quinone oxidoreductase, membrane protein SdhD	-1.774870099	7.726380612	5.87E-20	3.70E-18
b3077	ebgC	DUF386 domain-containing evolved β-D-galactosidase subunit &b	-1.775373373	4.26980239	0.016888505	0.046865337
b1171	ymgD	PF16456 family protein YmgD	-1.781067561	6.4515777	9.00E-17	3.82E-15
b0254	perR	putative transcriptional regulator PerR	-1.78566348	6.170836802	8.48E-14	2.44E-12
b4504	ykfH	DUF987 domain-containing protein YkfH	-1.789113124	4.708676994	2.05E-05	0.000134657
b0411	tsx	nucleoside-specific channel-forming protein Tsx	-1.794368337	7.914728575	1.36E-29	2.07E-27
b1172	ymgG	PF13488 family protein YmgG	-1.794549603	6.900431777	2.33E-21	1.87E-19
b2081	yegQ	putative peptidase YegQ	-1.800881559	8.289086105	1.40E-31	2.57E-29
b1079	flgH	flagellar L-ring protein	-1.807936074	4.875693489	6.22E-07	5.66E-06
b0527	ybcI	conserved inner membrane protein YbcI	-1.812106315	5.519639907	5.44E-09	7.30E-08
b0544	ybcK	DLP12 prophage; putative recombinase	-1.812666001	6.177749479	1.18E-15	4.60E-14
b3686	ibpB	small heat shock protein IbpB	-1.866542133	6.951156036	2.65E-20	1.74E-18
b2056	wcaD	putative colanic acid polymerase	-1.873343356	4.853033439	2.89E-07	2.79E-06
b1375	ynaE	Rac prophage; uncharacterized protein YnaE	-1.875114013	5.969210933	3.12E-11	5.90E-10
b1938	fliF	flagellar basal-body MS-ring and collar protein	-1.892200452	5.356597549	1.10E-08	1.42E-07
b1077	flgF	flagellar basal-body rod protein FlgF	-1.895414859	5.335705382	9.69E-06	6.87E-05
b1080	flgI	flagellar P-ring protein	-1.904093923	4.87524715	5.42E-07	4.97E-06
b2060	wzc	protein-tyrosine kinase Wzc	-1.904996006	5.324088239	4.58E-08	5.12E-07
b0685	ybfE	LexA-regulated protein	-1.909503398	6.408627658	4.43E-16	1.80E-14
b2140	dusC	tRNA-dihydrouridine16 synthase	-1.940133615	5.721237928	3.19E-10	5.03E-09
b1877	yecT	protein YecT	-1.972435322	6.578118541	2.08E-19	1.19E-17
b1702	ppsA	phosphoenolpyruvate synthetase	-1.988917342	9.42523142	2.90E-29	4.26E-27
b1544	ydfK	Qin prophage; cold shock protein YdfK	-2.015501968	5.801115105	5.66E-13	1.46E-11
b2888	uacT	urate:H+ symporter	-2.018067534	4.758460831	5.82E-06	4.32E-05
b2211	yojI	ABC transporter family protein microcin J25 efflux protein	-2.032362328	7.441222544	2.86E-25	3.08E-23
b0647	ybeT	Sel1 repeat-containing protein YbeT	-2.033540852	2.559291676	0.008632315	0.026745032
b0593	entC	isochorismate synthase EntC	-2.044001757	3.791240152	4.60E-05	0.000283113
b1072	flgA	flagellar basal body P-ring formation protein FlgA	-2.049069311	5.305212415	1.18E-09	1.73E-08
b0357	frmR	DNA-binding transcriptional repressor FrmR	-2.073135966	7.187311461	6.51E-31	1.15E-28
b1038	csgF	curli assembly component	-2.075560889	1.956675274	0.084907793	0.176574614
b4739	ymgL	protein YmgL	-2.102458429	3.531436937	0.000901778	0.003752452
b1891	flhC	DNA-binding transcriptional dual regulator FlhC	-2.131559917	6.758788709	9.54E-27	1.20E-24
b1944	fliL	flagellar protein FliL	-2.145459525	5.537565054	2.41E-10	3.93E-09

b2796	sdaC	SDAC-MONOMER	-2.157609589	8.372856575	4.42E-33	9.29E-31
b1078	flgG	flagellar basal-body rod protein FlgG	-2.160376042	5.713173298	8.96E-11	1.57E-09
b0356	frmA	S-(hydroxymethyl)glutathione dehydrogenase	-2.179948309	8.907328494	9.38E-34	2.18E-31
b4366	bglJ	DNA-binding transcriptional regulator BglJ	-2.189600476	5.870872897	3.13E-13	8.38E-12
b1422	ydcI	putative DNA-binding transcriptional repressor YdcI	-2.206778296	7.378984014	9.01E-24	8.65E-22
b1018	efeO	ferrous iron transport system protein EfeO	-2.209748792	6.96314969	3.30E-18	1.66E-16
b3528	dctA	C4 dicarboxylateorotate:H+	-2.23393897	7.73089785	9.50E-24	8.92E-22
b0721	sdhC	succinate:quinone oxidoreductase, membrane protein SdhC	-2.238497697	7.650395669	2.59E-25	2.86E-23
b2050	wcaI	putative colanic biosynthesis glycosyl transferase	-2.242832018	4.910101093	4.10E-07	3.84E-06
b2051	gmm	GDP-mannose mannosyl hydrolase	-2.264972532	4.251459013	3.26E-06	2.56E-05
b4744	ynaM	Rac prophage; protein YnaM	-2.267499494	5.883775485	2.56E-14	8.44E-13
b2053	gmd	GDP-mannose 4,6-dehydratase	-2.267589322	5.885029081	8.88E-11	1.56E-09
b4513	kdpF	K+ transporting P-type ATPase subunit KdpF	-2.26772744	3.134837716	0.000220981	0.001094984
b1945	fliM	flagellar motor switch protein FliM	-2.298774003	6.145563723	1.75E-12	4.21E-11
b2797	sdaB	L-serine deaminase II	-2.3095025	8.133603531	1.04E-38	3.54E-36
b4438	cyaR	small regulatory RNA CyaR	-2.374277429	4.558582619	1.26E-08	1.59E-07
b4735	ybgU	protein YbgU	-2.377578601	5.880390144	5.09E-15	1.75E-13
b4240	treB	trehalose-specific PTS enzyme IIBC component	-2.389554823	6.91169281	2.24E-17	1.05E-15
b0355	frmB	S-formylglutathione hydrolase FrmB	-2.440152236	8.07565162	1.60E-49	8.80E-47
b2242	glpB	anaerobic glycerol-3-phosphate dehydrogenase subunit B	-2.498902662	5.517818499	6.05E-10	9.21E-09
b1922	fliA	RNA polymerase, sigma 28 (sigma F) factor	-2.527973787	5.70580689	2.68E-14	8.64E-13
b1076	flgE	flagellar hook protein FlgE	-2.529237807	6.58387531	5.82E-18	2.83E-16
b2091	gatD	galactitol-1-phosphate 5-dehydrogenase	-2.640663762	8.72787468	1.11E-30	1.88E-28
b2061	wzb	G7106-MONOMER	-2.73316118	3.529313427	4.41E-05	0.000272007
b0590	fepD	ferric enterobactin ABC transporter membrane subunit FebD	-2.740791688	4.67944547	2.23E-10	3.66E-09
b4239	treC	TRE6PHYDRO-MONOMER	-2.761331892	6.738377134	9.22E-20	5.65E-18
b1892	flhD	DNA-binding transcriptional dual regulator FlhD	-2.77732015	6.624618182	3.57E-33	7.88E-31
b0698	kdpA	K+ transporting P-type ATPase subunit KdpA	-2.803328401	5.375861563	1.45E-14	4.89E-13
b1075	flgD	flagellar biosynthesis, initiation of hook assembly	-2.829173245	5.824842738	4.01E-15	1.43E-13
b4367	fhuF	hydroxamate siderophore iron reductase	-2.858434244	7.657638548	3.10E-50	1.95E-47
b4586	ykfM	uncharacterized protein YkfM	-3.002428021	5.449229184	2.75E-15	1.01E-13
b2055	wcaE	putative colanic acid biosynthesis glycosyl transferase WcaE	-3.051579787	4.326415005	1.42E-06	1.20E-05
b2054	wcaF	putative colanic acid biosynthesis acetyltransferase WcaF	-3.058529583	3.561444941	1.45E-06	1.22E-05
b2052	fcl	GDP-L-fucose synthase	-3.07376606	5.494241581	2.15E-13	5.92E-12
b3426	glpD	aerobic glycerol 3-phosphate dehydrogenase	-3.092372722	9.124059191	3.55E-54	3.92E-51
b2148	mglC	D-galactosemethyl-galactoside ABC transporter membrane subunit	-3.226636417	6.331132619	9.84E-24	9.05E-22
b2252	ais	putative lipopolysaccharide core heptose(II)-phosphate phosphatase	-3.295780926	7.038943417	1.73E-45	8.51E-43
b1074	flgC	flagellar basal-body rod protein FlgC	-3.414651199	5.18101959	3.49E-14	1.08E-12
b2241	glpA	anaerobic glycerol-3-phosphate dehydrogenase subunit A	-3.417225081	6.116713418	9.55E-14	2.72E-12
b0364	yaiS	putative deacetylase YaiS	-3.501809453	3.6760976	1.31E-05	8.97E-05
b2150	mglB	D-galactosemethyl-galactoside ABC transporter periplasmic binding prc	-3.812082966	7.076059445	4.31E-42	1.90E-39
b2062	wza	outer membrane polysaccharide export protein Wza	-3.869993731	4.838319054	7.90E-13	1.98E-11
b2149	mglA	D-galactosemethyl-galactoside ABC transporter ATP binding subunit	-4.042208733	6.757447734	4.63E-36	1.20E-33
b1073	flgB	flagellar basal-body rod protein FlgB	-4.072807206	5.366911678	2.63E-19	1.49E-17
b2240	glpT	GLPT-MONOMER	-4.317000402	8.291366926	4.13E-34	1.01E-31
