# Supplementary material for: High-throughput transcriptome sequencing and comparative analysis of Escherichia coli and Schizosaccharomyces pombe in respiratory and fermentative growth
Source: PLoS One. 2021 Mar 17;16(3):e0248513. doi: 10.1371/journal.pone.0248513 (PMC7968713; doi:10.1371/journal.pone.0248513)
Supplement: S2 File — (PDF) [file pone.0248513.s007.pdf]

Systematic ID	Gene	Description	logFC	logCPM	PValue	FDR
SPBTRNATHR.07	SPBTRNATHR.07	tRNA Threonine	7.30213689	2.98254886	2.60E-14	2.91E-13
SPCTRNATHR.08	SPCTRNATHR.08	tRNA Threonine	7.05184184	2.77382839	1.79E-12	1.74E-11
SPATRNATRP.01	SPATRNATRP.01	tRNA Tryptophan	6.96016403	2.70399118	1.83E-12	1.78E-11
SPBTRNATRP.03	SPBTRNATRP.03	tRNA Tryptophan	6.96016403	2.70399118	1.83E-12	1.78E-11
SPATRNAILE.03	SPATRNAILE.03	tRNA Isoleucine	6.95817202	2.70380494	3.52E-12	3.28E-11
SPATRNATHR.01	SPATRNATHR.01	tRNA Threonine	6.67787747	2.47501117	9.88E-10	6.90E-09
SPATRNATHR.03	SPATRNATHR.03	tRNA Threonine	6.30808157	2.20086998	5.47E-08	3.01E-07
SPCTRNASER.09	SPCTRNASER.09	tRNA Serine	5.81975273	1.86249656	3.19E-06	1.38E-05
SPCTRNASER.13	SPCTRNASER.13	tRNA Serine	5.74541083	1.81912221	1.45E-05	5.56E-05
SPCTRNASER.07	SPCTRNASER.07	tRNA Serine	5.61432361	1.72959961	1.74E-05	6.60E-05
SPCTRNASER.08	SPCTRNASER.08	tRNA Serine	5.61432361	1.72959961	1.74E-05	6.60E-05
SPATRNAPRO.01	SPATRNAPRO.01	tRNA Proline	5.45583127	1.63338275	6.24E-05	0.000212934
SPBTRNASER.06	SPBTRNASER.06	tRNA Serine	5.45250991	1.63320147	8.06E-05	0.000269441
SPATRNASER.04	SPATRNASER.04	tRNA Serine	5.36924958	1.58274486	0.00012393	0.000402739
SPBTRNAPRO.08	SPBTRNAPRO.08	tRNA Proline	5.28434898	1.53078682	0.00022719	0.000704673
SPRRNA.32	SPRRNA.32	5S rRNA	5.28337577	1.53070785	0.00023208	0.000718841
SPATRNAILE.04	SPATRNAILE.04	tRNA Isoleucine	5.08486191	1.41993685	0.00044565	0.001305476
SPBTRNAARG.07	SPBTRNAARG.07	tRNA Arginine	5.07539911	1.41935756	0.00089813	0.002439833
SPATRNAARG.01	SPATRNAARG.01	tRNA Arginine	4.84850619	1.299574	0.0014035	0.003635647
SPATRNASER.02	SPATRNASER.02	tRNA Serine	4.84504892	1.29932341	0.00268904	0.006528333
SPAPJ760.03c	adg1	Schizosaccharomyces specific protein Adg1	4.52458565	6.28501313	5.17E-112	2.07E-109
SPATRNATHR.02	SPATRNATHR.02	tRNA Threonine	4.49673202	2.96301526	4.24E-12	3.92E-11
SPATRNATHR.04	SPATRNATHR.04	tRNA Threonine	4.40678554	1.09805371	0.00955197	0.020211806
SPBTRNATRP.02	SPBTRNATRP.02	tRNA Tryptophan	4.36585837	2.8604366	5.99E-11	4.79E-10
SPBTRNAMET.06	SPBTRNAMET.06	tRNA Methionine	4.35063804	2.83979542	3.19E-10	2.36E-09
SPCTRNATHR.09	SPCTRNATHR.09	tRNA Threonine	4.20349169	2.72825058	1.40E-09	9.57E-09
SPBTRNAASN.03	SPBTRNAASN.03	tRNA Asparagine	4.1935804	3.48568797	2.02E-15	2.52E-14
SPATRNAMET.03	SPATRNAMET.03	tRNA Methionine	4.0670198	3.37174036	1.56E-13	1.65E-12
SPCTRNASER.12	SPCTRNASER.12	tRNA Serine	4.01425098	0.94617604	0.03500389	0.063707261
SPATRNAALA.03	scn1	tRNA Alanine	4.01089426	1.98441841	1.26E-05	4.90E-05
SPATRNATHR.05	SPATRNATHR.05	tRNA Threonine	3.96274879	2.96325849	1.45E-10	1.11E-09
SPATRNAILE.01	SPATRNAILE.01	tRNA Isoleucine	3.94494236	1.94506089	1.93E-05	7.23E-05
SPAC821.09	eng1	cell septum surface endo-1,3-beta-glucanase Eng1	3.89530086	8.7113161	7.65E-168	1.04E-164
SPATRNAGLY.01	SPATRNAGLY.01	tRNA Glycine	3.85825593	3.19903723	3.91E-13	4.02E-12
SPATRNASER.03	sup3	tRNA Serine	3.80704815	3.16434916	4.08E-11	3.37E-10
SPBTRNAASN.02	SPBTRNAASN.02	tRNA Asparagine	3.75433108	3.95897437	6.92E-19	1.17E-17
SPCTRNAMET.07	SPCTRNAMET.07	tRNA Methionine	3.6897811	3.53944971	5.08E-14	5.57E-13
SPBTRNASER.05	SPBTRNASER.05	tRNA Serine	3.67945637	1.7754652	0.00015844	0.000505232
SPCTRNAASN.06	SPCTRNAASN.06	tRNA Asparagine	3.60025581	3.82558172	5.69E-18	8.94E-17
SPATRNAVAL.02	SPATRNAVAL.02	tRNA Valine	3.53731629	2.94316086	1.27E-09	8.78E-09
SPCTRNAVAL.11	SPCTRNAVAL.11	tRNA Valine	3.51127976	2.92303542	1.50E-09	1.02E-08
SPCTRNALYS.11	SPCTRNALYS.11	tRNA Lysine	3.48549565	2.20135254	4.26E-06	1.80E-05
SPRRNA.36	SPRRNA.36	5S rRNA	3.43611949	1.63394842	0.00113608	0.003008188
SPCTRNASER.11	sup9	tRNA Serine	3.40567037	3.31065528	3.79E-10	2.77E-09
SPCTRNAGLY.10	SPCTRNAGLY.10	tRNA Glycine	3.37227696	3.6292627	1.16E-14	1.34E-13
SPBTRNAARG.05	SPBTRNAARG.05	tRNA Arginine	3.36973287	2.81813354	3.51E-08	2.01E-07

SPATRNALEU.02	SPATRNALEU.02	tRNA Leucine	3.34194609	1.5825827	0.00200955	0.005038688
SPATRNAALA.02	scn2	tRNA Alanine	3.33075531	2.09692618	2.65E-05	9.74E-05
SPCTRNAASN.05	SPCTRNAASN.05	tRNA Asparagine	3.28911938	3.97013639	1.81E-17	2.69E-16
SPBTRNAGLY.03	SPBTRNAGLY.03	tRNA Glycine	3.26962883	3.82578426	1.58E-16	2.16E-15
SPBTRNAILE.06	SPBTRNAILE.06	tRNA Isoleucine	3.21645401	2.70435042	3.96E-07	1.93E-06
SPATRNAPRO.02	SPATRNAPRO.02	tRNA Proline	3.21274245	2.02266588	8.25E-05	0.000275111
SPBTRNAILE.05	SPBTRNAILE.05	tRNA Isoleucine	3.2058874	2.94297672	3.53E-08	2.01E-07
SPBTRNAILE.08	SPBTRNAILE.08	tRNA Isoleucine	3.2058874	2.94297672	3.53E-08	2.01E-07
SPNCRNA.1415	#N/A	#N/A	3.14534871	1.4760402	0.00492191	0.0112065
SPBTRNAILE.07	SPBTRNAILE.07	tRNA Isoleucine	3.07231509	2.83971851	6.67E-08	3.63E-07
SPCTRNASER.10	SPCTRNASER.10	tRNA Serine	3.02275367	1.90463094	0.00027666	0.0008431
SPBTRNAGLY.07	SPBTRNAGLY.07	tRNA Glycine	3.01465693	3.84749835	2.63E-15	3.23E-14
SPBTRNAGLY.08	SPBTRNAGLY.08	tRNA Glycine	3.01465693	3.84749835	2.63E-15	3.23E-14
SPNCRNA.1307	#N/A	#N/A	2.95737969	5.10641622	2.60E-23	6.20E-22
SPBTRNALYS.07	SPBTRNALYS.07	tRNA Lysine	2.92192974	2.2018837	0.00016818	0.000533056
SPBTRNALYS.08	SPBTRNALYS.08	tRNA Lysine	2.92192974	2.2018837	0.00016818	0.000533056
SPBTRNAGLY.06	SPBTRNAGLY.06	tRNA Glycine	2.90058298	3.24837399	2.92E-09	1.91E-08
SPBTRNALYS.06	SPBTRNALYS.06	tRNA Lysine	2.86848241	2.44652796	1.79E-05	6.77E-05
SPAC5H10.03	SPAC5H10.03	phosphoglycerate mutase/6-phosphofructo-2-kinase family	2.8558236	7.1561947	2.13E-115	1.03E-112
SPNCRNA.452	#N/A	#N/A	2.77728638	1.77477048	0.00887338	0.018911167
SPNCRNA.941	#N/A	#N/A	2.77205913	3.64217874	6.60E-09	4.13E-08
SPBPB10D8.02c	SPBPB10D8.02c	arylsulfatase (predicted)	2.73205452	5.18883517	7.00E-21	1.47E-19
SPATRNAARG.02	SPATRNAARG.02	tRNA Arginine	2.72554163	1.72991398	0.00228812	0.005676515
SPNCRNA.236	#N/A	#N/A	2.72539128	3.60486276	1.86E-08	1.09E-07
SPNCRNA.385	#N/A	#N/A	2.64310537	1.68245606	0.00382054	0.008911136
SPATRNAMET.01	SPATRNAMET.01	tRNA Methionine	2.61474735	3.96990283	8.30E-12	7.42E-11
SPNCRNA.489	#N/A	#N/A	2.53446695	1.633754	0.00820438	0.017606692
SPNCRNA.1190	#N/A	#N/A	2.5336471	2.65822113	4.25E-05	0.000149359
ScpofMt22	#N/A	#N/A	2.52909881	4.30977699	2.82E-14	3.16E-13
SPCC74.04	SPCC74.04	amino acid transmembrane transporter (predicted)	2.52817846	7.37006481	1.76E-30	6.53E-29
SPCTRNAGLN.06	SPCTRNAGLN.06	tRNA Glutamine	2.50810062	4.28557988	3.28E-16	4.39E-15
SPCTRNAARG.08	SPCTRNAARG.08	tRNA Arginine	2.50759377	3.64301096	1.58E-10	1.21E-09
SPAC26H5.09c	SPAC26H5.09c	oxidoreductase involved in NADPH regeneration (predicted)	2.50193593	8.49755573	4.01E-172	6.82E-169
SPRRNA.12	SPRRNA.12	5S rRNA	2.4923572	4.1543153	7.48E-15	8.85E-14
SPCTRNAARG.09	SPCTRNAARG.09	tRNA Arginine	2.4739155	1.16863057	0.04602063	0.080589395
SPAPB1E7.04c	SPAPB1E7.04c	chitinase (predicted)	2.44077933	8.52647827	4.30E-114	1.83E-111
SPCC18.01c	adg3	beta-glucosidase Adg3 (predicted)	2.42988641	8.49375266	2.60E-58	3.93E-56
SPCTRNAGLN.05	SPCTRNAGLN.05	tRNA Glutamine	2.41651096	4.21287927	1.17E-14	1.35E-13
SPBTRNAVAL.05	SPBTRNAVAL.05	tRNA Valine	2.37415287	2.1336531	0.00194502	0.004896735
SPBTRNAVAL.08	SPBTRNAVAL.08	tRNA Valine	2.37290055	2.1337201	0.00247737	0.006079521
SPATRNAPRO.03	SPATRNAPRO.03	tRNA Proline	2.36689445	2.70518789	1.90E-05	7.14E-05
SPNCRNA.482	#N/A	#N/A	2.34711249	1.53075175	0.02346546	0.045082566
SPNCRNA.485	#N/A	#N/A	2.34711249	1.53075175	0.02346546	0.045082566
SPAPJ695.02	SPAPJ695.02	Schizosaccharomyces pombe specific protein	2.3459032	6.22671448	3.32E-28	1.05E-26
SPATRNALYS.03	SPATRNALYS.03	tRNA Lysine	2.33098285	2.68124005	8.21E-05	0.000273842
SPCTRNAILE.09	SPCTRNAILE.09	tRNA Isoleucine	2.32665277	2.09690279	0.00076	0.002108157
SPRRNA.33	SPRRNA.33	5S rRNA	2.30346332	1.81983277	0.00594325	0.013286398

SPBPB10D8.01	SPBPB10D8.01	cysteine transmembrane transporter (predicted)	2.29077014	4.92185253	1.84E-20	3.69E-19
SPAC19D5.07	uga1	4-aminobutyrate aminotransferase (GABA transaminase)	2.28647426	8.98402213	4.20E-150	3.57E-147
SPBTRNALEU.06	SPBTRNALEU.06	tRNA Leucine	2.25784046	4.13555807	5.14E-11	4.15E-10
SPAC869.01	SPAC869.01	hydrolase activity, implicated in cellular detoxification (prec	2.25773139	6.80189575	4.82E-68	9.94E-66
SPBTRNAVAL.07	SPBTRNAVAL.07	tRNA Valine	2.25589057	2.06148133	0.00360085	0.008464167
SPBTRNALEU.07	SPBTRNALEU.07	tRNA Leucine	2.24689908	4.12689331	6.70E-11	5.31E-10
SPNCRNA.1666	#N/A	#N/A	2.23624795	3.34335141	2.60E-07	1.30E-06
SPCTRNALYS.10	SPCTRNALYS.10	tRNA Lysine	2.21874423	1.77586156	0.0072329	0.015760454
SPBTRNAASN.04	SPBTRNAASN.04	tRNA Asparagine	2.19605356	4.60161117	7.30E-16	9.46E-15
SPBTRNATHR.06	SPBTRNATHR.06	tRNA Threonine	2.19548071	3.31288689	1.98E-07	1.00E-06
SPCC757.06	SPCC757.06	Schizosaccharomyces pombe specific protein, similar to a r	2.1920896	2.02294011	0.00754829	0.016374288
SPCTRNALEU.13	SPCTRNALEU.13	tRNA Leucine	2.16665168	4.22023653	1.16E-10	8.99E-10
SPNCRNA.1672	#N/A	#N/A	2.16303625	4.12808496	7.13E-11	5.63E-10
SPCTRNALEU.12	SPCTRNALEU.12	tRNA Leucine	2.15630571	4.21207338	1.51E-10	1.16E-09
SPCTRNATHR.10	SPCTRNATHR.10	tRNA Threonine	2.1445789	3.94118877	2.41E-10	1.81E-09
SPATRNALYS.05	SPATRNALYS.05	tRNA Lysine	2.14228147	2.20212783	0.00239305	0.005900262
SPATRNAARG.03	SPATRNAARG.03	tRNA Arginine	2.13928578	1.72998456	0.01265165	0.026018283
SPBC1861.02	abp2	unknown protein, may bind replication origins Abp2	2.13463707	6.84966478	6.66E-66	1.22E-63
SPNCRNA.163	#N/A	#N/A	2.13358526	1.73017816	0.01742508	0.03469212
SPATRNASER.01	SPATRNASER.01	tRNA Serine	2.13049263	4.33851714	6.86E-11	5.43E-10
SPCC191.05c	SPCC191.05c	nucleoside 2-deoxyribosyltransferase (predicted)	2.12380216	5.64780032	6.06E-17	8.63E-16
SPAC2E1P3.05c	SPAC2E1P3.05c	fungal cellulose binding domain protein	2.11946234	7.21236989	1.46E-73	3.31E-71
SPBC36.01c	SPBC36.01c	spermidine family transmembrane transporter (predicted)	2.11926041	6.94753489	1.75E-55	2.39E-53
SPBTRNAASN.01	SPBTRNAASN.01	tRNA Asparagine	2.11379527	4.13634986	1.30E-10	1.00E-09
SPATRNALYS.02	SPATRNALYS.02	tRNA Lysine	2.11189239	2.68135494	0.0002228	0.000693248
SPRRNA.24	SPRRNA.24	5S rRNA	2.08493751	2.16762658	0.00128393	0.003364327
SPNCRNA.421	#N/A	#N/A	2.06460354	3.61847941	8.39E-08	4.49E-07
SPATRNATYR.01	SPATRNATYR.01	tRNA Tyrosine	2.04658951	2.32971356	0.00158103	0.004052325
SPATRNALYS.01	SPATRNALYS.01	tRNA Lysine	2.03767603	1.68260221	0.01844237	0.036570433
SPNCRNA.1609	#N/A	#N/A	1.99093375	3.63079376	2.42E-07	1.21E-06
SPAC19G12.05	mce1	mitochondrial carrier, citrate (predicted)	1.9857223	5.52449849	1.90E-15	2.38E-14
SPBTRNAALA.08	SPBTRNAALA.08	tRNA Alanine	1.97777577	1.36085874	0.08438818	0.135598948
SPBTRNAALA.10	SPBTRNAALA.10	tRNA Alanine	1.97777577	1.36085874	0.08438818	0.135598948
SPRRNA.15	SPRRNA.15	5S rRNA	1.97465569	4.22186002	4.92E-11	3.98E-10
SPNCRNA.694	#N/A	#N/A	1.97306234	2.44899455	0.00493986	0.011231454
SPAC6G10.12c	ace2	transcription factor Ace2	1.96595386	6.64027552	2.10E-40	1.42E-38
SPBPB2B2.05	SPBPB2B2.05	class I glutamine amidotransferase family protein	1.95507804	3.91241305	2.70E-08	1.56E-07
SPAC22A12.04c	rps2201	40S ribosomal protein S15a (predicted)	1.95408934	6.86628746	4.91E-54	6.08E-52
SPAC19G12.16c	adg2	conserved fungal cell surface protein Adg2 (predicted)	1.92383236	8.55341927	4.48E-52	5.17E-50
SPBTRNAHIS.02	SPBTRNAHIS.02	tRNA Histidine	1.92115689	1.86297231	0.01116042	0.023253714
SPNCRNA.113	#N/A	#N/A	1.9112858	4.12886886	6.03E-10	4.31E-09
SPBPB2B2.01	SPBPB2B2.01	amino acid transmembrane transporter (predicted)	1.9064528	8.39727059	5.32E-92	1.72E-89
SPNCRNA.1674	#N/A	#N/A	1.8991248	4.51807225	5.27E-12	4.80E-11
SPAC8E11.10	SPAC8E11.10	sorbose reductase (predicted)	1.89263433	6.39663749	3.12E-28	9.91E-27
SPRRNA.27	SPRRNA.27	5S rRNA	1.87789551	3.09646132	0.0001238	0.000402522
SPAC23H4.06	gln1	glutamate-ammonia ligase Gln1	1.86159094	7.92982414	5.95E-75	1.40E-72
ScpofMt15	#N/A	#N/A	1.84929904	3.24873812	0.00013141	0.000425209

SPAC27D7.09c	SPAC27D7.09c	But2 family protein, similar to cell surface molecules	1.84837211	6.87606285	1.73E-44	1.49E-42
SPCC31H12.02c	mug73	multispanning 7TM plasma membrane rhodopsin family pr	1.84830034	3.65546977	1.16E-06	5.30E-06
SPNCRNA.231	#N/A	#N/A	1.846634	3.66742878	7.68E-06	3.11E-05
SPCTRNAPHE.04	SPCTRNAPHE.04	tRNA Phenylalanine	1.84085969	1.58317303	0.04416028	0.077747957
SPNCRNA.1375	#N/A	#N/A	1.83068878	1.29944668	0.12922588	0.195114734
SPCTRNAVAL.09	SPCTRNAVAL.09	tRNA Valine	1.82886387	1.82108159	0.04402998	0.077622802
SPNCRNA.51	#N/A	#N/A	1.79312354	2.47535905	0.00351961	0.008307689
SPAC869.04	SPAC869.04	formamidase-like protein, implicated in cellular detoxificati	1.78216793	4.24789093	7.87E-09	4.88E-08
SPRRNA.14	SPRRNA.14	5S rRNA	1.74142905	2.7966946	0.00056912	0.001621807
SPBC24C6.04	put2	delta-1-pyrroline-5-carboxylate dehydrogenase Put2 (predic	1.73903542	8.543841	4.44E-83	1.26E-80
SPBTRNAGLN.04	SPBTRNAGLN.04	tRNA Glutamine	1.73166206	4.35609295	4.95E-10	3.58E-09
SPBC1734.11	mas5	DNAJ domain protein Mas5 (predicted)	1.72438767	7.88094077	5.01E-53	5.98E-51
SPCC11E10.01	SPCC11E10.01	cystathionine beta-lyase (predicted)	1.72028413	8.10084356	2.45E-80	6.42E-78
SPBTRNAGLN.01	SPBTRNAGLN.01	tRNA Glutamine	1.7082444	4.370776	9.25E-10	6.48E-09
SPBTRNALEU.10	SPBTRNALEU.10	tRNA Leucine	1.69378973	2.6575265	0.00146435	0.003775785
SPBP8B7.26	SPBP8B7.26	Schizosaccharomyces specific protein	1.69058815	6.29587135	2.94E-24	7.29E-23
SPBTRNALEU.05	SPBTRNALEU.05	tRNA Leucine	1.68947408	1.94520751	0.02145409	0.04165337
SPATRNAVAL.01	SPATRNAVAL.01	tRNA Valine	1.68366944	3.28189457	5.49E-05	0.000189319
SPAC821.08c	slp1	substrate-specific mitotic metaphase/anaphase APC coacti	1.68099315	6.89283603	1.87E-42	1.50E-40
SPATRNALYS.04	SPATRNALYS.04	tRNA Lysine	1.67982583	2.26712689	0.00910418	0.019348506
SPBTRNALEU.08	SPBTRNALEU.08	tRNA Leucine	1.6782949	1.2354933	0.1237315	0.188185168
SPAC56F8.12	SPAC56F8.12	DUF2434 family conserved fungal multispanning membran	1.67820689	4.7182597	2.41E-11	2.05E-10
SPAC31G5.03	rps1101	40S ribosomal protein S11 (predicted)	1.67803239	6.52261936	1.13E-26	3.26E-25
SPAC23C11.05	ipp1	inorganic pyrophosphatase Ipp1 (predicted)	1.67787857	8.18427382	1.65E-76	4.01E-74
SPATRNAVAL.03	SPATRNAVAL.03	tRNA Valine	1.67595443	1.23558162	0.12205669	0.185982031
SPBTRNAGLN.02	SPBTRNAGLN.02	tRNA Glutamine	1.67432285	4.34876718	1.41E-09	9.60E-09
SPCTRNALYS.12	SPCTRNALYS.12	tRNA Lysine	1.65562744	2.09784029	0.02101027	0.040873321
SPRRNA.39	SPRRNA.39	5S rRNA	1.65473451	2.09772787	0.01961153	0.038526692
SPBC530.02	SPBC530.02	transmembrane transporter (predicted)	1.64874148	5.07001892	2.61E-10	1.96E-09
SPAC750.01	SPAC750.01	NADP-dependent aldo/keto reductase, unknown biological r	1.64365061	5.76685808	5.22E-17	7.48E-16
SPAC56E4.03	SPAC56E4.03	aromatic aminotransferase (predicted)	1.63079883	7.93273337	4.43E-31	1.69E-29
SPAC1B3.03c	wis2	cyclophilin family peptidyl-prolyl cis-trans isomerase Wis2	1.62289204	7.96177826	1.91E-42	1.51E-40
SPBC3E7.02c	hsp16	heat shock protein Hsp16	1.61798652	7.61580616	5.30E-38	3.00E-36
SPBTRNAARG.04	SPBTRNAARG.04	tRNA Arginine	1.61370617	3.07835755	0.00019687	0.000617663
SPBC839.13c	rpl1601	60S ribosomal protein L13/L16 (predicted)	1.6113427	7.2532594	7.04E-46	6.39E-44
SPBC409.08	SPBC409.08	spermine family transmembrane transporter (predicted)	1.610446	7.91898683	2.22E-52	2.61E-50
SPAC14C4.01c	SPAC14C4.01c	DUF1770 family protein	1.60867957	9.30030649	2.81E-94	1.01E-91
SPNCRNA.1036	#N/A	#N/A	1.60633838	7.97338142	1.58E-40	1.08E-38
SPNCRNA.1388	#N/A	#N/A	1.60530373	3.55531641	2.99E-05	0.000108289
SPBP22H7.08	rps1002	40S ribosomal protein S10 (predicted)	1.60250789	6.92525947	5.39E-42	4.03E-40
SPBTRNAGLY.05	SPBTRNAGLY.05	tRNA Glycine	1.58105046	2.79790608	0.00145857	0.00376397
SPBC660.14	mik1	mitotic inhibitor kinase Mik1	1.57311543	5.39965034	3.11E-16	4.17E-15
SPBP4H10.13	rps2302	40S ribosomal protein S23 (predicted)	1.56040813	6.98832069	2.90E-41	2.05E-39
SPATRNAVAL.04	SPATRNAVAL.04	tRNA Valine	1.55377853	1.68297886	0.12982573	0.195933488
SPCC24B10.09	rps1702	40S ribosomal protein S17 (predicted)	1.54777236	7.04606713	8.67E-43	7.11E-41
ScpofMt21	#N/A	#N/A	1.54626244	4.88286811	2.64E-11	2.23E-10
SPAC23A1.11	rpl1602	60S ribosomal protein L13/L16 (predicted)	1.54054149	7.60977955	6.02E-55	8.03E-53

SPNCRNA.383	#N/A	#N/A	1.53793337	2.65853358	0.00920174	0.01953146
SPBTRNATYR.02	SPBTRNATYR.02	tRNA Tyrosine	1.53691937	2.44738253	0.01112197	0.023180704
SPCP20C8.02c	SPCP20C8.02c	S. pombe specific UPF0321 family protein 1	1.53651748	3.84809577	2.36E-05	8.74E-05
SPBC29A3.12	rps902	40S ribosomal protein S9 (predicted)	1.53160696	7.35137211	1.43E-40	9.83E-39
SPATRNAILE.02	SPATRNAILE.02	tRNA Isoleucine	1.52772452	3.02304691	0.00070414	0.001971867
SPBTRNAGLN.03	SPBTRNAGLN.03	tRNA Glutamine	1.5241242	4.27120881	3.97E-08	2.25E-07
SPNCRNA.539	#N/A	#N/A	1.52202612	3.43274297	0.00023373	0.000723614
SPNCRNA.1673	#N/A	#N/A	1.51239457	5.05020935	1.56E-11	1.35E-10
ScpofMt23	#N/A	#N/A	1.51083338	3.65614327	8.03E-05	0.000268595
SPBC21B10.08c	SPBC21B10.08c	antibiotic biosynthesis monooxygenase-like domain (predic	1.51031029	6.96681209	1.38E-35	6.80E-34
SPAC25G10.05c	his1	ATP phosphoribosyltransferase	1.50544027	7.34418697	2.92E-39	1.84E-37
SPNCRNA.06	#N/A	#N/A	1.50335192	2.92529245	0.00150608	0.003877736
SPAC4G9.03	adk1	adenylate kinase Adk1	1.49580735	7.29373427	8.77E-46	7.86E-44
SPCTRNAPHE.05	SPCTRNAPHE.05	tRNA Phenylalanine	1.49462185	1.16832164	0.20273754	0.283774982
SPATRNALEU.04	SPATRNALEU.04	tRNA Leucine	1.49277897	1.1682533	0.19968721	0.280295268
SPCTRNALEU.11	SPCTRNALEU.11	tRNA Leucine	1.491439	1.16818496	0.19947276	0.280109812
SPNCRNA.264	#N/A	#N/A	1.491439	1.16818496	0.19947276	0.280109812
SPBC1271.09	tgp1	plasma membrane glycerophosphodiester transmembrane	1.49013271	6.13177703	1.09E-16	1.51E-15
SPBTRNAALA.09	SPBTRNAALA.09	tRNA Alanine	1.48915252	1.16852069	0.20298359	0.283926691
SPBTRNAALA.11	SPBTRNAALA.11	tRNA Alanine	1.48915252	1.16852069	0.20298359	0.283926691
SPRRNA.10	SPRRNA.10	5S rRNA	1.48606437	1.16901075	0.19770939	0.27809268
SPCC1827.03c	SPCC1827.03c	acetyl-CoA ligase (predicted)	1.48377449	7.88444369	1.93E-57	2.79E-55
SPNCRNA.897	#N/A	#N/A	1.47413855	6.97952029	1.28E-29	4.50E-28
SPCTRNAGLY.12	SPCTRNAGLY.12	tRNA Glycine	1.47100498	2.72972277	0.00389388	0.009062193
SPAC1071.07c	rps1502	40S ribosomal protein S15 (predicted)	1.46564215	7.49992511	4.39E-45	3.83E-43
SPNCRNA.122	#N/A	#N/A	1.46278411	1.63407034	0.09125165	0.145323542
SPBTRNAHIS.01	SPBTRNAHIS.01	tRNA Histidine	1.46248659	1.63365747	0.09638616	0.152005062
SPCTRNAHIS.04	SPCTRNAHIS.04	tRNA Histidine	1.46248659	1.63365747	0.09638616	0.152005062
SPAC513.07	SPAC513.07	flavonol reductase/cinnamoyl-CoA reductase family	1.46190239	7.18508581	7.95E-26	2.14E-24
SPAC1F8.07c	pdc101	pyruvate decarboxylase (predicted)	1.45896226	9.91088554	1.99E-92	6.79E-90
SPBTRNAMET.05	SPBTRNAMET.05	tRNA Methionine	1.45851744	3.26570297	0.00024355	0.000752325
SPAC23H4.05c	SPAC23H4.05c	Schizosaccharomyces pombe specific protein	1.45763978	1.98534455	0.03650469	0.066050091
SPNCRNA.1260	#N/A	#N/A	1.45715621	2.13382219	0.02965261	0.055162925
SPNCRNA.215	#N/A	#N/A	1.45415355	1.98515369	0.03391372	0.062105175
SPNCRNA.1134	#N/A	#N/A	1.45250695	4.46376186	8.00E-08	4.30E-07
SPAC1687.06c	rpl44	60S ribosomal protein L28/L44 (predicted)	1.42929874	7.1412101	4.82E-37	2.56E-35
SPBP4G3.02	pho1	acid phosphatase Pho1	1.42253581	8.97342804	5.24E-46	4.82E-44
SPBP26C9.02c	car1	arginase Car1	1.4205018	7.20239787	1.19E-26	3.40E-25
SPNCRNA.609	#N/A	#N/A	1.41106274	7.94472912	3.70E-48	3.65E-46
SPNCRNA.1166	#N/A	#N/A	1.40736713	2.94543453	0.00253867	0.006212016
SPAC11D3.14c	SPAC11D3.14c	5-oxoprolinase (ATP-hydrolizing) (predicted)	1.40461319	7.94725094	1.74E-48	1.77E-46
SPBC354.15	fap1	L-pipecolate oxidase	1.40229189	6.34473697	2.89E-20	5.72E-19
SPBC1773.03c	SPBC1773.03c	aminotransferase class-III, possible transaminase, unknowr	1.39789059	8.0360416	2.90E-54	3.73E-52
SPATRNAASP.02	SPATRNAASP.02	tRNA Aspartic acid	1.39582263	4.21513695	1.20E-06	5.50E-06
SPBTRNATYR.03	SPBTRNATYR.03	tRNA Tyrosine	1.39045447	2.47545803	0.01937021	0.038151735
SPNCRNA.716	#N/A	#N/A	1.38949673	4.88391658	1.22E-08	7.31E-08
SPBC776.11	rpl2801	60S ribosomal protein L27/L28	1.38880225	7.17918175	5.19E-32	2.12E-30

SPAPJ760.02c	abp1	cofilin/tropomyosin family, drebrin ortholog Abp1	1.38787263	7.38295647	6.62E-36	3.31E-34
SPATRNALEU.03	SPATRNALEU.03	tRNA Leucine	1.38118102	2.097782	0.03694349	0.066702163
SPBTRNAMET.04	SPBTRNAMET.04	tRNA Methionine	1.37649835	2.09783147	0.05109806	0.088261248
SPCTRNAASP.07	SPCTRNAASP.07	tRNA Asparagine	1.3724373	4.23177238	1.28E-06	5.83E-06
SPNCRNA.1181	#N/A	#N/A	1.36815825	6.5481767	5.45E-26	1.50E-24
SPCC1795.05c	ura6	uridylate kinase Ura6	1.36716451	6.13197514	2.31E-20	4.60E-19
SPBC839.14c	efm4	elongation factor EF-1 alpha (eEF1A) l methyltransferase E	1.36491533	4.6667547	9.12E-08	4.85E-07
SPNCRNA.1400	#N/A	#N/A	1.3620331	2.55593134	0.01089474	0.02276287
SPAC922.07c	atd2	aldehyde dehydrogenase (predicted)	1.36086404	8.29717591	1.89E-56	2.63E-54
SPCTRNAGLU.10	SPCTRNAGLU.10	tRNA Glutamic acid	1.36001631	3.643985	9.81E-05	0.000324414
SPNCRNA.34	#N/A	#N/A	1.35674629	3.32923408	0.00102133	0.002735204
SPAC2E1P3.04	cao1	copper amine oxidase Cao1	1.34709436	9.23348583	3.13E-78	7.89E-76
SPBTRNAPRO.04	SPBTRNAPRO.04	tRNA Proline	1.34236192	3.37386731	0.00073962	0.002060227
SPNCRNA.1096	#N/A	#N/A	1.34002275	5.51696728	1.22E-09	8.46E-09
SPAC1F7.13c	rpl801	60S ribosomal protein L8 (predicted)	1.33750735	8.24906126	7.69E-52	8.73E-50
SPCC4G3.06c	mrpl4	mitochondrial ribosomal protein subunit L4 (predicted)	1.33459209	4.99297735	5.09E-09	3.24E-08
SPBC1289.03c	spi1	Ran GTPase Spi1	1.3296759	7.13382793	3.29E-22	7.45E-21
SPNCRNA.219	#N/A	#N/A	1.32836303	2.33064104	0.04919468	0.08544406
SPAC977.14c	SPAC977.14c	aldo/keto reductase, predicted calcium channel regulator	1.32711962	8.26251207	2.10E-50	2.20E-48
SPNCRNA.1090	#N/A	#N/A	1.32704441	7.78810013	2.87E-46	2.68E-44
SPCC1795.06	map2	P-factor pheromone Map2	1.32638033	6.19192309	3.31E-20	6.49E-19
SPAC1786.02	SPAC1786.02	phospholipase (predicted)	1.32564893	8.12396075	1.51E-37	8.15E-36
SPAC1486.07c	mrpl19	mitochondrial ribosomal protein subunit L19 (predicted)	1.3213439	3.96331338	0.00094686	0.002557917
SPCC1682.08c	mpf2	meiotic pumilio family RNA-binding protein Mpf2	1.31999572	7.35675766	1.98E-31	7.70E-30
SPBTRNAPRO.07	SPBTRNAPRO.07	tRNA Proline	1.31772521	3.56828252	0.00024621	0.000759157
SPNCRNA.232	#N/A	#N/A	1.30986083	3.91154876	0.00073045	0.002038632
SPAC23G3.04	ies4	Ino80 complex subunit Ies4	1.3097177	3.26912552	0.05537971	0.094640621
SPCC970.05	rpl3601	60S ribosomal protein L36	1.30912266	6.44658164	2.01E-18	3.27E-17
SPBCPT2R1.08c	tlh2	RecQ type DNA helicase Tlh1	1.30112753	4.21450449	1.05E-05	4.14E-05
SPAP7G5.05	rpl1002	60S ribosomal protein L10	1.29503704	7.67210255	9.02E-44	7.58E-42
SPBC3E7.07c	SPBC3E7.07c	DUF757 family protein, human PBDC1 ortholog	1.29480135	4.81703155	1.75E-07	8.92E-07
SPNCRNA.373	#N/A	#N/A	1.28816338	2.79755088	0.03637374	0.065848178
SPAC9.02c	SPAC9.02c	polyamine N-acetyltransferase (predicted)	1.2823111	5.80770409	2.34E-13	2.45E-12
SPBC887.07	mrpl38	mitochondrial ribosomal protein subunit L38 (predicted)	1.27645664	4.83690833	4.30E-08	2.42E-07
SPATRNAPHE.01	SPATRNAPHE.01	tRNA Phenylalanine	1.27176585	1.09849593	0.30990765	0.403466917
SPAC27F1.05c	SPAC27F1.05c	aminotransferase class-III, unknown specificty	1.27048205	8.11326772	2.19E-31	8.40E-30
SPNCRNA.977	#N/A	#N/A	1.27048205	8.11326772	2.19E-31	8.40E-30
SPNCRNA.1659	#N/A	#N/A	1.26955714	8.63546024	1.69E-34	8.04E-33
SPNCRNA.199	#N/A	#N/A	1.26706118	1.73049978	0.11489794	0.176456892
SPNCRNA.1022	#N/A	#N/A	1.26548891	4.53684238	5.66E-05	0.000194872
SPAC13G7.06	met16	phosphoadenosine phosphosulfate reductase	1.2650332	5.88189553	1.81E-11	1.56E-10
SPCC306.11	SPCC306.11	Schizosaccharomyces specific protein	1.2582847	7.33645311	1.11E-29	3.95E-28
SPNCRNA.1139	#N/A	#N/A	1.25765304	5.04502197	3.60E-08	2.05E-07
SPCC70.09c	mug9	DUF2406 family conserved fungal protein	1.25753687	3.00433158	0.00876657	0.018712836
SPBTRNAGLY.04	SPBTRNAGLY.04	tRNA Glycine	1.25566774	2.77594073	0.01209782	0.02497745
SPBC4F6.12	pxl1	paxillin-like protein Pxl1	1.2551951	6.86247663	7.02E-28	2.19E-26
SPCC569.07	SPCC569.07	aromatic aminotransferase (predicted)	1.2549068	7.07164449	8.57E-27	2.49E-25

SPAC6G9.09c	rpl2401	60S ribosomal protein L24 (predicted)	1.25054753	7.02240996	1.55E-27	4.78E-26
SPNCRNA.1551	#N/A	#N/A	1.24876715	2.50448719	0.02605587	0.049293917
SPAC1071.08	rpp203	60S acidic ribosomal protein A2	1.24780162	5.59608273	7.75E-11	6.10E-10
SPBPJ4664.02	SPBPJ4664.02	crazy cell surface glycoprotein	1.24745721	8.95692744	3.49E-32	1.44E-30
SPNCRNA.933	#N/A	#N/A	1.24502395	7.73887468	6.01E-42	4.45E-40
SPCTRNAPRO.09	SPCTRNAPRO.09	tRNA Proline	1.2419434	3.41795059	0.00180743	0.004570631
SPAC977.08	ayr2	1-acyl DHAP reductase Ayr2 (predicted)	1.24162291	2.3902447	0.03463569	0.063189239
SPBC1348.09	SPBC1348.09	short chain dehydrogenase, implicated in cellular detoxifica	1.24162291	2.3902447	0.03463569	0.063189239
SPNCRNA.1693	#N/A	#N/A	1.24029731	6.28373033	9.93E-18	1.52E-16
SPAC25B8.12c	SPAC25B8.12c	HAD superfamily hydrolase, unknown role	1.23560873	7.77235291	2.88E-39	1.83E-37
SPBC29A10.11c	vps902	guanyl-nucleotide exchange factor, CUE domain absent Vps	1.23523778	5.6190545	1.30E-11	1.14E-10
SPAC4H3.07c	rdl2	mitochondrial outer membrane, thiosulfate sulfurtransfera:	1.23307	6.13493376	1.52E-13	1.61E-12
SPBC685.06	rps001	40S ribosomal protein S0A (p40)	1.23202952	8.1184343	3.44E-42	2.66E-40
SPBC2F12.04	rpl1701	60S ribosomal protein L17	1.22846487	7.69658197	1.02E-38	6.14E-37
SPNCRNA.1532	#N/A	#N/A	1.22523314	5.01646725	2.45E-06	1.08E-05
SPACUNK4.09	SPACUNK4.09	methyltransferase with rhodanese domain, unknown specif	1.22474572	5.66723171	1.15E-11	1.02E-10
SPNCRNA.39	#N/A	#N/A	1.22433374	2.26772692	0.06276837	0.105235624
SPAC212.11	tlh1	RecQ type DNA helicase	1.22304995	4.16451702	5.45E-05	0.000188101
SPNCRNA.843	#N/A	#N/A	1.22280278	7.60319927	7.02E-35	3.39E-33
SPCP31B10.08c	rpl35a	60S ribosomal protein L35a	1.2222347	6.75613418	4.02E-20	7.83E-19
SPBC21C3.10c	rib7	5-amino-6-(5-phosphoribosylamino) uracil reductase Rib7 (	1.21770822	5.10713579	1.16E-06	5.30E-06
SPAC1F12.07	ser1	phosphoserine aminotransferase (predicted)	1.21732372	7.73908069	7.36E-38	4.07E-36
SPAPYUG7.06	sdu1	PPPDE peptidase family deubiquitinase/desumoylase Sdu1	1.21631072	4.55015247	8.94E-05	0.000296814
SPBC18H10.13	rps1402	40S ribosomal protein S14 (predicted)	1.21339365	7.74292304	3.66E-39	2.24E-37
SPBTRNAPRO.05	SPBTRNAPRO.05	tRNA Proline	1.21283545	3.23375227	0.00255496	0.006240661
SPAC343.14c	tif222	translation initiation factor eIF2B beta subunit	1.21252252	6.17247584	1.43E-15	1.81E-14
SPBC19C2.07	fba1	fructose-bisphosphate aldolase Fba1	1.21161148	9.24636751	5.68E-40	3.72E-38
SPBC19G7.04	spr1	DNA-protein crosslink removal protease (predicted)	1.21100153	6.48867873	7.58E-18	1.17E-16
SPRRNA.30	SPRRNA.30	5S rRNA	1.20245324	3.63298968	0.00081741	0.002242929
SPCC576.09	rps20	40S ribosomal protein S20 (predicted)	1.19968817	7.36562374	5.45E-33	2.41E-31
SPAC57A10.12c	ura3	mitochondrial dihydroorotate dehydrogenase Ura3	1.19865707	7.42129882	2.67E-27	8.03E-26
SPCC576.11	rpl15	60S ribosomal protein L15 (predicted)	1.19761897	7.94928223	2.16E-26	6.10E-25
SPAPYUG7.03c	mid2	medial ring protein, anillin Mid2	1.19743548	8.79044517	6.01E-48	5.84E-46
SPNCRNA.687	#N/A	#N/A	1.19651314	5.46663898	3.61E-11	2.99E-10
SPBC8E4.03	SPBC8E4.03	agmatinase 2 (predicted)	1.19382013	8.47362628	7.16E-49	7.39E-47
SPBC215.09c	erg10	acetyl-CoA C-acetyltransferase Erg10 (predicted)	1.19249814	8.63343143	7.02E-51	7.47E-49
SPNCRNA.559	#N/A	#N/A	1.19049816	5.69751526	9.03E-12	8.05E-11
SPNCRNA.1182	#N/A	#N/A	1.18656223	3.32852564	0.00346164	0.008196668
SPBC2F12.10	SPBC2F12.10	mitochondrial ribosomal protein subunit L35 (predicted)	1.18231503	5.96483248	3.76E-10	2.75E-09
SPAC26A3.07c	rpl1101	60S ribosomal protein L11 (predicted)	1.17962756	7.4091751	3.11E-32	1.30E-30
SPNCRNA.870	#N/A	#N/A	1.17679405	8.27864894	1.07E-42	8.64E-41
SPAC694.05c	rps2502	40S ribosomal protein S25 (predicted)	1.16964349	6.54260343	1.16E-19	2.17E-18
SPCC1884.01	SPCC1884.01	Schizosaccharomyces pombe specific protein	1.16852385	4.75530479	9.74E-06	3.86E-05
SPNCRNA.629	#N/A	#N/A	1.16839654	6.87928239	1.01E-17	1.54E-16
SPBC1921.01c	rpl35b	60S ribosomal protein L35a (predicted)	1.16526375	6.54523566	3.57E-15	4.33E-14
SPNCRNA.31	#N/A	#N/A	1.16358921	5.09830689	3.90E-08	2.21E-07
SPAC806.07	ndk1	nucleoside diphosphate kinase Ndk1	1.16277957	6.902476	8.26E-18	1.27E-16

SPCTRNAALA.12	SPCTRNAALA.12	tRNA Alanine	1.16159458	1.29952317	0.24911386	0.338908406
SPBTRNALYS.09	SPBTRNALYS.09	tRNA Lysine	1.1611739	1.98579394	0.13228101	0.199241323
SPRRNA.06	SPRRNA.06	5S rRNA	1.16102463	1.29980186	0.24452958	0.333795222
SPAC23G3.03	sib2	ornithine N5 monooxygenase (predicted)	1.15389423	6.81197625	8.30E-20	1.56E-18
SPCC1223.05c	rpl3702	60S ribosomal protein L37 (predicted)	1.15238107	6.58917747	2.33E-18	3.76E-17
SPBC2F12.07c	rpl802	60S ribosomal protein L8 (predicted)	1.1514201	7.50476207	7.21E-26	1.96E-24
SPCC965.14c	SPCC965.14c	cytosine deaminase (predicted)	1.15006732	5.81460421	1.89E-08	1.11E-07
SPAC4F8.14c	hcs1	3-hydroxy-3-methylglutaryl-CoA synthase	1.14966704	8.47147594	2.61E-44	2.22E-42
SPBP16F5.08c	fmo1	ER flavin-containing N,N-dimethylaniline monooxygenase a	1.1485533	7.51449213	2.28E-29	7.77E-28
SPAC4G9.07	mug133	UPF0300 family protein 2	1.14737802	5.39240694	4.42E-07	2.14E-06
SPCC830.06	cnb1	calcineurin regulatory subunit (calcineurin B)	1.14693283	5.48262877	3.56E-09	2.30E-08
SPRRNA.53	SPRRNA.53	5S rRNA (predicted)	1.14543487	5.10201892	7.08E-08	3.83E-07
SPNCRNA.1261	#N/A	#N/A	1.14515432	7.51833359	1.62E-32	6.86E-31
SPBC1711.06	rpl401	60S ribosomal protein L4 (predicted)	1.13536054	8.15137971	3.95E-33	1.77E-31
SPBC428.11	met17	homocysteine synthase Met17	1.13404268	5.99312266	9.61E-11	7.50E-10
SPBC428.03c	pho4	thiamine-repressible acid phosphatase Pho4	1.12933847	6.10572598	3.20E-11	2.66E-10
SPNCRNA.1410	#N/A	#N/A	1.12846437	5.38102133	1.64E-09	1.11E-08
SPAC3G6.08	erv1	mitochondrial Mia40-Erv1 disulfide relay system sulfhydryl	1.12807726	5.24118218	3.65E-07	1.78E-06
SPAC15E1.08	naa10	NatA N-acetyltransferase complex catalytic subunit Naa10	1.12776384	6.20291665	2.72E-11	2.29E-10
SPBTRNAPRO.06	SPBTRNAPRO.06	tRNA Proline	1.12706157	3.44676318	0.00246165	0.006045307
SPNCRNA.1021	#N/A	#N/A	1.12525604	8.95533862	5.98E-43	4.96E-41
SPAC222.09	seb1	RNA-binding and 3'-end processing protein Seb1	1.1240453	7.81655474	4.10E-33	1.82E-31
SPBC27.04	uds1	septation protein Uds1	1.12228344	8.22301019	1.44E-20	2.91E-19
SPAC806.03c	rps2601	40S ribosomal protein S26 (predicted)	1.12006041	6.37372161	1.75E-15	2.20E-14
SPBC3E7.12c	cfh4	chitin synthase regulatory factor Cfh4 (predicted)	1.1195082	7.66737314	3.05E-23	7.22E-22
SPCC330.14c	rpl2402	60S ribosomal protein L24 (predicted)	1.11853673	6.97376126	6.15E-20	1.17E-18
SPNCRNA.718	#N/A	#N/A	1.11799125	7.32816228	4.83E-25	1.26E-23
SPBC25H2.05	egd2	nascent polypeptide-associated complex alpha subunit Egd2	1.11625784	7.27923772	1.35E-26	3.82E-25
SPBC1A4.02c	leu1	3-isopropylmalate dehydrogenase Leu1	1.11456077	7.56924019	5.17E-30	1.88E-28
SPBC12C2.07c	srm1	spermidine synthase Srm1 (predicted)	1.11394719	8.16589943	3.41E-41	2.39E-39
SPAC227.18	lys3	saccharopine dehydrogenase Lys3	1.11211176	7.98854725	1.29E-38	7.55E-37
SPCC4G3.14	mdj1	mitochondrial DNAJ domain protein Mdj1 (predicted)	1.11175284	6.94949094	8.35E-18	1.28E-16
SPBC365.03c	rpl2101	60S ribosomal protein L21 (predicted)	1.10863657	7.45217473	3.48E-27	1.04E-25
SPCC18.14c	rpp0	60S acidic ribosomal protein Rpp0 (predicted)	1.10709382	8.63446138	1.39E-36	7.13E-35
SPNCRNA.1107	#N/A	#N/A	1.10641379	1.47629843	0.32549278	0.420010016
SPBC1711.08	aha1	chaperone activator Aha1	1.10353659	7.27441541	3.55E-16	4.72E-15
SPAC25H1.08c	sqt1	ribosome assembly protein Sqt1 (predicted)	1.10241029	7.1152539	6.49E-18	1.01E-16
SPBC839.04	rpl803	60S ribosomal protein L8 (predicted)	1.10000083	7.98175838	6.91E-37	3.62E-35
SPBC215.08c	arg4	arginine specific carbamoyl-phosphate synthase Arg4	1.09444015	9.42417736	4.63E-32	1.90E-30
SPCC191.06	SPCC191.06	Schizosaccharomyces pombe specific protein	1.09359938	6.31486599	3.56E-10	2.62E-09
SPBTRNATYR.04	SPBTRNATYR.04	tRNA Tyrosine	1.09118837	2.50327269	0.06045325	0.101786933
SPBC800.04c	rpl4301	60S ribosomal protein L37a (predicted)	1.08961925	6.20542699	3.71E-12	3.45E-11
SPAC110.04c	pss1	heat shock protein Pss1	1.08917731	9.17345046	1.42E-41	1.03E-39
SPAC24H6.08	SPAC24H6.08	Schizosaccharomyces specific protein	1.08833959	5.3808777	2.20E-06	9.74E-06
SPBC1271.04c	dys1	eIF-5A-deoxyhypusine synthase Dys1 (predicted)	1.08797526	7.3685894	1.68E-25	4.44E-24
SPNCRNA.1517	#N/A	#N/A	1.08622782	8.17223438	3.11E-39	1.92E-37
SPCC330.05c	ura4	orotidine 5'-phosphate decarboxylase Ura4	1.07802697	6.87812027	1.02E-16	1.43E-15

SPRRNA.31	SPRRNA.31	5S rRNA	1.07489613	2.29944982	0.07800655	0.126481322
SPNCRNA.1447	#N/A	#N/A	1.07264988	8.24986488	1.10E-19	2.06E-18
SPNCRNA.1201	#N/A	#N/A	1.06975674	4.89339984	2.63E-05	9.67E-05
SPAC13G6.15c	SPAC13G6.15c	calcipressin (predicted)	1.06756382	4.852735	9.26E-05	0.000306629
SPAC56F8.04c	ppt1	para-hydroxybenzoate--polyprenyltransferase Ppt1	1.06381454	6.48424087	1.53E-13	1.62E-12
SPBC2G2.17c	SPBC2G2.17c	beta-glucosidase Psu2 (predicted)	1.06077128	4.48168102	9.85E-05	0.000325396
SPAC22H12.03	SPAC22H12.03	mitochondrial hydrolase (predicted)	1.05905288	5.09898822	2.26E-05	8.38E-05
SPCC1223.07c	drs1	cytoplasmic aspartate-tRNA ligase Drs1 (predicted)	1.05730879	8.09477229	1.27E-33	5.80E-32
SPBP8B7.03c	rpl402	60S ribosomal protein L4 (predicted)	1.05707103	8.8929918	3.82E-47	3.61E-45
SPCC794.03	SPCC794.03	amino acid transmembrane transporter (predicted)	1.05579886	4.49974226	0.00017609	0.000555806
SPAC21E11.04	aca1	L-azetidine-2-carboxylic acid acetyltransferase Aca1	1.05272612	6.37100868	2.86E-12	2.69E-11
SPBC1709.01	chs2	chitin synthase homolog Chs2	1.05176433	6.4586595	1.94E-14	2.19E-13
SPCC576.08c	rps2	40S ribosomal protein S2 (predicted)	1.05129039	8.4827045	1.22E-37	6.71E-36
SPAC1F12.04c	SPAC1F12.04c	peroxisomal protein Pex11 paralog (predicted)	1.05064599	5.31664021	7.06E-08	3.82E-07
SPCC1753.04	tol1	3'(2'),5'-bisphosphate nucleotidase/inositol-1,4- bisphospha	1.05049292	7.25962195	2.35E-24	5.85E-23
SPAC31G5.17c	rps1001	40S ribosomal protein S10 (predicted)	1.04995193	7.54042	1.82E-18	2.97E-17
SPBC428.05c	arg12	argininosuccinate synthase Arg12	1.04973318	8.59011186	6.64E-37	3.50E-35
SPNCRNA.1534	#N/A	#N/A	1.04863281	3.7867999	0.00465368	0.010673946
SPAC3G9.17	new8	holo-[acyl-carrier-protein] synthase (predicted)	1.04845613	3.58106306	0.0049223	0.0112065
SPAPJ698.02c	rps002	40S ribosomal protein S0B	1.04178844	8.47348284	1.28E-32	5.50E-31
SPBC646.16	scl1	20S proteasome complex subunit alpha 1	1.03691988	6.90893983	4.53E-20	8.79E-19
SPBC11C11.07	rpl1801	60S ribosomal protein L18	1.03589885	7.8928411	1.56E-31	6.13E-30
SPNCRNA.1594	#N/A	#N/A	1.03463226	7.89421222	1.68E-31	6.56E-30
SPBC1198.02	dea2	adenine deaminase Dea2	1.03173142	6.51184547	6.77E-14	7.31E-13
SPAC926.05c	dph4	diphthamide biosynthesis protein Dph4 (predicted)	1.02531057	4.98782567	5.57E-05	0.000191829
SPCC16A11.09c	tim23	TIM23 translocase complex subunit Tim23 (predicted)	1.02433834	6.37686365	1.34E-14	1.54E-13
SPBC17G9.09	tif213	translation initiation factor eIF2 gamma subunit	1.02341646	8.29141366	1.02E-36	5.26E-35
SPCC1259.05c	cox9	cytochrome c oxidase subunit VIIa (predicted)	1.02290683	5.04030794	2.78E-05	0.000101283
SPBC1105.09	ubc15	ubiquitin conjugating enzyme E2 Ubc15	1.01860535	5.49740956	1.03E-08	6.26E-08
SPCC11E10.06c	elp4	elongator complex subunit Elp4	1.01797042	5.75158394	1.73E-09	1.17E-08
SPAC16C9.05	cph1	Clr6 histone deacetylase associated PHD protein-1 Cph1	1.015408	6.5290478	4.75E-12	4.34E-11
SPBC409.06	uch2	ubiquitin C-terminal hydrolase Uch2	1.01453279	6.69845434	1.70E-15	2.14E-14
SPAC15F9.03c	nxt2	nuclear import receptor Nxt2	1.01446676	6.06128354	7.05E-12	6.37E-11
SPBC1778.01c	zuo1	ribosome-associated chaperone, zuotin (predicted)	1.01219019	7.56976775	3.52E-27	1.05E-25
SPBP16F5.07	apm1	AP-1 adaptor complex mu subunit Apm1	1.01214832	6.55424527	1.14E-15	1.46E-14
SPBC1773.10c	nrs1	cytoplasmic asparagine-tRNA ligase Nrs1 (predicted)	1.0111216	8.38769106	1.39E-34	6.67E-33
SPAC3H1.07	aru1	arginase Aru1	1.01055058	8.09707606	2.65E-33	1.20E-31
SPAPB1E7.12	rps602	40S ribosomal protein S6	1.01050972	7.26788026	6.02E-23	1.41E-21
SPRRNA.03	SPRRNA.03	5S rRNA	1.00813068	4.16539554	0.00040252	0.001187306
SPAC11E3.04c	ubc13	ubiquitin conjugating enzyme E2 Ubc13	1.00779826	6.27320537	1.84E-12	1.78E-11
SPNCRNA.1507	#N/A	#N/A	1.0076137	5.41125114	4.09E-08	2.31E-07
SPAC922.03	SPAC922.03	1-aminocyclopropane-1-carboxylate deaminase (predicted)	1.00667497	7.1855607	1.97E-19	3.60E-18
SPCP1E11.03	mug170	arrestin family Schizosaccharomyces specific protein Mug1	1.00645545	5.29216463	1.51E-06	6.82E-06
SPNCRNA.616	#N/A	#N/A	1.00617399	5.15474043	1.66E-06	7.44E-06
SPNCRNA.1340	#N/A	#N/A	1.00590688	2.68091978	0.13445506	0.201845725
SPBC25H2.16c	gga22	Golgi localized Arf binding gamma-adaptin ortholog Gga22	1.0043333	7.0118915	2.42E-16	3.28E-15
SPCC962.06c	bpb1	KH and CC/hC domain splicing factor Bpb1	1.00263761	7.07095837	1.15E-20	2.34E-19

SPNCRNA.1601	#N/A	#N/A	1.00257527	4.59432116	0.00020584	0.000644031
SPAC222.04c	ies6	Ino80 complex subunit Ies6	1.00041828	5.82676985	2.45E-06	1.08E-05
SPAC977.07c	pfl6	cell surface glycoprotein, flocculin Pfl6	0.99926815	3.31307416	0.01273224	0.026160288
SPBC30D10.02	ncb2	transcription corepressor Ncb2 (predicted)	0.99809751	5.57985426	9.57E-08	5.07E-07
SPBC16H5.02	pfk1	6-phosphofructokinase pfk1	0.9979864	9.82445822	2.60E-40	1.74E-38
SPCTRNAASP.06	SPCTRNAASP.06	tRNA Asparagine	0.9977953	4.24085095	0.00029209	0.000884195
SPNCRNA.932	#N/A	#N/A	0.99626118	4.39192053	0.01569485	0.03154267
SPBC17D1.17	tam11	Schizosaccharomyces specific protein Tam11	0.99619065	5.99590957	2.36E-11	2.01E-10
SPAC631.01c	acp2	F-actin capping protein beta subunit Acp2	0.99511345	6.63269473	1.74E-14	1.97E-13
SPAC22G7.09c	nup45	nucleoporin Nup45	0.99454799	6.76676347	7.37E-14	7.93E-13
SPBC16E9.05	erg6	sterol 24-C-methyltransferase Erg6	0.99450056	8.29134292	1.16E-32	5.00E-31
SPBC14C8.01c	cut2	securin, sister chromatid separation inhibitor	0.99430355	5.26106372	1.97E-06	8.72E-06
SPAP11E10.01	SPAP11E10.01	ornithine cyclodeaminase-like protein (predicted)	0.99360902	6.38885851	4.36E-12	4.01E-11
SPBP4H10.14c	SPBP4H10.14c	Schizosaccharomyces specific protein	0.99348707	5.51831767	6.07E-05	0.000207581
SPBP8B7.17c	SPBP8B7.17c	phosphomethylpyrimidine kinase (predicted)	0.9930838	6.15437314	4.00E-12	3.71E-11
SPAC521.05	rps802	40S ribosomal protein S8 (predicted)	0.98972329	7.73886956	2.10E-24	5.26E-23
SPBC1348.08c	SPBC1348.08c	cell surface glycoprotein, adhesion molecule (predicted)	0.98782854	3.4030565	0.01028985	0.021625215
SPAC644.16	rna15	RNA-binding protein Rna15	0.98723769	7.06597283	3.13E-18	5.03E-17
SPBC428.04	apq12	nuclear membrane organization protein Apq12 (predicted)	0.98627285	3.97134133	0.00761263	0.016492827
SPCC74.05	rpl2702	60S ribosomal protein L27 (predicted)	0.98588038	7.28830414	2.26E-19	4.10E-18
SPCC13B11.02c	SPCC13B11.02c	Schizosaccharomyces pombe specific protein	0.98484965	9.65980678	1.25E-37	6.80E-36
SPBC3B9.05	hot15	helper of TIM Hot15 (predicted)	0.98376741	4.55334999	0.00041588	0.001222485
SPCC13B11.01	adh1	alcohol dehydrogenase Adh1	0.98376552	9.65775092	5.91E-38	3.30E-36
SPAC23A1.12c	frs1	cytoplasmic phenylalanine-tRNA ligase beta subunit Frs1 (p	0.98345782	8.28457668	6.40E-32	2.56E-30
SPAC23C4.02	crn1	actin binding protein, coronin Crn1	0.98103422	7.70421247	2.49E-23	5.97E-22
SPCC736.13	SPCC736.13	short chain dehydrogenase (predicted)	0.98026262	7.22978125	2.52E-15	3.10E-14
SPBC839.05c	rps1701	40S ribosomal protein S17 (predicted)	0.98020264	7.05715112	4.04E-15	4.89E-14
SPNCRNA.1245	#N/A	#N/A	0.97924981	7.29596741	1.45E-19	2.67E-18
SPCC5E4.05c	mgl1	mitochondrial acylglycerol lipase Mgl1 (predicted)	0.97912412	6.88463557	5.39E-14	5.87E-13
SPAC4H3.06	SPAC4H3.06	human REX1BD ortholog, implicated in DNA repair	0.97906666	6.58431254	8.92E-11	6.98E-10
SPNCRNA.171	#N/A	#N/A	0.97854697	2.13265545	0.13396433	0.201331107
SPBC31F10.05	mug37	conserved fungal protein	0.97810155	6.92023723	4.17E-17	6.02E-16
SPBC23E6.06c	rib3	3,4-dihydroxy-2-butanone 4-phosphate synthase Rib3 (predi	0.9778729	6.70359932	7.11E-16	9.24E-15
SPBC31F10.06c	sar1	ADP-ribosylation factor Sar1	0.97773837	7.58118316	1.09E-26	3.15E-25
SPCC965.09	SPCC965.09	mitochondrial omega-amidase (predicted)	0.97477673	6.30807652	2.22E-11	1.90E-10
SPNCRNA.1561	#N/A	#N/A	0.97322175	4.40237821	0.00041246	0.001214014
SPAC5H10.05c	SPAC5H10.05c	FAD binding oxidoreductase, implicated in cellular detoxific	0.97267126	7.0239829	4.34E-19	7.58E-18
SPCC330.03c	SPCC330.03c	NADPH-hemoprotein reductase (predicted)	0.97201622	7.31113788	1.33E-17	2.00E-16
SPCC1223.14	aro2	chorismate synthase Aro2 (predicted)	0.97195307	7.85261939	1.82E-27	5.54E-26
SPBC106.11c	plg7	phospholipase A2, PAF family homolog	0.97160946	6.4144461	5.50E-10	3.95E-09
SPNCRNA.300	#N/A	#N/A	0.97112871	3.72773988	0.00426352	0.00984836
SPAC17A2.14	mnr2	vacuolar CorA family magnesium ion transmembrane trans	0.97107785	7.28111136	6.27E-21	1.33E-19
SPBC17G9.10	rpl1102	60S ribosomal protein L11 (predicted)	0.96991575	7.73309479	6.42E-24	1.57E-22
SPCC584.01c	met10	sulfite reductase NADPH flavoprotein subunit (predicted)	0.96875739	8.64227808	3.07E-38	1.77E-36
SPCC285.05	SPCC285.05	purine nucleoside transmembrane transporter (predicted)	0.96730568	5.89297178	1.41E-07	7.27E-07
SPAC6C3.02c	mix17	mitochondrial CHCH domain protein Mix17 (predicted)	0.96686831	6.8253505	9.33E-15	1.09E-13
SPNCRNA.1533	#N/A	#N/A	0.96553204	5.0069293	0.00025986	0.00079655

SPNCRNA.1614	#N/A	#N/A	0.96407424	4.96035345	2.01E-05	7.53E-05
SPAC23H3.09c	gly1	threonine aldolase Gly1 (predicted)	0.96395799	8.16854949	7.71E-26	2.08E-24
SPNCRNA.947	#N/A	#N/A	0.9638959	5.02651406	0.00012428	0.000403485
SPAC1805.02c	etf2	electron transfer flavoprotein beta subunit EtfB (predicted)	0.96365946	7.06420618	4.01E-16	5.31E-15
SPCC14G10.03c	ump1	proteasome maturation factor Ump1 (predicted)	0.9624085	5.97752258	1.96E-09	1.30E-08
SPAC9E9.12c	ybt1	ABC transmembrane transporter Ybt1	0.96135194	8.04639496	3.04E-26	8.48E-25
SPBC365.01	csr103	sec14 cytosolic factor family, phospholipid-intermembrane	0.96080235	6.75560581	5.31E-16	6.95E-15
SPBC16C6.11	rpl3201	60S ribosomal protein L32	0.96001843	7.15321117	8.54E-19	1.45E-17
SPCC613.08	SPCC613.08	CDK regulator, involved in ribosome export (predicted)	0.95963316	5.64679243	7.47E-07	3.50E-06
SPCTRNAASP.05	SPCTRNAASP.05	tRNA Asparagine	0.95900862	4.21623472	0.0005357	0.001538756
SPAC869.10c	put4	plasma membrane proline transmembrane transporter Put4	0.95825216	7.78665052	1.15E-22	2.67E-21
SPAC57A10.06	mug15	Schizosaccharomyces specific protein Mug15	0.95306741	2.75255497	0.05069522	0.087781412
SPCC191.09c	gst1	glutathione S-transferase Gst1	0.95212299	2.96610737	0.05809285	0.098485767
SPAC5H10.04	SPAC5H10.04	NADPH dehydrogenase, (Old yellow enzyme) involved in sm	0.95193191	7.03245142	2.68E-18	4.31E-17
SPNCRNA.90	#N/A	#N/A	0.95136528	1.42019484	0.28779001	0.380513323
SPNCRNA.411	#N/A	#N/A	0.95066896	4.3878267	0.00053252	0.00153033
SPBC4C3.03	thr1	homoserine kinase Thr1 (predicted)	0.95028926	6.66038645	2.51E-14	2.82E-13
SPNCRNA.715	#N/A	#N/A	0.94865002	7.36374982	3.26E-19	5.74E-18
SPBC29B5.03c	rpl26	60S ribosomal protein L26 (predicted)	0.94646711	7.42477529	1.73E-18	2.84E-17
SPNCRNA.1452	#N/A	#N/A	0.94560809	2.23564531	0.1585808	0.231675047
SPAC343.12	rds1	ferritin related conserved fungal protein	0.94446256	8.9841698	8.52E-15	9.99E-14
SPBC1685.02c	rps1202	40S ribosomal protein S12 (predicted)	0.94382294	7.48507537	5.88E-21	1.25E-19
SPAC2C4.08	SPAC2C4.08	conserved fungal protein	0.94200291	4.34960263	0.00091718	0.002487619
SPCC1450.07c	dao1	D-amino acid oxidase	0.94184811	6.50651979	1.27E-09	8.80E-09
SPBC18H10.04c	sce3	translation initiation factor (predicted)	0.94109383	8.15444962	1.44E-20	2.91E-19
SPNCRNA.361	#N/A	#N/A	0.94076658	4.09573879	0.00157859	0.004047594
SPAC17G6.02c	tco1	RTA1-like protein	0.93931711	7.15524027	4.90E-11	3.97E-10
SPNCRNA.878	#N/A	#N/A	0.93697474	7.28511415	8.88E-21	1.85E-19
SPNCRNA.1091	#N/A	#N/A	0.93629437	7.78466246	1.72E-21	3.81E-20
SPBC1861.01c	cnp3	CENP-C ortholog Cnp3	0.935983	7.37976248	3.44E-17	5.02E-16
SPAC4H3.10c	pyk1	pyruvate kinase	0.93567673	10.1376113	7.71E-42	5.64E-40
SPNCRNA.530	#N/A	#N/A	0.93486532	3.63220346	0.00855935	0.018299214
SPBC21C3.16c	spt4	DSIF transcription elongation factor complex subunit Spt4	0.93369361	4.99625307	5.98E-05	0.000205084
SPAC3A12.16c	tim17	TIM23 translocase complex subunit Tim17 (predicted)	0.93348552	5.31887325	3.48E-06	1.49E-05
SPNCRNA.1346	#N/A	#N/A	0.93258401	4.61060114	0.01105096	0.023046819
SPBC776.03	SPBC776.03	homoserine dehydrogenase (predicted)	0.93169822	8.18145584	1.76E-27	5.40E-26
SPBTRNAGLU.06	SPBTRNAGLU.06	tRNA Glutamic acid	0.93138301	4.54806548	0.00015187	0.000485868
SPBTRNAGLU.07	SPBTRNAGLU.07	tRNA Glutamic acid	0.93138301	4.54806548	0.00015187	0.000485868
SPAC1002.12c	SPAC1002.12c	succinate-semialdehyde dehydrogenase [NAD(P)+] (predicte	0.93108234	8.80131896	8.87E-36	4.41E-34
SPNCRNA.1117	#N/A	#N/A	0.93078041	5.02783186	7.45E-05	0.000250835
SPNCRNA.1209	#N/A	#N/A	0.92792999	3.00482829	0.0438383	0.077445382
SPBC1778.08c	arc3	ARP2/3 actin-organizing complex subunit Arc21	0.92781483	6.94189268	8.46E-16	1.09E-14
SPBC16G5.14c	rps3	40S ribosomal protein S3 (predicted)	0.92688722	8.29336325	8.84E-25	2.27E-23
SPBC29A3.13	pdp1	PWWP domain protein Pdp1	0.92645825	7.92733006	1.45E-23	3.52E-22
SPBC2A9.04c	san1	sir antagonist, ubiquitin-protein ligase E3	0.92299092	6.47396805	8.85E-11	6.93E-10
SPCC1450.08c	wtf16	wtf element Wtf16	0.92283781	3.11486739	0.0300268	0.055782791
SPCP1E11.09c	rpp103	60S acidic ribosomal protein Rpp1-3	0.92208878	6.8357958	1.16E-14	1.34E-13

SPATRNAGLU.03	SPATRNAGLU.03	tRNA Glutamic acid	0.92076449	4.14060308	0.00140943	0.0036496
SPAC4G9.20c	ymc1	mitochondrial carrier, glutamate/glycine (predicted)	0.92007742	5.67992669	5.93E-06	2.45E-05
SPCC645.08c	snd1	RNA-binding protein, involved in chromatin silencing by sma	0.91903972	8.09960011	1.49E-16	2.05E-15
SPATRNAASP.01	SPATRNAASP.01	tRNA Aspartic acid	0.91758982	4.47278734	0.00030084	0.000909867
SPBC839.17c	fkh1	FKBP-type peptidyl-prolyl cis-trans isomerase Fkh1	0.91572296	6.76653997	2.90E-11	2.44E-10
SPCC31H12.07	sec231	COPII cargo receptor subunit Sec23a (predicted)	0.91467578	8.52291434	3.00E-27	8.99E-26
SPAC56F8.05c	mug64	Golgi BAR domain protein (predicted)	0.91333268	7.3956145	1.12E-17	1.69E-16
SPAC8E11.03c	dmc1	RecA family ATPase Dmc1	0.9121835	5.58296035	9.47E-06	3.76E-05
SPCC1322.04	fyu1	UTP-glucose-1-phosphate uridylyltransferase Fyu1	0.91211792	9.20194817	9.37E-19	1.58E-17
SPAC8C9.05	dtd1	D-Tyr-tRNA deacylase Dtd1 (predicted)	0.91122564	6.58389413	8.64E-10	6.07E-09
SPCC1322.11	rpl2302	60S ribosomal protein L23	0.91027341	7.56080105	1.09E-20	2.24E-19
SPAC29A4.15	srs1	cytoplasmic serine-tRNA ligase Srs1 (predicted)	0.9086581	7.5062761	2.76E-19	4.92E-18
SPCC622.18	rpl6	60S ribosomal protein L6 (predicted)	0.90754039	8.42686882	1.85E-27	5.60E-26
SPBC23G7.09	mat1-Mc	M-specific trancription factor Mc	0.90682472	5.66828461	8.20E-08	4.40E-07
SPAC11D3.03c	SPAC11D3.03c	aminomethyltransferase-like and DUF1989 family protein, i	0.90579925	6.64540904	7.23E-12	6.53E-11
SPNCRNA.1455	#N/A	#N/A	0.90460996	1.73095018	0.39495456	0.490271034
SPBC18E5.07	aim21	barbed end F-actin assembly inhibitor	0.90285548	8.09827006	9.85E-22	2.21E-20
SPBC56F2.12	ilv5	acetohydroxyacid reductoisomerase (predicted)	0.90284801	9.12199123	2.33E-39	1.50E-37
SPAC6F6.09	eaf6	Mst2/NuA4 histone acetyltransferase complex subunit Eaf6	0.9022789	4.46734827	0.00313113	0.007492022
SPAC23H4.02	ppk9	serine/threonine protein kinase Ppk9 (predicted)	0.90222617	7.14193063	6.30E-13	6.35E-12
SPBC902.06	mto2	gamma tubulin complex linker Mto2	0.90190364	5.92895357	7.23E-05	0.000244017
SPNCRNA.880	#N/A	#N/A	0.90145634	4.85477296	0.00024691	0.000760968
SPNCRNA.703	#N/A	#N/A	0.9010358	4.48786041	0.0036389	0.008532983
SPBC16A3.08c	oga1	Stm1 homolog Oga1	0.90035635	8.77763499	1.31E-30	4.86E-29
SPAC17A5.15c	gus1	cytoplasmic glutamate-tRNA ligase Gus1 (predicted)	0.89909443	9.03778602	1.59E-32	6.76E-31
SPBC3E7.10	fma1	methionine aminopeptidase Fma1 (predicted)	0.89889413	7.286858	4.88E-16	6.41E-15
SPNCRNA.565	#N/A	#N/A	0.89878955	4.81455893	0.0061648	0.013700672
SPAC222.17	SPAC222.17	conserved fungal protein	0.89848176	4.25816215	0.00171603	0.00437199
SPAC1071.09c	SPAC1071.09c	nucleolar DNAJ domain protein, DNAJC9 family, implicated	0.89789082	7.09079519	6.66E-14	7.20E-13
SPBC1685.01	pmp1	dual-specificity MAP kinase phosphatase Pmp1	0.89766487	7.73167091	1.99E-19	3.61E-18
SPNCRNA.50	#N/A	#N/A	0.89740713	2.38982198	0.12822098	0.193898615
SPBC3B8.03	lys9	saccharopine dehydrogenase	0.89724461	8.81697419	1.19E-24	3.03E-23
SPNCRNA.1359	#N/A	#N/A	0.89688122	4.09451762	0.00606035	0.013506769
SPAC9E9.11	plr1	pyridoxal reductase Plr1	0.89643436	8.23410377	4.54E-23	1.07E-21
SPNCRNA.1578	#N/A	#N/A	0.89516456	6.67184159	8.37E-13	8.35E-12
SPBC21C3.12c	SPBC21C3.12c	thioredoxin family protein, peroxidase	0.89380833	5.97792427	6.60E-06	2.70E-05
SPBC713.12	erg1	squalene monooxygenase Erg1 (predicted)	0.89367098	8.25508243	5.93E-16	7.75E-15
SPBC405.07	rpl3602	60S ribosomal protein L36	0.89150186	6.736293	2.03E-13	2.13E-12
SPAC343.20	#N/A	#N/A	0.89144229	8.94681863	9.02E-15	1.05E-13
SPCC4G3.15c	not2	CCR4-Not complex NOT box subunit Not2	0.89108525	6.22744153	3.10E-10	2.30E-09
SPCC1682.10	rpn8	19S proteasome regulatory subunit Rpn8	0.89008923	7.4086467	3.78E-18	6.04E-17
SPAC22A12.16	acl2	ATP-citrate synthase subunit 2 (predicted)	0.88989551	8.56482732	3.35E-29	1.12E-27
SPAC31G5.04	lys12	homoisocitrate dehydrogenase Lys12	0.88947788	8.01827531	1.74E-24	4.37E-23
SPBC1711.02	mat3-Mc	mating type M-specific HMG-box transcription factor Mc at	0.88818992	5.67427144	1.25E-07	6.51E-07
SPAC644.15	rpp101	60S acidic ribosomal protein A1	0.8877228	6.8542963	2.09E-15	2.60E-14
SPCTRNAASP.08	SPCTRNAASP.08	tRNA Asparagine	0.88465794	4.47305286	0.00046572	0.00135843
SPBC800.09	sum2	translation initiation inhibitor (predicted)	0.88462637	7.35868463	5.03E-16	6.60E-15

SPNCRNA.579	#N/A	#N/A	0.88459618	5.02712244	0.00015651	0.000500019
SPNCRNA.668	#N/A	#N/A	0.88290768	4.89086144	7.16E-05	0.000242105
SPCC576.01c	xan1	alpha-ketoglutarate-dependent xanthine dioxygenase Xan1	0.8812039	6.0739163	1.09E-09	7.56E-09
SPBC19F5.03	sac11	inositol polyphosphate phosphatase (predicted)	0.88038099	7.98863903	5.72E-25	1.48E-23
SPAC3G9.06	frs2	cytoplasmic phenylalanine-tRNA ligase alpha subunit Frs2 (	0.88034504	8.17494776	1.08E-20	2.22E-19
SPBC685.07c	rpl2701	60S ribosomal protein L27	0.87925982	7.48868103	1.37E-19	2.53E-18
SPATRNAGLU.02	SPATRNAGLU.02	tRNA Glutamic acid	0.87871904	4.53478668	0.00034233	0.001026231
SPNCRNA.955	#N/A	#N/A	0.87437576	5.29839516	3.43E-06	1.47E-05
SPNCRNA.686	#N/A	#N/A	0.87316598	6.92749097	4.89E-11	3.96E-10
SPBC18H10.12c	rpl701	RNase MRP subunit, ribosomal protein L7-like Rpl701	0.87299772	8.43672009	3.57E-23	8.45E-22
SPBC29A3.04	rpl8	60S ribosomal protein L7a/L8 (predicted)	0.87240981	8.58562639	1.61E-29	5.62E-28
SPAC16C9.02c	SPAC16C9.02c	S-methyl-5-thioadenosine phosphorylase (predicted)	0.87115121	6.92254547	4.78E-11	3.89E-10
SPNCRNA.1562	#N/A	#N/A	0.86882705	4.33575321	0.00132501	0.003458637
SPATRNAGLU.01	SPATRNAGLU.01	tRNA Glutamic acid	0.86784928	4.52855282	0.00044831	0.001311571
SPBC19C7.11	SPBC19C7.11	plasma membrane ClC chloride channel (predicted)	0.86502601	7.07537326	3.15E-11	2.63E-10
SPBC16D10.02	trm11	tRNA (guanine-N2-)-methyltransferase catalytic subunit Trı	0.86396384	5.3822916	4.22E-06	1.78E-05
SPAC589.06c	snd302	SRP-independent ER targeting protein Snd3b (predicted)	0.86293876	7.54629349	9.34E-18	1.43E-16
SPCC1442.16c	zta1	NADPH quinone oxidoreductase/ARE-binding protein (predi	0.8621959	7.11389654	5.04E-14	5.53E-13
SPNCRNA.1470	#N/A	#N/A	0.86010365	7.04298533	3.01E-11	2.53E-10
SPBC1198.12	mfr1	meiotic APC activator Mfr1	0.85987716	5.56776895	7.14E-07	3.36E-06
SPAC22A12.17c	SPAC22A12.17c	short chain dehydrogenase (predicted)	0.85774353	8.62067464	8.12E-27	2.37E-25
SPBP8B7.06	rpp201	60S acidic ribosomal protein A2	0.85644341	7.0913484	3.26E-15	3.98E-14
SPBC13G1.08c	ash2	Ash2-trithorax family protein	0.85567944	7.00895088	1.68E-09	1.14E-08
SPBC887.06c	snx3	sorting nexin Snx3 (predicted)	0.85363551	5.03766299	0.00011603	0.000378331
SPBC1105.05	exg1	cell wall glucan 1,6-beta-glucosidase Exg1	0.85297515	7.70653569	3.56E-16	4.73E-15
SPAC27F1.06c	ani2	CENP-A amino terminus domain (NTD) isomerase Ani2	0.85291038	6.45746903	9.42E-10	6.59E-09
SPBC15D4.15	pho2	4-phosphoerythronate phosphastase/2-phosphoglycolate pł	0.85168997	6.69508572	1.56E-11	1.35E-10
SPBC106.18	rpl2501	60S ribosomal protein L25 (predicted)	0.85104242	7.60952606	1.58E-15	1.99E-14
SPATRNAGLU.04	SPATRNAGLU.04	tRNA Glutamic acid	0.84963624	4.14959207	0.00331427	0.007872102
SPBTRNAGLU.05	SPBTRNAGLU.05	tRNA Glutamic acid	0.84963624	4.14959207	0.00331427	0.007872102
SPCC70.08c	SPCC70.08c	methyltransferase (predicted)	0.84855096	6.10539353	5.36E-07	2.57E-06
SPCC663.11	saf1	splicing associated factor Saf1	0.84802591	4.72182693	0.00081277	0.002231113
SPRRNA.18	SPRRNA.18	5S rRNA	0.84801457	4.81202095	0.00027589	0.000841151
SPCC191.03c	SPCC191.03c	Schizosaccharomyces pombe specific protein	0.84209927	6.01687583	4.42E-07	2.14E-06
SPNCRNA.635	#N/A	#N/A	0.84199196	5.54856854	8.49E-06	3.41E-05
SPCC4G3.11	nur1	Lem2-Nur1 complex subunit Nur1	0.84128148	4.57329597	0.00145953	0.003765018
SPNCRNA.1465	#N/A	#N/A	0.84027731	7.88155398	1.10E-21	2.45E-20
SPCC364.03	rpl1702	60S ribosomal protein L17 (predicted)	0.84024058	7.90459784	5.03E-21	1.09E-19
SPCC306.09c	cap1	adenylyl cyclase-associated protein Cap1	0.83924649	8.67182915	1.83E-28	5.95E-27
SPAC343.02	img1	mitochondrial ribosomal protein subunit L19 (predicted)	0.83786904	5.50623662	2.09E-06	9.25E-06
SPAC1039.02	SPAC1039.02	extracellular 5'-nucleotidase, human NT5E family (predicte	0.83772039	5.7847411	6.87E-05	0.000233103
SPAC27E2.02	yih1	IMPACT homolog, cytoplasmic translational regulator Yih1 (	0.83760179	5.33366494	7.32E-06	2.98E-05
SPBC18H10.14	rps1601	40S ribosomal protein S16 (predicted)	0.83729107	7.87777197	1.63E-21	3.61E-20
SPAC8C9.09c	mug129	Schizosaccharomyces specific protein Mug129	0.83713036	5.33937398	2.66E-05	9.76E-05
SPCC1739.13	ssa2	heat shock protein Ssa2	0.83680988	10.1919938	6.79E-30	2.46E-28
SPBC21C3.13	rps1901	40S ribosomal protein S19 (predicted)	0.83669219	7.69448917	9.42E-19	1.59E-17
SPAC29A4.02c	SPAC29A4.02c	translation elongation factor EF-1 gamma subunit	0.83586672	9.0229084	3.16E-29	1.06E-27

SPAC2F7.13c	wrs1	cytoplasmic tryptophan-tRNA ligase Wrs1 (predicted)	0.83586073	7.45355198	4.95E-17	7.12E-16
SPBC6B1.08c	ofd1	HIF prolyl-3,4-dihydroxylase Ofd1	0.83579972	7.36973407	1.57E-16	2.16E-15
SPBC18E5.04	rpl1001	60S ribosomal protein L10	0.83456245	8.47216538	1.25E-25	3.31E-24
SPBC530.09c	mrl1	cation dependent mannose-6-phosphate cargo receptor Mrl	0.83391406	6.0304585	5.43E-07	2.60E-06
SPCC1183.08c	rpl101	60S ribosomal protein L10a	0.8337095	8.36150803	2.26E-26	6.36E-25
SPNCRNA.1246	#N/A	#N/A	0.833564	4.29125785	0.0055289	0.01241721
SPCC613.05c	rpl35	60S ribosomal protein L35	0.83293641	7.53517791	4.56E-19	7.92E-18
SPAC17A2.01	bsu1	plasma membrane high-affinity import carrier for pyridoxin	0.83284723	5.66684755	2.64E-06	1.16E-05
SPNCRNA.904	#N/A	#N/A	0.83202122	5.73507973	1.28E-05	4.96E-05
SPCC338.14	ado1	adenosine kinase (predicted)	0.83163395	8.07588826	2.87E-20	5.69E-19
SPAC3G9.04	ssu72	protein phosphatase Ssu72	0.83048767	4.42571739	0.00315342	0.007537419
SPAC959.07	rps403	40S ribosomal protein S4 (predicted)	0.82997295	8.33624648	3.37E-24	8.31E-23
SPNCRNA.136	#N/A	#N/A	0.82990147	4.83406522	0.00074853	0.002079076
SPNCRNA.601	#N/A	#N/A	0.82990147	4.83406522	0.00074853	0.002079076
SPAC1F8.08	SPAC1F8.08	Schizosaccharomyces pombe specific protein	0.82958359	5.01079998	4.74E-05	0.000165628
SPBC1539.06	acb1	fatty-acyl-CoA binding protein Acb1 (predicted)	0.82662676	6.18464278	2.59E-07	1.29E-06
SPAC144.03	ade2	adenylosuccinate synthetase Ade2	0.82625713	8.37735444	7.21E-24	1.75E-22
SPBC28F2.03	ppi1	cyclophilin family peptidyl-prolyl cis-trans isomerase Cyp2	0.82598111	7.93144938	7.13E-20	1.35E-18
SPAC8E11.08c	#N/A	#N/A	0.82453261	2.44711585	0.18880807	0.267563286
SPNCRNA.1454	#N/A	#N/A	0.82453051	6.30795066	1.83E-06	8.16E-06
SPBC106.16	pre6	20S proteasome complex subunit alpha 4 Pre6	0.82443008	7.02308738	4.68E-13	4.76E-12
SPAC57A7.12	ssz1	heat shock protein Ssz1 (predicted)	0.82287242	8.48116335	3.41E-24	8.39E-23
SPBC1347.13c	mrm1	mitochondrial ribose methyltransferase Mrm1 (predicted)	0.82187683	4.32921675	0.00320934	0.007660303
SPRRNA.40	SPRRNA.40	5S rRNA	0.82027435	4.52262953	0.00189449	0.004776588
SPAC212.06c	SPAC212.06c	DNA helicase in rearranged telomeric region, truncated	0.81918029	2.16817958	0.23474194	0.32212521
SPAC3C7.11c	cnx1	calnexin Cnx1	0.81903583	9.09681196	2.84E-30	1.04E-28
SPNCRNA.707	#N/A	#N/A	0.8165282	6.40100575	1.36E-09	9.35E-09
SPCC1682.14	rpl1902	60S ribosomal protein L19	0.81567585	7.78892005	6.74E-18	1.04E-16
SPAC1250.05	rpl3002	60S ribosomal protein L30 (predicted)	0.81466327	5.21724675	0.0006899	0.001936796
SPAPB24D3.07c	SPAPB24D3.07c	Schizosaccharomyces pombe specific protein	0.81381448	9.20553246	4.02E-21	8.74E-20
SPBC11C11.02	imp2	contractile ring protein Imp2	0.81250838	7.33593947	5.75E-12	5.24E-11
SPCTRNAHIS.03	SPCTRNAHIS.03	tRNA Histidine	0.81208656	1.82003344	0.3083086	0.401692512
SPCC126.03	pus1	TruA family tRNA/ U2 snRNA pseudouridine synthase Lsp1	0.81184566	6.31841103	4.30E-09	2.76E-08
SPAC140.01	sdh2	succinate dehydrogenase (ubiquinone) iron-sulfur protein su	0.81166274	7.44454389	7.53E-16	9.74E-15
SPNCRNA.446	#N/A	#N/A	0.81070206	4.29647121	0.00284186	0.006869309
SPBC4B4.04	eif21	translation initiation factor eIF2A (predicted)	0.80983207	7.92282652	6.88E-20	1.31E-18
SPAC1002.07c	SPAC1002.07c	spermidine/spermine N1-acetyltransferase (predicted)	0.80912126	6.43551557	5.47E-09	3.47E-08
SPCC4G3.02	aph1	bis(5'-nucleosidyl)-tetraphosphatase	0.80909241	5.02968769	0.00029176	0.000883594
SPAC31G5.02	rot1	ER chaperone Rot1 (predicted)	0.80859726	6.40370255	8.47E-10	5.95E-09
SPAC186.07c	SPAC186.07c	hydroxyacid dehydrogenase, implicated in cellular detoxifica	0.80815518	1.68352635	0.2877378	0.380513323
SPAC222.08c	sno1	glutamine aminotransferase subunit Sno1 (predicted)	0.80728834	6.74974344	9.49E-09	5.81E-08
SPBC14F5.06	rli1	iron-sulfur ATPase involved in ribosome biogenesis and trar	0.8066652	8.03566151	2.48E-19	4.45E-18
SPBC17G9.07	rps2402	40S ribosomal protein S24 (predicted)	0.80652917	7.7244817	8.86E-18	1.36E-16
SPBC23E6.05	arx1	ribosomal export complex protein Arx1, peptidase family (p	0.8065111	6.54702846	2.19E-09	1.45E-08
SPAC21E11.07	iba57	mitochondrial [4Fe-4S] cluster asssembly protein Iba57 (pre	0.80600341	4.81380591	0.00257688	0.006278439
SPNCRNA.1161	#N/A	#N/A	0.80596715	2.9839961	0.08355893	0.134520589
SPBC14F5.04c	pgk1	phosphoglycerate kinase Pgk1 (predicted)	0.80587799	9.93012281	9.02E-29	2.98E-27

SPBC2G5.05	SPBC2G5.05	transketolase (predicted)	0.80465807	9.98232663	4.36E-28	1.37E-26
SPBC32H8.06	mug93	TPR repeat protein, meiotically spliced	0.80398316	3.64340946	0.06055471	0.101923028
SPAC227.16c	psf3	GINS complex subunit Psf3	0.80279444	5.01927941	0.00013157	0.000425343
SPAC30C2.02	mmd1	deoxyhypusine hydroxylase	0.80180834	6.27311958	1.21E-08	7.30E-08
SPBC30D10.18c	rpl102	60S ribosomal protein L10a	0.80103266	6.94993856	2.53E-12	2.40E-11
SPBC2G2.03c	sbh1	translocon beta subunit Sbh1 (predicted)	0.80091521	6.07438556	4.86E-06	2.04E-05
SPAC22A12.05	rpc11	DNA-directed RNA polymerase III complex subunit Rpc11	0.8008631	4.87623718	0.00090729	0.00246373
SPRRNA.35	SPRRNA.35	5S rRNA	0.80037927	5.02057174	7.79E-05	0.000261244
SPNCRNA.1097	#N/A	#N/A	0.80032559	5.13747523	6.84E-05	0.000232139
SPBC4.07c	rpt2	19S proteasome base subcomplex ATPase subunit Rpt2	0.79978951	7.29605144	2.03E-12	1.95E-11
SPAC26A3.04	rpl2002	60S ribosomal protein L20 (predicted)	0.79976407	7.92955899	1.58E-20	3.18E-19
SPCC191.02c	acs1	acetyl-CoA ligase (predicted)	0.79873922	8.84445875	2.66E-16	3.59E-15
SPNCRNA.88	#N/A	#N/A	0.79840387	1.68294966	0.36288329	0.458403715
SPCC576.03c	tpx1	thioredoxin peroxidase Tpx1	0.79752652	8.38246137	6.62E-21	1.39E-19
SPAC11D3.05	mfs2	transmembrane transporter Mfs2 (predicted)	0.79747761	6.19690416	1.52E-08	9.01E-08
SPBC336.10c	tif512	translation elongation and termination factor eIF5A (predic	0.79637379	8.06417858	5.40E-20	1.03E-18
SPBTRNAASP.04	SPBTRNAASP.04	tRNA Aspartic acid	0.79635465	4.16613978	0.0037675	0.00880723
SPAC186.03	SPAC186.03	L-asparaginase (predicted)	0.79586779	6.02021394	1.08E-07	5.71E-07
SPAC19D5.06c	din1	RNA pyrophosphohydrolase Din1	0.7947088	5.42188261	0.00071626	0.002002125
SPAC3H5.10	rpl3202	60S ribosomal protein L32 (predicted)	0.79456441	7.37143924	2.99E-16	4.01E-15
SPAC23H3.05c	swd1	Set1C complex subunit Swd1	0.79272294	6.27801923	3.31E-07	1.64E-06
SPBC19C2.10	SPBC19C2.10	BAR adaptor protein, human endophilin-A2 ortholog, implic	0.79175926	6.37073676	3.08E-06	1.33E-05
SPAC1805.11c	rps2602	40S ribosomal protein S26 (predicted)	0.79174551	7.73617383	2.32E-19	4.19E-18
SPNCRNA.702	#N/A	#N/A	0.79120514	6.78585652	2.53E-10	1.90E-09
SPAC1687.03c	rfc4	DNA replication factor C complex subunit Rfc4	0.79079055	6.93046861	4.67E-12	4.28E-11
SPBTRNALEU.09	SPBTRNALEU.09	tRNA Leucine	0.78993163	1.36079823	0.57086208	0.659208633
SPNCRNA.699	#N/A	#N/A	0.78919947	3.77495165	0.1013338	0.15874229
SPNCRNA.842	#N/A	#N/A	0.78687924	5.33513089	6.61E-05	0.000224679
SPNCRNA.1595	#N/A	#N/A	0.78604363	4.72823179	0.00649855	0.014357989
SPBC3H7.09	erf2	palmitoyltransferase Erf2	0.78581416	5.13388756	0.00074801	0.002079076
SPAC9E9.03	leu2	3-isopropylmalate dehydratase Leu2	0.78509946	9.01967238	5.19E-21	1.12E-19
SPCC285.13c	nup60	nucleoporin Nup60	0.78501832	7.41190711	1.43E-15	1.81E-14
SPAC18B11.08c	SPAC18B11.08c	UPF0139 family conserved fungal ER membrane protein	0.78486901	4.6547228	0.00835297	0.017902988
SPBC800.05c	atb2	tubulin alpha 2	0.78462277	7.94324297	1.39E-17	2.10E-16
SPCC1223.08c	dfr1	dihydrofolate reductase/ serine hydrolase family fusion pro	0.78461075	8.5749766	4.17E-24	1.02E-22
SPBC115.03	SPBC115.03	gfo/idh/mocA family oxidoreductase (predicted)	0.78381598	6.43485147	4.60E-07	2.23E-06
SPCC1223.04c	set11	ribosomal protein lysine methyltransferase Set11	0.78350095	4.86611801	0.00095505	0.002575935
SPAC25G10.09c	pan1	actin cortical patch component, with EF hand and WH2 mot	0.78345651	8.8031304	1.39E-18	2.30E-17
SPAC328.05	hrb1	RNA-binding protein involved in export of mRNAs Hrb1 (pre	0.78243382	6.26748189	1.41E-08	8.41E-08
SPCC16C4.03	pin1	peptidyl-prolyl cis-trans isomerase Pin1	0.78206904	5.59088933	3.11E-05	0.000112158
SPAC869.02c	yhb1	nitric oxide dioxygenase Yhb1	0.78197512	8.89036824	7.63E-26	2.07E-24
SPCC24B10.05	tim9	Tim9-Tim10 complex subunit Tim9 (predicted)	0.7815195	6.24757116	2.93E-08	1.69E-07
SPBC1773.04	SPBC1773.04	methylglyoxyl reductase (NADPH-dependent) (predicted)	0.78138259	7.79116345	1.55E-18	2.57E-17
SPRRNA.34	SPRRNA.34	5S rRNA	0.78082813	4.13276617	0.00866071	0.018504287
SPBP4H10.16c	SPBP4H10.16c	G-patch RNA-binding protein, involved in splicing (predictec	0.78034052	5.42952507	4.09E-05	0.00014474
SPCC191.04c	SPCC191.04c	Schizosaccharomyces pombe specific protein	0.77985104	2.65902699	0.11216584	0.172846135
SPNCRNA.1419	#N/A	#N/A	0.77937774	3.7520296	0.02406469	0.046012979

SPBC3B9.01	fes1	Hsp70 nucleotide exchange factor Fes1 (predicted)	0.77871293	6.49379143	6.74E-07	3.19E-06
SPNCRNA.865	#N/A	#N/A	0.77802947	9.31289192	3.04E-20	5.99E-19
SPAC637.05c	vma2	V-type ATPase V1 subunit B	0.77695183	7.71349714	6.82E-18	1.05E-16
SPCC1450.06c	grx3	CIA machinery monothiol glutaredoxin Grx3	0.77641806	5.19757621	0.00036379	0.001082948
SPAC1805.16c	SPAC1805.16c	purine nucleoside phosphorylase (predicted)	0.77612218	6.11328635	4.87E-07	2.35E-06
SPBC365.04c	SPBC365.04c	RNA-binding protein, involved in ribosome biogenesis (pred	0.77577621	5.48797745	0.00049589	0.001439158
SPAC7D4.08	SPAC7D4.08	Schizosaccharomyces pombe specific protein	0.77566422	4.31992414	0.00351963	0.008307689
SPCC1393.03	rps1501	40S ribosomal protein S15 (predicted)	0.77463701	7.77326105	8.65E-15	1.01E-13
SPNCRNA.402	#N/A	#N/A	0.77366892	4.54841674	0.00308339	0.007390791
SPAC637.07	moe1	translation initiation factor eIF3d Moe1	0.77153028	8.48318747	1.12E-20	2.30E-19
SPNCRNA.1222	#N/A	#N/A	0.77128982	3.32978062	0.05546552	0.094758013
SPAC22A12.10	ept1	diacylglycerol cholinephosphotransferase/ diacylglycerol etl	0.77096156	8.26844735	6.18E-23	1.44E-21
SPBC1347.02	ani1	CENP-A amino terminus domain (NTD) isomerase Ani1	0.77006438	7.42320865	2.23E-12	2.12E-11
SPBC11G11.05	rpa34	DNA-directed RNA polymerase I complex subunit Rpa34 (pr	0.76871335	6.69233525	1.96E-09	1.31E-08
SPCC777.10c	ubc12	NEDD8-conjugating enzyme Ubc12	0.76750625	5.02479629	0.00054347	0.001557186
SPNCRNA.213	#N/A	#N/A	0.76596829	1.16850296	0.52950427	0.620933408
SPAC959.02	sec17	alpha SNAP (predicted)	0.76511677	7.1706058	4.41E-13	4.50E-12
SPNCRNA.1597	#N/A	#N/A	0.76429931	9.20370255	3.62E-25	9.48E-24
SPNCRNA.135	#N/A	#N/A	0.76394034	6.07319629	2.19E-05	8.15E-05
SPAC17G6.15c	fsf1	mitochondrial carrier, serine Fsf1 (predicted)	0.76370249	6.30065783	1.06E-08	6.42E-08
SPNCRNA.812	#N/A	#N/A	0.7635614	6.99278623	4.77E-10	3.45E-09
SPAC13A11.06	pdc202	pyruvate decarboxylase (predicted)	0.76239916	5.98025231	2.99E-06	1.30E-05
SPAC2F3.03c	rpa49	DNA-directed RNA polymerase I complex subunit Rpa49 (pr	0.75993037	6.96436943	5.17E-09	3.29E-08
SPAC1952.11c	ure2	nickel-dependent urease Ure2	0.75965039	8.25842148	6.87E-16	8.94E-15
SPNCRNA.1149	#N/A	#N/A	0.75781877	6.12232208	3.63E-06	1.55E-05
SPNCRNA.1041	#N/A	#N/A	0.75750578	8.25842672	9.19E-16	1.18E-14
SPNCRNA.625	#N/A	#N/A	0.75745043	6.88280319	1.31E-09	9.03E-09
SPNCRNA.741	#N/A	#N/A	0.75689494	7.39273389	1.56E-13	1.65E-12
SPAC5H10.01	dgc1	mitochondrial D-glutamate cyclase Dgc1 (predicted)	0.75683915	6.68431596	4.54E-10	3.31E-09
SPAPJ691.03	mic10	MICOS complex subunit Mic10 (predicted)	0.75646583	6.00371906	7.80E-07	3.64E-06
SPAC3G6.13c	rpl4101	60S ribosomal protein L41	0.75627331	6.75406805	3.43E-09	2.22E-08
SPAC24C9.03	mvd1	diphosphomevalonate decarboxylase mvd1 (predicted)	0.75611712	7.65716706	8.95E-17	1.25E-15
SPBC16E9.13	ksp1	serine/threonine protein kinase Ksp1 (predicted)	0.75535678	8.34127548	2.15E-15	2.67E-14
SPAC26F1.13c	lrs1	cytoplasmic leucine-tRNA ligase Lrs1 (predicted)	0.75486843	9.34621908	2.75E-23	6.55E-22
SPNCRNA.1332	#N/A	#N/A	0.75436086	6.77347935	1.89E-09	1.27E-08
SPCTRNAGLU.09	SPCTRNAGLU.09	tRNA Glutamic acid	0.75381673	4.16708616	0.00719134	0.015679928
SPAC6F12.07	tom20	mitochondrial TOM complex subunit Tom20 (predicted)	0.7535467	5.99912794	3.65E-06	1.56E-05
SPBC337.15c	coq7	ubiquinone biosynthesis protein Coq7	0.7528506	6.59299415	2.36E-08	1.37E-07
SPNCRNA.1292	#N/A	#N/A	0.7527413	6.19629081	9.90E-06	3.92E-05
SPAC16E8.14c	tae1	ribosomal protein AdoMet-dependent proline dimethyltrans	0.75204191	5.85671413	6.32E-06	2.60E-05
SPBC215.06c	SPBC215.06c	nucleolar RNA-binding protein, human LYAR homolog, impl	0.751802	5.09414498	0.00050749	0.001470815
SPAC2H10.01	SPAC2H10.01	transcription factor, zf-fungal binuclear cluster type (predic	0.75179046	5.63534978	0.00030642	0.000926328
SPCC1494.09c	tfb6	holo TFIIH complex subunit Tfb6	0.75030348	4.46947287	0.04304717	0.076265557
SPAC27E2.10c	rfc3	DNA replication factor C complex subunit Rfc3	0.750138	5.8627694	1.31E-05	5.06E-05
SPBC21B10.04c	nrf1	vacuolar transporter chaperone (VTC) complex, GTPase reg	0.74850182	5.38664995	0.00017165	0.000543045
SPCC1827.06c	SPCC1827.06c	aspartate semialdehyde dehydrogenase (predicted)	0.74771215	7.85415092	4.51E-16	5.95E-15
SPAC56E4.02c	alg13	N-acetylglucosaminyldiphosphodolichol N-acetylglucosamir	0.74771049	5.32668541	0.00120868	0.003181844

SPCC613.06	rpl902	60S ribosomal protein L9	0.74720633	8.08894128	1.94E-19	3.56E-18
SPBC800.06	brx1	ribosome biogenesis protein Brx1 (predicted)	0.74679347	6.7151024	4.21E-09	2.71E-08
SPBC14C8.06	arc1	ARP2/3 actin-organizing complex subunit Sop2	0.74628372	8.42327793	3.34E-21	7.31E-20
SPNCRNA.1122	#N/A	#N/A	0.74560128	3.26718324	0.07697284	0.125131432
SPCC297.05	SPCC297.05	DENN domain Rab GDP-GTP exchange factor, implicated in	0.74547342	7.00940578	2.53E-11	2.16E-10
SPAC1687.21	SPAC1687.21	fructose-2,6-bisphosphatase, human TIGAR ortholog (predi	0.74523097	7.12492536	6.82E-13	6.86E-12
SPBC947.04	pfl3	cell surface glycoprotein, flocculin Pfl3, DIPSY family	0.74517265	7.46420439	4.65E-15	5.58E-14
SPAC1687.05	pli1	SUMO E3 ligase Pli1	0.74489779	8.23790262	5.44E-19	9.40E-18
SPBC3H7.02	SPBC3H7.02	sulfate transmembrane transporter (predicted)	0.74390195	7.24674379	4.98E-11	4.03E-10
SPAC20G8.05c	cdc15	extended Fer/CIP4 (EFC) domain protein Cdc15	0.74373229	8.19811807	1.12E-11	9.90E-11
SPAC2C4.16c	rps801	40S ribosomal protein S8 (predicted)	0.74317571	8.06680865	1.11E-19	2.07E-18
SPNCRNA.366	#N/A	#N/A	0.74284091	0.94610028	0.69607586	0.768570505
SPAC8E11.02c	rad24	14-3-3 protein Rad24	0.7427109	8.84826081	9.38E-25	2.40E-23
SPNCRNA.612	#N/A	#N/A	0.74269392	6.70528727	5.26E-10	3.79E-09
SPBC582.09	pex11	peroxisomal biogenesis factor 11 (predicted)	0.74144849	6.26630135	3.31E-05	0.000118593
SPAC1783.08c	rpl1502	60S ribosomal protein L15b (predicted)	0.74134332	7.75033595	2.01E-16	2.73E-15
SPBC8D2.23	new15	mitochondrial protein, ribosomal subunit L35 (predicted)	0.74104987	3.6570729	0.06403986	0.107074015
SPCP1E11.10	SPCP1E11.10	ankyrin repeat protein, unknown biological role	0.74095118	5.62269421	1.06E-05	4.18E-05
SPAC2C4.04c	aim29	DUF2340, human C2orf76 ortholog, implicated in mitochon	0.74088918	5.6225415	4.20E-05	0.000147555
SPNCRNA.575	#N/A	#N/A	0.74067789	5.14312168	0.0001893	0.000595288
SPAC186.08c	SPAC186.08c	L-lactate dehydrogenase (predicted)	0.74067684	0.94641462	0.69617265	0.768570505
SPAC186.09	pdc102	pyruvate decarboxylase (predicted)	0.74067684	0.94641462	0.69617265	0.768570505
SPNCRNA.46	#N/A	#N/A	0.74067684	0.94641462	0.69617265	0.768570505
SPAC19D5.05c	imp3	U3 snoRNP-associated protein Imp3 (predicted)	0.74049735	5.12930861	0.0003304	0.000994426
SPCC584.15c	SPCC584.15c	arrestin involved in ubiquitin-dependent endocytosis	0.74020185	7.65113143	3.62E-13	3.74E-12
SPBC3B8.07c	dsd1	dihydroceramide delta-4 desaturase	0.7401865	7.86692578	4.45E-11	3.64E-10
SPBC19F8.08	rps401	40S ribosomal protein S4 (predicted)	0.73912187	8.4507124	1.16E-21	2.57E-20
SPNCRNA.117	#N/A	#N/A	0.73847833	0.94629987	0.69615475	0.768570505
SPNCRNA.1540	#N/A	#N/A	0.73847833	0.94629987	0.69615475	0.768570505
SPCC550.06c	hsp10	mitochondrial heat shock protein Hsp10 (predicted)	0.7379282	7.46427161	3.97E-14	4.39E-13
SPBC776.01	rpl29	60S ribosomal protein L29	0.73779658	6.59846461	3.23E-07	1.60E-06
SPBC557.02c	SPBC557.02c	DUF2458 conserved fungal protein	0.73717828	6.02120686	1.65E-06	7.39E-06
SPNCRNA.435	#N/A	#N/A	0.73637207	0.94685974	0.69634877	0.768570505
SPAC19A8.04	erg5	C-22 sterol desaturase Erg5	0.73611905	8.91260997	1.17E-14	1.35E-13
SPAC1805.12c	uep1	ribosomal-ubiquitin fusion protein Ubi2	0.73530083	7.68307483	3.48E-15	4.23E-14
SPBC530.13	lsc1	Lsk1 associated cyclin	0.73524875	5.09547381	0.00160552	0.004107351
SPAC22E12.04	ccs1	superoxide dismutase copper chaperone Ccs1	0.73512979	6.36367413	4.91E-07	2.37E-06
SPCC4G3.17	hdd1	HD domain protein phosphoric ester hydrolase family (pred	0.73395397	6.30237975	1.26E-06	5.76E-06
SPAC3H5.07	rpl702	60S ribosomal protein L7b involved in cytoplasmic translati	0.73307352	7.77505361	1.46E-15	1.85E-14
SPBC1709.02c	vrs1	cytoplasmic valine-tRNA ligase Vrs1/Vas1	0.73274992	9.17204822	2.69E-24	6.68E-23
SPAC3A12.10	rpl2001	60S ribosomal protein L20a (predicted)	0.73184274	8.04029443	1.76E-16	2.40E-15
SPCC5E4.07	rpl2802	60S ribosomal protein L27/L28	0.73123111	7.92877288	2.83E-16	3.82E-15
SPAC13C5.05c	SPAC13C5.05c	N-acetylglucosamine-phosphate mutase (predicted)	0.7311091	8.27543508	3.15E-13	3.28E-12
SPAC1687.02	rce1	CAAX prenyl protease (predicted)	0.730895	7.23889135	3.33E-13	3.45E-12
SPAC26F1.06	gpm1	monomeric 2,3-bisphosphoglycerate (BPG)-dependent phos	0.7298411	8.9280706	5.55E-23	1.30E-21
SPBC1815.01	eno101	enolase (predicted)	0.72982241	10.0091927	5.36E-27	1.59E-25
SPAC17A5.03	rpl301	60S ribosomal protein L3	0.72965634	9.31596575	5.39E-21	1.15E-19

SPAC890.07c	rmt1	type I protein arginine N-methyltransferase Rmt1	0.72962207	6.9417597	9.19E-10	6.44E-09
SPAPB1A10.15	arv1	Arv1-like family protein Arv1 (predicted)	0.72827388	4.8107808	0.00257436	0.006274532
SPAC4G9.04c	pcf11	mRNA cleavage and polyadenylation specificity factor (prec	0.72799921	8.22662045	3.65E-19	6.41E-18
SPAC3F10.03	grs1	mitochondrial and cytoplasmic glycine-tRNA ligase Grs1	0.72764253	8.74599028	3.46E-17	5.04E-16
SPAC144.11	rps1102	40S ribosomal protein S11 (predicted)	0.72751684	6.66574344	1.14E-07	5.99E-07
SPAC15E1.10	fub1	PI31 proteasome inhibitor Fub1 (predicted)	0.72721235	4.84919549	0.02077873	0.040492337
SPAP32A8.03c	bop1	ubiquitin-protein ligase E3, human RNF126 ortholog (predic	0.72626531	7.22484246	1.47E-12	1.44E-11
SPAC2E1P5.02c	mug109	Rab GTPase binding protein upregulated in meiosis II (pred	0.72589641	4.7611905	0.0013412	0.003490197
SPNCRNA.371	#N/A	#N/A	0.72516443	2.6340634	0.20099147	0.281661238
SPBC337.03	rhn1	RNA polymerase II transcription termination factor homolo	0.72486789	5.63842253	0.00027123	0.000828044
SPCC962.04	rps1201	40S ribosomal protein S12 (predicted)	0.72471625	7.5857953	8.47E-15	9.96E-14
SPNCRNA.1358	#N/A	#N/A	0.72460195	6.27463613	6.84E-08	3.71E-07
SPBP8B7.11	nxt3	ubiquitin protease cofactor Nxt3 (predicted)	0.72458459	7.47242743	7.90E-12	7.09E-11
SPCC1442.07c	wss2	ubiquitin/metalloprotease fusion protein Udp7	0.72380705	6.22600316	6.44E-07	3.06E-06
SPAC1002.17c	urg2	uracil phosphoribosyltransferase (predicted)	0.72375186	5.20678317	0.00060033	0.001701123
SPNCRNA.639	#N/A	#N/A	0.72371541	9.0769092	2.80E-22	6.39E-21
SPNCRNA.1639	#N/A	#N/A	0.72283153	5.93417079	8.60E-06	3.45E-05
SPBC119.10	asn1	asparagine synthetase	0.72244891	9.19131957	1.85E-23	4.44E-22
SPNCRNA.1658	#N/A	#N/A	0.72085698	8.89555193	3.62E-16	4.81E-15
SPCC285.15c	rps2802	40S ribosomal protein S28, Rps2802	0.72058693	6.87229395	6.68E-10	4.75E-09
SPCC1672.03c	gud1	guanine deaminase Gud1 (predicted)	0.72027855	7.22110331	4.31E-12	3.98E-11
SPBC1348.10c	SPBC1348.10c	phospholipase (predicted)	0.72025938	8.47882776	1.11E-14	1.29E-13
SPAC3H5.12c	rpl501	60S ribosomal protein L5 (predicted)	0.72006468	8.6389804	9.86E-21	2.05E-19
SPAC22E12.01	pet3	Golgi phosphoenolpyruvate transmembrane transporter Pet	0.71997165	6.10517172	4.16E-07	2.02E-06
SPAC11D3.02c	SPAC11D3.02c	ELLA family acetyltransferase (predicted)	0.71980989	6.59121615	6.01E-06	2.48E-05
SPBC30B4.04c	sol1	SWI/SNF complex subunit Sol1	0.71888577	6.81223924	1.57E-09	1.07E-08
SPNCRNA.1300	#N/A	#N/A	0.71851432	4.74568269	0.004658	0.010676219
SPNCRNA.287	#N/A	#N/A	0.71851432	4.74568269	0.004658	0.010676219
SPNCRNA.608	#N/A	#N/A	0.71786752	7.83344061	5.86E-15	6.99E-14
SPBC12D12.05c	SPBC12D12.05c	mitochondrial carrier, ATP:ADP antiporter (predicted)	0.71756607	6.63403538	1.72E-08	1.02E-07
SPAC186.01	pfl9	cell surface glycoprotein, flocculin Pfl9, DIPSY family	0.7174822	5.04822632	0.00065448	0.001844949
SPBC16G5.05c	scs2	VAP family protein Scs2	0.71672705	8.48154771	4.85E-18	7.67E-17
SPAC16C9.06c	upf1	ATP-dependent RNA helicase Upf1	0.71654899	6.88891738	1.89E-08	1.11E-07
SPCPB16A4.03c	ade10	bifunctional IMP cyclohydrolase/phosphoribosylaminoimida	0.71541443	8.59877235	1.17E-18	1.96E-17
SPBC106.03	SPBC106.03	mitochondrial Rossman fold DUF1776 family protein	0.71349719	6.83123886	1.69E-08	9.96E-08
SPBC1604.13c	mrpl32	mitochondrial ribosomal protein subunit L32 (predicted)	0.71335565	5.0425238	0.01430409	0.029117353
SPBTRNAGLU.08	SPBTRNAGLU.08	tRNA Glutamic acid	0.71285851	3.84121943	0.03078162	0.056982833
SPBC336.06c	rnh1	ribonuclease H Rnh1 (predicted)	0.71213843	5.96801995	1.97E-06	8.73E-06
SPAC3F10.06c	rit1	initiator methionine tRNA 2'-O-ribosyl phosphate transfera	0.71149233	6.20906428	3.66E-06	1.56E-05
SPBC16G5.15c	fkh2	forkhead transcription factor Fkh2	0.71132487	6.58222768	1.28E-07	6.67E-07
SPCC24B10.21	tpi1	triosephosphate isomerase	0.71094629	8.94110685	1.19E-19	2.21E-18
SPAC977.09c	SPAC977.09c	phospholipase (predicted)	0.71066215	8.50287612	3.67E-14	4.09E-13
SPCC1322.15	rpl3402	60S ribosomal protein L34	0.71064891	5.95282751	8.88E-05	0.000295211
SPBC887.10	mcs4	response regulator Mcs4	0.70982444	6.55029903	9.27E-06	3.70E-05
SPBC21B10.10	rps402	40S ribosomal protein S4 (predicted)	0.70964695	8.14575692	6.23E-19	1.06E-17
SPBC23G7.15c	rpp202	60S acidic ribosomal protein A4	0.70893587	7.33715241	1.36E-11	1.19E-10
SPBP23A10.16	tim18	TIM22 inner membrane protein import complex anchor sub	0.70892177	7.10234399	1.08E-11	9.53E-11

SPAC11G7.04	ubi1	ribosomal-ubiquitin fusion protein Ubi1 (predicted)	0.70816151	7.7027435	2.73E-15	3.34E-14
SPAC23C4.14	alg1	mannosyltransferase complex subunit Alg1 (predicted)	0.70787789	5.74537827	4.53E-05	0.00015872
SPRRNA.55	SPRRNA.55	5S rRNA (predicted)	0.70783813	4.16669772	0.01718541	0.034265084
SPBC405.01	ade1	phosphoribosylamine-glycine ligase/phosphoribosylformylg	0.70606356	8.54252755	3.40E-19	5.98E-18
SPAC1805.13	rpl14	60S ribosomal protein L14 (predicted)	0.70569178	7.69780152	4.46E-14	4.92E-13
SPCC777.09c	arg1	acetylornithine aminotransferase	0.70524745	8.60955225	8.89E-21	1.85E-19
SPBC11C11.08	srp1	SR family protein, human SRFS2 ortholog Srp1	0.70409229	7.68275648	6.65E-14	7.20E-13
SPAC644.05c	dut1	deoxyuridine 5'-triphosphate nucleotidohydrolase (predictec	0.70355908	5.67793498	1.73E-05	6.55E-05
SPBP8B7.10c	utp16	U3 snoRNP-associated protein Utp16 (predicted)	0.70292453	5.16940436	0.00040769	0.001201014
SPBC3D6.15	rps2501	40S ribosomal protein S25 (predicted)	0.70218974	6.5854301	6.66E-08	3.63E-07
SPCC417.08	tef3	translation elongation factor eEF3	0.702144	10.6431746	5.82E-27	1.71E-25
SPBP16F5.05c	yar1	ribosome biogenesis protein Yar1 (predicted)	0.7018383	5.42936119	7.57E-05	0.000254441
SPAC4G9.11c	cmb1	cytosine-mismatch binding protein 1	0.70180111	5.71188929	3.51E-05	0.000125571
SPAC20G4.06c	adf1	actin depolymerizing factor, cofilin	0.7016693	7.50740288	3.35E-12	3.13E-11
SPBC24C6.05	sec28	coatomer epsilon subunit (predicted)	0.70144338	7.02304783	6.62E-11	5.26E-10
SPBC19G7.03c	rps3002	40S ribosomal protein S30 (predicted)	0.70115957	6.50768453	1.46E-06	6.59E-06
SPNCRNA.1103	#N/A	#N/A	0.70114713	8.12552747	4.53E-18	7.18E-17
SPCC162.03	SPCC162.03	short chain dehydrogenase (predicted)	0.70112334	5.53043753	0.00010293	0.000338523
SPCC1795.04c	pre10	20S proteasome complex subunit alpha 7, Pre10	0.70060193	6.94907165	3.51E-10	2.59E-09
SPNCRNA.1434	#N/A	#N/A	0.70055395	3.98357442	0.0340075	0.0622198
SPCC645.06c	rgf3	RhoGEF Rgf3	0.70016683	8.19992236	2.16E-12	2.07E-11
SPAC5H10.06c	adh4	alcohol dehydrogenase Adh4	0.69998054	6.25265568	1.75E-05	6.64E-05
SPBC2G2.04c	mmf1	mitochondrial matrix protein, YjgF family protein Mmf1, re	0.69997437	7.43712119	5.07E-13	5.14E-12
SPCC126.12	SPCC126.12	GTP cyclohydrolase (predicted)	0.6996637	5.97216822	1.95E-06	8.66E-06
SPAC1805.06c	hem2	porphobilinogen synthase Hem2 (predicted)	0.69950373	7.21967497	5.76E-11	4.61E-10
SPAC12B10.02c	SPAC12B10.02c	endoplasmic reticulum resident protein required for packag	0.6991465	6.28171438	1.60E-07	8.20E-07
SPCC1739.05	set5	histone lysine methyltransferase Set5 (predicted)	0.69895533	5.59736902	2.72E-05	9.97E-05
SPBC17A3.04c	rar1	cytoplasmic methionine-tRNA ligase Mrs1 (predicted)	0.69833242	8.71303958	2.96E-19	5.24E-18
SPNCRNA.1143	#N/A	#N/A	0.69762277	7.23249948	2.89E-11	2.43E-10
SPBC2D10.12	rhp23	Rad23 homolog Rhp23	0.69628517	6.98771613	6.42E-08	3.50E-07
SPBC215.15	sec13	COPII-coated vesicle component Sec13	0.69596462	7.4179807	8.60E-12	7.68E-11
SPAC139.03	toe2	transcription factor, zf-fungal binuclear cluster type (predic	0.6957945	5.53009907	0.00026691	0.000816321
SPBC359.01	SPBC359.01	amino acid transmembrane transporter (predicted)	0.69556508	6.05886602	7.20E-06	2.93E-05
SPBC16E9.08	mcp4	prospore membrane protein Mcp4/Mug101	0.69468949	4.50073346	0.00994977	0.021007813
SPAC23A1.08c	rpl3401	60S ribosomal protein L34	0.69465934	7.36826528	6.01E-13	6.07E-12
SPBC14C8.03	fma2	methionine aminopeptidase Fma2 (predicted)	0.69417107	8.09243679	6.72E-17	9.53E-16
SPAC821.06	spn2	mitotic and meiotic (sporulation) septin Spn2	0.69232359	6.90638218	2.61E-09	1.71E-08
SPAPB24D3.06c	SPAPB24D3.06c	Alpha/Beta hydrolase fold, DUF1749 family protein	0.69198499	6.78642641	1.99E-08	1.17E-07
SPNCRNA.1136	#N/A	#N/A	0.69140118	5.53122273	0.00025589	0.000785788
SPAC56F8.08	mud1	UBA domain protein Mud1	0.69132951	7.20569853	1.60E-08	9.46E-08
SPAC26A3.03c	rmi1	RecQ mediated genome instability protein Rmi1 (predicted	0.69078073	8.41725543	2.28E-19	4.13E-18
SPBC244.02c	utp6	U3 snoRNP-associated protein Utp6 (predicted)	0.68978773	5.78406138	0.00013715	0.000442103
SPCC1840.03	sal3	karyopherin/importin beta family nuclear import signal rece	0.68926508	8.54209333	5.81E-19	9.99E-18
SPCC825.05c	pwi1	splicing coactivator Srm160 (predicted)	0.68857909	5.8908523	2.05E-05	7.66E-05
SPBC582.08	SPBC582.08	alanine aminotransferase (predicted)	0.68855841	7.48291778	2.18E-12	2.08E-11
SPAC1142.06	get3	GET complex (ER membrane insertion) subunit Get3 (predi	0.68835892	6.98812261	1.81E-08	1.07E-07
SPBC56F2.10c	alg5	dolichyl-phosphate beta-glucosyltransferase Alg5	0.68820017	5.3815166	0.00156386	0.004012847

SPBC1677.03c	tda1	threonine ammonia-lyase Tda1	0.68663475	8.47561558	9.02E-15	1.05E-13
SPAC22F3.07c	atp20	F1-FO ATP synthase subunit G (predicted)	0.68634507	6.10159547	0.00016391	0.000521216
SPAC3G9.03	rpl2301	60S ribosomal protein L23	0.68594656	7.42586413	2.07E-12	1.98E-11
SPNCRNA.1104	#N/A	#N/A	0.6857641	6.49024025	3.34E-07	1.65E-06
SPNCRNA.415	#N/A	#N/A	0.68539351	2.68367571	0.25450726	0.344798307
SPAC26A3.11	SPAC26A3.11	mitochondrial omega-amidase (predicted)	0.68498059	7.2111288	5.92E-12	5.38E-11
SPAC926.04c	hsp90	Hsp90 chaperone	0.68496132	9.99219383	3.42E-20	6.70E-19
SPCC550.04c	gpi2	pig-C (predicted)	0.68492097	4.89169747	0.00135317	0.003515957
SPAC19D5.10c	#N/A	#N/A	0.68344097	1.63362452	0.49051885	0.585199995
SPNCRNA.1233	#N/A	#N/A	0.68325102	7.07613085	9.39E-08	4.99E-07
SPNCRNA.553	#N/A	#N/A	0.68239559	6.10197397	1.23E-05	4.80E-05
SPBC365.16	SPBC365.16	mitochondrial membrane protein, conserved in yeast and a	0.68214741	7.62812844	2.74E-07	1.37E-06
SPAC13G6.07c	rps601	40S ribosomal protein S6	0.68166115	8.39516448	4.52E-19	7.87E-18
SPCC63.12c	pup3	20S proteasome complex subunit beta 3, Pup3	0.68155292	7.1742286	7.95E-11	6.24E-10
SPAC1565.02c	SPAC1565.02c	Rho-type GTPase activating protein, with CRAL-TRIO_doma	0.6809544	6.88900848	3.67E-09	2.37E-08
SPAC139.01c	ath2	Ath1 complex protein Ath2 nuclease, XP-G family	0.68051538	7.45909172	4.78E-09	3.05E-08
SPBC83.18c	fic1	C2 domain protein Fic1	0.68019623	6.07352215	2.84E-05	0.000103336
SPNCRNA.1063	#N/A	#N/A	0.68007458	4.09556005	0.02124056	0.041274135
SPBC36B7.04	dus1	tRNA dihydrouridine synthase Dus1 (predicted)	0.67973548	6.14862878	4.55E-06	1.91E-05
SPAPB1A10.05	SPAPB1A10.05	Schizosaccharomyces specific protein	0.67930169	5.03553248	0.00675225	0.014829784
SPNCRNA.927	#N/A	#N/A	0.67922289	6.8355201	8.69E-08	4.64E-07
SPAC12G12.04	mcp60	mitochondrial heat shock protein Hsp60/Mcp60	0.67909253	9.1768145	5.36E-21	1.15E-19
SPAC1F3.07c	rsc58	RSC complex subunit Rsc58	0.67901503	6.03144635	8.47E-06	3.40E-05
SPAC6F6.07c	rps13	40S ribosomal protein S13	0.67888522	8.11664451	3.11E-15	3.80E-14
SPAC20G4.07c	erg4	C-24(28) sterol reductase Erg4	0.67832852	6.91621591	5.03E-08	2.79E-07
SPBC17G9.12c	SPBC17G9.12c	HAD superfamily hydrolase, unknown role (predicted)	0.67829762	5.44794686	0.00050863	0.001472852
SPBC27.08c	sua1	sulfate adenylyltransferase	0.67815509	8.49284146	4.38E-14	4.84E-13
SPNCRNA.860	#N/A	#N/A	0.67787809	5.71685825	0.00054692	0.001565742
SPNCRNA.78	#N/A	#N/A	0.67740318	3.31407689	0.09325179	0.148023886
SPAC22E12.13c	rlp24	ribosomal L24-like protein involved in ribosome biogenesis	0.67698777	6.52241708	9.74E-06	3.86E-05
SPAC343.05	vma1	V-type ATPase V1 domain, subunit A	0.67649438	8.63493087	5.55E-19	9.56E-18
SPAC12G12.07c	SPAC12G12.07c	conserved fungal protein	0.67633496	8.01990399	6.25E-12	5.66E-11
SPBC19C7.05	SPBC19C7.05	vesicle-mediated transport protein (predicted)	0.67611698	6.80664139	0.00051916	0.001496359
SPBC56F2.09c	arg5	arginine specific carbamoyl-phosphate synthase subunit Ar	0.67583201	8.08643489	8.96E-14	9.60E-13
SPAC1399.02	SPAC1399.02	transmembrane transporter (predicted)	0.67543259	6.46397685	4.23E-07	2.06E-06
SPNCRNA.697	#N/A	#N/A	0.67527917	6.61419339	2.00E-07	1.01E-06
SPCC1795.02c	vcx1	vacuolar proton/calcium exchanger (predicted)	0.67498549	7.38373779	2.28E-10	1.72E-09
SPCC16C4.17	mug123	Schizosaccharomyces specific protein Mug123	0.67490766	5.13987445	0.00099802	0.002675943
SPAC17G6.14c	uap56	TREX complex subunit, ATP-dependent RNA helicase Uap56	0.67318531	8.29779229	2.51E-17	3.71E-16
SPBC16E9.14c	zrg17	Golgi cation diffusion family zinc transmembrane transport	0.67314592	5.34646965	0.00111636	0.002962888
SPBC17D1.01	spp41	transcriptional regulatory protein Spp41 (predicted)	0.67299691	8.08778555	5.82E-13	5.88E-12
SPAC589.10c	ubi5	ribosomal-ubiquitin fusion protein Ubi5 (predicted)	0.67273366	7.87273483	1.74E-14	1.98E-13
SPAC1687.19c	qtr1	queuine tRNA-ribosyltransferase Qtrt1	0.67235654	6.60776721	2.03E-07	1.03E-06
SPBC17G9.03c	krs1	cytoplasmic lysine-tRNA ligase Krs1 (predicted)	0.67146165	8.74937329	3.89E-18	6.19E-17
SPCC1450.15	SPCC1450.15	pig-F/3-ketosphinganine reductase fusion protein (predicte	0.6700475	7.38357326	3.16E-11	2.63E-10
SPBC21C3.08c	car2	ornithine transaminase Car2	0.66982202	9.06911882	2.34E-19	4.21E-18
SPRRNA.04	SPRRNA.04	5S rRNA	0.66950895	5.57384167	0.00012585	0.000407994

SPNCRNA.1393	#N/A	#N/A	0.66945159	6.42455924	3.57E-06	1.53E-05
SPBC119.01	rpn3	19S proteasome regulatory subunit Rpn3	0.66942568	7.93295533	5.87E-11	4.69E-10
SPBC83.02c	rpl4302	60S ribosomal protein L37a (predicted)	0.66695874	6.71977916	1.11E-07	5.83E-07
SPBC1539.03c	arg41	argininosuccinate lyase (predicted)	0.66677288	8.01115561	5.26E-11	4.24E-10
SPAC7D4.10	vma13	V-type ATPase V1 subunit H (predicted)	0.6666962	7.0046506	8.76E-09	5.40E-08
SPAC1786.04	SPAC1786.04	Schizosaccharomyces pombe specific protein	0.66610874	2.09754316	0.39143273	0.4868762
SPAC1610.02c	mrpl1	mitochondrial ribosomal protein subunit L1 (predicted)	0.66599222	6.14151277	2.58E-05	9.49E-05
SPBC4C3.07	eif6	translation initiation factor eIF3f	0.66593709	7.68673399	1.58E-11	1.37E-10
SPAC1039.06	SPAC1039.06	D-serine ammonia-lyase activity (predicted)	0.66573502	7.85670862	1.28E-11	1.12E-10
SPAC323.07c	SPAC323.07c	MatE family transmembrane transporter (predicted)	0.66539842	7.29763462	2.20E-09	1.45E-08
SPNCRNA.1390	#N/A	#N/A	0.66302078	5.08212507	0.00159516	0.004083932
SPCC1223.09	uro1	uricase Uro1	0.66226308	6.62617632	2.25E-05	8.35E-05
SPAC1F3.05	gga21	Golgi localized Arf binding gamma-adaptin ortholog Gga21	0.66089351	7.38667236	1.13E-10	8.77E-10
SPAC1565.01	rcf2	cytochrome c oxidase assembly protein Rcf2	0.65940183	8.71775097	1.04E-15	1.34E-14
SPAC21E11.03c	pcr1	transcription factor Pcr1	0.65935445	5.74898302	0.00137714	0.003572783
SPBC342.03	gas4	spore wall 1,3-beta-glucanosyltransferase Gas4	0.65882668	7.39174455	2.11E-11	1.82E-10
SPRRNA.05	SPRRNA.05	5S rRNA	0.65874738	5.38413008	0.00034817	0.001042375
SPBC12C2.04	SPBC12C2.04	NAD binding dehydrogenase family protein	0.65874211	6.76257735	5.78E-06	2.40E-05
SPBC16E9.06c	uvi31	mitochondrial [4Fe-4S] cluster transfer protein Uvi31 (pred	0.65857113	8.69031818	4.28E-16	5.66E-15
SPRRNA.20	SPRRNA.20	5S rRNA	0.65782602	5.62756201	0.00010886	0.000356653
SPAC2F3.02	SPAC2F3.02	ER protein translocation subcomplex subunit (predicted)	0.65776208	7.53288279	1.23E-09	8.48E-09
SPBC17D1.04	acr1	RNA polymerase I upstream activation factor complex subu	0.65766877	6.27673481	0.00010364	0.00034054
SPCC330.12c	sdh3	succinate dehydrogenase (ubiquinone) cytochrome b subuni	0.65706689	7.09440398	1.88E-07	9.53E-07
SPCC132.01c	mtr1	microtubule regulator Mtr1	0.65660944	7.39663376	2.54E-08	1.47E-07
SPBC428.14	SPBC428.14	1-acylglycerol-3-phosphate acyltransferase (predicted)	0.6558326	7.28589188	4.87E-11	3.96E-10
SPAC3G6.11	chl1	ATP-dependent DNA helicase Chl1 (predicted)	0.65550306	7.44531384	8.09E-12	7.25E-11
SPNCRNA.1278	#N/A	#N/A	0.65503359	4.71649958	0.00893414	0.019022778
SPCC1259.01c	rps1802	40S ribosomal protein S18 (predicted)	0.65398179	7.64086559	1.00E-12	9.95E-12
SPCC584.12	mug42	Schizosaccharomyces pombe specific protein	0.65309974	2.65786133	0.33705751	0.431628967
SPAC22A12.09c	sap114	U2 snRNP subunit Sap114	0.6525793	5.80788746	7.57E-05	0.000254441
SPBP4H10.20	nhm1	m7G(5')pppN diphosphatase Nhm1	0.65148958	5.30880435	0.0005791	0.001648169
SPAC1783.04c	hst4	Sirtuin family histone deacetylase Hst4	0.65142064	7.27450458	1.22E-10	9.39E-10
SPCC18.17c	SPCC18.17c	proteasome assembly chaperone (predicted)	0.65111807	6.72061041	2.80E-06	1.22E-05
SPNCRNA.824	#N/A	#N/A	0.65082956	6.86182973	1.51E-06	6.82E-06
SPCC18.02	SPCC18.02	transmembrane transporter (predicted)	0.64995373	6.91576299	2.55E-09	1.67E-08
SPRRNA.11	SPRRNA.11	5S rRNA	0.64942188	5.27293986	0.0005154	0.001488675
SPBC25B2.07c	mmb1	mitochondrial microtubule binder Mmb1	0.6487412	6.81019969	3.88E-06	1.65E-05
SPAC22G7.01c	fra1	iron responsive transcriptional regulator, peptidase family (	0.64851388	8.33226524	4.36E-12	4.01E-11
SPCC74.01	sly1	SNARE binding protein Sly1 (predicted)	0.64829934	7.47175355	5.09E-08	2.82E-07
SPAC18G6.15	mal3	microtubule plus-end binding protein, EB1 family Mal3	0.6478668	6.78425881	1.59E-06	7.14E-06
SPRRNA.38	SPRRNA.38	5S rRNA	0.64765284	5.66413587	0.00011319	0.000369765
SPAC1556.07	pmm1	phosphomannomutase Pmm1	0.64736492	8.68989617	8.29E-16	1.07E-14
SPCC757.12	SPCC757.12	alpha-amylase homolog (predicted)	0.64703133	8.12421559	1.71E-12	1.67E-11
SPBC1709.05	sks2	heat shock protein, ribosome associated molecular chapero	0.64699947	10.0153493	4.78E-20	9.16E-19
SPAC4G9.10	arg3	ornithine carbamoyltransferase Arg3	0.64691997	7.11376779	1.89E-09	1.27E-08
SPAC8C9.08	rps5	40S ribosomal protein S5 (predicted)	0.64661807	8.31325781	1.58E-16	2.16E-15
SPAC607.03c	snu13	U3 snoRNP-associated protein Snu13	0.64647478	6.9461868	3.85E-08	2.18E-07

SPBC215.05	gpd1	glycerol-3-phosphate dehydrogenase Gpd1	0.6461687	9.08241085	4.86E-16	6.40E-15
SPCC126.11c	SPCC126.11c	RNA-binding protein, rrm type	0.64607339	5.98588907	7.27E-05	0.000245116
SPAC6B12.05c	ies2	Ino80 complex subunit Ies2	0.64543436	6.74686784	2.56E-06	1.12E-05
SPBC8D2.06	irs1	cytoplasmic isoleucine-tRNA ligase Irs1 (predicted)	0.64520569	8.83473327	1.51E-15	1.91E-14
SPBC36.04	cys11	cysteine synthase	0.64448463	7.5687692	1.58E-11	1.36E-10
SPNCRNA.952	#N/A	#N/A	0.64432991	5.73463653	0.00059342	0.001683297
SPAC24C9.05c	mug70	CBS and PB1 domain protein, conserved in fungi and plants,	0.64382083	7.52285247	5.15E-10	3.72E-09
SPNCRNA.1694	#N/A	#N/A	0.6437641	3.93362116	0.12898928	0.194800716
SPCC1739.11c	cdc11	SIN component scaffold protein, centriolin ortholog Cdc11	0.64269912	7.0819412	6.37E-10	4.54E-09
SPBP8B7.15c	mpe1	mRNA cleavage ubiquitin-protein ligase E3 Mpe1 (predicte	0.64250196	7.96647165	4.33E-13	4.44E-12
SPNCRNA.1127	#N/A	#N/A	0.64244853	4.77413911	0.00655877	0.01448165
SPCC1450.04	tef5	translation elongation factor EF-1 beta subunit, guanyl-nucl	0.64217584	8.29281059	5.09E-14	5.57E-13
SPBC2G2.11	myr1	N-myristoyltransferase Myr1 (predicted)	0.64217357	7.17617144	1.51E-09	1.03E-08
SPAC12G12.06c	rcl1	rRNA processing protein Rcl1 (predicted)	0.64199372	6.75324162	4.82E-08	2.69E-07
SPAC3C7.14c	obr1	NAD(P)H dehydrogenase (quinone) (predicted)	0.64140119	8.20374339	3.32E-15	4.04E-14
SPAC1783.03	fta2	CENP-P ortholog Fta2	0.64119572	7.44266711	3.51E-11	2.91E-10
SPAC17C9.10	stm1	vacuolar amino acid transmembrane transporter Stm1 (pre	0.64099446	6.96306509	5.51E-07	2.64E-06
SPCC1183.02	SPCC1183.02	glutathione S-transferase, translational elongation factor e	0.64088506	7.12357758	3.58E-10	2.63E-09
SPAC6G9.08	ubp6	ubiquitin C-terminal hydrolase Ubp6	0.64028312	8.39325772	6.44E-15	7.66E-14
SPAC10F6.05c	ubc6	ubiquitin conjugating enzyme E2 Ubc6 (predicted)	0.64024116	5.23326062	0.00141627	0.003664528
SPAC3H5.05c	rps1401	40S ribosomal protein S14 (predicted)	0.63896747	6.87375805	1.03E-07	5.42E-07
SPAC22E12.07	rna1	RanGAP GTPase activating protein Rna1	0.63729963	7.29055568	1.95E-09	1.30E-08
SPAC19A8.15	trp2	tryptophan synthase (predicted)	0.63694431	8.78803573	1.32E-16	1.83E-15
SPAC15A10.08	ain1	alpha-actinin	0.63693492	6.94640109	2.26E-07	1.14E-06
SPCC16C4.07	scw1	RNA-binding protein Scw1	0.6365362	7.34755919	5.59E-11	4.50E-10
ScpofMt27	#N/A	#N/A	0.6345515	4.14911392	0.06116705	0.102801135
SPBC119.18	mdm35	mitochondrial phosphatidylserine translocation complex sul	0.63444329	5.08131451	0.0043046	0.009933126
SPCC16C4.13c	rpl1201	60S ribosomal protein L12.1/L12A	0.63360381	8.05357968	1.99E-13	2.10E-12
SPAC15F9.01c	glm1	Glomulin, ubiquitin-protein transferase inhibitor Glm1 (pre	0.63357328	1.47717345	0.81205525	0.863014401
SPBC776.02c	dis2	serine/threonine protein phosphatase PP1, Dis2	0.63331555	7.49485448	2.82E-10	2.12E-09
SPCC4G3.05c	mus81	Holliday junction resolvase subunit Mus81	0.63258273	6.05013278	0.00017367	0.00054865
SPAC1F7.05	cdc22	ribonucleoside reductase large subunit Cdc22	0.63242833	9.10047324	7.43E-10	5.25E-09
SPAC6F6.10c	arc2	ARP2/3 actin-organizing complex subunit Arc34	0.63211057	7.4983113	1.80E-10	1.37E-09
SPAC3C7.09	set8	lysine methyltransferase Set8, unknown specificity (predict	0.62987408	5.00627294	0.02110697	0.041037986
SPBC30B4.05	kap109	karyopherin/importin beta family nuclear export signal rece	0.62890539	7.77885499	4.67E-11	3.81E-10
SPCC1281.03c	emc4	ER membrane protein complex subunit Emc4 (predicted)	0.62816871	5.90403423	0.00076442	0.002117164
SPAC14C4.04	SPAC14C4.04	ThiJ domain protein (predicted)	0.62781916	6.52499102	5.61E-07	2.68E-06
SPAC10F6.01c	sir1	sulfite reductase beta subunit Sir1	0.62716352	9.970315	6.48E-21	1.37E-19
SPBC354.10	def1	RNAPII degradation factor Def1 (predicted)	0.62709938	8.88770485	2.38E-14	2.67E-13
SPCC31H12.03c	mlo1	RNA binding protein (predicted)	0.62702625	6.52458281	1.53E-06	6.89E-06
SPCC18B5.07c	nup61	nucleoporin Nup61	0.62643316	7.3712994	4.71E-10	3.42E-09
SPBC21D10.07	cmc1	copper-binding protein of the mitochondrial intermembrane	0.62624941	5.83977548	0.00012131	0.00039479
SPAC18G6.07c	mra1	rRNA (pseudouridine) methyltransferase Mra1	0.62597059	6.90080596	2.79E-07	1.39E-06
SPBC11C11.09c	rpl502	60S ribosomal protein L5	0.62562373	8.48430629	2.01E-15	2.51E-14
SPAC3G6.05	SPAC3G6.05	mitochondrial Mpv17/PMP22 family protein 1 (predicted)	0.62530996	5.83441289	0.00117821	0.003110065
SPNCRNA.444	#N/A	#N/A	0.62513579	3.92349539	0.14913915	0.220054622
SPAC16.02c	srp2	splicing factor Srp2	0.62389504	7.56692023	6.93E-09	4.33E-08

SPAC19G12.14	its3	1-phosphatidylinositol-4-phosphate 5-kinase Its3	0.62371583	7.51753399	1.97E-08	1.16E-07
SPCPB16A4.05c	ure7	urease accessory protein UreG	0.62362069	7.47717554	3.26E-09	2.12E-08
SPBC1271.03c	SPBC1271.03c	NLI interacting factor family phosphatase (predicted)	0.62202824	6.79429622	1.24E-07	6.45E-07
SPAPB2B4.05	vma5	V-type ATPase V1 subunit C (predicted)	0.62142672	6.43797293	5.12E-06	2.14E-05
SPBC19G7.17	sec6102	translocon subunit Sec61 homolog (predicted)	0.62104461	7.40290063	2.57E-07	1.29E-06
SPBC1A4.08c	cct3	chaperonin-containing T-complex gamma subunit Cct3	0.62031586	8.41253808	2.37E-15	2.93E-14
SPCC1223.13	cbf12	CBF1/Su(H)/LAG-1 family transcription factor Cbf12	0.62016134	7.15427563	1.21E-05	4.73E-05
SPBC839.07	ibp1	Cdc25 family phosphatase Ibp1, unknown role, implicated in	0.61931348	5.03096003	0.00291206	0.007012224
SPNCRNA.986	#N/A	#N/A	0.61863867	7.57014706	8.95E-09	5.50E-08
SPAC688.14	set13	ribosome L32 lysine methyltransferase Set13	0.61822061	5.9219447	0.00106977	0.00285145
SPNCRNA.662	#N/A	#N/A	0.61813054	6.33860825	4.16E-05	0.000146569
SPCC24B10.15	swt1	RNA endoribonuclease involved in mRNP quality control Sw	0.61811753	6.44237743	1.92E-05	7.22E-05
SPAC19G12.17	erh1	enhancer of rudimentary homolog Erh1	0.61801416	5.19445816	0.0020026	0.005023098
SPCC584.04	sup35	cytoplasmic translation release factor class II eRF3	0.61681832	8.65037711	7.23E-15	8.58E-14
SPBC16D10.08c	hsp104	heat shock protein Hsp104	0.61655911	8.76095075	9.89E-06	3.92E-05
SPAC6F12.13c	fps1	geranyltranstransferase Fps1	0.61593276	7.78329386	1.28E-09	8.83E-09
SPCC1183.03c	fxn1	mitochondrial [2Fe-2S] cluster assembly frataxin Fxn1	0.61570563	7.44073907	7.95E-11	6.24E-10
SPBPB21E7.09	SPBPB21E7.09	L-asparaginase (predicted)	0.61533593	7.10228701	3.22E-09	2.09E-08
SPNCRNA.360	#N/A	#N/A	0.6146096	2.88344725	0.21265865	0.296120745
SPBC2G2.10c	mug110	Schizosaccharomyces specific protein Mug110	0.61380721	4.1334136	0.09767	0.153710541
SPCC1620.06c	SPCC1620.06c	ribose-phosphate pyrophosphokinase (predicted)	0.61317051	7.93026076	9.14E-13	9.08E-12
SPNCRNA.1072	#N/A	#N/A	0.6125961	8.4684045	9.72E-15	1.13E-13
SPCC550.05	nse1	Smc5-6 complex ubiquitin-protein ligase E3 subunit Nse1	0.61221602	7.76974639	2.12E-12	2.03E-11
SPNCRNA.1000	#N/A	#N/A	0.61218392	2.5034993	0.34237811	0.43704427
SPCP31B10.07	eft202	translation elongation factor 2 (EF-2) Eft2,B	0.61217398	10.327812	1.63E-20	3.26E-19
SPAC10F6.13c	caa1	cytoplasmic aspartate aminotransferase Caa1 (predicted)	0.61061017	8.33030525	3.38E-11	2.81E-10
SPNCRNA.255	#N/A	#N/A	0.61013853	1.29992961	0.56601767	0.655237593
SPBC1685.07c	avt5	vacuolar amino acid transmembrane transporter Avt5	0.60946869	6.34304258	1.54E-05	5.91E-05
SPAPB8E5.09	rvb1	ASTRA/Swr1/Ino80 complex AAA family ATPase Rvb1	0.60922007	7.85842295	4.43E-11	3.64E-10
SPAC4F8.02c	mrpl40	mitochondrial ribosomal protein subunit L40 (predicted)	0.60828238	5.91770533	0.00040216	0.001186786
SPBC1289.12	usp109	U1 snRNP-associated protein Usp109	0.60793689	6.10399366	0.00099297	0.002664485
SPCC61.01c	str2	plasma membrane siderophore-iron transmembrane transp	0.60745634	9.09634481	1.72E-17	2.56E-16
SPCC622.19	jmj4	peptidyl-lysine 3-dioxygenase activity jmj4 (predicted)	0.60733373	5.99181231	6.96E-05	0.000235745
SPCC777.04	SPCC777.04	amino acid transmembrane transporter (predicted)	0.6073156	5.08652753	0.00549596	0.012351382
SPNCRNA.855	#N/A	#N/A	0.6068697	1.29973607	0.56478636	0.654079503
SPBC839.19	new20	UPF0428 family, human CXorf56 and C15orf40 ortholog	0.60561304	4.57512968	0.01476433	0.029919966
SPAC12G12.17	SPAC12G12.17	non-classical export protein 1 (predicted)	0.60525063	4.68219326	0.01728256	0.034438594
SPAPYUG7.04c	rpb9	DNA-directed RNA polymerase II complex subunit Rpb9	0.60502552	5.90303565	0.00041314	0.001214958
SPRRNA.19	SPRRNA.19	5S rRNA	0.60487183	5.64665148	0.00028523	0.000866911
SPAPB8E5.06c	rpl302	60S ribosomal protein L3	0.60453781	9.21136249	1.53E-17	2.28E-16
SPBC649.02	rps1902	40S ribosomal protein S19 (predicted)	0.60424368	7.16988968	1.84E-08	1.09E-07
SPNCRNA.899	#N/A	#N/A	0.60386097	3.77415434	0.07326888	0.120114372
SPRRNA.26	SPRRNA.26	5S rRNA	0.60382892	2.16820878	0.4326589	0.528305004
SPBPB8B6.03	fah1	fatty acid amide hydrolase Fah1 (predicted)	0.60381933	4.98172092	0.01888386	0.037356007
SPAC1071.12c	stp1	protein tyrosine phosphatase Stp1, unknown biological role	0.60366714	5.54006151	0.00132969	0.003465544
SPAC343.21	#N/A	#N/A	0.60364361	3.40635192	0.18012898	0.257516332
SPBC1711.07	rrb1	WD repeat protein Rrb1 (predicted)	0.6036233	7.12684643	1.10E-07	5.77E-07

SPBC2F12.13	klp5	kinesin-8 family plus-end directed microtubule motor Klp5	0.60340105	7.47982973	1.97E-06	8.73E-06
SPBC1861.06c	mug131	UPF0300 family protein 4	0.60247474	6.26693039	1.47E-05	5.66E-05
SPNCRNA.1353	#N/A	#N/A	0.60208726	5.59267675	0.00038877	0.001150765
SPAC1687.01	rpc19	DNA-directed RNA polymerase I and III subunit Rpc19	0.60207297	5.5562642	0.00084776	0.002316393
SPNCRNA.890	#N/A	#N/A	0.60198204	5.93212951	0.00160329	0.004103182
SPBC1604.05	pgi1	glucose-6-phosphate isomerase (predicted)	0.60179892	9.64666034	6.21E-18	9.70E-17
SPBC839.16	thf1	C1-5,6,7,8-tetrahydrofolate (THF) synthase, trifunctional en	0.60148573	9.01813422	1.23E-14	1.42E-13
SPBC3H7.07c	ser2	phosphoserine phosphatase Ser2 (predicted)	0.60147895	7.44411122	3.00E-09	1.95E-08
SPBC31F10.12	tma20	RNA-binding protein Tma20 (predicted)	0.60114963	6.15310418	7.29E-05	0.000245821
SPBPB2B2.08	SPBPB2B2.08	conserved fungal protein	0.60093357	1.30003177	0.56928462	0.65816885
SPAC13G7.03	upf3	up-frameshift suppressor 3 family protein (predicted)	0.60093253	4.44547054	0.03545105	0.064400529
SPNCRNA.790	#N/A	#N/A	0.60076403	5.28318359	0.00346175	0.008196668
SPCC1450.03	utp502	ribonucleoprotein (RNP) complex Utp502 (predicted)	0.60069225	5.12141497	0.00666653	0.014674599
SPBC1711.18	tam9	mitochondrial ribosomal protein subunit L36, MrpL36/YmL3	0.60044856	5.63310047	0.00057652	0.001642194
SPAC20H4.08	whi2	G-patch RNA-binding protein, involved in splicing (predictec	0.59989775	5.22009257	0.00159246	0.004078535
SPAPB15E9.01c	pfl2	cell surface glycoprotein, flocculin Pfl2	0.59965759	10.3995716	5.63E-09	3.56E-08
SPAP27G11.12	SPAP27G11.12	human HID1 ortholog 1, possible Golgi protein (by similarity	0.59961188	7.33259196	1.53E-07	7.86E-07
SPBC14F5.09c	ade8	adenylosuccinate lyase Ade8	0.59926299	7.95771795	1.49E-10	1.14E-09
SPAC13A11.02c	erg11	sterol 14-demethylase Erg11	0.59875778	8.80791524	3.03E-10	2.26E-09
SPBC582.07c	rpn7	19S proteasome regulatory subunit Rpn7	0.59776127	7.79180052	2.94E-10	2.20E-09
SPAC12B10.11	exg2	cell wall glucan glucosidase Exg2	0.59590229	5.63164814	0.00416438	0.009647638
SPNCRNA.1607	#N/A	#N/A	0.59562816	5.41165954	0.00381583	0.008907959
SPBC8D2.20c	sec31	COPII-coated vesicle component Sec31 (predicted)	0.5951813	9.57780322	2.55E-17	3.75E-16
SPBC83.15	wdr74	ribosome assembly factor, WD repeat protein Nsa1/Wdr74	0.595173	6.00008523	0.0009514	0.002568889
SPNCRNA.1500	#N/A	#N/A	0.59468692	7.07825219	5.32E-08	2.94E-07
SPAC3H1.06c	SPAC3H1.06c	transmembrane transporter (predicted)	0.59415069	8.3060922	2.60E-11	2.20E-10
SPAC4H3.09	SPAC4H3.09	mitochondrial [2Fe-2S] cluster assembly and type II fatty ac	0.59358515	5.52546655	0.00381819	0.008910412
SPBC21H7.07c	his5	imidazoleglycerol-phosphate dehydratase His5	0.5935848	7.07145519	4.72E-08	2.64E-07
SPBC83.07	jmj3	Lid2 complex subunit, histone demethylase H3-K36 specific	0.59316897	6.40271081	1.68E-05	6.40E-05
SPAC1F7.10	SPAC1F7.10	hydantoin racemase family (predicted)	0.59266191	5.53138611	0.00775571	0.016754803
SPAC19B12.06c	rbd4	rhomboid family protease, unknown biological role, associa	0.59212981	7.04773899	1.39E-05	5.36E-05
SPBC1685.06	cid11	poly(A) polymerase Cid11, terminal uridylyl transferase (pre	0.59181512	5.35493723	0.00461851	0.010603554
SPCC18.05c	rsa4	notchless-like ribosome biogenesis protein Rsa4 (predicted)	0.58989776	6.42886498	2.51E-05	9.27E-05
SPBC11B10.02c	his3	histidinol-phosphate aminotransferase imidazole acetol ph	0.58988533	7.76549041	3.45E-09	2.23E-08
SPBC13G1.01c	nam9	mitochondrial ribosomal protein subunit S4 (predicted)	0.58973135	6.20375145	8.47E-05	0.000282285
SPBC4F6.04	rpl2502	60S ribosomal protein L25 (predicted)	0.58904111	7.26567558	1.57E-08	9.28E-08
SPAC4A8.15c	cdc3	profilin	0.58900782	7.1893728	5.52E-08	3.04E-07
SPNCRNA.1198	#N/A	#N/A	0.58896499	4.94091577	0.02171675	0.042115276
SPNCRNA.1593	#N/A	#N/A	0.58888446	6.04171327	0.00204872	0.005121793
SPBC887.14c	pfh1	5' to 3' DNA helicase Pif1/Pfh1	0.58831979	7.39959899	3.53E-07	1.73E-06
SPCC330.06c	pmp20	thioredoxin-related chaperone Pmp20 (predicted)	0.58819013	7.52973973	9.62E-07	4.45E-06
SPCC794.12c	mae2	malic enzyme, malate dehydrogenase (oxaloacetate decarb	0.58771634	9.73450648	5.54E-15	6.61E-14
SPNCRNA.1412	#N/A	#N/A	0.58747927	6.55701288	1.04E-05	4.12E-05
SPAC6G10.11c	ubi3	ribosomal-ubiquitin fusion protein Ubi3 (predicted)	0.58706353	8.04079243	3.41E-12	3.18E-11
SPNCRNA.1389	#N/A	#N/A	0.58574045	5.19963101	0.00292441	0.007039472
SPAC4G9.19	SPAC4G9.19	DNAJ domain protein DNAJB family (predicted)	0.58556844	3.91653142	0.15896835	0.232124149
SPBC1773.05c	tms1	hexitol dehydrogenase (predicted)	0.58485663	6.52686436	3.07E-05	0.000110596

SPAC23D3.04c	gpd2	glycerol-3-phosphate dehydrogenase Gpd2	0.58455471	7.70612244	1.21E-10	9.37E-10
SPNCRNA.1125	#N/A	#N/A	0.58449055	2.33066055	0.37619155	0.47188636
SPAC32A11.04c	tif212	translation initiation factor eIF2 beta subunit (predicted)	0.58429647	8.21080377	2.66E-12	2.50E-11
SPAC1F5.08c	yam8	plasma membrane stretch-activated calcium ion channel Ya	0.58416463	5.87966804	0.0006353	0.001793127
SPBC1703.05	rio2	protein kinase, RIO family Rio2 (predicted)	0.58341604	6.06276126	0.00423747	0.009794834
SPAC1565.08	cdc48	AAA family ATPase involved in ubiquitin-mediated protein c	0.58284393	9.73543499	3.97E-12	3.68E-11
SPCC188.02	par1	protein phosphatase PP2A regulatory subunit B-56 Par1	0.57983165	7.19493383	3.63E-06	1.55E-05
SPBC13G1.02	mpg2	mannose-1-phosphate guanyltransferase (predicted)	0.57931793	8.27826528	9.93E-12	8.80E-11
SPBC106.15	idi1	isopentenyl-diphosphate delta-isomerase Idi1	0.57885683	6.30886398	2.56E-05	9.43E-05
SPCC1393.07c	mug4	Schizosaccharomyces specific protein, DNAJ domain	0.57863891	4.3660379	0.10533887	0.163585344
SPAC13A11.05	ysp2	peptidase family M17 cytoplasmic leucyl aminopeptidase ys	0.57847443	7.82722803	6.90E-10	4.90E-09
SPAC1805.10	SPAC1805.10	Schizosaccharomyces specific protein	0.57836655	9.03264617	2.50E-15	3.08E-14
SPNCRNA.1202	#N/A	#N/A	0.5779458	8.53782532	7.87E-14	8.46E-13
SPAC57A7.04c	pabp	poly(A) binding Pabp/Pab1	0.57783698	9.52462332	2.50E-11	2.13E-10
SPAC11G7.05c	mct1	mitochondrial [acyl-carrier protein] S-malonyltransferase M	0.57752321	6.52002302	3.74E-06	1.59E-05
SPAC8F11.05c	mug130	Schizosaccharomyces specific protein	0.57737788	4.13390332	0.08447155	0.135700865
SPBC16A3.04	rsm25	mitochondrial ribosomal protein subunit Rsm25 (predicted)	0.5771394	6.18919703	0.00016521	0.000524739
SPNCRNA.1002	#N/A	#N/A	0.57708268	5.62866653	0.00511057	0.011569333
SPNCRNA.1064	#N/A	#N/A	0.57699276	3.81871398	0.06754219	0.11185802
SPAC19D5.11c	ctf8	Ctf18 RFC-like complex subunit Ctf8	0.57611139	4.34248016	0.04288257	0.076013509
SPAC926.09c	fas1	fatty acid synthase beta subunit Fas1	0.57574658	11.0630916	1.45E-17	2.17E-16
SPBC11G11.06c	sme1	Sm snRNP core protein Sme1	0.57572318	7.15577633	2.09E-07	1.05E-06
SPBC577.02	rpl3801	60S ribosomal protein L38 (predicted)	0.57556569	6.28024634	0.00011593	0.000378173
SPAC664.05	rpl13	60S ribosomal protein L13 (predicted)	0.57533908	8.67767344	4.18E-11	3.44E-10
SPBC1539.07c	fmd1	glutathione-dependent formaldehyde dehydrogenase (predi	0.57472853	7.70854019	2.00E-10	1.51E-09
SPBC1709.07	erg27	3-keto sterol reductase Erg27 (predicted)	0.57440197	6.5620116	1.78E-05	6.74E-05
SPAC22F3.10c	gcs1	glutamate-cysteine ligase Gcs1	0.57386882	8.11585896	1.51E-10	1.16E-09
SPAC17A2.05	osm1	fumarate reductase Osm1 (predicted)	0.57311206	7.45872421	5.77E-08	3.17E-07
SPAC1834.10c	SPAC1834.10c	mitochondrial proton-transporting ATP synthase complex as	0.57294815	5.57834486	0.00142432	0.00368396
SPAC513.01c	eft201	translation elongation factor 2 (EF-2) Eft2,A	0.57282184	10.3345178	8.05E-17	1.13E-15
SPAC24H6.07	rps901	40S ribosomal protein S9	0.57252325	8.11732104	3.10E-11	2.59E-10
SPAC4D7.09	tif223	translation initiation factor eIF2B gamma subunit	0.57251025	7.61327663	1.30E-08	7.79E-08
SPNCRNA.1498	#N/A	#N/A	0.5723733	5.79294292	0.00077559	0.002141158
SPBC21.01	mis17	CENP-U ortholog Mis17	0.57117205	5.40499945	0.007938	0.017105149
SPBC428.13c	mob1	Sid2-Mob1 kinase complex regulatory subunit Mob1	0.57111219	5.50037502	0.00182779	0.00461867
SPAC11D3.06	SPAC11D3.06	MatE family transmembrane transporter (predicted)	0.56978707	6.72873098	4.16E-05	0.000146413
SPNCRNA.1643	#N/A	#N/A	0.56961408	6.93905731	1.25E-05	4.85E-05
SPBP23A10.11c	SPBP23A10.11c	circularly permuted 1,3-beta-glucanase (predicted)	0.56926754	8.88986194	2.36E-07	1.18E-06
SPCC16C4.08c	skb15	p21 activated protein kinase inhibitor Skb15	0.56704553	6.02356451	9.51E-05	0.000314818
SPNCRNA.1523	#N/A	#N/A	0.56703153	5.34503281	0.03378561	0.061887228
SPAC8C9.04	SPAC8C9.04	Schizosaccharomyces specific protein	0.56691012	8.99617613	6.95E-10	4.93E-09
SPBC9B6.10	cdc37	Hsp90 co-chaperone Cdc37	0.56593521	7.29048691	1.40E-05	5.39E-05
SPBPB21E7.07	aes1	phenazine biosynthesis PhzF protein family	0.56560429	7.48792674	0.0002005	0.000628747
SPBC56F2.02	rpl1901	60S ribosomal protein L19	0.56559997	8.37164927	4.74E-11	3.86E-10
SPAC644.17c	mrpl9	mitochondrial ribosomal protein subunit L9 (predicted)	0.5648525	7.93372272	1.09E-11	9.68E-11
SPBC8D2.19	mde3	serine/threonine protein kinase, meiotic Mde3	0.56473724	6.8313239	9.89E-06	3.92E-05
SPAC4G9.16c	rpl901	60S ribosomal protein L9	0.56389225	8.22757691	1.48E-12	1.44E-11

SPCC1393.14	ten1	nuclear telomere cap complex subunit Ten1	0.5636744	3.98440636	0.11994897	0.183427581
SPBC725.15	ura5	orotate phosphoribosyltransferase Ura5	0.56278105	6.69003418	7.96E-06	3.22E-05
SPAC26A3.05	chc1	clathrin heavy chain Chc1 (predicted)	0.56207216	10.2063868	7.29E-13	7.30E-12
SPCC31H12.04c	rpl1202	60S ribosomal protein L12.1/L12A	0.5620254	8.23797477	8.78E-10	6.16E-09
SPBC21D10.08c	SPBC21D10.08c	conserved fungal protein	0.56195747	5.87223805	0.00053665	0.001540243
SPAC922.06	SPAC922.06	3-oxoacyl-[acyl-carrier-protein]reductase (predicted)	0.56184098	6.30928999	0.00014033	0.000451296
SPCC1442.06	pre8	20S proteasome complex subunit alpha 2, Pre8	0.56177661	7.9105707	3.46E-10	2.56E-09
SPAC18G6.14c	rps7	40S ribosomal protein S7 (predicted)	0.56136751	8.40788941	1.59E-11	1.37E-10
SPNCRNA.1114	#N/A	#N/A	0.56045885	2.29839582	0.48960502	0.584417149
SPBC1105.02c	lys4	homocitrate synthase	0.56037719	8.64559221	7.90E-10	5.57E-09
SPCC31H12.05c	sds21	serine/threonine protein phosphatase PP1 subfamily, Sds21	0.55921305	6.60859258	1.54E-05	5.90E-05
SPBC23E6.10c	mri1	methylthioribose-1-phosphate isomerase Mri1 (predicted)	0.55919004	5.68820688	0.01327708	0.027205815
SPAC29E6.06c	SPAC29E6.06c	cytoplasmic cysteine-tRNA ligase Crs1 (predicted)	0.55911867	8.25052042	1.30E-08	7.79E-08
SPAC890.08	rpl31	60S ribosomal protein L31 (predicted)	0.55892103	7.63945971	4.69E-10	3.41E-09
SPAC637.13c	slm1	cytoskeletal signaling protein Slm1 (predicted)	0.55870362	7.32556097	7.82E-07	3.65E-06
SPRRNA.07	SPRRNA.07	5S rRNA	0.55850537	5.68305616	0.00073585	0.002051386
SPAC24B11.13	hem3	hydroxymethylbilane synthase Hem3 (predicted)	0.55848285	6.76780911	8.66E-07	4.03E-06
SPBC29A3.18	cyt1	cytochrome c1 Cyt1 (predicted)	0.55781341	7.58883644	2.19E-08	1.27E-07
SPCC1906.01	mpg1	mannose-1-phosphate guanyltransferase Mpg1	0.55746218	9.17602346	1.80E-14	2.04E-13
SPBC106.12c	tho4	THO complex subunit Tho4 (predicted)	0.55712857	5.90159334	0.00050743	0.001470815
SPNCRNA.925	#N/A	#N/A	0.55553252	9.01699853	1.91E-09	1.28E-08
SPCC1322.13	ade6	phosphoribosylaminoimidazole carboxylase Ade6	0.55551377	7.35702401	3.77E-08	2.14E-07
SPCC830.08c	yop1	ER membrane protein DP1/Yop1	0.55500788	7.48744662	6.73E-08	3.66E-07
SPBC14F5.08	med7	mediator complex subunit Med7	0.55451163	8.01636302	5.87E-09	3.70E-08
SPAC23C11.02c	rps23	40S ribosomal protein S23 (predicted)	0.55443638	7.71588578	5.47E-10	3.94E-09
SPAC343.09	ubx3	UBX domain protein Ubx3, Cdc48 cofactor	0.55390581	7.47387817	1.30E-07	6.76E-07
SPBP22H7.07	prp5	Prp19 complex WD repeat protein Prp5	0.55268215	7.05207471	2.86E-07	1.42E-06
SPNCRNA.1515	#N/A	#N/A	0.55245018	7.83159833	1.59E-10	1.21E-09
SPNCRNA.1289	#N/A	#N/A	0.55194667	6.23399908	0.00197167	0.004960158
SPBC17D11.03c	SPBC17D11.03c	carboxymuconolactone decarboxylase-like protein	0.55177398	5.29685527	0.00317204	0.007579257
SPAC4F10.20	grx1	glutaredoxin Grx1	0.55169043	7.19038636	1.87E-07	9.48E-07
SPBC887.01	adi1	acireductone dioxygenase family Adi1 (predicted)	0.55153291	4.62789735	0.07589028	0.123666997
SPNCRNA.1152	#N/A	#N/A	0.55134862	5.35752926	0.00357965	0.008425984
SPAC30C2.04	asc1	cofactor for cytoplasmic methionyl-and glutamyl-tRNA synt	0.55096253	8.06585527	5.73E-11	4.60E-10
SPBC106.17c	cys2	homoserine O-acetyltransferase (predicted)	0.55021021	6.39999938	0.00039739	0.001173716
SPAC3G9.09c	tif211	translation initiation factor eIF2 alpha subunit	0.54956536	8.16697917	6.48E-11	5.17E-10
SPCC14G10.04	SPCC14G10.04	Schizosaccharomyces specific protein	0.54908032	8.12195812	3.58E-10	2.63E-09
SPAC11E3.13c	gas5	cell wall protein 1,3-beta-glucanosyltransferase Gas5 (pred	0.5490177	8.07989011	6.21E-09	3.91E-08
SPBC4F6.17c	hsp78	mitochondrial heatshock protein Hsp78 (predicted)	0.54857202	8.51256669	3.11E-05	0.000112033
SPAPB17E12.13	rpl1802	60S ribosomal protein L18 (predicted)	0.54850152	8.17919717	1.84E-10	1.40E-09
SPBP23A10.10	ppk32	serine/threonine protein kinase Ppk32	0.54798172	7.06342516	2.31E-07	1.16E-06
SPNCRNA.230	#N/A	#N/A	0.54794751	4.58795967	0.0266649	0.050306244
SPBC3D6.09	dpb4	DNA polymerase epsilon subunit Dpb4	0.54689115	5.3042184	0.00347238	0.008218968
SPBC16G5.16	SPBC16G5.16	transcription factor, zf-fungal binuclear cluster type (predic	0.54645771	7.14101513	2.80E-07	1.39E-06
SPNCRNA.752	#N/A	#N/A	0.54631569	7.47533736	1.50E-07	7.70E-07
SPBC1773.17c	gor2	glyoxylate reductase (predicted)	0.54579691	7.0901477	1.54E-05	5.89E-05
SPBC336.03	efc25	Ras1 guanyl-nucleotide exchange factor Efc25	0.54565156	7.23205479	7.70E-07	3.60E-06

SPNCRNA.1259	#N/A	#N/A	0.54549407	3.28406394	0.17220735	0.247962551
SPAP8A3.07c	SPAP8A3.07c	phospho-2-dehydro-3-deoxyheptonate aldolase (predicted)	0.54546518	8.66380892	2.24E-11	1.92E-10
SPBC16H5.04	snd301	SRP-independent ER targeting protein Snd3a (predicted)	0.54424532	6.4938163	4.03E-05	0.000142474
SPBC336.15	pic1	INCENP ortholog Pic1	0.54415427	6.77669538	6.88E-05	0.000233345
SPNCRNA.1424	#N/A	#N/A	0.543991	2.39063236	0.36241554	0.457982869
SPAC458.02c	SPAC458.02c	mRNP complex (predicted)	0.54342214	7.90420829	2.20E-08	1.28E-07
SPAC3H8.02	csr102	sec14 cytosolic factor family, phospholipid-intermembrane	0.54335807	8.08339573	7.31E-11	5.77E-10
SPBC3D6.02	but2	But2 family protein But2, similar to cell surface molecules	0.54301386	8.75711669	3.32E-06	1.43E-05
SPBC9B6.04c	tuf1	mitochondrial translation elongation factor EF-Tu Tuf1	0.54286777	8.39835474	5.72E-11	4.60E-10
SPBC9B6.11c	SPBC9B6.11c	CCR4/nocturin family endoribonuclease (predicted)	0.54262827	8.23309719	1.49E-08	8.89E-08
SPCC645.04	nse3	Smc5-6 complex non-SMC MAGE family subunit Nse3	0.5424596	5.97930502	0.00046712	0.001361414
SPBC17G9.06c	SPBC17G9.06c	siderophore-iron biosynthesis protein (predicted)	0.54223084	7.78289635	7.39E-10	5.23E-09
SPCC320.03	SPCC320.03	transcription factor (predicted)	0.54136507	7.54039421	2.04E-07	1.03E-06
SPAP7G5.04c	lys1	aminoadipate-semialdehyde dehydrogenase	0.54109557	9.76204078	2.22E-13	2.33E-12
SPCC320.10	srp72	signal recognition particle subunit Srp72 (predicted)	0.54091123	7.02690731	0.00010885	0.000356653
SPBC119.05c	lsb1	Wiskott-Aldrich syndrome homolog binding protein Lsb1 (pr	0.54080704	7.77586032	2.31E-09	1.52E-08
SPNCRNA.362	#N/A	#N/A	0.54080116	4.5474752	0.04158223	0.073920351
SPBC2F12.02c	mrpl7	mitochondrial ribosomal protein subunit L7 (predicted)	0.5406776	6.18095105	0.00436747	0.01005775
SPAC13G6.10c	asl1	cell wall protein Asl1, predicted O-glucosyl hydrolase	0.54024735	9.2507876	7.73E-09	4.81E-08
SPBC1711.03	emc3	ER membrane protein complex subunit Emc3 (predicted)	0.53923407	5.73223109	0.00104089	0.002779928
SPNCRNA.472	#N/A	#N/A	0.53877811	2.13294574	0.40566212	0.500912847
SPNCRNA.1242	#N/A	#N/A	0.53869562	3.16681667	0.40227922	0.497729105
SPCC1223.03c	gut2	glycerol-3-phosphate dehydrogenase Gut2 (predicted)	0.53852011	6.87807248	0.00702102	0.015357777
SPNCRNA.793	#N/A	#N/A	0.53802397	8.34446333	1.33E-11	1.16E-10
SPNCRNA.779	#N/A	#N/A	0.53658183	4.46042822	0.03067264	0.056817887
SPCC2H8.02	SPCC2H8.02	plasma membrane inorganic phosphate transmembrane tra	0.53560159	6.82335645	4.12E-06	1.75E-05
SPAC694.04c	SPAC694.04c	nuclear/mitochondrial metal-dependent protein hydrolase,	0.53553363	6.65644135	0.00026896	0.00082186
SPBC725.17c	rrn11	RNA polymerase I general transcription initiation factor sub	0.53522597	4.97950166	0.03826499	0.06883248
SPAC1B1.01	deb1	transcription factor Deb1/Rdp1	0.5351994	7.07954444	1.78E-05	6.74E-05
SPNCRNA.1531	#N/A	#N/A	0.53515996	6.33120813	0.00056507	0.001611598
SPAC2C4.03c	smd2	Sm snRNP core protein Smd2	0.5349801	5.58297375	0.00176035	0.004468182
SPCC18B5.04	rsm18	mitochondrial ribosomal protein subunit S18 (predicted)	0.53480911	5.33085308	0.01075863	0.022519979
SPBC1A4.01	apc10	anaphase-promoting complex substrate recognition subunit	0.53477452	4.07025763	0.09798	0.154091489
SPBC19C2.15c	phs1	3-hydroxyacyl-CoA dehydratase involved in very long-chain f	0.53390497	6.14073481	0.00035641	0.00106562
SPAC27E2.03c	SPAC27E2.03c	Obg-like ATPase, human OLA1 ortholog (predicted)	0.53362079	8.36559759	1.96E-09	1.31E-08
SPAC1399.03	fur4	plasma membrane uracil transmembrane transporter	0.53344396	6.98102024	1.41E-05	5.42E-05
SPBC36.05c	clr6	histone deacetylase (class I) Clr6	0.53336642	7.14403686	4.86E-07	2.34E-06
SPCC16C4.14c	sfc4	transcription factor TFIIIC complex subunit Sfc4	0.53316111	7.67394061	1.21E-08	7.28E-08
SPAP8A3.09c	paa1	protein phosphatase regulatory subunit Paa1	0.53304828	8.35105009	4.67E-11	3.81E-10
SPBC354.02c	sec61	translocon alpha subunit Sec61	0.53227839	8.17264014	3.07E-10	2.28E-09
SPCC663.04	rpl39	60S ribosomal protein L39	0.53036764	7.07782654	3.48E-06	1.49E-05
SPAC6F6.11c	SPAC6F6.11c	pyridoxine-pyridoxal-pyridoxamine kinase (predicted)	0.52999718	6.18547282	0.00064994	0.001832919
SPAC1A6.07	sle1	eisosome assembly protein Seg1	0.52993543	6.95430533	1.00E-06	4.62E-06
SPAC1006.07	tif1	translation initiation factor eIF4A tif1	0.52965907	9.2907446	4.63E-13	4.71E-12
SPBC21C3.15c	SPBC21C3.15c	aldehyde dehydrogenase, implicated in cellular detoxificatio	0.52964281	7.80237299	1.32E-09	9.06E-09
SPNCRNA.1079	#N/A	#N/A	0.52870157	5.85338665	0.00054723	0.001565989
SPCC645.14c	sti1	chaperone activator Sti1 (predicted)	0.5286505	7.68910627	1.48E-05	5.68E-05

SPAC9G1.05	aip1	actin binding WD repeat protein Aip1	0.52847412	8.2585473	1.14E-08	6.88E-08
SPCC1442.14c	hnt1	adenosine 5'-monophosphoramidase (predicted)	0.52846346	6.48391477	0.00017495	0.000552458
SPAC31G5.13	rpn11	19S proteasome regulatory subunit, thiol-dependent ubiqui	0.52822317	7.74571849	1.25E-08	7.50E-08
SPBC17A3.08	SPBC17A3.08	TatD homolog (predicted)	0.52808671	6.2472013	0.00016092	0.000512412
SPBC19C2.03	rpc10	DNA-directed RNA polymerase I, II and III subunit Rpc10	0.52765496	5.86209088	0.00053162	0.001529019
SPAPB18E9.04c	SPAPB18E9.04c	Schizosaccharomyces pombe specific protein, possible cell	0.52722965	6.64032406	0.04119787	0.073332862
SPNCRNA.739	#N/A	#N/A	0.52563379	5.39763303	0.0052332	0.01182334
SPRRNA.41	SPRRNA.41	5S rRNA	0.52504337	1.09882413	0.7363981	0.803332654
SPAC1006.05c	och1	alpha-1,6-mannosyltransferase Och1	0.52495794	7.60016877	1.51E-07	7.75E-07
SPBC83.13	yhm2	mitochondrial carrier, tricarboxylic acid Yhm2 (predicted)	0.52404624	7.66947579	4.63E-08	2.59E-07
SPAC23C11.09	ala1	mitochondrial and cytoplasmic alanine-tRNA ligase Ala1 (p	0.52364564	9.17757601	6.60E-11	5.26E-10
SPAC3A12.06c	SPAC3A12.06c	sodium/calcium exchanger (predicted)	0.52353334	6.96670302	6.12E-05	0.000209213
SPAP32A8.02	SPAP32A8.02	xylose and arabinose reductase (predicted)	0.52301823	7.76766874	8.71E-09	5.37E-08
SPCC18.18c	fum1	fumarate hydratase (predicted)	0.52284061	8.28871575	5.51E-07	2.64E-06
SPBC17D1.06	dbp3	ATP-dependent RNA helicase Dbp3 (predicted)	0.52279847	7.14889687	0.00026647	0.000815334
SPBC9B6.07	nop52	nucleolar protein Nop52 family Rrp1 (predicted)	0.5209739	5.36733885	0.00885366	0.018875052
SPAC6G10.08	idp1	isocitrate dehydrogenase Idp1 (predicted)	0.52095242	8.44112312	1.84E-10	1.40E-09
SPATRNAMET.02	SPATRNAMET.02	tRNA Methionine	0.52076671	1.82042476	0.53239182	0.623889503
SPAPB8E5.10	SPAPB8E5.10	conserved fungal protein, with meiosis specific splicing	0.51942667	7.2620055	4.17E-07	2.03E-06
SPNCRNA.121	#N/A	#N/A	0.51940202	1.09839285	0.73609483	0.803130563
SPBC16A3.18	cip1	RNA-binding protein Cip1	0.51939815	7.23377852	1.76E-06	7.88E-06
SPAC890.04c	ytm1	ribosome biogenesis WD repeat WDR12/Ytm1 protein (pre	0.51938361	7.02993836	6.63E-06	2.72E-05
SPAC23C11.17	mdm28	mitochondrial inner membrane protein involved in translati	0.51911173	7.03294671	4.12E-06	1.75E-05
SPAPB2B4.01c	gpi12	pig-L, N-acetylglucosaminylphosphatidyl inositoldeacetylas	0.51861097	6.04320177	0.00133383	0.003473677
SPAC9G1.03c	rpl3001	60S ribosomal protein L30 (predicted)	0.51764912	7.29030174	3.57E-07	1.75E-06
SPAC824.09c	SPAC824.09c	GTPase activating protein (predicted)	0.51723448	6.60590161	0.00016525	0.000524739
SPCC338.07c	naa15	NatA N-acetyltransferase complex regulatory subunit Naa1	0.51709147	7.98230523	2.62E-09	1.72E-08
SPAC1783.02c	vps66	1-acylglycerol-3-phosphate O-acyltransferase Vps66 (predi	0.51706674	6.08961046	0.00480638	0.010983005
SPAPB17E12.05	rpl3703	60S ribosomal protein L37 (predicted)	0.5168683	7.21903982	1.56E-06	7.02E-06
SPAC22H12.04c	rps102	40S ribosomal protein S3a (predicted)	0.5166566	8.75452437	3.10E-11	2.59E-10
SPBC16C6.12c	las1	Las1 pre-rRNA processing protein	0.51624253	6.22357713	0.00022136	0.000689104
SPAC25G10.02	cce1	mitochondrial cruciform-cutting endonuclease Cce1	0.51601655	4.63289927	0.06887561	0.113789397
SPCC1322.02	pxd1	structure-specific DNA nuclease regulator Pxd1	0.51540709	5.56097922	0.00892791	0.019015471
SPNCRNA.536	#N/A	#N/A	0.5150183	5.21161452	0.00734895	0.016003064
SPNCRNA.1360	#N/A	#N/A	0.51485814	6.44512824	0.00032485	0.00097901
SPCC1259.02c	erm1	ER metallopeptidase Erm1 (predicted)	0.51432954	8.78371746	1.21E-11	1.07E-10
SPAC57A10.02	cdr2	serine/threonine protein kinase Cdr2	0.51419248	7.25619233	2.77E-05	0.000101196
SPAC29A4.10	rrn5	RNA polymerase I upstream activation factor complex subu	0.5141305	6.26701347	0.00530487	0.011957485
SPAC19B12.02c	gas1	cell wall protein 1,3-beta-glucanosyltransferase Gas1 (pred	0.51402022	9.22007738	8.80E-09	5.41E-08
SPCC338.15	wbp1	dolichyl-di-phosphooligosaccharide-protein glycotransferas	0.51386416	7.98204121	3.92E-09	2.53E-08
SPAC1B2.02c	ugo1	mitochondrial fusion protein Ugo1 (predicted)	0.51216899	7.14974047	6.92E-06	2.82E-05
SPNCRNA.768	#N/A	#N/A	0.51170931	3.94664687	0.16038684	0.233861673
SPAC13D6.02c	byr3	translational activator, zf-CCHC type zinc finger protein (pre	0.51169028	7.79630107	2.05E-08	1.20E-07
SPCC594.02c	SPCC594.02c	DUF2456 family conserved fungal protein	0.51085519	8.42264095	1.01E-08	6.15E-08
SPBC3F6.01c	SPBC3F6.01c	TPR repeat serine/threonine protein phosphatase (predicte	0.5106802	7.11627877	7.64E-06	3.10E-05
SPAC821.03c	slf1	cell cortex node protein Slf1	0.51066109	6.79825325	6.49E-06	2.67E-05
SPAC343.13	gta2	mitochondrial glutamyl-tRNA amidotransferase beta subun	0.51052812	5.71638305	0.01512872	0.030567434

SPBC1D7.03	clg1	cyclin-like protein involved in autophagy Clg1 (predicted)	0.50851315	9.03304506	3.15E-09	2.05E-08
SPCC576.19c	#N/A	#N/A	0.50850273	3.04298573	0.2612899	0.351923919
SPNCRNA.1392	#N/A	#N/A	0.50837811	2.70641833	0.33244871	0.426780113
SPAC10F6.10	rio1	protein kinase, RIO family Rio1 (predicted)	0.50815724	5.6343964	0.00333596	0.007918112
SPAC16A10.04	rho4	Rho family GTPase Rho4	0.50802465	6.04306597	0.00074699	0.002077352
SPBC2G2.13c	dcd1	deoxycytidylate deaminase (predicted)	0.5077934	8.53567951	1.10E-08	6.67E-08
SPNCRNA.1432	#N/A	#N/A	0.50739075	3.69444918	0.16894552	0.24419589
SPBC651.07	SPBC651.07	Schizosaccharomyces specific protein	0.50655528	6.02314947	0.00355462	0.00837841
SPBPB7E8.02	SPBPB7E8.02	PSP1 family protein	0.50626086	8.68133373	9.32E-09	5.72E-08
SPBC1271.14	SPBC1271.14	acetyl-CoA:L-glutamate N-acetyltransferase (predicted)	0.50605847	7.98989019	3.37E-09	2.18E-08
SPBC3F6.03	trr1	thioredoxin reductase Trr1	0.50592426	7.4390879	3.86E-06	1.64E-05
SPBC3B9.11c	ctf1	mRNA cleavage and polyadenylation specificity factor comp	0.50533093	6.25944197	0.00018852	0.000593085
SPNCRNA.1235	#N/A	#N/A	0.5050356	6.95684122	0.00675658	0.014831782
SPAC18G6.13	SPAC18G6.13	Schizosaccharomyces specific protein	0.5045312	4.58240678	0.04052013	0.072296664
SPCC970.03	cbr1	NADH-dependent reductase for Dph3, Cbr1 (predicted)	0.50410449	7.11547827	8.74E-05	0.000290681
SPAC9E9.13	wos2	p23 homolog, predicted co-chaperone Wos2	0.50381881	8.30467464	5.59E-10	4.01E-09
SPNCRNA.1011	#N/A	#N/A	0.50326722	6.08754588	0.00061648	0.001743613
SPCC1672.12c	get4	GET complex (ER membrane insertion) subunit Get4 (predi	0.50286465	6.97968543	1.42E-05	5.48E-05
SPAC806.04c	SPAC806.04c	metal-dependent phosphatase involved in cellular detoxifica	0.5023795	7.2957883	1.35E-06	6.15E-06
SPBC2F12.14c	gua1	IMP dehydrogenase Gua1	0.50177186	8.57621841	2.88E-10	2.16E-09
SPAC14C4.06c	nab2	poly(A) binding protein Nab2 (predicted)	0.50149267	6.23075089	0.0007401	0.002060724
SPAC3G9.16c	bet5	TRAPP complex subunit Bet5 (predicted)	0.50039709	5.29625883	0.03028602	0.056157046
SPAC8E11.07c	alp31	tubulin specific chaperone cofactor A, Alp31	0.49980558	5.70366744	0.00488249	0.011138239
SPAC6G9.14	puf4	pumilio family RNA-binding protein Puf4 (predicted)	0.49963613	7.51849991	1.20E-06	5.49E-06
SPBC947.11c	elg1	DNA replication factor C complex subunit Elg1	0.49963162	6.61830452	0.00239632	0.005904042
SPNCRNA.1335	#N/A	#N/A	0.4994138	4.58268293	0.05482546	0.09376408
SPNCRNA.1322	#N/A	#N/A	0.4990992	7.23386382	1.27E-05	4.92E-05
SPAC664.04c	rps1602	40S ribosomal protein S16 (predicted)	0.49795078	7.33891197	4.80E-07	2.32E-06
SPBC1105.03c	mrpl1601	mitochondrial ribosomal protein subunit L16 (predicted)	0.49788366	6.29643241	0.00060917	0.001724362
SPCC24B10.04	SPCC24B10.04	Schizosaccharomyces specific protein	0.49757786	4.92458828	0.03421774	0.062527308
SPCC584.11c	svf1	Svf1 family protein, lipocalin superfamily Svf1	0.4974212	7.64639645	1.43E-07	7.40E-07
SPNCRNA.240	#N/A	#N/A	0.49676322	2.7757412	0.36126488	0.456953063
SPBC3B9.19	mge1	mitochondrial [2Fe-2S] cluster assembly and protein impor	0.49656494	7.11110333	1.00E-05	3.95E-05
SPBC31F10.10c	SPBC31F10.10c	Armadillo-type fold protein, zf-MYND type zinc finger prote	0.49652688	7.62932844	7.83E-06	3.17E-05
SPBC1861.08c	lea1	U2 snRNP-associated protein Lea1 (predicted)	0.49648569	6.54148809	0.00050835	0.001472673
SPCC18.13	trm82	tRNA (guanine-N7-)-methyltransferase WD repeat subunit	0.49628006	5.98991889	0.00160672	0.004108877
SPAC328.09	SPAC328.09	mitochondrial carrier, 2-oxoadipate/2-oxoglutarate (predict	0.49619711	6.71498504	3.13E-05	0.000112733
ScpofMt28	#N/A	#N/A	0.49592917	4.365566	0.13965859	0.208320114
SPNCRNA.902	#N/A	#N/A	0.49513656	6.40923017	0.0009874	0.002651628
SPAC6F6.19	SPAC6F6.19	RNA-binding protein, G-patch type, human GPATCH11 ortho	0.49511675	3.88310931	0.11243972	0.173111385
SPBC2G2.12	hrs1	mitochondrial and cytoplasmic histidine-tRNA ligase Hrs1 (	0.49495387	8.11376763	3.56E-07	1.75E-06
SPAC6G10.03c	cld1	mitochondrial cardiolipin-specific phospholipase Cld1 (pred	0.49490582	7.57560388	1.89E-07	9.59E-07
SPAPB1E7.07	glt1	glutamate synthase Glt1	0.49421323	10.1869478	1.61E-11	1.39E-10
SPAC16E8.01	shd1	cytoskeletal protein binding protein Sla1 family, Shd1 (pred	0.49419456	9.46074136	1.10E-10	8.52E-10
SPNCRNA.1049	#N/A	#N/A	0.49386653	6.27501134	0.00769844	0.016647768
SPAC26A3.17c	rmt2	N-methyltransferase (predicted)	0.49379812	6.47336484	0.00094661	0.002557917
SPBC1711.13	his2	histidinol dehydrogenase His2 (predicted)	0.49373657	7.92979941	2.08E-09	1.38E-08

SPBC2G2.08	ade9	C-1-tetrahydrofolatesynthase/methylenetetrahydrof olated	0.49370356	7.8609022	1.15E-07	6.01E-07
SPAC4A8.11c	fas2	fatty acid synthase alpha subunit Fas2	0.49335385	10.5358863	9.73E-12	8.64E-11
SPNCRNA.1373	#N/A	#N/A	0.49264721	5.23198293	0.01538698	0.031015518
SPNCRNA.1690	#N/A	#N/A	0.49247689	7.70517578	0.00249946	0.006129307
SPCC162.11c	urk1	uridine kinase/uracil phosphoribosyltransferase (predicted)	0.4920066	6.39373649	0.00116046	0.003067947
SPAC17C9.09c	tim13	Tim8-Tim13 mitochondrial intermembrane space protein tr	0.49180782	5.98199678	0.00204789	0.005121616
SPAC5D6.01	rps2202	40S ribosomal protein S15a (predicted)	0.49142931	6.83991858	7.17E-05	0.000242429
SPAC1B3.01c	SPAC1B3.01c	uracil phosphoribosyltransferase (predicted)	0.49119829	7.36257932	6.21E-07	2.96E-06
SPAC1A6.10	tcd1	tRNA threonylcarbamoyladenosine dehydratase Tcd1 (predi	0.49095169	7.03545579	1.45E-05	5.58E-05
SPCC126.06	twf1	twinfilin (predicted)	0.49085878	6.02151178	0.00527771	0.011912042
SPAC1F12.10c	SPAC1F12.10c	NADPH-hemoprotein reductase (predicted)	0.49010978	5.83649828	0.00286633	0.006924172
SPAC20G4.01	caf16	CCR4-Not complex subunit Caf16 (predicted)	0.4900373	6.38711337	0.0007874	0.002170202
SPAC23A1.07	SPAC23A1.07	ubiquitin-protein ligase E3 (predicted)	0.48969136	5.06465846	0.01567951	0.031521141
SPBC15D4.02	gsf1	transcription factor, zf-fungal binuclear cluster type Gsf1	0.48945436	7.35485686	0.00176205	0.004470824
SPAC13G6.02c	rps101	40S ribosomal protein S3a	0.4894471	8.56845508	3.63E-10	2.66E-09
SPBC28E12.03	rga4	RhoGAP, GTPase activating protein Rga4	0.48867767	7.94736434	2.28E-08	1.32E-07
SPNCRNA.1160	#N/A	#N/A	0.48846257	6.41528808	0.00032508	0.000979277
SPNCRNA.488	#N/A	#N/A	0.48805872	1.90504182	0.60086429	0.684102642
SPBC1289.16c	cao2	copper amine oxidase-like protein Cao2	0.48745995	7.71327092	0.00274382	0.006658939
SPCC736.12c	mmi1	nucleus specific RNA binding exosome specificity factor Mn	0.48728571	7.48348206	4.36E-05	0.000153107
SPAC27D7.07c	smd1	Sm snRNP core protein Smd1	0.48726284	6.10108211	0.00127708	0.003347653
SPAC17G8.06c	SPAC17G8.06c	dihydroxy-acid dehydratase (predicted)	0.48658851	8.9772779	4.59E-10	3.34E-09
SPNCRNA.637	#N/A	#N/A	0.4863741	5.67105973	0.00323507	0.007713622
SPAC750.07c	SPAC750.07c	S. pombe specific protein	0.48617991	4.29892916	0.06312016	0.105744133
SPBC19C7.06	prs1	cytoplasmic proline-tRNA ligase Prs1 (predicted)	0.48527063	9.03049258	5.84E-12	5.31E-11
SPAC6B12.15	cpc2	RACK1 ortholog Cpc2	0.48490222	9.30971944	2.42E-12	2.30E-11
SPAC56E4.05	snd2	SRP-independent ER targeting protein Snd2 (predicted)	0.48470022	5.34751464	0.01987345	0.039007447
SPAPJ696.02	lsb4	actin cortical patch component Lsb4 (predicted)	0.48368946	7.38349832	4.95E-07	2.38E-06
SPAPB18E9.01	trm5	tRNA (guanine(37)-N(1))-methyltransferase activity Trm5 (	0.48272404	5.90872614	0.00798021	0.017181163
SPAP7G5.03	prm1	conjugation protein Prm1	0.48250909	9.8948368	1.38E-11	1.20E-10
SPAC3G6.06c	rad2	FEN-1 endonuclease Rad2	0.48193717	6.06801248	0.00076024	0.002108157
SPBC1685.09	rps29	40S ribosomal protein S29 (predicted)	0.48167708	6.16539907	0.00239464	0.005902042
SPAC3A11.12c	rpt5	19S proteasome base subcomplex ATPase subunit Rpt5	0.48126914	8.27560499	1.08E-08	6.54E-08
SPBC1105.01	SPBC1105.01	rRNA processing protein Rrp12-like (predicted)	0.48066056	7.18463475	6.69E-06	2.74E-05
SPAC30D11.12	rpl3802	60S ribosomal protein L38 (predicted)	0.48021777	6.74928238	0.00010168	0.00033492
SPAC9G1.13c	swc4	Swr1 complex subunit Swc4	0.48012987	6.07415949	0.00097385	0.002621486
SPAC607.04	arg82	inositol polyphosphate kinase Arg82 (predicted)	0.48007125	4.43334398	0.15242022	0.224166758
SPNCRNA.789	#N/A	#N/A	0.48007125	4.43334398	0.15242022	0.224166758
SPCC24B10.20	SPCC24B10.20	short chain dehydrogenase, unknown specificity (predicted)	0.47990827	4.51695429	0.15760616	0.230597701
SPAC9.12c	atp12	mitochondrial F1-FO ATP synthase chaperone Atp12 (predic	0.47972726	5.86047033	0.0024455	0.006012145
SPAC1527.02	sft2	Golgi transport protein Sft2 (predicted)	0.47908311	5.27709329	0.05874119	0.099373793
SPBC16D10.11c	rps1801	40S ribosomal protein S18 (predicted)	0.47907588	7.53288284	1.11E-06	5.09E-06
SPAC2F3.09	hem1	5-aminolevulinate synthase Hem1	0.47883246	8.29444725	4.78E-07	2.31E-06
SPAC212.02	SPAC212.02	Schizosaccharomyces pombe specific protein	0.47872466	5.87189171	0.00223974	0.005564589
SPCC126.04c	sgf73	SAGA complex subunit Sgf73	0.47851931	5.34408901	0.02003381	0.039276892
SPAC56F8.07	SPAC56F8.07	ER membrane integral protein, implicated in sterol metabo	0.47814115	5.94085942	0.00256463	0.006257543
SPAC227.09	fol3	folylpolyglutamate synthase Fol3 (predicted)	0.47809911	6.32654586	0.00098447	0.00264482

SPNCRNA.1448	#N/A	#N/A	0.47801693	7.36106313	1.09E-06	5.02E-06
SPAC3A12.18	zwf1	glucose-6-phosphate 1-dehydrogenase Zwf1	0.47775485	8.59875089	3.37E-09	2.18E-08
SPBC14C8.05c	meu17	glucan-alpha-1,4-glucosidase	0.47771915	5.18993838	0.01910466	0.037726987
SPCC18.16c	fmn1	riboflavin kinase Fmn1	0.47765991	5.34665361	0.02385799	0.045669099
SPCC1393.09c	gir2	RWD domain protein, involved in cytoplasmic translation Gi	0.47754277	6.42254435	0.00042949	0.001260319
SPAC1F7.02c	has1	ATP-dependent RNA helicase Has1 (predicted)	0.47696696	6.79193102	0.00294443	0.007077657
SPAC5H10.10	SPAC5H10.10	NADPH dehydrogenase, (Old yellow enzyme) involved in sm	0.47671234	6.04121126	0.00146482	0.003775785
SPBC29A10.08	gas2	cell wall 1,3-beta-glucanosyltransferase Gas2	0.47663175	9.79631178	8.29E-12	7.42E-11
SPBC18E5.12c	mas2	mitochondrial processing peptidase (MPP) complex alpha s	0.47574957	7.02249556	1.59E-05	6.08E-05
SPCC11E10.03	jnm1	dynactin complex subunit, dynamitin Jnm1	0.47568531	5.11997773	0.05080779	0.087886885
SPAC27F1.03c	uch1	ubiquitin C-terminal hydrolase Uch1	0.47392316	6.17282694	0.00239082	0.005896896
SPBC25H2.06c	hrf1	COPII-coated vesicle component Hrf1 (predicted)	0.47362083	7.4816154	4.53E-06	1.91E-05
SPBC32F12.02	rec14	Ski complex subunit Rec14	0.47295419	5.69597591	0.02194976	0.042518687
SPNCRNA.195	#N/A	#N/A	0.47287268	2.50413633	0.41263285	0.507677914
SPAC10F6.03c	cts1	CTP synthase Cts1 (predicted)	0.47277534	8.72003176	4.19E-09	2.70E-08
SPBC1198.08	dug1	dipeptidase Dug1 (predicted)	0.47261107	8.71278112	4.40E-09	2.82E-08
SPBC19C2.14	smd3	Sm snRNP core protein Smd3	0.47231961	6.91558006	1.66E-05	6.31E-05
SPCC16C4.10	SPCC16C4.10	6-phosphogluconolactonase (predicted)	0.47218557	7.59380097	5.89E-06	2.44E-05
SPBC15C4.04c	SPBC15C4.04c	amino acid transmembrane transporter (predicted)	0.47201426	6.72035359	0.00022382	0.000696101
SPBC16D10.06	zrt1	plasma membrane ZIP zinc transmembrane transporter Zrt	0.47162679	7.88392113	5.17E-05	0.000179198
SPBP35G2.11c	nbr1	cargo receptor for selective autophagy pathway	0.47075063	6.1296735	0.01021329	0.021497496
SPNCRNA.1258	#N/A	#N/A	0.47059053	4.63309235	0.06601545	0.109783751
SPNCRNA.652	#N/A	#N/A	0.4705154	7.80181399	7.78E-07	3.64E-06
SPBC354.13	rga6	RhoGAP, GTPase activating protein Rga6 (predicted)	0.47042007	7.61315792	3.28E-06	1.41E-05
SPNCRNA.1488	#N/A	#N/A	0.46986152	5.42447369	0.00985183	0.02080747
SPAC31G5.01	sap49	U2 snRNP-associated RNA-binding protein Sap49	0.46935579	5.98809029	0.00204143	0.005109208
SPNCRNA.1648	#N/A	#N/A	0.46903494	7.65790119	1.02E-07	5.40E-07
SPAC18G6.12c	SPAC18G6.12c	ThiJ domain protein (predicted)	0.46897156	5.71391256	0.00627645	0.013921516
SPBC725.12	nbl1	Borealin homolog Nbl1	0.46888613	3.67059018	0.17468193	0.251052118
SPAC6G9.05	pcd1	coenzyme A diphosphatase (predicted)	0.46835023	5.2348404	0.02424516	0.046306003
SPCC1393.10	ctr4	plasma membrane copper transporter complex subunit Ctr4	0.46771848	6.99351568	0.00012731	0.000412552
SPBC839.03c	dcn1	neddylation protein Dcn1 (predicted)	0.46769438	5.24818454	0.02099306	0.040858677
SPBC713.03	dld2	mitochondrial D-lactate dehydrogenase, cytochrome Dld2 (p	0.46756172	7.413823	3.27E-05	0.000117167
SPAC17H9.11	gmf1	cofilin/tropomyosin family Glia Maturation Factor homolog	0.46756005	5.82935664	0.00352515	0.008314944
SPAC20H4.03c	tfs1	general transcription elongation factor TFIIS	0.46722961	6.63728982	0.00014457	0.000464289
SPAC23A1.17	bbc1	WIP family cytoskeletal protein Bbc1 (predicted)	0.46722454	8.84125421	5.62E-09	3.56E-08
SPAC4D7.08c	ade4	amidophosphoribosyltransferase Ade4	0.4667897	8.31064678	1.71E-09	1.16E-08
SPCC70.03c	put1	proline dehydrogenase Put1 (predicted)	0.46661604	9.89836641	5.79E-11	4.64E-10
SPBC25B2.09c	mrs1	mitochondrial and cytoplasmic arginine-tRNA ligase Rrs1/N	0.46645018	8.70310221	7.13E-10	5.05E-09
SPNCRNA.1494	#N/A	#N/A	0.46637909	5.21497381	0.02236299	0.043220717
SPBC1A4.07c	sof1	U3 snoRNP-associated protein Sof1 (predicted)	0.46607868	6.57811847	0.00035853	0.001071035
SPBC29A10.09c	tri1	triman, ribonuclease involved in priRNA formation Tri1	0.46598458	9.73473306	2.60E-12	2.46E-11
SPBC25H2.02	trs1	cytoplasmic threonine-tRNA ligase Trs1 (predicted)	0.46590493	9.18364532	2.43E-09	1.60E-08
SPNCRNA.1406	#N/A	#N/A	0.4658328	4.70650299	0.07100831	0.116717766
SPBC14C8.04	ilv6	acetolactate synthase regulatory unit Ilv6 (predicted)	0.46455294	7.86183161	4.11E-08	2.32E-07
SPAC1006.08	etd1	Spg1-binding protein Etd1	0.46391622	6.13484745	0.01054937	0.022122782
SPAC27E2.05	cdc1	DNA polymerase delta small subunit Cdc1	0.46378242	6.87027118	9.14E-05	0.000303075

SPBC9B6.08	clc1	clathrin light chain Clc1	0.4637278	7.23344018	2.44E-06	1.07E-05
SPBC23G7.13c	SPBC23G7.13c	plasma membrane urea transmembrane transporter (predi	0.46352884	5.43885205	0.01057448	0.022161782
SPBC1604.04	SPBC1604.04	mitochondrial carrier, thiamine pyrophosphate (predicted)	0.46321676	5.55291243	0.01310823	0.026876013
SPAC6F6.08c	cdc16	two-component GAP Cdc16	0.4631517	5.80733951	0.00427791	0.009874879
SPAPB8E5.03	mae1	plasma membrane malate/succinate:proton symporter Ma	0.46307412	8.44033535	5.95E-06	2.46E-05
SPCC777.12c	SPCC777.12c	ER stress response thioredoxin family membrane disulfide	0.46303004	6.85267605	0.00020235	0.000633964
SPAC1250.03	ubc14	ubiquitin conjugating enzyme E2 for HECT-type and RBR far	0.46223226	6.19668592	0.00134065	0.003490093
SPBC32F12.11	tdh1	glyceraldehyde-3-phosphate dehydrogenase Tdh1	0.46215467	10.064004	6.30E-10	4.50E-09
SPBC1861.05	SPBC1861.05	bifunctional pseudouridylate synthase/pseudouridine kinase	0.46212444	7.72376307	7.53E-07	3.53E-06
SPBC4C3.10c	pre3	20S proteasome complex subunit beta 1 Pre3	0.4618228	7.17809982	4.10E-05	0.000144761
SPNCRNA.497	#N/A	#N/A	0.46176412	4.41909192	0.06571343	0.109385979
SPBC336.08	spc24	NMS complex subunit Spc24	0.4614616	8.59954782	1.32E-08	7.86E-08
SPBC21C3.18	spo4	serine/threonine protein kinase Spo4	0.46140371	4.89574335	0.10122071	0.158601645
SPNCRNA.359	#N/A	#N/A	0.46103716	3.76422904	0.2031872	0.284153082
SPNCRNA.1184	#N/A	#N/A	0.46099257	5.59286385	0.02110628	0.041037986
SPBC27.01c	pga2	protein trafficking protein Pga2 (predicted)	0.46066405	5.57224051	0.01447687	0.029381179
SPNCRNA.1445	#N/A	#N/A	0.46066405	5.57224051	0.01447687	0.029381179
SPNCRNA.1077	#N/A	#N/A	0.45948046	6.54368698	0.00087107	0.002372002
SPAC24H6.11c	SPAC24H6.11c	sulfate transmembrane transporter (predicted)	0.45933901	7.90848822	5.95E-07	2.84E-06
SPNCRNA.1468	#N/A	#N/A	0.45908241	5.4350098	0.01554048	0.031297119
SPAC22F3.02	atf31	transcription factor Atf31	0.45860429	6.11453889	0.00174169	0.004429074
SPBCPT2R1.02	SPBCPT2R1.02	Schizosaccharomyces pombe specific protein	0.45859882	5.83615888	0.00474736	0.01086273
SPBP35G2.14	puf2	pumilio family RNA-binding protein Puf2	0.4585891	8.97003886	1.05E-07	5.56E-07
SPNCRNA.892	#N/A	#N/A	0.45814248	6.96028727	4.15E-05	0.000146413
SPBC215.07c	pdp2	PWWP domain protein Pdp2 (predicted)	0.45791645	7.35665085	0.00011529	0.000376459
SPCC1840.08c	pdi5	protein disulfide isomerase (predicted)	0.45729569	5.91983502	0.00330644	0.007861754
SPBC4F6.13c	erb1	WD repeat/BOP1NT protein Erb1 (predicted)	0.45684352	7.46958329	4.69E-05	0.000164082
SPBC12C2.13c	fnx1	vacuolar amino acid transmembrane transporter Fnx1	0.45544822	6.36975791	0.00054549	0.001562309
SPBP8B7.01c	pop7	RNAseP RNAse MRP subunit Pop7	0.45532541	5.79365478	0.03178856	0.058576002
SPAC17G8.08c	gdt2	Golgi Ca(2+)/H(+) antiporter Gdt2	0.45510941	7.11744983	4.14E-05	0.000145971
SPBC1289.05c	vma10	V-type ATPase V1 domain subunit G (predicted)	0.45444529	6.52087113	0.00046714	0.001361414
SPAC8C9.11	fra2	iron-sensor Fra2	0.45429278	6.50838072	0.00164448	0.004195971
SPNCRNA.1081	#N/A	#N/A	0.45426913	5.18746289	0.02639872	0.049910719
SPAC823.11	sgp1	sphingosine-1-phosphate phosphatase	0.4537507	6.66864368	0.00310733	0.007440316
SPCC18.11c	sdc1	Lid2 complex Dpy-30 domain subunit Sdc1	0.45244357	6.9413891	5.30E-05	0.00018345
SPCC285.04	SPCC285.04	transthyretin/hydroxyisourate hydrolase (predicted)	0.45206021	6.27147012	0.00109888	0.002923323
SPAC890.03	ppk16	serine/threonine protein kinase Ppk16 (predicted)	0.451252	6.30780037	0.00708796	0.015474346
ScpofMt13	#N/A	#N/A	0.45027071	3.6333526	0.25000166	0.339912353
SPBC27B12.10c	tom7	mitochondrial TOM complex subunit Tom7 (predicted)	0.45016044	6.28303325	0.00242864	0.005977175
SPAC22F8.08	sec24	coincidence detector Sec24/Sfb2 subunit	0.44864907	8.76725006	6.05E-08	3.32E-07
SPCC757.05c	SPCC757.05c	peptidase family M20 protein involved in glutathione catab	0.44863264	6.9126059	0.00049091	0.001425798
SPCC613.04c	rng3	UCS-domain protein Rng3	0.44850892	6.24084009	0.0010594	0.002824933
SPAC19G12.15c	tpp1	trehalose-6-phosphate phosphatase Tpp1	0.44829226	9.01990296	9.06E-08	4.83E-07
SPNCRNA.719	#N/A	#N/A	0.44807359	6.70559913	0.00238405	0.005882339
SPNCRNA.19	#N/A	#N/A	0.44794054	4.53561248	0.09371566	0.148621546
SPCC4G3.07c	phf1	PHD finger protein Phf1	0.4476145	5.87562156	0.01956983	0.038466974
SPCC584.05	sec1	SNARE binding protein Sec1 (predicted)	0.44665314	8.14258788	3.80E-08	2.16E-07

SPBC15C4.05	dhx29	ATP-dependent RNA helicase, human DHX29 ortholog (pred	0.44646427	7.1960867	0.00021456	0.000669438
SPBC18H10.20c	any1	arrestin-related endocytic adaptor Any1	0.44619682	6.86086726	0.00036006	0.001074192
SPAC27F1.02c	cdc8	tropomyosin	0.44597125	7.89621165	5.09E-07	2.45E-06
SPBC16G5.08	trp4	phosphoribosylanthranilate transferase Trp4	0.44564376	6.68256334	0.00129007	0.00337912
SPCC663.09c	SPCC663.09c	short chain dehydrogenase, unknown specificity (predicted)	0.44548654	6.87977861	0.00017042	0.000539401
SPAC1B1.03c	kap95	karyopherin/importin beta family nuclear import signal rec	0.44453629	8.61322668	7.87E-09	4.88E-08
SPBC16A3.05c	rae1	RNA export factor, nucleoporin Rae1	0.44452218	7.18658707	0.00011644	0.000379298
SPCC613.10	qcr2	ubiquinol-cytochrome-c reductase complex core protein Qc	0.44448221	7.80514041	1.80E-06	8.04E-06
SPBC646.09c	int6	eIF3e subunit Int6	0.44336615	8.6269206	1.31E-08	7.81E-08
SPNCRNA.1527	#N/A	#N/A	0.44316234	4.03352564	0.15905877	0.232124149
SPAC1786.03	cut11	spindle pole body docking protein Cut11	0.44305473	6.24737337	0.00493594	0.011230051
SPAC21E11.05c	cyp8	cyclophilin family peptidyl-prolyl cis-trans isomerase Cyp8	0.44176122	7.37309873	3.58E-05	0.000127827
SPBC3B9.13c	rpp102	60S acidic ribosomal protein A3	0.44169177	7.21258512	4.20E-05	0.000147555
SPAC10F6.16	igo1	endosulfine (ENSA) serine/threonine protein kinase Igo1	0.44167635	7.56946181	4.87E-06	2.04E-05
SPBC1709.03	SPBC1709.03	DUF3844 family transmembrane protein, conserved in fung	0.44106729	7.38969885	6.57E-05	0.000223714
SPAC105.02c	SPAC105.02c	ankyrin repeat protein, protein phosphatase regulatory sub	0.44033015	7.48007554	1.78E-05	6.74E-05
SPAC7D4.07c	trx1	cytosolic thioredoxin Trx1	0.43962141	7.11795518	1.47E-05	5.66E-05
ScpofMt20	#N/A	#N/A	0.43909567	5.1355923	0.02597359	0.049165576
SPCC965.07c	gst2	glutathione S-transferase Gst2	0.43909486	7.84337438	1.88E-06	8.37E-06
SPCC1753.01c	ssb2	single-stranded DNA binding protein Ssb2	0.43898945	8.06709166	1.95E-07	9.89E-07
SPAC29A4.20	elp3	elongator complex subunit Elp3	0.43809107	6.85483259	0.00129705	0.003392171
SPBC354.12	gpd3	glyceraldehyde 3-phosphate dehydrogenase Gpd3	0.43802385	9.68910811	8.71E-08	4.65E-07
SPAC4G8.03c	puf5	pumilio family RNA-binding protein Puf5 (predicted)	0.43711926	6.31527303	0.01140766	0.023739791
SPCC550.14	vgl1	KH domain RNA binding protein, vigilin (predicted)	0.43676028	9.54365362	1.61E-08	9.53E-08
SPAPB24D3.08c	SPAPB24D3.08c	NADP-dependent oxidoreductase, implicated in cellular det	0.43653276	7.81873846	3.25E-06	1.40E-05
SPCC1322.03	trp1322	plasma membrane TRP-like calcium ion channel Trp1322	0.43637677	6.07099769	0.01948253	0.038328606
SPNCRNA.623	#N/A	#N/A	0.43632563	6.0335474	0.0384394	0.069109676
SPBC19G7.15	nup44	nucleoporin Nup44	0.43623061	6.42803192	0.00111812	0.002966385
SPCC18.07	rpc53	DNA-directed RNA polymerase III complex subunit Rpc53 (p	0.43604512	5.922978	0.01168335	0.024239394
SPBC1921.05	ape2	M1 aminopeptidase Ape2	0.43537503	9.47946598	3.76E-11	3.11E-10
SPAC959.08	rpl2102	60S ribosomal protein L21 (predicted)	0.43526817	7.9602355	5.92E-07	2.83E-06
SPNCRNA.142	#N/A	#N/A	0.4348544	4.96484429	0.07454153	0.121731485
SPBC428.15	SPBC428.15	Obg-like ATPase, human OLA1 ortholog (predicted)	0.43472243	6.88813706	0.0001493	0.000478545
SPBC3H7.11	trm141	serine tRNA C32 methyl transferase Trm141	0.43466144	5.889259	0.00818551	0.017571733
SPAC29A4.18	prw1	Clr6 histone deacetylase complex subunit Prw1	0.43438673	7.06757131	4.72E-05	0.000165135
SPBC16C6.07c	rpt1	19S proteasome base subcomplex ATPase subunit Rpt1	0.43335405	8.08251674	3.71E-06	1.58E-05
SPNCRNA.1380	#N/A	#N/A	0.43334881	4.96412256	0.06043264	0.101786933
SPNCRNA.322	#N/A	#N/A	0.43334881	4.96412256	0.06043264	0.101786933
SPAC630.03	arp3	Arp2/3 protein complex, actin-like protein subunit Arp3	0.43315448	8.43167345	5.67E-06	2.36E-05
SPNCRNA.827	#N/A	#N/A	0.43295682	3.95463731	0.18078789	0.258349761
SPNCRNA.562	#N/A	#N/A	0.432744	5.66779184	0.0078611	0.016960935
SPCC1223.15c	spc19	DASH complex subunit Spc19	0.4325323	4.28166998	0.24787052	0.337351776
SPNCRNA.679	#N/A	#N/A	0.43225796	4.30755441	0.12596434	0.191037967
SPBP8B7.21	ubp3	ubiquitin C-terminal hydrolase Ubp3	0.43222223	6.65194029	0.00602904	0.013451683
SPAC26A3.15c	nsp1	nucleoporin Nsp1	0.43136314	8.39894624	1.27E-07	6.62E-07
SPAC6G10.07	cbc1	nuclear cap-binding complex large subunit (predicted)	0.43073211	7.62977452	1.75E-05	6.63E-05
SPBC4B4.10c	atg5	autophagy associated protein Atg5	0.43055465	5.20981731	0.03004671	0.055804549

SPCC1259.04	iec3	Ino80 complex subunit Iec3	0.43043919	4.69954606	0.12188699	0.185806672
SPAC4G9.22	SPAC4G9.22	Schizosaccharomyces pombe specific protein	0.43034608	6.54067091	0.0019899	0.004998611
SPAC694.03	SPAC694.03	nicotinamide-nucleotide adenylyltransferase (predicted)	0.42998616	6.9893352	0.00257166	0.006270204
SPBC119.17	cym1	mitochondrial metalloendopeptidase (predicted)	0.42987954	8.46196444	0.0009097	0.002469286
SPAC5D6.02c	mug165	Clr6 histone deacetylase complex subunit Mug165	0.42951722	4.52479508	0.18753536	0.266036715
SPBC359.02	alr2	alanine racemase Alr2 (predicted)	0.42945285	5.95727219	0.01031506	0.021659921
SPBP35G2.12	ysa1	ADP-ribose diphosphatase, NudF subfamily Ysa1 (predicted	0.42939858	6.80730861	0.00044782	0.001310728
SPAC24B11.08c	erv46	COPII-coated vesicle component Erv46 (predicted)	0.42911727	7.04044112	0.00031413	0.000948808
SPBC19G7.09	ulp1	SUMO deconjugating enzyme Ulp1	0.42866951	6.86351696	0.00133335	0.003473677
SPAC24C9.13c	mrp10	mitochondrial ribosomal protein subunit Mrp10 (predicted)	0.4283861	5.11717185	0.03668134	0.066334443
SPAPB17E12.14c	SPAPB17E12.14c	6-phosphofructo-2-kinase/fructose-2,6-bisphosphate 2-pho:	0.42735534	8.9589864	5.68E-08	3.12E-07
SPCC777.11	gta3	mitochondrial glutamyl-tRNA amidotransferase complex su	0.42674203	6.87807859	0.00051882	0.001495995
SPAC9G1.12	cpd1	tRNA (m1A) methyltransferase complex catalytic subunit C	0.42631993	7.11749521	0.00091798	0.002487786
SPAC3A12.13c	hcr1	translation initiation factor eIF3j (p35)	0.42605715	7.13230872	8.57E-05	0.000285317
SPCC830.07c	psi1	DNAJ domain protein Psi1	0.42558817	8.06072431	0.0012152	0.0031953
SPAC17G6.13	slt1	Schizosaccharomyces specific protein Slt1	0.42547053	7.54330527	0.00180567	0.00456788
SPBC16G5.10	rrp42	exosome subunit Rrp42	0.42494069	6.95763323	0.00309903	0.007425661
SPNCRNA.826	#N/A	#N/A	0.42480525	8.34910896	9.09E-08	4.83E-07
SPCC1235.06	get2	GET complex (ER membrane insertion) subunit Get2	0.42477502	5.60880429	0.009956	0.021014439
SPCC1682.13	laf2	Clr6 associated factor 2, Laf2	0.42474077	5.51966919	0.04252677	0.075481127
SPAC29B12.05c	mtq1	mitochondrial N(5)-glutamine methyltransferase (predicted	0.42461975	5.23332432	0.03700331	0.066792443
SPCC14G10.01	cab5	dephospho-CoA kinase (predicted)	0.42433827	5.27383494	0.02038778	0.039798867
SPBC106.01	mph1	dual specificity protein kinase Mph1	0.42431378	6.82665293	0.00431924	0.009963527
SPNCRNA.1399	#N/A	#N/A	0.42402659	3.29826748	0.30025666	0.39322179
SPNCRNA.334	#N/A	#N/A	0.42402659	3.29826748	0.30025666	0.39322179
SPCC757.09c	rnc1	KH domain RNA-binding protein Rnc1	0.42400162	7.27004959	0.00191361	0.004821227
SPAC869.03c	SPAC869.03c	plasma membrane urea transmembrane transporter (predi	0.42376261	4.06941561	0.15691438	0.22963492
SPNCRNA.1430	#N/A	#N/A	0.42351148	3.2984677	0.27645161	0.368584093
SPAC4G9.09c	arg11	N-acetyl-gamma-glutamyl-phosphate reductase/acetylgluta	0.42303818	8.81771513	5.20E-09	3.30E-08
SPAC15F9.02	seh1	SEA complex subunit Seh1	0.42299921	6.69348852	0.00380241	0.008885786
SPAC18B11.10	tup11	transcriptional corepressor Tup11	0.42287497	7.13489146	4.35E-05	0.000152624
SPBC651.06	mug166	Schizosaccharomyces specific protein Mug166	0.42266503	4.68914058	0.18424813	0.262357918
SPBC1703.13c	SPBC1703.13c	mitochondrial carrier, inorganic phosphate (predicted)	0.42218273	8.26224833	9.82E-08	5.20E-07
SPNCRNA.1231	#N/A	#N/A	0.4213347	5.67780501	0.01040305	0.021829412
SPAC31G5.07	dni1	tetraspan protein, claudin Dni1	0.42132608	4.3082617	0.25101819	0.340749801
SPBC18E5.01	SPBC18E5.01	unknown with similarity to bacterial carboxy-cis,cis-mucona	0.42118788	7.49712181	0.00018485	0.000582364
SPCP1E11.02	ppk38	Ark1/Prk1 family protein kinase Ppk38	0.4211129	6.83418453	0.0022397	0.005564589
SPCPJ732.01	vps5	retromer complex subunit Vps5	0.42068064	6.67972978	0.00197086	0.004959954
SPBC4B4.09	usp105	U1 snRNP-associated protein Usp105	0.42028937	6.84197154	0.00131246	0.003431162
SPCC338.05c	mms2	ubiquitin conjugating enzyme E2 Mms2	0.41956075	6.52396548	0.00134646	0.003499876
SPRRNA.37	SPRRNA.37	5S rRNA	0.41745857	5.3599313	0.02484596	0.047287741
SPBC530.03c	bag102	BAG family molecular chaperone regulator Bag102	0.41744422	5.79841591	0.00970878	0.020518098
SPBC17A3.07	pgr1	mitochondrial glutathione reductase Pgr1	0.41736337	8.12617584	1.42E-06	6.45E-06
SPBC17D11.08	dca7	WD repeat protein, DDB1 and CUL4-associated factor Dca7	0.41702954	8.09676279	1.34E-05	5.18E-05
SPAC1705.03c	ecm33	extracellular leucine-rich repeat domain, receptor L domain	0.41685741	9.20207937	3.72E-08	2.11E-07
SPAC14C4.10c	SPAC14C4.10c	Nudix family hydrolase	0.41683121	3.6319554	0.25182587	0.341778032
SPBC4B4.03	rsc1	RSC complex subunit Rsc1	0.4168124	7.42268251	3.84E-05	0.000136227

SPBC1604.06c	noc4	CBF/Mak21 family Noc4 (predicted)	0.4167248	6.56994857	0.00278545	0.006743158
SPAC25G10.08	SPAC25G10.08	translation initiation factor eIF3b (p84)	0.41648345	9.25448963	5.02E-08	2.79E-07
SPAC25B8.10	SPAC25B8.10	trans-aconitate 3-methyltransferase (predicted)	0.41624049	7.25936901	1.75E-05	6.64E-05
SPAC1093.02	pdx3	pyridoxamine 5'-phosphate oxidase Pdx3 (predicted)	0.41590726	6.8950402	0.00051971	0.001496653
SPAC24C9.12c	shm1	serine hydroxymethyltransferase Shm1 (predicted)	0.41575722	8.98313473	1.66E-07	8.46E-07
SPBC28F2.02	mep33	translation machinery associated protein Mep33	0.41574059	4.61506517	0.0907409	0.144645536
SPAC1071.10c	pma1	plasma membrane P-type proton exporting ATPase, P3-type	0.41520688	10.6646606	9.85E-07	4.55E-06
SPAP14E8.05c	SPAP14E8.05c	mitochondrial TMEM14 family protein, implicated in heme	0.41508968	5.08016816	0.0619502	0.103988919
SPAC9E9.06c	SPAC9E9.06c	threonine synthase (predicted)	0.41485481	8.38443334	2.87E-07	1.42E-06
SPNCRNA.908	#N/A	#N/A	0.41475559	8.3729147	7.43E-07	3.49E-06
SPBC30D10.05c	SPBC30D10.05c	sepiapterin reductase (predicted)	0.41453725	7.43693436	6.28E-05	0.000214202
SPAC23H4.09	cdb4	endonuclease Cdb4 (predicted)	0.41442618	8.01527448	1.63E-06	7.34E-06
SPBC3E7.16c	leu3	2-isopropylmalate synthase Leu3	0.41442047	9.31383371	5.09E-09	3.24E-08
SPBC26H8.11c	SPBC26H8.11c	acyl-coenzyme A thioesterase	0.41355051	7.69589185	1.96E-06	8.67E-06
SPAC2F3.05c	SPAC2F3.05c	xylose and arabinose reductase (predicted)	0.41325749	6.2078768	0.07230635	0.118679382
SPBC18H10.03	tif35	translation initiation factor eIF3g	0.41321724	7.86460349	5.40E-06	2.25E-05
SPAC23C4.03	hrk1	haspin related kinase Hrk1	0.41319251	7.75493622	2.64E-05	9.72E-05
SPCC162.07	ent1	epsin	0.41316635	8.74523357	2.02E-07	1.02E-06
SPBC428.18	cdt1	replication licensing factor Cdt1	0.41260712	5.29378524	0.04264106	0.075664249
SPNCRNA.799	#N/A	#N/A	0.41240098	8.01714568	1.52E-05	5.83E-05
SPAC3G9.14	sak1	transcriptional activator Sak1	0.4123949	8.14736274	7.23E-07	3.40E-06
SPBC19F5.04	SPBC19F5.04	aspartate kinase (predicted)	0.41213823	8.23832496	3.33E-06	1.43E-05
SPAC23A1.14c	SPAC23A1.14c	pyridoxal phosphate-dependent transferase, unknown speci	0.41209471	6.85968853	0.00049593	0.001439158
SPCC162.09c	hmg1	3-hydroxy-3-methylglutaryl-CoA reductase Hmg1	0.41194898	8.55430665	1.85E-05	6.98E-05
SPBC3B9.18c	vma7	V-type ATPase V1 subunit F (predicted)	0.41175472	6.25073511	0.00366142	0.008579868
SPAC664.11	ssc1	mitochondrial (2Fe-2S) cluster assembly chaperone Ssc1	0.41136054	9.97979229	8.32E-10	5.86E-09
SPBC13G1.07	swf1	palmitoyltransferase Swf1 (predicted)	0.41121306	5.38727528	0.0229567	0.044192459
SPBC23G7.08c	rga7	RhoGAP, GTPase activating protein Rga7	0.41089369	6.34743551	0.02525975	0.048028106
SPAC22A12.14c	SPAC22A12.14c	BSD domain protein, unknown biological role	0.41071934	6.74949981	0.00509596	0.0115401
SPBC16C6.04	dbl6	double strand break localizing protein Dbl6	0.4104853	5.03952537	0.06161131	0.103496654
SPBC1718.03	ker1	DNA-directed RNA polymerase I complex subunit Ker1	0.41035271	5.7599431	0.01301655	0.026704146
SPBC557.04	ppk29	Ark1/Prk1 family protein kinase Ppk29	0.41024895	8.69316475	5.02E-08	2.79E-07
SPBC36.11	SPBC36.11	Schizosaccharomyces specific protein	0.41007841	6.43009248	0.00290356	0.006994232
SPACUNK4.16c	tps3	alpha,alpha-trehalose-phosphate synthase (predicted)	0.40927843	8.91794106	0.00151226	0.003892174
SPBC428.02c	eca39	branched chain amino acid aminotransferase Eca39	0.4092757	9.06672987	5.13E-08	2.84E-07
SPAC3A11.07	nde2	mitochondrial NADH dehydrogenase (ubiquinone) Nde2 (pre	0.40925606	9.06343387	6.49E-06	2.66E-05
SPBC29A10.16c	SPBC29A10.16c	cytochrome b5 (predicted)	0.4091582	6.95058598	0.00141027	0.003650405
SPBC27B12.06	gpi13	pig-O (predicted)	0.40899545	6.7394286	0.00082436	0.002259261
SPBC3B8.06	SPBC3B8.06	DUF2427 family conserved fungal protein	0.40885762	7.21630314	0.00105707	0.002819822
SPAC323.02c	pup2	20S proteasome complex subunit alpha 5, Pup2	0.40872773	7.65710901	1.69E-05	6.44E-05
SPBC3B9.12	trs23	TRAPP complex subunit Trs23 (predicted)	0.40840871	5.05001399	0.05902596	0.099744645
SPAC17C9.03	tif471	translation initiation factor eIF4G	0.40789049	9.8549761	3.58E-08	2.04E-07
SPAC4F8.06	mrps12	mitochondrial ribosomal protein subunit MrpS12 (predicted	0.40780906	4.38981403	0.12550133	0.190378181
SPAC14C4.12c	laf1	Clr6 L associated factor 1 Laf1	0.40779931	5.64837057	0.04282741	0.075935521
SPBC3H7.14	mug176	BRCT domain protein	0.40769406	5.74680279	0.02779817	0.052111993
SPBC947.01	knk1	AAA family ATPase kink, Knk1	0.40710283	7.58641107	2.40E-05	8.90E-05
SPAC6B12.16	meu26	DUF4451 family conserved fungal protein	0.40698737	3.86206189	0.22068211	0.305461923

SPBC418.01c	his4	imidazoleglycerol-phosphate synthase His4	0.40679953	8.15650142	3.43E-07	1.69E-06
SPAC3A12.14	cam1	calmodulin Cam1	0.40676176	7.71338926	5.32E-06	2.21E-05
SPBC16G5.11c	bag101	BAG family molecular chaperone regulator Bag101 (predict	0.40656081	6.59743024	0.00325828	0.007760794
SPAC24H6.04	hxk1	hexokinase 1	0.40641309	9.39311384	6.52E-09	4.09E-08
SPAC19B12.04	rps3001	40S ribosomal protein S30 (predicted)	0.40593326	6.17728256	0.00641409	0.014180635
SPCC16A11.06c	gpi10	pig-B (predicted)	0.40589886	6.65890704	0.0028995	0.006989416
SPAC1071.11	SPAC1071.11	NADH-dependent flavin oxidoreductase (predicted), possibly	0.40513563	6.10108148	0.01291142	0.02650444
SPCC965.12	dpe2	dipeptidyl peptidase, unknown specificity, implicated in glu	0.40457648	6.35205216	0.00328989	0.007827871
SPNCRNA.1514	#N/A	#N/A	0.40447247	4.95386223	0.06685149	0.110957167
SPCC24B10.13	skb5	protein kinase activator Skb5	0.40430751	6.00900139	0.01375434	0.028073875
SPNCRNA.1173	#N/A	#N/A	0.40385053	6.26904305	0.01652376	0.033071828
SPNCRNA.1309	#N/A	#N/A	0.40371326	5.96763507	0.01501196	0.030358517
SPBC11G11.04	trs20	TRAPP complex subunit Trs20 (predicted)	0.40282568	5.05983557	0.05335077	0.091576586
SPAC4G8.07c	trm2	tRNA (m5U54) methyltransferase Trm2 (predicted)	0.402804	6.30942203	0.01003093	0.021159482
SPNCRNA.252	#N/A	#N/A	0.40217868	1.23618088	0.76995387	0.829171714
SPBC651.01c	nog1	ribosome export GTP binding protein Nog1 (predicted)	0.40201205	7.9787779	3.79E-05	0.000134449
SPAC1420.02c	cct5	chaperonin-containing T-complex epsilon subunit Cct5	0.40196817	8.57662831	2.47E-07	1.24E-06
SPAC3C7.08c	elf1	AAA family ATPase Elf1	0.40156868	8.70742004	3.04E-06	1.32E-05
SPCC777.13	vps35	retromer complex subunit Vps35	0.40118604	7.80651393	3.76E-05	0.000133436
SPBC16D10.09	pcn1	PCNA	0.39965811	7.72394616	3.25E-05	0.000116752
SPBC1289.10c	adn2	transcription factor Adn2 (predicted)	0.39946614	6.76813821	0.00288573	0.006958675
SPAC13D6.03c	trm9	tRNA (uridine) methyltransferase Trm9 (predicted)	0.39921646	5.09280308	0.05436334	0.093067307
SPNCRNA.805	#N/A	#N/A	0.39896693	6.97194177	0.00031988	0.000965306
SPAC926.03	rlc1	myosin II regulatory light chain Rlc1	0.39869235	4.98976951	0.05815953	0.098549697
SPAC1805.17	crm1	karyopherin/importin beta family nuclear export signal rece	0.39852668	8.59359875	4.27E-06	1.80E-05
SPBC31F10.07	lsb5	actin cortical patch component Lsb5 (predicted)	0.39848329	6.13831574	0.01499585	0.030334937
SPBC30D10.13c	pdb1	pyruvate dehydrogenase e1 component beta subunit Pdb1	0.39845915	7.84774054	4.23E-06	1.79E-05
SPAC4G9.08c	rpc2	DNA-directed RNA polymerase III complex subunit Rpc2	0.39831905	7.85620843	6.96E-06	2.84E-05
SPAC3A12.07	rpb11	RNA polymerase II complex subunit Rpb11	0.39826161	6.08507427	0.01663878	0.033253127
SPNCRNA.318	#N/A	#N/A	0.39825663	1.23573102	0.76272761	0.822955581
SPAC15A10.04c	zpr1	EF-1 alpha binding zinc finger protein Zpr1 (predicted)	0.39817852	6.87150273	0.00086438	0.00235662
SPNCRNA.782	#N/A	#N/A	0.39796418	3.43341664	0.31949304	0.413887331
SPNCRNA.442	#N/A	#N/A	0.39792379	4.29892317	0.27602088	0.368081926
SPBC211.02c	cwf3	Prp19 complex subunit Cwf3	0.39789982	7.23529848	0.00085434	0.002332043
SPBC354.14c	vac8	vacuolar protein Vac8 (predicted)	0.39765668	6.44705001	0.00311206	0.007449015
SPAC821.11	pro1	gamma-glutamyl phosphate reductase Pro1	0.39722082	6.89654365	0.00503439	0.011423486
SPCC285.17	spp27	RNA polymerase I upstream activation factor complex subu	0.39688945	7.81241657	4.88E-05	0.000170015
SPAC1687.13c	csn5	COP9/signalosome complex protease subunit Csn5	0.39679766	4.97476043	0.05954352	0.100469532
SPBC30D10.06	lsm4	Lsm2-8 complex subunit Lsm4	0.39607043	7.86397845	2.74E-05	0.000100355
SPCC74.02c	ppn1	mRNA cleavage and polyadenylation specificity factor comp	0.39592413	7.20479192	0.00032993	0.00099343
SPCC1795.11	sum3	translation initiation RNA helicase Sum3	0.39587805	9.88923721	5.64E-09	3.57E-08
SPCPB1C11.01	amt1	plasma membrane ammonium transmembrane transporter	0.39559054	8.1809234	0.00051204	0.001479591
SPBC32H8.05	SPBC32H8.05	UPF0642 conserved fungal protein	0.39492626	5.58483013	0.03402202	0.0622198
SPAC26A3.02	myh1	adenine DNA glycosylase Myh1	0.3947809	6.8591321	0.00114911	0.003040312
SPBC30D10.07c	bpl1	biotin-protein ligase Bpl1 (predicted)	0.39454243	7.86462516	3.12E-05	0.000112529
SPAPB1A10.03	nxt1	mRNA export receptor Nxt1	0.3943768	3.73187634	0.34079324	0.435511362
SPCC645.09	mrpl37	mitochondrial ribosomal protein subunit L37 (predicted)	0.39397255	4.11470618	0.22474618	0.310163568

SPAC27F1.07	ost1	dolichyl-diphospho-oligosaccharide-protein glycosyltransfer	0.39394766	8.23852116	4.57E-06	1.92E-05
SPCC553.12c	SPCC553.12c	transmembrane transporter (predicted)	0.39391599	7.6145565	5.55E-05	0.000191225
SPBC16H5.12c	SPBC16H5.12c	DUF2433 metallo phosphatase superfamily conserved fung	0.39341536	8.75720527	2.32E-06	1.02E-05
SPAC23H4.03c	erv25	COPII-coated vesicle component Erv25 (predicted)	0.39300045	6.8243335	0.00552405	0.012410427
SPAC343.16	lys2	homoaconitate hydratase Lys2	0.39283397	8.66598623	1.45E-07	7.47E-07
SPNCRNA.1363	#N/A	#N/A	0.39278692	6.93694065	0.00051723	0.001492048
SPAC30D11.08c	phf2	Lsd1/2 complex PHD finger protein Phf2	0.39268781	6.50407315	0.00355575	0.00837841
SPNCRNA.1416	#N/A	#N/A	0.3926411	4.90988859	0.07024155	0.115680962
SPCC895.06	elp2	elongator complex WD repeat protein Elp2 (predicted)	0.39222136	7.57861643	0.00016793	0.000532742
SPAC323.04	SPAC323.04	mitochondrial ATPase (predicted)	0.39212747	7.73995448	8.25E-06	3.32E-05
SPCC338.11c	efm2	elongation factor EF2/EF3 methyltransferase lysine methyl	0.39168261	6.22915066	0.01347305	0.027541039
SPBC577.12	dph6	diphthamide synthetase Dph6 (predicted)	0.39164506	6.72241416	0.00447411	0.010289385
SPBC4C3.05c	nuc1	DNA-directed RNA polymerase I complex large subunit Nuc	0.39163085	9.19222946	5.93E-08	3.25E-07
SPAC139.04c	fap2	L-saccharopine oxidase	0.39148015	7.08140008	0.0002261	0.000701925
SPBC119.08	pmk1	MAP kinase Pmk1	0.39141875	6.46200787	0.00416528	0.009647638
SPBC83.17	mbf1	transcriptional coactivator, multiprotein bridging factor Mb	0.39132644	7.24693115	0.00050147	0.001454598
SPBC800.10c	SPBC800.10c	EPS15 repeat family actin cortical patch component (predic	0.39130236	8.78996178	4.08E-06	1.73E-05
SPCC622.14	SPCC622.14	GTPase activating protein (predicted)	0.39127124	7.34043866	9.56E-05	0.000316123
SPAC23H4.08	iwr1	RNA polymerase II nuclear import protein Iwr1 (predicted)	0.39081176	5.5503977	0.03404135	0.062238417
SPBC409.15	tsr2	rRNA processing protein Tsr2 (predicted)	0.39072268	6.26900025	0.00683596	0.014986693
SPBC26H8.08c	grn1	GTPase Grn1	0.3905619	7.2245429	0.00054973	0.001572015
SPAC1B3.02c	SPAC1B3.02c	transcription elongation factor, Elf1 family (predicted)	0.39050022	6.99085851	0.00058042	0.00165123
SPAC24B11.06c	sty1	MAP kinase Sty1	0.39033802	7.64024058	1.38E-05	5.34E-05
SPNCRNA.123	#N/A	#N/A	0.38925456	5.04713287	0.0993843	0.156191719
SPBC1604.08c	imp1	importin alpha family nuclear import signal receptor adapt	0.38918184	7.24616285	0.00094344	0.002551718
SPAP8A3.08	cdc4	myosin II light chain	0.38892061	8.22849629	3.81E-06	1.62E-05
SPCC11E10.07c	tif221	translation initiation factor eIF2B alpha subunit	0.38890582	7.28441474	0.00021062	0.000657775
SPAC4G9.15	ifa38	ketoreductase involved in fatty acid elongation (predicted)	0.38872708	7.48956206	8.89E-05	0.000295345
SPBC1711.05	srp40	nucleocytoplasmic transport chaperone Srp40 (predicted)	0.3886644	7.34365887	0.00014995	0.000480191
SPAC1B1.04c	pan3	PAN complex protein phosphotransferase subunit Pan3 (pre	0.38840295	6.90682583	0.00095341	0.002572553
SPBC409.09c	mis13	NMS complex subunit Mis13/Dsn1	0.38835693	4.92006959	0.07037978	0.115880575
SPBC17G9.11c	pyr1	pyruvate carboxylase Pyr1	0.38824378	10.1695957	9.49E-08	5.03E-07
SPAC17G8.07	yaf9	YEATS family histone acetyltransferase subunit Yaf9	0.38815659	5.63060522	0.03023918	0.056085483
SPAC21E11.06	tif224	translation initiation factor eIF2B delta subunit	0.38784291	7.44220978	7.33E-05	0.000247061
SPBC800.12c	SPBC800.12c	ubiquitin family protein (predicted)	0.387762	4.64591489	0.13168859	0.198436851
SPNCRNA.1069	#N/A	#N/A	0.38730947	4.97059784	0.10793482	0.167082906
SPBC1685.03	sec11	signal peptidase subunit Sec11 (predicted)	0.38721657	6.26173054	0.01165085	0.024179339
SPBC211.05	sap10	splicing factor 3B	0.38574199	4.1589127	0.19309597	0.272787645
SPBC32H8.01c	SPBC32H8.01c	endocytic trafficking protein (predicted)	0.38526917	3.6338324	0.34889944	0.44416153
SPAC23H4.10c	thi4	bifunctional thiamine-phosphate dipyrophosphorylase/hydr	0.3846079	8.40062656	1.91E-06	8.47E-06
SPBC30D10.17c	smi1	cell wall biosynthesis/ cell cycle regulator (predicted)	0.38349841	7.49896558	3.00E-05	0.000108298
SPBC9B6.06	mrpl10	mitochondrial ribosomal protein subunit L15 (predicted)	0.38339279	5.62987847	0.03674437	0.066413128
SPAC1071.06	arp9	SWI/SNF and RSC complex subunit Arp9	0.38325141	7.34996882	0.00036085	0.001075583
SPBC30D10.16	pha2	phrenate dehydratase	0.38238361	5.95262516	0.02085197	0.040611815
SPAC1142.07c	vps32	ESCRT III complex subunit Vps32	0.38181191	6.45565863	0.0040154	0.009327282
SPCC338.10c	cox5	cytochrome c oxidase subunit V (predicted)	0.38175095	7.15088289	0.00016457	0.000523065
SPCC162.05	coq3	hexaprenyldihydroxybenzoate methyltransferase Coq3	0.38134896	6.72210747	0.00136312	0.003537767

SPAC2F3.12c	plp1	thioredoxin fold protein Plp1 (predicted)	0.38134775	6.82163584	0.00172507	0.004391724
SPCC1442.09	trp3	anthranilate synthase component I Trp3	0.38061627	7.8926271	2.68E-05	9.82E-05
SPBPJ4664.04	cop1	coatomer alpha subunit Cop1 (predicted)	0.37993468	9.55448542	1.49E-08	8.87E-08
SPBC725.04	hac1	2-hydroxyacyl-CoA lyase Hac1 (predicted)	0.37959568	6.87724867	0.00514004	0.011628305
SPAC25G10.06	rps2801	40S ribosomal protein S28 (predicted)	0.37958545	6.63256712	0.00132846	0.003465544
SPCC4B3.05c	hem12	uroporphyrinogen decarboxylase Hem12 (predicted)	0.37955693	7.20526894	0.00027587	0.000841151
SPAC926.08c	rpf2	Brix domain ribosome biogenesis protein Rpf2	0.37939394	6.67002887	0.0034976	0.008263492
SPNCRNA.1019	#N/A	#N/A	0.37813003	5.16291201	0.07802624	0.126481322
SPCC794.06	SPCC794.06	transmembrane transporter (predicted)	0.37780488	6.21904433	0.02379596	0.045588823
SPNCRNA.730	#N/A	#N/A	0.37747035	8.69003354	9.43E-07	4.37E-06
SPCC825.03c	psy1	plasma membrane SNARE Psy1	0.37722174	7.57737366	0.00095168	0.002568889
SPAC19B12.09	srp14	signal recognition particle subunit Srp14	0.37720006	5.58656189	0.06777108	0.112155206
SPNCRNA.745	#N/A	#N/A	0.37712066	2.39028237	0.58860251	0.674316514
SPCC1322.05c	lap2	vacuolar aminopeptidase Lap2	0.37709375	7.17799964	0.00097174	0.002618889
SPAC9G1.06c	cyk3	Nebulin-family actin filament anchoring protein Cyk3	0.37648607	8.91303673	5.51E-06	2.29E-05
SPCC1442.10c	rpb3	RNA polymerase II subunit 3	0.37608372	8.29735887	3.35E-06	1.44E-05
SPBC11C11.06c	SPBC11C11.06c	Schizosaccharomyces specific protein	0.37575461	6.38967219	0.0149774	0.030315641
SPCC794.07	lat1	dihydrolipoamide S-acetyltransferase E2, Lat1 (predicted)	0.37546306	8.39902783	1.20E-05	4.67E-05
SPAC1A6.02	wdr55	WD repeat protein, human WDR55 family, involved in ribos	0.37522504	7.13505697	0.00036103	0.001075646
SPNCRNA.913	#N/A	#N/A	0.37489045	7.60764382	7.91E-05	0.000265067
SPBC24C6.11	cwf14	G10 protein	0.37434824	8.36961504	0.00045959	0.00134169
SPBC8D2.10c	rmt3	type I ribosomal protein arginine N-methyltransferase Rmt	0.3741115	7.63695964	0.00077707	0.002144347
SPNCRNA.1230	#N/A	#N/A	0.37361659	6.06799605	0.0192619	0.037982386
SPBC83.01	ucp8	UBA/EH/EF hand domain protein Ucp8	0.37355218	8.34945786	5.38E-05	0.000185672
SPBC1773.12	SPBC1773.12	transcription factor, zf-fungal binuclear cluster type (predic	0.37351579	4.36655307	0.18143343	0.259109027
SPBC17D1.05	SPBC17D1.05	Schizosaccharomyces specific protein	0.37349444	8.14680266	2.03E-05	7.61E-05
SPBC1734.06	rhp18	Rad18 homolog ubiquitin protein ligase E3, Rhp18	0.37335125	6.39755855	0.00522647	0.011812073
SPAC3H8.05c	mms1	Cul8-RING ubiquitin ligase complex subunit Mms1 (predicte	0.3732151	7.37508606	0.00021045	0.000657528
SPAC18B11.07c	rhp6	Rad6 homolog, ubiquitin conjugating enzyme E2 Rhp6	0.37318258	6.71462912	0.00491862	0.0112065
SPAC823.07	pga3	GPI-phospholipase A2 activity regulator Pga3 (predicted)	0.37230129	6.05285454	0.03273165	0.06006982
SPBC27.06c	mgr2	TIM23 translocase complex subunit Mgr2 (predicted)	0.37185863	6.20685848	0.00944248	0.020023716
SPAC25G10.01	SPAC25G10.01	RNA-binding protein involved in histone acetylation	0.37181341	7.30880971	0.00011638	0.000379289
SPAC2G11.07c	ptc3	MAP kinase threonine phosphatase, protein phosphatase 2c	0.37127803	8.72709517	3.49E-07	1.71E-06
SPAC29A4.07	srb6	mediator complex subunit Med22	0.37119089	7.48170167	0.00019225	0.000603714
SPAC25B8.17	ypf1	intramembrane aspartyl protease of the perinuclear ER me	0.37017635	5.69704043	0.02919486	0.054430422
SPNCRNA.734	#N/A	#N/A	0.36974213	6.94922718	0.00110736	0.002942448
SPCC4B3.07	nro1	negative regulator of Ofd1, Nro1	0.36973594	7.69589406	7.83E-05	0.000262506
SPAC1687.07	SPAC1687.07	ER membrane DUF2015 family conserved fungal protein	0.36952813	5.61573693	0.03228138	0.05930745
SPAPB1A10.12c	alo1	D-arabinono-1,4-lactone oxidase (predicted)	0.36937787	7.56618074	0.00145524	0.003756793
SPNCRNA.615	#N/A	#N/A	0.36927197	6.17729199	0.01439134	0.029259961
SPBC409.16c	saw1	recombination protein Saw1 (predicted)	0.36877975	5.99211669	0.01447224	0.029381179
SPAC19G12.11	coq9	ubiquinone biosynthesis protein Coq9 (predicted)	0.36874177	7.02181152	0.00067221	0.001891017
SPAC1071.05	hpm1	ribosome methytransferase involved in ribosome assembly	0.36831988	6.5214373	0.0048379	0.011047628
SPAC30D11.04c	nup124	nucleoporin Nup124	0.36802859	7.92957404	0.00062276	0.001759906
SPAC821.05	tif38	translation initiation factor eIF3h (p40)	0.3676799	8.02469579	2.73E-05	1.00E-04
SPBC543.02c	SPBC543.02c	TPR domain protein, DNAJC7 family	0.36637065	7.67503802	0.0003592	0.00107208
SPAC3F10.04	gsa1	glutathione synthetase large and small subunit Gsa1	0.3657561	8.56356531	6.89E-06	2.82E-05

SPCC1620.05	bet4	Rab geranylgeranyltransferase alpha subunit Bet4 (predicte	0.36569791	8.42874054	4.76E-06	2.00E-05
SPAC29B12.04	snz1	pyridoxine biosynthesis protein	0.36567034	8.65214942	5.31E-05	0.000183462
SPBP8B7.31	SPBP8B7.31	HAD superfamily hydrolase, unknown role	0.36539002	4.65125526	0.17381488	0.250012734
SPAC23C11.01	ice2	ER membrane protein involved triglyceride mobilization ICE	0.36535454	8.25872824	5.27E-06	2.19E-05
SPBC337.09	erg28	Erg28 protein (predicted)	0.36524008	7.1296706	0.00112128	0.002972692
SPBC543.09	yta12	mitochondrial m-AAA protease Yta12 (predicted)	0.36486553	8.32141045	3.48E-06	1.49E-05
SPNCRNA.1015	#N/A	#N/A	0.36484334	6.99779225	0.00327188	0.007790459
SPBC11G11.07	mtr10	karyopherin/importin-beta family nuclear import receptor M	0.36447822	7.00254952	0.00099139	0.002661308
SPAC13G7.08c	crb3	Rix1 complex WD repeat subunit Crb3	0.36421552	6.78223764	0.00240234	0.005914588
SPCC550.15c	rei1	ribosome biogenesis protein Rei1 (predicted)	0.3641385	7.3067609	0.00035828	0.001070755
SPAC16C9.03	nmd3	export adaptor Nmd3 (predicted)	0.36391579	7.22003945	0.0010877	0.002896991
SPAC9E9.02	#N/A	#N/A	0.36384058	4.5367916	0.14570373	0.215687156
SPCC777.05	gtr2	Gtr1/RagA G protein Gtr2	0.36354009	5.87129145	0.02795617	0.052336102
SPCC645.07	rgf1	RhoGEF for Rho1, Rgf1	0.36350433	7.83385969	3.34E-05	0.000119568
SPBC409.18	SPBC409.18	diacylglycerol diphosphate phosphatase (predicted)	0.36332186	7.1559796	0.00034168	0.001024723
SPBC725.06c	ppk31	serine/threonine protein kinase Ppk31	0.36306921	5.93370782	0.03484632	0.063488412
SPNCRNA.602	#N/A	#N/A	0.36232883	4.70531821	0.1519012	0.223548375
SPNCRNA.1351	#N/A	#N/A	0.36212482	5.45730998	0.07051583	0.116048419
SPAC22E12.17c	glo3	ARF GTPase activating protein (predicted)	0.36190084	8.165259	1.09E-05	4.26E-05
ScpofMt34	#N/A	#N/A	0.36122416	4.69449387	0.16970925	0.244987581
SPBC428.12c	SPBC428.12c	peptidyl-prolyl cis-trans isomerase E (predicted)	0.36115992	5.49320248	0.07622957	0.124160418
SPCC794.09c	tef101	translation elongation factor EF-1 alpha Ef1a-a	0.36113826	10.1170289	5.47E-08	3.02E-07
SPBC577.09	ckn1	ERCC-8 DNA repair homolog	0.36101029	5.81308897	0.03676787	0.066437954
SPBC4B4.07c	usp102	U1 snRNP-associated protein Usp102	0.36059548	5.96561306	0.02929638	0.054589768
SPAC17G6.11c	SPAC17G6.11c	sphingolipid biosynthesis protein (predicted)	0.35999762	7.95291585	0.00020604	0.000644356
SPAC9.08c	SPAC9.08c	steroid reductase (predicted)	0.35985216	6.12686261	0.02708994	0.051023252
SPAC16E8.15	tif45	translation initiation factor eIF4E, 4F complex subunit	0.35926971	7.40379117	0.00016551	0.000525325
SPBC20F10.04c	nse4	Smc5-6 complex non-SMC delta-kleisin subunit Nse4	0.35922817	5.64221197	0.05831463	0.098763324
SPCC1494.05c	ubp12	CSN-associated deubiquitinating enzyme Ubp12	0.35900404	7.72158998	0.00026849	0.000820795
SPAC212.12	SPAC212.12	S. pombe specific GPI anchored protein family 1	0.35859597	4.36024237	0.17333699	0.249430796
SPAC24H6.10c	SPAC24H6.10c	phospho-2-dehydro-3-deoxyheptonate aldolase (predicted)	0.35842098	8.08273107	0.00011534	0.000376459
SPAC30D11.07	nth1	DNA endonuclease III	0.35811462	4.53016515	0.20674458	0.288712679
SPBC582.03	cdc13	G2/M B-type cyclin Cdc13	0.35738958	7.85672012	0.0008206	0.002250773
SPBC30D10.14	SPBC30D10.14	dienelactone hydrolase family (predicted)	0.35737752	6.39176857	0.0607074	0.102129503
SPNCRNA.1247	#N/A	#N/A	0.35696946	3.62273707	0.34988816	0.445294361
SPAC17H9.14c	pdi2	ER protein disulfide isomerase Pdi2	0.35622032	8.43327565	3.04E-06	1.32E-05
SPBC26H8.06	grx4	CIA machinery monothiol glutaredoxin Grx4	0.35489393	7.23238745	0.00099547	0.002670153
SPAC17D4.01	pex7	peroxin-7 (predicted)	0.35473401	8.81717571	1.76E-06	7.86E-06
SPAP7G5.02c	gua2	GMP synthase [glutamine-hydrolyzing] Gua2 (predicted)	0.35469106	8.39647723	1.11E-05	4.36E-05
SPAC8F11.04	SPAC8F11.04	U3 snoRNP-associated protein Cic1/Utp30 family (predictec	0.35439671	7.25190296	0.00048058	0.001398185
SPBC1D7.04	mlo3	RNA binding protein Mlo3	0.35424966	7.58089615	0.00018741	0.000589874
SPBC3B9.07c	rpa43	DNA-directed RNA polymerase I complex subunit Rpa43	0.35356693	6.40790922	0.00869765	0.018577373
SPBC18E5.06	rps21	40S ribosomal protein S21	0.3528267	7.41397563	0.00041857	0.001229325
SPCC306.02c	yip3	Rab GTPase binding involved in ER to Golgi vesicle transpor	0.3526025	7.14005348	0.01952238	0.038384794
SPBC19C2.13c	ctu2	cytosolic thiouridylase subunit Ctu2	0.35247129	5.9645691	0.02543006	0.048271005
SPAPB17E12.08	eos1	N-glycosylation protein Eos1 (predicted)	0.35244385	5.16155165	0.08105183	0.130940392
SPAC31G5.11	pac2	cAMP-independent regulatory protein Pac2	0.35242278	7.8827485	7.41E-05	0.000249547

SPNCRNA.1286	#N/A	#N/A	0.35197929	6.9671249	0.00161009	0.004114395
SPAC3F10.15c	spo12	Spo12 family nuclear protein	0.35188546	7.82415668	0.00028886	0.000876235
SPAPB17E12.11	ost3	oligosaccharyltransferase gamma subunit Ost3 (predicted)	0.35187955	7.35779562	0.00177013	0.004489663
SPAC6C3.05	meu43	Schizosaccharomyces specific protein Meu43	0.35179116	5.18194599	0.14195877	0.211374798
SPBC26H8.07c	nda3	tubulin beta Nda3	0.35173265	8.70741289	3.01E-06	1.31E-05
SPAC12G12.08	mrpl1602	mitochondrial ribosomal protein subunit L16 (predicted)	0.35131655	6.74377599	0.00529729	0.011944351
SPBP22H7.09c	mis15	CENP-N ortholog Mis15	0.35121506	4.02217263	0.26894815	0.360344985
SPAC27D7.05c	apc14	anaphase-promoting complex subunit Apc14	0.35105952	5.20532983	0.17468505	0.251052118
SPAC1296.02	cox4	cytochrome c oxidase subunit IV (predicted)	0.35103844	7.12933501	0.00436514	0.010055777
SPBC1734.15	rsc4	RSC complex subunit Rsc4	0.35060684	6.5683317	0.00686182	0.015038547
SPBC1778.06c	fim1	fimbrin	0.3505324	9.33912681	8.68E-07	4.04E-06
SPCC622.15c	SPCC622.15c	Schizosaccharomyces specific protein	0.35013299	7.51006048	0.00319559	0.007630183
SPBC16A3.15c	nda2	tubulin alpha 1	0.35009684	8.49073102	8.62E-06	3.45E-05
SPAC31A2.11c	cuf1	nutritional copper sensing transcription factor Cuf1	0.34961033	5.69101901	0.04458215	0.07833244
SPAC17D4.04	trm401	tRNA (cytosine-5-)-methyltransferase (predicted)	0.34945648	7.19933241	0.00054066	0.001549791
SPBC16G5.12c	top3	DNA topoisomerase III	0.34937046	6.04091647	0.0237757	0.045562834
SPNCRNA.600	SPNCRNA.600	translationally silent transcript from tco1 locus	0.34931025	7.60621474	0.00465104	0.010673946
SPBC36.02c	SPBC36.02c	spermidine family transmembrane transporter (predicted)	0.34920278	7.01614657	0.00174899	0.004444313
SPBC530.11c	SPBC530.11c	transcription factor, zf-fungal binuclear cluster type (predic	0.34900509	7.81306081	9.13E-05	0.000302854
SPBC19F8.05	SPBC19F8.05	Schizosaccharomyces specific protein	0.34859888	7.54495131	0.0018704	0.004721102
SPAC1556.02c	sdh1	succinate dehydrogenase Sdh1 (predicted)	0.34854926	9.41387649	1.21E-07	6.34E-07
SPBC1604.14c	shk1	PAK-related kinase Shk1	0.34835936	7.56834177	0.00075517	0.002096655
SPAC4D7.06c	met8	siroheme synthase Met8 (predicted)	0.34830832	6.7934069	0.00623715	0.013847892
SPNCRNA.1137	#N/A	#N/A	0.34799866	6.44828167	0.01257118	0.025876244
SPNCRNA.1675	#N/A	#N/A	0.34764772	7.01774191	0.00388506	0.009047863
SPCC16C4.18c	taf6	SAGA complex/transcription factor TFIID complex histone H	0.34723809	7.65982831	0.00011143	0.000364569
SPAC12B10.06c	emi5	succinate dehydrogenase complex assembly protein Emi5 (|	0.34678535	6.03881691	0.02409978	0.046067136
SPAC3G6.04	rnp24	RNA-binding protein Rnp24	0.34612015	5.96029281	0.02987951	0.055539484
SPNCRNA.788	#N/A	#N/A	0.34597795	5.40371125	0.07451885	0.121723653
SPBC365.07c	tmf1	Golgi coiled-coil protein Tmf1 (predicted)	0.34565328	5.95805944	0.03961276	0.070956777
SPCC285.12	lsm7	Lsm2-8 complex Lsm7 (predicted)	0.34536004	6.40024544	0.00744319	0.016172072
SPAC1782.06c	phb1	prohibitin Phb1 (predicted)	0.34515588	6.62929828	0.01188114	0.02459725
SPNCRNA.856	#N/A	#N/A	0.34506891	2.9047581	0.47991918	0.574973597
SPCC1183.06	ung1	uracil DNA N-glycosylase Ung1	0.34475593	5.92518475	0.04409539	0.077684038
SPBC211.01	rsm10	mitochondrial ribosomal protein subunit S10 (predicted)	0.34460073	7.11668594	0.00219852	0.005470172
SPAC19D5.02c	pex22	peroxisomal membrane protein Pex22 (predicted)	0.34457831	5.80826186	0.04860662	0.084508962
SPAC11D3.04c	SPAC11D3.04c	polyketide cyclase SnoaL-like domain protein	0.34444842	6.66058402	0.00418465	0.009689189
SPCC126.10	iah1	isoamyl acetate hydrolytic enzyme Iah1 (predicted)	0.34389964	5.7287007	0.03687811	0.06660178
SPCC31H12.08c	ccr4	CCR4-Not complex 3'-5'-exoribonuclease subunit Ccr4	0.34357884	7.86498222	0.00037522	0.001114994
SPAC29A4.08c	prp19	Prp19 complex subunit, ubiquitin-protein ligase E4 Prp19	0.3430905	7.53084631	0.00083086	0.00227618
SPAC11E3.12	SPAC11E3.12	mitochondrial thioredoxin family protein, implicated in sulf	0.34307038	8.40536632	1.73E-05	6.58E-05
SPCC1442.08c	cox12	cytochrome c oxidase assembly protein VIb (predicted)	0.34258205	6.61442578	0.00614851	0.013668923
SPAC22E12.10c	etp1	mitochondrial [2Fe-2S] cluster assembly ferredoxin Etp1/ c	0.34234457	8.46475306	7.77E-06	3.15E-05
SPAPJ695.01c	SPAPJ695.01c	S. pombe specific UPF0321 family protein 3	0.34232386	2.13291368	0.623681	0.705518045
SPNCRNA.746	#N/A	#N/A	0.34225332	3.92376877	0.32147668	0.415665749
SPAC1D4.05c	SPAC1D4.05c	Erd1 homolog (predicted)	0.34214871	8.86078612	3.32E-06	1.43E-05
SPAC24C9.10c	mrp4	mitochondrial ribosomal protein subunit S2 (predicted)	0.34198611	5.63094796	0.08571354	0.137436539

SPNCRNA.1421	#N/A	#N/A	0.34190144	5.9082471	0.04948866	0.085928648
SPAC8C9.06c	ppr4	mitochondrial Cox1 translation regulator Ppr4	0.34174953	8.14842366	3.27E-05	0.00011722
SPAC977.11	fex1	plasma membrane fluoride efflux channel Fex1	0.34125576	6.25634768	0.01684953	0.03363481
SPAC2E1P5.03	erj5	endoplasmic reticulum DNAJ domain protein Erj5 (predicte	0.34117475	7.40832859	0.00084544	0.002311527
SPAC1F7.04	rho1	Rho family GTPase Rho1	0.34094645	8.50657369	3.23E-05	0.000115957
SPAC821.07c	moc3	transcription factor Moc3	0.34080703	6.69224705	0.00638844	0.014137662
SPCC895.07	alp14	TOG/XMAP215 microtubule plus end tracking polymerase A	0.34021513	6.86715393	0.01020049	0.021480508
SPAC3H8.07c	pac10	prefoldin subunit 3 Pac10 (predicted)	0.33996757	5.75147908	0.04095169	0.072970991
SPNCRNA.1059	#N/A	#N/A	0.33989517	2.90524602	0.4696252	0.564429439
SPBC1773.14	arg7	argininosuccinate lyase	0.33967285	5.94615804	0.02726135	0.051317704
SPBC365.06	pmt3	ubiquitin-like protein modifier SUMO	0.33945715	7.34064888	0.00373026	0.008723161
SPBC365.14c	uge1	UDP-glucose 4-epimerase Uge1	0.33927004	8.50786287	1.34E-05	5.19E-05
SPAC222.07c	hri2	eIF2 alpha kinase Hri2	0.33911258	6.21726992	0.02793702	0.052329037
SPCC1393.08	fil1	transcription factor, zf-GATA type	0.33870279	9.09477993	0.00116557	0.003079072
SPBC2G2.15c	mrm2	mitochondrial 2' O-ribose methyltransferase Mrm2 (predict	0.33810626	6.1183918	0.04389393	0.077468482
SPBC4F6.16c	ero11	ER oxidoreductin Ero1a	0.33751712	7.85310782	0.0005113	0.001478073
SPBP4H10.15	aco2	aconitate hydratase/mitochondrial ribosomal protein subur	0.33680608	9.52075331	1.23E-06	5.60E-06
SPAC15A10.07	SPAC15A10.07	Schizosaccharomyces specific protein	0.33670101	5.49923651	0.07346398	0.120347235
SPCC24B10.16c	SPCC24B10.16c	proteasome assembly chaperone Pac4	0.33667445	6.94595014	0.00667307	0.014681612
SPNCRNA.1208	#N/A	#N/A	0.33648055	3.90481533	0.30661919	0.400180972
SPCC338.16	pof3	F-box protein Pof3	0.33581126	5.8000538	0.0430799	0.076283811
SPNCRNA.1506	#N/A	#N/A	0.33520633	2.29942271	0.65878048	0.736487785
SPNCRNA.688	#N/A	#N/A	0.33517567	7.03181908	0.00277596	0.006724955
SPAC664.09	ggt1	gamma-glutamyltranspeptidase Ggt1	0.33496091	6.80661253	0.00599288	0.013379772
SPBC1198.03c	SPBC1198.03c	DUF4646 family conserved fungal protein	0.33421245	5.51372086	0.10405677	0.162000981
SPAC1F7.14c	tam6	mitochondrial DUF4536, human DMAC1 ortholog, possibly I	0.33419996	3.5704888	0.40730302	0.502574266
SPBC1271.07c	SPBC1271.07c	N-acetyltransferase (predicted)	0.33406732	7.10016125	0.02178347	0.042220603
SPNCRNA.112	#N/A	#N/A	0.33377212	5.85574573	0.02881711	0.053799841
SPBC1198.04c	zas1	transcription regulator Zas1	0.33372141	6.03826039	0.05879287	0.099400117
SPNCRNA.1299	#N/A	#N/A	0.3329612	2.68420928	0.58345328	0.66977051
SPAC2C4.14c	ppk11	PAK-related GC kinase Ppk11	0.33284495	7.53354705	0.000419	0.001230053
SPAC1142.08	fhl1	forkhead transcription factor Fhl1	0.33281038	7.4018318	0.00040304	0.001188338
SPNCRNA.853	#N/A	#N/A	0.33186577	6.38421306	0.01588038	0.031877873
SPCC70.07c	tmp1	thymidylate kinase Tmp1	0.33158111	6.20568446	0.06509047	0.10847457
SPAC1687.11	spb1	rRNA methyltransferase Spb1 (predicted)	0.3297525	7.78704943	0.00038671	0.001146148
SPNCRNA.1179	#N/A	#N/A	0.32940523	6.05675971	0.02537759	0.048211747
SPCC1902.02	mug72	oxidoreductase (predicted)	0.32928786	7.29120151	0.00293861	0.007071167
SPBC20F10.09	lsm5	Lsm2-8 complex subunit Lsm5	0.32880094	5.64764796	0.04409896	0.077684038
SPAC20G8.07c	erg2	C-8 sterol isomerase Erg2	0.3281179	6.89297973	0.00774983	0.016747401
SPAC16C9.04c	mot2	CCR4-Not complex ubiquitin-protein ligase E3 subunit Mot2	0.32795058	6.53036995	0.05072424	0.08779913
SPBC29A3.09c	gcn20	AAA family ATPase Gcn20 (predicted)	0.32765904	8.0663315	0.00010545	0.000346143
SPCC24B10.19c	nts1	Clr6 histone deacetylase complex subunit Nts1	0.32762507	5.91469847	0.04199111	0.074608219
SPAC7D4.14c	iss10	NURS complex subunit Iss10	0.32755789	6.08481663	0.02956456	0.055020829
SPBC16H5.11c	skb1	type II protein arginine N-methyltransferase Skb1	0.3273581	7.41100829	0.00092355	0.002501906
SPBC21C3.17c	SPBC21C3.17c	ER membrane DUF4448 family conserved fungal protein	0.32689123	5.97671365	0.11111479	0.171498329
SPAC17H9.16	tom22	mitochondrial TOM complex subunit Tom22 (predicted)	0.32668958	6.90658009	0.00741283	0.016111247
SPBC16G5.01	rpn12	19S proteasome regulatory subunit Rpn12	0.32667815	7.57728142	0.00656552	0.014491227

SPAPB1E7.09	ogm2	protein O-mannosyltransferase Ogm2	0.32608437	8.52793062	2.79E-05	0.000101648
SPAC4F10.02	aap1	aspartyl metalloaminopeptidase Aap1	0.32566338	7.25984129	0.00294265	0.007075874
SPNCRNA.1187	#N/A	#N/A	0.32528195	2.13366978	0.74181479	0.807662133
SPBC646.13	sds23	PP2A-type phosphatase inhibitor Sds23/Moc1	0.3252194	8.34119196	2.85E-05	0.000103819
SPAPB1E7.06c	eme1	Holliday junction resolvase subunit Eme1	0.32516308	7.1870493	0.00778313	0.016808686
SPBC11C11.05	SPBC11C11.05	cell wall 1,6-beta-glucan biosynthesis protein, KRE9 family	0.32513047	6.32231778	0.03401522	0.0622198
SPAC644.04	pct1	RNA 5'-triphosphatase	0.32507291	7.16774019	0.00637186	0.014110148
SPAC1952.14c	mrpl25	mitochondrial ribosomal protein subunit L25 (predicted)	0.32499444	5.31926094	0.14213812	0.211491942
SPBC31F10.14c	hip3	HIRA interacting protein Hip3	0.32466551	8.20772526	0.00028031	0.00085308
SPAC323.01c	pos5	mitochondrial NADH kinase Pos5 (predicted)	0.32466229	5.81733422	0.08514755	0.136561183
SPAC16E8.06c	nop12	RNA-binding protein Nop12 (predicted)	0.32457374	6.78410179	0.01269299	0.026095414
SPBC725.13c	psf2	GINS complex subunit Psf2	0.32457333	6.26055904	0.02308827	0.044420601
SPAC1B3.12c	rpb10	DNA-directed RNA polymerase I, II, and III subunit Rpb10	0.32440069	6.7019922	0.0165747	0.03314582
SPCC320.04c	gem1	ERMES complex GTPase subunit Gem1 (predicted)	0.32399794	6.38546452	0.01874926	0.037132919
SPAC1B3.21	coa3	cytochrome c oxidase assembly protein Coa3 (predicted)	0.32367837	4.60845879	0.18135957	0.259057908
SPAC25H1.09	mde5	alpha-amylase homolog Mde5	0.32356113	6.98132954	0.00302067	0.007245564
SPBC337.05c	cct8	chaperonin-containing T-complex theta subunit Cct8	0.32353276	8.50395875	5.84E-05	0.000200846
SPAC1006.06	rgf2	RhoGEF Rgf2	0.32350705	8.20803195	0.00013884	0.000446911
SPNCRNA.755	#N/A	#N/A	0.32344687	6.2824736	0.03907364	0.070066879
SPBC2G2.07c	mug178	mitochondrial ribosomal protein subunit L51-b (predicted)	0.3232759	5.4719908	0.14111774	0.21022465
SPCC1682.16	rpt4	19S proteasome base subcomplex ATPase subunit Rpt4	0.32325018	7.81037572	0.00120184	0.003166555
SPBC887.19	rft1	ER Man5GlcNac2-PP-Dol translocation protein Rft1 (predict	0.32323283	8.44750581	2.92E-05	0.00010603
ScpofMt24	#N/A	#N/A	0.32309285	3.81873403	0.37930114	0.475017676
SPAC110.03	cdc42	Rho family GTPase Cdc42	0.32267706	7.56714476	0.00604679	0.013486863
SPCC1682.12c	ubp16	ubiquitin C-terminal hydrolase Ubp16	0.32255841	6.50010574	0.01222411	0.025230529
SPBC146.07	prp2	U2AF large subunit (U2AF-59)	0.32247823	7.2057748	0.00331116	0.007870223
SPAC23A1.10	tef102	translation elongation factor EF-1 alpha Ef1a-b	0.32223651	10.0024687	1.73E-06	7.73E-06
SPNCRNA.690	#N/A	#N/A	0.32212619	6.85297242	0.00625608	0.013885399
SPBC19G7.01c	msh2	MutS protein homolog 2	0.32188733	8.42622165	0.00233438	0.005774421
SPAC26F1.05	mug106	Schizosaccharomyces pombe specific protein Mug106	0.32172677	4.76360169	0.16581217	0.240432941
SPCC825.02	gbs1	glucosidase II beta subunit	0.32171895	7.61520223	0.00135435	0.003517682
SPBC1539.02	SPBC1539.02	nuclear protein, human IK ortholog, implicated in meiotic cl	0.32159122	8.40001364	0.00051955	0.001496653
SPBC12C2.11	gfa1	glutamine-fructose-6-phosphate transaminase Gfa1 (predic	0.32151067	9.32602179	3.01E-06	1.31E-05
SPAC824.04	swd22	mRNA cleavage and polyadenylation specificity factor comp	0.32140207	6.26278129	0.04100201	0.07304153
SPNCRNA.1060	#N/A	#N/A	0.32058052	4.1399912	0.29739261	0.390385171
SPBC211.08c	mtr3	exosome subunit Mtr3	0.32027743	7.01012824	0.00264633	0.006433827
SPAC890.06	nup155	nucleoporin, WD repeat Nup155	0.3201436	8.81522247	2.05E-05	7.66E-05
SPAC12G12.05c	taf9	SAGA complex/TFIID complex subunit Taf9	0.32006252	6.42102897	0.03251638	0.05969084
SPAC24B11.09	mpc2	mitochondrial carrier, pyruvate Mpc2 (predicted)	0.31910659	7.13595082	0.04307616	0.076283811
SPNCRNA.1085	#N/A	#N/A	0.31872188	7.83258402	0.00103256	0.002759838
SPAC12B10.09	pet801	mitochondrial carrier, S-adenosylmethionine (predicted)	0.31866561	5.51305063	0.0923688	0.146965096
SPAC824.07	glo2	glyoxalase II	0.31856246	7.05142897	0.06915459	0.114056472
SPNCRNA.871	#N/A	#N/A	0.31840556	4.88492889	0.23011495	0.316669808
SPCC285.08	ret2	coatomer delta subunit Ret2 (predicted)	0.31807684	7.26205935	0.00134383	0.003494358
SPNCRNA.1199	#N/A	#N/A	0.31776308	5.80616851	0.06419734	0.107258255
SPBC725.08	pir2	NURS complex subunit, zf-C2H2 type zinc finger protein	0.31727111	7.21656507	0.00708199	0.015466276
SPCC553.11c	toa2	transcription factor TFIIA complex small subunit Toa2 (pred	0.31681691	5.92464149	0.04485065	0.078743202

SPBC27B12.11c	pho7	transcription factor Pho7	0.31666208	7.49055097	0.00363393	0.008524269
SPBC800.11	SPBC800.11	inosine-uridine preferring nucleoside hydrolase (predicted)	0.31651706	6.56776438	0.02234038	0.043189284
SPAC5H10.09c	ecm31	3-methyl-2-oxobutanoatehydroxymethyltransferase Ecm31	0.31650454	5.5393397	0.14312716	0.212566639
SPCC553.04	cyp9	WD repeat containing cyclophilin family peptidyl-prolyl cis-t	0.31608574	6.60372017	0.01882295	0.037268015
SPAC17H9.04c	nrp1	nucleolar RNA-binding protein, human TEX13A and TEX13B	0.31608199	7.86627385	0.00231239	0.005726272
SPCC613.11c	meu23	mug2/mug135/meu2 family	0.31589547	4.38107276	0.3774115	0.473329388
SPBP22H7.06	nrk1	nicotinamide riboside kinase Nrk1 (predicted)	0.3155954	5.72642898	0.06401386	0.107072222
SPBC776.18c	pmh1	transcription factor TFIIK complex ubiquitin-protein ligase E	0.31552423	5.88157949	0.05311289	0.091280675
SPBC2A9.07c	hpz1	zf-PARP type zinc finger protein, G1-S transition regulator H	0.31537317	6.22698613	0.05413695	0.092726392
SPBPJ4664.05	SPBPJ4664.05	endomembrane system protein, FAR-17a/AIG1-like family	0.3153022	6.07544143	0.0796541	0.128905145
SPBC21C3.09c	oaa1	mitochondrial acylpyruvase Oaa1 (predicted)	0.31520823	6.58900728	0.01761047	0.03504071
SPAC1D4.04	cct2	chaperonin-containing T-complex beta subunit Cct2	0.3148561	8.69571409	2.45E-05	9.05E-05
SPAC630.04c	SPAC630.04c	Schizosaccharomyces specific protein	0.31481855	8.76089761	0.00038056	0.001128919
SPBC685.09	orc2	origin recognition complex subunit Orc2	0.31455724	6.25734301	0.04387849	0.077468482
SPCC16A11.16c	rpn1302	19S proteasome regulatory subunit Rpn13b	0.31424546	6.85303262	0.04617475	0.080817681
SPCC18.20	#N/A	#N/A	0.31366033	4.24230921	0.31707334	0.411300821
SPAC2F3.11	ppx1	exopolyphosphatase, prune Ppx1 (predicted)	0.31348726	7.28353766	0.00485939	0.01109298
SPCC11E10.05c	ynd1	nucleoside diphosphatase Ynd1	0.31316998	6.43361669	0.01716429	0.034232989
SPAC18B11.02c	pus9	tRNA pseudouridine synthase Pus9 (predicted)	0.31295056	6.71512637	0.00682536	0.014968289
SPCC338.17c	rad21	mitotic cohesin complex, non-SMC subunit Rad21 (kleisin)	0.31264203	7.45679141	0.00240171	0.005914588
SPCC1795.12c	SPCC1795.12c	Schizosaccharomyces specific protein	0.31188933	4.21213801	0.46121497	0.55648367
SPCC1672.05c	yrs1	cytoplasmic tyrosine-tRNA ligase Yrs1 (predicted)	0.31096971	8.33712689	0.00011196	0.00036595
SPCC576.14	dph5	diphthine synthase Dph5 (predicted)	0.31084718	7.02975625	0.00439171	0.010110139
SPAC328.10c	rps502	40S ribosomal protein S5 (predicted)	0.31063457	8.35594755	5.82E-05	0.000200094
SPNCRNA.1345	#N/A	#N/A	0.31043847	6.87036423	0.02251318	0.043461612
SPNCRNA.568	#N/A	#N/A	0.31031394	7.26088758	0.00577727	0.012936598
SPAC18B11.03c	SPAC18B11.03c	N-acetyltransferase (predicted)	0.31018898	7.15733408	0.00611363	0.01360027
SPBC646.10c	nop56	U3 snoRNP protein Nop56 (predicted)	0.31003971	8.53358259	0.00024609	0.000759144
SPAC9G1.15c	mzt1	mitotic spindle organizing protein Mzt1	0.30980206	4.02220448	0.32519687	0.419758093
SPNCRNA.1156	#N/A	#N/A	0.3097375	2.94634632	0.57708577	0.664478628
SPCC736.03c	msf1	mitochondrial phenylalanyl-tRNA synthetase Msf1 (predicte	0.30965502	6.05754394	0.05851185	0.099072684
SPBC1105.15c	htd2	3-hydroxyacyl-ACP dehydratase Htd2 (predicted)	0.30950509	4.33873901	0.28755995	0.380413192
SPCC1259.15c	ubc11	ubiquitin conjugating enzyme E2, Ubc11/UbcP4	0.30948675	6.34147448	0.02154633	0.041808603
SPAC1142.05	ctr5	plasma membrane copper transporter complex subunit Ctr5	0.30888553	6.99872957	0.0101371	0.021363572
SPCP1E11.11	puf6	pumilio family RNA-binding protein Puf6 (predicted)	0.30868715	7.89785541	0.00053107	0.001528092
SPNCRNA.1329	#N/A	#N/A	0.30857912	5.33897624	0.10178858	0.15927139
SPCC790.03	rbd2	Golgi rhomboid protease Rbd2	0.30785785	4.53060624	0.32726421	0.421946367
SPBC725.01	maa1	mitochondrial aspartate aminotransferase Maa1 (predictec	0.30779901	8.30280494	0.00038711	0.001146841
SPAC1782.11	met14	adenylyl-sulfate kinase (predicted)	0.3077111	6.88842514	0.0100893	0.021269414
SPCC1020.09	gnr1	heterotrimeric G protein beta (WD repeat) subunit Gnr1	0.30735755	4.67663557	0.20240007	0.283401738
SPBPB8B6.06c	fex2	plasma membrane fluoride export channel Fex2	0.30699843	6.37685215	0.02588979	0.049027013
SPBC19C2.12	mrpl51	mitochondrial ribosomal protein subunit L51 (predicted)	0.30690176	5.29109865	0.1691939	0.244362879
SPCC338.04	cid2	mitochondrial eukaryotic conserved protein Cid2	0.30662776	5.37977366	0.11331457	0.174182436
SPBC32H8.09	wdr8	mitosis-specific spindle pole body WD repeat protein Wdr8	0.30660732	5.43000966	0.1138978	0.17503941
SPAC222.11	hem13	coproporphyrinogen III oxidase Hem13 (predicted)	0.30655469	6.98199107	0.0120095	0.024810147
SPAC1751.03	tif313	translation initiation factor eIF3m	0.3065103	8.41476853	5.95E-05	0.000204218
SPAC15E1.03	rpl42	60S ribosomal protein L36/L42	0.30608497	7.5501158	0.00154156	0.003961604

SPNCRNA.1382	#N/A	#N/A	0.30524	7.52911857	0.00098259	0.002640804
SPCC1450.12	SPCC1450.12	mitochondrial DUF3818 and PXA domain conserved fungal	0.30497814	7.40335236	0.00899848	0.019147795
SPBC409.10	ade7	phosphoribosylamidoimidazolesuccinocarboxamide synthas	0.30497255	7.33043723	0.00525007	0.011857521
SPBC106.07c	nat2	N alpha-acetylation related protein Nat2 (predicted)	0.30473849	8.58600238	0.00033203	0.000998882
SPNCRNA.1682	#N/A	#N/A	0.30420736	5.93969068	0.06510068	0.10847457
SPNCRNA.1678	#N/A	#N/A	0.30409493	7.17102102	0.01337	0.027363264
SPBC8E4.04	SPBC8E4.04	alditol NADP+ 1-oxidoreductase activity (predicted)	0.30399085	8.39877941	0.00057706	0.001643056
SPAC19A8.07c	imp4	U3 snoRNP-associated protein Imp4 (predicted)	0.30397355	6.2416851	0.02649195	0.050021566
SPAC8C9.10c	rrp14	ribosome biogenesis protein Rrp14 (predicted)	0.30386519	6.41072085	0.05190413	0.089487618
SPBC16C6.13c	sec27	coatomer beta' subunit (predicted)	0.3034953	8.77780883	0.00012487	0.000405216
SPBC1271.02	stt3	oligosaccharyltransferase subunit Stt3	0.30334194	9.1954497	8.22E-06	3.32E-05
SPCC16A11.02	utp13	U3 snoRNP-associated protein Utp13 (predicted)	0.30252785	7.09608339	0.00658804	0.014527406
SPAC4G9.06c	chz1	histone H2A-H2B dimer chaperone Chz1 (predicted)	0.30245403	7.30842982	0.0074514	0.016184737
SPBC1703.07	acl1	ATP citrate synthase subunit 1 (predicted)	0.30190188	9.03016	9.85E-05	0.000325459
SPAC664.13	SPAC664.13	Schizosaccharomyces pombe specific protein	0.30177615	3.93627425	0.32101102	0.415356293
SPBP23A10.07	rpa2	DNA-directed RNA polymerase I complex subunit Rpa2	0.30167073	8.80122631	6.07E-05	0.000207522
SPBC16H5.06	rip1	ubiquinol-cytochrome-c reductase complex subunit 5, Rip1	0.30067366	7.90684667	0.00188638	0.00475791
SPBC83.04	apc15	anaphase-promoting complex, platform subcomplex scaffol	0.30060326	5.03154045	0.18605632	0.264213944
SPAC19A8.13	usp101	U1 snRNP-associated protein Usp101	0.30015711	6.70386609	0.01521569	0.03071574
SPCC24B10.08c	ada2	SAGA complex subunit Ada2	0.29971372	5.85312695	0.06882801	0.113738365
SPNCRNA.128	rrk1	RNase P K-RNA	0.29969753	7.78527656	0.00155531	0.003992418
SPCC126.01c	SPCC126.01c	WD repeat protein	0.29959643	6.00188423	0.07011826	0.115505873
SPAC6B12.04c	SPAC6B12.04c	2-aminoadipate transaminase/kynurenine-oxoglutarate tra	0.29928692	7.40866361	0.00851156	0.01820847
SPAC9G1.04	oxa101	mitochondrial inner membrane insertase Oxa101	0.29900694	6.22822445	0.0386185	0.069340072
SPAC222.06	mak16	nuclear HMG-like acidic protein Mak16 (predicted)	0.29897036	5.8884308	0.05646623	0.096111235
SPNCRNA.787	#N/A	#N/A	0.29864259	6.32586277	0.14051408	0.209462933
SPBC577.08c	txl1	thioredoxin-like I protein Txl1	0.29802788	7.13877942	0.01091575	0.022792776
SPCC1020.01c	pma2	P-type proton ATPase, P3-type Pma2	0.29757668	7.35072122	0.00174631	0.004439156
SPAC144.01	SPAC144.01	Schizosaccharomyces specific protein	0.29738587	4.83121747	0.18444584	0.262456954
SPNCRNA.1009	#N/A	#N/A	0.29738587	4.83121747	0.18444584	0.262456954
SPAC17C9.12	scs22	VAP family protein Scs22	0.29711111	8.41276663	0.00024902	0.000766766
SPBC1734.12c	alg12	dolichyl pyrophosphate Man7GlcNAc2 alpha-1,6-mannosylt	0.29703907	6.92209458	0.00757145	0.01641406
SPAC1805.09c	fmt1	mitochondrial methionyl-tRNA formyltransferase Fmt1 (pre	0.29703428	5.25614076	0.14705408	0.217402351
SPAC2F7.07c	cph2	Clr6 histone deacetylase associated PHD protein Cph2	0.29648364	7.59745298	0.00349435	0.008262364
SPNCRNA.545	#N/A	#N/A	0.29632574	2.20278455	0.74453954	0.809729744
SPBC106.06	cct4	chaperonin-containing T-complex delta subunit Cct4	0.29627409	8.39064241	0.00072158	0.002015755
SPBC1685.10	rps27	40S ribosomal protein S27 (predicted)	0.29621763	7.26297034	0.00352234	0.008311212
SPAC5H10.12c	otg1	alpha-1,3-galactosyltransferase (predicted)	0.29618752	6.19765411	0.05052572	0.087577051
SPNCRNA.67	#N/A	#N/A	0.29567707	5.89066307	0.12620785	0.191265755
SPAC977.15	SPAC977.15	dienelactone hydrolase family, implicated in cellular detoxi	0.29521355	4.91406033	0.23892289	0.327136878
SPBC23G7.10c	SPBC23G7.10c	NADH-dependent flavin oxidoreductase, implicated in cellul	0.29509671	6.42380161	0.11005311	0.170091171
SPBC12C2.06	dbp5	cytoplasmic ATP-dependent RNA helicase Dbp5 (predicted)	0.29496016	8.01630648	0.00058544	0.001664136
SPCC1494.04c	tyr1	prephenate dehydrogenase Tyr1	0.29488279	6.55244326	0.0442739	0.0778596
SPNCRNA.1644	#N/A	#N/A	0.29406404	5.06773942	0.30696276	0.400444696
SPAPB1E7.02c	mcl1	DNA polymerase alpha accessory factor Mcl1	0.29355315	7.24720909	0.01197934	0.024762888
SPNCRNA.759	#N/A	#N/A	0.29346612	4.99046574	0.22586211	0.311573412
SPBC19G7.02	abz2	4-amino-4-deoxychorismate lyase Abz2 (predicted)	0.29344108	6.37159935	0.05290633	0.091007974

SPCC594.03	SPCC594.03	Schizosaccharomyces pombe specific protein	0.2934114	4.41108198	0.26783541	0.359136936
SPNCRNA.228	#N/A	#N/A	0.29337711	3.82976396	0.42403481	0.519047528
SPBC25D12.04	suc22	ribonucleotide reductase small subunit Suc22	0.29292355	8.25624482	0.00035889	0.001071634
SPBC409.20c	psh3	ER chaperone SHR3 homologue Psh3	0.29274713	7.11418194	0.04725438	0.082452835
SPNCRNA.1664	#N/A	#N/A	0.29265595	6.32620281	0.07343636	0.120330945
SPAC27D7.06	etf1	electron transfer flavoprotein alpha subunit EtfA (predicted	0.29193527	7.3199986	0.00286974	0.006927487
SPAC1782.04	cox24	mitochondrial mRNA processing protein Cox24/Pet20 (pred	0.29185225	5.51247596	0.10790965	0.167081954
SPBC1289.11	spf38	U5 snRNP complex subunit Spf38	0.29181295	6.32946176	0.02740629	0.051547764
SPBC1703.08c	SPBC1703.08c	5-formyltetrahydrofolate cyclo-ligase (predicted)	0.29165472	7.98754595	0.00181335	0.004583897
SPBC947.12	kms2	mitotic and meiotic spindle pole body KASH domain protein	0.29130183	5.04657475	0.20286121	0.283872206
SPAC2F3.18c	SPAC2F3.18c	small endoribonuclease with 2 transmembrane domains (p	0.29114842	6.28683413	0.04156837	0.073915013
SPBC1215.01	shy1	cytochrome c oxidase assembly protein Shy1 (predicted)	0.29100523	8.62128572	0.0007898	0.002174196
SPAP8A3.11c	mtg2	mitochondrial translation factor (GTPase) Mtg2 (predicted)	0.29070712	4.94898192	0.17053591	0.245815894
SPNCRNA.477	#N/A	#N/A	0.29058449	3.73042128	0.44919562	0.544782781
SPNCRNA.1237	#N/A	#N/A	0.29042129	4.63893829	0.32112642	0.415370712
SPAC4A8.16c	tif33	translation initiation factor eIF3c	0.29040039	9.31357276	9.88E-05	0.000326162
SPBC26H8.13c	SPBC26H8.13c	Siva family protein, human apoptosis-inducing factor orthol	0.29013809	5.34485721	0.17190465	0.247579078
SPNCRNA.891	#N/A	#N/A	0.29012927	5.30490891	0.21359299	0.297239323
SPAC4G8.02c	sss1	translocon gamma subunit Sss1 (predicted)	0.29005554	6.30514236	0.05634712	0.095932491
SPBC1539.04	tts1	tetra spanning protein 1, Tts1	0.28990534	6.9778757	0.02208309	0.04276387
SPAC15A10.02	taf12	transcription factor TFIID complex subunit A/ SAGA comple	0.28985781	7.2477156	0.01193364	0.024683407
SPNCRNA.1310	#N/A	#N/A	0.28960366	1.73054283	0.82385026	0.872983651
SPBC1347.04	tim54	TIM22 inner membrane protein insertion complex subunit T	0.28915949	6.32427963	0.07627221	0.124200141
SPBC2G5.01	SPBC2G5.01	ER protein involved in ER-nucleus signaling (predicted)	0.28873568	7.40831074	0.00282369	0.006828435
SPBC1703.10	ypt1	GTPase Ypt1	0.28852078	7.4413374	0.01255794	0.025856809
SPAC3F10.10c	map3	pheromone M-factor receptor Map3	0.28849426	6.9894633	0.01369667	0.027964557
SPBC27B12.12c	SPBC27B12.12c	CorA family magnesium ion transmembrane transporter (pı	0.28837782	8.82878106	4.87E-05	0.000169902
SPAC30C2.05	erv14	cornichon family protein Erv14 (predicted)	0.2881988	5.51192274	0.17258097	0.248447952
SPAC22F3.03c	rdh54	ATP-dependent DNA helicase Rdh54	0.28816553	6.47575694	0.02779577	0.052111993
SPNCRNA.806	#N/A	#N/A	0.28778521	4.9347689	0.16511837	0.239580071
SPNCRNA.1158	#N/A	#N/A	0.28749881	4.694764	0.24995311	0.339912353
SPAC644.08	utr4	methionine salvage haloacid dehalogenase-like hydrolase U	0.28723344	6.13310674	0.09221889	0.146795218
SPAC6F6.17	rif1	telomere length regulator protein Rif1	0.28720374	8.01384423	0.00505633	0.011461809
SPACUNK4.13c	SPACUNK4.13c	mitochondrial NTPase Obg family, human OLA1 ortholog, ir	0.28718166	5.88498313	0.10594922	0.164383136
SPBC19G7.10c	pdc2	topoisomerase II-associated deadenylation-dependent mRN	0.28714742	8.43693098	0.00039904	0.001178087
SPAC16E8.10c	rsm7	mitochondrial ribosomal protein subunit S7, Rsm7(predicte	0.28669067	6.088033	0.06801005	0.112523318
SPCC1494.08c	SPCC1494.08c	cortical variant C2 domain protein, human FAM102A and FA	0.28603175	5.62226484	0.13691189	0.204946203
SPBC1711.16	pwp1	WD repeat protein Pwp1 (predicted)	0.28600176	5.50648262	0.13843042	0.206673756
SPAC26F1.03	pda1	pyruvate dehydrogenase e1 component alpha subunit Pda1	0.28527668	8.92202039	0.00021899	0.000682336
SPBC1A4.09	pus7	tRNA/snRNA/rRNA pseudouridine synthase Pus7 (predictec	0.2849281	8.01722646	0.0020765	0.00518174
SPBC1198.05	SPBC1198.05	guanylate kinase (predicted)	0.28449239	7.73237078	0.00132487	0.003458637
SPAC1002.13c	psu1	cell wall beta-glucosidase Psu1 (predicted)	0.28388551	9.374978	0.00329349	0.007833686
SPCC4G3.16	rib2	CMP deaminase family/ methyltransferase bifunctional enz	0.28387274	5.65031112	0.09368469	0.148607071
SPAC31F12.01	zds1	zds family protein phosphatase type A regulator Zds1 (pred	0.28380284	7.90449559	0.0017787	0.004506349
SPBC25D12.05	trm1	tRNA (guanine-N2-)-methyltransferase Trm1	0.28315567	6.67767074	0.04142683	0.073682583
SPAC23A1.05	SPAC23A1.05	serine palmitoyltransferase subunit A (predicted)	0.28288798	6.08874009	0.16444943	0.238915105
SPNCRNA.957	#N/A	#N/A	0.28283433	9.45730797	6.84E-05	0.000232139

SPCC663.05c	cia1	histone H3-H4 chaperone Cia1	0.28254492	6.67048	0.02700479	0.050876963
SPBP16F5.04	ubc7	Hrd1 ubiquitin ligase complex ubiquitin conjugating enzyme	0.28205319	6.40707115	0.04739223	0.082629798
SPBC1604.11	atp17	F1-FO ATP synthase subunit F (predicted)	0.28189586	6.47757332	0.04814394	0.083790148
SPCC613.07	bcd1	snoRNA biogenesis protein Bcd1 (predicted)	0.28168228	5.41416952	0.16028332	0.233760826
SPBC1215.02c	naa25	NatB N-acetyltransferase complex regulatory subunit Naa2	0.28144514	8.28220843	0.00268591	0.006523072
SPAC212.08c	SPAC212.08c	S. pombe specific GPI anchored protein family 1	0.28087373	3.79916895	0.39735861	0.49271599
SPNCRNA.1456	#N/A	#N/A	0.2807783	4.97278159	0.26740628	0.358702886
SPCC70.12c	ecl1	extender of the chronological lifespan protein Ecl1	0.28066458	6.6212502	0.1959831	0.276349977
SPCC18B5.05c	SPCC18B5.05c	phosphomethylpyrimidine kinase (predicted)	0.28065063	4.37377298	0.3793161	0.475017676
SPBC776.04	sec2302	COPII cargo receptor subunit Sec23b (predicted)	0.28058719	7.6506727	0.00198373	0.004984972
SPAC30C2.08	SPAC30C2.08	UPF0662 family conserved fungal protein	0.28037238	7.93465388	0.00366762	0.008591437
SPCC830.03	grc3	polynucleotide 5'-hydroxyl-kinase Grc3 (predicted)	0.28022347	7.04024426	0.02026448	0.03962638
SPAC22G7.06c	ura1	carbamoyl-phosphate synthase (glutamine hydrolyzing), as	0.28001939	10.5046632	7.84E-05	0.000262663
SPBC32F12.10	SPBC32F12.10	phosphoglucomutase (predicted)	0.28001183	9.35620468	0.00087731	0.002387086
SPAC144.18	vrg4	Golgi GDP-mannose transmembrane transporter Vrg4	0.27988156	7.39251923	0.00796239	0.01715229
SPBC16E9.10c	rix7	ribosome assembly ATPase Rix7 (predicted)	0.27938817	6.70809261	0.02138753	0.041535997
SPBC1773.06c	adh8	alcohol dehydrogenase (predicted)	0.27895158	6.77259638	0.04731091	0.082530315
SPNCRNA.1211	#N/A	#N/A	0.27866538	7.14331172	0.00629933	0.013958621
SPCC970.08	kcs1	inositol polyphosphate kinase Kcs1 (predicted)	0.27842333	7.9561891	0.00444025	0.010214981
SPCC1739.10	mug33	Tea1-interacting protein involved in exocytosis	0.27791754	7.37018631	0.02465153	0.046976659
SPBC12D12.03	cct1	chaperonin-containing T-complex alpha subunit Cct1	0.27682927	8.60534428	0.00055497	0.001584788
SPAC18G6.03	ypt3	Rab11 family GTPase Ypt3	0.27676304	7.72788188	0.00709655	0.015483175
SPCC320.09	hem15	ferrochelatase Hem15 (predicted)	0.27657078	5.84535778	0.15951216	0.232735903
SPBC146.14c	sec26	coatomer beta subunit (predicted)	0.27556718	9.23536102	0.00121324	0.003192613
SPCC70.02c	inh1	mitochondrial proton-transporting ATP synthase inhibitor In	0.27517502	6.43130837	0.05788341	0.098204092
SPAC26A3.08	smb1	Sm snRNP core protein Smb1	0.27474787	6.60992697	0.09265709	0.147320442
SPNCRNA.56	#N/A	#N/A	0.27471137	5.47496158	0.1415885	0.210879791
SPBC2F12.03c	ebs1	EST1 family nonsense-mediated mRNA decay (NMD) pathw	0.27414889	8.06312602	0.01073769	0.022483078
SPBC2A9.08c	sec22	SNARE Sec22 (predicted)	0.27388624	6.37957177	0.06803274	0.112533491
SPAC1B2.03c	elo2	fatty acid elongase Elo2 (predicted)	0.27339365	8.33720821	0.02014769	0.039443339
SPAC27D7.04	omt2	4-alpha-hydroxytetrahydrobiopterin dehydratase (predicted)	0.27290917	5.74390019	0.24360828	0.332882395
SPBC530.15c	SPBC530.15c	spermidine family transmembrane transporter (predicted)	0.27198121	8.14925768	0.00361632	0.00849449
SPAC23H3.07c	mrp2	mitochondrial ribosomal protein subunit S14 (predicted)	0.27187471	6.88092803	0.01471954	0.029838083
SPAC4G9.05	mpf1	meiotic pumilio family RNA-binding protein Mpf1 (predicte	0.27132073	7.32681463	0.01904256	0.037618266
SPBC17G9.08c	cnt5	Centaurin Cnt5	0.27075663	7.61305955	0.00473939	0.010851793
SPBC106.08c	mug2	mug2/mug135/meu2 family	0.27023772	5.55938349	0.16310694	0.237167246
SPNCRNA.1692	#N/A	#N/A	0.27023031	6.76667573	0.03170817	0.058459517
SPAC22H10.04	ppa3	protein phosphatase type 2A Ppa1	0.26999026	5.30975148	0.16702331	0.242034424
SPAC18G6.04c	shm2	serine hydroxymethyltransferase Shm2 (predicted)	0.26982885	7.93816752	0.00426926	0.009858255
SPNCRNA.1349	#N/A	#N/A	0.2696156	6.8306017	0.01787798	0.035525143
SPNCRNA.1192	#N/A	#N/A	0.26826072	3.50394356	0.46236506	0.557251355
SPBC4F6.18c	arf1	ADP-ribosylation factor, Arf family Arf1	0.26812672	9.28033659	0.00011908	0.000387716
SPBC1289.15	pfl5	cell surface glycoprotein, flocculin Pfl5	0.26792572	7.14904584	0.02650317	0.050025551
SPNCRNA.726	#N/A	#N/A	0.26788516	4.37746224	0.41581075	0.510848917
SPAC1805.14	SPAC1805.14	Schizosaccharomyces specific protein	0.26781574	6.35778415	0.06770189	0.112067952
SPCC1620.13	SPCC1620.13	phosphoglycerate mutase/6-phosphofructo-2-kinase family	0.26779427	5.28169293	0.16238276	0.236316233
SPAC105.03c	SPAC105.03c	transcription factor (predicted)	0.26750535	7.26554632	0.01821854	0.036166024

SPNCRNA.1655	#N/A	#N/A	0.26729056	6.45322535	0.04648291	0.081273434
SPNCRNA.1325	#N/A	#N/A	0.26688117	5.64051756	0.17106132	0.246468835
SPNCRNA.822	#N/A	#N/A	0.26673294	5.5768298	0.1181231	0.181042277
SPBC660.12c	egt2	Ergothioneine biosynthesis protein Egt2	0.26634875	8.21089178	0.00121375	0.003192726
SPNCRNA.1188	#N/A	#N/A	0.26578146	6.34950144	0.06258626	0.1050048
SPAC1250.07	sfc7	transcription factor TFIIIC subunit Sfc7 (predicted)	0.26555703	5.29053125	0.15637141	0.228938776
SPNCRNA.778	#N/A	#N/A	0.26519161	4.13256787	0.38879237	0.484300218
SPNCRNA.867	#N/A	#N/A	0.26502237	6.65727832	0.03899409	0.069959082
SPAC11D3.13	hsp3104	ThiJ domain protein, implicated in cellular detoxification	0.26492276	4.87320917	0.25191612	0.341799911
SPAC6F6.15	ypt5	GTPase Ypt5	0.26439357	6.79432528	0.02276738	0.043852813
SPBC4F6.11c	asn2	asparagine synthase (predicted)	0.26408663	7.28455682	0.02686838	0.050648018
SPAC1142.02c	SPAC1142.02c	TPR repeat protein, SGT2 family (predicted)	0.26406884	7.70311902	0.00639242	0.014141869
SPBC1347.05c	SPBC1347.05c	DNAJ domain protein Scj1 (predicted)	0.26393022	7.09054477	0.03867167	0.069417219
SPBC13G1.09	enp1	bystin family U3 and U14 snoRNA associated protein Enp1 (	0.26335897	6.74607778	0.02421136	0.046254444
SPCC126.15c	sec65	signal recognition particle subunit Sec65 (predicted)	0.26317072	6.47667549	0.0439887	0.077591715
SPAC11E3.06	map1	MADS-box transcription factor Map1	0.26315457	5.71376979	0.14460762	0.214338578
SPAC23A1.03	apt1	adenine phosphoribosyltransferase (APRT) Apt1	0.26260069	6.23416388	0.05672309	0.096461422
SPCC663.14c	trp663	plasma membrane TRP-like ion channel (predicted)	0.26253408	5.24311246	0.17811373	0.255225086
SPAC1952.12c	csn71	COP9/signalosome complex subunit 7a (predicted)	0.26160848	5.08585928	0.24962956	0.339542107
SPCC1259.03	rpa12	DNA-directed RNA polymerase complex I subunit Rpa12	0.26148125	5.10661176	0.2682121	0.359500359
SPAC17G8.03c	bur6	transcription regulator complex subunit Bur6 (predicted)	0.26143064	5.44865582	0.15820811	0.23137894
SPAC139.02c	oac1	mitochondrial carrier, ocaloacetate family anion (predicted)	0.26130138	7.04244758	0.02219211	0.042927032
SPNCRNA.1241	#N/A	#N/A	0.26085288	3.31455335	0.70613527	0.776668905
SPBP19A11.01	gcv3	glycine decarboxylase complex subunit H (predicted)	0.26071419	6.22797053	0.15210765	0.223803789
SPCC290.03c	nup186	nucleoporin Nup186	0.26036781	8.69164828	0.00117935	0.003111867
SPAC24H6.05	cdc25	M phase inducer tyrosine phosphatase Cdc25	0.26021384	7.27014219	0.05940826	0.100291054
SPAC4F10.05c	lip2	mitochondrial lipoate-protein ligase Lip2	0.26010703	5.25182613	0.22686831	0.312707895
SPAC4C5.01	SPAC4C5.01	pseudouridine-5'-phosphatase (predicted)	0.25948643	6.81205212	0.0349985	0.063707261
SPAC140.03	arb1	argonaute inhibitor protein 1	0.25928535	6.96259237	0.038524	0.069188657
SPBC25D12.03c	mcm7	MCM complex subunit Mcm7	0.25743062	7.46718207	0.00880555	0.018790141
SPAC1834.01	erf1	cytoplasmic translation release factor class I eRF1	0.2568986	7.94310581	0.01280101	0.026293652
SPNCRNA.1586	#N/A	#N/A	0.25672743	7.68217589	0.00451452	0.010375323
SPAC1805.08	dlc1	dynein light chain Dlc1	0.25639309	5.00962016	0.30699779	0.400444696
SPNCRNA.747	#N/A	#N/A	0.25622037	6.37125336	0.05573869	0.09513464
SPBC685.03	SPBC685.03	Schizosaccharomyces specific protein	0.25618191	5.51680884	0.14279128	0.212160406
SPAC17A5.08	erp2	COPII-coated vesicle component Erp2/3/4 (predicted)	0.25599952	7.1241313	0.02530314	0.048097171
SPAC29A4.19c	cta5	Ca2+/Mn2+ transporting P-type ATPase P5 type Cta5	0.25551994	7.93664564	0.00605516	0.013501111
SPCC622.12c	gdh1	NADP-specific glutamate dehydrogenase Gdh1 (predicted)	0.25548625	9.54176618	0.00028836	0.000875243
SPCC576.10c	rpt3	19S proteasome base subcomplex ATPase subunit Rpt3	0.25534203	7.99492202	0.00300896	0.007220022
SPAC1002.11	gaa1	GPI-anchor transamidase complex subunit Gaa1 (predicted)	0.25523671	5.63541385	0.19691627	0.277430217
SPAC1399.04c	uck2	uracil phosphoribosyltransferase Uck2	0.25510786	4.80979988	0.29003004	0.382665054
SPCC1183.01	sec15	exocyst complex subunit Sec15	0.25448716	7.45257604	0.00792058	0.017073027
SPAC6G10.04c	pre5	20S proteasome complex subunit alpha 6 Pre5	0.25444513	7.78645141	0.00441312	0.010156003
SPAC3F10.05c	mug113	GIY-YIGT nuclease superfamily protein	0.25443124	6.64810541	0.04019595	0.071789989
SPNCRNA.1478	#N/A	#N/A	0.25430609	6.91251581	0.02956814	0.055020829
SPBC19F8.06c	meu22	amino acid transmembrane transporter Meu22 (predicted)	0.25401311	7.28069126	0.02806323	0.052507634
SPCC777.06c	SPCC777.06c	hydrolase, conserved in fungi, bacteria, plants, protazoa (pr	0.25388922	7.18207977	0.01146807	0.023836353

SPAC15E1.04	hal3	thymidylate synthase / phosphopantothenoylcysteine decar	0.25363878	7.6160055	0.00607561	0.013528975
SPBC1734.04	anp1	mannosyltransferase complex subunit, Anp family (predicte	0.25327529	8.72495149	0.00170716	0.00435103
SPNCRNA.1040	#N/A	#N/A	0.25324398	6.23088247	0.09084996	0.144785481
SPCC4G3.10c	rhp42	DNA repair protein Rhp42	0.25301868	6.75258046	0.04467903	0.078462139
SPAC10F6.14c	SPAC10F6.14c	mitochondrial ABC1 kinase family protein, human ADCK5 or	0.25296355	5.31215205	0.2970023	0.38999401
SPNCRNA.1017	#N/A	#N/A	0.25286293	7.92944622	0.01558491	0.031377305
SPBC16H5.08c	SPBC16H5.08c	ribosome biogenesis ATPase, Arb family ABCF2-like (predic	0.25284677	8.71830718	0.00070707	0.001978444
SPAC18G6.05c	gcn1	translation initiation regulator Gcn1	0.25281601	9.70715562	0.00086807	0.002365721
SPAC2G11.10c	uba42	thiosulfate sulfurtransferase, URM1 activating enzyme E1-t	0.25249023	5.36282475	0.25926978	0.349782135
SPBC17G9.13c	SPBC17G9.13c	mitochondrial conserved fungal protein	0.25235523	4.48912783	0.32722854	0.421946367
SPCC23B6.04c	pdr16	meiotic sec14 cytosolic factor family, phospholipid-interme	0.25180633	8.70058364	0.00137783	0.003573217
SPNCRNA.611	#N/A	#N/A	0.25179588	7.0890424	0.02352902	0.045179168
SPAC328.03	tps1	alpha,alpha-trehalose-phosphate synthase [UDP-forming]	0.25159435	8.75375599	0.05097044	0.088101045
SPNCRNA.829	#N/A	#N/A	0.25155421	7.92941508	0.01173126	0.024323958
SPBC106.05c	tim11	F1-FO ATP synthase subunit E (predicted)	0.25121944	6.53463475	0.06170792	0.103633373
SPBC725.14	arg6	acetylglutamate synthase Arg6	0.25121371	7.24535957	0.02022558	0.039561684
SPAC23C4.09c	SPAC23C4.09c	DNA-binding TFAR19-related protein, human Programmed	0.25101591	7.49450594	0.03454644	0.063060217
SPAC1834.11c	sec18	secretory pathway protein Sec18 (predicted)	0.25094257	8.79613064	0.0102425	0.021545662
SPNCRNA.1405	#N/A	#N/A	0.25071296	6.02947734	0.14423209	0.213880876
SPNCRNA.640	#N/A	#N/A	0.25070618	4.98986553	0.28943619	0.382004119
SPAC1527.03	lar1	RNA-binding protein, LARP1 family Lar1 (predicted)	0.25064172	6.92699521	0.07696689	0.125131432
SPAPB2B4.07	SPAPB2B4.07	ubiquitin family protein, human UBTD1 homolog	0.25026858	6.55419716	0.05058386	0.087633184
SPAC1D4.09c	rtf2	replication termination factor Rtf2	0.24939749	5.93693201	0.0936792	0.148607071
SPCC569.08c	ade5	phosphoribosylglycinamide formyltransferase	0.24930872	6.76428321	0.05253301	0.090470049
SPAC22E12.03c	sdj1	glyoxylase III sdj1	0.24906423	7.2516439	0.04310621	0.076310551
SPAC5H10.07	SPAC5H10.07	Schizosaccharomyces pombe specific protein	0.24807111	3.16935799	0.6597174	0.737292967
SPCC1235.02	bio2	biotin synthase	0.24718663	8.35598876	0.01007352	0.021242737
SPAC3H5.04	aar2	U5 snRNP-associated protein Aar2 (predicted)	0.24648434	7.83213624	0.00597712	0.013348968
SPCC576.15c	ksg1	serine/threonine protein kinase Ksg1	0.24647659	6.53646176	0.06389233	0.106906141
SPAC607.10	spo3	sporulation protein Spo3	0.24644085	7.11097107	0.02761108	0.051832666
SPBC1773.07c	sbp1	Ran GTPase binding protein Sbp1	0.2462745	7.87207277	0.00328102	0.007809489
SPAC1039.08	SPAC1039.08	serine acetyltransferase (predicted)	0.24614624	6.81922862	0.07064284	0.116188915
SPBC2A9.12	orc6	origin recognition complex subunit Orc6	0.24585903	5.20297834	0.26964299	0.361133748
SPAC6F12.10c	ade3	phosphoribosylformylglycinamidine synthase Ade3	0.24573895	9.42230655	0.00103694	0.002770474
SPAC9.11	SPAC9.11	Schizosaccharomyces specific protein	0.24564615	6.27470755	0.11847434	0.181458001
SPNCRNA.1252	#N/A	#N/A	0.24561511	8.00909795	0.02218577	0.042926973
SPCC736.11	35642	argonaute	0.24554813	7.09357907	0.05261727	0.090556525
SPBC337.14	rpb4	DNA-directed RNA polymerase II complex subunit Rpb4	0.24439348	6.551686	0.05707455	0.096928449
SPBC17A3.03c	SPBC17A3.03c	phosphoprotein phosphatase (predicted)	0.24434908	6.53556632	0.10208835	0.159630329
SPAC15E1.07c	moa1	meiotic kinetochore protein (Meikin) Moa1	0.24430286	1.47685623	0.80202743	0.855185942
SPBC3D6.06c	prs5	ribose-phosphate pyrophosphokinase Prs5 (predicted)	0.24423008	6.89103473	0.04024258	0.071839126
SPNCRNA.999	#N/A	#N/A	0.24394771	7.02606448	0.02459071	0.046887021
SPCC622.06c	#N/A	#N/A	0.24371871	5.70822332	0.15028308	0.221454389
SPCC4B3.03c	mam301	multi-pass membrane and CBS domain protein, implicated	0.24344306	8.95855686	0.00102437	0.002741196
SPBC1271.13	mrpl8	mitochondrial ribosomal protein subunit L8 (predicted)	0.24335093	5.92119428	0.10388745	0.161885534
SPNCRNA.1180	#N/A	#N/A	0.24303805	3.92671035	0.47865995	0.573666947
SPBC17G9.04c	nup85	nucleoporin Nup85	0.24300574	7.48592428	0.01195103	0.024711862

SPAC13G7.13c	msa1	RNA-binding protein Msa1	0.24299891	6.4887357	0.11969438	0.183079395
SPAC1A6.03c	SPAC1A6.03c	lysophospholipase (predicted)	0.2429689	9.65340893	0.00367834	0.008613595
SPAC11G7.02	pub1	HECT-type ubiquitin-protein ligase E3 Pub1	0.24276804	8.27429363	0.00231009	0.005722671
SPCC1442.03	mme1	mitochondrial carrier, magnesium ion Mme1 (predicted)	0.24229135	6.75544574	0.04425036	0.077849967
SPNCRNA.1066	#N/A	#N/A	0.24217039	5.62385334	0.24512763	0.334488368
SPBC23G7.12c	rpt6	19S proteasome base subcomplex ATPase subunit Rpt6	0.24216409	7.9511455	0.02643556	0.04995667
SPCC18.10	SPCC18.10	pyridoxine-pyridoxal-pyridoxamine kinase (predicted)	0.24205778	7.23779149	0.01471481	0.029837383
SPAC29E6.09	SPAC29E6.09	Schizosaccharomyces specific protein	0.24203777	5.11900793	0.23779352	0.32585278
SPBC4F6.05c	emp46	lectin family glycoprotein receptor Emp46 (predicted)	0.24151251	5.3095413	0.21636476	0.300482083
SPBTRNAALA.07	SPBTRNAALA.07	tRNA Alanine	0.24137545	1.47697837	0.80424039	0.857142657
SPBC28E12.01c	apc13	anaphase-promoting complex TPR lobe accessory factor Ap	0.24133323	4.18515346	0.42950185	0.524920988
SPAC2F3.17c	lsm6	Lsm2-8 complex subunit Lsm6	0.24128829	6.03373992	0.13065472	0.19714088
SPAC23D3.09	arp42	SWI/SNF and RSC complex subunit Arp42	0.24110807	7.47637588	0.0192083	0.037887666
SPAC19G12.12	dlp1	decaprenyl diphosphate synthase subunit 2 Dlp1	0.2409191	6.85072914	0.02926833	0.054552444
SPBC776.07	mam33	mitochondrial Cox1 translation regulator Mam33 (predictec	0.24038014	7.17923665	0.03203476	0.058886148
SPNCRNA.1688	#N/A	#N/A	0.23970147	4.37544196	0.49377645	0.588364343
SPAC5D6.05	med18	mediator complex subunit Med18	0.23955518	5.91340685	0.18457446	0.262493035
SPAC1486.06	npt1	nicotinate phosphoribosyltransferase Npt1 (predicted)	0.23933341	7.26145758	0.0297907	0.05538954
ScpofMt36	#N/A	#N/A	0.23915308	3.90445495	0.54465606	0.635416506
SPNCRNA.963	#N/A	#N/A	0.23902201	1.47691843	0.79964616	0.853315371
SPNCRNA.1076	#N/A	#N/A	0.23895461	6.87841247	0.04105624	0.073119006
SPAC1556.04c	cdd1	cytidine deaminase Ccd1 (predicted)	0.23893512	7.23003176	0.06024521	0.101577966
SPAC23D3.08	usp108	U1 snRNP-associated protein Usp108	0.23850604	7.46698685	0.01996707	0.039177782
SPNCRNA.704	#N/A	#N/A	0.23837853	7.14173084	0.03047178	0.056470708
SPNCRNA.804	#N/A	#N/A	0.23780387	3.76329645	0.5405496	0.631610742
SPBC1685.11	rlp1	RecA family ATPase Rlp1	0.23770187	6.15145154	0.10395418	0.161915358
SPCC162.02c	SPCC162.02c	AMP-binding dehydrogenase (predicted)	0.23765347	6.80064941	0.05380349	0.092248107
SPBP23A10.14c	ell1	RNA polymerase II transcription elongation factor Ell1	0.23686657	6.61587722	0.07775191	0.126156829
SPAC607.05	rpn9	19S proteasome regulatory subunit Rpn9	0.23664589	7.99861638	0.00622272	0.013820373
SPAC2H10.02c	nas2	26S proteasome regulatory particle assembly protein Nas2	0.23661495	5.67971253	0.26056274	0.351114741
SPCC2H8.05c	dbl1	double strand break localizing Dbl1	0.23650062	6.4137123	0.11132175	0.171700929
SPBC776.09	ste13	ATP-dependent RNA helicase Ste13	0.23640983	8.91922123	0.00132344	0.003458015
SPCC1450.14c	ero12	ER protein folding oxidoreductin Ero1b	0.23585478	8.21301626	0.0106763	0.0223683
SPAC513.06c	dhd1	D-xylose 1-dehydrogenase (NADP+) (predicted)	0.23562504	9.16337908	0.00236177	0.005833693
SPNCRNA.525	#N/A	#N/A	0.23502714	3.04358266	0.66043995	0.737979284
SPAC2G11.06	vps4	AAA family ATPase Vps4	0.23497411	7.07072714	0.02719068	0.051198829
SPAC323.03c	SPAC323.03c	Schizosaccharomyces specific protein	0.23488213	5.73912727	0.1645215	0.238920322
SPAC750.08c	SPAC750.08c	NAD-dependent malic enzyme (predicted), partial	0.23441676	1.47736074	1	1
SPBC17D11.07c	rpn2	19S proteasome regulatory subunit Rpn2	0.23405957	9.6029301	0.00505246	0.011456839
SPAC8E11.04c	SPAC8E11.04c	palmitoyl-(protein) hydrolase (predicted)	0.23372268	6.50780468	0.09457754	0.149744105
SPAC3F10.13	ucp6	UBA domain protein Ucp6	0.23358661	7.66217378	0.02173757	0.042143637
SPBC29A3.16	rrs1	ribosome biogenesis protein Rrs1	0.23318368	5.66568158	0.17672007	0.253460842
SPBC1604.21c	ptr3	ubiquitin activating enzyme E1	0.2331075	9.55706137	0.00370166	0.008662248
SPAC1783.06c	atg12	autophagy associated ubiquitin-like protein modifier Atg12	0.23304724	3.78575371	0.54618357	0.636761893
SPAC959.05c	pdi4	ER membrane protein disulfide isomerase Pdi4 (predicted)	0.23293718	5.67527579	0.21992368	0.304678473
SPBC17A3.09c	aim22	lipoate-protein ligase A (predicted)	0.23252283	6.92459652	0.03076144	0.056960981
SPBC16H5.10c	prp43	ATP-dependent RNA helicase Prp43	0.23238767	7.51483453	0.01570953	0.031562838

SPNCRNA.406	#N/A	#N/A	0.2323359	3.3613744	0.67147214	0.747728342
SPAC3G9.10c	rrp41	exosome subunit Rrp41	0.23197901	6.00443872	0.14786055	0.218499678
SPAC959.03c	utp7	U3 snoRNP-associated protein Utp7 (predicted)	0.23176518	7.14211786	0.03608886	0.065436902
SPBC19G7.14c	cog5	Golgi transport complex subunit Cog5 (predicted)	0.23165547	6.68585892	0.07400032	0.120992827
SPBC83.14c	rfc5	DNA replication factor C complex subunit Rfc5	0.23154713	6.67988096	0.06658376	0.110539759
SPNCRNA.835	#N/A	#N/A	0.2311495	3.89400876	0.5090176	0.601365407
SPAC8C9.15c	tif225	translation initiation factor eIF2B epsilon subunit	0.23100129	8.28518289	0.00665114	0.014652316
SPBC19C7.12c	omh1	alpha-1,2-mannosyltransferase Omh1	0.23099349	8.70175325	0.01598664	0.032081707
SPNCRNA.924	#N/A	#N/A	0.23052232	4.95742304	0.31205148	0.405714613
SPAC1B3.16c	vht1	plasma membrane vitamin H transmembrane transporter V	0.22999106	8.18646676	0.06292976	0.105477103
SPNCRNA.1676	#N/A	#N/A	0.22981833	2.0992342	0.87133262	0.912639445
SPBC28F2.09	toa1	transcription factor TFIIA complex large subunit Toa1 (predi	0.22916948	7.60473041	0.0173305	0.034513912
SPCC4B3.08	lsg1	Lsk1 complex gamma subunit Lsg1	0.22909255	6.20558046	0.11834333	0.181298146
SPBC3H7.06c	pof9	F-box protein Pof9	0.22888954	7.86925232	0.02002365	0.039268281
SPBP26C9.03c	fet4	plasma membrane iron/zinc ion transmembrane transporte	0.22864484	5.30918329	0.30199257	0.39505179
SPCC4B3.17	cbp3	mitochondrial Cbp3-Cbp6 complex Cbp3 (predicted)	0.22842058	6.17203652	0.16984845	0.24503258
SPAC4F8.15	itr1	myo-inositol transmembrane transporter Itr1	0.22836032	7.3509206	0.0264644	0.049993506
SPBC8D2.07c	sfc9	transcription factor TFIIIC complex subunit Sfc9 (predicted)	0.22829864	7.05086486	0.0806456	0.130354706
SPAC1F5.02	pdi1	ER protein disulfide isomerase (predicted)	0.22824973	9.32505978	0.00246649	0.006055004
SPAC3F10.09	SPAC3F10.09	1-(5-phosphoribosyl)-5-[(5-phosphoribosylamino) methylide	0.22757887	7.4172856	0.03191425	0.058743976
SPNCRNA.1028	#N/A	#N/A	0.22730113	5.21537554	0.29682289	0.38999401
SPAC8C9.14	prr1	transcription factor Prr1	0.22724913	8.42026234	0.0091081	0.019350811
SPAC3G6.01	hrp3	ATP-dependent DNA helicase Hrp3	0.22678574	8.51691879	0.0097825	0.020667469
SPAC4H3.01	SPAC4H3.01	DNAJ domain protein Caj1/Djp1 type (predicted)	0.22674091	7.74269298	0.02484952	0.047287741
SPAC4G9.17c	mrp5	mitochondrial ribosomal protein subunit S5 (predicted)	0.22660598	6.37820261	0.14347373	0.213034859
SPAC1610.01	saf5	splicing factor Saf5	0.22659554	5.123926	0.28562804	0.378740998
SPBC28F2.04c	cwf7	Prp19 complex subunit Cwf7	0.22659505	6.71547739	0.06424756	0.107315822
SPNCRNA.1376	#N/A	#N/A	0.22657547	8.4994917	0.00477096	0.010913065
SPAC17H9.13c	pro2	glutamate 5-kinase Pro2	0.22644141	7.19399749	0.02650876	0.050025551
SPAC14C4.05c	man1	LEM domain nuclear inner membrane protein Man1, Sad1 i	0.22622533	7.3694219	0.07892645	0.127840824
SPBC713.10	tim16	TIM23 translocase complex subunit Tim16 (predicted)	0.22615374	5.83693286	0.15883616	0.2319983
SPAC3G9.01	nsk1	Clp1-interacting, microtubule plus-end binding Nsk1	0.22614087	4.84012907	0.48833046	0.583254585
SPBC4C3.06	syp1	F-BAR domain protein Syp1 (predicted)	0.22606756	7.41266205	0.03134029	0.057922506
SPBC14C8.14c	pol5	polymerase phi, polymerase-related transcriptional regulato	0.22534284	7.83181854	0.01498883	0.030329766
SPAC1834.05	alg9	mannosyltransferase complex subunit Alg9 (predicted)	0.22517415	7.27586576	0.03177497	0.05856682
SPAC17H9.02	mtl1	TRAMP/MTREC complex subunit, ATP-dependent RNA helic	0.22508418	7.13702987	0.04389612	0.077468482
SPAC9G1.10c	inp53	inositol polyphosphate phosphatase Inp53 (predicted)	0.22464929	8.38773631	0.01200719	0.024810147
SPAC630.14c	tup12	transcriptional corepressor Tup12	0.22456764	7.8882297	0.0109135	0.022792776
SPBC3H7.04	SPBC3H7.04	superoxide dismutase, mitochondrial ribosomal protein sub	0.22456433	6.56284015	0.12084318	0.184529725
SPBC17D11.06	spp2	DNA primase large subunit Spp2	0.2244983	6.51373418	0.12415579	0.188673552
SPNCRNA.689	#N/A	#N/A	0.22430581	5.32107306	0.32820978	0.422925119
SPCC16A11.07	coq10	mitochondrial ubiquinone binding protein Coq10	0.22421292	6.25729129	0.13499665	0.202435484
SPNCRNA.1058	#N/A	#N/A	0.22386525	2.94630309	0.7249127	0.793219318
SPBC119.02	ubc4	ubiquitin conjugating enzyme E2 for APC and SCF Ubc4/Ubc	0.2237703	8.25327817	0.00666379	0.014674599
SPBC30B4.03c	ldb1	LIM domain binding protein, transcription co-repressor	0.22361101	5.83832994	0.18967851	0.268628981
SPNCRNA.03	#N/A	#N/A	0.22351934	7.7807623	0.08884459	0.142088706
SPBC2F12.12c	cay1	cactin, spliceosome complex subunit	0.22293031	5.26929957	0.29230633	0.385045401

SPBC1861.03	mak10	NatC N-acetyltransferase complex subunit Mak10 (predicte	0.22277342	8.40313791	0.01553221	0.031289723
SPAC4D7.05	sum1	translation initiation factor eIF3i	0.22270189	8.38147574	0.00577653	0.012936598
SPAC17G8.15	wip1	CENP-W ortholog	0.22243981	3.91472699	0.5068435	0.599825561
SPAC2F3.15	lsk1	P-TEFb-associated cyclin-dependent protein kinase Lsk1	0.22236188	6.81547451	0.09405683	0.149023691
SPNCRNA.636	#N/A	#N/A	0.221593	8.01294181	0.00771636	0.016680369
SPBC530.08	SPBC530.08	membrane-tethered transcription factor (predicted)	0.22128837	8.24144605	0.02685766	0.050641835
SPCC1450.10c	nar1	CIA machinery iron hydrogenase Nar1 (predicted)	0.22128105	5.72896609	0.17401202	0.250243405
SPBC646.06c	agn2	glucan endo-1,3-alpha-glucosidase Agn2	0.22126016	6.39273609	0.10168752	0.159229072
SPCC1906.02c	cue3	CUE domain protein Cue3, human ASCC2 ortholog, activatin	0.22038469	6.94062869	0.05085429	0.087931122
SPAC732.01	vma11	V-type ATPase V0 proteolipid subunit (predicted)	0.21976655	5.97064185	0.13738882	0.205524499
SPAC16.03c	ura2	dihydroorotase Ura2	0.21976159	6.32172368	0.11123297	0.17160289
SPAC6F12.05c	tnr3	thiamine diphosphokinase Tnr3/ Nudix hydrolase fusion pro	0.2196527	8.11810032	0.01151071	0.023917681
SPBC215.11c	SPBC215.11c	aldo/keto reductase, unknown biological role	0.2196301	7.84582569	0.04427872	0.0778596
SPNCRNA.372	#N/A	#N/A	0.2195739	4.25065956	0.49044443	0.585199995
SPBC119.09c	orm1	ORMDL family protein Orm1 (predicted)	0.21951036	7.35497576	0.09598125	0.151473194
SPBC2F12.11c	rep2	MBF transcription factor activator Rep2	0.2192849	4.08748487	0.49586617	0.589757617
SPBC216.02	num1	cortical anchoring factor for dynein Mcp5/Num1	0.2192195	6.52449133	0.11962624	0.183016308
SPAC26H5.11	mug56	spore wall assembly protein Mug56 (predicted)	0.21895359	8.01319235	0.02522199	0.047969722
SPAC513.05	ams1	alpha-mannosidase Ams1 (predicted)	0.21889811	9.17470435	0.00518102	0.011713241
SPBC691.05c	ist2	anoctamin calcium-activated chloride channel OR Ca(2+)-ac	0.21850841	6.91527645	0.06809237	0.112604751
SPBC354.07c	SPBC354.07c	sterol intermembrane transfer protein (predicted)	0.21817304	7.79943042	0.01725516	0.034394071
SPBC25B2.05	mis3	rRNA processing protein Mis3	0.21786769	6.40340885	0.12059404	0.184248422
SPNCRNA.566	#N/A	#N/A	0.21732326	3.34401682	0.69344387	0.766675143
SPAC222.19	lam3	Ragulator complex subunit, human LAMTOR3 ortholog	0.21675242	4.98697999	0.42122193	0.516471209
SPCC1620.04c	fzr3	meiotic fizzy-related APC coactivator Fzr3	0.21674773	5.35352535	0.34585142	0.440733876
SPBC32F12.08c	duo1	DASH complex subunit Duo1	0.21670165	4.47772292	0.47817398	0.573286459
SPBC16E9.09c	erp5	COPII vesicle coat component Erp5/Erp6 (predicted)	0.21661208	7.34486227	0.03135422	0.057932516
SPCC63.08c	atg1	autophagy serine/threonine protein kinase Atg1	0.21660604	6.54237349	0.100049	0.157091239
SPBC14C8.13	ber1	SRR1 family protein involved in microtubule stabilization Be	0.21606021	7.88501362	0.0194234	0.038245435
SPBC3E7.06c	fnx2	vacuolar amino acid transmembrane transporter Fnx2	0.21605099	6.89619555	0.08932379	0.142720913
SPBC19C2.11c	mdm34	ERMES complex subunit Mdm34 (predicted)	0.21581521	6.99959876	0.08495107	0.136310314
SPBC359.03c	aat1	plasma membrane amino acid transmembrane transporter	0.21572882	7.74671865	0.01633636	0.032715978
SPAC17H9.12c	cyc2	mitochondrial cytochrome c-heme linkage protein Cyc2 (pre	0.21490771	6.82175961	0.1127858	0.173604927
SPAC22F8.06	pam1	20S proteasome complex subunit beta 6 Pam1	0.21489412	7.2516052	0.0331609	0.060824768
SPAC13G7.12c	eki1	choline/ethanolamine kinase Eki1 (predicted)	0.2145796	7.33932128	0.06896897	0.113890856
SPNCRNA.1590	#N/A	#N/A	0.21447891	3.62136478	0.58394382	0.670220564
SPAC926.06c	SPAC926.06c	leucine-rich repeat protein, unknown role	0.21432097	6.81079058	0.06620432	0.110017195
SPAC9E9.05	bsp1	synaptojanin to cortical actin cytoskeletal adaptor Bsp1 (pre	0.21426931	7.69314682	0.01844375	0.036570433
SPBC428.07	meu6	pleckstrin homology domain protein, implicted in meiotic cl	0.21421682	6.69417906	0.13494552	0.2024034
SPAC4F10.19c	hit1	zf-HIT family C/D snoRNP assembly protein Hit1 (predicted)	0.21377605	4.6513275	0.57359449	0.66141
SPNCRNA.983	#N/A	#N/A	0.21321803	5.15596069	0.36687792	0.462248514
SPAC11D3.18c	SPAC11D3.18c	carboxylic acid transmembrane transporter (predicted)	0.21282885	7.14543571	0.05261004	0.090556525
SPAC227.05	gim3	prefoldin subunit 4, Gim3 (predicted)	0.2126075	6.16847979	0.16544097	0.239945818
SPBC1685.15c	klp6	kinesin-8 family plus-end directed microtubule motor Klp6	0.21253897	6.76662251	0.06584654	0.1095564
SPAC17C9.11c	SPAC17C9.11c	zf-C2H2 type zinc finger protein/UBA domain protein	0.21246841	6.57496148	0.1178056	0.180596331
SPAC806.11	#N/A	#N/A	0.21242114	7.7906564	0.10430539	0.162243155
SPBC1709.12	rid1	GTPase binding protein Rid1 (predicted)	0.21240391	8.20419097	0.02512065	0.047790338

SPBC3H7.10	elp6	elongator complex subunit Elp6	0.2123648	6.58773289	0.07697092	0.125131432
SPACUNK4.15	SPACUNK4.15	2',3'-cyclic-nucleotide 3'-phosphodiesterase (predicted)	0.21194761	6.4984985	0.16649174	0.241366906
SPNCRNA.1599	#N/A	#N/A	0.21113447	7.94541713	0.03747568	0.067537606
SPAC4D7.12c	fet5	predicted GTPAse with a role in RNA polymerase localizatio	0.21097019	6.33077968	0.13312756	0.200294729
SPAC57A7.07c	SPAC57A7.07c	homocysteine methyltransferase (predicted)	0.21070049	5.78779181	0.22163718	0.306428483
SPCC18.03	SPCC18.03	shuttle craft like transcriptional repressor/ubiquitin-protein	0.2106892	6.67891299	0.09509774	0.150427731
SPBC354.06	mrps16	mitochondrial ribosomal protein subunit S16 (predicted)	0.2104874	6.55354432	0.0989514	0.155583245
SPACUNK4.07c	cta4	P-type ATPase, calcium transporting Cta4	0.20995192	8.69243893	0.00755161	0.01637625
SPNCRNA.771	#N/A	#N/A	0.20956892	6.98673912	0.09233241	0.146941537
SPBC1A4.10c	med14	mediator complex subunit Med14	0.20948729	7.33211861	0.05245848	0.090374668
SPAC8F11.08c	SPAC8F11.08c	ER membrane associated esterase/lipase (predicted)	0.20896257	7.35215794	0.04700712	0.082126679
SPBC21C3.19	rtc3	SBDS family protein Rtc3 (predicted)	0.20859784	6.62596559	0.18703117	0.26537679
SPNCRNA.1632	#N/A	#N/A	0.20844907	6.9489994	0.09584162	0.151287918
SPBC32H8.11	mei4	meiotic forkhead transcription factor Mei4	0.2083685	5.50196058	0.26273955	0.35362789
SPCC4B3.18	ppc1	phosphopantothenate-cysteine ligase Cab2 (predicted)	0.20830233	6.57336318	0.18313132	0.261204916
SPAC2F3.04c	rim1	mitochondrial single-stranded DNA binding protein Rim1	0.20828027	5.63962151	0.21291203	0.296412924
SPNCRNA.1568	#N/A	#N/A	0.20818045	6.03713651	0.19148402	0.270791507
SPBC28F2.11	hmo1	HMG box protein Hmo1	0.20814379	7.74986963	0.02028081	0.039646918
SPCC4G3.18	rix1	Rix1 complex Armadillo-type fold Rix1	0.2079161	7.58410388	0.04280836	0.075921528
SPBC32H8.12c	act1	actin Act1	0.20763366	9.95757096	0.00389112	0.009058858
SPAC6F6.16c	tpz1	shelterin complex subunit Tpz1	0.2075249	7.48793509	0.03971055	0.071057134
SPAC3H1.09c	avt3	vacuolar amino acid transmembrane transporter Avt3	0.20724205	7.64297818	0.03465627	0.063209847
SPBC354.01	gtp1	cytoplasmic translation associated GTP binding protein Gtp	0.20708973	6.58717446	0.13445318	0.201845725
SPNCRNA.578	#N/A	#N/A	0.20683516	7.2291775	0.12866631	0.194399257
SPBC2G5.06c	hmt2	sulfide-quinone oxidoreductase	0.20679703	8.03280158	0.0472401	0.082449058
SPCC330.07c	SPCC330.07c	transmembrane transporter (predicted)	0.20644531	7.22197397	0.03625521	0.065668533
SPAC25B8.19c	loz1	transcription factor zf-C2H2 type	0.20638299	6.48500276	0.19757495	0.277961042
SPAC1D4.08	pis1	CDP-diacylglycerol--inositol 3-phosphatidyltransferase Pis1	0.20633447	6.73191282	0.24019625	0.328748087
SPCC1442.05c	mic26	MICOS complex subunit Mic23/26/27 (predicted)	0.20553554	5.81336785	0.25567725	0.345901322
SPCC1919.09	tif6	translation initiation factor-like ribosome biogenesis protei	0.20547929	7.47755789	0.02950127	0.054941479
SPCC1281.02c	spf30	splicing factor Spf30 (predicted)	0.20536522	5.09769089	0.35988028	0.455694705
SPAC25G10.03	zip1	transcription factor Zip1	0.20519954	7.7846371	0.04621039	0.080859266
SPCC63.05	tap42	TAP42 family protein involved in TOR signalling Tap42 (prec	0.20450163	5.66955079	0.21776974	0.302124994
SPBC1105.17	cnp1	centromere-specific histone H3 CENP-A	0.20431405	5.93800767	0.20123366	0.281942568
SPBC713.05	wdr83	WD repeat protein, human MAPK organizer 1 (MORG1) fam	0.2042612	4.95098573	0.35826304	0.453924523
SPNCRNA.852	#N/A	#N/A	0.20405608	6.77627416	0.1031317	0.160855196
SPAC22H10.05c	SPAC22H10.05c	mRNA cleavage and polyadenylation specificity factor comp	0.20394647	7.55109433	0.05158654	0.089007709
SPBC2A9.10	SPBC2A9.10	Bin3 family, 7SK RNA methyltransferase (predicted)	0.20380056	5.74708725	0.23111946	0.317666714
SPAC12G12.03	cip2	RNA-binding protein Cip2	0.20325875	8.47443909	0.01563799	0.031465549
SPNCRNA.1207	#N/A	#N/A	0.2031885	1.90608024	1	1
SPBC649.04	uvi15	tail anchored plasma membrane protein Uvi15	0.20213765	7.21425124	0.10078784	0.158068973
SPCC1682.15	mug122	PX/PXA domain protein	0.20207562	7.11276441	0.10042537	0.15760946
SPAC23C11.15	pst2	Clr6 histone deacetylase complex subunit Pst2	0.20197071	7.47190258	0.06277023	0.105235624
SPAC2F7.05c	tif5	translation initiation factor eIF5, Tif5(predicted)	0.2017565	9.02483084	0.01286159	0.026410119
SPAC4G8.04	SPAC4G8.04	GTPase activating protein (predicted)	0.2012964	7.61065536	0.04971066	0.086252176
SPBC4.04c	mcm2	MCM complex subunit Mcm2	0.201284	6.9684803	0.08590209	0.137706415
SPBC14F5.11c	snx41	PX domain sorting nexin Snx41	0.20118872	7.27602939	0.06657467	0.110539759

SPNCRNA.1620	#N/A	#N/A	0.20117078	6.50830357	0.11885676	0.181920889
SPAC26A3.06	bud23	rRNA (guanine-N7-)-methyltransferase Bud23 (predicted)	0.20087032	5.59821059	0.25391877	0.344195074
SPAC186.06	SPAC186.06	phenazine biosynthesis PhzF protein family	0.20069967	1.58369085	0.81720783	0.867295586
SPAC17A5.10	hua1	conserved fungal protein, possibly DNA J domain Hua1	0.19968882	5.16542407	0.45673985	0.551866955
SPBC83.03c	tas3	RITS complex subunit 3	0.19960216	5.50590024	0.32816341	0.422925119
SPCC1322.01	rpm1	mitochondrial 3'-5' exonuclease for RNA 3' ss-tail	0.19949877	6.22975601	0.19403821	0.273948137
SPBC337.10c	dre2	CIA machinery anamorsin Dre2 (predicted)	0.19945532	7.95750625	0.03616321	0.065536796
SPBC19G7.19	tom5	mitochondrial TOM complex subunit Tom5 (predicted)	0.19876985	5.77960835	0.255149	0.345388712
SPAC29E6.05c	mxr1	peptide-methionine (S)-S-oxide reductase MsrA	0.19846131	6.69028252	0.13801426	0.206188151
SPNCRNA.1474	#N/A	#N/A	0.19841664	4.55822488	0.44535516	0.541282701
SPAC10F6.11c	atg17	autophagy associated protein kinase activator Atg17	0.19813906	7.60161935	0.06378727	0.106756604
SPAC23C4.17	trm402	tRNA (cytosine-5-)-methyltransferase (predicted)	0.19796205	7.11220116	0.06546187	0.109023012
SPBC3B8.02	php5	CCAAT-binding factor complex subunit Php5	0.19787617	7.64657625	0.06572803	0.109385979
SPNCRNA.1444	#N/A	#N/A	0.19738691	2.84291301	0.81953371	0.869086317
SPBC1709.17	met7	folylpolyglutamate synthase Met7 (predicted)	0.1971746	7.74304817	0.03850355	0.069170174
SPAC1556.03	azr1	serine/threonine protein phosphatase Azr1	0.19699651	7.65184803	0.09303071	0.147776369
SPBC2D10.03c	ess1	DUF866 family protein, human C1orf123 ortholog	0.19695871	5.54378607	0.26756168	0.358840601
SPCC553.06	swp1	oligosaccharyltransferase delta subunit Swp1 (predicted)	0.19694853	7.4509986	0.04738405	0.082629798
SPAC30D11.03	ddx27	ATP-dependent RNA helicase Ddx27/Drs1 (predicted)	0.19691066	7.13829944	0.06898723	0.113890856
SPCC4B3.16	tip41	TIP41-like type 2a phosphatase regulator Tip41	0.19649777	5.77535775	0.2767946	0.368824607
SPNCRNA.1502	#N/A	#N/A	0.19632694	3.81011862	0.59447699	0.678305821
SPAC5D6.06c	alg14	UDP-GlcNAc transferase associated protein Alg14 (predicte	0.19589693	4.96912311	0.42408586	0.519047528
SPCC645.13	bye1	transcription elongation regulator Bye1 (predicted)	0.19587321	6.93430188	0.06698819	0.111129843
SPNCRNA.749	#N/A	#N/A	0.19561847	5.7551525	0.31197159	0.405688263
SPBC23G7.05	sui1	translation initiation factor eIF1	0.19533346	7.33535818	0.06102923	0.102594847
SPAC30D11.02c	SPAC30D11.02c	Schizosaccharomyces pombe specific protein	0.19532936	1.58398852	0.81304932	0.863825232
SPCC613.03	SPCC613.03	endoplasmic reticulum EF hand protein (predicted)	0.19517939	7.34813545	0.08039455	0.130010665
SPAC3A11.02	cps3	zf-CCCH type zinc finger protein, unknown biological role Cp	0.19496009	6.80920358	0.12715659	0.192545753
SPBC106.10	pka1	cAMP-dependent protein kinase catalytic subunit Pka1	0.19488203	7.60509579	0.0614526	0.103255534
SPBC16H5.07c	ppa2	serine/threonine protein phosphatase Ppa2	0.19474573	8.70724403	0.01786707	0.03552014
SPAC17A5.04c	mde10	spore wall assembly ADAM family peptidase Mde10	0.19446736	6.19678224	0.2891651	0.381868528
SPAC1039.07c	SPAC1039.07c	aminotransferase class-III, possible transaminase, unknowr	0.19355528	8.08445491	0.02556076	0.048492037
SPAC23H3.12c	SPAC23H3.12c	mitochondrial hydrogen/potassium transport system protei	0.19336301	3.83174446	0.58272781	0.669163473
SPBC18H10.17c	SPBC18H10.17c	mitochondrial recombinase Mhr1 (predicted)	0.19327844	6.04844863	0.22321158	0.308354613
SPBC16C6.08c	qcr6	ubiquinol-cytochrome-c reductase complex subunit 8, hinge	0.19324523	7.77659795	0.04064208	0.072495251
SPBC577.03c	SPBC577.03c	N-acetyltransferase (predicted)	0.19313122	7.47479013	0.03966067	0.07098655
SPBC83.10	ecm7	ER membrane protein complex subunit Ecm7 (predicted)	0.19256949	5.24959948	0.36440645	0.459815663
SPCC1235.07	fta7	CENP-Q homolog Fta7	0.19256232	6.45622619	0.14560543	0.215588542
SPCC1840.05c	SPCC1840.05c	phosphoglucomutase (predicted)	0.19250076	8.16174603	0.02541438	0.0482547
SPNCRNA.351	#N/A	#N/A	0.19220719	2.70719798	0.8609173	0.904375152
SPBC13E7.06	msd1	microtubule-anchoring factor Msd1	0.19218293	4.14328565	0.59925698	0.68261529
SPAPB1A10.16	dpc13	mitochondrial conserved protein Dpc13 (predicted)	0.19204639	5.71761974	0.22600276	0.311704251
SPBC32F12.07c	SPBC32F12.07c	membrane associated ubiquitin-protein ligase E3, MARCH f	0.19167472	7.6596653	0.07442949	0.121636097
ScpofMt18	#N/A	#N/A	0.19153988	1.58411855	0.81420791	0.864786147
SPCC965.03	vma8	V-type ATPase V1 subunit D (predicted)	0.19146021	7.21927181	0.07046102	0.115986273
SPAC31G5.19	abo1	ATPase with bromodomain protein	0.19090071	8.39004573	0.07670213	0.124870334
SPCC330.10	pcm1	P-TEFb-cap methyltransferase Pcm1	0.19031445	5.90532336	0.3315185	0.426059191

SPCC285.03	dbp6	ATP-dependent RNA helicase Dbp6 (predicted)	0.18935041	7.7674341	0.03673011	0.066404997
SPBC1718.05	trs31	TRAPP complex subunit Trs31 (predicted)	0.1892373	5.39031501	0.33573722	0.430181096
SPAC4F10.03c	trm7	tRNA 2'-O-ribose methyltransferase Trm7 (predicted)	0.18899199	5.83207099	0.25055863	0.340397576
SPBC17G9.02c	cdc73	RNA polymerase II accessory factor, Cdc73 family (predicte	0.18893854	6.81395533	0.09561867	0.1510411
SPBC17A3.02	aim19	mitochondrial conserved fungal membrane protein Aim19	0.18861	5.2861319	0.39797726	0.493393197
SPCC16A11.17	mcm4	MCM complex subunit Mcm4/Cdc21	0.1881901	7.35268203	0.05157909	0.089007709
SPBC32F12.06	pch1	P-TEFB associated cyclin, cyclin T Pch1	0.18755755	7.71750335	0.06983014	0.115086965
SPAP27G11.07c	bud32	EKC/KEOPS complex associated ATPase Bud32 (predicted)	0.18742797	5.78899689	0.30806975	0.401458191
SPNCRNA.1397	#N/A	#N/A	0.18740308	6.54152461	0.17659905	0.253428199
SPCC1322.12c	bub1	mitotic spindle checkpoint kinase Bub1	0.18701896	6.92519337	0.1342096	0.201621676
SPAC23H4.12	alp13	MRG family Clr6 histone deacetylase complex subunit Alp1	0.18697236	7.2503718	0.07621399	0.124160418
SPAC1F7.11c	SPAC1F7.11c	transcription factor, zf-fungal binuclear cluster type (predic	0.1865681	6.90222602	0.12394492	0.188437263
SPNCRNA.1290	#N/A	#N/A	0.18649469	6.76137939	0.27778855	0.369859332
SPAC4F10.07c	atg13	autophagy associated protein kinase regulatory subunit Atg	0.18638864	6.80789469	0.12490403	0.189641218
SPCC1742.01	gsf2	cell surface glycoprotein, galactose-specific flocculin Gsf2	0.18604567	7.99477047	0.07502136	0.122397595
SPNCRNA.1132	#N/A	#N/A	0.18598806	7.25507889	0.17113702	0.246525704
SPCC24B10.22	pog1	mitochondrial DNA polymerase gamma Pog1	0.18591233	7.26653388	0.08244768	0.133014803
SPNCRNA.82	mrp1	RNAse MRP	0.18589068	7.93014426	0.05085921	0.087931122
SPAC1F3.04c	tsr3	SSU-rRNA maturation protein Tsr3 (predicted)	0.18587648	6.71695942	0.1552662	0.227614501
SPBC2A9.06c	nus1	di-trans,poly-cis-decaprenylcistransferase Nus1	0.1856162	5.79165963	0.2990688	0.391980582
SPAC23C11.14	zhf1	ER zinc ion transmembrane transporter Zhf1	0.18524397	7.40084051	0.14636449	0.21647693
SPAC1142.01	rqc1	ribosome quality control complex (RQC) complex subunit R	0.18477151	7.31045615	0.08506128	0.136454977
SPCC16C4.11	pef1	Pho85/PhoA-like cyclin-dependent kinase Pef1	0.18475222	5.8996156	0.38697699	0.482719965
SPAC2F7.03c	pom1	DYRK family cell polarity protein kinase Pom1	0.18473033	7.852596	0.02895188	0.054021811
SPCC417.09c	SPCC417.09c	transcription factor (predicted)	0.18470816	7.27227923	0.06430355	0.107380337
SPBC13E7.11	rbd1	mitochondrial rhomboid protease (predicted)	0.18444404	5.73206243	0.31028824	0.403885126
SPCC1223.01	SPCC1223.01	ubiquitin-protein ligase E3 involved in rescue of stalled ribo	0.18404687	6.32910106	0.19468544	0.274679138
SPAC23D3.02	rfc2	DNA replication factor C complex subunit Rfc2	0.18377775	5.96802771	0.29273421	0.385459805
SPBC646.08c	SPBC646.08c	sterol intermembrane transfer protein (predicted)	0.1837229	8.07587101	0.06723401	0.111483286
SPBC8D2.04	hht2	histone H3 h3.2	0.18302663	7.39389911	0.10428952	0.162243155
SPCC1322.09	SPCC1322.09	Golgi protein, associated with COP vesicles (predicted), DU	0.18277334	8.14215938	0.05187758	0.089464508
SPBC1861.09	ppk22	serine/threonine protein kinase Ppk22 (predicted)	0.18266162	6.48160122	0.21917487	0.303826636
SPCC970.06	erv29	COP II adaptor Erv29 (predicted)	0.18206426	7.30441476	0.07512318	0.12253434
SPBC21B10.03c	ath1	ataxin-2 homolog	0.18183437	9.48087682	0.01102384	0.022997315
SPAC1A6.01c	SPAC1A6.01c	human thyroid receptor interacting protein homolog, transc	0.18181229	7.54872546	0.05772557	0.097960716
SPAC57A10.10c	sla1	La protein homolog	0.18137393	7.23735174	0.11328889	0.174182314
SPBC660.11	tcg1	single-stranded telomeric binding protein Tgc1	0.18129325	7.96872572	0.07926584	0.128337857
SPAC26A3.01	sxa1	aspartic protease Sxa1	0.18091507	9.3721808	0.01175084	0.024357134
SPNCRNA.1407	#N/A	#N/A	0.18082212	7.14477575	0.09308384	0.147826266
SPBC1539.09c	trp1	anthranilate synthase component II, multifunctional enzym	0.17988777	8.71460035	0.02478245	0.047186508
SPAC637.10c	rpn10	19S proteasome regulatory subunit Rpn10	0.17984857	7.40409351	0.05582543	0.095234902
SPBC25B2.03	SPBC25B2.03	zf-C3HC4 type zinc finger	0.17962315	7.1354318	0.10724794	0.166170815
SPAC17G6.10	ssr1	SWI/SNF and RSC complex subunit Ssr1	0.17953322	7.30720055	0.10682877	0.165634495
SPNCRNA.1354	#N/A	#N/A	0.17947273	6.92217651	0.17411259	0.25033513
SPAC13G7.05	are1	acyl-coA-sterol acyltransferase Are1 (predicted)	0.17939047	5.81189299	0.28569071	0.378750295
SPBC106.04	ada1	adenosine deaminase Ada1	0.17898516	8.27544931	0.04381933	0.077431974
SPCP1E11.04c	pal1	membrane associated protein Pal1	0.17893018	7.67172222	0.07149758	0.117436893

SPNCRNA.714	#N/A	#N/A	0.17878451	6.55053022	0.14463523	0.214338578
SPAC1783.01	SPAC1783.01	methionine synthase reductase (predicted)	0.17761716	7.29040376	0.09316059	0.147913633
SPCC1840.04	pca1	metacaspase Pca1	0.177613	8.49997898	0.0227759	0.043856824
SPBC577.06c	stt4	1-phosphatidylinositol 4-kinase Stt4	0.17722063	9.10449487	0.0646112	0.107790937
SPBC16G5.09	kex1	Golgi serine carboxypeptidase (predicted)	0.17715734	6.80642225	0.13782254	0.205946941
SPBC3B8.01c	arh1	mitochondrial [2Fe-2S] cluster asssembly NADPH-ferredoxi	0.17676132	7.74781945	0.1107497	0.171051233
SPCC132.04c	gdh2	NAD-dependent glutamate dehydrogenase Gdh2	0.1753145	8.80826328	0.0587627	0.099373793
SPAC227.02c	rrp15	rRNA processing protein Rrp15 (predicted)	0.17530506	4.97050542	0.45556663	0.550644923
SPAC7D4.06c	alg3	dol-P-Man:Man(5)GlcNAc(2)-PP-Dol alpha-1,3-mannosyltra	0.17491317	5.953726	0.26821056	0.359500359
SPBC4B4.06	vps25	ESCRT II complex subunit Vps25	0.17467959	6.62885646	0.18931229	0.268221974
SPBP19A11.02c	SPBP19A11.02c	Schizosaccharomyces pombe specific protein, predicted GP	0.17453282	5.69704218	0.4590115	0.554218105
SPCC576.06c	SPCC576.06c	mitochondrial tyrosine-tRNA ligase (predicted)	0.1740415	5.95419706	0.2808085	0.373441833
SPBC1711.09c	SPBC1711.09c	SNARE associated Golgi protein (predicted)	0.17397762	5.93117825	0.28722146	0.380039285
SPBC16H5.15	SPBC16H5.15	DUF3807 family conserved fungal protein, domain associat	0.17361156	4.449456	0.53397024	0.625308467
SPAC3H5.08c	SPAC3H5.08c	WD repeat protein, human WDR44 family	0.17332844	7.5095712	0.11427621	0.175581307
SPAC23G3.11	rpn6	19S proteasome regulatory subunit Rpn6	0.17320497	8.36776747	0.0258073	0.048891616
SPCC1902.01	gaf1	transcription factor Gaf1	0.17304235	8.54051911	0.07979299	0.129099208
SPAC1B3.05	not3	CCR4-Not complex NOT box subunit Not3/5	0.1729601	7.71050282	0.08116821	0.131074915
SPCC1672.09	SPCC1672.09	triglyceride lipase-cholesterol esterase (predicted)	0.17267744	6.5458757	0.31128467	0.405027185
SPAC227.12	rna4	U4/U6 x U5 tri-snRNP complex WD repeat subunit Rna4	0.1723022	6.16308796	0.27544199	0.367474597
SPAC9.03c	brr2	U5 snRNP complex subunit Brr2	0.17225873	9.31033556	0.01073145	0.022476926
SPBP23A10.05	ssr4	SWI/SNF and RSC complex subunit Ssr4	0.17162185	7.34609002	0.11042239	0.170623156
SPAC12G12.15	sif3	mitochondrial protein, involved in mitochondrial gene expre	0.17154052	6.3298119	0.26016042	0.350711507
SPAC11D3.07c	toe4	transcription factor, zf-fungal binuclear cluster type(predict	0.17145543	7.75109226	0.10700378	0.165868039
SPAC23C4.15	rpb5	DNA-directed RNA polymerase I, II and III subunit Rpb5	0.17126776	6.72327175	0.15026829	0.221454389
SPAC22F8.04	pet1	Golgi phosphoenolpyruvate transmembrane transporter Pet	0.17114902	8.17935683	0.03414313	0.062407743
SPCC290.04	ams2	cell cycle regulated GATA-type transcription factor Ams2	0.17083873	8.0821011	0.10617077	0.164651803
SPCC825.01	SPCC825.01	ATPase, involved in cytoplasmic translational initiation (pre	0.17070211	8.40673186	0.031801	0.058583061
SPBC1289.04c	pob1	Boi family protein	0.17068628	8.59809057	0.05896037	0.09965854
SPAC3F10.07c	erf4	palmitoyltransferase complex subunit Erf4	0.17036911	4.89466836	0.44458575	0.540540647
SPCC622.16c	epe1	Jmjc domain chromatin associated protein Epe1	0.16999845	7.62146541	0.07142177	0.117340685
SPAC17A5.12	ucp7	UBA/TPR/DNAJ domain protein Ucp7	0.16958131	6.89550931	0.13886615	0.207233365
SPAC19E9.02	fin1	serine/threonine protein kinase, NIMA related Fin1	0.16939897	7.5626768	0.1051985	0.163468956
SPAC5H10.13c	gmh2	alpha-1,2-galactosyltransferase Gmh2	0.16939317	7.91230106	0.05706138	0.096928449
SPNCRNA.922	#N/A	#N/A	0.16918429	6.53487752	0.17300697	0.249008553
SPAC664.02c	arp8	Ino80 complex actin-like protein Arp8	0.16916531	6.72296376	0.16196543	0.23586021
SPAC15E1.05c	ect1	ethanolamine-phosphate cytidylyltransferase (predicted)	0.16913817	6.91921767	0.1336845	0.200999344
SPBC1539.01c	mrp15	mitochondrial ribosomal protein subunit L15 Mrp15 (predic	0.16871953	6.22935283	0.30603679	0.399650812
SPCC1322.06	kap113	karyopherin/importin beta family nuclear import signal rec	0.16791094	6.63900695	0.20928064	0.291894807
SPBC800.07c	tsf1	mitochondrial translation elongation factor EF-Ts Tsf1	0.16764308	7.51068378	0.10202124	0.159562063
SPBC12D12.02c	cdm1	DNA polymerase delta subunit Cdm1	0.16756014	6.33462469	0.33748796	0.432017606
SPAC8C9.12c	mrs3	mitochondrial carrier, iron ion Mrs3 (predicted)	0.16688864	6.22909155	0.30916102	0.4025719
SPAPYUG7.05	SPAPYUG7.05	delta-1-pyrroline-5-carboxylate reductase (predicted)	0.1668482	7.26211986	0.10275437	0.16040308
SPAC806.02c	SPAC806.02c	CIA machinery CIA1/CFD1 fusion protein (predicted)	0.16684184	6.48599921	0.24457061	0.333795222
SPBC800.13	cnp20	histone H4 variant, CENP-T ortholog	0.16666272	4.3159825	0.57584254	0.66360855
SPAC6G9.01c	SPAC6G9.01c	DUF1764 family conserved eukaryotic protein, fungi, plants,	0.1663463	5.78474157	0.35025013	0.44558836
SPBC1D7.05	byr2	MAP kinase kinase kinase Byr2	0.16624433	6.16992505	0.30506543	0.398611798

SPNCRNA.1413	#N/A	#N/A	0.16595646	7.35236604	0.1187157	0.18174586
SPBC17A3.01c	tim50	TIM23 translocase complex subunit Tim50 (predicted)	0.16574958	7.21550256	0.10831102	0.167627129
SPBC8E4.01c	pho84	inorganic phosphate transmembrane transporter (predictec	0.16569784	10.0346376	0.01341735	0.027443666
SPCC584.14	mug160	Armadillo repeat protein, human ATXN10 ortholog	0.16497183	5.29770668	0.38183082	0.477463932
SPBC15D4.03	slm9	histone H3.3 H4 chaperone, hira family Slm9	0.16486433	6.65774598	0.21578884	0.299865843
SPAC24C9.09	SPAC24C9.09	mitochondrial threonine-tRNA ligase (predicted)	0.16474945	6.9721912	0.22474894	0.310163568
SPAC25H1.05	meu29	calcium transport regulatory factor (predicted)	0.16457326	5.9809989	0.31177431	0.405509203
SPBC1709.15c	cft2	cleavage factor two Cft2/polyadenylation factor CPSF-73 (p	0.16432194	7.35140456	0.12261008	0.186658069
SPAC22H10.08	SPAC22H10.08	DUF2009 family protein, conserved in yeast and apicomplex	0.1640109	6.37059125	0.22743854	0.313430385
SPCC613.01	SPCC613.01	transmembrane transporter (predicted)	0.16392416	6.48317988	0.23789412	0.325925
SPAC227.07c	pab1	protein phosphatase PP2A regulatory subunit B-55 Pab1	0.16392341	7.7119888	0.08453617	0.135772623
SPAC1486.01	sod2	mitochondrial superoxide dismutase Sod2	0.1639188	7.80860724	0.06867401	0.113511456
SPNCRNA.742	#N/A	#N/A	0.16390569	6.31582601	0.28430744	0.377210394
SPAC17A5.02c	dbr1	RNA lariat debranching enzyme Dbr1	0.16384709	6.12143264	0.36644504	0.461788608
SPAC1F12.02c	tma19	translationally controlled tumor protein homolog Tma19/Tp	0.16373712	8.29458145	0.03623733	0.065653634
SPAC9E9.15	SPAC9E9.15	mitochondrial protein complex assembly protein (predicted	0.16293595	5.78710911	0.37996155	0.475527853
SPBC17D11.01	nep1	NEDD8 protease Nep1	0.16291352	6.87044452	0.1991955	0.279951542
SPNCRNA.705	#N/A	#N/A	0.1629085	5.20612464	0.41379491	0.508831652
SPBC25D12.02c	dnt1	nucleolar protein Dnt1	0.16268524	7.19370361	0.10885172	0.16842563
SPAC13G7.10	teb1	transcription factor Teb1	0.16260098	6.4970241	0.26423885	0.355331135
SPAC9G1.09	sid1	PAK-related GC kinase Sid1	0.16197177	8.5057166	0.05811055	0.098491227
SPCC1672.10	mis16	kinetochore protein Mis16	0.16189789	6.81426716	0.15521093	0.227582498
SPAC4C5.02c	ryh1	GTPase Ryh1	0.16127389	7.67779073	0.13421693	0.201621676
SPNCRNA.1464	#N/A	#N/A	0.16125458	5.29839975	0.44176825	0.537787651
SPBC106.14c	sda1	SDA1 family protein (predicted)	0.16092499	7.01835162	0.20052727	0.28124239
SPNCRNA.905	#N/A	#N/A	0.16092471	6.41002439	0.27188358	0.363438678
SPNCRNA.761	#N/A	#N/A	0.16083685	6.92024853	0.16215084	0.236079692
SPBC725.16	res1	MBF transcription factor complex subunit Res1	0.16075216	6.85763884	0.18502408	0.2629676
SPAC26A3.12c	dhp1	5'-3' exoribonuclease Dhp1	0.16010746	8.76690742	0.04498938	0.078966399
SPCC320.11c	nip7	RNA-binding ribosome biogenesis protein Nip7 (predicted)	0.15978835	6.01124869	0.40696553	0.502248905
SPAC9.06c	SPAC9.06c	5'-methylthioribulose-1-phosphate dehydratase, adducin (p	0.15976323	5.70340209	0.33841422	0.432958975
SPAC3H5.06c	pol1	DNA polymerase alpha catalytic subunit	0.1593524	7.72984074	0.12150483	0.185348656
SPAC139.05	SPAC139.05	succinate-semialdehyde dehydrogenase (predicted)	0.15926824	6.32737144	0.3813433	0.476941951
SPAC26A3.16	dsk2	UBA domain protein Dph1/Dsk2	0.15875784	7.9129743	0.07469566	0.121953919
SPBC2D10.11c	nap2	histone H2A-H2B chaperone Nap2	0.15822887	7.70622752	0.13541252	0.202969643
SPAC2C4.13	vma16	V-type ATPase V0 subunit c''(predicted)	0.15789438	8.01328815	0.0632644	0.105959693
SPBC660.06	SPBC660.06	WW domain containing conserved fungal protein	0.15784166	8.60377944	0.03936524	0.070569133
SPAC11E3.15	rpl22	60S ribosomal protein L22 (predicted)	0.15774351	8.06842522	0.05055014	0.087597074
SPAC22E12.11c	set3	histone lysine methyltransferase Set3	0.15771772	8.15114588	0.07062918	0.116188915
SPBC2G2.05	rpl1603	60S ribosomal protein L13/L16 (predicted)	0.1574197	7.87190184	0.09006371	0.143700716
SPNCRNA.1051	#N/A	#N/A	0.15741863	8.74195162	0.034255	0.062578588
SPAPB24D3.02c	SPAPB24D3.02c	amino acid transmembrane transporter (predicted)	0.15731751	7.43152547	0.14918124	0.220069013
SPAC1635.01	por1	mitochondrial outer membrane voltage-dependent anion-se	0.15723223	8.44246673	0.11291303	0.173722179
SPNCRNA.1565	#N/A	#N/A	0.1571772	5.77766692	0.33472578	0.428965894
SPCC16A11.01	sfk1	plasma membrane protein involved in inositol lipid-mediate	0.15708398	7.17526448	0.18976269	0.268692281
SPNCRNA.315	#N/A	#N/A	0.15693384	3.40410974	0.73177647	0.799171801
SPAC29A4.04c	cbf5	pseudouridine synthase Cbf5 (predicted)	0.15637441	8.74052406	0.03529637	0.064153795

SPNCRNA.673	#N/A	#N/A	0.15632579	5.52641154	0.38628233	0.482055974
SPNCRNA.1073	#N/A	#N/A	0.15592787	3.00579002	0.82437159	0.873400071
SPAC3A12.12	atp11	mitochondrial F1-FO ATP synthase chaperone Atp11 (predic	0.15581239	6.35775323	0.26041687	0.350987684
SPAC343.17c	wdr70	DDB1-CUL4-associated factor (DCAF), Wdr70	0.15561311	9.28388013	0.02729817	0.051372805
SPBTRNAPHE.03	SPBTRNAPHE.03	tRNA Phenylalanine	0.15533629	1.16886479	1	1
SPBC19F5.05c	ppp1	pescadillo-family BRCT domain protein Ppp1 (predicted)	0.15527698	7.56405876	0.12085951	0.184529725
SPBC1E8.02	SPBC1E8.02	ER ubiquitin family protein (predicted)	0.15512201	6.28008797	0.29310998	0.385805301
SPCC63.10c	sec59	dolichol kinase Sec59 (predicted)	0.15500737	5.52567523	0.47495549	0.569604229
SPAC13G6.06c	gcv2	glycine cleavage complex subunit P (predicted)	0.15485332	8.81330886	0.04314904	0.076366508
SPAC26F1.02	pnn1	splicing factor, pinin ortholog Pnn1	0.15470525	4.28862041	0.71297419	0.7821682
SPBC11G11.03	mrt4	mRNA turnover and ribosome assembly protein Mrt4 (pred	0.15433229	7.10455694	0.14267043	0.212027145
SPAC10F6.06	vip1	RNA-binding protein Vip1	0.15419039	8.22599045	0.07734741	0.125648744
SPNCRNA.837	#N/A	#N/A	0.15409975	7.42828572	0.18817881	0.266782661
SPAC1556.08c	cbs2	AMP-activated protein kinase gamma subunit Cbs2	0.15381323	7.43696549	0.13766031	0.205794906
SPBC947.03c	naa38	NatC N-acetyltransferase non-catalytic Sm-like domain sub	0.15358794	8.32833116	0.08952871	0.142981187
SPBC146.04	erv2	ER thiol oxidase Erv2 (predicted)	0.15340453	5.39767849	0.42342148	0.51842086
SPBC543.04	npr3	SEA/Iml1/Npr2/3 complex subunit Npr3	0.15333729	6.68913284	0.25662774	0.346916831
SPBC15D4.10c	amo1	nuclear rim protein Amo1	0.15332073	6.40890266	0.31533686	0.409240131
SPNCRNA.1225	#N/A	#N/A	0.15323254	5.7217848	0.38485961	0.480544892
SPBC1734.05c	spf31	DNAJ protein, splicing factor Spf31 (predicted)	0.15317723	5.4618857	0.44564954	0.541491617
SPBC29A3.02c	his7	phosphoribosyl-AMP cyclohydrolase/phosphoribosyl- ATP py	0.15299533	8.18196266	0.09905583	0.155711466
SPAC26F1.12c	hgh1	Armadillo-type fold protein, human HGH1 ortholog, implica	0.15287743	6.56010136	0.26537383	0.356398045
SPBC409.19c	mtx2	metaxin 2 Mtx2 (predicted)	0.15286425	7.95512675	0.07290182	0.119570237
SPCC1223.06	tea1	cell end marker Tea1	0.15275563	7.89939674	0.12040753	0.184004765
SPNCRNA.1435	#N/A	#N/A	0.15274348	4.58943417	0.56184724	0.651229852
SPBC13E7.08c	leo1	RNA polymerase II associated Paf1 complex subunit Leo1	0.15260053	7.60500041	0.10258299	0.160219696
SPCC1494.07	trm72	tRNA 2'-O-methylase subunit Trm72 (predicted)	0.15258655	8.13353679	0.06087489	0.102385967
SPNCRNA.811	#N/A	#N/A	0.15246003	2.60958557	0.7872634	0.843142503
SPBC13E7.07	SPBC13E7.07	Schizosaccharomyces specific protein	0.1522889	7.60652155	0.10273837	0.16040308
SPBC27.02c	ask1	DASH complex subunit Ask1	0.15214195	5.06581517	0.53579905	0.626802908
SPAC607.06c	SPAC607.06c	metallopeptidase (predicted)	0.15194475	6.9929818	0.24745831	0.337127889
SPCC1682.09c	ggc1	mitochondrial carrier, guanine nucleotide Ggc1 (predicted)	0.15191456	6.20660395	0.3035962	0.396920678
SPNCRNA.674	#N/A	#N/A	0.15177617	8.05825065	0.09400567	0.148977319
SPAC1D4.13	byr1	MAP kinase kinase Byr1	0.15152842	8.05940724	0.09480553	0.150046862
SPCC126.14	prp18	U5 snRNP-associated protein Prp18 (predicted)	0.15136337	7.06621398	0.16868538	0.243975351
SPBC21H7.06c	opi10	hikeshi family nuclear import carrier Opi10	0.15100481	5.04051614	0.59917416	0.68261529
SPAC6G9.04	spo7	sporulation protein Spo7	0.15093804	6.66883161	0.26707853	0.35833387
SPNCRNA.1476	#N/A	#N/A	0.15069697	5.35447983	0.57080805	0.659208633
SPAC4G8.13c	prz1	calcineurin responsive transcription factor Prz1	0.15061133	9.10988891	0.08459978	0.135810692
SPAC24C9.02c	cyt2	cytochrome c1 heme lyase Cyt2 (predicted)	0.15028534	6.93990132	0.21223513	0.295591499
SPCC645.10	cca2	ATP(CTP) tRNA nucleotidyltransferase Cca2 (predicted)	0.15013539	6.89016865	0.21631307	0.300471614
SPNCRNA.284	#N/A	#N/A	0.14973152	2.36190802	0.881994	0.91998301
SPAC10F6.08c	nht1	Ino80 complex HMG box subunit Nht1	0.14972102	5.77398739	0.43086319	0.526367309
SPAC23A1.19c	hrq1	RecQ type DNA helicase Hrq1 (predicted)	0.14954301	7.26295793	0.13455917	0.201912939
SPAC30.04c	abc4	vacuolar phytochelatin and glutathione S-conjugate ABC far	0.14938538	9.39374615	0.04003157	0.071575103
SPAC1952.08c	SPAC1952.08c	pyridoxamine 5'-phosphate oxidase (predicted)	0.14912311	7.61582715	0.15834848	0.231484724
SPAP27G11.16	SPAP27G11.16	Schizosaccharomyces pombe specific protein	0.1487841	3.20270768	0.75979879	0.821485666

SPNCRNA.1642	#N/A	#N/A	0.14841694	4.84260707	0.54058219	0.631610742
SPBC146.13c	myo1	myosin type I	0.1484074	9.55778183	0.05620144	0.0957084
SPCC330.09	enp2	rRNA processing protein Enp2 (predicted)	0.1477513	6.9866982	0.32084476	0.415322162
SPNCRNA.731	#N/A	#N/A	0.14721509	7.6506558	0.19476514	0.274689483
SPAC23C4.18c	rad4	BRCT domain protein Rad4	0.14682885	7.5910086	0.15335634	0.225202829
SPAC11E3.10	SPAC11E3.10	Golgi multispanning membrane VanZ-like family protein	0.14672427	6.75724978	0.25544208	0.34565189
SPNCRNA.1067	#N/A	#N/A	0.14628482	4.65330554	0.59241345	0.676763897
SPAC4F10.14c	btf3	nascent polypeptide-associated complex beta subunit	0.14626242	8.15757377	0.06906226	0.11395943
SPNCRNA.80	#N/A	#N/A	0.14576706	1.16931716	1	1
SPBC2D10.08c	SPBC2D10.08c	mitochondrial ribosomal protein subunit Yml6 (predicted)	0.1454924	6.26680201	0.32651351	0.421138061
SPAC4F10.04	ypa1	protein phosphatase type 2A regulator, PTPA family Ypa1	0.14539793	5.71609157	0.38206241	0.47757801
SPNCRNA.1575	#N/A	#N/A	0.1452529	6.432148	0.33823066	0.432872562
SPBC1E8.03c	SPBC1E8.03c	ER membrane protein, conserved in fungi plants and protoz	0.14521658	5.90533017	0.45939455	0.554582212
SPAC17G6.17	pof8	LARP7 family RNA-binding protein Pof8/Lar7	0.14520342	5.38154828	0.48111052	0.576096614
SPAC1687.09	irs4	autophagy/CVT pathway ENTH/VHS domain protein Irs4 (pr	0.14510874	8.17873501	0.15313199	0.224970468
SPAC2F7.15	rsm24	mitochondrial ribosomal protein subunit S24 (predicted)	0.14506743	6.09075713	0.40428462	0.499383474
SPCC550.10	atd3	betaine-aldehyde dehydrogenase Atd3 (predicted)	0.14430698	6.28758693	0.29703732	0.38999401
SPAC23C11.11	cka1	serine/threonine protein kinase Cka1	0.14402316	8.08975207	0.08421975	0.135392246
SPAC4H3.02c	swc3	Swr1 complex subunit Swc3	0.14378567	5.70796709	0.44958657	0.545126
SPCC1739.01	SPCC1739.01	zf-CCCH type zinc finger protein	0.14352235	8.33933573	0.15842651	0.231549055
SPNCRNA.1450	#N/A	#N/A	0.14333197	5.62594702	0.50826688	0.600687064
SPNCRNA.614	#N/A	#N/A	0.14328972	5.64265194	0.48851369	0.583319118
SPAP27G11.09c	rib1	GTP cyclohydrolase Rib1 (predicted)	0.14298924	7.43053788	0.18009304	0.257516332
SPCC736.14	dis1	TOG/XMAP215 microtubule plus end tracking polymerase D	0.14267361	7.37888486	0.26630265	0.357363341
SPCC1494.10	adn3	transcription factor Adn3	0.14237267	7.28528864	0.28615026	0.379137946
SPAC3H1.02c	sdd3	mitochondrial protease Sdd3 (predicted)	0.14225131	7.74150111	0.16877266	0.243997866
SPBC1347.07	rex2	RNA exonuclease (predicted)	0.14212797	6.92809912	0.25748185	0.347927714
SPBC13A2.01c	cbc2	nuclear cap-binding complex small subunit (predicted)	0.14188247	7.82172935	0.16979554	0.24503258
SPAC31A2.04c	pre1	20S proteasome complex subunit beta 4 Pre1	0.14137291	6.41946433	0.33245575	0.426780113
SPCC188.06c	srp54	signal recognition particle subunit Srp54	0.14135127	8.05622077	0.10430769	0.162243155
ScpofMt14	#N/A	#N/A	0.14129762	2.36210963	0.88387225	0.921229937
SPAC343.10	met11	methylenetetrahydrofolate reductase Met11	0.14126941	6.27393458	0.33293328	0.427312512
SPAC4A8.06c	SPAC4A8.06c	esterase/lipase, implicated in fatty acid biosynthetic proces	0.14113019	7.43914259	0.23864596	0.326823459
SPBC25H2.12c	cct7	chaperonin-containing T-complex eta subunit Cct7	0.14089048	8.51037558	0.08312233	0.133976186
SPAC23H3.06	apl6	AP-3 adaptor complex subunit Apl6 (predicted)	0.14080165	7.90074803	0.10962479	0.169506175
SPBC2A9.11c	iss9	SAC3/GANP/THP3 family protein, human LENG8 ortholog	0.14077416	6.21846123	0.3529018	0.448382828
SPBC19C7.04c	SPBC19C7.04c	DUF2406 family conserved fungal protein	0.14045099	8.45532597	0.20789586	0.290141784
SPBC2A9.02	SPBC2A9.02	NADH-dependent glycolaldehyde/furfural/butyraldehyde/pr	0.14035199	7.75113299	0.16001512	0.233419696
SPAC26H5.06	pot1	shelterin complex subunit Pot1	0.1403257	8.30019898	0.10405447	0.162000981
SPBC32H8.03	bem46	esterase/lipase, human ABHD13 ortholog (predicted)	0.13987075	6.06122884	0.40387431	0.499066921
SPBC32H8.04c	utp24	18S rRNA endonuclease Utp24	0.13986721	6.81245048	0.27402464	0.365778274
SPBC776.17	rrp7	rRNA processing protein Rrp7 (predicted)	0.13971039	5.26094846	0.47447671	0.56915459
SPAC22G7.03	SPAC22G7.03	Schizosaccharomyces specific protein	0.13940301	4.8735107	0.62927172	0.709677498
SPBC1198.10c	slm5	mitochondrial asparagine-tRNA ligase Slm5 (predicted)	0.13906662	8.03845756	0.09096314	0.1449319
SPCC18B5.10c	tex1	TREX complex subunit Tex1 (predicted)	0.13891793	6.09508105	0.35827019	0.453924523
SPAC22E12.05c	rer1	Rer1 family protein (predicted)	0.13884673	7.42922898	0.1759686	0.252630021
SPAC2C4.11c	pno1	KH domain RNA-binding protein Pno1 (predicted)	0.13764795	5.88918936	0.4188883	0.513887665

SPAC922.05c	SPAC922.05c	transmembrane transporter (predicted)	0.1375311	7.68094083	0.14215491	0.211491942
SPAC25H1.07	emc1	ER membrane protein complex subunit Emc1 (predicted)	0.13752623	8.20118801	0.08973674	0.143246194
SPCP20C8.01c	SPCP20C8.01c	mug2/mug135/meu2 family	0.13742062	6.36591021	0.33121414	0.42574844
SPBC354.04	SPBC354.04	Schizosaccharomyces specific protein	0.13734672	5.81208396	0.39547863	0.490832043
SPCC594.06c	vsl1	vacuolar SNARE Vsl1/Vam7	0.13711499	5.52414931	0.49993816	0.593368332
SPAC2C4.15c	ubx2	UBX domain protein Ubx2	0.13675341	6.83496084	0.31851861	0.412860789
SPBC11B10.01	alg2	mannosyltransferase complex subunit Alg2 (predicted)	0.13658027	8.61472857	0.07956085	0.128784867
SPAC30C2.07	lst4	Lst4-Lst7 complex subunit Lst4	0.13657702	7.37825923	0.23319506	0.320131603
SPBC1347.10	cdc23	MCM-associated protein Mcm10	0.13636294	6.64377403	0.35085797	0.446111451
SPAC1B3.14	vma3	V-type ATPase V0 subunit c (proteolipid subunit)	0.13634069	7.42462362	0.30479375	0.398409807
SPBC17F3.02	nak1	PAK-related GC kinase Nak1	0.13600571	7.21980074	0.16813554	0.243387007
SPCC553.08c	ria1	GTPase Ria1 (predicted)	0.13556924	8.14992062	0.15151144	0.223119527
SPNCRNA.1608	#N/A	#N/A	0.13551516	6.78231129	0.27545745	0.367474597
SPNCRNA.727	#N/A	#N/A	0.135422	8.00415202	0.14900492	0.219946365
SPAC19D5.03	cid1	terminal uridylyltransferase Cid1	0.13527069	4.83842188	0.62476485	0.706233354
SPBC1A4.04	SPBC1A4.04	Schizosaccharomyces specific protein	0.13507561	5.98642206	0.4742801	0.56901906
SPBC25H2.15	trs401	SSU-rRNA maturation protein Tsr4 homolog 1 Tsr401 (pred	0.13481579	6.84373148	0.30753718	0.40084093
SPBC1539.08	arf6	ADP-ribosylation factor, Arf family Arf6	0.13480055	6.04611973	0.36598227	0.461329127
SPBC2G2.06c	apl1	AP-2 adaptor complex beta subunit Apl1 (predicted)	0.13427922	7.94952512	0.15034479	0.221497353
SPBC13G1.13	tfb2	transcription factor TFIIH complex subunit Tfb2	0.13399607	6.79974913	0.25040559	0.340325551
SPAC18G6.06	utp11	U3 snoRNP-associated protein Utp11 (predicted)	0.13386793	5.81996784	0.43702808	0.532684229
SPBC1709.16c	SPBC1709.16c	aromatic ring-opening dioxygenase (predicted)	0.13343009	5.8967686	0.39812201	0.493412525
SPBC1703.12	ubp9	ubiquitin C-terminal hydrolase Ubp9	0.13314877	7.24169617	0.1943664	0.274297666
SPAC521.03	SPAC521.03	short chain dehydrogenase, human DHRS7 family(predicted	0.13290323	7.93003436	0.11959203	0.183005111
SPAP8A3.06	uaf2	U2AF small subunit, U2AF-23	0.13281398	5.78787967	0.48525289	0.580341991
SPAC1687.15	gsk3	serine/threonine protein kinase Gsk3	0.13272384	7.25251186	0.2847583	0.377734933
SPBC12D12.01	sad1	spindle pole body SUN domain protein Sad1	0.13269446	7.69799195	0.35466213	0.450023458
SPBC839.15c	tef103	translation elongation factor EF-1 alpha Ef1a-c	0.13260714	10.2867409	0.04918849	0.08544406
SPBC13G1.11	ykt6	SNARE Ykt6 (predicted)	0.13249121	6.58189499	0.384502	0.480186472
SPNCRNA.551	#N/A	#N/A	0.13244052	5.16631378	0.54889877	0.639051516
SPNCRNA.1495	#N/A	#N/A	0.13183302	5.68475351	0.49624045	0.589860427
SPBC651.04	SPBC651.04	Schizosaccharomyces specific protein	0.13163663	6.51836143	0.31721371	0.411326091
SPNCRNA.958	#N/A	#N/A	0.1314315	4.93856823	0.57119252	0.659366424
SPAC22H10.12c	gdi1	GDP dissociation inhibitor Gdi1 (predicted)	0.13096081	8.00560507	0.22071363	0.305461923
SPBC1734.07c	trs8502	TRAPP complex subunit Trs85b (predicted)	0.13085762	7.03137941	0.23668658	0.324466591
SPAC4G8.05	ppk14	serine/threonine protein kinase Ppk14 (predicted)	0.13066044	7.09367192	0.23590916	0.323531199
SPAC57A7.10c	sec21	coatomer gamma subunit Sec21 (predicted)	0.13062445	9.27952103	0.06535088	0.108864802
SPNCRNA.1567	#N/A	#N/A	0.13050469	6.67140005	0.31998577	0.414446732
SPNCRNA.1171	#N/A	#N/A	0.13019822	5.82018719	0.43511402	0.530921801
ScpofMt32	#N/A	#N/A	0.13011873	5.18131279	0.51955154	0.610946647
SPBC1289.07c	rpc40	DNA-directed RNA polymerase I and III subunit Rpc40	0.13000985	6.76152091	0.28936407	0.381983027
SPBC29A3.06	utp18	CGI-48 family Utp18 (predicted)	0.12980833	6.59224119	0.38008704	0.475545556
SPCC1450.05c	rox3	mediator complex subunit Med19	0.12976175	5.72185795	0.46232527	0.557251355
SPNCRNA.1377	#N/A	#N/A	0.12952083	6.8317281	0.27666377	0.368722476
SPAC8E11.01c	suc2	sucrose alpha-glucosidase Suc2 (predicted)	0.1290895	6.16819652	0.45180462	0.547069472
SPAC823.14	ptf1	Mst2 histone acetytransferase acytyltransferase complex, p	0.12856268	7.12647592	0.32964997	0.424138411
SPCC18.15	dph7	diphthamide biosynthesis complex WD repeat protein subu	0.12838187	6.81888943	0.34893278	0.44416153

SPAC22H12.01c	mug35	Schizosaccharomyces specific protein Mug35	0.12825467	6.54055322	0.41347092	0.508525147
SPBC947.02	apl2	AP-1 adaptor complex subunit beta subunit Apl2	0.12736269	8.52417136	0.16875269	0.243997866
SPAC227.06	yip5	Rab GTPase binding Yip5 (predicted)	0.12704163	6.61347275	0.33955355	0.434090163
SPBC31A8.01c	rtn1	reticulon Rtn1	0.12670368	8.05875735	0.28711264	0.37996918
SPAC3H8.10	spo20	sec14 cytosolic factor family, phospholipid-intermembrane	0.12666336	7.87780775	0.13720627	0.205341685
SPNCRNA.695	#N/A	#N/A	0.12633802	6.33515058	0.35796569	0.453707675
SPBC56F2.08c	puf1	pumilio family RNA-binding protein Puf1 (predicted)	0.12573519	7.73150235	0.17968862	0.257102822
SPAC13G7.11	mba1	mitochondrial membrane-associated ribosome receptor Mt	0.12541903	6.55928094	0.36821282	0.463501334
SPAC24H6.13	SPAC24H6.13	DUF221 family protein implicated in Golgi to plasma meml	0.12523197	8.33834549	0.10395064	0.161915358
SPAC3F10.18c	rpl4102	60S ribosomal protein L41 (predicted)	0.1249983	6.00433878	0.43091731	0.526367309
SPBC337.13c	gtr1	Gtr1/RagA G protein Gtr1	0.12490904	6.267633	0.38724001	0.482719965
SPBC23G7.07c	cms1	U3-containing 90S preribosome complex subunit Cms1 (pre	0.12475524	4.95869733	0.61087619	0.693413258
SPNCRNA.1126	#N/A	#N/A	0.12426593	8.13324707	0.18512235	0.263052321
SPBC1709.04c	cyp3	cyclophilin family peptidyl-prolyl cis-trans isomerase Cyp3	0.12402727	5.94844117	0.50389134	0.597383369
SPAC2F7.04	med1	mediator complex subunit Med1	0.12386441	5.41514768	0.57117612	0.659366424
SPCC364.07	ser3	D-3 phosphoglycerate dehydrogenase Ser3 (predicted)	0.12382032	8.47346821	0.13640918	0.204328514
SPBP8B7.09c	los1	karyopherin/importin-beta family nuclear import receptor L	0.12376684	7.36353357	0.27857072	0.370724927
SPBC4F6.14	nop4	RNA-binding protein Nop4 (predicted)	0.12327972	7.51995508	0.21219847	0.295591499
ScpofMt35	#N/A	#N/A	0.12323192	5.40557123	0.5156063	0.606936668
SPAC3H1.05	ste24	CAAX prenyl protease (predicted)	0.1230072	7.85708497	0.34437314	0.439261337
SPCC61.04c	SPCC61.04c	Rab GTPase binding (predicted)	0.12285368	5.51831686	0.58538835	0.671425542
SPBC3B9.17	isa2	mitochondrial [4Fe-4S] cluster assembly and transfer prote	0.12256734	5.12972216	0.57167679	0.659813529
SPCC622.07	SPCC622.07	Schizosaccharomyces pombe specific protein	0.12210237	4.61248562	0.66211188	0.739361884
SPBC115.01c	rrp46	exosome subunit Rrp46	0.12117086	6.39597884	0.46455292	0.55932106
SPBC21B10.14	ymr31	mitochondrial ribosomal protein Ymr1 (predicted)	0.12091573	4.78554986	0.66616911	0.74279548
SPAC688.16	SPAC688.16	human TMEM254 ortholog	0.12058298	5.41767952	0.58212439	0.668809126
SPBC3B9.04	oms1	mitochondrial methyltransferase Oms1 (predicted)	0.11951729	5.32604778	0.53476904	0.626028443
SPCC1235.15	dga1	diacylglycerol O-acyltransferase Dga1	0.11906467	6.52325496	0.34120022	0.43587033
SPAC17G6.06	rps2401	40S ribosomal protein S24 (predicted)	0.11878858	7.90051044	0.17485222	0.251239302
SPBC1711.10c	npl4	Hrd1p ubiquitin ligase complex Npl4 (predicted)	0.1187151	6.39424819	0.45338895	0.548366356
SPBC1921.02	rad60	DNA repair protein, SUMO-related Rad60	0.11857791	6.18330223	0.46492532	0.559670411
SPAC1A6.08c	mug125	Schizosaccharomyces specific protein Mug125	0.11809992	3.65728591	0.82157972	0.870984574
SPAC9.09	met26	homocysteine methyltransferase Met26	0.1180082	10.2890509	0.09251885	0.147169412
SPCC330.13	rpc37	DNA-directed RNA polymerase III complex subunit Rpc37 (p	0.11774845	4.978925	0.62371735	0.705518045
SPNCRNA.1496	#N/A	#N/A	0.11756322	5.41959663	0.56110367	0.650811393
SPAC1D4.06c	csk1	cyclin-dependent kinase activating kinase Csk1	0.11740822	5.55251805	0.50783027	0.60048392
SPAC21E11.08	lcb2	serine palmitoyltransferase Lcb2 (predicted)	0.11733098	8.62863642	0.13978306	0.208418871
SPAC824.03c	SPAC824.03c	mitochondrial endonuclease family, related to holliday junc	0.11725501	6.10823975	0.49879011	0.592471061
SPCC965.11c	agp3	plasma membrane leucine transmembrane transporter Agp	0.11695622	8.29758215	0.14650561	0.21663857
SPCC1259.16	SPCC1259.16	Schizosaccharomyces specific protein	0.11692961	5.79050549	0.53893609	0.630039531
SPAC25B8.05	deg1	tRNA-pseudouridine synthase Deg1 (predicted)	0.11684733	6.05165301	0.48581811	0.580813813
SPCC1827.04	vms1	Cdc48p-Npl4p-Vms1p AAA ATPase complex subunit involve	0.11622004	6.76038282	0.35290793	0.448382828
SPAC890.02c	alp7	TACC protein Alp7	0.11584873	5.93564793	0.49352313	0.588265034
SPAPB2C8.01	SPAPB2C8.01	cell surface glycoprotein, adhesion molecule (predicted)	0.11552734	8.96276298	0.15158306	0.223176697
SPAC17A2.04c	cns1	HSP chaperone complex subunit Cns1 (predicted)	0.1153553	6.11326012	0.45710493	0.552210018
SPCC364.06	nap1	histone H2A-H2B chaperone Nap1	0.11481188	8.71422769	0.12110285	0.184859784
SPBC1289.08	uap1	UDP-N-acetylglucosamine diphosphorylase Uap1/Qri1(pred	0.11477997	6.09780954	0.50024228	0.593470839

SPAC19A8.14	pth2	mitochondrial aminoacyl-tRNA hydrolase Pth2 (predicted)	0.11450063	6.27684003	0.413285	0.508388363
SPAP8A3.02c	ofd2	histone H2A dioxygenase Ofd2	0.11441177	5.09719913	0.66255626	0.739736727
SPAC6F12.04	tvp15	COPI-coated vesicle associated protein (predicted)	0.11429112	7.24062127	0.31242074	0.406117126
SPAC30D11.14c	SPAC30D11.14c	KH domain RNA-binding protein, involved in splicing (predic	0.11423103	7.25897025	0.30651488	0.400180972
SPAC1851.02	slc1	1-acylglycerol-3-phosphate O-acyltransferase Slc1 (predicte	0.11397914	7.18445015	0.25046626	0.340340031
SPBC21B10.05c	pop3	WD repeat protein Pop3	0.11394479	6.67251895	0.41789533	0.51303946
SPAC1952.10c	SPAC1952.10c	conserved fungal multispanning membrane protein	0.11392311	5.00499343	0.64271443	0.722085467
SPBC19C7.01	mni1	exon-exon junction complex disassembly factor, human par	0.11368618	5.01512301	0.62538677	0.706818964
SPBPB8B6.04c	grt1	transcription factor Grt1 (predicted)	0.11355012	6.19056541	0.52305937	0.6146467
SPBC1683.05	SPBC1683.05	plasma membrane uricil/uridine transmembrane transport	0.11338519	7.44628919	0.3292364	0.423799069
SPNCRNA.841	#N/A	#N/A	0.11286071	7.11734519	0.35572185	0.451199849
SPBC2D10.09	snr1	3-hydroxyisobutyryl-CoA hydrolase snr1	0.11282059	6.39914452	0.49624555	0.589860427
SPBC29A10.12	oxs1	oxidative stress transcription coactivator Oxs1	0.11273287	6.57070632	0.46282805	0.557564862
SPNCRNA.1239	#N/A	#N/A	0.11233334	5.6481734	0.51689622	0.608034361
SPAC3A12.17c	cys12	cysteine synthase-like protein Cys12	0.1121209	5.79548925	0.51110383	0.602981181
SPBC25H2.07	tif11	translation initiation factor eIF1A	0.11205805	7.53283704	0.27578743	0.367842698
SPNCRNA.680	#N/A	#N/A	0.1120346	3.97539569	0.78403621	0.840744784
SPAC22E12.02	SPAC22E12.02	splicing factor, WW domain -binding Rbm42 (predicted)	0.1120126	7.51826226	0.3239787	0.418344409
SPAC19G12.08	scs7	ER sphingosine hydroxylase Scs7	0.11181	8.3368437	0.28699058	0.37988755
SPCC285.14	trs130	TRAPP complex subunit Trs130 (predicted)	0.11174365	8.51013621	0.17366678	0.249852527
SPAC26H5.02c	mgs1	DNA replication ATPase Mgs1 (predicted)	0.11146255	5.94448903	0.49438253	0.588777229
SPNCRNA.1321	#N/A	#N/A	0.11075741	5.22693904	0.63445988	0.714581179
SPAC664.01c	swi6	heterochromatin (HP1) family chromodomain protein Swi6	0.11054851	6.97435182	0.31345173	0.407301703
SPCC162.01c	snp27	U4/U6 x U5 tri-snRNP complex subunit (predicted)	0.10985785	3.65942929	0.8198599	0.869296763
SPCC24B10.17	emp24	COPII-coated vesicle component Emp24 (predicted)	0.10962818	7.17818904	0.34246285	0.437070465
SPAC56F8.03	tif52	translation initiation factor eIF5B Tif52 (predicted)	0.10941815	9.16677421	0.12975262	0.195866587
SPBC4B4.01c	ptk1	fumble family pantothenate kinase (predicted)	0.10907559	8.15723343	0.27766139	0.369811031
SPBC646.11	cct6	chaperonin-containing T-complex zeta subunit Cct6	0.10900245	8.3980444	0.19713565	0.277575512
SPBC25B2.06c	btb2	BTB/POZ domain protein Btb2	0.1089419	6.03958833	0.53341647	0.624875038
SPAC19A8.09	yos1	ER to Golgi transport protein Yos1 (predicted)	0.10885555	3.69231593	0.84878016	0.894525167
SPCC663.13c	naa50	NatA N-acetyltransferase subunit Naa50 (predicted)	0.10883738	5.55966079	0.59923701	0.68261529
SPCC1672.08c	tfa2	transcription factor TFIIE beta subunit, TFIIEB, Tfa2	0.1087071	6.82521089	0.4160939	0.511104513
SPNCRNA.1596	#N/A	#N/A	0.10800693	3.69296939	0.80753097	0.859762188
SPAC23G3.12c	htr12	serine protease, involved in lipid metabolism (predicted)	0.10790217	8.28390718	0.19936923	0.280080015
SPNCRNA.971	#N/A	#N/A	0.10783431	5.40593098	0.59973811	0.682934715
SPAC688.03c	SPAC688.03c	human AMMECR1 homolog	0.1077672	5.74315388	0.56125193	0.650872429
SPAC1834.02	aro1	pentafunctional aromatic polypeptide Aro1 (predicted)	0.10770321	9.92233897	0.11640253	0.178566099
SPAC29A4.05	cam2	myosin I light chain Cam2	0.1076494	7.03867246	0.39971024	0.49518081
SPAP14E8.02	tos4	chromatin binding FHA domain protein Tos4 (predicted)	0.10755377	7.80478326	0.41066043	0.505919368
SPNCRNA.1654	#N/A	#N/A	0.10747522	5.25925721	0.58972043	0.675256182
SPAC1002.02	pom34	nucleoporin Pom34	0.10728463	5.78701113	0.56870677	0.657724265
SPBC342.02	qrs1	cytoplasmic glutaminyl-tRNA ligase Qrs1 (predicted)	0.10724001	8.34847691	0.26189535	0.352631157
SPBC9B6.09c	mdl1	mitochondrial peptide-transporting ATPase	0.10706567	7.75568521	0.29004943	0.382665054
SPNCRNA.1467	#N/A	#N/A	0.10681213	7.79702705	0.29089504	0.383557598
SPAPB24D3.03	SPAPB24D3.03	agmatinase (predicted)	0.10676773	7.08025524	0.3450334	0.439938595
SPAC25B8.16	pop100	RNase P and RNase MRP subunit Pop100	0.10671696	6.75375962	0.46242445	0.557251355
SPBC215.12	cwf10	U5 snRNP GTPase subunit Cwf10	0.10643345	8.01829133	0.23941522	0.327745038

SPBC3F6.04c	nop14	U3 snoRNP protein Nop14 (predicted)	0.10605569	7.04821297	0.33628689	0.430804269
SPNCRNA.1250	#N/A	#N/A	0.10586125	6.03755751	0.56504592	0.654268757
SPBC13E7.09	vrp1	verprolin	0.10575333	7.35620139	0.32294416	0.417246063
SPAC29B12.10c	pgt1	plasma membrane glutathione transmembrane transporter	0.10569766	7.57433004	0.28643183	0.379363298
SPAPJ696.01c	vps17	retromer complex subunit Vps17	0.1056273	7.94269095	0.21963417	0.304401327
SPAC1093.01	ppr5	mitochondrial PPR repeat protein Ppr5	0.10529968	8.9159313	0.26431833	0.355331135
SPCC1393.13	SPCC1393.13	metal-dependent phosphatase involved in cellular detoxifica	0.10522493	7.76857874	0.27065899	0.362280566
SPNCRNA.291	#N/A	#N/A	0.10478048	2.98576243	0.91811858	0.947640971
SPAC10F6.17c	SPAC10F6.17c	mitochondrial pyruvate dehydrogenase (lipoamide) phospha	0.10459902	7.8874879	0.28172548	0.374441777
SPNCRNA.1513	#N/A	#N/A	0.10405162	5.13399288	0.67410736	0.749558924
SPAC12B10.14c	tea5	pseudokinase Tea5	0.10394278	7.11446789	0.36109667	0.456825221
SPAC1B3.15c	SPAC1B3.15c	transmembrane transporter (predicted)	0.10386022	6.42619521	0.48319468	0.578286979
SPCC285.16c	msh6	MutS protein homolog	0.10368945	8.40137332	0.31924517	0.413644968
SPBC4.06	SPBC4.06	acid phosphatase Fmp10 (predicted)	0.10341478	7.9145477	0.24770008	0.337210122
SPBP23A10.17	SPBP23A10.17	conserved fungal protein	0.10325099	7.12687347	0.38703802	0.482719965
SPNCRNA.1613	#N/A	#N/A	0.10296005	5.5537855	0.58770279	0.673739468
SPBC21D10.12	hob1	BAR adaptor protein Hob1	0.10245636	8.42746577	0.21356136	0.297239323
SPCC1281.06c	ole1	acyl-coA desaturase (predicted)	0.10222042	9.6175397	0.2047775	0.286259419
SPAC323.05c	mtq2	eRF1 methyltransferase Mtq2 (predicted)	0.10217793	7.60623215	0.26985352	0.361344594
SPBC29A3.15c	rsm23	mitochondrial ribosomal protein subunit S23 (predicted)	0.10187687	6.57740186	0.4951559	0.589285747
SPAC3A11.03	efm3	elongation factor EF2 methyltransferase Efm3 (predicted)	0.10167311	7.13576783	0.35010962	0.445492889
SPAC2F3.01	imt1	mannosyltransferase Imt1	0.10157055	7.65672062	0.25467661	0.344959063
SPCC70.10	SPCC70.10	Schizosaccharomyces specific protein	0.10121477	5.66355027	0.60521808	0.688368549
SPAC26H5.07c	SPAC26H5.07c	seven transmembrane receptor protein (predicted)	0.1007401	8.65258375	0.29382631	0.386523883
SPAC24B11.10c	cfh1	SEL1/TPR repeat protein Cfh1 (predicted)	0.10061264	7.3994523	0.33375533	0.427987749
SPNCRNA.1334	#N/A	#N/A	0.10060321	4.83692906	0.71032832	0.780020045
SPCC24B10.06	SPCC24B10.06	Schizosaccharomyces specific protein, predicted GPI anchor	0.10060121	6.23724405	0.47501896	0.569604229
SPBC405.02c	SPBC405.02c	Schizosaccharomyces specific protein	0.10045872	5.28530342	0.68798954	0.762503061
SPAC16E8.07c	vph1	V-type ATPase V0 subunit a (predicted)	0.10034632	8.9460322	0.16328927	0.237381645
SPAC3A11.13	gim1	prefoldin subunit 6, Gim1 (predicted)	0.10031785	5.58863099	0.60817713	0.691387468
SPAC17C9.13c	cut8	tethering factor for nuclear proteasome Cut8	0.10031051	7.79761279	0.26580809	0.356910817
SPNCRNA.1489	#N/A	#N/A	0.10029719	6.06989937	0.51585144	0.607120214
SPCC70.06	sac32	nuclear export factor Sac32 (predicted)	0.10013135	6.83652169	0.47042999	0.565197044
SPBC29A10.17	lam1	Ragulator complex subunit, human LAMTOR1 ortholog	0.0999392	7.42486128	0.38592618	0.481699864
SPBC6B1.12c	sus1	SAGA complex subunit Sus1	0.09959862	4.77405145	0.72721789	0.795358042
SPAC1142.04	noc201	Noc complex subunit Noc201 (predicted)	0.09942387	7.66541262	0.27132556	0.362958607
SPBC1105.18c	pth3	mitochondrial aminoacyl-tRNA hydrolase Pth3 (predicted)	0.09929105	5.27584784	0.62885978	0.709670528
SPCC550.11	nmd5	karyopherin/importin beta family nuclear import/export sig	0.09905844	8.5280229	0.19371376	0.273546824
SPAC11G7.06c	mug132	UPF0300 family protein 3	0.09903465	5.46177664	0.57976258	0.666771064
SPAC167.04	pam17	TIM23 translocase complex-associated motor subunit Pam1	0.09895583	6.63014943	0.41849736	0.513593244
SPAC31G5.21	SPAC31G5.21	DUF1754 family, human FAM32A homolog, implicated in m	0.09888195	4.86430556	0.69032863	0.764472955
SPAC31G5.05c	SPAC31G5.05c	ribulose phosphate 3-epimerase (predicted)	0.09871113	8.06120078	0.22827835	0.314460354
SPAC29A4.16	hal4	serine/threonine protein kinase Hal4	0.09837122	8.85254673	0.20536095	0.286957135
SPAC14C4.14	atp1	F1-FO ATP synthase alpha subunit	0.09834464	9.55557456	0.17906291	0.256369264
SPBC36.12c	git7	SGT1-like protein Git7	0.0983358	5.55311512	0.58517516	0.67140734
SPNCRNA.764	#N/A	#N/A	0.09830412	5.80266423	0.70234801	0.773503511
SPBC36.07	elp1	elongator complex WD repeat protein Elp1	0.09830363	8.22545369	0.27818177	0.370310433

SPNCRNA.576	#N/A	#N/A	0.09807588	8.36231921	0.30337324	0.396705397
SPAC17A2.08c	ntr2	spliceosome complex disassembly protein Ntr2 (predicted)	0.09765287	5.8454494	0.55710718	0.646947844
SPNCRNA.1383	#N/A	#N/A	0.0976108	6.32606789	0.54841418	0.638596593
SPBP18G5.03	toc1	Tor complex Tor2 interacting protein 1	0.09761041	6.77984258	0.40435018	0.499383474
SPCC1919.07	SPCC1919.07	Schizosaccharomyces specific protein	0.097572	5.91274208	0.54278449	0.633602954
SPBC651.09c	prf1	RNA polymerase II associated Paf1 complex (predicted)	0.09751298	7.87422552	0.30713375	0.400545259
SPAC1B9.03c	SPAC1B9.03c	RNA-binding protein involved in ribosomal large subunit ass	0.09748288	6.69203762	0.42446672	0.519420254
SPAC2G11.11c	prh1	ATP-dependent RNA helicase Prh1 (predicted)	0.09701312	7.02799415	0.38585416	0.481698318
SPNCRNA.324	#N/A	#N/A	0.09658437	1.30013051	1	1
SPAC212.03	SPAC212.03	hypothetical protein	0.09654919	1.29967241	1	1
SPNCRNA.1680	#N/A	#N/A	0.09653224	5.00438988	0.67252718	0.748413316
SPAC926.07c	dlc2	dynein light chain Dlc2 (predicted)	0.09623316	5.70095035	0.65426913	0.732217772
SPAC977.16c	dak2	dihydroxyacetone kinase Dak2	0.09581322	7.67398848	0.30497453	0.398569557
SPNCRNA.693	#N/A	#N/A	0.09575427	5.54658273	0.62036986	0.702665928
SPNCRNA.874	#N/A	#N/A	0.09551064	4.6224348	0.75039251	0.814421215
SPNCRNA.1129	#N/A	#N/A	0.09544994	3.86157796	0.88121888	0.919456377
SPAC31A2.13c	sft1	SNARE Sft1 (predicted)	0.09492453	5.24581524	0.66479016	0.74162247
SPBC1711.04	mtd1	methylenetetrahydrofolate reductase Mtd1 (predicted)	0.09486121	6.8527855	0.43550044	0.531202812
SPAC19E9.01c	nup40	nucleoporin Nup40	0.09432774	6.74997984	0.42484889	0.519794441
SPNCRNA.94	#N/A	#N/A	0.0940583	3.59686835	0.8661821	0.907803663
SPAC24B11.11c	sid2	NDR kinase Sid2	0.09372918	7.2882975	0.48126643	0.576181918
SPAC57A10.11c	mia40	mitochondrial Mia40-Erv1 disulfide relay system thiol oxida	0.09363051	7.29408589	0.37753487	0.473396866
SPBC577.07	ubp10	ubiquitin C-terminal hydrolase Ubp10 (predicted)	0.09362378	5.34028976	0.69253229	0.766165216
SPCC1494.06c	dbp9	ATP-dependent RNA helicase Dbp9 (predicted)	0.09324248	6.15460159	0.63265059	0.713133548
SPCC1739.02c	mrpl22	mitochondrial ribosomal protein subunit L22 (predicted)	0.09317329	5.75685435	0.58117293	0.668167225
SPBC4F6.10	vps901	guanyl-nucleotide exchange factor Vps902	0.09305405	8.32077615	0.29868811	0.391679662
SPBC146.01	med15	mediator complex subunit Med15	0.09303975	7.66763583	0.32832673	0.422995724
SPCC162.12	tco89	TORC1 subunit Tco89	0.09288206	6.78671686	0.45252164	0.547645342
SPBC887.04c	lub1	WD repeat protein Lub1	0.09206767	7.97642391	0.38937311	0.484846109
SPAC19A8.06	pbr1	ER oxidoreductase Pbr1, implicated in lipid metabolism (pr	0.09199433	6.67191594	0.47268775	0.567472603
SPNCRNA.574	#N/A	#N/A	0.09175552	6.83286463	0.45108888	0.546397268
SPNCRNA.706	#N/A	#N/A	0.09172887	6.62532931	0.55874215	0.648735767
SPBC11B10.07c	ivn1	plasma membrane phospholipid-translocating ATPase com	0.09172775	7.22964928	0.40326581	0.498633063
SPAC24B11.07c	SPAC24B11.07c	oxidoreductase (predicted)	0.09166295	6.13103524	0.63873566	0.718445649
SPNCRNA.582	#N/A	#N/A	0.09153066	3.62224523	0.87626688	0.915658598
SPAPB1A10.13	SPAPB1A10.13	Schizosaccharomyces specific protein	0.09139321	7.64385376	0.43679429	0.532494648
SPBC16E9.02c	SPBC16E9.02c	CUE domain protein, human TOLLIP ortholog	0.09118639	8.36413716	0.33046613	0.425027782
SPAC17G6.07c	use1	SNARE Use1 (predicted)	0.09106019	6.39617699	0.55051865	0.640499129
SPCC553.07c	kpa1	DinB translesion DNA repair polymerase, pol kappa	0.09101156	5.95931354	0.58148961	0.668418374
SPCC777.02	SPCC777.02	transcription factor (predicted)	0.09093886	7.27850683	0.43377868	0.529387353
SPAC2C4.09	SPAC2C4.09	mitochondrial calcium uniporter regulator (predicted)	0.09082831	6.7163863	0.53196006	0.623598311
SPNCRNA.1112	#N/A	#N/A	0.0906778	4.85124287	0.70165461	0.772990065
SPBC337.06c	cwf15	Prp19 complex subunit Cwf15	0.09023039	6.29109388	0.63527875	0.715148365
SPBC16G5.17	SPBC16G5.17	transcription factor, zf-fungal binuclear cluster type (predic	0.09014682	5.92147076	0.54755643	0.637706916
SPBC1306.02	rtt10	WD repeat protein, human WDR6 family, involved in endoc	0.0901102	7.26311952	0.4637082	0.55850164
SPAC25B8.14	mal2	CENP-O ortholog Mal2	0.09000246	6.30859686	0.54065137	0.631610742
SPNCRNA.1411	#N/A	#N/A	0.08990693	4.90104061	0.74510241	0.80997155

SPAC12B10.03	bun62	WD repeat protein Wdr20	0.08961072	6.28121056	0.60768493	0.690943345
SPBC119.03	SPBC119.03	O-methyltransferase, human COMT catechol homolog 1	0.08906251	8.41825309	0.27189806	0.363438678
SPBC17A3.05c	SPBC17A3.05c	DNAJ/DUF1977, human DNAJB12 homolog, Hsp70 co-chape	0.08864716	6.49582937	0.50750955	0.600239497
SPBC18H10.08c	ubp4	ubiquitin C-terminal hydrolase Ubp4	0.0886258	7.25296927	0.39260933	0.487893805
SPCC777.08c	bit61	Protor homolog, Bit61	0.08842826	6.50064073	0.49598572	0.589757617
SPAC1250.02	mug95	Schizosaccharomyces specific protein Mug95	0.08815856	4.04185218	0.84194139	0.888969924
SPCC417.10	dal51	dipeptide transmembrane transporter Dal5h1 (predicted)	0.08795909	6.86037635	0.48837394	0.583254585
SPAC6G9.15c	ebp1	Ell binding protein Ebp1	0.08792366	6.22339144	0.59137831	0.676260978
SPBC13G1.04c	abh1	tRNA demethylase (predicted)	0.08736763	6.20507069	0.56240987	0.651659995
SPBC660.16	gnd1	phosphogluconate dehydrogenase, decarboxylating	0.08714275	9.76517176	0.18789552	0.266436551
SPBC582.05c	brc1	BRCT domain protein Brc1	0.08673492	6.90074596	0.51399635	0.605705181
SPAC19B12.11c	bud20	zinc finger ribosome biogenesis protein Bud20 (predicted)	0.08651591	6.1987534	0.61989169	0.702323926
SPNCRNA.685	#N/A	#N/A	0.08627796	6.4539372	0.59095965	0.676220017
SPAC30D11.13	hus5	SUMO conjugating enzyme E2 Hus5	0.08623625	5.74661056	0.64201737	0.721421434
SPBC409.13	rib4	6,7-dimethyl-8-ribityllumazine synthase	0.08618641	6.61269631	0.52024898	0.611555415
SPACUNK4.14	mdb1	BRCT domain protein Mdb1	0.08603675	6.33558374	0.59174858	0.676440297
SPNCRNA.859	#N/A	#N/A	0.08557193	5.83588132	0.65028167	0.728782402
SPAC823.10c	hem25	mitochondrial carrier, glycine Hem25 (predicted)	0.08545066	4.82059146	0.79100107	0.845814307
SPBC119.12	rud3	Golgi matrix protein Rud3 (predicted)	0.08537942	6.64398099	0.54516467	0.635791871
SPAC17H9.07	srp21	signal recognition particle subunit Srp21 (predicted)	0.08494561	6.78330824	0.50080216	0.594031491
SPNCRNA.1263	#N/A	#N/A	0.08482799	7.74132879	0.3720327	0.467318228
SPAC22H10.02	SPAC22H10.02	DUF4452 family conserved fungal protein	0.08461017	5.05808013	0.78662046	0.842719174
SPBC651.11c	apm3	AP-3 adaptor complex subunit Apm3 (predicted)	0.08439836	6.98883323	0.45343975	0.548366356
SPCC1223.02	nmt1	4-amino-5-hydroxymethyl-2-methylpyrimidine phosphate sy	0.08435925	5.58816502	0.67030811	0.746676494
SPAC57A10.03	cyp1	cyclophilin family peptidyl-prolyl cis-trans isomerase Cyp1	0.0839473	5.98786948	0.58748379	0.673601889
SPNCRNA.313	#N/A	#N/A	0.08383661	1.94706239	1	1
SPCC1259.06	taf8	transcription factor TFIID complex subunit 8 (predicted)	0.08359739	5.5987016	0.67159402	0.747741705
SPAC959.04c	omh6	alpha-1,2-mannosyltransferase Omh6 (predicted)	0.08354646	6.52184599	0.55351875	0.643329645
SPBC19F5.02c	utp4	U3 snoRNP-associated WD repeat protein Utp4 (predicted)	0.08300313	7.07515541	0.49593761	0.589757617
SPAC664.14	amt2	plasma membrane ammonium transmembrane transporter	0.08297374	7.57000998	0.42631793	0.521216945
SPBC19C2.02	pmt1	tRNA (cytosine-5-)-methyltransferase Pmt1	0.08289501	5.88650238	0.6539329	0.732030493
SPNCRNA.281	#N/A	#N/A	0.08283996	1.94603187	1	1
SPBC31F10.17c	SPBC31F10.17c	Schizosaccharomyces pombe specific protein	0.08253734	6.52528346	0.55642741	0.646379061
SPNCRNA.1196	#N/A	#N/A	0.08242232	7.0362776	0.46586889	0.560707077
SPNCRNA.114	#N/A	#N/A	0.08201932	3.55719253	0.86326878	0.905983208
SPNCRNA.622	#N/A	#N/A	0.08187785	7.98661391	0.32105424	0.415356293
SPAC6G9.03c	mug183	histone H3.3 H4 heterotetramer chaperone Rtt106-like (pre	0.08150311	5.50655139	0.70771956	0.778034186
SPBC2D10.20	ubc1	ubiquitin conjugating enzyme E2 Ubc1 (predicted)	0.08147115	7.01725373	0.50692091	0.599825561
SPCC4B3.11c	fra3	mitochondrial [4Fe-4S] cluster transfer protein Fra3 (predic	0.0812157	5.12536435	0.7125677	0.781848311
SPAC1093.03	fig4	inositol polyphosphate phosphatase Fig4 (predicted)	0.08100442	7.63206646	0.48723933	0.582308334
SPBC83.08	rvb2	ASTRA/Swr1/Ino80 complex AAA family ATPase Rvb2	0.08086627	7.97211892	0.38215972	0.477611923
SPBC21H7.02	taf10	SAGA complex/transcription factor TFIID complex subunit T	0.08074079	6.51647574	0.64886703	0.727611467
SPNCRNA.1487	#N/A	#N/A	0.08073775	5.76280421	0.70452092	0.775269219
SPNCRNA.569	#N/A	#N/A	0.08054896	6.39686058	0.61309427	0.695698933
SPAC9.07c	SPAC9.07c	GTPase Obg family, involved in cytoplasmic translation Rbg	0.08046264	7.50598319	0.40394719	0.499066921
SPCP1E11.05c	are2	acyl-coA-sterol acyltransferase Are2 (predicted)	0.07969385	7.58853303	0.44859432	0.544441648
SPBP23A10.06	mtm1	mitochondrial carrier, manganese ion Mtm1 (predicted)	0.07956817	6.39181998	0.57653193	0.664049896

SPBC1683.12	SPBC1683.12	carboxylic acid transmembrane transporter (predicted)	0.07948122	8.03039542	0.38913601	0.484639564
SPAC3G6.03c	SPAC3G6.03c	Maf-like protein, nucleoside-triphosphate diphosphatase, h	0.07935503	5.53540193	0.68661461	0.761475295
SPCC737.06c	gcs2	glutamate-cysteine ligase regulatory subunit Gcs2 (predicte	0.0782652	7.14540579	0.46908783	0.563883185
SPBC4F6.06	kin1	microtubule affinity-regulating kinase Kin1	0.077844	8.52361225	0.3925128	0.487862935
SPAC2E12.02	hsf1	transcription factor Hsf1	0.07758333	7.78223507	0.46626851	0.56089044
SPBC1105.08	emp70	EMP70 family endosomal transport protein Emp70	0.07741531	7.07813667	0.46977983	0.564515579
SPAC20H4.05c	mde1	5'-methylthioribulose-1-phosphate dehydratase, adducin M	0.07729587	7.00268864	0.51057206	0.602574204
SPNCRNA.1138	#N/A	#N/A	0.07695609	7.51027142	0.42198282	0.517217779
SPCC550.13	dfp1	Hsk1-Dfp1 kinase complex regulatory subunit Dfp1	0.07684253	7.4338928	0.49452957	0.588849297
SPAC19A8.03	ymr1	phosphatidylinositol-3-phosphatase, myotubularin family (p	0.07672957	7.6522613	0.42587723	0.520865304
SPAC1B3.06c	SPAC1B3.06c	UbiE family methyltransferase (predicted)	0.07668992	7.45810897	0.42085565	0.516115103
SPBC18H10.15	cdk11	serine/threonine protein kinase Cdk11	0.07646219	5.96933969	0.62632685	0.707646392
SPAC24C9.08	cps1	vacuolar carboxypeptidase (predicted)	0.07643085	8.71223042	0.34562497	0.440527802
SPBP8B7.18c	SPBP8B7.18c	phosphomethylpyrimidine kinase (predicted)	0.07639396	6.41392829	0.59149234	0.676260978
SPAC328.06	ubp2	ubiquitin C-terminal hydrolase Ubp2	0.0761767	8.59678684	0.39200971	0.487415689
SPNCRNA.1145	#N/A	#N/A	0.0759243	5.923557	0.71065865	0.780256874
SPNCRNA.1471	#N/A	#N/A	0.07591908	8.20511457	0.37025912	0.465620502
SPBC119.11c	pac1	double-strand-specific ribonuclease Pac1	0.07586107	6.11738386	0.62779095	0.708947468
SPCC794.08	efr3	phosphatidylinositol-4 kinase plasma membrane scaffold E	0.07567335	8.20145394	0.41426589	0.509134803
SPBC12D12.06	srb11	cyclin CycC, Srb mediator subunit Srb11	0.07563783	5.82829004	0.70880136	0.778720253
SPAC23C11.13c	hpt1	guanine/xanthine/hypoxanthine phosphoribosyltransferase	0.07557366	6.39322229	0.59192934	0.676533281
SPAC652.01	SPAC652.01	BC10 family small membrane protein, unknown biological r	0.07540248	6.38588846	0.68925128	0.763523215
SPAC644.09	SPAC644.09	pyridoxal phosphate homeostasis protein (predicted)	0.07488051	6.28746055	0.62854431	0.709562716
SPBC36.08c	cog2	Golgi transport complex subunit Cog2 (predicted)	0.07481348	8.42492411	0.38020519	0.475605939
SPCC4E9.02	cig1	cyclin Cig1	0.07459023	6.75238379	0.57232647	0.660227438
SPBC691.04	mss116	mitochondrial ATP-dependent RNA helicase Mss116 (predic	0.07415969	6.95827329	0.50921519	0.601369707
SPAC1002.09c	dld1	dihydrolipoamide dehydrogenase Dld1	0.07383811	8.99384097	0.30157953	0.394587327
SPAC1782.12c	SPAC1782.12c	ER transmembrane protein, DUF423 family protein, human	0.07383475	7.44407126	0.46422059	0.559019837
SPNCRNA.909	#N/A	#N/A	0.07343695	6.35181897	0.60892298	0.691888609
SPCC16A11.10c	oca8	cytochrome b5 (predicted)	0.07326837	7.40024105	0.49248361	0.587296249
SPAC25B8.06c	dia4	mitochondrial serine-tRNA ligase (predicted)	0.07286664	7.03659139	0.58996389	0.675307703
SPCC1259.10	pgp1	mitochondrial metallopeptidase, tRNA N6-threonyl-carbam	0.07284954	6.36778235	0.62617066	0.707587403
SPNCRNA.750	#N/A	#N/A	0.07280956	5.27007052	0.78784219	0.843582702
SPAC1805.04	nup132	nucleoporin, WD repeat Nup132	0.07217103	7.91539024	0.39813787	0.493412525
SPBC32F12.12c	SPBC32F12.12c	Golgi membrane protein involved in vesicle-mediated trans	0.07206528	6.80454892	0.59816226	0.681824816
SPAC23D3.07	pup1	20S proteasome complex subunit beta 2 Pup1	0.07172057	7.73491811	0.51212655	0.603780516
SPBC21H7.05	sfc6	transcription factor TFIIIC complex subunit Sfc6	0.07140894	6.68621239	0.6099041	0.692772057
SPAC4G9.14	sym1	mitochondrial Mpv17/PMP22 family protein 2 (predicted)	0.07135668	5.75308303	0.69079583	0.764741433
SPCP31B10.04	SPCP31B10.04	DUF4448 family conserved fungal membrane protein, simil	0.07094763	6.94770849	0.58081597	0.667869666
SPAC26F1.14c	aif1	mitochondrial inner membrane anchored oxidoreductase, a	0.07074327	8.05826522	0.59242947	0.676763897
SPBC29A10.01	ccr1	NADPH-cytochrome p450 reductase	0.07063761	8.36448951	0.35846364	0.454085088
SPCC61.05	SPCC61.05	Schizosaccharomyces specific multicopy membrane protein	0.07053514	6.29528047	0.66763107	0.744181728
SPBC6B1.07	prp1	U4/U6 x U5 tri-snRNP complex subunit Prp1	0.07017037	7.44821484	0.53868928	0.629859198
SPAC12B10.15c	SPAC12B10.15c	ribonuclease H2 complex subunit (predicted)	0.06990118	5.56397728	0.74130369	0.807389818
SPAC31A2.02	trm112	eRF1 methyltransferase complex and tRNA (m2G10) methy	0.06958513	5.2464125	0.75710074	0.819740739
SPNCRNA.643	#N/A	#N/A	0.06958513	5.2464125	0.75710074	0.819740739
SPBC3E7.15c	lac1	sphingosine N-acyltransferase Lac1	0.06955286	8.26139238	0.44893663	0.54456573

SPCC1020.04c	rpb6	DNA-directed RNA polymerase I, II and III subunit Rpb6	0.06912898	6.89329889	0.57061198	0.659208633
SPCC757.13	SPCC757.13	dipeptide transmembrane transporter (predicted)	0.06912437	6.05620066	0.6410749	0.720481369
SPAC823.02	#N/A	#N/A	0.06829052	5.63275048	0.73667831	0.80337476
SPNCRNA.1695	#N/A	#N/A	0.06805962	8.04683813	0.52608381	0.617135036
SPAC1B3.13	nan1	U3 snoRNP-associated protein Nan1 (predicted)	0.06778324	7.88050498	0.49946565	0.592962969
SPAC15A10.16	bud6	nucleation-promoting factor Bud6	0.06776465	8.10955705	0.43808639	0.533687408
SPCC5E4.03c	taf5	SAGA complex subunit/TATA-binding protein associated fac	0.06775417	7.71335224	0.49670258	0.59030057
SPBC14F5.03c	kap123	karyopherin/importin beta family nuclear import signal rece	0.06772076	8.65787913	0.36575557	0.461175964
SPAC1039.10	mmf2	mitochondrial matrix protein, YjgF family protein Mmf2, rea	0.06752414	6.31809062	0.62905696	0.709670528
SPAC29B12.06c	rcd1	CCR4-Not complex RNA-binding protein subunit Rcd1	0.067293	6.26663484	0.72153897	0.789908732
SPNCRNA.758	#N/A	#N/A	0.0671013	7.50403713	0.52590661	0.617033528
SPBC29A3.10c	atp14	F1-FO ATP synthase subunit H (predicted)	0.06689799	5.76724019	0.76098201	0.822111854
SPAC1039.03	SPAC1039.03	esterase/lipase, implicated in cellular detoxification (predic	0.06677553	6.80611108	0.56997531	0.658855438
SPNCRNA.847	#N/A	#N/A	0.06668395	7.87172649	0.44872449	0.544502522
SPBC16D10.03	kae1	EKC/KEOPS complex N(6)-L-threonylcarbamoyladenine synt	0.06666106	6.66253131	0.58972018	0.675256182
SPNCRNA.1661	#N/A	#N/A	0.06648729	7.21157943	0.51532791	0.606713916
SPAC1F5.04c	cdc12	formin Cdc12	0.06643028	8.29176542	0.40341323	0.498678838
SPNCRNA.528	#N/A	#N/A	0.06590839	4.63969964	0.81881346	0.868513631
SPAC1B3.20	SPAC1B3.20	Schizosaccharomyces specific protein	0.06590257	5.58980231	0.7283216	0.796181284
SPAC2E1P3.02c	amt3	plasma membrane ammonium transmembrane transporte	0.0657648	5.88879112	0.69304333	0.766585408
SPAC26H5.05	mga2	IPT/TIG ankyrin repeat gene-specific transcription coactivat	0.06565183	8.34424207	0.45250442	0.547645342
SPCC584.03c	SPCC584.03c	GTPase regulator (predicted)	0.06561992	6.06482063	0.69064452	0.76469833
SPAC1687.17c	SPAC1687.17c	Der1-like (degradation in the ER) family (predicted)	0.0654225	6.1747045	0.70187849	0.773111541
SPCC330.21	new25	Schizosaccharomyces specific SAP domain containing prote	0.06516839	5.28500755	0.79183468	0.846439684
SPBC29A10.07	pom152	nucleoporin Pom152	0.06509108	8.96812293	0.44289844	0.538874285
SPAC6G9.12	cfr1	exomer complex BRCT domain subunit Cfr1	0.06486355	8.21227096	0.45641169	0.551568382
SPAC18B11.06	lcp5	U3 snoRNP-associated protein Lcp5 (predicted)	0.06455422	5.75634555	0.73439821	0.801536461
SPBC32H8.10	cdk9	P-TEFb-associated cyclin-dependent protein kinase Cdk9	0.06406193	7.5388833	0.51402607	0.605705181
SPBC646.12c	gap1	GTPase activating protein Gap1	0.06314769	8.79119561	0.39680359	0.492296885
SPAC1486.10	thi1	transcription factor Thi1	0.06300897	6.82581309	0.6108064	0.693413258
SPAC3G6.10c	vps51	GARP complex subunit Vps51 (predicted)	0.06270134	5.35862889	0.76273047	0.822955581
SPAC3G9.08	png1	ING family homolog Png1	0.06268928	5.11559901	0.77071838	0.829732409
SPAC19G12.07c	rsd1	RNA-binding protein Rsd1 (predicted)	0.06214529	7.504525	0.57661585	0.664049896
SPCC4G3.04c	coq5	C-methyltransferase (predicted)	0.06204048	5.39973025	0.77685098	0.834043243
SPAC17A5.09c	glc8	protein phosphatase regulatory subunit Glc8 (predicted)	0.06183417	6.51766404	0.66087203	0.738219657
SPAC26F1.01	sec74	guanyl-nucleotide exchange factor Sec74 (predicted)	0.06143096	6.6047436	0.70446115	0.775269219
SPBP8B7.12c	fta3	CENP-H ortholog Fta3	0.06127418	6.51857853	0.69688886	0.768832369
SPCC757.08	rrp45	exosome subunit Rrp45	0.06121709	6.10470924	0.71182845	0.781289133
SPBC30D10.15	SPBC30D10.15	snoRNP assembly factor Naf1 (predicted)	0.06121625	6.39730739	0.69697677	0.768832369
SPAC9E9.04	SPAC9E9.04	bcap family homolog, implicated in vesicle-mediated trans	0.06117208	7.01988292	0.67582458	0.750977505
SPAC1F3.08c	#N/A	#N/A	0.06089062	2.02449831	1	1
SPAC6F6.03c	nog2	ribosome export GTPase Nog2 (predicted)	0.06086689	7.88296191	0.48580041	0.580813813
SPAC20H4.04	fml2	ATP-dependent 3' to 5' DNA helicase (predicted)	0.06080836	7.03017814	0.61996471	0.702323926
SPNCRNA.754	#N/A	#N/A	0.06071496	6.50366575	0.64902301	0.727611467
SPAC25H1.06	pcf3	CAF assembly factor (CAF-1) complex subunit C, Pcf3	0.06069929	6.81525093	0.59088943	0.676220017
SPAC1486.05	nup189	nucleoporin Nup98 and Nup96	0.06065755	9.68893572	0.54282222	0.633602954
SPAC23H4.21	SPAC23H4.21	DUF4050 family protein, conserved in fungi and plants	0.06059067	4.86191393	0.84856875	0.894440886

SPBC1105.04c	cbp1	CENP-B homolog	0.06054597	7.05499229	0.57776422	0.665147275
SPCC569.01c	SPCC569.01c	mug2/mug135/meu2 family	0.06050496	6.42607383	0.70365664	0.774819328
SPNCRNA.1629	#N/A	#N/A	0.06047269	6.42128749	0.66314138	0.740250464
SPAC23G3.05c	SPAC23G3.05c	conserved endomembrane protein, regulator of G-protein s	0.06042539	4.20215308	0.89717743	0.931110635
SPAC29B12.08	clr5	Clr5 protein	0.06039258	8.26808156	0.55309527	0.64294727
SPAC10F6.07c	mug94	Schizosaccharomyces pombe specific protein Mug94	0.06003802	4.08883056	0.84848745	0.894440886
SPBC1198.09	ubc16	ubiquitin conjugating enzyme E2 Ubc16 (predicted)	0.06003398	7.73010347	0.50705174	0.599876056
SPNCRNA.1266	#N/A	#N/A	0.05963826	4.47044804	0.86404168	0.906258271
SPCC16C4.06c	pus3	tRNA pseudouridine synthase Pus3 (predicted)	0.05950523	6.15018412	0.73658963	0.80337476
SPAC4A8.02c	SPAC4A8.02c	conserved protein, UPF0047 family	0.0593528	7.37369865	0.58337153	0.66977051
SPBC21.07c	ppk24	serine/threonine protein kinase Ppk24	0.0593003	4.66926328	0.89248289	0.927511618
SPAC2F7.14c	rrp4	exosome subunit Rrp4	0.05929058	5.90840305	0.75455546	0.817934271
SPNCRNA.820	#N/A	#N/A	0.05907695	5.66939814	0.74693727	0.811577213
SPAC3G9.15c	fcf2	rRNA processing protein Fcf2 (predicted)	0.05894481	6.52097509	0.63383315	0.714111684
SPAP14E8.04	oma1	metallopeptidase Oma1 (predicted)	0.05882847	5.77936648	0.73888387	0.805269817
SPAC14C4.15c	dpp1	vacuolar dipeptidyl peptidase (predicted)	0.05849257	6.75979544	0.6392665	0.718805107
SPAC959.10	sen15	tRNA-splicing endonuclease subunit Sen15 (predicted)	0.05835326	5.66099471	0.73699771	0.80347155
SPAC23A1.04c	mnl1	alpha mannosidase Mnl1 (predicted)	0.0581886	7.00179679	0.62791439	0.708969206
SPACUNK4.12c	iph1	insulinase pombe homologue 1	0.05787107	7.74188868	0.5455233	0.636101105
SPBC428.19c	utp15	U3 snoRNP-associated WD repeat protein Utp15 (predicted	0.05773871	7.26077661	0.59322101	0.677099798
SPAC11H11.06	arp2	ARP2/3 actin-organizing complex subunit Arp2	0.05773314	8.61794343	0.45039061	0.545695988
SPNCRNA.1588	#N/A	#N/A	0.0575594	5.24678321	0.77084422	0.829736622
SPAC19G12.13c	poz1	shelterin complex subunit Poz1	0.05708914	7.46300033	0.57855527	0.665832677
SPBC16D10.01c	SPBC16D10.01c	TPR repeat protein, conserved fungal protein	0.05681278	7.36928882	0.65367405	0.732030493
SPNCRNA.928	#N/A	#N/A	0.05673966	8.19641955	0.49924203	0.592904362
SPCC962.02c	bir1	survivin, Bir1	0.056643	6.13662968	0.71921629	0.787619381
SPBC25H2.04c	tim22	TIM22 inner membrane protein insertion complex subunit T	0.05642676	5.6197194	0.80876778	0.860485422
SPAC823.03	ppk15	serine/threonine protein kinase Ppk15 (predicted)	0.05623502	8.11449276	0.5662104	0.655282616
SPAC17C9.06	sam50	mitochondrial sorting and assembly machinery complex sub	0.05621721	7.26135938	0.64825356	0.72734797
SPAC6G10.10c	SPAC6G10.10c	human mmtag2 ortholog, implicated in splicing	0.05616876	4.95236096	0.84458285	0.891067639
SPACUNK4.11c	mpp6	nuclear exosome-associated RNA binding protein Mpp6	0.05589222	4.86183763	0.8225578	0.871749853
SPBC1289.13c	gmh6	alpha-1,2-galactosyltransferase Gmh6	0.05577275	6.61106369	0.72756293	0.795607543
SPAC4G8.06c	trm12	tRNA 4-demethylwyosine alpha-amino-alpha-carboxypropy	0.05573966	6.32041392	0.77692875	0.834043243
SPNCRNA.1185	#N/A	#N/A	0.05570107	5.90268109	0.76877161	0.828410559
SPAC1093.04c	cca1	ATP(CTP) tRNA nucleotidyltransferase Cca1 (predicted)	0.05551196	8.0350593	0.51815251	0.609406819
SPAC16.05c	sfp1	transcription factor Sfp1 (predicted)	0.05514831	7.79437072	0.61792731	0.700599022
SPAC6G9.07c	arc4	ARP2/3 actin-organizing complex subunit Arc4	0.05477466	7.04616763	0.64565074	0.724786088
SPNCRNA.645	#N/A	#N/A	0.05469277	6.2690062	0.76023489	0.821826596
SPBC1604.03c	SPBC1604.03c	conserved fungal protein, implicated in vesicle trafficking o	0.05457779	6.00462043	0.75800188	0.820324875
SPCC737.05	SPCC737.05	peroxin Pex28/29 (predicted)	0.05425568	7.51261076	0.57212892	0.660111449
SPBC1198.13c	tfg2	transcription factor TFIIF complex beta subunit Tfg2 (predic	0.05422718	7.58250448	0.59272883	0.676934725
SPAC17D4.02	cdc45	DNA replication pre-initiation complex subunit Cdc45	0.0539501	6.42306822	0.70649211	0.77693581
SPBC23E6.09	ssn6	transcriptional corepressor Ssn6	0.05382957	8.16461447	0.61657318	0.699296753
SPNCRNA.1277	#N/A	#N/A	0.05378465	6.73116694	0.66719736	0.743820122
SPAPB1E7.01c	SPAPB1E7.01c	DUF3245 nucleolar protein, conserved in fungi and plant fa	0.05341867	6.3238615	0.77558154	0.833122714
SPBP19A11.03c	mts4	19S proteasome regulatory subunit Rpn1/Mts4	0.05331568	9.1906514	0.48239242	0.577428394
SPAC16E8.11c	tfb1	transcription factor TFIIH complex subunit Tfb1 (predicted)	0.05321504	6.31890999	0.74364398	0.808903015

SPCC1259.09c	pdx1	pyruvate dehydrogenase protein x component, Pdx1 (predic	0.05302814	7.58230198	0.59726689	0.6811465
SPBC1734.14c	suc1	cyclin-dependent protein kinase regulatory subunit Suc1	0.05273624	6.16391231	0.7744242	0.832405094
SPNCRNA.1308	#N/A	#N/A	0.0523188	3.00533472	1	1
SPBP35G2.08c	air1	TRAMP complex zinc knuckle subunit Air1	0.05226635	6.09057263	0.76240312	0.822955581
SPNCRNA.906	#N/A	#N/A	0.05215968	7.7050651	0.57934412	0.666515085
SPBC36B7.03	sec63	ER protein translocation subcomplex subunit Sec63 (predict	0.05214278	7.99807544	0.57074956	0.659208633
SPBC21D10.05c	ucp3	GTPase activating protein Ucp3	0.05161945	7.88002654	0.55918978	0.649144735
SPBC1778.07	SPBC1778.07	methyltransferase, human CARNMT1 ortholog	0.05008618	6.72517659	0.73569056	0.802818192
SPCC1672.07	utp21	U3 snoRNP-associated protein Utp21 (predicted)	0.0499634	7.80701342	0.5727246	0.660574725
SPAC12G12.02	efg1	rRNA processing protein Efg1 (predicted)	0.04986656	6.58555861	0.70517645	0.775865119
SPCC14G10.02	urb1	ribosome biogenesis protein Urb1 (predicted)	0.04946618	7.75293497	0.72824531	0.796181284
SPBC1683.11c	SPBC1683.11c	isocitrate lyase (predicted)	0.04942457	7.22310971	0.63497215	0.714945904
SPNCRNA.923	#N/A	#N/A	0.04938563	5.95744836	0.77251255	0.83126943
SPCC584.13	SPCC584.13	amino acid transmembrane transporter (predicted)	0.04923733	5.19047636	0.87291715	0.913455517
SPAC20G8.04c	SPAC20G8.04c	mitochondrial electron transfer flavoprotein-ubiquinone oxi	0.0491937	8.18825504	0.55137896	0.641280774
SPAC3A11.10c	dpe1	dipeptidyl peptidase, unknown specificity, implicated in glu	0.04898851	8.72985772	0.5825942	0.669122962
SPNCRNA.1229	#N/A	#N/A	0.04896781	5.14570971	0.83227441	0.880128574
SPAC8F11.06	brr6	nuclear envelope protein Brr6/Brl1	0.04890241	5.22360853	0.85876111	0.902388715
SPAC823.15	ppa1	minor serine/threonine protein phosphatase Ppa1	0.04866689	6.60340362	0.74343608	0.808806162
SPBC25H2.09	mic19	MICOS complex subunit Mic19/25	0.04797313	5.65626323	0.8022387	0.855277193
SPCC188.09c	pfl4	cell surface glycoprotein, flocculin Pfl4	0.04764017	6.64175584	0.74974145	0.814104123
SPBC14C8.19	tam10	nucleolar RNA binding protein, human KNOP1 ortholog, imp	0.0476138	5.42740724	0.84646153	0.892772896
SPAC6F6.02c	pof5	F-box protein Pof5	0.04754197	5.26361489	0.85593108	0.899970798
SPNCRNA.866	#N/A	#N/A	0.04733595	6.90265435	0.70436556	0.775269219
SPBC15C4.03	mrs6	Rab geranylgeranyltransferase escort protein Mrs6 (predict	0.04715525	6.80951499	0.69197561	0.765673826
SPBC16G5.04	mrpl23	mitochondrial ribosomal protein subunit L13 (predicted)	0.04672239	5.89360125	0.76891335	0.828410559
SPAC13C5.04	SPAC13C5.04	class I glutamine amidotransferase family protein, conserve	0.04620927	6.27301875	0.79673303	0.851024111
SPAC1F5.06	lsh1	ER heat shock protein Lsh1 (predicted)	0.04558414	9.07004348	0.56304244	0.652170855
SPAC4F10.18	nup37	nucleoporin, WD repeat Nup37	0.04543578	6.79860981	0.74008406	0.806190498
SPAPB8E5.07c	rrp12	rRNA processing protein Rrp12 (predicted)	0.04511398	8.5269948	0.61560488	0.698314925
SPBC4.02c	SPBC4.02c	rRNA 2'-O-methyltransferase fibrillarin-like (predicted)	0.04510281	7.3741036	0.71388518	0.782915174
SPAC17H9.17c	mdm10	ERMES complex subunit Mdm10 (predicted)	0.04507633	5.66802709	0.81053598	0.86209711
SPBC106.09	cut4	anaphase-promoting complex, platform subcomplex scaffol	0.04500591	7.51139363	0.71114459	0.780664448
SPAC2C4.12c	tpt1	tRNA 2'-phosphotransferase Tpt1/ CIA machinery involved i	0.04492591	5.83002296	0.79064331	0.845670538
SPNCRNA.1503	#N/A	#N/A	0.04463849	8.15702098	0.59421121	0.678116259
SPAC22G7.02	kap111	karyopherin/importin beta family nuclear import signal rece	0.04427695	7.42032707	0.63905511	0.718686175
SPBC1709.06	dus2	tRNA dihydrouridine synthase Dus2 (predicted)	0.04382295	6.36853297	0.76079564	0.822041022
SPBC651.10	nse5	Smc5-6 complex non-SMC subunit Nse5	0.04362587	5.36306026	0.86430816	0.906278141
SPNCRNA.677	#N/A	#N/A	0.04349193	7.02337161	0.71762734	0.786258899
SPAC664.03	paf1	RNA polymerase II associated Paf1 complex (predicted)	0.04334535	7.57879306	0.65819199	0.735950792
SPBPB8B6.05c	SPBPB8B6.05c	L-asparaginase (predicted)	0.04329746	6.95594111	0.70119964	0.77261392
SPNCRNA.784	#N/A	#N/A	0.04286131	2.90481446	1	1
SPCC1795.03	gms1	Golgi UDP-galactose transmembrane transporter Gms1	0.04268296	7.55340999	0.69744309	0.76897282
SPCC18.12c	utp23	rRNA processing protein Utp23 (predicted)	0.04210477	6.12974707	0.84454413	0.891067639
SPBC646.07c	tsc13	enoyl reductase (predicted)	0.04186197	6.64313939	0.80174086	0.855014347
SPCC1682.02c	mcm3	MCM complex subunit Mcm3	0.04184596	8.33364238	0.59696631	0.680917828
SPAC12B10.07	acp1	F-actin capping protein alpha subunit	0.04180598	7.81503918	0.63542664	0.71519654

SPCC1753.05	rsm1	RNA export factor Rsm1	0.04148936	7.22748277	0.69651349	0.768570505
SPCC70.05c	nnk1	serine/threonine protein kinase Nnk1 (predicted)	0.04146336	8.33020728	0.65340165	0.731917399
SPNCRNA.1016	#N/A	#N/A	0.04106793	3.99408091	0.94894458	0.969604787
SPNCRNA.766	#N/A	#N/A	0.04060046	5.01610107	0.88729164	0.92338578
SPBC15D4.06	naa30	NatC N-acetyltransferase complex catalytic subunit Naa30 (	0.04053569	5.52762259	0.84126319	0.888391686
SPAC4F10.11	spn1	mitotic septin Spn1	0.04045371	6.82528761	0.73913177	0.805282051
SPBPB7E8.01	SPBPB7E8.01	Schizosaccharomyces specific protein, predicted GPI anchor	0.03982414	9.41362844	0.59304229	0.677099798
SPAC767.01c	vps1	dynamin family protein Vps1	0.03960414	8.87836708	0.61819235	0.70078277
SPAC959.11	SPAC959.11	Schizosaccharomyces specific protein	0.03924232	5.32659005	0.87850068	0.917323476
SPNCRNA.1520	#N/A	#N/A	0.03912455	8.08728225	0.63326344	0.713598452
SPBP4H10.03	oxa102	mitochondrial inner membrane insertase Oxa102	0.0389687	5.96528488	0.87520856	0.91529034
SPNCRNA.967	#N/A	#N/A	0.0388346	7.44281426	0.69093195	0.764767719
SPBC16G5.18	erg24	C-14 sterol reductase Erg24 (predicted)	0.03877922	6.58634897	0.77435505	0.832405094
SPBC12C2.09c	izh2	Haemolysin-III family plasma membrane receptor implicate	0.03846607	5.89422615	0.84503815	0.891409802
SPAC22A12.12c	rrp40	exosome subunit Rrp40	0.03785786	6.15356131	0.82980015	0.877784866
SPCC1739.14	npp106	nucleoporin Npp106	0.03757215	7.83582677	0.67472509	0.750000686
SPCC320.05	SPCC320.05	sulfate transmembrane transporter (predicted)	0.03753525	6.93606146	0.80492222	0.857734996
SPCC794.10	ugp1	UTP-glucose-1-phosphate uridylyltransferase-like Ugp1	0.03743904	6.89188122	0.7703392	0.829455415
SPAC16A10.07c	taz1	shelterin complex subunit Taz1	0.03726599	6.87239339	0.77373123	0.832054517
SPAC31A2.09c	apm4	AP-2 adaptor complex mu subunit Apm4 (predicted)	0.0372624	7.09717702	0.75476907	0.817997054
SPBC1778.10c	ppk21	serine/threonine protein kinase Ppk21 (predicted)	0.03725838	7.05055225	0.73176603	0.799171801
SPAC23D3.06c	nup146	nucleoporin, WD repeat Nup146	0.03686812	8.45282236	0.69651009	0.768570505
SPAC20H4.01	utp5	U3 snoRNP-associated protein Utp5 (predicted)	0.03670075	7.11454581	0.80046712	0.854057498
SPBC16A3.14	SPBC16A3.14	superoxide dismutase, mitochondrial ribosomal protein sub	0.03648664	6.61567332	0.79674866	0.851024111
SPBC119.06	sco1	mitochondrial copper chaperone for cytochrome c oxidase S	0.03628285	6.48811608	0.79725618	0.85129896
SPBC1861.04c	prp24	RNA-binding protein Prp24	0.03620506	7.74535271	0.70672953	0.77707133
SPBP4H10.18c	SPBP4H10.18c	Schizosaccharomyces specific protein	0.03613284	6.15995572	0.80758265	0.859762188
SPBC3H7.12	rav2	RAVE complex subunit Rav2	0.03607037	6.1934196	0.82707447	0.875854611
SPBC106.19	ppr10	mitochondrial PPR repeat protein Ppr10	0.03516655	6.32967103	0.83478997	0.882240369
SPBC13E7.03c	vts1	Smaug family RNA-binding protein Vts1 (predicted)	0.03484338	7.81615522	0.69316672	0.766585408
SPAC1782.09c	clp1	Cdc14-related protein phosphatase Clp1/Flp1	0.03473025	7.10944252	0.77725213	0.83425879
SPBC902.04	rmn1	RNA-binding protein	0.03453742	7.73387648	0.74202846	0.807662133
SPNCRNA.166	#N/A	#N/A	0.03438995	1.53106549	1	1
SPCC364.02c	bis1	splicing factor Bis1	0.03434736	5.46003605	0.920965	0.949426874
SPAC13G6.04	tim8	Tim8-Tim13 mitochondrial intermembrane space protein tr	0.03432231	5.7570302	0.85366261	0.898418502
SPAC22F3.06c	lon1	mitochondrial matrix Lon protease	0.03417736	9.14611334	0.68472115	0.759746849
SPNCRNA.814	#N/A	#N/A	0.03395388	6.13925562	0.82737409	0.875899296
SPCC1281.05	rsc7	RSC complex subunit Rsc7	0.03386021	7.14027198	0.78426077	0.840853085
SPAC6G9.02c	nop9	pumilio family RNA-binding protein Nop9 (predicted)	0.03378996	7.40819657	0.78680488	0.842784075
SPAC11D3.15	SPAC11D3.15	5-oxoprolinase (ATP-hydrolizing) (predicted)	0.03377218	8.94705631	0.73199674	0.799171801
SPBP4H10.05c	spe2	S-adenosylmethionine decarboxylase proenzyme Spe2	0.03337937	7.36164706	0.73910008	0.805282051
SPAP27G11.06c	vas2	AP-1 adaptor complex sigma subunit Aps1	0.03293842	6.50496174	0.86476175	0.906594312
SPAC1952.07	rad1	checkpoint clamp complex protein Rad1	0.03244528	7.11889647	0.78059326	0.837316699
SPBP4H10.07	SPBP4H10.07	ubiquitin-protein ligase E3, unknown specificity (predicted)	0.0322293	6.77718916	0.78589629	0.842341195
SPBC16E9.03c	coa1	cytochrome c oxidase assembly protein Coa1 (predicted)	0.03215332	5.94746513	0.86256322	0.905824493
SPBC23E6.07c	rfc1	DNA replication factor C complex subunit Rfc1	0.03162922	7.42493196	0.7690036	0.828410559
SPNCRNA.299	#N/A	#N/A	0.03141544	5.00951917	0.9322208	0.957693623

SPBC557.03c	pim1	RCC1 family Ran GDP/GTP exchange factor	0.03140086	7.90497421	0.71405786	0.782978361
SPAC4H3.14c	rng8	contractile ring myosin V regulator Rng8	0.03128824	5.74480023	0.89654924	0.930742571
SPCC1322.16	phb2	prohibitin Phb2 (predicted)	0.03073771	7.50034387	0.74685448	0.811577213
SPAC1687.22c	puf3	pumilio family RNA-binding protein Puf3 (predicted)	0.0298742	8.10649526	0.77507156	0.832706345
SPCC622.05	#N/A	#N/A	0.02935366	5.32939229	0.93603367	0.960016445
SPAC16E8.04c	aro7	chorismate mutase Aro7 (predicted)	0.02883968	6.83128147	0.8407702	0.888008882
SPNCRNA.192	#N/A	#N/A	0.02880555	3.25261136	1	1
SPBC1347.14c	#N/A	#N/A	0.02879512	5.79007369	0.92032318	0.949196613
SPNCRNA.1422	#N/A	#N/A	0.02876144	6.31040534	0.84919612	0.894824962
ScpofMt25	#N/A	#N/A	0.02839681	4.13174976	0.95861102	0.977746094
SPAC17A2.12	rrp1	ATP-dependent DNA helicase/ ubiquitin-protein ligase E3 (p	0.02833004	6.43489833	0.90084116	0.93391592
SPNCRNA.563	#N/A	#N/A	0.02822215	6.51200408	0.86432699	0.906278141
SPAC22A12.13	mug84	pig-P subunit (predicted)	0.02807415	5.57148144	0.87229474	0.9134019
SPAC18G6.11c	rrn3	RNA polymerase I general transcription inititation factor su	0.02805783	7.22365909	0.84378762	0.890504765
SPBC646.04	pla1	poly(A) polymerase Pla1	0.02784487	7.2335098	0.81119999	0.862398984
SPBP35G2.09	usp103	U1 snRNP-associated protein Usp103	0.02769704	4.94958598	0.93059049	0.956596422
SPCC736.08	cbf11	CBF1/Su(H)/LAG-1 family transcription factor Cbf11	0.02766717	8.02146262	0.75015389	0.814293588
SPAC13G7.02c	ssa1	heat shock protein Ssa1 (predicted)	0.02759575	8.14461253	0.8387838	0.88604839
SPBC1105.06	pmc4	mediator complex subunit Med4	0.0275925	6.37546311	0.87664245	0.915804709
SPCC417.12	SPCC417.12	carboxylesterase, type B family protein	0.02759069	5.74350913	0.95427181	0.974316524
SPBP4H10.12	SPBP4H10.12	protein with a role in ER insertion of tail-anchored membra	0.02746232	6.58658487	0.84763592	0.893734493
SPNCRNA.1035	#N/A	#N/A	0.02699314	3.39090162	1	1
SPAC8F11.09c	nnt1	nicotinamide N-methyltransferase Nnt1 (predicted)	0.02684336	6.18352517	0.8810995	0.919456377
SPBC3D6.12	dip2	U3 snoRNA associated protein Dip2 (predicted)	0.02656896	7.61169517	0.79118555	0.845878661
SPBC36B7.08c	ccp1	histone chaperone, CENP-A nucleosome disassembly Ccp1	0.02632342	7.38671934	0.7958731	0.850375307
SPAC1782.10c	nhp2	box H/ACA snoRNP complex subunit Nhp2	0.02607826	7.4011517	0.81581737	0.866138327
SPBC83.11	pet2	Golgi phosphoenolpyruvate transmembrane transporter Pet	0.02587264	7.67533654	0.79589131	0.850375307
SPNCRNA.1574	#N/A	#N/A	0.02551008	7.64427962	0.80857097	0.860485422
SPCC23B6.01c	SPCC23B6.01c	sterol intermembrane transfer protein (predicted)	0.02545725	8.59890746	0.77762573	0.834528166
SPBC887.22	new19	signal peptidase complex subunit Spc1 (predicted)	0.02491667	5.88423696	0.92077834	0.949378274
SPBC36B7.07	tlg1	SNARE Tlg1 (predicted)	0.02483884	7.38407075	0.8107552	0.862195525
SPBP8B7.14c	dpb2	DNA polymerase epsilon catalytic subunit B, Dpb2	0.02478327	6.67174582	0.8727106	0.913455517
SPBC409.17c	SPBC409.17c	DUF1769 family protein	0.02450047	5.73020994	0.89332513	0.928103439
SPBC365.10	arp5	Ino80 complex actin-like protein Arp5	0.02446565	7.58124342	0.80366283	0.85666127
SPAC17G6.04c	cpp1	protein farnesyltransferase beta subunit Cpp1	0.024193	8.29024896	0.7890923	0.844436724
SPBC651.08c	rpc1	DNA-directed RNA polymerase III complex large subunit Rp	0.02386195	8.47636427	0.7712952	0.830090755
SPBC11B10.03	cog8	Golgi transport complex subunit Cog8 (predicted)	0.02370427	7.55091831	0.81093087	0.862247592
SPAC222.03c	tim10	Tim9-Tim10 complex subunit Tim10 (predicted)	0.02364118	6.51907001	0.89970539	0.932956877
SPAC12G12.14c	pfs2	mRNA cleavage and polyadenylation specificity factor comp	0.02362013	6.44186733	0.8728236	0.913455517
SPCC1442.17c	ist1	MVB sorting pathway protein Ist1 (predicted)	0.02347005	5.44483627	0.93506561	0.95916815
SPCC1620.12c	SPCC1620.12c	GTPase activating protein (predicted)	0.02301225	8.48314113	0.78129551	0.837937891
SPBC19F8.02	nud3	nuclear distribution protein NUDC homolog	0.02298247	5.36789884	0.91668384	0.946558828
SPBC15D4.09c	met3	cystathionine gamma-synthase Met3	0.02263759	7.64125602	0.82965511	0.877767885
SPAC1705.02	SPAC1705.02	SERF family protein, DUF, human 4F5S homolog, implicate	0.02254862	5.85011227	0.92009165	0.94910166
SPBC16A3.12c	SPBC16A3.12c	triglyceride lipase-cholesterol esterase (predicted)	0.02240412	7.12474911	0.83647553	0.88388447
SPAC11E3.07	vma4	V-type ATPase V1 subunit E (predicted)	0.02226954	7.69269671	0.8253082	0.874256277
SPNCRNA.1018	#N/A	#N/A	0.02208451	5.90055438	0.91922698	0.948353489

SPNCRNA.950	#N/A	#N/A	0.02197263	1.53134485	1	1
SPNCRNA.1101	#N/A	#N/A	0.02191869	5.66185896	0.93795664	0.960974845
SPCC338.02	mug112	Schizosaccharomyces pombe specific protein Mug112	0.02177618	3.55850003	1	1
SPCC188.13c	dcr1	dicer	0.02173598	7.78658737	0.81215933	0.863014401
SPBC1703.02	rsc9	RSC complex subunit Rsc9	0.02132224	7.1835008	0.87615447	0.915658598
SPAC22A12.15c	bip1	ER heat shock protein BiP	0.02126369	10.0931966	0.75068632	0.81461017
SPNCRNA.1369	#N/A	#N/A	0.02124722	4.21643559	1	1
SPAC1639.01c	elo1	fatty acid elongase Elo1 (predicted)	0.02107056	8.07426467	0.854196	0.898701885
SPAC227.17c	SPAC227.17c	DUF3128 family, human c22orf39 ortholog	0.02092276	6.57992764	0.87928539	0.918002009
SPBC19G7.06	mbx1	MADS-box transcription factor Mbx1	0.02067121	7.89895674	0.89615293	0.930473103
SPAC3F10.11c	abc2	vacuolar phytochelatin and glutathione S-conjugate ABC far	0.02036642	8.99179528	0.79762784	0.851562196
SPNCRNA.1119	#N/A	#N/A	0.02016266	2.6584093	1	1
SPAC1006.02	asa1	ASTRA complex WD repeat subunit Asa1	0.0201056	4.42757812	0.95667434	0.976330069
SPAC25B8.15c	tyw3	wybutosine biosynthesis protein Tyw3 (predicted)	0.01984325	6.36262789	0.91426703	0.944955523
SPBC16H5.09c	omh2	alpha-1,2-mannosyltransferase Omh2 (predicted)	0.01963997	5.45798952	0.96826403	0.985256113
SPAC5H10.08c	pan6	pantoate-beta-alanine ligase	0.01946108	6.81458019	0.87790672	0.916843958
SPNCRNA.1511	#N/A	#N/A	0.01936071	5.63943978	0.97034552	0.986878082
SPAC1687.04	mcb1	MCM binding protein homolog Mcb1	0.01882755	6.49203509	0.93403255	0.958686504
SPNCRNA.1068	#N/A	#N/A	0.0179541	5.6766407	0.94713869	0.968341166
SPAC22A12.06c	fsh2	serine hydrolase-like, human TSTD2 and OVCA2 ortholog, u	0.01778256	7.09695423	0.88706114	0.923287101
SPCC285.09c	cgs2	cAMP-specific phosphodiesterase Cgs2	0.01772457	7.19810436	0.8825643	0.920295749
SPAC23D3.10c	eng2	cell wall and ascospore endo-1,3-beta-glucanase Eng2	0.01769363	7.32037306	0.88080426	0.919305676
SPNCRNA.849	#N/A	#N/A	0.01761107	4.87403893	0.96005194	0.978602971
SPAPB1A10.06c	SPAPB1A10.06c	ATP-dependent RNA helicase Dhr1 (predicted)	0.01759245	7.48788148	0.8834793	0.921056628
SPAC16C9.01c	SPAC16C9.01c	ribokinase-like, unknown specificity (predicted)	0.01749229	7.16475532	0.89768434	0.931276203
SPBC405.06	xdj1	DNAJ protein Xdj1 (predicted)	0.01708998	6.60624675	0.89415978	0.92868701
SPNCRNA.541	#N/A	#N/A	0.01708998	6.60624675	0.89415978	0.92868701
SPAC23G3.06	nop58	U3 snoRNP protein Nop58 (predicted)	0.01689736	7.20252244	0.89109278	0.92646268
SPCC1827.01c	utp25	U3 associated protein Utp25 (predicted)	0.01627444	6.60883494	0.9378581	0.960974845
SPAC227.14	yfh7	uridine kinase Yfh7 (predicted)	0.01623003	6.28079344	0.9093664	0.940600854
SPNCRNA.1582	#N/A	#N/A	0.01614164	6.61544496	0.91679195	0.946558828
SPBC18E5.05c	elp5	elongator complex subunit Elp5 (predicted)	0.01598139	5.8653201	0.9734334	0.989279311
SPAC2E1P3.01	SPAC2E1P3.01	dehydrogenase (predicted)	0.01542255	7.81702566	0.90466946	0.936743107
SPBC16E9.18	psd1	phosphatidylserine decarboxylase Psd1	0.01520522	7.5025922	0.88494179	0.921602286
SPNCRNA.915	#N/A	#N/A	0.01482163	2.36051452	1	1
SPBC2G2.16	mpi1	mannose-6-phosphate isomerase Mpi1 (predicted)	0.01477379	8.06148774	0.88400169	0.921229937
SPNCRNA.1402	#N/A	#N/A	0.01445327	5.10189218	1	1
SPNCRNA.1269	#N/A	#N/A	0.01439496	5.4060697	1	1
SPAC9.05	fml1	ATP-dependent 3' to 5' DNA helicase, FANCM ortholog Fml	0.01417084	8.09996002	0.87554315	0.915358905
SPBC21D10.11c	nfs1	mitochondrial [2Fe-2S] cluster asssembly and tRNA modific	0.01378526	8.40570668	0.88033601	0.918957898
SPBC839.11c	hut1	ER uridine diphosphate-glucose transmembrane transporte	0.01363774	7.75023563	0.91614371	0.946176646
SPAC4F10.13c	mpd2	GYF domain protein	0.01332602	8.33299746	0.87541124	0.915358905
SPBC12D12.09	rev7	DNA polymerase zeta Rev7 (predicted)	0.01305444	5.21492089	1	1
SPAC11G7.01	mtl2	plasma membrane-associated serine-rich cell wall sensor M	0.013014	7.13282127	0.93271343	0.957775599
SPCC1235.05c	fft2	SMARCAD1 family ATP-dependent DNA helicase Fft2 (predi	0.01273266	8.23605403	0.87414866	0.914603568
SPAC17A5.05c	SPAC17A5.05c	methyltransferase (predicted)	0.01247892	5.38257909	0.96698437	0.984489618
SPAC13C5.01c	pre9	20S proteasome complex subunit alpha 3 Pre9	0.01231152	7.69146861	0.90657891	0.938149249

SPAC16E8.13	SPAC16E8.13	ubiquitin-protein ligase E3, human BRAP ortholog (predicte	0.01161947	7.15125149	0.92520838	0.952502728
SPCC13B11.03c	SPCC13B11.03c	hydroxyacylglutathione hydrolase (predicted)	0.01157812	4.25333484	1	1
SPBC1289.06c	ppr8	mitochondrial PPR repeat protein Ppr8	0.01149933	7.5573399	0.94240844	0.964519389
SPAC10F6.04	ats1	RCC domain protein Ats1 (predicted)	0.01118498	7.52044632	0.92465249	0.952074475
SPBC3F6.02c	erg26	3 beta-hydroxysteroid dehydrogenase/delta 5-->4-isomeras	0.01109131	8.109099	0.92576127	0.952850756
SPBC2A9.09	plp2	phosducin family protein Plp2 (predicted)	0.01108878	6.80167035	0.94602414	0.967346995
SPCC553.02	qns1	glutamine-dependent NAD(+) synthetase Qns1 (predicted)	0.01091185	8.45958793	0.89672537	0.930783422
SPCC576.17c	SPCC576.17c	pyridoxamine/pyridoxine/pyridoxal transmembrane transpc	0.01075507	6.7224646	0.94423563	0.96580843
SPNCRNA.242	#N/A	#N/A	0.01054122	5.77779022	1	1
SPNCRNA.583	#N/A	#N/A	0.01032057	7.5698232	0.93306608	0.957838987
SPBC26H8.16	sdh8	mitochondrial respiratory chain complex II assembly factor	0.01020636	6.17050819	0.9525734	0.972728388
SPBC13G1.06c	isd11	mitochondrial [2Fe-2S] cluster assembly protein Isd11 (pre	0.0099065	6.10913852	0.97947243	0.994822376
SPAC7D4.09c	dfg10	3-oxo-5-alpha-steroid 4-dehydrogenase (predicted)	0.00979228	5.53365311	0.97460212	0.99031916
SPAC9E9.07c	ypt2	GTPase Ypt2	0.00966645	7.66725179	0.92859989	0.954982964
SPBC8E4.12c	ecl3	extender of the chronological lifespan protein Ecl3	0.00917998	3.63391517	1	1
SPBC13G1.10c	slh1	ATP-dependent RNA helicase Slh1, human ASCC3 ortholog (	0.00896767	8.55111299	0.92782474	0.95447428
SPBP35G2.07	ilv1	acetolactate synthase catalytic subunit	0.00874545	8.90106124	0.91388562	0.944704792
SPNCRNA.86	#N/A	#N/A	0.00874318	5.81812014	1	1
SPBC1703.01c	pop4	RNase P and RNase MRP subunit Pop4	0.00849392	4.81239407	1	1
SPNCRNA.633	#N/A	#N/A	0.00844224	7.33393275	0.95556322	0.975447258
SPBC16A3.13	meu7	alpha-amylase homolog Aah4	0.00816331	7.42017727	0.95878026	0.977746094
SPAC1805.05	cki3	serine/threonine protein kinase Cki3	0.00813376	8.20373422	0.93094384	0.956670614
SPBC14C8.12	rpb8	DNA-directed RNA polymerase I, II and III subunit Rpb8	0.00811323	6.33679517	1	1
SPBC2G2.14	csi1	mitotic centromere-SPB clustering protein Csi1	0.00774913	7.04092334	0.93937435	0.962137633
SPAC589.05c	qtr3	tRNA queuosine modification protein Qtr3	0.00733167	5.78513557	1	1
SPCC1450.02	bdf1	Swr1 complex bromodomain subunit Bdf1	0.00706293	8.12321244	0.93901366	0.961912985
SPAC644.10	med11	mediator complex subunit Med11	0.00684196	5.65472525	1	1
SPBC1683.10c	pcl1	vacuolar ferrous iron/manganese transmembrane transpor	0.0063729	7.96320323	0.96371885	0.981752512
SPBP4G3.03	fub2	PI31 proteasome regulator Fub2 (predicted)	0.00557257	8.15205169	0.97612979	0.991723383
SPBC660.13c	ssb1	DNA replication factor A subunit Ssb1	0.00540128	7.91506425	0.95959918	0.978288004
SPBP4H10.11c	lcf2	long-chain-fatty-acid-CoA ligase	0.00504124	8.4509922	0.95050606	0.970908694
SPNCRNA.1539	#N/A	#N/A	0.00471264	6.62991356	0.98245945	0.997357583
SPAC821.04c	cid13	poly(A) polymerase Cid13	0.0042839	7.13620078	1	1
SPBP4H10.04	ppb1	calcium-dependent serine/threonine protein phosphatase c	0.00422925	7.36667234	0.98549557	0.999407576
SPCC830.11c	fap7	nucleoside-triphosphatase involved in SSU-rRNA maturatio	0.00418451	5.46584745	1	1
SPNCRNA.796	#N/A	#N/A	0.00403656	6.92277772	0.98273581	0.997392568
SPNCRNA.965	#N/A	#N/A	0.00384904	6.00131316	1	1
SPAC1B3.08	SPAC1B3.08	TREX2 complex subunit (predicted)	0.0038016	7.1165473	1	1
SPCC1235.16	vma21	V-ATPase assembly protein Vma21 (predicted)	0.00366554	5.38739263	1	1
SPCC4B3.13	SPCC4B3.13	MatE family transmembrane transporter (predicted)	0.00365826	7.45285521	0.97167138	0.987636102
SPAC1687.16c	erg31	C-5 sterol desaturase Erg31	0.00328681	6.86340823	1	1
SPAC27D7.08c	mtl16	23S rRNA/U6 snRNA (adenine-N(6))-methyltransferase Mtl	0.0030796	5.47212431	1	1
SPNCRNA.618	#N/A	#N/A	0.00296696	5.73331963	1	1
SPNCRNA.269	#N/A	#N/A	0.00275578	4.44093648	1	1
SPBC16G5.03	mrz1	ubiquitin-protein ligase E3/SUMO transferase, Topors, poss	0.00262581	6.29844515	1	1
SPBC23G7.11	mag2	DNA-3-methyladenine glycosidase Mag2	0.00260535	4.56547529	1	1
SPBC1709.18	tif452	translation initiation factor eIF4E, 4F complex E subunit iso	0.00212387	7.1654805	0.98560239	0.999407576

SPCC1235.08c	pdh1	Golgi to ER retrograde transport protein (predicted)	0.00201215	7.15918995	1	1
SPBC776.08c	utp22	small-subunit processome, UTP-C complex subunit Utp22 (p	0.00175377	7.41183775	1	1
SPBC14C8.17c	spt8	SAGA complex subunit Spt8	0.00150952	6.71551309	0.98450729	0.998634142
SPAC6B12.11	drc1	replication preinitiation complex assembly protein	0.001386	5.79454019	1	1
SPAC23C4.12	hhp2	serine/threonine protein kinase Hhp2	0.00099891	6.68911227	1	1
SPAC26F1.04c	etr1	enoyl-[acyl-carrier protein] reductase (predicted)	0.00094419	7.29005003	1	1
SPAC15A10.09c	pun1	SUR7 family protein Pun1 (predicted)	0.00081002	6.43463218	1	1
SPBC1105.16c	rpr2	RNase P subunit Rpr2 (predicted)	0.00046258	5.81504471	1	1
SPAC926.10	new9	Schizosaccharomyces specific protein New9	-0.0002021	3.55740699	1	1
SPAC6B12.07c	SPAC6B12.07c	ubiquitin-protein ligase E3 with SPX domain, human LORNF	-0.0002518	7.66399435	0.9935991	1
SPAC1F3.03	sro7	Lgl family protein Sro7 (predicted)	-0.0004882	8.25829463	1	1
SPBC244.01c	sid4	SIN component scaffold protein Sid4	-0.0006803	7.44988277	1	1
SPBC1709.08	cft1	mRNA cleavage and polyadenylation specificity factor comp	-0.0008651	8.39040187	1	1
SPCC1795.07	sws2	mitochondrial ribosomal protein subunit S37 (predicted)	-0.0010483	8.11662637	0.99767254	1
SPCC1919.03c	amk2	AMP-activated protein kinase beta subunit Amk2	-0.001376	7.50653581	1	1
SPAC806.06c	SPAC806.06c	nicotinamide mononucleotide (NMN) adenylyltransferase (p	-0.0014582	7.34927801	1	1
SPNCRNA.729	#N/A	#N/A	-0.0016417	5.68928656	1	1
SPBC12C2.05c	bzz1	F-BAR domain protein Bzz1	-0.001747	7.34301404	1	1
SPAC22A12.07c	ogm1	protein O-mannosyltransferase Ogm1	-0.0020357	9.02852117	0.98605654	0.999719125
SPAC4F10.22	cmc4	mitochondrial intermembrane space protein, mature-T-Cell	-0.0021865	4.14945	1	1
SPAC3A12.11c	cwf2	zf-CCCh type zinc finger protein, Prp19 complex subunit, RN	-0.0021929	6.61042372	1	1
SPAC31G5.18c	sde2	intron-specific pre-mRNA splicing-ubiquitin fusion protein S	-0.0023007	6.41156007	1	1
SPAC7D4.02c	sfp47	Ubp4 interactor Sfp47	-0.0023044	5.98311964	1	1
SPAPB24D3.01	toe3	transcription factor (predicted)	-0.0027649	7.33083306	1	1
SPBC27B12.13	tom40	mitochondrial TOM complex subunit Tom40 (predicted)	-0.0027718	8.50482471	0.98860447	1
SPBC4B4.08	ght2	plasma membrane glucose/fructose:proton symporter Ght2	-0.0027789	7.3995171	1	1
SPNCRNA.938	#N/A	#N/A	-0.002859	4.07993186	1	1
SPAC23H3.14	avl9	post-Golgi vesicle-mediated transport protein Avl9 (predicte	-0.0028708	6.73122093	1	1
SPBC3H7.13	far10	SIP/FAR complex FHA domain subunit Far10/Csc1	-0.0029553	7.0066542	0.96366987	0.981752512
SPBC1604.12	SPBC1604.12	Schizosaccharomyces specific phosphoprotein	-0.0030531	7.89943768	1	1
SPNCRNA.1633	#N/A	#N/A	-0.0032414	6.99306709	0.98161464	0.996700631
SPNCRNA.1583	#N/A	#N/A	-0.0037403	4.11507293	1	1
SPNCRNA.1004	#N/A	#N/A	-0.0037444	6.07496294	1	1
SPNCRNA.1030	#N/A	#N/A	-0.0037839	8.26856713	0.96583697	0.983615768
SPCC1223.11	ptc2	MAP kinase threonine phosphatase, protein phosphatase 2c	-0.0039943	7.41201059	0.98435762	0.998634142
SPNCRNA.1665	#N/A	#N/A	-0.0043832	6.79997157	0.98357159	0.998091962
SPBC800.03	clr3	histone deacetylase (class II) Clr3	-0.004471	7.76872058	0.98454614	0.998634142
SPCC417.06c	mug27	meiosis specific NDR family protein kinase Mug27/Slk1	-0.0046137	7.26255004	0.9698283	0.986553442
SPAC9E9.08	rad26	ATRIP, ATR checkpoint kinase regulatory subunit Rad26	-0.0049888	6.12401811	0.98022814	0.995441351
SPBC3D6.08c	lsm1	mRNA decapping complex subunit (predicted)	-0.005	6.8752735	0.98255477	0.997357583
SPCC1259.12c	gid1	GID complex subunit, Ran GTPase binding protein Gid1 (pre	-0.0050261	6.49670757	1	1
SPAC343.01c	erg8	phosphomevalonate kinase Erg8 (predicted)	-0.0051134	7.54271197	0.97090294	0.987075033
SPBC725.11c	php2	CCAAT-binding factor complex subunit Php2	-0.0053008	8.39954352	0.95922965	0.978057801
SPAC1687.12c	coq4	ubiquinone biosynthesis protein Coq4 (predicted)	-0.0058321	7.08552472	0.97097432	0.987075033
SPAC29A4.09	SPAC29A4.09	rRNA exonuclease Rrp17 (predicted)	-0.0058963	6.3851585	1	1
SPNCRNA.1482	#N/A	#N/A	-0.0061463	5.18812439	1	1
SPBC1347.08c	SPBC1347.08c	ribonuclease H2 complex subunit (predicted)	-0.0061576	7.05816042	1	1

SPAC806.05	SPAC806.05	mitochondrial ANC9 family protein	-0.0063873	7.37460203	0.95246024	0.972728388
SPAC30.01c	sec72	Sec7 domain protein, ARF GEF Sec72	-0.0065599	8.93276489	0.93674185	0.960453263
SPCC16A11.03c	SPCC16A11.03c	DUF2009 family protein, conserved in yeast and apicomplex	-0.006738	6.60699743	1	1
SPCC1322.10	SPCC1322.10	conserved fungal cell surface protein, Kre9/Knh1 family	-0.0069562	7.71046522	0.95864152	0.977746094
SPNCRNA.1519	#N/A	#N/A	-0.0070099	3.97567644	1	1
SPBC216.06c	swi1	replication fork protection complex subunit Swi1	-0.0078933	8.85658955	0.92293159	0.950878039
SPCC4G3.19	alp16	gamma tubulin complex subunit Alp16	-0.007935	6.7537169	0.9614416	0.979848792
SPAC23H4.15	tsr1	ribosome biogenesis protein Tsr1 (predicted)	-0.0081605	7.59178203	0.94763665	0.968413789
SPCC550.02c	cwf5	Prp19 complex subunit, RNA-binding Cwf5	-0.0082004	6.26654306	1	1
SPAPB17E12.12c	SPAPB17E12.12c	mitochondrial carrier, 3'-phosphoadenosine 5'-phosphosulfa	-0.0088224	5.13710185	1	1
SPCC4B3.09c	mrpl12	mitochondrial ribosomal protein subunit L12 (predicted)	-0.0088907	7.44603427	0.91610946	0.946176646
SPBC1271.05c	SPBC1271.05c	zf-AN1 type zinc finger protein, involved in ER membrane tı	-0.0088947	6.93147178	0.96156212	0.979848792
SPAC824.06	tim14	TIM23 translocase complex subunit Tim14 (predicted)	-0.0089038	6.64736584	0.9682417	0.985256113
SPAC1851.03	ckb1	CK2 family regulatory subunit Ckb1	-0.0089204	8.01592793	0.92395384	0.951787148
SPCC126.08c	SPCC126.08c	lectin family glycoprotein receptor (predicted)	-0.0094076	7.37797736	0.94415914	0.96580843
SPNCRNA.1033	#N/A	#N/A	-0.0095509	7.41827079	0.92426793	0.95193755
SPAC630.09c	mug58	GLYK family kinase of unknown specificity, implicated in nu	-0.0100089	7.03672883	0.91261235	0.943531915
SPBC1539.10	nop16	ribosome biogenesis protein Nop16 (predicted)	-0.0100135	6.11476419	0.94761362	0.968413789
SPCC4B3.12	set9	histone lysine H3-K20 methyltransferase Set9	-0.0102037	7.58868145	0.93290565	0.957818792
SPAP27G11.15	slx1	structure-specific endonuclease catalytic subunit Slx1	-0.0103661	7.54377471	0.92748063	0.954334345
SPBC24C6.13	coa6	cytochrome c oxidase assembly protein Coa6 (predicted)	-0.0104405	5.5019642	0.96801558	0.985256113
SPBC336.05c	hen1	small RNA 2'-O-methyltransferase Hen1 (predicted)	-0.0104636	7.43263865	0.89977343	0.932956877
SPAPB1E7.05	gde1	glycerophosphoryl diester phosphodiesterase Gde1 (predict	-0.0107208	8.61778173	0.9374218	0.960741615
SPAC1071.02	mms19	CIA machinery protein Mms19	-0.0109422	7.74256554	0.93258409	0.957775599
SPNCRNA.1483	#N/A	#N/A	-0.0110089	5.5134495	1	1
SPAC2C4.06c	rcm1	tRNA (cytosine-5-)-methyltransferase Rcm1 (predicted)	-0.0113129	5.72865447	0.96988134	0.986553442
SPAC6G9.10c	sen1	ATP-dependent 5' to 3' DNA/RNA helicase Sen1	-0.0116468	8.35011784	0.88497889	0.921602286
SPAC22E12.16c	pik1	1-phosphatidylinositol 4-kinase Pik1	-0.0119869	7.95058692	0.90814813	0.939630234
SPBC336.04	cdc6	DNA polymerase delta catalytic subunit Cdc6	-0.0121155	8.21941844	0.8879868	0.923826662
SPCC1739.12	ppe1	serine/threonine protein phosphatase Ppe1	-0.01217	8.07498128	0.90274328	0.935033184
SPCPB16A4.04c	trm8	tRNA (guanine-N7-)-methyltransferase catalytic subunit Trı	-0.0122737	5.84834769	0.94959445	0.970123137
SPAC17A5.16	ftp105	Golgi localized protein, human HID1 ortholog 3, implicated	-0.0123146	6.96556719	0.92437962	0.95193755
SPBC4C3.09	otg3	alpha-1,3-galactosyltransferase	-0.0125429	7.20132845	0.91738498	0.947027422
SPAC607.02c	SPAC607.02c	conserved fungal protein	-0.0126772	5.49485597	0.96655189	0.984196564
SPAC4A8.07c	lcb4	sphingoid long chain base kinase (predicted)	-0.0130362	7.88270057	0.90262397	0.935033184
SPBC1711.12	ppp16	serine-type peptidase activity	-0.0130392	7.83265467	0.92754847	0.954334345
SPCC63.06	SPCC63.06	WD repeat protein, human WDR89 family	-0.0130538	6.89430255	0.93466587	0.95916815
SPAC23H4.17c	srb10	cyclin-dependent protein Srb mediator subunit kinase Srb1C	-0.0133309	5.16209041	0.96831637	0.985256113
SPAC1805.01c	ppk6	serine/threonine protein kinase Ppk6 (predicted)	-0.0134885	7.49197746	0.88427258	0.921371137
SPBC11B10.10c	pht1	histone H2A variant H2A.Z, Pht1	-0.013632	6.52070668	0.93502195	0.95916815
SPNCRNA.1116	#N/A	#N/A	-0.0137211	6.94900854	0.95757732	0.977105058
SPNCRNA.1080	#N/A	#N/A	-0.0138013	4.46207201	1	1
SPBC19C2.05	pat1	serine/threonine protein kinase Ran1/Pat1	-0.0138589	7.30766106	0.92202266	0.950085437
SPNCRNA.458	#N/A	#N/A	-0.0140277	6.95147379	0.94348355	0.965329358
SPBC651.02	nit1	bis(5'-adenosyl)-triphosphatase Nit1 (predicted)	-0.0142772	6.34171026	0.97850398	0.993987096
SPBC1289.09	tim21	TIM23 translocase complex subunit Tim21 (predicted)	-0.0144168	6.96631631	0.9365003	0.960350291
SPCC794.11c	ent3	ENTH/VHS domain protein Ent3 (predicted)	-0.0144562	8.30023458	0.88908986	0.924832846

SPCC594.01	SPCC594.01	DUF1769 family protein	-0.0152622	8.48569284	0.85128023	0.896604541
SPCC1919.08c	mrpl33	mitochondrial ribosomal protein subunit YmL33 (predicted)	-0.0152646	5.23864425	0.96579954	0.983615768
SPAC23E2.01	fep1	iron-sensing transcription factor Fep1	-0.0155356	7.18999272	0.89127935	0.92646268
SPNCRNA.08	#N/A	#N/A	-0.0157117	3.3305742	1	1
SPBC1734.16c	pst3	SIN3 family co-repressor Pst3	-0.0161605	8.53559779	0.85410248	0.898701885
SPAC2F7.08c	snf5	SWI/SNF complex subunit Snf5	-0.0166348	6.98388568	0.91495089	0.945518726
SPBC342.01c	alg6	glucosyltransferase Alg6	-0.0172776	6.88718181	0.88586775	0.922186025
SPBC25H2.11c	spt7	SAGA complex bromodomain subunit Spt7	-0.0176452	7.43811899	0.87711018	0.916152692
SPBC1271.10c	SPBC1271.10c	transmembrane transporter (predicted)	-0.017729	7.64756881	0.85329135	0.898305633
SPBC12C2.08	dnm1	mitochondrial dynamin family scission GTPase Dnm1	-0.0179868	8.43444058	0.82725195	0.875899296
SPBC409.07c	wis1	MAP kinase kinase Wis1	-0.0183147	8.02156203	0.85020475	0.895610425
SPBC691.02c	drp1	RINT1 family protein (predicted)	-0.0183494	7.11636885	0.90172116	0.934543477
SPBC3E7.14	smf1	Sm snRNP core protein Smf1	-0.0186661	7.2695121	0.87514731	0.91529034
SPAC23H3.02c	ini1	RING finger-like protein Ini1	-0.0188322	5.9015871	0.89977898	0.932956877
SPNCRNA.511	#N/A	#N/A	-0.0190367	5.41681085	0.93732391	0.960741615
SPAC16E8.03	gna1	glucosamine-phosphate N-acetyltransferase (predicted)	-0.0191281	4.0895282	1	1
SPAC1071.03c	sil1	nucleotide exchange factor for the ER lumenal Hsp70 chape	-0.0194794	7.20964599	0.88992694	0.925420664
SPBC21C3.11	ubx4	UBX domain protein Ubx4 (predicted)	-0.0195331	5.97701059	0.92930689	0.955565638
SPBPJ4664.01	dps1	decaprenyl diphosphate synthase subunit Dps1	-0.0195905	6.2236522	0.9095009	0.940600854
SPNCRNA.781	#N/A	#N/A	-0.0197271	6.26542048	0.91164554	0.942675565
SPAC57A10.14	sgf11	SAGA complex subunit Sgf11	-0.0197408	5.41622974	0.93478645	0.95916815
SPAC1B2.04	cox6	cytochrome c oxidase subunit VI (predicted)	-0.0198273	6.39452841	0.90340911	0.935580432
SPBC1271.11	pet802	mitochondrial carrier, S-adenosylmethionine Pet802 (predic	-0.0200149	5.66342835	0.94070564	0.963066329
SPNCRNA.25	#N/A	#N/A	-0.0200986	7.15725176	0.87264496	0.913455517
SPBC21C3.04c	mrx14	mitochondrial ribosomal protein subunit L34, Mrx14 (predic	-0.0205055	7.1414629	0.88503614	0.921602286
SPBC30D10.12c	rsm27	mitochondrial ribosomal protein subunit S27 (predicted)	-0.0206644	6.55575863	0.90895191	0.9403189
SPAC6F6.05	ost2	oligosaccharyltransferase epsilon subunit Ost2 (predicted)	-0.021279	7.59913247	0.85674094	0.90054404
SPAC19B12.01	SPAC19B12.01	TPR repeat protein, human TTC27 ortholog	-0.0214164	7.41045192	0.81746783	0.867436231
SPCC285.11	dsc5	UBX domain Sre1 cleavage protein	-0.0217198	7.20330684	0.85279423	0.897921203
SPAC27E2.14	SPAC27E2.14	Schizosaccharomyces pombe specific protein	-0.0218649	4.92088071	0.9580831	0.977474588
SPAC17G6.16c	ysh1	mRNA cleavage and polyadenylation specificity factor comp	-0.0219015	8.21894984	0.81573509	0.866138327
SPNCRNA.713	#N/A	#N/A	-0.0219519	6.87493108	0.87579062	0.915476987
SPBC16H5.03c	uba2	SUMO activating enzyme E1-type Uba2	-0.0228196	6.81169179	0.85250871	0.897759477
SPBC30D10.11	gpi1	pig-Q	-0.0230347	6.27981208	0.91597355	0.946176646
SPNCRNA.1226	#N/A	#N/A	-0.0233092	6.85007851	0.8631487	0.905983208
SPAC20G8.06	not1	CCR4-Not complex scaffold subunit Not1	-0.0235655	9.29466404	0.75380149	0.817729819
SPCC1450.09c	SPCC1450.09c	phospholipase (predicted)	-0.023701	6.85284061	0.86292947	0.905983208
SPAC1783.05	hrp1	ATP-dependent DNA helicase Hrp1	-0.0237465	8.30544426	0.79725537	0.85129896
SPAC10F6.02c	prp22	ATP-dependent RNA helicase Prp22	-0.0238279	8.18251044	0.77779856	0.83458202
SPNCRNA.653	#N/A	#N/A	-0.0240528	5.99389653	0.92582648	0.952850756
SPBC646.05c	erg9	farnesyldiphosphate:farnesyldiphosphatefarnesyltransferas	-0.0242027	8.62155072	0.79346034	0.848044233
SPAC17H9.19c	cdt2	WD repeat protein Cdt2	-0.0243109	6.8773261	0.88251521	0.920295749
SPAC222.14c	sey1	GTP binding protein Sey1 (predicted)	-0.0244684	7.74548955	0.7980218	0.851715553
SPAC683.02c	SPAC683.02c	zf-CCHC type zinc finger protein, human ZCCHC9 ortholog, ir	-0.0245556	5.74483783	0.94747367	0.968413789
SPNCRNA.1564	#N/A	#N/A	-0.0245898	5.4296929	0.97057264	0.986961571
SPNCRNA.1331	#N/A	#N/A	-0.024657	7.79869709	0.80141514	0.854934949
SPBC1685.08	cti6	histone deacetylase complex ubiquitin-like protein ligase su	-0.0247781	6.28569031	0.94278361	0.96475827

SPCC613.12c	raf1	CLRC ubiquitin ligase complex WD repeat subunit Raf1/Dos	-0.0248283	6.70289725	0.90190861	0.934595412
SPCC1450.11c	cek1	serine/threonine protein kinase Cek1	-0.0248775	8.61626476	0.75897877	0.820917459
SPBC1709.13c	set10	ribosomal lysine methyltransferase Set10	-0.0249315	7.01466767	0.82226303	0.871573192
SPBC32C12.03c	ppk25	serine/threonine protein kinase Ppk25 (predicted)	-0.0255835	5.9689144	0.89774752	0.931276203
SPAC637.08	nbp35	CIA machinery ATPase Nbp35 (predicted)	-0.0256014	6.73037968	0.85467545	0.899004034
SPAC6C3.08	nas6	proteasome assembly chaperone, gankyrin	-0.0256168	5.76379417	0.91919988	0.948353489
SPAC8F11.10c	pvg1	Golgi pyruvyltransferase Pvg1	-0.0257574	8.10824312	0.80866658	0.860485422
SPBC3D6.05	ptp4	phosphatidate cytidylyltransferase Ptp4 (predicted)	-0.0258437	6.88735684	0.85745445	0.901154829
SPAC16A10.06c	nse2	Smc5-6 complex non-SMC SUMO ligase subunit Nse2	-0.0258828	5.52696977	0.91583978	0.946176646
SPBC2G5.02c	ckb2	CK2 family regulatory subunit Ckb2 (predicted)	-0.0260429	6.28228321	0.87636803	0.915658598
SPAC22G7.04	pan2	PAN complex (poly(A)-specific ribonuclease) ubiquitin C-ter	-0.0261859	7.66482155	0.79070429	0.845670538
SPAC19A8.01c	sec73	guanyl-nucleotide exchange factor Sec73 (predicted)	-0.0262668	7.29298052	0.80690952	0.859448935
SPAP8A3.03	zip3	ER ZIP zinc transmembrane transporter Zip3 (predicted)	-0.0265159	6.79052519	0.8493357	0.894833477
SPBC29A3.11c	ort1	mitochondrial carrier, ornithine Ort1 (predicted)	-0.0266221	5.96196786	0.89520689	0.929632671
SPNCRNA.556	#N/A	#N/A	-0.0267905	5.38655711	0.93272284	0.957775599
SPAC19D5.01	pyp2	tyrosine phosphatase Pyp2	-0.0273132	5.34781984	0.94200146	0.964247887
SPAC222.10c	byr4	two-component GAP Byr4	-0.0273235	6.57597405	0.85474741	0.899004034
SPAC2E12.03c	SPAC2E12.03c	plasma membrane basic amino acid transmembrane transp	-0.0274496	6.37395852	0.88487862	0.921602286
SPBC428.08c	clr4	histone H3 lysine methyltransferase Clr4	-0.0278455	7.55690045	0.83705377	0.884358159
SPBC1703.03c	syo2	armadillo repeat protein, involved in nucleocytoplasmic tra	-0.0283329	7.17472342	0.81030053	0.861981411
SPAC688.04c	gst3	glutathione S-transferase Gst3	-0.0291294	7.41508811	0.76899113	0.828410559
SPBC1861.07	SPBC1861.07	elongin C (predicted)	-0.0292993	3.87326944	1	1
SPCC794.15	SPCC794.15	Schizosaccharomyces specific protein	-0.0295214	5.69943595	0.91913839	0.948353489
SPAP27G11.13c	nop10	box H/ACA snoRNP complex protein (predicted)	-0.0298034	5.77934374	0.88356468	0.921056628
SPBC20F10.06	mad2	mitotic spindle checkpoint protein Mad2	-0.0298541	5.50967698	0.94010188	0.962737894
SPBC1683.02	SPBC1683.02	adenine deaminase (predicted)	-0.029908	5.99807357	0.88555766	0.922004262
SPAC2C4.10c	csc4	SIP/FAR complex subunit, Csc4	-0.0301795	6.36061219	0.82819398	0.876551477
SPAC25A8.03c	SPAC25A8.03c	mitochondrial arginine methyltransferase, human NDUFAF	-0.0302059	5.36379404	0.90615821	0.937856515
SPNCRNA.09	#N/A	#N/A	-0.0304528	3.76484183	1	1
SPAC25B8.01	dap1	cytochrome P450 regulator Dap1	-0.0309572	6.16517607	0.82544389	0.874263919
SPBP23A10.08	alp5	actin-like protein Arp4	-0.0310795	7.60624834	0.74226829	0.807793969
SPBC342.06c	rtt109	RTT109 family histone lysine acetyltransferase	-0.0313893	4.79203695	0.92129036	0.949618431
SPBC1734.03	fol1	trifunctional dihydropteroatesynthase/2-amino-4-hydroxy-6	-0.031667	7.50849387	0.75436949	0.817934271
SPBC32F12.09	rum1	CDK inhibitor Rum1	-0.0321362	6.75530462	0.83362318	0.881280985
SPNCRNA.1462	#N/A	#N/A	-0.032244	8.60884507	0.68066095	0.755981352
SPAC1399.05c	toe1	transcription factor, zf-fungal binuclear cluster type	-0.0323442	6.06445552	0.8973329	0.931129976
SPBC13G1.14c	rns1	exon junction complex subunit, RNA-binding protein Rns1	-0.0327613	5.76692989	0.86132873	0.904667692
SPBC1703.11	opa3	mitochondrial outer membrane lipid metabolism regulator	-0.0330059	5.43343529	0.94060493	0.963066329
SPAC227.04	atg10	Atg12 conjugating enzyme Atg10	-0.0341346	5.09168391	0.89133742	0.92646268
SPCC1620.11	nup97	nucleoporin Nic96 homolog	-0.0343243	8.27349051	0.66055641	0.737988235
SPAC19B12.05c	fcp1	CTD phosphatase Fcp1	-0.0349796	8.05108055	0.68396769	0.75903459
SPBC146.08c	tif1102	translation initiation factor eIF1A-like (predicted)	-0.0353845	6.46827951	0.81586285	0.866138327
SPNCRNA.1254	#N/A	#N/A	-0.0355709	5.97764126	0.85546068	0.89961519
SPBC691.01	pfa5	vacuolar membrane palmitoyltransferase Pfa5 (predicted)	-0.0356463	5.99944307	0.8818441	0.919967671
SPCC1235.03	cue2	nucleolar Smr domain protein, implicated in DNA repair	-0.035776	5.91544325	0.90258454	0.935033184
SPNCRNA.722	#N/A	#N/A	-0.0358205	7.6264027	0.75588165	0.818786624
SPNCRNA.1491	#N/A	#N/A	-0.0361286	6.4679242	0.84369987	0.890504765

SPAC1F5.05c	mso1	exocytic docking protein Mso1	-0.0361898	5.36879968	0.89310223	0.928013544
SPAC4F8.01	did4	ESCRT III complex subunit Did4	-0.0364396	6.8353675	0.76702795	0.826936823
SPAC328.01c	msn5	karyopherin/importin beta family nuclear import/export sig	-0.037838	8.45314063	0.66600657	0.742735936
SPAC13A11.03	mcp7	meiosis specific coiled-coil protein Mcp7	-0.0379775	6.93584279	0.78792236	0.843582702
SPNCRNA.249	#N/A	#N/A	-0.0380268	1.8219343	1	1
SPBC2F12.05c	osh2	sterol intermembrane transfer protein Osh2 (predicted)	-0.0380525	9.15522078	0.69712128	0.768840872
SPBC12C2.12c	glo1	glyoxalase I	-0.0380845	7.94555353	0.75597889	0.818786624
SPBC646.14c	orc5	origin recognition complex subunit Orc5	-0.038257	6.21068095	0.85668515	0.90054404
SPBC776.15c	kgd2	dihydrolipoamide S-succinyltransferase, e2 component of o	-0.0384527	8.20855373	0.66805651	0.744533992
SPAC630.15	mug177	Schizosaccharomyces pombe specific protein	-0.0384939	7.13314425	0.70795346	0.778048298
SPCC1827.05c	SPCC1827.05c	nucleolar RNA-binding protein NIFK (predicted)	-0.0387487	6.48329863	0.80870997	0.860485422
SPNCRNA.1408	#N/A	#N/A	-0.0392859	7.51856344	0.68561778	0.760617701
SPBP8B7.25	cyp4	cyclophilin family peptidyl-prolyl cis-trans isomerase Cyp4	-0.039362	8.23996808	0.62397807	0.705695653
SPBC83.16c	SPBC83.16c	protein with a role in clearing protein aggregates (predicte	-0.0395433	8.8107617	0.60589805	0.689026781
SPAC23H3.08c	bub3	mitotic spindle checkpoint WD repeat protein Bub3	-0.0395952	4.80420837	0.88979852	0.925420664
SPNCRNA.1545	#N/A	#N/A	-0.0397035	4.80589179	0.92074589	0.949378274
SPCC4B3.14	cwf20	complexed with Cdc5 protein Cwf20	-0.03975	5.13686355	0.86338003	0.905983208
SPNCRNA.1174	#N/A	#N/A	-0.03975	5.13686355	0.86338003	0.905983208
SPAC1782.08c	rex3	exonuclease Rex3 (predicted)	-0.0397661	7.31701062	0.73391256	0.801134905
SPAC18G6.02c	chp1	heterochromatin (HP1) family chromodomain protein Chp1	-0.0397907	7.00488117	0.73148051	0.799121025
SPCC790.02	pep3	HOPS/CORVET complex subunit, ubiquitin-protein ligase E3	-0.039811	7.66880363	0.69377112	0.766912354
SPNCRNA.1240	#N/A	#N/A	-0.0398556	6.59833654	0.7616659	0.822589496
SPBC2F12.17	cox7	cytochrome c oxidase subunit VII Cox7 (predicted)	-0.0401257	6.00601023	0.81684928	0.867050279
SPBC2G2.09c	crs1	meiosis specific cyclin Crs1	-0.0407224	4.04342199	0.94586222	0.967326785
SPBP23A10.04	apc2	anaphase-promoting complex cullin family subunit Apc2	-0.0407978	6.33381596	0.82824776	0.876551477
SPCC553.03	pex1	AAA family ATPase Pex1 (predicted)	-0.0412381	6.97684296	0.76255556	0.822955581
SPBC9B6.05c	lsm3	Lsm2-8 complex subunit Lsm3	-0.0413599	5.93299039	0.81844513	0.868337875
SPAC29A4.06c	SPAC29A4.06c	splicing protein, human NSRP1 ortholog	-0.0414078	5.46742535	0.87232902	0.9134019
SPAC23C4.13	bet1	SNARE Bet1 (predicted)	-0.0422528	5.67078531	0.87097501	0.912405313
SPBC16C6.05	tma22	translation machinery associated protein ortholog Tma22 (|	-0.0424376	7.29498734	0.72365994	0.791975855
SPBC530.04	mod5	Tea1 anchoring protein Mod5	-0.0427363	7.46425819	0.70796107	0.778048298
SPBC15D4.04	gpt2	UDP-N-acetylglucosamine--dolichyl-phosphate N-acetylgluc	-0.0429085	6.72048691	0.77436748	0.832405094
SPAC227.08c	yth1	mRNA cleavage and polyadenylation specificity factor comp	-0.0429895	5.96909438	0.78625346	0.842458637
SPAC23C4.08	rho3	Rho family GTPase Rho3	-0.0430578	7.07182419	0.76304934	0.823169109
SPAC31G5.16c	dpm1	dolichyl-phosphate beta-D-mannosyltransferase catalytic su	-0.0431061	8.09663561	0.62759538	0.708844241
SPNCRNA.724	#N/A	#N/A	-0.0431089	8.21030747	0.59742217	0.681209423
SPCC126.05c	mrpl17	mitochondrial ribosomal protein subunit L17 (predicted)	-0.0432864	5.96438147	0.78882272	0.844281001
SPAC823.12	vps11	HOPs/CORVET complex ubiquitin protein ligase E3 subunit \	-0.0434944	7.75229954	0.64405615	0.723234666
SPAP27G11.04c	tad3	tRNA specific adenosine deaminase subunit Tad3	-0.0440732	5.54627637	0.86520263	0.906916808
SPAC1834.13	#N/A	#N/A	-0.0442463	2.20189615	1	1
SPBC713.04c	pwp2	U3 snoRNP-associated protein Utp1 (predicted)	-0.0442665	7.80063731	0.62277837	0.70492462
SPAC1F7.12	yak3	aldose reductase ARK13 family YakC, implicated in cellular	-0.0444612	7.7058521	0.69946641	0.770953829
SPNCRNA.795	#N/A	#N/A	-0.0444968	7.73723985	0.66323396	0.740250464
SPCC4B3.20	cmc2	copper binding protein of the mitochondrial inner membrar	-0.0445759	4.10803457	0.95566596	0.975447258
SPCC4G3.12c	SPCC4G3.12c	ubiquitin-protein ligase E3 (predicted)	-0.0447657	7.22945301	0.71750442	0.786250813
SPBC146.10	mug57	cell surface fascilin domain protein, implicated in adhesion	-0.0448789	6.41787684	0.79074234	0.845670538
SPBC3B9.16c	nup120	nucleoporin, WD repeat Nup120	-0.044937	7.72754924	0.64000725	0.719519142

SPCC1682.05c	srp68	signal recognition particle subunit (predicted)	-0.04528	8.35639948	0.66347423	0.740397206
SPAC6B12.12	tom70	mitochondrial TOM complex subunit Tom70 (predicted)	-0.0453468	9.18155224	0.55157772	0.641402319
SPCC1682.04	cdc31	spindle pole body half bridge protein, centrin Cdc31	-0.0462555	8.35688618	0.65729274	0.735066081
SPBC21.06c	cdc7	SIN pathway serine/threonine protein kinase Cdc7	-0.0463069	6.59181866	0.75815916	0.820364617
SPAC1F5.10	fal1	exon junction complex subunit, ATP-dependent RNA helicas	-0.0463368	7.38731173	0.65605922	0.733927826
SPBC27B12.08	sip1	Pof6 interacting protein Sip1, predicted AP-1 accessory prot	-0.0470234	9.12458994	0.53578581	0.626802908
SPBC18E5.02c	lcb1	serine palmitoyltransferase complex subunit (predicted)	-0.0471036	8.25638859	0.58416774	0.670364498
SPAC57A7.08	pzh1	serine/threonine protein phosphatase Pzh1	-0.0475901	8.13276884	0.57855183	0.665832677
SPCC1020.02	spc7	NMS complex subunit, Spc105/KNL-1 family member, blink	-0.0475906	7.08517857	0.71335182	0.782456343
SPBC409.21	sec66	ER protein translocation subcomplex subunit Sec66 (predict	-0.0478621	5.96638176	0.81133954	0.862412617
SPAC644.13c	SPAC644.13c	Rab GTPase binding (predicted)	-0.0478773	8.4229036	0.56220613	0.651534861
SPBPB2B2.09c	pan5	2-dehydropantoate 2-reductase Pan5 (predicted)	-0.0479148	6.38524562	0.78988701	0.845154257
SPNCRNA.657	#N/A	#N/A	-0.0480647	5.97153129	0.76050494	0.821987952
SPBC115.02c	afg1	AFG1 family mitochondrial ATPase Afg1 (predicted)	-0.0481173	6.21301315	0.80506096	0.857736679
SPNCRNA.335	#N/A	#N/A	-0.0481784	2.06156389	1	1
SPBC146.11c	mug97	meiotically upregulated gene Mug97	-0.0484162	5.97131652	0.80517589	0.857736679
SPBC16E9.12c	pab2	poly(A) binding protein Pab2	-0.0484434	7.35504807	0.63327275	0.713598452
SPAC17A5.13	fol2	GTP cyclohydrolase Fol2 (predicted)	-0.0486404	6.56919527	0.72069025	0.78910654
SPNCRNA.698	#N/A	#N/A	-0.0489125	4.80379773	0.90562336	0.937588154
SPBC839.10	usp107	U1 snRNP-associated protein Usp107	-0.0490811	7.31224513	0.63850077	0.718445649
SPBC8D2.18c	SPBC8D2.18c	adenosylhomocysteinase (predicted)	-0.0494558	9.50841708	0.46830931	0.563046792
SPAC664.10	klp2	kinesin-14 family minus-end directed microtubule motor Kl	-0.0495377	7.13764736	0.6893588	0.763523215
SPNCRNA.1473	#N/A	#N/A	-0.0500479	7.20320116	0.67002038	0.746478169
SPNCRNA.665	#N/A	#N/A	-0.0502005	6.30205812	0.74597894	0.810794876
SPCC1672.01	SPCC1672.01	histidinol-phosphatase (predicted)	-0.050933	9.18901832	0.52433216	0.61571706
SPNCRNA.1525	#N/A	#N/A	-0.0510505	6.15534965	0.75420751	0.817934271
SPBC365.20c	pnc1	nicotinamidase Pnc1 (predicted)	-0.0511723	8.21705532	0.54167125	0.632585014
SPAC1296.06	tah18	CIA machinery NADPH-dependent diflavin oxidoreductase T	-0.0516188	6.69937817	0.66490232	0.741626011
SPBP35G2.05c	cki2	serine/threonine protein kinase Cki2	-0.0517499	7.75534095	0.58818585	0.67406613
SPNCRNA.1110	#N/A	#N/A	-0.0518919	4.57225028	0.86370916	0.906049152
SPNCRNA.1070	#N/A	#N/A	-0.0518923	5.19838965	0.84308295	0.890037155
SPNCRNA.72	#N/A	#N/A	-0.0523016	6.18321595	0.74476098	0.809729744
SPBC685.08	SPBC685.08	Schizosaccharomyces specific protein	-0.0525251	3.75344699	0.93744663	0.960741615
SPAC6F12.08c	exo84	exocyst complex subunit Exo84 (predicted)	-0.052606	7.01561437	0.6436098	0.722852727
SPBC15D4.01c	klp9	kinesin-6 family microtubule motor protein Klp9	-0.0527383	6.31824356	0.77642513	0.833897251
SPAC6G10.09	gls1	alpha glucosidase I Gls1 (predicted)	-0.0528077	8.76567042	0.59958737	0.682877329
SPBC21C3.03	SPBC21C3.03	mitochondrial membrane ABC1 kinase family protein, unkn	-0.0529149	7.11012523	0.66444283	0.74135653
SPBC14F5.02	trs65	TRAPP complex subunit Trs65 (predicted)	-0.0529221	7.13718131	0.62433938	0.705959821
SPAC17G8.04c	arc5	ARP2/3 actin-organizing complex subunit Arc5	-0.0530002	6.94328212	0.73012396	0.797895217
SPAC17C9.07	alg8	dolichyl pyrophosphate Glc1Man9GlcNAc2 alpha-1,3-glucos	-0.0537576	6.6354343	0.76786825	0.827711616
SPAC694.02	SPAC694.02	DEAD/DEAH box helicase, human DDL60 and DDL60L orthol	-0.0541629	10.0586556	0.3941712	0.489477188
SPAC4C5.04	rad31	SUMO activating enzyme E1-type Rad31	-0.0541642	6.03562711	0.77308731	0.831624903
SPAC9G1.11c	spn4	mitotic septin Spn4	-0.0542541	6.81641407	0.66948592	0.746004864
SPAC1486.02c	dsc2	Golgi Dsc E3 ligase complex subunit Dsc2	-0.0546284	6.4750284	0.68229839	0.757552705
SPBC1734.01c	esf1	pre-rRNA processing protein Esf1 (predicted)	-0.0548625	7.35403332	0.58176152	0.668617991
SPAC4F10.12	fta1	CENP-L ortholog Fta1	-0.0551815	5.6968191	0.7747937	0.832670738
SPAC17G8.05	med20	mediator complex subunit Med20	-0.0552895	5.00928594	0.86805455	0.909485941

SPBC3D6.13c	pdi3	ER associated protein disulfide isomerase Pdi3	-0.0555566	8.52056791	0.51674967	0.608034361
SPAC2F7.10	akr1	palmitoyltransferase Akr1 (predicted)	-0.0559384	6.81321284	0.67499403	0.750177102
SPCC74.09	mug24	RNA-binding protein, rrm type	-0.0560512	4.05395202	0.90605159	0.937856515
SPBC29A10.06c	ely5	nuclear pore protein Ely5	-0.0563206	7.17055415	0.6543154	0.732217772
SPBC14C8.16c	bot1	mitochondrial ribosomal protein subunit S35	-0.056409	7.24181154	0.6489467	0.727611467
SPAC4H3.17	#N/A	#N/A	-0.056531	2.50335927	1	1
SPCC4B3.15	mid1	medial ring protein Mid1	-0.0567093	8.34408453	0.53456527	0.625897573
SPAC15A10.06	SPAC15A10.06	CPA1 sodium ion/proton antiporter (predicted)	-0.0569229	7.30411436	0.59277796	0.676934725
SPNCRNA.1306	#N/A	#N/A	-0.0571214	5.66449134	0.86025008	0.903813765
SPAC22F8.02c	pvg5	Golgi 4,6-pyruvylated galactose (PvGal) residue biosynthesi	-0.0574998	5.61558519	0.75903198	0.820917459
SPAC1B1.02c	SPAC1B1.02c	NAD/NADH kinase (predicted)	-0.0579731	6.54989634	0.68389897	0.75903459
SPBC3E7.01	fab1	1-phosphatidylinositol-3-phosphate 5-kinase Fab1	-0.0581499	9.58893879	0.4942016	0.588664774
SPBC15D4.08c	SPBC15D4.08c	Schizosaccharomyces specific protein	-0.0581658	4.30032628	0.90117696	0.934121736
SPNCRNA.432	#N/A	#N/A	-0.0583053	5.6407561	0.74192172	0.807662133
SPAC15A10.11	ubr11	UBR ubiquitin-protein ligase E3 Ubr11	-0.0583509	9.59360871	0.44568619	0.541491617
SPNCRNA.737	#N/A	#N/A	-0.0589342	7.44763388	0.55074732	0.640655642
SPAC23C4.19	spt5	DSIF transcription elongation factor complex subunit Spt5	-0.0591479	8.24241736	0.54511807	0.635791871
SPNCRNA.270	#N/A	#N/A	-0.0593838	5.42271929	0.80712345	0.859542268
SPBC21B10.06c	inp2	myosin binding vezatin family protein involved in peroxisom	-0.0596604	6.36815642	0.65208746	0.730565549
SPAC17C9.08	pnu1	mitochondrial endodeoxyribonuclease Pnu1	-0.0598076	6.94136429	0.66194145	0.739292882
SPNCRNA.769	#N/A	#N/A	-0.0603967	7.24070445	0.64663084	0.725766599
SPNCRNA.1228	#N/A	#N/A	-0.0607958	5.35800975	0.75766436	0.820220482
SPBC776.14	plh1	phospholipid-diacylglycerol acyltransferase Plh1	-0.0608009	8.56591922	0.45268509	0.547745736
SPBC31F10.16	bch1	exomer complex ChAPs family (Chs5p-Arf1p-binding) protei	-0.0608095	8.5148012	0.53382058	0.625240796
SPAC637.06	gmh5	alpha-1,2-galactosyltransferase (predicted)	-0.0611092	7.83497531	0.52459028	0.615913879
SPBC6B1.10	prp17	Prp19 complex WD repeat protein Prp17	-0.0611614	9.59161001	0.47258637	0.567472603
SPAP8A3.12c	tpp2	tripeptidyl-peptidase II Tpp2	-0.0612047	9.24955683	0.57604022	0.663723962
SPAC22H10.03c	kap114	karyopherin/importin beta family nuclear import signal rec	-0.0614364	7.9378212	0.55991419	0.649653209
SPCC188.03	cnd3	condensin complex non-SMC subunit Cnd3	-0.0615415	7.6144392	0.57524358	0.663030585
SPCC306.06c	big1	ER membrane protein, BIG1 family, implicated in protein g	-0.0616365	6.98015449	0.60485111	0.688066166
SPAC922.04	SPAC922.04	Schizosaccharomyces specific protein	-0.0617057	7.93258802	0.46284848	0.557564862
SPAC17C9.15c	SPAC17C9.15c	Schizosaccharomyces specific protein	-0.0618516	6.62176541	0.6205545	0.702758095
SPBC3B9.22c	dad4	DASH complex subunit Dad4	-0.06201	7.15580118	0.58193195	0.668700927
SPCC548.04	urm1	ubiquitin-like protein modifier Urm1 (predicted)	-0.0620669	6.86882223	0.65388902	0.732030493
SPCC548.05c	dbl5	ubiquitin-protein ligase E3 Dbl5	-0.0620669	6.86882223	0.65388902	0.732030493
SPAPB24D3.09c	pdr1	ABC transmembrane transporter Pdr1	-0.0621266	7.60568531	0.54359516	0.634396336
SPAC1D4.01	tls1	splicing factor Tls1	-0.0621582	7.11857066	0.64866975	0.727575017
SPBC216.04c	mxr2	peptide-methionine (R)-S-oxide reductase MsrB	-0.0625662	6.01675446	0.72887597	0.796659323
SPAC1F8.06	pfl8	cell surface glycoprotein, flocculin Pfl8	-0.062882	6.04745617	0.71841072	0.786863826
SPAC57A7.11	mip1	WD repeat protein, Raptor homolog Mip1	-0.0629709	8.54587434	0.4878672	0.582853986
SPAC23C4.10	sec2	guanyl-nucleotide exchange factor Sec2 (predicted)	-0.0632256	6.08433611	0.74903987	0.813472124
SPBC1921.03c	mex67	mRNA export receptor, Tap, nucleoporin Mex67	-0.0634307	6.99200995	0.65917787	0.736811006
SPAC3G6.02	rpn15	multifunctional proteasome assembly, lid subcomplex subu	-0.0638731	6.90084331	0.62170035	0.703938581
SPBC24C6.03	SPBC24C6.03	mitochondrial proline-tRNA ligase (predicted)	-0.0641814	7.59447772	0.51118272	0.602981181
SPCC18.06c	caf1	CCR4-Not complex CAF1 family ribonuclease subunit Caf1	-0.0642871	6.70450429	0.60960888	0.692552328
SPNCRNA.1050	#N/A	#N/A	-0.0644075	5.5051141	0.79799805	0.851715553
SPBC1271.08c	SPBC1271.08c	Schizosaccharomyces pombe specific protein	-0.0648063	6.67117691	0.71834119	0.786863826

SPNCRNA.844	#N/A	#N/A	-0.0653533	5.83534369	0.68677256	0.76152636
SPCC188.04c	spc25	NMS complex subunit Spc25	-0.0666898	7.62305703	0.550214	0.640254155
SPCC1672.02c	sap1	switch-activating protein Sap1	-0.0667281	8.76056566	0.4661636	0.560863387
SPNCRNA.158	#N/A	#N/A	-0.0667359	3.40613777	0.93015916	0.956297487
SPNCRNA.1686	#N/A	#N/A	-0.0668503	6.10022121	0.71009711	0.780017886
SPBC1347.06c	cki1	serine/threonine protein kinase Cki1	-0.0668866	7.47706893	0.54165247	0.632585014
SPAC9E9.10c	cbh1	CENP-B homolog Cbh1	-0.0669457	7.0298934	0.58854442	0.674316514
SPNCRNA.968	#N/A	#N/A	-0.0673418	5.53812584	0.77368211	0.832054517
SPBP8B7.16c	dbp2	ATP-dependent RNA helicase Dbp2	-0.0676242	7.37535113	0.50825938	0.600687064
SPNCRNA.234	#N/A	#N/A	-0.0676468	3.54430612	0.93111044	0.956697346
SPAC31G5.08	hem4	uroporphyrinogen-III synthase Ups	-0.067743	8.26393042	0.40088397	0.496273494
SPNCRNA.630	#N/A	#N/A	-0.067895	5.2725184	0.74786996	0.812460898
SPBC2D10.04	aly2	arrestin Aly1 related Aly2	-0.0680841	7.6025988	0.56607521	0.655237593
SPNCRNA.981	#N/A	#N/A	-0.0682813	4.97440905	0.77681648	0.834043243
SPAC1A6.09c	lag1	sphingosine N-acyltransferase Lag1	-0.0686957	7.49889329	0.50677857	0.599825561
SPAC1687.10	mcp1	microtubule binding protein Mcp1	-0.0694891	5.60354523	0.69838579	0.769887465
SPNCRNA.1038	#N/A	#N/A	-0.070173	4.86910947	0.81886605	0.868513631
SPAC22F3.15	aim41	mitochondrial aspartyl/glutamyl-tRNA amidotransferase su	-0.0705661	4.67001328	0.8534789	0.898364102
SPCC584.16c	SPCC584.16c	Schizosaccharomyces specific protein	-0.0710028	6.8726774	0.5261832	0.617145238
SPAC27F1.08	pdt1	Nramp family manganese ion transmembrane transporter	-0.0710907	6.10126912	0.67124648	0.747599392
SPAC1420.04c	cox1101	cytochrome c oxidase assembly protein Cox1101/ mitochon	-0.0711598	8.02842481	0.53320963	0.624740274
SPBC1734.13	atp3	F1-FO ATP synthase gamma subunit (predicted)	-0.0712338	8.05601663	0.39121356	0.486692559
SPBC1773.01	far8	SIP/FAR complex WD repeat (scaffold) subunit, striatin, Far	-0.0714302	7.89506948	0.50810629	0.600687064
SPAC31G5.15	psd3	phosphatidylserine decarboxylase Psd3	-0.0714814	7.26550582	0.5394311	0.630509904
SPAC343.19	lsb6	1-phosphatidylinositol 4-kinase Lsb6 (predicted)	-0.0716941	6.78407809	0.59314159	0.677099798
SPBC18A7.02c	SPBC18A7.02c	seven transmembrane receptor-like protein (predicted)	-0.0718352	7.78663015	0.42289962	0.518061553
SPCC188.11	prp45	Prp19 complex subunit Prp45	-0.0719049	6.6876334	0.62262782	0.704871451
SPAC25A8.01c	fft3	SMARCAD1 family ATP-dependent DNA helicase Fft3	-0.071993	8.38356163	0.43160293	0.527110184
SPAC13G6.03	gpi7	GPI anchor biosynthesis protein Gpi7 (predicted)	-0.0720228	7.06100524	0.50753535	0.600239497
SPAC4C5.03	SPAC4C5.03	multispanning membrane protein, CTNS/PQ domain protein	-0.0722475	7.35564199	0.5595436	0.649333932
SPNCRNA.701	#N/A	#N/A	-0.0723472	5.57983909	0.69721044	0.768840872
SPNCRNA.692	#N/A	#N/A	-0.0724421	6.81990767	0.57881897	0.666023515
SPBC4F6.15c	swi10	DNA repair endonuclease non-catalytic subunit Swi10	-0.0725615	5.48684085	0.75193189	0.815831719
SPBC1604.10	srb7	mediator complex subunit Med21	-0.0725951	6.24713824	0.67293285	0.748742324
SPNCRNA.560	#N/A	#N/A	-0.0730385	5.36478051	0.75504563	0.818166479
SPBP8B7.19	spt16	histone H2A-H2B chaperone, FACT complex subunit Spt16	-0.0730685	8.88573788	0.49998149	0.593368332
SPCC16A11.05c	dim1	U4/U6 x U5 tri-snRNP complex subunit Dim1	-0.0731687	5.51560953	0.68895437	0.763323755
SPBC30D10.03c	isn1	IMP 5'-nucleotidase Isn1 (predicted)	-0.0732313	7.89288338	0.44430515	0.540392588
SPBC1348.03	ftm6	sub-telomeric 5Tm protein family Ftm6	-0.0733857	6.23503617	0.62898429	0.709670528
SPAC22H12.02	tfg3	TFIID, TFIIF, Ino80, SWI/SNF, and NuA3 complex subunit Tfg	-0.0734579	7.93768143	0.43543484	0.531202812
SPNCRNA.98	srp7	7SL signal recognition particle component	-0.0738398	7.69462832	0.43619499	0.531859331
SPNCRNA.684	#N/A	#N/A	-0.0742891	4.74807986	0.78539957	0.841941405
SPCC16A11.14	sfh1	RSC complex subunit Sfh1	-0.0745745	7.42569569	0.45840685	0.55358627
SPCC1795.08c	vid21	NuA4 histone acetyltransferase complex subunit Vid21	-0.0750547	8.04546064	0.37785655	0.473625684
SPAC521.02	wss1	WLM domain metallopeptidase implicated in DNA repair W	-0.075137	6.81857119	0.56257608	0.651741614
SPAC1F3.09	mug161	CwfJ family protein, splicing factor (predicted)	-0.0755078	7.79521173	0.54674657	0.637127676
SPAC1B2.05	mcm5	MCM complex subunit Mcm5	-0.075575	8.2181695	0.34180274	0.436425951

SPCC1442.12	pps1	CDP-diacylglycerol-serine O-phosphatidyltransferase Pps1	-0.0758296	6.37376817	0.62958804	0.70991659
SPBC29B5.02c	isp4	plasma membrane OPT oligopeptide transmembrane trans	-0.076324	10.1702321	0.29115728	0.383754657
SPBC26H8.02c	sec9	SNAP-25 homologue, t-SNARE component Sec9	-0.0763324	8.11809229	0.36215378	0.457737083
SPBC36.06c	spo9	ER farnesyl pyrophosphate synthetase Erg20	-0.0764852	5.73918426	0.70546908	0.776061607
SPCC1259.07	rxt3	transcriptional regulatory protein Rxt3	-0.0765311	6.69390118	0.59126522	0.676260978
SPAC1486.11	fmc1	mitochondrial complex assembly factor Fmc1 (predicted)	-0.0768132	5.67493747	0.69325005	0.766585408
SPCC18.09c	hnt3	aprataxin Hnt3	-0.0769449	6.93591208	0.56905609	0.658016425
SPBC16A3.07c	nrm1	MBF complex corepressor Nrm1	-0.0769947	7.69259868	0.47274095	0.567472603
SPAC5D6.08c	mes1	meiotic APC inhibitor Mes1	-0.077382	4.84201706	0.78616484	0.842458637
SPNCRNA.1220	#N/A	#N/A	-0.0774557	3.69509535	0.86768218	0.909235788
SPBC119.15	npa3	RNA polymerase II binding AAA family ATPase Npa3 (predic	-0.0774581	6.63100307	0.56152717	0.651032329
SPBC660.17c	SPBC660.17c	Schizosaccharomyces specific protein	-0.0775074	6.02458732	0.6486311	0.727575017
SPBC83.06c	SPBC83.06c	mitochondrial ribosomal protein subunit L36, Rtc6 (predicte	-0.0775584	4.67094863	0.83006382	0.877927303
SPBP23A10.12	frg1	FRG1 family protein, involved in mRNA splicing (predicted)	-0.0775953	6.08815711	0.68820956	0.762622713
SPNCRNA.403	#N/A	#N/A	-0.0776343	4.59537404	0.78029656	0.837130391
SPAC3A12.15	vps53	GARP complex subunit Vps53 (predicted)	-0.0776717	7.0215458	0.51489659	0.606415935
SPNCRNA.1170	#N/A	#N/A	-0.0776795	6.66221872	0.58988714	0.675307703
SPCC895.03c	sua5	tRNA N6-threonyl-carbamoyl-adenosine (t6A) Sua5 (predict	-0.0777526	5.93129274	0.71461668	0.783464882
SPNCRNA.961	#N/A	#N/A	-0.0784473	5.55346653	0.67311921	0.748827237
SPBC839.08c	its8	pig-N (predicted)	-0.0786617	8.32738377	0.40349762	0.498692573
SPBC2D10.06	rep1	MBF transcription factor activator Rep1	-0.0787121	4.95842992	0.73679087	0.80337476
SPNCRNA.15	#N/A	#N/A	-0.0787924	5.28474133	0.71198433	0.781334194
SPCC18.08	SPCC18.08	mitochondrial lysine-tRNA ligase (predicted)	-0.0788822	6.92949146	0.56771839	0.656692777
SPBC29A3.21	SPBC29A3.21	Schizosaccharomyces pombe specific protein	-0.0789312	5.02006614	0.76602264	0.826049184
SPAC19B12.13	cox1102	cytochrome c oxidase assembly protein Cox1102/ mitochon	-0.0794048	8.0247642	0.48076365	0.575782588
SPAC6C3.09	rpp40	RNase P and RNase MRP subunit Rpp40	-0.0795014	5.90049689	0.62916217	0.709671563
SPNCRNA.736	#N/A	#N/A	-0.0796767	7.53718426	0.44236397	0.538416533
SPBC19G7.05c	bgs1	primary septum and spore wall linear 1,3-beta-glucan synth	-0.0797007	9.90658547	0.32250006	0.416751408
SPAC4A8.13c	pts1	20S proteasome complex subunit beta 5	-0.0798986	7.60589159	0.49829558	0.591986975
SPBC19G7.07c	ppr3	mitochondrial PPR repeat protein Ppr3	-0.0800922	8.4135682	0.37838293	0.474110816
SPCC663.15c	SPCC663.15c	DUF3818 and PXA domain conserved fungal protein	-0.0801635	8.66327135	0.42149614	0.516714328
SPCC663.12	cid12	poly(A) polymerase Cid12	-0.0802712	6.88463475	0.49396331	0.588483954
SPAC4F8.05c	mrpl28	mitochondrial ribosomal protein subunit L28 (predicted)	-0.0805473	5.68341903	0.68715855	0.761789817
SPBC16G5.06	SPBC16G5.06	Schizosaccharomyces specific protein	-0.0807144	7.21914162	0.42299753	0.51808823
SPBC18H10.16	can1	plasma membrane arginine/lysine transmembrane transpo	-0.0809459	7.98274718	0.39999729	0.495446221
SPBC3H7.18	tam8	Schizosaccharomyces specific protein Tam8	-0.0812587	5.54599526	0.75847963	0.820580899
SPNCRNA.1634	#N/A	#N/A	-0.0815258	5.32956229	0.73112516	0.798861065
SPBC6B1.02	ppk30	Ark1/Prk1 family protein kinase Ppk30	-0.081594	7.80786593	0.44455816	0.540540647
SPNCRNA.1006	#N/A	#N/A	-0.0816344	5.40034701	0.72330828	0.791718329
SPAC31A2.14	bun107	WD repeat protein, human WDR48 family Bun107	-0.0817385	8.49569331	0.39218623	0.487546095
SPAC821.12	orb6	serine/threonine protein kinase Orb6	-0.0818716	7.56628281	0.48478274	0.579983573
SPNCRNA.475	#N/A	#N/A	-0.081934	4.30758949	0.76225215	0.822955581
SPNCRNA.204	#N/A	#N/A	-0.0819671	3.71802018	0.88754802	0.923511358
SPBC32F12.03c	gpx1	glutathione peroxidase Gpx1	-0.0822693	8.09052468	0.39355376	0.488888884
SPBC16G5.02c	rbk1	ribokinase Rbk1 (predicted)	-0.0823342	7.16780373	0.53516064	0.626271391
SPAC23H3.11c	SPAC23H3.11c	beta-glucan biosynthesis protein (predicted)	-0.0829624	5.76126572	0.75459094	0.817934271
SPCC550.09	pex32	peroxin Pex32 (predicted)	-0.0830896	6.94888173	0.50455397	0.597960599

SPBC119.16c	SPBC119.16c	DUF2347 family protein, human LCHN ortholog, AVL9 clan (	-0.0832043	7.24154917	0.46821031	0.563027237
SPBC13E7.04	atp16	F1-FO ATP synthase delta subunit (predicted)	-0.0834832	7.48593116	0.40833436	0.50348167
SPBC146.12	coq6	monooxygenase Coq6 (predicted)	-0.0835502	7.32399838	0.39710405	0.492579848
SPAC56F8.11	spc3	signal peptidase subunit Spc3 (predicted)	-0.0838033	6.82005915	0.49338353	0.588205139
SPAC6B12.10c	spp1	DNA primase catalytic subunit Spp1	-0.0840355	6.64872226	0.52428877	0.61571706
SPBC1709.10c	atx1	copper chaperone Atx1	-0.0841425	4.13308179	0.78829194	0.843845627
SPNCRNA.1681	#N/A	#N/A	-0.0841614	3.09860709	0.92159995	0.949793681
SPCC576.07	ret3	coatomer zeta subunit (predicted)	-0.0843156	7.80336222	0.33717906	0.431703393
SPAC589.03c	noc12	CCAAT-binding factor, Noc1 related	-0.0843861	4.47031137	0.77499416	0.832706345
SPBC1773.11c	mug89	phospholipid-translocating ATPase complex Lem3 family su	-0.0843933	6.70397078	0.53609354	0.627039624
SPAC3A11.11c	SPAC3A11.11c	oxidoreductase, implicated in vitamin metabolism, or cellul	-0.0845706	7.16564098	0.40978315	0.504993542
SPAC13G7.04c	mac1	plasma membrane anchored protein Mac1	-0.085364	8.10066795	0.35458988	0.450015692
SPAC9G1.08c	SPAC9G1.08c	palmitoyl-(protein) hydrolase (predicted)	-0.0855251	7.616853	0.38312767	0.478645826
SPBC28E12.05	esf2	U3 snoRNP-associated protein Esf2 (predicted)	-0.0855374	8.67018166	0.27178807	0.363434424
SPBC4B4.12c	SPBC4B4.12c	Schizosaccharomyces specific protein	-0.0855611	5.95378319	0.62876148	0.709670528
SPCC757.15	cox14	cytochrome c oxidase assembly protein Cox14 (predicted)	-0.0859722	6.51556267	0.51115761	0.602981181
SPAPB8E5.04c	npc2	Niemann-Pick disease type C2 protein hE1 homolog Npc2 (p	-0.0860374	7.35067381	0.53506684	0.62626932
SPBC3H7.15	hhp1	serine/threonine protein kinase Hhp1	-0.0861222	8.55206604	0.31280208	0.406535172
SPAC27E2.13	#N/A	#N/A	-0.0862472	7.24997495	0.40330292	0.498633063
SPCC553.10	SPCC553.10	conserved fungal cell surface protein, Kre9/Knh1 family (pre	-0.0863196	8.92583394	0.48951816	0.584415984
SPCPB16A4.02c	opy1	pleckstrin homology domain protein Opy1	-0.0864196	6.28068137	0.59594149	0.679862837
SPNCRNA.510	#N/A	#N/A	-0.0864388	5.73473803	0.60882811	0.691888609
SPBC887.09c	sog2	leucine-rich repeat protein Sog2	-0.0864766	6.9369725	0.50018135	0.593470839
SPNCRNA.1023	#N/A	#N/A	-0.0865945	7.39496776	0.426697	0.521586684
SPBC13E7.10c	brf1	transcription factor TFIIIB complex subunit Brf1	-0.0869966	7.07453493	0.45042974	0.545695988
SPBC32H8.08c	omh5	alpha-1,2-mannosyltransferase Omh5 (predicted)	-0.0874915	6.61600593	0.49253486	0.587296249
SPAC14C4.11	vtc2	vacuolar transporter chaperone (VTC) complex polyphospha	-0.0875904	8.63448454	0.29961888	0.392625934
SPAPB1E7.03	rpc82	DNA-directed RNA polymerase III complex subunit Rpc82 (p	-0.087655	7.00973312	0.51522427	0.606696857
SPAC6F12.06	rdi1	Rho GDP dissociation inhibitor Rdi1 (predicted)	-0.0878602	7.35741092	0.4079806	0.503227832
SPAC139.06	hat1	histone acetyltransferase Hat1	-0.0878609	6.11847579	0.6264385	0.707655043
SPCC63.02c	aah3	alpha-amylase homolog Aah3	-0.0881866	8.35247958	0.33210927	0.426558521
SPAC4A8.14	SPAC4A8.14	ribose-phosphate pyrophosphokinase (predicted)	-0.0882755	7.45766935	0.37093392	0.466267279
SPAPB1A10.08	SPAPB1A10.08	conserved fungal protein	-0.0883089	6.54859695	0.63863471	0.718445649
SPCC576.02	SPCC576.02	hydantoin racemase family, implicated in amino acid, or de	-0.0890361	5.7875764	0.6088868	0.691888609
SPAC977.12	SPAC977.12	L-asparaginase (predicted)	-0.0890477	7.08639365	0.38742521	0.482773949
SPBC1773.13	SPBC1773.13	aromatic aminotransferase (predicted)	-0.0893584	6.17289815	0.5467048	0.637127676
SPAC29B12.07	sec16	multidomain vesicle coat component Sec16 (predicted)	-0.0895105	9.53676581	0.27995296	0.372376831
SPNCRNA.819	#N/A	#N/A	-0.0895119	3.36185004	0.93090166	0.956670614
SPCC1840.11	csl4	exosome subunit Csl4	-0.0896117	6.48318435	0.55395674	0.643728757
SPAPB18E9.05c	SPAPB18E9.05c	Schizosaccharomyces pombe specific protein	-0.0896494	6.41253014	0.61292201	0.695619461
SPBC28E12.06c	lvs1	beige protein homolog Lvs1	-0.0897419	9.13913706	0.20090871	0.281603247
SPAC1F7.01c	spt6	nucleosome remodeling protein Spt6	-0.0898506	8.81095095	0.25863735	0.349142458
SPNCRNA.990	#N/A	#N/A	-0.0898566	7.06624823	0.46138813	0.556495253
SPAC26H5.10c	tif51	translation elongation and termination factor eIF5A (predic	-0.0899635	8.92779286	0.21688535	0.301082178
SPBC16G5.13	ptf2	Mst2 histone acetytransferase acytyltransferase complex su	-0.0901527	5.27710336	0.67234891	0.74841146
SPNCRNA.1371	#N/A	#N/A	-0.0901571	6.43131969	0.52978603	0.621156779
SPNCRNA.1618	#N/A	#N/A	-0.090164	5.97912835	0.61868593	0.70111819

SPBC16D10.07c	sir2	Sirtuin family histone deacetylase Sir2	-0.09029	5.98527012	0.62327114	0.705247769
SPAC25B8.03	psd2	phosphatidylserine decarboxylase Psd2	-0.0904224	7.85043304	0.34079311	0.435511362
SPAP8A3.04c	hsp9	heat shock protein Hsp9	-0.0906233	8.4850207	0.27107605	0.362696125
SPBC800.08	gcd10	tRNA (m1A) methyltransferase non-catalytic subunit Gcd10	-0.0912287	6.94511143	0.44697102	0.542858786
SPAC30C2.06c	dml1	mitochondrial inheritance GTPase, tubulin-like (predicted)	-0.0913513	6.42610658	0.55690571	0.646824264
SPAC1F7.09c	dal2	allantoicase Dal2	-0.09166	6.66488458	0.50855479	0.600922965
SPAC1527.01	mok11	alpha-1,3-glucan synthase Mok11	-0.0916656	8.41345842	0.31536447	0.409240131
SPCC4G3.09c	gyp3	GTPase activating protein Gyp3 (predicted)	-0.091964	7.66359299	0.31060638	0.404221921
SPNCRNA.607	#N/A	#N/A	-0.0920159	6.09750222	0.59146188	0.676260978
SPAPB1A10.09	ase1	antiparallel microtubule cross-linking factor Ase1	-0.0927622	7.67035932	0.31351419	0.407305093
SPBC56F2.05c	SPBC56F2.05c	transcription factor (predicted)	-0.0928416	5.42233861	0.61464986	0.697347835
SPBC839.09c	mrp21	mitochondrial ribosomal protein subunit Mrp21 (predicted)	-0.092875	5.15532164	0.71508736	0.783738047
SPAC6F6.01	cch1	plasma membrane calcium ion import channel Cch1	-0.0929146	8.02922693	0.29403854	0.386728313
SPAC22A12.03c	csn4	COP9/signalosome complex subunit Csn4	-0.0936652	5.64208559	0.63499383	0.714945904
SPBP23A10.02	pkr1	V-ATPase assembly factor Pkr1 (predicted)	-0.0939854	6.47593816	0.48491175	0.580035934
SPAC12G12.12	gms2	Golgi UDP-galactose transmembrane transporter Gms2 (pr	-0.0939983	6.74336759	0.44963883	0.545126
SPCC417.03	#N/A	#N/A	-0.0941231	3.1161803	0.92817148	0.954686652
ScpofMt31	#N/A	#N/A	-0.0941456	3.87588473	0.76158044	0.822589496
SPAC19G12.10c	cpy1	vacuolar carboxypeptidase Y	-0.0941723	11.127495	0.19067733	0.269875048
SPAC1039.05c	klf1	transcription factor, zf-fungal binuclear cluster type Klf1	-0.0942819	7.6685159	0.37260777	0.467822116
SPAC25B8.04c	mss51	mitochondrial Cox1 translation regulator Mss51 (predicted)	-0.094649	5.47661594	0.65469347	0.732520401
SPBC36.09	sap61	U2 snRNP-associated protein sap61	-0.0948948	7.15794382	0.3739594	0.469259397
SPBC887.15c	sur2	sphingosine hydroxylase/sphingolipid delta-4 desaturase ac	-0.0952691	8.09348489	0.44314754	0.539080984
SPBC16E9.01c	php4	CCAAT-binding factor complex subunit Php4	-0.0954846	6.09024968	0.57654661	0.664049896
SPAC17G6.03	SPAC17G6.03	extracellular 5'-nucleotidase, human NT5E family (predicte	-0.0955211	8.00146784	0.24649174	0.336012875
SPAC1D4.11c	lkh1	dual specificity protein kinase Lkh1	-0.095608	7.98448428	0.44829497	0.544271182
SPNCRNA.18	#N/A	#N/A	-0.0961207	5.83685191	0.60325006	0.686589177
SPAC24B11.12c	dnf2	plasma membrane phospholipid-translocating ATPase com	-0.0961988	9.86959417	0.21375642	0.297405943
SPBC106.20	exo70	exocyst complex subunit Exo70	-0.0963153	6.35411676	0.52338087	0.614918304
SPCC126.13c	sap18	splicing factor Sap18 (predicted)	-0.0963648	5.41838629	0.63089659	0.711274234
SPNCRNA.53	#N/A	#N/A	-0.0963701	10.0539495	0.15587356	0.228316411
SPACUNK4.19	mug153	Schizosaccharomyces pombe specific protein	-0.0966404	3.65986927	0.8137707	0.864456701
SPNCRNA.910	#N/A	#N/A	-0.0968713	5.42615156	0.64446138	0.723570321
SPAC13G7.07	arb2	argonaute binding protein 2	-0.0969446	4.59564875	0.75015525	0.814293588
SPAC688.07c	rng10	coiled-coil protein involved in septum formation Rng10	-0.0969813	8.5467388	0.22884552	0.315077815
SPNCRNA.1552	#N/A	#N/A	-0.0969919	6.0164246	0.51683646	0.608034361
SPNCRNA.1518	#N/A	#N/A	-0.0970248	7.12642177	0.4114177	0.506549208
SPBC1604.07	atp4	F1-FO ATP synthase subunit (predicted)	-0.0972082	8.01065541	0.25705296	0.347417158
SPBC18A7.01	xpa1	X-Pro dipeptidase (predicted)	-0.0972889	7.093445	0.40218687	0.497729105
SPNCRNA.1232	#N/A	#N/A	-0.097365	4.25367129	0.74466419	0.809729744
SPAC27E2.11c	SPAC27E2.11c	Schizosaccharomyces specific protein	-0.0974018	10.0582665	0.14999234	0.221169641
SPNCRNA.1451	#N/A	#N/A	-0.0974669	8.22266398	0.28682543	0.379810673
SPAC29A4.14c	pex3	peroxin-3 peroxisome import protein Pex3 (predicted)	-0.0978165	7.01643462	0.39036965	0.48582031
SPNCRNA.1357	#N/A	#N/A	-0.0978323	6.68681722	0.46181361	0.556909727
SPNCRNA.1288	#N/A	#N/A	-0.0978628	4.99401885	0.68282354	0.758012106
SPAC15A10.10	mde6	Muskelin homolog, kelch repeat, expressed during meiotic (	-0.0982121	8.39921532	0.24423422	0.333491457
SPAC6C3.06c	neo1	Golgi membrane phospholipid translocase (flippase) Neo1 (	-0.0982423	8.18164175	0.2306727	0.317184519

SPBC1703.06	pof10	F-box/WD repeat protein Pof10	-0.0983817	7.16882638	0.50934486	0.601369707
SPNCRNA.1563	#N/A	#N/A	-0.0983817	7.16882638	0.50934486	0.601369707
SPAC630.13c	tsc2	tuberin, GTPase activator Tsc2	-0.0984076	7.84893108	0.33859751	0.433030649
SPCC16C4.04	SPCC16C4.04	Schizosaccharomyces specific protein	-0.0984661	4.33153088	0.75454056	0.817934271
SPNCRNA.886	#N/A	#N/A	-0.0986192	4.49124192	0.73192977	0.799171801
SPAC26A3.14c	SPAC26A3.14c	DUF1748 family protein	-0.0986197	5.38974161	0.67962266	0.754951391
SPBC887.02	SPBC887.02	ClC chloride channel (predicted)	-0.0987418	7.76448843	0.28159119	0.374393479
SPAC29A4.13	ure6	urease accessory protein UreF	-0.0990674	6.25805591	0.53866884	0.629859198
SPAC57A10.09c	nhp6	High-mobility group non-histone chromatin protein (predict	-0.0993552	7.50205778	0.37095827	0.466267279
SPAC23H4.07c	srp102	signal recognition particle receptor beta subunit Srp102 (pr	-0.0995259	6.30833434	0.46610859	0.560863387
SPBC17F3.01c	rga5	RhoGAP, GTPase activating protein Rga5	-0.0997278	7.0947524	0.41176657	0.506887028
SPBC1709.20	pop8	RNase P and RNase MRP subunit Pop8	-0.0998241	3.83104067	0.84735907	0.893581041
SPAC23A1.18c	mrp51	mitochondrial ribosomal protein subunit L51-b (predicted)	-0.0999735	6.77061685	0.4124944	0.507599345
SPCC1827.02c	pcy1	cholinephosphate cytidylyltransferase Pcy1 (predicted)	-0.1002142	7.46039969	0.34672891	0.441686671
SPAC6F12.12	par2	protein phosphatase PP2A regulatory subunit B-56 Par2	-0.1002412	7.04438547	0.40680016	0.502135879
SPBC21B10.02	SPBC21B10.02	conserved fungal protein	-0.1006144	6.37835993	0.44051143	0.536449588
SPNCRNA.1646	#N/A	#N/A	-0.1008363	4.82716693	0.67241553	0.74841146
SPAC1B3.11c	ypt4	GTPase Ypt4	-0.1009922	6.21226928	0.47267931	0.567472603
SPBC32F12.15	tfb5	transcription factor TFIIH complex subunit Tfb5 (predicted)	-0.1012676	6.0184191	0.49478705	0.588949779
SPAC4A8.03c	ptc4	protein phosphatase 2C Ptc4	-0.1014096	6.40408459	0.44510519	0.541075529
SPAC20G8.09c	nat10	rRNA/tRNA cytidine N-acetyltransferase	-0.1014177	8.11900895	0.25345028	0.343640001
SPNCRNA.555	#N/A	#N/A	-0.1014398	6.10777607	0.49467184	0.588915655
SPNCRNA.1003	#N/A	#N/A	-0.1020451	5.81044133	0.559525	0.649333932
SPBC16A3.11	eso1	mitotic cohesin N-acetyltransferase/DNA polymerase eta E	-0.1022272	7.41601359	0.38000301	0.475527853
SPAC343.18	rfp2	SUMO-targeted ubiquitin-protein ligase subunit Rfp2	-0.1023653	5.56099389	0.58530573	0.671425542
SPBC3H7.05c	SPBC3H7.05c	mitochondrial Membrane Bound O-Acyl Transferase (MBO/	-0.102599	7.78015213	0.35437734	0.449913767
SPBC3B8.05	dph1	diphthamide biosynthesis protein Dph1 (predicted)	-0.1026959	6.09294569	0.57364318	0.66141
SPAC27E2.06c	msm1	mitochondrial methionine-tRNA ligase Msm1 (predicted)	-0.1027268	6.55806806	0.46135576	0.556495253
SPAC637.12c	mst1	KAT5 family histone acetyltransferase Mst1	-0.102825	7.17315471	0.32551548	0.420010016
SPBP4H10.17c	SPBP4H10.17c	protein repair carboxyl methyl esterase (predicted)	-0.1030393	6.92331635	0.37121338	0.466501767
SPBC543.07	pek1	MAP kinase kinase Pek1	-0.1032902	6.83814337	0.36802427	0.463349701
SPAC23G3.10c	ssr3	SWI/SNF and RSC complex subunit Ssr3	-0.1036709	8.08449346	0.24770597	0.337210122
SPCC1020.12c	xap5	xap-5-like protein	-0.1037789	5.31229892	0.65724307	0.735066081
SPAC688.11	end4	Clathrin adaptor End4	-0.1047611	9.07978308	0.18957545	0.268538908
SPNCRNA.1479	#N/A	#N/A	-0.1049496	4.64699257	0.74286598	0.808315158
SPACUNK4.06c	rpb7	DNA-directed RNA polymerase complex II subunit Rpb7	-0.1052987	6.95463864	0.36378297	0.459284437
SPAC20G4.08	pdc1	P-body assembly protein	-0.1054512	8.26035687	0.21536526	0.299460686
SPAC644.12	cdc5	Prp19 complex subunit Cdc5	-0.1055369	8.14308666	0.22814469	0.314339868
SPBC947.07	rrp1402	ribosome biogenesis protein Rrp14 (predicted)	-0.1057914	5.52456735	0.54687182	0.637127676
SPAPB1A11.03	SPAPB1A11.03	cytochrome b2 (L-lactate cytochrome-c oxidoreductase) (pr	-0.1060146	5.40927077	0.58242649	0.669043258
SPAC31A2.03	mrp11	mitochondrial ribosomal protein subunit L11 (predicted)	-0.1060688	6.54228058	0.46011661	0.555256879
SPBC577.04	tho5	human THOC5 ortholog Tho5 (predicted)	-0.1064063	4.98265381	0.63397656	0.714155021
SPBC19F8.04c	lcl3	mitochondrial nuclease Lcl3, implicated in DNA repair (prec	-0.1066243	5.26059399	0.61078008	0.693413258
SPNCRNA.1589	#N/A	#N/A	-0.1066243	5.26059399	0.61078008	0.693413258
SPBC3D6.07	gpi3	pig-A, phosphatidylinositol N-acetylglucosaminyltransferas	-0.1067183	5.98609168	0.52139136	0.612792434
SPCC16C4.15	rml2	mitochondrial ribosomal protein subunit L2 (predicted)	-0.1072599	7.13904644	0.34150721	0.436178032
SPAC23G3.08c	ubp7	ubiquitin C-terminal hydrolase Ubp7	-0.1085877	7.71326787	0.223515	0.308711097

SPBC16C6.09	ogm4	protein O-mannosyltransferase Ogm4	-0.1094013	8.30634958	0.15587921	0.228316411
SPAC6B12.06c	rrg9	mitochondrial genome maintenance protein Rrg9 (predicte	-0.1096252	5.29735136	0.6186943	0.70111819
SPAPB8E5.02c	rpn502	19S proteasome regulatory subunit Rpn502	-0.1097284	8.64943498	0.20156181	0.282344197
SPNCRNA.1484	#N/A	#N/A	-0.1100322	6.33902427	0.45751849	0.552611522
SPBC31E1.03	hub1	ubiquitin-like protein modifier Hub1	-0.1104982	5.70813746	0.51147006	0.603111027
SPBC530.10c	anc1	mitochondrial carrier, ATP:ADP antiporter Anc1	-0.1106254	9.03204623	0.20330768	0.284263156
SPAC2F3.06c	kap104	karyopherin/importin beta family nuclear import signal rece	-0.1106852	8.38008509	0.18638029	0.264508419
SPBC21H7.04	dbp7	ATP-dependent RNA helicase Dbp7 (predicted)	-0.110756	6.71892941	0.37234085	0.467573254
SPAC3F10.16c	SPAC3F10.16c	GTP binding protein, HSR1-related (predicted)	-0.1109881	7.22453984	0.26925935	0.360690915
SPBP8B7.20c	nop2	rRNA (cytosine-C5-)-methyltransferase activity Nop2 (predi	-0.111018	9.54056881	0.18519716	0.263103685
SPNCRNA.1131	#N/A	#N/A	-0.1110699	6.26993705	0.42289	0.518061553
SPBC839.12	rpc31	DNA-directed RNA polymerase III complex subunit Rpc31	-0.1110889	6.49825294	0.41206033	0.507156911
SPNCRNA.728	#N/A	#N/A	-0.1112457	6.3355512	0.42602303	0.520949993
SPBC216.05	rad3	ATR checkpoint kinase Rad3	-0.1115597	9.49802907	0.14839099	0.21914078
SPNCRNA.1521	#N/A	#N/A	-0.1116103	5.31529747	0.57197752	0.660048672
SPAC26F1.10c	pyp1	tyrosine phosphatase Pyp1	-0.111695	7.41937088	0.32049567	0.414949205
SPBC1105.11c	hht3	histone H3 h3.3	-0.1117265	7.70137819	0.22173996	0.306508318
SPAC26F1.09	gyp51	GTPase activating protein Gyp51 (predicted)	-0.1119586	7.56903762	0.33828307	0.432872562
SPBC31F10.03	ggg1	glutathione-specific gamma-glutamylcyclotransferase Ggg1	-0.1126597	7.96358484	0.23456467	0.321946874
SPNCRNA.888	#N/A	#N/A	-0.1140255	7.40438228	0.28299596	0.375836745
SPBC582.04c	dsh1	RNAi protein, Dsh1	-0.1141349	6.57903846	0.36179737	0.457371564
SPNCRNA.934	#N/A	#N/A	-0.1146289	7.99572622	0.19965039	0.280295268
SPNCRNA.858	#N/A	#N/A	-0.1147883	7.38702224	0.28375912	0.376703245
SPAC664.06	rlp7	ribosomal protein L7-like Rlp7 involved in ribosome biogene	-0.1148006	7.48178859	0.32900607	0.4237105
SPBC577.10	pre4	20S proteasome complex subunit beta 7, Pre4	-0.1149318	8.0165919	0.1813076	0.259038048
SPBC405.04c	ypt7	GTPase Ypt7	-0.1149834	7.92143742	0.18999832	0.268969956
SPNCRNA.1401	#N/A	#N/A	-0.1151508	5.37702315	0.56158116	0.651032329
SPCC965.10	SPCC965.10	transcription factor (predicted)	-0.1151796	7.91499545	0.18363943	0.261819887
SPBC2D10.19c	alb1	pre-60S shuttling factor Alb1 (predicted)	-0.1152056	5.55688554	0.54725386	0.637463623
SPAC29A4.03c	mrps9	mitochondrial ribosomal protein subunit Mrps9 (predicted)	-0.1153077	7.24300688	0.28637395	0.379360466
SPBC1734.09	yea4	ER UDP-N-acetylglucosamine transmembrane transporter (	-0.1156015	7.60149387	0.21801037	0.302397176
SPCC962.03c	cut15	importin alpha family nuclear import signal receptor adapto	-0.1156275	7.81361161	0.23483486	0.322187743
SPBC947.06c	SPBC947.06c	spermidine family transmembrane transporter (predicted)	-0.1163039	7.33325501	0.23301395	0.319947524
SPCC338.13	cog4	Golgi transport complex subunit Cog4 (predicted)	-0.1164829	6.6212428	0.34120224	0.43587033
SPAC13G6.11c	erg12	mevalonate kinase Erg12 (predicted)	-0.1165773	7.14870961	0.50299028	0.596419031
SPBC31E1.01c	atg2	autophagy associated protein Atg2	-0.116643	7.87771701	0.22859932	0.314838773
SPBC16A3.01	spn3	mitotic septin Spn3	-0.1168485	7.13186738	0.27044709	0.362068151
SPNCRNA.1379	#N/A	#N/A	-0.1169995	4.65086666	0.69166015	0.765449229
SPAC2F7.11	nrd1	RNA-binding protein Nrd1	-0.1173208	6.03296022	0.50625773	0.599438613
SPAC7D4.05	SPAC7D4.05	HAD superfamily hydrolase, unknown role	-0.117384	6.03385041	0.44837388	0.544271182
SPNCRNA.1557	#N/A	#N/A	-0.1180349	6.76219962	0.31486796	0.408829707
SPBC1734.10c	ipa1	cleavage and polyadenylation HECT-type ubiquitin-protein li	-0.1182231	7.58517033	0.2062974	0.28814733
SPAC17A2.02c	SPAC17A2.02c	ER protein, DUF887 family protein, implicated in lipid meta	-0.1189884	7.7200068	0.1829943	0.261064195
SPCC24B10.10c	yta4	mitochondrial outer membrane ATPase Msp1/Yta4 (predict	-0.1192891	6.54671264	0.36403605	0.459433477
SPAC222.12c	atp2	F1-FO ATP synthase beta subunit Atp2	-0.1206922	10.0475045	0.05688369	0.096652564
SPBC2G2.02	syj1	inositol-polyphosphate 5-phosphatase, synaptojanin homolo	-0.1207396	7.5112081	0.19346835	0.273256976
SPBC1347.12	arp1	dynactin complex subunit, centractin family actin-like prote	-0.1207663	3.92783065	0.70829653	0.778291275

SPBC19G7.16	iws1	transcription elongation factor complex subunit Iws1 (predi	-0.1211989	6.67471397	0.32932395	0.423799069
SPNCRNA.1508	#N/A	#N/A	-0.1211989	6.67471397	0.32932395	0.423799069
SPAC1805.18	pop6	RNase P and RNase MRP subunit Pop6/Pop7 ortholog	-0.1212126	4.85370665	0.61742773	0.70014926
SPBC11B10.04c	mrps28	mitochondrial ribosomal protein subunit S28 (predicted)	-0.1218162	6.76642019	0.29872409	0.391679662
SPBC21.02	rtc5	TLDc domain protein 2	-0.1219892	6.42157372	0.43568883	0.531337359
SPAC13F5.01c	msh1	mitochondrial MutS protein Msh1 (predicted)	-0.1222375	6.323795	0.39164801	0.487054959
SPAC56F8.02	SPAC56F8.02	AMP binding enzyme, human DIP2 family (predicted)	-0.1224535	8.0852964	0.17964155	0.257089532
SPAC19B12.10	sst2	human AMSH/STAMBP protein homolog, ubiquitin specific-	-0.1229055	7.21767377	0.26264713	0.353573433
SPNCRNA.935	#N/A	#N/A	-0.1230499	8.06230283	0.18451727	0.262466558
SPAC824.08	gda1	Golgi nucleoside-diphosphatase Gda1	-0.1231875	8.47663012	0.13247131	0.199483789
SPBC25D12.06	SPBC25D12.06	mitochondrial ATP-dependent RNA helicase (predicted)	-0.1234615	7.12990664	0.29839057	0.391393186
SPBC365.02c	cox10	protoheme IX farnesyltransferase (predicted)	-0.123501	5.98457366	0.48768237	0.582735475
SPAC664.07c	rad9	checkpoint clamp complex protein Rad9	-0.1240819	4.93885009	0.58814727	0.67406613
SPAC3H1.10	pcs2	phytochelatin synthetase	-0.1252062	6.42532192	0.40037492	0.495823717
SPNCRNA.1338	#N/A	#N/A	-0.1253265	4.26771763	0.7004704	0.771935399
SPAC3C7.10	pex13	peroxin 13 (predicted)	-0.1253802	6.46230223	0.34739991	0.442458615
SPAC56E4.04c	cut6	acetyl-CoA/biotin carboxylase	-0.1254088	10.478432	0.07529876	0.122791288
SPAC3C7.01c	sac12	inositol polyphosphate phosphatase (predicted)	-0.1254266	7.58615636	0.20749614	0.289643333
SPBC23E6.04c	utp10	U3 snoRNP-associated protein Utp10 (predicted)	-0.1254481	8.83572134	0.12375145	0.188185168
SPBC27B12.05	SPBC27B12.05	WD repeat protein involved in transcriptional regulation (pr	-0.1256265	6.01998603	0.44885642	0.544565509
SPBC16A3.16	coa5	cytochrome c oxidase assembly protein Coa5 (predicted)	-0.1257456	6.20651689	0.45036321	0.545695988
SPAC4G8.08	SPAC4G8.08	mitochondrial carrier, iron ion (predicted)	-0.1264917	6.33531262	0.36849379	0.463718697
SPAC17A5.19	SPAC17A5.19	Schizosaccharomyces specific protein	-0.1265299	4.24305459	0.75782363	0.82026241
SPNCRNA.1403	#N/A	#N/A	-0.1270711	5.71886598	0.45436906	0.549294984
SPBC776.12c	hsk1	Dbf4(Dfp1)-dependent protein kinase Hsk1	-0.1272787	5.90614838	0.41669812	0.511754319
SPAC17A2.03c	vma6	V-type ATPase V0 subunit d (predicted)	-0.1283814	7.77078692	0.16963184	0.244927784
SPNCRNA.453	#N/A	#N/A	-0.128729	6.45534486	0.33070627	0.425175927
SPAC5D6.12	mtf2	mitochondrial translation protein Mtf2 (predicted)	-0.1288204	6.0544117	0.41078562	0.505954055
SPBC3H7.08c	SPBC3H7.08c	mitochondrial conserved fungal membrane protein	-0.1292414	5.78467971	0.45952239	0.554638141
SPBC24C6.07	cdc14	SIN component Cdc14	-0.1296507	6.86977175	0.25923752	0.349782135
SPAC13G6.05c	trs33	TRAPP complex subunit Trs33 (predicted)	-0.1299647	6.20639601	0.43308551	0.528703004
SPAPB1E7.08c	SPAPB1E7.08c	transmembrane transporter (predicted)	-0.1299774	7.05354589	0.29128298	0.383845986
SPAC24C9.06c	aco1	aconitate hydratase Aco1 (predicted)	-0.1300832	10.3821488	0.05938216	0.100271852
SPAC1F5.07c	hem14	protoporphyrinogen oxidase Hem14 (predicted)	-0.1301231	7.15588974	0.28392275	0.37677346
SPAC6F12.17	rna14	mRNA cleavage and polyadenylation specificity factor comp	-0.1302018	7.85902208	0.19264227	0.272259746
SPAC15E1.06	vps29	retromer complex subunit Vps29	-0.1307035	6.5673595	0.29377058	0.386523883
SPAC343.07	mug28	RNA-binding protein Mug28	-0.1308379	6.86579634	0.30133962	0.394349258
SPCC737.02c	qcr7	ubiquinol-cytochrome-c reductase complex subunit 6 (predi	-0.1309609	7.52293438	0.18436214	0.262456954
SPBC29B5.04c	SPBC29B5.04c	phosphatidate phosphatase converting phosphatidate to dia	-0.1312421	7.38517797	0.18499019	0.2629676
SPAC19B12.03	bgs3	cell wall 1,3-beta-glucan synthase catalytic subunit Bgs3	-0.1320685	9.49401107	0.09481286	0.150046862
SPAC630.12	SPAC630.12	GPI-remodelling mannose-ethanolamine phosphate phosph	-0.1322803	6.6486912	0.27769792	0.369811031
SPCC320.13c	ark1	aurora-B kinase Ark1	-0.1328165	5.67483442	0.50451709	0.597960599
SPAC227.01c	erd1	Erd1 homolog (predicted)	-0.1330879	5.83624455	0.44250892	0.538496637
SPBC25B2.10	SPBC25B2.10	Usp (universal stress protein) family protein	-0.133329	6.92623583	0.33442349	0.428739989
SPAC24B11.05	SPAC24B11.05	pyrimidine 5'-nucleotidase (predicted)	-0.1334892	7.06481166	0.24528602	0.334634767
SPAC824.02	bst1	GPI inositol deacylase Bst1 (predicted)	-0.1337043	8.80756217	0.12527455	0.19016135
SPCC1682.11c	ctl1	choline transporter-like, implicated in autophagy Ctl1	-0.1337218	6.83628268	0.26620833	0.357307239

SPCC364.01	cif1	calnexin independence factor Cif1	-0.1343508	5.39447617	0.57015119	0.658946812
SPBC32F12.01c	css1	inositol phosphosphingolipid phospholipase C, Css1	-0.1344872	7.03152173	0.28785261	0.380513323
SPBP8B7.05c	nce103	carbonic anhydrase (predicted)	-0.1346793	6.85294203	0.27303394	0.364670456
SPAC630.08c	erg25	C-4 methylsterol oxidase Erg25 (predicted)	-0.1348273	8.25085189	0.25519683	0.345388712
SPBC725.10	tps0	mitochondrial lipid translocator protein, tspO	-0.1348675	8.14066049	0.13587249	0.203569416
SPAC1B3.18c	mrps18	mitochondrial ribosomal protein subunit S18 (predicted)	-0.135111	6.0204398	0.41537777	0.510409122
SPBP23A10.15c	qcr1	mitochondrial processing peptidase (MPP) complex beta su	-0.1351115	9.1723524	0.09114368	0.145185561
SPBC1271.12	kes1	sterol intermembrane transfer protein Kes1 (predicted)	-0.1352874	8.37854139	0.10173814	0.159229072
SPNCRNA.1323	#N/A	#N/A	-0.1353553	7.45021508	0.15361135	0.225431367
SPAC17G8.02	urh2	uridine ribohydrolase Urh2 (predicted)	-0.1358307	6.1389321	0.39408096	0.489454444
SPNCRNA.515	#N/A	#N/A	-0.1359135	5.49245561	0.45209365	0.547322052
SPBC28F2.10c	ngg1	SAGA complex subunit Ngg1/Ada3	-0.1362467	6.97777719	0.26302335	0.353939866
SPAC823.06	taf3	transcription factor TFIID complex subunit Taf3 (predicted)	-0.1365794	4.66148848	0.59901895	0.68261529
SPBC336.02	cdh1	18S rRNA dimethylase Cdh1 (predicted)	-0.1369204	6.48713981	0.2883927	0.380996371
SPBC660.10	mef2	mitochondrial translation elongation factor G Mef2 (predict	-0.1374435	6.88870343	0.27295197	0.364670456
SPNCRNA.491	#N/A	#N/A	-0.1380806	5.80022425	0.37030686	0.465620502
SPBC887.13c	cem1	3-oxoacyl-[acyl-carrier-protein]-synthase condensing enzym	-0.1380941	6.53889856	0.36333185	0.458800012
SPNCRNA.659	#N/A	#N/A	-0.1381509	5.97020921	0.49938489	0.592962969
SPAC664.08c	bfr2	traub family protein involved in ribosome biogenesis (predi	-0.1382325	6.27330656	0.35504605	0.450426616
SPAC29A4.17c	SPAC29A4.17c	mitochondrial FUN14 family protein involved in mitophagy	-0.138874	5.76077697	0.45368523	0.548565736
SPAC15A10.05c	mug182	NADHX epimerase (predicted)	-0.1389041	5.98014547	0.4181974	0.513317698
SPAC1002.18	urg3	DUF1688 family fungal conserved protein, implicated in ura	-0.1389407	6.09900554	0.43719283	0.532789609
SPNCRNA.1083	#N/A	#N/A	-0.1389962	5.66814746	0.4404189	0.536432905
SPCC1620.14c	snf22	ATP-dependent DNA helicase Snf22	-0.1393561	8.8145688	0.05817822	0.098556834
SPAC29B12.02c	set2	histone lysine methyltransferase Set2	-0.1395241	7.56392388	0.15771603	0.230708847
SPCC1259.13	chk1	Chk1 protein kinase	-0.1396644	7.07423038	0.18196843	0.259764037
SPBC530.05	prt1	transcription factor (predicted)	-0.1398045	7.25623893	0.19717855	0.277575512
SPBC4B4.02c	nca2	mitochondrial protein Nca2 (predicted)	-0.1398326	6.80084609	0.36001299	0.455708411
SPBC16E9.11c	pub3	HECT-type ubiquitin-protein ligase E3 Pub3 (predicted)	-0.1400662	8.66941245	0.11001314	0.17006802
SPAC1420.03	rpn501	19S proteasome regulatory subunit Rpn501	-0.1401055	8.58084226	0.11234186	0.172999861
SPBC16A3.17c	SPBC16A3.17c	transmembrane transporter (predicted)	-0.1402886	7.39761525	0.18447195	0.262456954
SPNCRNA.808	#N/A	#N/A	-0.1404655	5.89202164	0.36519726	0.460642698
SPAPB1A11.02	SPAPB1A11.02	esterase/lipase (predicted)	-0.1405898	2.94617388	0.82874455	0.876940861
SPBC24C6.08c	bhd1	Lst4-Lst7 complex subunit, folliculin Bhd1/Lst7	-0.1407462	6.87007028	0.22642327	0.312157686
SPBC19C2.01	cdc28	ATP-dependent RNA helicase Cdc28	-0.1408646	7.91263114	0.15283509	0.224631266
SPNCRNA.1217	#N/A	#N/A	-0.1410144	7.7638809	0.11852521	0.181495059
SPCC31H12.06	mug111	major facilitator family transmembrane transporter Mug11	-0.1411066	4.50215137	0.72711854	0.795358042
SPCC16C4.19	rpp21	RNase MRP subunit Rpp21	-0.1413785	5.89656177	0.3599352	0.455694705
SPAC17H9.09c	ras1	GTPase Ras1	-0.1420159	6.50283282	0.36722919	0.462605454
SPBC342.04	rpn1301	19S proteasome regulatory subunit Rpn13a	-0.14216	6.02282477	0.41743812	0.51257062
SPAC144.16	cia2	CIA machinery protein Cia2 (predicted)	-0.1422307	7.67391542	0.24771689	0.337210122
SPNCRNA.732	#N/A	#N/A	-0.1425618	4.91685358	0.54424751	0.635048748
SPBC3B9.10	vti1	SNARE Vti1 (predicted)	-0.1428377	7.17275992	0.1965083	0.276975756
SPCC1620.03	mug163	mitochondrial protein, human C6orf136 ortholog	-0.1429055	6.13882825	0.39472287	0.490072823
SPNCRNA.850	#N/A	#N/A	-0.14307	6.83375909	0.2362052	0.323871926
SPNCRNA.926	#N/A	#N/A	-0.1430921	6.00218154	0.40060043	0.496012727
SPAC1296.04	mug65	dysferlin-like membrane trafficking protein Mug65 (predicte	-0.1432823	5.28354103	0.480733	0.575782588

SPAC22E12.09c	krp1	kexin	-0.1433878	8.95658699	0.05067743	0.087772955
SPCC1223.10c	eaf1	RNA polymerase II transcription elongation factor Eaf1	-0.1434185	6.39682989	0.38711332	0.482719965
SPBC582.10c	SPBC582.10c	ATP-dependent DNA helicase Rhp16b (predicted)	-0.143431	6.29435533	0.32568449	0.420148423
SPBC30B4.01c	wsc1	plasma membrane-associated serine-rich cell wall sensor W	-0.143602	7.65601581	0.1983492	0.278877338
SPCC1620.08	lsc2	succinate-CoA ligase beta subunit Lsc2 (predicted)	-0.1440395	7.58993489	0.16456743	0.238933513
ScpofMt26	#N/A	#N/A	-0.1442879	3.8311352	0.75971618	0.821485666
SPAP27G11.10c	nup184	nucleoporin Nup184	-0.1452854	8.72745338	0.07800775	0.126481322
SPAC16.04	dus3	tRNA dihydrouridine synthase Dus3 (predicted)	-0.1454622	7.20741808	0.17666725	0.253460842
SPAC869.11	cat1	plasma membrane arginine/lysine amino acid transmembr	-0.1463798	6.71286067	0.30069928	0.393662682
SPAC31G5.09c	spk1	MAP kinase Spk1	-0.1468148	8.0959238	0.07065242	0.116188915
SPNCRNA.14	#N/A	#N/A	-0.1468794	5.50939747	0.47391892	0.568786282
SPBC902.03	SPBC902.03	Nem1-Spo7 complex regulatory subunit (predicted)	-0.1469373	4.55678896	0.71509618	0.783738047
SPBC27B12.04c	far11	SIP/FAR complex subunit, Far11/Csc2	-0.1470674	6.28898748	0.33639409	0.430860488
SPCC4B3.01	tum1	thiosulfate sulfurtransferase, involved in tRNA wobble posi	-0.1472862	6.89757955	0.29302107	0.385762886
SPNCRNA.1685	#N/A	#N/A	-0.1473407	6.46733393	0.32482972	0.41936374
SPAC3A12.05c	taf2	TATA-binding protein associated factor Taf2 (predicted)	-0.1475946	8.17973589	0.07791913	0.12639802
SPBC342.05	crb2	DNA repair protein Rad9 homolog Crb2	-0.1479653	6.22362949	0.28796144	0.380574407
SPAC22A12.02c	mug103	Schizosaccharomyces specific protein Mug103	-0.1481141	2.16917366	0.87430464	0.914626145
SPAC11E3.11c	syt22	guanyl-nucleotide exchange factor Syt22	-0.148135	7.95795261	0.09956184	0.156434614
SPAC3C7.06c	pit1	serine/threonine protein kinase, meiotic Pit1	-0.1482747	6.44828263	0.3171525	0.411325094
SPBP4H10.08	qcr10	Reiske ISP-associated protein, ubiquinol-cytochrome-c redu	-0.1485081	5.67788946	0.45133668	0.546600121
SPAC4H3.13	pcc1	EKC/KEOPS complex subunit Pcc1 (predicted)	-0.1488763	5.49955897	0.38720077	0.482719965
SPBC28F2.12	rpb1	RNA polymerase II large subunit Rpb1	-0.1489853	9.37551785	0.04210948	0.074799016
SPAC16E8.17c	SPAC16E8.17c	succinate-CoA ligase alpha subunit (predicted)	-0.1491303	6.51500174	0.29689433	0.38999401
SPBPB2B2.18	SPBPB2B2.18	conserved fungal plasma membrane protein	-0.149497	8.24977168	0.11998871	0.183447128
SPBC646.15c	pex16	Pex16 family peroxisome import protein Pex16 (predicted)	-0.1496215	5.87481957	0.35983143	0.455694705
SPCC1020.08	tyw1	wybutosine biosynthesis protein Tyw1 (predicted)	-0.1499003	7.87431673	0.08831763	0.141312357
SPAC167.01	ire1	serine/threonine protein kinase, sensor for unfolded protei	-0.1499975	6.80539402	0.22267584	0.30773946
SPAC30.03c	tsn1	translin	-0.150243	6.29402798	0.36157196	0.45717153
SPAC31A2.08	mrp20	mitochondrial ribosomal protein subunit L23 (predicted)	-0.1502681	6.31388381	0.29268808	0.385459805
SPAC19D5.04	ptr1	HECT-type ubiquitin-protein ligase E3 Ptr1	-0.1505	10.1369207	0.03521485	0.064022725
SPAPB18E9.02c	ppk18	greatwall kinase Ppk18	-0.1506355	8.63621879	0.04420331	0.077787309
SPNCRNA.507	#N/A	#N/A	-0.1509072	5.88697568	0.33057038	0.425081528
SPNCRNA.893	#N/A	#N/A	-0.1510322	5.048125	0.47949778	0.574569889
SPNCRNA.543	#N/A	#N/A	-0.1516152	1.77654745	1	1
SPBC1773.09c	mug184	DNAJ domain protein, exocytosis associated Mug184 (predi	-0.1516524	6.25728065	0.28179631	0.374462778
SPBP8B7.02	rng9	contractile ring myosin V regulator Rng9	-0.1518173	4.8703231	0.50564261	0.598937856
SPNCRNA.1627	#N/A	#N/A	-0.1518173	4.8703231	0.50564261	0.598937856
SPAC23C4.07	tht2	karyogamy protein Tht2	-0.1520141	5.5057726	0.50632963	0.599438613
SPAC26A3.09c	rga2	RhoGAP, GTPase activating protein Rga2	-0.1520643	7.58626959	0.10243421	0.160024053
SPAC27F1.09c	prp10	U2 snRNP-associated protein Sap155	-0.1521613	7.81469815	0.14461306	0.214338578
SPNCRNA.1273	#N/A	#N/A	-0.1521695	6.66302328	0.30681474	0.4003594
SPAC24C9.15c	spn5	meiotic septin Spn5	-0.1521917	5.9086	0.33213787	0.426558521
SPAC24C9.16c	cox8	cytochrome c oxidase subunit VIII (predicted)	-0.1522197	5.24832581	0.47417948	0.568998656
SPAC22F3.04	mug62	AMP binding enzyme, human DIP2 family (predicted)	-0.15233	7.37490576	0.15353482	0.225367661
SPNCRNA.1670	#N/A	#N/A	-0.1523582	1.09884873	1	1
SPNCRNA.200	#N/A	#N/A	-0.1523582	1.09884873	1	1

SPNCRNA.675	#N/A	#N/A	-0.1530478	5.92811157	0.41422384	0.509134803
SPCC569.03	SPCC569.03	mug2/mug135/meu2 family	-0.1530845	6.94996401	0.35385568	0.449419173
SPNCRNA.1548	#N/A	#N/A	-0.1531983	6.35147425	0.40903644	0.504255969
SPNCRNA.748	#N/A	#N/A	-0.153326	5.44726925	0.39867348	0.493986351
SPBC725.09c	hob3	BAR adaptor protein Hob3	-0.153446	8.21035774	0.05591385	0.095290187
SPBC28F2.08c	hrd3	Hrd1 ubiquitin ligase complex subunit (predicted)	-0.1541881	7.9496552	0.10171699	0.159229072
SPBC21.03c	SPBC21.03c	EVE domain (PUA-related) protein, implicated in tRNA met	-0.1546104	6.44511494	0.32303486	0.417284019
SPCC23B6.05c	ssb3	DNA replication factor A subunit Ssb3	-0.1546996	3.97747905	0.65102389	0.729494085
SPAC343.08c	mrp17	mitochondrial ribosomal protein subunit Mrp17 (predicted)	-0.1547475	7.23381209	0.14525403	0.215115056
SPCC320.08	SPCC320.08	transmembrane transporter (predicted)	-0.1548409	7.69772004	0.1339322	0.201327287
SPAC3F10.02c	trk1	plasma membrane potassium ion transmembrane transpor	-0.1549033	7.7098894	0.0846669	0.135886387
SPCC962.01	tcb1	tricalbin, C2 domain protein (phospholipid binding) ER-plası	-0.1549426	9.47419935	0.02093122	0.04075449
SPBC29A10.13	atp7	F1-FO ATP synthase subunit D (predicted)	-0.1550497	7.44136235	0.12883174	0.194605996
SPNCRNA.785	#N/A	#N/A	-0.1551049	6.44167252	0.35148434	0.446824379
SPBC32F12.17	#N/A	#N/A	-0.1554178	5.04598528	0.55463021	0.644401324
SPCC24B10.07	gad8	AGC family protein kinase Gad8	-0.1555553	7.71637307	0.10104138	0.158357112
SPBC336.13c	mmp2	mitochondrial inner membrane peptidase complex catalytic	-0.1557302	6.37571478	0.40966298	0.504936896
SPBC354.03	swd3	WD repeat protein Swd3	-0.1557833	6.41465673	0.27655981	0.368656128
SPBC21B10.11	dpm2	dolichol-phosphate mannosyltransferase regulatory subunit	-0.156189	4.9057713	0.50937472	0.601369707
SPAC18G6.09c	edc1	Dcp2-Dcp1 mRNA-decapping complex subunit Edc1	-0.1561965	8.15486407	0.09552595	0.150964726
SPBC609.05	pob3	histone H2A-H2B chaperone, FACT complex subunit Pob3	-0.1562554	7.70544114	0.15164514	0.223219801
SPAC25B8.09	SPAC25B8.09	trans-aconitate 3-methyltransferase (predicted)	-0.1562865	6.79458923	0.2000238	0.280594095
SPNCRNA.567	#N/A	#N/A	-0.1571349	6.73019881	0.19745805	0.277854018
SPCC576.13	swc5	Swr1 complex subunit Swc5	-0.1572601	6.24830645	0.37206909	0.467318228
SPCC61.02	spt3	SAGA complex subunit Spt3	-0.1572741	6.73164924	0.23067541	0.317184519
SPNCRNA.998	#N/A	#N/A	-0.1573175	7.96099543	0.0757327	0.123469339
SPBC16A3.09c	ufd1	Hrd1 ubiquitin ligase complex subunit Ufd1	-0.1573322	6.47957043	0.29695503	0.38999401
SPBC609.01	SPBC609.01	cytoplasmic P body 3'-5'-exoribonuclease, Dis3L2-related (p	-0.1573424	6.87548812	0.18631	0.264465872
SPBC146.02	SPBC146.02	Schizosaccharomyces specific protein	-0.1574399	5.79573694	0.36785022	0.463216269
SPAC1D4.02c	grh1	human GRASP protein family Golgi protein (predicted)	-0.1576479	7.40520458	0.22054867	0.305357825
ScpofMt19	#N/A	#N/A	-0.1580414	4.37701449	0.57520725	0.663030585
SPBC18H10.02	lcf1	long-chain-fatty-acid-CoA ligase Lcf1	-0.1584041	9.44468839	0.03950574	0.07080236
SPAC222.18	SPAC222.18	Srp1 family splicing factor (predicted)	-0.1586613	5.69784128	0.3698299	0.465278698
SPAC890.05	pxr1	ribosome biogenesis protein, G-patch domain, PINX1 family	-0.1586642	6.27308523	0.26393942	0.355064163
SPBC530.14c	dsk1	SR protein-specific kinase Dsk1	-0.1589324	7.54005962	0.10445987	0.162405615
SPCC5E4.04	cut1	separase/separin	-0.1590074	8.079834	0.07762128	0.125974909
SPAC17A5.07c	ulp2	SUMO deconjugating cysteine peptidase Ulp2 (predicted)	-0.159078	6.95627982	0.16821453	0.243449563
SPAC1142.03c	swi2	Swi5 complex subunit Swi2	-0.159274	7.11067425	0.20076188	0.281455416
SPCC613.02	SPCC613.02	transmembrane transporter (predicted)	-0.1600106	7.04024686	0.19195535	0.271401649
SPAC2F7.02c	psr1	CTD small phosphatase Psr1 (predicted)	-0.1601734	6.6105155	0.18335811	0.26147358
SPNCRNA.641	#N/A	#N/A	-0.1601866	4.15227727	0.62307189	0.70513957
SPAC20G4.04c	hus1	checkpoint clamp complex protein Hus1	-0.1602474	5.78747308	0.4082742	0.50348167
SPNCRNA.1417	#N/A	#N/A	-0.1602481	6.29962579	0.33328277	0.427599787
SPBC337.16	cho1	phosphatidyl-N-dimethylethanolamine N-methyltransferase	-0.160416	6.26315172	0.29106548	0.383707985
SPBC1198.07c	SPBC1198.07c	mannan endo-1,6-alpha-mannosidase (predicted)	-0.1604415	7.49234955	0.08714654	0.139569829
SPAC7D4.11c	sec39	secretory pathway protein Sec39 (predicted)	-0.1606334	6.27314042	0.25925932	0.349782135
SPBC543.06c	dbp8	ATP-dependent RNA helicase Dbp8 (predicted)	-0.1608655	6.67075302	0.20523477	0.286839717

SPBC543.03c	pku80	Ku domain protein Pku80	-0.161414	7.02751625	0.19093469	0.270126934
SPCC584.02	cuf2	middle-meiotic transcription factor Cuf2	-0.1614156	4.02435164	0.64999765	0.728584088
SPNCRNA.964	#N/A	#N/A	-0.1615376	6.39212562	0.25823643	0.348739608
SPAC959.09c	apc5	anaphase-promoting complex, platform subcomplex scaffol	-0.1616909	6.01895582	0.37297659	0.468198806
SPAC144.09c	sfc2	transcription factor TFIIIA	-0.1621431	6.71726545	0.18631145	0.264465872
SPAC17A2.13c	rad25	14-3-3 protein Rad25	-0.1622637	8.42643176	0.05105653	0.088227453
SPNCRNA.1251	#N/A	#N/A	-0.1622853	2.0988334	0.86363876	0.906049152
SPAC25B8.20	SPAC25B8.20	Schizosaccharomyces specific protein	-0.1626268	5.61622562	0.4058632	0.501070221
SPAC521.04c	sst1	ER membrane calcium trnamembrane transporter (predicte	-0.1627198	7.58562263	0.10094453	0.158241771
SPCC188.12	spn6	meiotic (sporulation) septin Spn6	-0.162847	6.25082138	0.30657018	0.400180972
SPNCRNA.792	#N/A	#N/A	-0.1629721	3.42071432	0.64104819	0.720481369
SPBC16A3.03c	ppr7	mitochondrial PPR repeat protein Ppr7	-0.1634044	6.96286101	0.16750494	0.242577383
SPAC17G8.14c	pck1	protein kinase C (PKC)-like Pck1	-0.1637774	7.89920023	0.06431775	0.107380337
SPBC13G1.03c	pex14	peroxisomal docking protein Pex14 (predicted)	-0.1638195	5.96006342	0.28933502	0.381983027
SPAC17G8.01c	trl1	tRNA ligase Trl1 (predicted)	-0.1639535	7.23800353	0.12530861	0.190170624
SPAC167.03c	snu66	U4/U6 x U5 tri-snRNP complex subunit Snu66	-0.1647583	7.93649406	0.0591306	0.099871868
SPAC6G10.05c	trs120	TRAPP complex subunit Trs120	-0.1647783	8.40261351	0.05791852	0.098239167
SPNCRNA.1477	#N/A	#N/A	-0.1654177	3.1351163	0.75582191	0.818786624
SPBC11C11.10	pus4	tRNA pseudouridine synthase Pus4 (predicted)	-0.1656215	6.93456649	0.19086983	0.270091329
SPCC1223.12c	meu10	GPI anchored cell surface protein involved in ascospore wal	-0.1658658	6.14667747	0.25518992	0.345388712
SPBC14C8.10	mrpl24	mitochondrial ribosomal protein subunit L28 (predicted)	-0.1659989	7.3599846	0.10004741	0.157091239
SPAC23C11.12	hcn1	anaphase-promoting complex subunit TPR lobe accessory fa	-0.1661052	4.11794429	0.59123846	0.676260978
SPAC17G6.09	sec62	ER protein translocation subcomplex subunit Sec62 (predict	-0.1661443	7.48603762	0.12489999	0.189641218
SPAC32A11.01	mug8	DUF1708 family conserved fungal protein, cell division site	-0.1662888	7.3978198	0.13425391	0.201632721
SPBC26H8.14c	cox17	mitochondrial copper chaperone for cytochrome c oxidase C	-0.1666414	5.61410909	0.33848808	0.432972061
SPAC977.02	ftm2	sub-telomeric 5Tm protein family Ftm2	-0.1667681	7.56793868	0.0866206	0.138792837
SPAC31A2.07c	dbp10	ATP-dependent RNA helicase Dbp10 (predicted)	-0.1673841	7.33746125	0.1422558	0.211503322
SPBC947.10	dsc1	Golgi Dsc E3 ligase complex subunit Dsc1	-0.1674782	7.3853091	0.10569696	0.164066559
SPAC4G9.02	rnh201	ribonuclease H2 complex subunit Rnh201	-0.1675302	5.8693036	0.33882961	0.433246046
SPAC23H4.13c	SPAC23H4.13c	Schizosaccharomyces specific protein	-0.1677196	6.44508644	0.40224534	0.497729105
SPBC21D10.10	bdc1	bromodomain protein Bdc1	-0.1678808	6.45441387	0.30024358	0.39322179
SPAC19A8.12	dcp2	mRNA decapping complex catalytic subunit Dcp2	-0.1680012	7.43565205	0.13737582	0.205524499
SPAC19G12.02c	pms1	MutL family mismatch-repair protein Pms1	-0.1680154	8.23919063	0.04271857	0.075782028
SPBC25H2.03	vac14	PAS complex subunit, involved in phosphoinositide metabol	-0.1687553	6.73252469	0.16864202	0.243964488
SPCC663.01c	ekc1	protein phosphatase regulatory subunit Ekc1	-0.1687924	9.786591	0.01025524	0.021565793
SPAC343.15	tit1	tRNA isopentenyltransferase Tit1	-0.1688242	6.34292209	0.23723123	0.325147733
SPAC1093.07	#N/A	#N/A	-0.1689359	6.56626938	0.33377384	0.427987749
SPNCRNA.503	#N/A	#N/A	-0.1690527	3.50463793	0.68147265	0.756759367
SPAC3F10.12c	SPAC3F10.12c	transcription factor (predicted)	-0.1692046	5.78739322	0.38224647	0.477632617
SPCC4F11.03c	SPCC4F11.03c	Schizosaccharomyces specific protein	-0.1693772	7.96950001	0.04416952	0.077747957
SPAC144.17c	SPAC144.17c	6-phosphofructo-2-kinase (predicted)	-0.1696831	7.75736792	0.14618087	0.216258062
SPNCRNA.1395	#N/A	#N/A	-0.1702265	3.85270814	0.74870332	0.813236409
SPBP8B7.07c	set6	histone lysine methyltransferase Set6 (predicted)	-0.170296	6.47664767	0.1798178	0.257233575
SPAC22A12.08c	crd1	cardiolipin synthase/ hydrolase fusion protein Crd1 (predicte	-0.1704105	7.99701383	0.11164674	0.172085178
SPCC1235.09	hif2	Set3 complex subunit Hif2	-0.1705208	7.26295118	0.14400388	0.213682161
SPCC550.01c	coa4	cytochrome c oxidase assembly protein Coa4 (predicted)	-0.1708735	4.87476651	0.52381003	0.615316281
SPAC1952.04c	SPAC1952.04c	conserved fungal plasma membrane protein	-0.1712366	6.23940316	0.2786021	0.370724927

SPAPB1A10.04c	cwp1	geranylgeranyltransferase I and farnesyltransferase alpha s	-0.1714237	6.86435059	0.19895944	0.279677541
SPBC29A3.17	gef3	Cdc42/Rho3/Rho4 RhoGEF Gef3	-0.172042	5.85854288	0.39045493	0.485837597
SPAC167.02	ptb1	geranylgeranyltransferase II beta subunit Ptb1	-0.1721107	8.1913231	0.04115679	0.073278903
SPNCRNA.918	#N/A	#N/A	-0.1721536	5.37477713	0.53237326	0.623889503
SPBC15C4.01c	oca3	TPR repeat protein Oca3/ ER membrane protein complex E	-0.1724801	6.7057331	0.1788044	0.256053021
SPCC830.05c	epl1	NuA4 histone acetyltransferase complex Epl1	-0.1726762	7.06737378	0.09711275	0.152904275
SPNCRNA.1007	#N/A	#N/A	-0.1726784	5.20050719	0.36852178	0.463718697
SPCC16C4.12	naa20	NatB N-acetyltransferase complex catalytic subunit Naa20	-0.1727881	6.24026818	0.26445958	0.355450812
SPAC32A11.03c	phx1	stationary phase-specific homeobox transcription factor Ph	-0.172989	8.24174482	0.03067582	0.056817887
SPNCRNA.1043	#N/A	#N/A	-0.1731202	6.32370206	0.22116625	0.305839532
SPBC29A3.08	pof4	elongin-A, F-box protein Pof4 (predicted)	-0.173401	4.22141144	0.54934041	0.639346929
SPAC1B9.02c	sck1	serine/threonine protein kinase Sck1	-0.1736224	8.31532394	0.05337145	0.091576586
SPAP8A3.10	ups1	mitochondrial phosphatidic acid transfer protein Ups1 (pred	-0.1737276	5.47158407	0.37501533	0.470497666
SPAC212.01c	SPAC212.01c	S. pombe specific DUF999 family protein 2	-0.1739932	5.98452979	0.3782307	0.474007344
SPBC211.03c	gea1	guanyl-nucleotide exchange factor (predicted)	-0.1743089	8.48651567	0.03814037	0.068644604
SPBC1921.04c	#N/A	#N/A	-0.1743938	5.76213704	0.28838271	0.380996371
SPAPB17E12.10c	SPAPB17E12.10c	mitochondrial 2' O-ribose methyltransferase Mrm2-like (pr	-0.1744074	4.70907558	0.49756842	0.591226315
SPBC1778.04	spo6	Spo4-Spo6 kinase complex regulatory subunit Spo6	-0.1745378	6.5758446	0.24425003	0.333491457
SPBC14F5.12c	cbh2	CENP-B homolog Cbh2	-0.1745707	6.9882794	0.18396383	0.262172542
SPBC1734.08	hse1	STAM like protein Hse1	-0.1747246	6.47237792	0.1649974	0.239455601
SPBC11B10.09	cdc2	cyclin-dependent protein kinase Cdk1/Cdc2	-0.1747401	7.07318444	0.10788952	0.167081954
SPAC16E8.09	scd1	RhoGEF Scd1	-0.1749467	7.58178745	0.073523	0.120385947
SPCC736.09c	tfx1	TRAX	-0.1751825	5.42347876	0.3878681	0.48323735
SPAC22F3.12c	rgs1	regulator of G-protein signaling Rgs1	-0.1752371	7.30456049	0.13332832	0.200552429
SPBC409.11	meu18	Schizosaccharomyces specific protein Meu18	-0.1755443	6.23926953	0.32907307	0.423716598
SPBC28E12.02	SPBC28E12.02	RNA-binding protein	-0.1755678	6.98445287	0.12622303	0.191265755
SPAC8C9.16c	mug63	TLDc domain protein 1, implicated in response to oxidative	-0.1758052	5.45138279	0.34757305	0.442596295
SPAC3H5.11	SPAC3H5.11	NAD/NADH kinase (predicted)	-0.1758129	6.96077063	0.15445615	0.22652459
SPAC1071.01c	pta1	mRNA cleavage and polyadenylation specificity factor comp	-0.1760199	7.87799002	0.04831309	0.08404155
SPNCRNA.1651	#N/A	#N/A	-0.1764364	5.7147031	0.30875076	0.402191595
SPCC11E10.09c	SPCC11E10.09c	alpha-amylase homolog (predicted)	-0.1765097	7.50974501	0.10071768	0.157995342
SPAC30D11.10	rad52	DNA recombination protein, Rad51 mediator Rad52 (previo	-0.1765616	7.43787809	0.06767023	0.112042802
SPAC977.17	SPAC977.17	MIP water channel (predicted)	-0.1767578	7.49032572	0.1590474	0.232124149
SPBC1289.01c	chr4	1,3-beta-glucan synthase regulatory factor Chf3/Chr4	-0.1769758	7.45090199	0.06460577	0.107790937
SPBC2G5.04c	erv41	COPII-coated vesicle component Erv41 (predicted)	-0.177366	6.52909566	0.17873982	0.25601441
SPNCRNA.1529	#N/A	#N/A	-0.177379	7.36609423	0.10169452	0.159229072
SPRRNA.49	SPRRNA.49	28S ribosomal RNA	-0.1775939	12.0904106	0.03499164	0.063707261
SPCC188.14	SPCC188.14	Schizosaccharomyces pombe specific protein	-0.1778333	6.40248324	0.22050018	0.305352807
SPAC589.04	mtx1	metaxin 1 (predicted)	-0.1778722	5.75829835	0.29176794	0.38441061
SPAPB17E12.04c	csn2	COP9/signalosome complex subunit Csn2	-0.1785463	6.6342928	0.17000754	0.245210108
SPAC56E4.07	SPAC56E4.07	N-acetyltransferase (predicted)	-0.1787106	8.20853794	0.04747327	0.082728714
SPBC14C8.15	SPBC14C8.15	triglyceride lipase-cholesterol esterase (predicted)	-0.178723	7.96664076	0.06454139	0.107727282
SPAC23H3.10	ssr2	SWI/SNF and RSC complex subunit Ssr2	-0.1787651	6.60893963	0.15425838	0.226283317
SPAC1296.01c	SPAC1296.01c	phosphoacetylglucosamine mutase (predicted)	-0.1789666	8.12987798	0.04122042	0.073353813
SPBC30B4.07c	tfb4	transcription factor TFIIH complex subunit Tfb4	-0.1791597	5.67739442	0.38646487	0.482195354
SPNCRNA.1669	#N/A	#N/A	-0.1792382	4.79677934	0.51019057	0.602228416
SPBC4.05	mlo2	ubiquitin protein ligase E3 component human N-recognin 7	-0.179277	6.49043838	0.21142985	0.294711214

SPRRNA.47	SPRRNA.47	28S ribosomal RNA	-0.1793798	12.0923359	0.03218819	0.059152207
SPRRNA.48	SPRRNA.48	28S ribosomal RNA	-0.1798403	12.0928046	0.03164366	0.058372223
SPNCRNA.979	#N/A	#N/A	-0.1798545	6.6527305	0.1606343	0.234122173
SPCC297.06c	SPCC297.06c	conserved fungal protein	-0.1799841	5.36660455	0.39350171	0.488888884
SPAC1002.15c	med6	mediator complex subunit Med6	-0.18111	6.90568658	0.11108626	0.171493192
SPNCRNA.1512	#N/A	#N/A	-0.1812273	6.18121142	0.1922647	0.271782568
SPBC32H8.13c	mok12	prospore membrane alpha-1,3-glucan synthase Mok12	-0.1814724	8.19927392	0.05166921	0.089127753
SPNCRNA.1656	#N/A	#N/A	-0.181677	6.86984448	0.12213358	0.186057528
SPAC11D3.10	SPAC11D3.10	nifs homolog, possible cysteine desulfurase	-0.1817853	7.13402999	0.08285999	0.133616647
SPNCRNA.1485	#N/A	#N/A	-0.1820517	3.43365174	0.67461023	0.749995521
SPNCRNA.1271	#N/A	#N/A	-0.1823082	6.19140246	0.22111492	0.305830693
SPCC1020.06c	tal1	transaldolase (predicted)	-0.1824518	9.06824187	0.02180148	0.042243475
SPBC31E1.06	bms1	GTP binding protein Bms1 (predicted)	-0.1829083	8.44904305	0.01936349	0.038149555
SPAC3F10.17	ltv1	ribosome biogenesis protein Ltv1 (predicted)	-0.1832379	6.68298318	0.16748059	0.242577383
SPBC1711.17	prp16	ATP-dependent RNA helicase Prp16	-0.183247	7.29152826	0.10277111	0.16040308
SPNCRNA.1265	#N/A	#N/A	-0.1835393	5.23992772	0.390262	0.485775178
SPCC553.09c	spb70	DNA polymerase alpha B-subunit	-0.1836268	6.79729742	0.18866065	0.267410059
SPAC4A8.12c	sds22	protein phosphatase regulatory subunit Sds22	-0.1842654	6.26327918	0.24756547	0.337206365
SPAC4F10.09c	noc1	ribosome biogenesis protein Noc1 (predicted)	-0.1843001	7.67612846	0.07736491	0.125648744
SPNCRNA.661	#N/A	#N/A	-0.1843287	5.63508395	0.36397878	0.459433477
SPBC336.12c	cdc10	MBF transcription factor complex subunit Cdc10	-0.1843912	7.49509797	0.0776067	0.125974909
SPBC8D2.09c	msl1	U2 snRNP-associated protein Msl1 (predicted)	-0.1848755	4.76633748	0.42319981	0.518242697
SPAC23C4.06c	efm6	elongation factor EF-1 alpha (eEF1A) lysine 390 methylase	-0.1850246	6.35372571	0.27144479	0.363046735
SPAC15A10.17	coa2	cytochrome c oxidase assembly protein Coa2 (predicted)	-0.1850949	4.46254676	0.52540589	0.616668232
SPAC222.13c	SPAC222.13c	6-phosphofructo-2-kinase (predicted)	-0.1857145	6.72156616	0.12125407	0.185049098
SPAC23C11.16	plo1	Polo kinase Plo1	-0.1858043	7.30091152	0.07009769	0.115499938
SPNCRNA.1301	#N/A	#N/A	-0.18587	4.84774847	0.50620008	0.599438613
SPRRNA.43	SPRRNA.43	18S ribosomal RNA	-0.1859639	10.958911	0.02688751	0.050670029
SPRRNA.44	SPRRNA.44	18S ribosomal RNA	-0.1859902	10.9596904	0.02681588	0.050577062
SPCC16C4.09	sts5	cytoplasmic P body 3'-5'-exoribonuclease, Dis3L2-related (p	-0.186102	9.50814911	0.01492105	0.030210569
SPAC12B10.12c	rhp41	DNA repair protein Rhp41	-0.1864676	6.65423516	0.14890074	0.219845857
SPNCRNA.1032	#N/A	#N/A	-0.1865796	5.29790217	0.32784411	0.422613976
SPBC18E5.11c	edc3	enhancer of mRNA decapping Edc3	-0.1869994	8.28995919	0.02880082	0.053784196
SPBC1105.10	rav1	RAVE complex subunit Rav1	-0.1870187	7.88374591	0.0289896	0.054062538
SPBC13A2.03	SPBC13A2.03	ER phosphatidate cytidylyltransferase	-0.1870342	6.82069106	0.2306128	0.317184519
SPAC8C9.17c	spc34	DASH complex subunit Spc34	-0.1870348	6.07953983	0.28588284	0.378931195
SPBC2F12.09c	atf21	transcription factor, Atf-CREB family Atf21	-0.187088	5.94531356	0.22604969	0.311705807
SPAC23C4.04c	SPAC23C4.04c	Schizosaccharomyces pombe specific protein	-0.1872186	4.80171893	0.43807229	0.533687408
SPAC23C4.11	atp18	F1-FO ATP synthase subunit J (predicted)	-0.1874083	5.75779157	0.27889795	0.37104605
SPNCRNA.1378	#N/A	#N/A	-0.1875771	7.04370275	0.08918821	0.142559272
SPAC664.12c	SPAC664.12c	mitochondrial succinate dehydrogenase assembly factor 1 (	-0.1876588	6.18222294	0.26131838	0.351923919
SPNCRNA.1554	#N/A	#N/A	-0.1877757	5.02127665	0.38411335	0.479789166
SPAC23A1.02c	ted1	GPI-remodelling mannose-ethanolamine phosphate phosph	-0.1878442	6.45539202	0.16452324	0.238920322
SPNCRNA.1147	#N/A	#N/A	-0.1878978	4.53098384	0.51967418	0.610985279
SPBC3E7.11c	SPBC3E7.11c	DNAJ domain protein Caj1/Djp1-type (predicted)	-0.1879098	6.63345332	0.14393743	0.213636183
SPNCRNA.744	#N/A	#N/A	-0.1883201	5.50611101	0.28699514	0.37988755
SPCC1739.06c	met1	uroporphyrin methyltransferase Met1	-0.1892019	6.67924299	0.18405558	0.262248365

SPRRNA.46	SPRRNA.46	18S ribosomal RNA	-0.1898684	10.9598782	0.02475748	0.04715216
SPAC1071.04c	spc2	signal peptidase subunit Spc2 (predicted)	-0.1899484	4.44978034	0.52589849	0.617033528
SPAC3G9.05	spa2	ARF GTPase-activating protein Spa2	-0.1899812	6.2916719	0.27420642	0.365949142
SPCC4G3.13c	cue1	Hrd1 ubiquitin ligase complex, CUE domain protein Cue1 (pı	-0.1901958	6.60188463	0.16262214	0.23652273
SPBC216.07c	tor2	serine/threonine protein kinase Tor2	-0.1904045	9.16804019	0.01587561	0.031877695
SPNCRNA.1102	#N/A	#N/A	-0.1907007	4.27805254	0.51442409	0.606069245
SPBC577.11	SPBC577.11	DUF3074 family protein, implicated in vesicle trafficking or	-0.190919	7.02659054	0.08962517	0.143101661
SPBC18H10.07	wbp4	WW domain-binding protein Wbp4 (predicted)	-0.1909503	7.30526968	0.0648611	0.108154813
SPBC1685.16	vma9	V-type ATPase V0 subunit e (predicted)	-0.1910161	5.97350188	0.37336285	0.468597234
SPBC31E1.05	gle1	RNA export factor, cytoplasmic nucleoporin Gle1	-0.1911698	5.98255054	0.37151665	0.466796682
SPCC1259.14c	meu27	UPF0300 family protein 5	-0.1911729	6.45550624	0.14075063	0.209723675
SPAC57A7.15c	SPAC57A7.15c	Schizosaccharomyces specific protein	-0.1914717	3.9564182	0.60362209	0.686897713
SPAC22G7.10	iss1	mRNA cleavage and polyadenylation specificity factor comp	-0.1916062	8.06524805	0.03610604	0.06545061
SPBC56F2.01	pof12	F-box protein Pof12	-0.1920088	6.44393135	0.25101676	0.340749801
SPBC27B12.09c	flx1	mitochondrial carrier, FAD Flx1 (predicted)	-0.1921443	6.30452387	0.18416733	0.262352667
SPRRNA.42	SPRRNA.42	18S ribosomal RNA	-0.1922088	10.4330709	0.02325216	0.044710629
SPAC227.10	gim4	prefoldin subunit 2, Gim4(predicted)	-0.192825	7.37881348	0.06732943	0.1115517
SPAPB17E12.07c	sen2	tRNA-splicing endonuclease subunit catalytic subunit Sen2	-0.1930609	5.78997691	0.23103335	0.31761251
SPCC576.05	sac3	TREX2 complex sununit Sac3 (predicted)	-0.193293	7.50034094	0.05590061	0.095290187
SPNCRNA.711	#N/A	#N/A	-0.1934757	7.13818526	0.05482269	0.09376408
SPAC23H4.01c	osh3	sterol intermembrane transfer protein Osh3	-0.1937004	8.38115908	0.02132297	0.041422444
SPBC651.12c	dbl7	Schizosaccharomyces specific protein, double strand break	-0.1937846	4.8482776	0.37982273	0.475477134
SPBC18E5.10	SPBC18E5.10	mitochondrial iron-sulfur cluster protein (predicted)	-0.1940117	8.28358331	0.02383713	0.04565484
SPAC1F3.10c	35703	mitochondrial intermediate peptidase Oct1 (predicted)	-0.1941342	8.36298064	0.04712531	0.082290927
SPRRNA.45	SPRRNA.45	18S ribosomal RNA	-0.1942645	10.0917137	0.02146361	0.041659971
SPNCRNA.809	#N/A	#N/A	-0.1943671	3.51870587	0.6043712	0.687635181
SPNCRNA.942	#N/A	#N/A	-0.1945427	5.87109934	0.21544578	0.299511448
SPAC1565.05	utp8	t-UTP complex subunit Utp8 (predicted)	-0.1945891	7.76028561	0.04017859	0.071781394
SPBC32H8.07	git5	heterotrimeric G protein beta (WD repeat) subunit Git5	-0.1945983	6.15899659	0.24744792	0.337127889
SPAC3H1.11	hsr1	transcription factor Hsr1	-0.1947808	8.50978065	0.02398861	0.045880407
SPNCRNA.1343	#N/A	#N/A	-0.1954251	4.65280705	0.46233142	0.557251355
SPBC19C7.08c	ppm2	tRNA methyltransferase Ppm2 (predicted)	-0.1956577	6.70082263	0.21090662	0.29404211
SPCC330.02	rhp7	Rad7 homolog Rhp7	-0.1957599	7.12360075	0.07707523	0.125267955
SPCC1840.06	atp5	F1-FO ATP synthase delta subunit (predicted)	-0.1961307	6.84901249	0.08397167	0.135057245
SPBC32F12.04	gtb1	gamma-tubulin Gtb1	-0.1961399	7.32136427	0.06212461	0.104255966
SPNCRNA.1480	#N/A	#N/A	-0.1961518	2.9666094	0.73854364	0.805027951
SPNCRNA.1457	#N/A	#N/A	-0.1963244	6.18715378	0.16436529	0.23884386
SPAC13G6.01c	rad8	ubiquitin-protein ligase E3/ ATP-dependent DNA helicase R	-0.1964333	8.27071287	0.0241363	0.046123981
SPAC644.14c	rad51	RecA family recombinase Rad51/Rhp51	-0.1964356	6.70369968	0.15126142	0.222799558
SPAC1296.03c	sxa2	serine carboxypeptidase Sxa2	-0.1967466	5.66631867	0.35454945	0.450015692
SPBC19F8.01c	spn7	meiotic septin Spn7	-0.1974024	5.6101556	0.25239175	0.342341211
SPBC146.06c	fan1	Fanconi-associated nuclease Fan1	-0.1978774	6.12777943	0.2687909	0.360205219
SPCC1795.01c	mad3	mitotic spindle checkpoint protein Mad3	-0.1981687	6.06906672	0.1799388	0.257352576
SPCC1840.07c	SPCC1840.07c	vacuolar endopolyphosphatase (predicted)	-0.1981707	7.19173144	0.06722912	0.111483286
SPAC823.09c	SPAC823.09c	threonine aspartase, unknown biological role (predicted)	-0.1983777	5.89134037	0.22434638	0.309733635
SPAC13C5.02	dre4	splicing associated factor Dre4	-0.1988245	6.48096281	0.12249183	0.186519788
SPBC337.08c	ubi4	protein modifier, ubiquitin	-0.1989104	9.92513173	0.0025184	0.00616907

SPNCRNA.1440	#N/A	#N/A	-0.1993086	5.23127406	0.350357	0.44564101
SPAC25H1.10c	atp19	F1-FO ATP synthase subunit K (predicted)	-0.1995946	6.34460716	0.14727426	0.217680572
SPBC887.05c	cwf29	RNA-binding protein Cwf29	-0.1998606	6.5400491	0.10414206	0.16209668
SPCC757.07c	ctt1	catalase	-0.1999307	8.57426928	0.08119349	0.131084619
SPCP25A2.02c	rhp26	SNF2 family ATP-dependent DNA helicase Rhp26	-0.2002437	7.24649014	0.11313293	0.173981822
SPNCRNA.894	#N/A	#N/A	-0.2003368	6.05636069	0.21906001	0.303729293
SPNCRNA.816	#N/A	#N/A	-0.2005766	5.86633457	0.22990154	0.316440122
SPBC1604.25	pet117	cytochrome c oxidase assembly protein Pet117 (predicted)	-0.2007604	4.93812295	0.38732041	0.48273175
SPCC830.10	ham1	nucleoside triphosphatase Ham1 (predicted)	-0.2007993	6.61014721	0.14224528	0.211503322
SPAC343.11c	msc1	Swr1 complex subunit Msc1	-0.200844	8.43990871	0.01904408	0.037618266
SPNCRNA.1584	#N/A	#N/A	-0.2012043	4.66643783	0.41920155	0.514179256
SPAPB1A11.04c	mca1	transcription factor, zf-fungal binuclear cluster type Mca1	-0.2012118	7.52618525	0.02870564	0.053621161
SPBC3F6.05	rga1	RhoGAP, GTPase activating protein Rga1	-0.2012238	8.52013862	0.00768526	0.016634278
SPAC23G3.07c	snf30	SWI/SNF complex subunit Snf30	-0.2014028	6.86390262	0.14223965	0.211503322
SPCC965.06	osr2	potassium channel, beta subunit, aldo-keto reductase (pred	-0.2016096	8.00008354	0.05442648	0.093151956
SPBC15D4.05	SPBC15D4.05	conserved CobW/HypB/UreG nucleotide binding domain pro	-0.2016222	6.45886403	0.14953302	0.220540141
SPBC365.08c	der1	Hrd1 ubiquitin ligase complex (derlin) Der1/Dfm1 (predicte	-0.201867	5.78885693	0.35042732	0.445647147
SPBC354.09c	tre1	Tre1 family protein, involved in vacuolar protein degradatio	-0.201995	7.7658142	0.04326039	0.076543672
SPBC31E1.02c	pmr1	plasma membrane P-type ATPase, calcium transporting Pm	-0.2020922	9.24559971	0.00287871	0.006944664
SPBC1683.04	SPBC1683.04	glycosyl hydrolase family 3 (predicted)	-0.2024553	7.36445907	0.05587472	0.095290187
SPAC22F3.05c	alp41	GTP-binding protein involved in beta-tubulin folding Alp41	-0.2024842	7.61898871	0.03450532	0.063018975
SPBC428.06c	rxt2	histone deacetylase complex subunit Rxt2	-0.2028537	5.98213891	0.2989451	0.391893929
SPAC1782.01	ecm29	proteasome assembly chaperone Ecm29	-0.2029141	8.83751755	0.00587051	0.013141059
SPBC2G5.03	ctu1	cytosolic thiouridylase subunit Ctu1	-0.203087	5.64053512	0.24571768	0.335091942
SPAC17H9.20	psc3	mitotic cohesin complex, HEAT repeat subunit Psc3	-0.203128	7.23847365	0.04437449	0.077999406
SPNCRNA.1275	#N/A	#N/A	-0.2036006	5.72549677	0.28219228	0.374915752
SPBP35G2.02	txc1	26S proteasome co-factor Txc1	-0.2037037	7.11823591	0.06500313	0.108365098
SPNCRNA.1510	#N/A	#N/A	-0.2038384	3.92693426	0.48989677	0.584662844
SPNCRNA.1078	#N/A	#N/A	-0.2038985	6.33147238	0.13263462	0.199685528
SPAC637.11	rpm2	mitochondrial ATP-dependent RNA helicase Rpm2	-0.2039904	6.36310004	0.1279667	0.193600128
SPAC144.10c	gwt1	pig-W	-0.2042948	7.79726236	0.03195303	0.058799456
SPAC1782.02c	SPAC1782.02c	conserved fungal protein	-0.2043827	6.01571117	0.25987648	0.350398142
SPBC557.05	SPBC557.05	arrestin, implicated in vesicle-mediated transport	-0.2048756	7.3447849	0.09265169	0.147320442
SPAC1002.01	mrx11	mitochondrial expression network (MIOREX) component Mı	-0.2055152	3.98795761	0.54682743	0.637127676
SPCC1442.19	mrp49	mitochondrial ribosomal protein Mrp49 (predicted)	-0.2059017	5.03566306	0.34182958	0.436425951
SPAC23G3.09	taf4	transcription factor TFIID complex subunit Taf4 (predicted)	-0.2059904	7.48219432	0.05421678	0.092839749
SPBC17D1.02	dph2	diphthamide biosynthesis protein Dph2 (predicted)	-0.2061097	6.46974757	0.13575997	0.20344562
SPAC19G12.03	cda1	chitin deacetylase Cda1	-0.2061821	7.7133411	0.02741557	0.051550964
SPAC24C9.04	SPAC24C9.04	Schizosaccharomyces specific protein	-0.2062842	6.51865389	0.13100936	0.19750082
SPAPB1E7.14	iec5	Ino80 complex subunit Iec5	-0.2066426	6.77055066	0.15423543	0.226283317
SPBC337.04	ppk27	calcium/calmodulin-dependent protein kinase Ppk27 (predi	-0.2069099	5.33959277	0.2645261	0.355470002
SPNCRNA.1194	#N/A	#N/A	-0.2071304	8.25979049	0.03538816	0.064303452
SPCC622.08c	hta1	histone H2A alpha	-0.2071513	8.24651558	0.03727142	0.067205098
SPNCRNA.11	#N/A	#N/A	-0.2075766	2.53201523	0.76062722	0.821989557
SPCC736.04c	gma12	alpha-1,2-galactosyltransferase Gma12	-0.2076437	7.77191569	0.02271865	0.04377135
SPNCRNA.1005	#N/A	#N/A	-0.2079891	7.27525679	0.04239723	0.07527085
SPAC13G6.09	trs402	SSU-rRNA maturation protein Tsr4 homolog 2 Tsr402 (pred	-0.2085141	6.24759784	0.13909208	0.207525016

SPAC17A5.14	exo2	exonuclease II Exo2	-0.2090128	8.49552817	0.02030611	0.039684974
SPCC970.02	SPCC970.02	mannan endo-1,6-alpha-mannosidase (predicted)	-0.2090742	6.44656792	0.23260806	0.319454668
SPAC4D7.10c	spt20	SAGA complex subunit Spt20	-0.2091151	6.56789403	0.09486392	0.150092757
SPCC1494.01	SPCC1494.01	iron/ascorbate oxidoreductase family	-0.2092003	6.49972301	0.09284873	0.147521746
SPAC27F1.10	SPAC27F1.10	Schizosaccharomyces pombe specific protein	-0.2094268	3.86593425	0.55201178	0.641797398
SPAC30C2.03	SPAC30C2.03	Schizosaccharomyces specific protein	-0.2094637	5.14918945	0.3212083	0.415397659
SPCC4B3.06c	SPCC4B3.06c	NADPH-dependent FMN reductase (predicted)	-0.2097521	6.17929827	0.18074452	0.258342045
SPAC17G6.08	pep7	prevacuole/endosomal FYVE tethering component Pep7 (pr	-0.2098765	6.53390588	0.11281759	0.173614587
SPAC1610.03c	crp79	poly(A) binding protein Crp79	-0.2103064	6.07047199	0.26536711	0.356398045
SPBC29B5.01	atf1	transcription factor, Atf-CREB family Atf1	-0.2104906	8.31627118	0.02795242	0.052336102
SPAC343.06c	SPAC343.06c	mitochondrial phospholipid scramblase (predicted)	-0.2108795	6.19129328	0.1940791	0.273949031
SPAC1851.04c	ric1	Ypt/Rab-specific guanyl-nucleotide exchange factor (GEF) s	-0.2110668	7.96817927	0.00965622	0.020419695
SPNCRNA.617	#N/A	#N/A	-0.2110754	8.28556532	0.01622051	0.032503106
SPNCRNA.1610	#N/A	#N/A	-0.2111442	6.62019318	0.16906332	0.24431427
SPNCRNA.1573	#N/A	#N/A	-0.2123097	6.46433088	0.12800703	0.193618108
SPAC4F10.10c	mnn9	mannosyltransferase complex subunit, Anp family Mnn9 (pı	-0.2125964	8.44318598	0.01201562	0.024815264
SPNCRNA.883	#N/A	#N/A	-0.2127601	6.06413454	0.26611472	0.357252053
SPAC144.08	jac1	mitochondrial (2Fe-2S) cluster assembly co-chaperone Jac1	-0.2129565	7.21040684	0.05946082	0.100354885
SPAC3G6.09c	tps2	trehalose-phosphate synthase Tps2 (predicted)	-0.2132362	7.84407205	0.09640762	0.152005062
SPAC11D3.11c	SPAC11D3.11c	zn(2)-C6 fungal-type DNA-binding transcription factor, trunc	-0.2135268	6.78197479	0.08920167	0.142559272
SPBC26H8.12	cyc3	cytochrome c heme lyase Cyc3 (predicted)	-0.2137266	7.68503764	0.0358309	0.065003808
SPAC688.13	scn3	TatD DNase family Scn1	-0.2138463	5.26850635	0.29814864	0.391151238
SPAC3G9.07c	hos2	histone deacetylase (class I) Hos2	-0.2141401	6.19790412	0.12700137	0.192396326
SPNCRNA.471	#N/A	#N/A	-0.2144064	4.11754661	0.5113633	0.603089645
SPBC409.05	skp1	SCF ubiquitin ligase complex subunit Skp1	-0.2145848	6.86332465	0.11135039	0.171706182
SPCC297.03	ssp1	Ca2+/calmodulin-dependent (CaMMK)-like protein kinase S	-0.2147675	8.70125153	0.00557785	0.012518884
SPBC1A4.05	blt1	ubiquitin domain-like protein Blt1	-0.2150458	7.70589949	0.0368294	0.066531469
SPAC10F6.15	SPAC10F6.15	UPF0300 family protein 1	-0.2153055	4.91619457	0.41068311	0.505919368
SPNCRNA.400	#N/A	#N/A	-0.2153363	2.70667757	0.70436327	0.775269219
SPCC1259.08	SPCC1259.08	DUF2457 family conserved fungal protein	-0.2153864	8.26943116	0.00666772	0.014674599
SPBP35G2.16c	ecl2	extender of chronological lifespan protein Ecl2	-0.2155844	8.08540584	0.25082475	0.340623111
SPCC777.14	prp4	serine/threonine protein kinase Prp4	-0.2163056	6.14271035	0.22091372	0.305676669
SPAC4F8.10c	stg1	SM22/transgelin-like actin modulating protein Stg1	-0.2164874	6.82559859	0.08085016	0.13065432
SPBC2D10.07c	mmp1	mitochondrial inner membrane peptidase complex catalytic	-0.2165497	6.17425522	0.15343069	0.225263402
SPBC21B10.09	SPBC21B10.09	endomembrane system acetyl-CoA transmembrane transpo	-0.2166591	7.29707117	0.03199126	0.058837985
SPAC1B3.09c	noc202	Noc complex subunit Noc202 (predicted)	-0.2167637	7.22943461	0.02819054	0.052716849
SPAC24H6.09	gef1	Cdc42 RhoGEF Gef1	-0.216856	7.71953348	0.04138259	0.073623136
SPAC26H5.13c	kei1	inositol phoshorylceramide synthase regulatory subunit Kei1	-0.2169015	7.55214599	0.02897221	0.05404493
SPAC29B12.01	ino80	SNF2 family ATP-dependent 3' to 5' DNA helicase Ino80	-0.2169019	8.82082434	0.00313349	0.007495033
SPBC21D10.09c	rkr1	RQC complex ubiquitin-protein ligase E3 Rkr1 (predicted)	-0.2170596	8.35309902	0.07694506	0.125131432
SPNCRNA.1365	#N/A	#N/A	-0.2171428	3.13260491	0.71022512	0.780020045
SPBC1683.13c	cha4	transcription factor Cha4 (predicted)	-0.2173144	6.38903811	0.16541698	0.239945818
SPBPB2B2.17c	ftm7	sub-telomeric 5Tm protein family Ftm7	-0.2180673	6.07452495	0.20835786	0.29072693
SPAC1687.20c	mis6	CENP-I ortholog Mis6	-0.2182725	5.07187726	0.30243534	0.395554972
SPBC354.05c	sre2	membrane-tethered transcription factor Sre2	-0.2187502	8.2123507	0.02766917	0.051927394
SPCC1682.07	ssl1	transcription factor TFIIH complex subunit Ssl1	-0.2190382	6.81705753	0.07894007	0.127840824
SPBC336.09c	rrn7	RNA polymerase I general transcription initiation factor sub	-0.2193336	6.68324233	0.15323438	0.225072294

SPAC12G12.01c	sea4	SEA complex ubiquitin-protein ligase E3 subunit Sea4 (pred	-0.2198622	7.72780219	0.01784514	0.035486893
SPAC3H1.13	ppk13	serine/threonine protein kinase Ppk13 (predicted)	-0.2198758	6.21972231	0.16091809	0.23448557
SPBC428.01c	nup107	nucleoporin Nup107	-0.2198957	8.13599349	0.00638763	0.014137662
SPBC4F6.08c	mrpl39	mitochondrial ribosomal protein subunit L39 (predicted)	-0.2200207	4.06152955	0.5923633	0.676763897
SPAC29E6.04	nnf1	NMS complex subunit Nnf1	-0.2204253	5.345883	0.2639632	0.355064163
SPBP8B7.29	abz1	para-aminobenzoate synthase (predicted)	-0.2204766	6.9354931	0.07692998	0.125131432
SPBP8B7.30c	thi5	transcription factor Thi5	-0.2213336	6.01383039	0.18768327	0.266191045
SPCC1919.13c	bmt5	ribosome biogenesis protein Bmt5 (predicted)	-0.2215056	5.73448512	0.24827581	0.33783581
SPAC57A7.06	utp14	U3 snoRNP protein Utp14 (predicted)	-0.221519	7.82585224	0.01000292	0.021106929
SPCC132.02	hst2	Sirtuin family histone deacetylase Hst2	-0.2215494	6.61969866	0.09546852	0.150909014
SPBC776.06c	SPBC776.06c	Arf3/6 docking factor (predicted)	-0.2218394	6.34932479	0.15900956	0.232124149
SPBC83.05	SPBC83.05	mitochondrial RNA-binding protein (predicted)	-0.2220613	8.30268295	0.04750259	0.082758605
SPBP4H10.21c	sld5	GINS complex subunit Sld5	-0.2221486	6.28798008	0.11120129	0.171592926
SPAC27D7.14c	tpr1	RNA polymerase II associated Paf1 complex subunit Tpr1	-0.2228678	8.46253474	0.035103	0.063870572
SPCC417.02	dad5	DASH complex subunit Dad5	-0.222918	6.36160704	0.09745273	0.153404078
SPAC17H9.05	ebp2	rRNA processing protein Ebp2	-0.2231061	8.2514451	0.01958087	0.038477568
SPNCRNA.946	#N/A	#N/A	-0.2233201	6.36700939	0.09448992	0.14964019
SPAC26H5.12	rpo41	mitochondrial RNA polymerase Rpo41	-0.2233466	8.2373525	0.00699209	0.015309094
SPAC977.04	SPAC977.04	truncated C terminal region of membrane transporter	-0.2234302	5.5330848	0.24210961	0.331033929
SPAC977.18	SPAC977.18	conserved fungal plasma membrane protein	-0.2234316	8.52043412	0.01130008	0.023537515
SPAC20G8.01	cdc17	ATP-dependent DNA replication ligase Cdc17	-0.2236053	7.18465587	0.06462799	0.107792525
SPAC144.13c	srw1	substrate-specific mitotic G1 APC coactivator Srw1/Ste9	-0.2237345	7.01836905	0.06624491	0.110057759
SPCC1795.10c	svp26	Sed5 Vesicle Protein Svp26 (predicted)	-0.2242026	6.54465568	0.07349907	0.120375731
SPBC2A9.03	SPBC2A9.03	WD40/YVTN repeat-like protein	-0.2244175	5.32916262	0.29011804	0.382681389
SPCC18B5.06	dom34	Dom34-Hbs1 translation release factor complex subunit, pe	-0.2244576	6.27194933	0.11064055	0.170921446
SPBC3H7.01	spo14	GDP/GTP exchange factor, WD repeat protein Spo14	-0.2244985	7.10113318	0.04398954	0.077591715
SPAC25A8.02	atg14	autophagy associated protein Atg14	-0.2245277	6.40084263	0.11431796	0.175605797
SPAC11D3.17	SPAC11D3.17	transcription factor, zf-fungal binuclear cluster type	-0.2245393	7.38725279	0.10086776	0.15815786
SPNCRNA.1628	#N/A	#N/A	-0.2245854	7.04957793	0.04405948	0.077654692
SPCC16A11.11	mrpl31	mitochondrial ribosomal protein subunit L31 (predicted)	-0.2251925	5.48806413	0.34425005	0.439186646
SPCC13B11.04c	fmd3	glutathione-dependent formaldehyde dehydrogenase (predi	-0.2253377	6.20323323	0.13097913	0.197498995
SPAC22E12.19	snt1	Set3 complex subunit Snt1	-0.2254678	7.34877991	0.02385578	0.045669099
SPAC3H8.03	img2	mitochondrial ribosomal protein subunit Mrpl49/Img2 (pre	-0.225506	5.14344767	0.39725828	0.492681353
SPBC3D6.04c	mad1	mitotic spindle checkpoint protein Mad1	-0.2256983	5.9953139	0.15308213	0.224945772
SPCC757.11c	SPCC757.11c	transmembrane transporter (predicted)	-0.2257303	6.77219528	0.0737827	0.12072403
SPAC23D3.03c	SPAC23D3.03c	GTPase activating protein (predicted)	-0.2257502	7.36045104	0.03715081	0.067023136
SPBC13A2.02	nup82	nucleoporin, WD repeat Nup82	-0.2259786	7.58408336	0.02771063	0.051990858
SPNCRNA.733	#N/A	#N/A	-0.2260361	4.18639114	0.47853455	0.573617688
SPNCRNA.1587	#N/A	#N/A	-0.2260976	5.46860775	0.21393152	0.297588717
SPAC26F1.07	SPAC26F1.07	glucose 1-dehydrogenase (NADP+) (predicted)	-0.2261018	8.72999158	0.0318739	0.058685568
SPAC1F12.06c	SPAC1F12.06c	inosine-containing RNA endoribonuclease (predicted)	-0.2261445	7.14482527	0.17608147	0.252738753
SPNCRNA.1094	#N/A	#N/A	-0.2264424	7.89916056	0.01380107	0.0281608
SPAC3C7.12	tip1	CLIP170 family protein Tip1	-0.2264447	7.30586182	0.02351395	0.045162981
SPAC4H3.04c	SPAC4H3.04c	MEMO1 family, human MEMO1 ortholog, ancient conserve	-0.2267727	4.97127775	0.30030472	0.39322179
SPBC609.02	ptn1	phosphatidylinositol-3,4,5-trisphosphate3-phosphatase Ptn1	-0.2268092	4.66653788	0.36323229	0.458759414
SPNCRNA.1212	#N/A	#N/A	-0.2271608	6.66302597	0.07366949	0.120596788
SPBC1105.07c	pci2	TREX/TREX2 complex subunit Pci2 (predicted)	-0.227206	7.20999789	0.09129555	0.145359438

SPNCRNA.777	#N/A	#N/A	-0.2273395	4.9844927	0.37970862	0.475421743
SPBC26H8.01	thi2	thiazole biosynthetic enzyme	-0.2276449	6.74152607	0.12157609	0.185374252
SPNCRNA.817	#N/A	#N/A	-0.2277666	5.14002968	0.28163413	0.374393479
SPNCRNA.762	#N/A	#N/A	-0.2278755	6.08886299	0.22043549	0.305325364
SPNCRNA.572	#N/A	#N/A	-0.2284234	5.56732147	0.24040787	0.32897156
SPBC12D12.04c	pck2	protein kinase C (PKC)-like Pck2	-0.2291283	8.21815693	0.00455035	0.010454126
SPAC57A10.07	SPAC57A10.07	conserved membrane protein with Rossmann-like alpha/be	-0.2291591	6.52916801	0.07448601	0.121699237
SPAC23H4.16c	not11	CCR4-Not complex subunit Not11	-0.2291911	4.83050292	0.39009877	0.485660839
SPAC1952.13	ned1	lipin, phosphatidate phosphatase Ned1	-0.2291972	7.62095257	0.09040209	0.14413922
SPAC24C9.07c	bgs2	spore wall 1,3-beta-glucan synthase catalytic subunit Bgs2	-0.2294745	7.9424811	0.00852738	0.018236576
SPBC29A10.15	orc1	origin recognition complex subunit Orc1	-0.2299035	7.4776538	0.02759535	0.051817427
SPBC13G1.05	SPBC13G1.05	ER protein folding protein (predicted)	-0.2300746	6.24753583	0.25478464	0.345036708
SPAC144.02	iec1	Ino80 complex subunit Iec1	-0.2301223	6.57958613	0.05499104	0.094023626
SPBC11C11.01	SPBC11C11.01	U2-associated protein (predicted)	-0.2303065	6.17763067	0.13664812	0.204596357
SPCC11E10.04	ppr6	mitochondrial PPR repreat protein Ppr6	-0.2303895	6.5927051	0.08459066	0.135810692
SPAC4H3.11c	ppc89	spindle pole body protein Ppc89	-0.2319304	7.23738976	0.12707748	0.192468783
SPAC22F8.10c	sap145	U2 snRNP-associated protein Sap145	-0.2319315	6.37647709	0.11830919	0.181286651
SPNCRNA.838	#N/A	#N/A	-0.231968	7.13282644	0.09650504	0.152065368
SPBC25H2.10c	tan1	tRNA acetyltransferase Tan1 (predicted)	-0.232142	6.38700509	0.17005976	0.245233458
SPBC8D2.14c	sed5	SNARE Sed5 (predicted)	-0.2323456	7.48755832	0.02055206	0.040073563
SPBC1778.03c	SPBC1778.03c	NADH pyrophosphatase (predicted)	-0.2323869	6.19198021	0.12458547	0.189241991
SPBC11C11.04c	alp1	tubulin specific chaperone cofactor D, Alp1	-0.232419	6.65264268	0.26076809	0.351321893
SPBC8D2.13	shq1	box H/ACA snoRNP assembly protein Shq1 (predicted)	-0.2324668	7.48590222	0.02036942	0.039774424
SPAC29E6.01	pof11	F-box/WD repeat protein Pof11	-0.2324692	7.45063893	0.03398078	0.062211255
SPAC4D7.03	pop2	F-box/WD repeat protein Pop2	-0.2328704	7.01685134	0.03127677	0.05782081
SPBC28E12.04	SPBC28E12.04	Schizosaccharomyces specific protein	-0.2330904	5.55311343	0.19786281	0.278250961
SPBC947.14c	cbp6	mitochondrial Cob1 translation regulator Cbp6 (predicted)	-0.2331594	6.97999464	0.03512646	0.063889154
SPNCRNA.193	#N/A	#N/A	-0.2334722	4.15209784	0.44782021	0.543793096
SPAC3H8.06	aur1	inositol phosphorylceramide synthase Aur1 (predicted)	-0.2341142	7.67749531	0.03849426	0.069170174
SPNCRNA.1183	#N/A	#N/A	-0.2341863	4.71466533	0.40782271	0.503124287
SPNCRNA.1466	#N/A	#N/A	-0.2344385	4.14366003	0.54209182	0.63296754
SPAC821.10c	sod1	superoxide dismutase Sod1	-0.2355097	8.80341278	0.00177398	0.004497734
SPCC757.04	SPCC757.04	transcription factor (predicted)	-0.2355234	7.4893819	0.01982964	0.038943933
SPAC13A11.01c	rga8	RhoGAP, GTPase activating protein Rga8	-0.2355727	8.22796787	0.00626501	0.013900687
SPBC887.11	pus2	tRNA pseudouridine synthase Pus2 (predicted)	-0.2355731	5.83201613	0.14290532	0.212283502
SPNCRNA.969	#N/A	#N/A	-0.2359073	6.52817949	0.10437425	0.162309595
SPAC25B8.02	sds3	Clr6 histone deacetylase complex subunit Sds3	-0.2363132	5.46368519	0.33037476	0.424990594
SPAC3A11.09	sod22	plasma membrane sodium ion/proton antiporter Sod22	-0.2363787	7.23049229	0.03089074	0.057169286
SPBC4C3.12	35673	forkhead transcription factor Sep1	-0.2363795	7.22460585	0.0408319	0.072795677
SPAC458.06	atg1803	autophagy associated WD repeat protein Atg18c	-0.2364759	4.86138426	0.33168179	0.42618855
SPAC17C9.02c	lys7	alpha-aminoadipate reductase phosphopantetheinyl transfe	-0.2374999	3.96669094	0.4870422	0.582174982
SPNCRNA.59	#N/A	#N/A	-0.2376524	5.11786486	0.25605211	0.346339616
SPAC4F8.12c	spp42	U5 snRNP complex subunit Spp42	-0.2377643	10.0045094	0.00083863	0.002296517
SPNCRNA.277	#N/A	#N/A	-0.2381851	3.67153286	0.52541402	0.616668232
SPAC4A8.08c	vrs2	mitochondrial valine-tRNA ligase Vrs2/Vas2	-0.238319	7.45958381	0.05192161	0.089495082
SPBC36B7.09	gcn2	eIF2 alpha kinase Gcn2	-0.2386011	8.7507068	0.00231619	0.005733596
SPBC146.09c	lsd1	histone demethylase SWIRM1	-0.2388639	7.60259166	0.03103614	0.057422757

SPAC1F12.09	gpi17	pig-S (predicted)	-0.2391734	6.84761737	0.03576849	0.064914785
SPAPB1A10.10c	ypt71	GTPase Ypt71	-0.2395272	6.64803886	0.05389405	0.092380104
SPAC3A11.08	pcu4	cullin 4	-0.2397978	7.24395003	0.06418425	0.107258255
SPNCRNA.978	#N/A	#N/A	-0.2403352	3.76735012	0.49161482	0.586404701
SPAC19G12.01c	cut20	anaphase-promoting complex, platform subcomplex scaffol	-0.2403385	6.55379519	0.05073125	0.08779913
SPAPB17E12.03	pex12	ubiquitin-protein ligase E3 Pex12 involved in peroxisome or	-0.2406416	6.77131163	0.05309313	0.091280675
SPCC1840.01c	mog1	Ran GTPase binding protein Mog1	-0.240792	5.4694738	0.16262893	0.23652273
SPCC1235.01	SPCC1235.01	Schizosaccharomyces specific protein	-0.241058	9.80833631	0.00067412	0.001895623
SPAC1952.02	tma23	ribosome biogenesis protein Tma23 (predicted)	-0.2411344	6.11002596	0.10589634	0.164338568
SPBC1709.14	ngl1	peptide N-glycanase Ngl1	-0.2412467	6.88439905	0.03490486	0.063578052
SPBC56F2.11	met6	homoserine O-acetyltransferase Met6	-0.2412834	8.11461589	0.00253097	0.006195409
SPBC16A3.10	ale1	membrane bound O-acyltransferase, MBOAT Ale1 (predicte	-0.2413337	7.27251009	0.01803124	0.035804674
SPCC306.10	wtf8	wtf element Wtf8	-0.2415046	4.40530322	0.42985447	0.525257618
SPBC2D10.14c	myo51	myosin type V	-0.2419101	7.48859033	0.01470824	0.029832953
SPBC1921.06c	pvg3	Golgi galactosylxylosylprotein 3-beta-galactosyltransferase	-0.2420477	6.55773549	0.06904731	0.11395943
SPNCRNA.1459	#N/A	#N/A	-0.2421083	6.10503118	0.14422847	0.213880876
SPAC19A8.05c	sst4	sorting receptor for ubiquitinated membrane proteins, ESCI	-0.2422692	7.645621	0.01852106	0.036713023
SPAC5D6.13	vps74	Golgi phosphoprotein 3 family Vps74 (predicted)	-0.2423234	7.69388403	0.01022463	0.021514718
SPAC1327.01c	SPAC1327.01c	transcription factor, zf-fungal binuclear cluster type (predic	-0.2423823	7.83269301	0.00408417	0.009479128
SPAC3A12.03c	meu34	ubiquitin-protein ligase E3 Meu34, human RNF13 family ho	-0.2424892	5.51966318	0.15824972	0.231390068
SPCC1322.14c	vtc4	vacuolar transporter chaperone (VTC) complex subunit (pre	-0.2426043	8.71567171	0.00162573	0.004152809
SPAC1A6.06c	meu31	Schizosaccharomyces specific protein Meu31	-0.2431247	3.94450081	0.67389624	0.749446632
SPNCRNA.710	#N/A	#N/A	-0.2431247	3.94450081	0.67389624	0.749446632
SPAC13G7.01c	erg7	lanosterol synthase Erg7	-0.2431555	6.81837935	0.05156859	0.089007709
SPBC646.02	cwf11	U2-type spliceosomal complex ATPase Cwf11	-0.2433795	7.5712956	0.01952097	0.038384794
SPBC20F10.07	ltc1	GRAM domain membrane contact site protein Ltc1	-0.2439286	7.83087446	0.02646974	0.049993506
SPAC26A3.10	cnt6	centaurin ADP ribosylation factor GTPase activating protein	-0.2440864	8.20250917	0.00343888	0.008151028
SPCC736.10c	mrps8	mitochondrial ribosomal protein subunit S8 (predicted)	-0.2445111	6.51069708	0.05911064	0.099862932
SPBC685.05	gpi15	pig-H (predicted)	-0.2447158	3.76754245	0.49578471	0.589757617
SPCC757.10	vph2	endoplasmic reticulum membrane protein involved in asser	-0.2450469	5.31976753	0.28246191	0.375200724
SPCC1020.11c	emc6	ER membrane protein complex subunit Emc6 (predicted)	-0.2450899	5.57183486	0.3607415	0.456545641
SPCC16A11.13	luc7	U1 snRNP-associated protein Luc7	-0.2451054	6.00584266	0.11301398	0.173838188
SPAC17H9.08	SPAC17H9.08	mitochondrial carrier, coenzyme A (predicted)	-0.2455294	7.25473033	0.08360983	0.134570687
SPCC736.06	dar2	mitochondrial aspartate-tRNA ligase Dar2 (predicted)	-0.2459553	7.51943095	0.02540513	0.048250611
SPAC2E1P5.05	rrp9	U3 snoRNP-associated protein Rrp9 (predicted)	-0.2459694	7.06146018	0.04645115	0.081238782
SPBC19C7.07c	sen34	tRNA-splicing endonuclease catalytic subunit Sen34 (predic	-0.2463039	6.7079359	0.09967273	0.156572698
SPBC1604.15	gpi16	pig-T, Gpi16 (predicted)	-0.24641	7.68382439	0.01028257	0.021616593
SPBC713.08	tom13	mitochondrial TOM complex assembly protein Tom13 (prec	-0.2470524	6.13979324	0.21770225	0.302092947
SPBC17D11.04c	nto1	histone acetyltransferase complex PHD finger subunit Nto1	-0.2470652	7.11481875	0.04389691	0.077468482
SPBC1703.15c	vps33	HOPS/CORVET complex subunit, vacuolar sorting protein Vp	-0.2470993	7.18030854	0.02019847	0.039520015
SPAC15A10.13	ppk3	protein kinase domain and HEAT repeat protein Ppk3	-0.2477036	7.07333106	0.03368726	0.061723701
SPNCRNA.840	#N/A	#N/A	-0.2477196	6.16831741	0.13117215	0.197702435
SPBC16H5.05c	cyp7	cyclophilin family peptidyl-prolyl cis-trans isomerase Cyp7 (	-0.2480778	6.63695931	0.06978624	0.115042479
SPAC19A8.11c	irc6	clathrin coat adaptor Irc6	-0.2483512	8.13936653	0.00333828	0.007920844
SPAC644.11c	pkp1	mitochondrial pyruvate dehydrogenase (lipoamide) kinase F	-0.2485895	7.27286355	0.01506224	0.030451143
SPNCRNA.1657	#N/A	#N/A	-0.2486188	4.43615769	0.41087349	0.505970696
SPBC36B7.02	svf2	Svf1 family protein, lipocalin superfamily Svf2	-0.248643	7.3217292	0.05399946	0.092514178

SPBC21B10.12	rec6	meiotic recombination protein Rec6	-0.2489363	5.23147338	0.24323281	0.332436083
SPAC458.05	pik3	phosphatidylinositol 3-kinase Pik3	-0.2490334	7.17511936	0.02753221	0.051727403
SPAC27E2.12	SPAC27E2.12	Schizosaccharomyces pombe specific protein	-0.2492203	2.2031052	0.74160804	0.807592045
SPBC29A3.01	ccc2	Golgi copper transporting ATPase Ccc2 (predicted)	-0.2494981	8.26738288	0.0028789	0.006944664
SPBC336.14c	ppk26	PAN complex protein phosphotransferase subunit Ppk26 (pr	-0.2496866	6.78679755	0.03562914	0.064706777
SPAC23A1.16c	rtr1	RNA polymerase II CTD phosphatase Rtr1 (predicted)	-0.2497637	5.12702795	0.23205309	0.318756818
SPAC2C4.05	cor1	cornichon family protein (predicted)	-0.2498778	6.52501109	0.13200236	0.198865631
SPAC23H4.04	slm3	mitochondrial tRNA-specific 2-thiouridylase Slm3 (predicte	-0.2499408	6.18271059	0.13754187	0.205684307
SPCC550.07	fah2	fatty-acid amide hydrolase (predicted)	-0.2499848	6.63379205	0.06734084	0.1115517
SPAC1A6.05c	ptl3	triacylglycerol lipase ptl3	-0.2499985	6.87936416	0.02823859	0.05279219
SPAC1952.16	rga9	RhoGAP, GTPase activating protein Rga9 (predicted)	-0.2500512	7.10419811	0.02222482	0.042978089
SPBC23G7.16	ctr6	vacuolar copper exporter Ctr6	-0.250115	6.02882087	0.11145574	0.171829708
SPAC31A2.12	rod1	arrestin/PY protein involved in ubiquitin-mediated endocytc	-0.2503789	7.43714027	0.01886746	0.03733441
SPBC4F6.07c	mak5	ATP-dependent RNA helicase Mak5 (predicted)	-0.2505516	7.08722678	0.05682152	0.096571034
SPAC2G11.12	rqh1	RecQ type DNA helicase Rqh1	-0.2507994	7.79272371	0.0096875	0.020479475
SPAC6B12.14c	SPAC6B12.14c	conserved fungal protein	-0.2509857	7.35766825	0.01690159	0.033728832
SPBC12D12.07c	trx2	mitochondrial thioredoxin Trx2	-0.2511938	5.97898108	0.09779947	0.15387871
SPCC306.08c	mdh1	malate dehydrogenase Mdh1 (predicted)	-0.2515537	8.47134685	0.00526237	0.011881368
SPBC83.12	SPBC83.12	Schizosaccharomyces pombe specific protein	-0.2516433	5.23197667	0.2058824	0.287626713
SPNCRNA.1624	#N/A	#N/A	-0.2517137	6.25219496	0.09618551	0.151760356
SPNCRNA.92	#N/A	#N/A	-0.2518625	6.12665594	0.34484862	0.439785397
SPNCRNA.1414	#N/A	#N/A	-0.2518684	6.92027602	0.05360077	0.091946877
SPNCRNA.1214	#N/A	#N/A	-0.2519253	7.5744007	0.00916992	0.019476059
SPNCRNA.1047	#N/A	#N/A	-0.2520285	5.53151343	0.18489245	0.262890326
SPAPB1E7.10	rpc17	DNA-directed RNA polymerase III complex subunit Rpc17	-0.2520578	5.43884813	0.18585117	0.263977711
SPBC56F2.03	arp10	dynactin complex actin-like protein Arp10 (predicted)	-0.252147	4.62753894	0.37780802	0.473625684
SPAC1250.04c	atl1	alkyltransferase-like protein Atl1	-0.2521884	3.54412046	0.57040151	0.659124175
SPAC31A2.10	SPAC31A2.10	Ran GTPase binding protein (predicted)	-0.2523261	6.0212957	0.10229299	0.159896222
SPBC2D10.10c	fib1	fibrillarin, rRNA methyltransferase	-0.2523912	5.68730707	0.18221342	0.260059205
SPAC630.10	bmt2	rRNA (adenine) methyltransferase activity Bmt2 (predicted	-0.252421	5.77247619	0.17009973	0.245239126
SPAC6F6.12	atg24	autophagy associated PX/BAR domain sorting nexin Atg24	-0.2526701	7.27569474	0.0102994	0.02163859
SPAC20H4.06c	SPAC20H4.06c	G-patch RNA-binding protein, involved in splicing (predictec	-0.2531041	5.73358244	0.16683596	0.24181442
SPBC16E9.19	SPBC16E9.19	proteasome assembly chaperone 3 (predicted)	-0.2536264	6.75901667	0.03468532	0.063237366
SPAC227.11c	yos9	sensor for misfolded ER glycoproteins Yos9 (predicted)	-0.2537213	8.12013882	0.00381518	0.008907959
SPCC1020.03	mmt1	mitochondrial iron ion transmembrane transporter Mmt1 (|	-0.2540394	5.97098514	0.15003599	0.221186077
SPBC646.03	gta1	mitochondrial glutamyl-tRNA amidotransferase alpha subu	-0.2548437	6.27071201	0.21743354	0.301781611
SPNCRNA.803	#N/A	#N/A	-0.2552889	7.74848393	0.00398212	0.009254881
SPBP23A10.13	orc4	origin recognition complex subunit Orc4	-0.255932	7.57207461	0.00705491	0.015420831
SPNCRNA.875	#N/A	#N/A	-0.2562233	5.78573532	0.16135351	0.235069707
SPAC637.09	rex1	3'-5'- exoribonuclease Rex1 (predicted)	-0.2562942	7.22900954	0.01306355	0.026792476
SPBC36.03c	mfs3	plasma membrane spermidine transmembrane transporter	-0.2570275	9.48447541	0.000731	0.002038721
SPBC25B2.04c	mtg1	mitochondrial translation factor (GTPase) Mtg1 (predicted)	-0.2572625	5.3632	0.23016699	0.31667739
SPBC11C11.03	ndc80	NMS complex subunit Ndc80	-0.2573492	6.77125185	0.03468995	0.063237366
SPCC191.07	cyc1	cytochrome c	-0.2575832	8.34234305	0.00508156	0.011511321
SPNCRNA.669	#N/A	#N/A	-0.2576494	5.0847861	0.25842367	0.348923223
SPAC13F5.06c	sec10	exocyst complex subunit Sec10	-0.2578915	6.97127027	0.02892836	0.053992737
SPCC553.01c	dbl2	DNA recombination protein Dbl2	-0.2585599	5.59049201	0.19739792	0.277826851

SPCC16C4.05	pop23	RNase P and RNase MRP subunit Pop23	-0.2585705	4.89295674	0.24147321	0.330296523
SPNCRNA.1615	#N/A	#N/A	-0.2588265	5.73357266	0.12731343	0.192740359
SPBC56F2.04	utp20	U3 snoRNP protein Utp20 (predicted)	-0.2591651	8.97197281	0.0031421	0.007513008
SPCC576.12c	mhf2	CENP-X ortholog, FANCM-MHF complex subunit Mhf2	-0.2591885	7.36200333	0.02589318	0.049027013
SPNCRNA.1398	#N/A	#N/A	-0.2592244	6.26871218	0.20899285	0.291553169
SPBC4C3.04c	dss4	guanyl-nucleotide exchange factor (predicted)	-0.2596045	3.37691196	0.59752788	0.681215823
SPBP16F5.06	nop8	ribosome biogenesis protein Nop8 (predicted)	-0.2597486	6.36547977	0.08140003	0.131386912
SPBC28F2.05c	SPBC28F2.05c	xylose and arabinose reductase (predicted)	-0.2598854	6.45278384	0.07870083	0.127514078
SPBC1604.19c	trs8501	TRAPP complex subunit Trs85a (predicted)	-0.2600816	7.00986986	0.05875422	0.099373793
SPBC12D12.08c	ned8	ubiquitin-like protein modifier for cullin Ned8	-0.260166	5.02242542	0.32200836	0.416274105
SPCC1620.09c	tfg1	transcription factor TFIIF complex alpha subunit Tfg1 (predi	-0.2608139	7.13352363	0.03977828	0.071159614
SPAC1B3.17	clr2	chromatin silencing protein Clr2	-0.2612589	5.9943876	0.08914654	0.142538115
SPAC30D11.09	cwf19	complexed with Cdc5 protein Cwf19	-0.2613221	6.98505518	0.01419582	0.028905608
SPNCRNA.419	#N/A	#N/A	-0.2617403	5.44406767	0.17771477	0.254707029
SPCC777.07	omh3	alpha-1,2-mannosyltransferase Omh3 (predicted)	-0.2618233	7.29565449	0.00656737	0.014491227
SPBC1709.19c	nfu1	mitochondrial [4Fe-4S] cluster transfer protein Nfu1 (predi	-0.2619999	6.87483843	0.04988276	0.086528729
SPAC1783.07c	pap1	transcription factor Pap1/Caf3	-0.2621321	7.97767074	0.0020972	0.005231455
SPAC24C9.14	otu1	ubiquitin-specific cysteine protease, OTU family, Otu1	-0.262386	7.36330685	0.01460126	0.0296248
SPBC106.13	gid9	GID complex subunit Gid9 (predicted)	-0.2623983	6.17196529	0.1557338	0.228201663
SPCC1020.07	SPCC1020.07	pseudouridine-5'-phosphatase (predicted)	-0.2624157	6.01427941	0.10522636	0.163468956
SPCC1672.11c	SPCC1672.11c	P-type ATPase P5 type (predicted)	-0.262681	8.02177939	0.00299189	0.007181584
SPCC24B10.02c	SPCC24B10.02c	NAD/NADH kinase (predicted)	-0.2627264	6.58955355	0.03203308	0.058886148
SPBC8D2.17	gmh4	alpha-1,2-galactosyltransferase (predicted)	-0.2628435	7.59042778	0.01100049	0.022955628
SPAC29E6.07	SPAC29E6.07	Schizosaccharomyces pombe specific protein, expressed du	-0.2630094	2.06196991	0.72579753	0.794059837
SPBC887.03c	noc3	Noc2p-Noc3p complex subunit Noc3	-0.2631365	7.23335495	0.01946217	0.038299613
SPNCRNA.712	#N/A	#N/A	-0.2638821	6.98386408	0.01335984	0.027358924
SPNCRNA.1204	#N/A	#N/A	-0.264024	5.73816898	0.12197178	0.185894273
SPAC6F12.09	rdp1	RNA-directed RNA polymerase Rdp1	-0.2642734	7.81450392	0.00386262	0.00900175
SPCC1620.07c	lnp1	lunapark Lnp1	-0.2644994	6.29051791	0.07398842	0.120992827
SPBC19C7.02	ubr1	N-end-recognizing protein, UBR ubiquitin-protein ligase E3	-0.2645699	8.8202739	0.00084547	0.002311527
SPNCRNA.1042	#N/A	#N/A	-0.265241	6.54148172	0.0457075	0.0801029
SPAC1639.02c	trk2	plasma membrane potassium ion transmembrane transpor	-0.2653021	7.5134847	0.00754112	0.01636395
SPBC1773.02c	bcp1	thioredoxin peroxidase Bcp1	-0.265343	6.82677581	0.02353591	0.04517965
SPAC1D4.03c	aut12	autophagy associated protein Aut12 (predicted)	-0.2656675	6.53656534	0.02825751	0.052813056
SPAC23D3.17	SPAC23D3.17	protease inhibitor 178 family	-0.2657211	5.6807504	0.13082165	0.19734899
SPBC14F5.13c	pho8	vacuolar membrane alkaline phosphatase (predicted)	-0.2657226	7.82616203	0.00696563	0.015256225
SPBC12C2.10c	pst1	Clr6 histone deacetylase complex subunit Pst1	-0.2657707	8.70886607	0.00080204	0.002206089
SPCC576.04	bxi1	BAX inhibitor family protein Bxi1	-0.2660349	6.6482136	0.07998379	0.129377156
SPBC1289.14	SPBC1289.14	adducin (predicted)	-0.2668569	3.63522092	0.47178984	0.566730781
SPAC23A1.09	rbm8	exon junction complex subunit, RNA-binding protein Rbm8	-0.267315	5.83255912	0.11233572	0.172999861
SPCC965.04c	yme1	mitochondrial inner membrane i-AAA protease complex sul	-0.2678246	8.21891404	0.00274946	0.006670256
SPAC589.02c	med13	mediator complex subunit Med13	-0.2678706	6.57545962	0.03164006	0.058372223
SPAC23D3.01	pdp3	PWWP domain protein, involved in chromatin remodeling (|	-0.2681162	6.04812547	0.10711641	0.166004818
SPBC3B8.09	utp3	U3 snoRNP-associated protein Utp3 (predicted)	-0.2686331	7.81608471	0.00607882	0.013531695
SPNCRNA.1475	#N/A	#N/A	-0.2688559	6.67755978	0.02463085	0.046950394
SPBC30B4.06c	ips1	mitochondrial GIDA family tRNA uridine 5-carboxymethylar	-0.2690605	6.74685382	0.14236856	0.211624735
SPNCRNA.585	#N/A	#N/A	-0.269189	5.0474084	0.25624422	0.346530584

SPAC3H5.13	new4	DUF4598 family, human C12orf45 ortholog, domain archite	-0.2693197	5.0802499	0.21619462	0.300368396
SPAC13D6.05	alp11	tubulin specific chaperone cofactor B	-0.2693725	6.8832277	0.04949894	0.085928648
SPAC1093.05	SPAC1093.05	ATP-dependent RNA helicase Hca4 (predicted)	-0.2696983	7.5537258	0.00613485	0.013643026
SPBC1773.16c	SPBC1773.16c	transcription factor, zf-fungal binuclear cluster type(predict	-0.2698359	6.59880066	0.0587334	0.099373793
SPCC1235.10c	sec6	exocyst complex subunit Sec6	-0.2698981	7.11305898	0.0129945	0.026666932
SPBC1271.15c	mti2	mitochondrial translation initiation factor IF-2Mt/Mti2 (pre	-0.269903	7.21702767	0.00669413	0.014718439
SPCC1450.13c	rib5	riboflavin synthase Rib5	-0.2700795	7.16582957	0.02598628	0.049175927
SPBC6B1.06c	ubp14	Lys48-specific deubiquitinase Ubp14	-0.2703009	8.09435189	0.00491938	0.0112065
SPBP8B7.08c	ppm1	leucine carboxyl methyltransferase, involved in regulation o	-0.2705285	6.44596166	0.03399737	0.0622198
SPNCRNA.1280	#N/A	#N/A	-0.2705993	8.2215118	0.00243926	0.00600114
SPAC5H10.11	gmh1	alpha-1,2-galactosyltransferase Gmh1 (predicted)	-0.2708164	4.53078034	0.31458722	0.408621122
SPAC3A12.04c	rpp1	RNase P and RNase MRP subunit p30, Rpp1	-0.2711449	5.40381016	0.22363626	0.308815894
SPAC17C9.14	pex19	Pex19 protein (predicted)	-0.2718172	6.23209698	0.05396618	0.092480452
SPNCRNA.1157	#N/A	#N/A	-0.2718971	4.54534466	0.29802609	0.391065854
SPBC428.10	SPBC428.10	Schizosaccharomyces pombe specific protein	-0.2719646	8.10184147	0.00471777	0.010805934
SPAC23H4.18c	rbx1	SCF complex, Cul4-RING and CLRC ubiquitin ligase ligase E3	-0.2720721	6.26668975	0.05027011	0.087171131
SPNCRNA.1538	#N/A	#N/A	-0.272351	8.09900813	0.00421468	0.009748768
SPAC4F8.11	sea2	SEA complex WD repeat subunit Sea2 (predicted)	-0.2735128	8.92261476	0.00032164	0.000969799
SPNCRNA.1522	#N/A	#N/A	-0.2738402	3.58511539	0.43314023	0.528703004
SPAC31G5.10	eta2	Myb family protein, RNA polymerase I termination factor E	-0.274221	7.0807518	0.0094946	0.020115138
SPNCRNA.678	#N/A	#N/A	-0.274245	5.83660497	0.09582335	0.151287918
SPNCRNA.248	#N/A	#N/A	-0.2744572	5.31679235	0.14599521	0.216071642
SPAC22G7.05	kri1	ribosome biogenesis protein Kri1 (predicted)	-0.2746024	5.83122845	0.0952709	0.150631612
SPNCRNA.1056	#N/A	#N/A	-0.2746555	3.58423141	0.44656969	0.542468178
SPAC6G10.02c	tea3	cell end marker Tea3	-0.2749226	8.03889979	0.00394197	0.009170965
SPNCRNA.1603	#N/A	#N/A	-0.2751293	6.35708786	0.06412259	0.107186003
SPCC364.04c	coy1	CASP family protein involved in Golgi vesicle transport Coy1	-0.2755969	7.50991457	0.00813464	0.017495647
SPCC2H8.04	SPCC2H8.04	mitochondrial ACN9 family protein, implicated in protein as	-0.2756262	5.32822321	0.16475025	0.239147923
SPBC1604.20c	tea2	kinesin family plus-end directed microtubule motor Tea2	-0.275705	6.70491962	0.0446595	0.078448083
SPCC1739.03	hrr1	Helicase Required for RNAi-mediated heterochromatin asse	-0.2758393	7.36914275	0.00380512	0.008889057
SPAC589.07c	atg1801	autophagy associated WD repeat protein Atg18a	-0.2758413	5.70013434	0.14618473	0.216258062
SPBC21D10.06c	map4	cell surface adhesion protein for conjugation Map4	-0.2758838	5.3807118	0.25194245	0.341799911
SPAC458.07	tfa1	transcription factor TFIIE alpha subunit, TFIIEA, Tfa1	-0.2759746	6.60735523	0.06653731	0.110516564
SPNCRNA.648	#N/A	#N/A	-0.2763498	4.55126921	0.3610797	0.456825221
SPNCRNA.1438	#N/A	#N/A	-0.2763989	5.97060273	0.07181644	0.117932167
SPCC338.12	pbi2	vaculoar proteinase B inhibitor Pbi2	-0.2770251	6.65536784	0.05581013	0.095232675
SPAC13D6.04c	btb3	substrate adaptor for cullin 3 ubiquitin ligase Btb3	-0.2771304	6.79540474	0.0158603	0.031856348
SPNCRNA.691	#N/A	#N/A	-0.2772332	6.29625032	0.03836534	0.068994742
SPCC1393.04	fta4	Mis6-Sim4 complex subunit Fta4	-0.2776196	5.53162691	0.14523876	0.215115056
SPBC1706.03	fzo1	mitofusin, mitochondrial dynamin family fusion GTPase prc	-0.2778586	7.2496996	0.01346249	0.02752772
SPBC19G7.13	tbf1	DNA binding factor Trf1	-0.2779283	7.2530272	0.00495612	0.01126466
SPNCRNA.580	#N/A	#N/A	-0.2780209	6.29226125	0.06732731	0.1115517
SPAC1834.06c	pmo25	mo25 family protein Pmo25	-0.2781996	6.47893335	0.13808427	0.206247464
SPBC14F5.05c	sam1	S-adenosylmethionine synthetase	-0.2785209	8.56002307	0.00039586	0.001169691
SPNCRNA.1344	#N/A	#N/A	-0.2788113	6.12223928	0.13527968	0.20281521
SPBC25B2.01	hbs1	Dom34-Hbs1 translation release factor complex subunit Hb:	-0.2788605	6.50809233	0.10323331	0.160976779
SPAC222.15	meu13	Tat binding protein 1(TBP-1)-interacting protein (TBPIP) hor	-0.2789594	4.15273667	0.32368078	0.418039036

SPNCRNA.770	#N/A	#N/A	-0.2789831	8.53855839	0.00112137	0.002972692
SPCC306.07c	SPCC306.07c	U3 snoRNP-associated protein Cic1/Utp30 family (predicte	-0.2790378	5.68776198	0.10023972	0.157354397
SPNCRNA.73	#N/A	#N/A	-0.279262	5.60507041	0.10604897	0.164500397
SPBC32F12.16	gem7	human GEMIN7 ortholog	-0.2794261	4.7945455	0.31147136	0.405192622
SPAC25B8.18	SPAC25B8.18	mitochondrial thioredoxin-related protein (predicted)	-0.2795925	4.62938265	0.2419627	0.330899548
SPCC74.06	mak3	histidine kinase Mak3	-0.2800089	9.65454857	5.07E-05	0.000176296
SPCC622.03c	SPCC622.03c	Schizosaccharomyces pombe specific protein	-0.2800836	6.34736544	0.06694137	0.111079258
SPNCRNA.1577	#N/A	#N/A	-0.2801856	6.74299459	0.03023389	0.056085483
SPAC144.06	apl5	AP-3 adaptor complex subunit Apl5 (predicted)	-0.2804806	7.9785192	0.0013295	0.003465544
SPAC23D3.16	lam4	Ragulator complex subunit, human LAMTOR4 ortholog	-0.2805165	5.28102652	0.13812111	0.206257215
SPNCRNA.1253	#N/A	#N/A	-0.2805481	7.50548989	0.00397063	0.009234486
SPAC4A8.10	rog1	acylglycerol lipase (predicted)	-0.2809045	7.63288122	0.00192864	0.004857288
SPBC530.01	gyp1	GTPase activating protein Gyp1 (predicted)	-0.2821048	6.93364571	0.01675048	0.033466525
SPNCRNA.953	#N/A	#N/A	-0.2823015	5.33821154	0.17673353	0.253460842
SPAC1F12.05	any2	arrestin-related endocytic adaptor Any2 (predicted)	-0.2824735	7.3282365	0.08365201	0.134606743
SPNCRNA.991	#N/A	#N/A	-0.2826325	7.71621487	0.0017561	0.004459043
SPAC328.07c	bsd1	human NEDD4 family-interacting protein ortholog (predicte	-0.2828356	6.84894758	0.02387445	0.045687749
SPBC15D4.14	taf73	transcription factor TFIID complex subunit Taf5-like	-0.2829399	7.14826982	0.01082752	0.022650258
SPAC20G8.03	itr2	myo-inositol transmembrane transporter	-0.2829743	9.78475993	3.71E-05	0.000131855
SPNCRNA.930	#N/A	#N/A	-0.2831194	7.70353739	0.00244028	0.006001478
SPBC215.02	bob1	prefoldin subunit 5 (predicted)	-0.2831823	7.07135603	0.01270314	0.026108385
SPCC1235.04c	nad1	FAD synthetase Nad1 (predicted)	-0.283554	5.50057915	0.1795438	0.257003691
SPAC13D6.01	pof14	F-box protein Pof14	-0.2839399	5.3719138	0.12771455	0.193304609
SPBC1703.14c	top1	DNA topoisomerase I	-0.284184	7.88855332	0.00129623	0.003391319
SPAC806.08c	mod21	gamma tubulin complex subunit Mod21	-0.2846839	5.9678115	0.09524002	0.150617789
SPAC26H5.03	pcf2	CAF assembly factor (CAF-1) complex subunit B, Pcf2	-0.2847299	6.08054832	0.04918804	0.08544406
SPAC630.06c	SPAC630.06c	DUF2011 family conserved fungal protein	-0.2850692	8.45935837	0.00255807	0.006246019
SPAC821.13c	dnf1	trans-Golgi network aminophospholipid translocase (flippa	-0.2853177	9.24054712	7.05E-05	0.000238618
SPNCRNA.1490	#N/A	#N/A	-0.2854264	4.59193104	0.24593185	0.335316812
SPNCRNA.655	#N/A	#N/A	-0.2862168	6.91412002	0.04349505	0.076918865
SPBP8B7.13	vac7	PAS complex phosphatidylinositol phosphate kinase activat	-0.2862527	5.75619499	0.08372132	0.134686426
SPBC31F10.15c	atp15	F1-FO ATP synthase epsilon subunit (predicted)	-0.2863445	6.45323062	0.05467658	0.093556487
SPAC144.05	SPAC144.05	DNA-dependent ATPase/ ubiquitin-protein ligase E3 (predic	-0.2865518	7.68940793	0.00234769	0.005803128
SPAC3C7.07c	ate1	arginine-tRNA protein transferase Ate1 (predicted)	-0.2871096	6.461735	0.03111827	0.057543425
SPAC8C9.07	fyv7	rRNA processing protein Fyv7 (predicted)	-0.2877849	5.85351936	0.12615258	0.191265755
SPNCRNA.1449	#N/A	#N/A	-0.2878666	6.51783136	0.04198101	0.074608219
SPNCRNA.610	#N/A	#N/A	-0.2880423	7.30151784	0.01019812	0.021480508
SPBC19C7.03	cyr1	adenylate cyclase	-0.2882923	8.69711514	0.00016836	0.000533385
SPNCRNA.1592	#N/A	#N/A	-0.2883015	5.67937766	0.09274866	0.147397153
SPAC3H1.03	mug151	mouse transcriptional regulator HCNGP-like, conflicting inf	-0.2891122	5.05435704	0.23132359	0.317883081
SPCC622.11	SPCC622.11	Golgi localized LMBR1-like multi-pass membrane protein,	-0.2891725	6.18270003	0.20063432	0.281334551
SPCC1183.04c	pet127	mitochondrial RNA 5'-end processing Pet127	-0.28925	6.61603648	0.04547601	0.079799969
SPBC211.04c	mcm6	MCM complex subunit Mcm6	-0.2897159	7.95499594	0.00066111	0.001862103
SPNCRNA.1124	#N/A	#N/A	-0.2898504	2.7991866	0.68723405	0.761789817
SPAC1782.07	qcr8	ubiquinol-cytochrome-c reductase complex subunit 7	-0.2898604	7.27264105	0.00497546	0.011304834
SPBC660.07	ntp1	alpha,alpha-trehalase Ntp1	-0.2898747	9.02321585	0.02265596	0.043675301
SPBC11B10.05c	rsp1	random septum position protein, DNAJ domain protein Rsp	-0.2899125	6.0140092	0.10351683	0.161344946

SPCC736.15	pil1	eisosome BAR domain protein Pil1	-0.2900277	9.18689843	0.00013893	0.000447015
SPAC27E2.04c	#N/A	#N/A	-0.290125	2.58434669	0.67896987	0.754349382
SPAC4G8.10	gos1	SNARE Gos1 (predicted)	-0.2902688	6.57719695	0.01683343	0.03361253
SPBC20F10.08c	rtp1	RNA polymerase II nuclear import protein Rtp1 (predicted)	-0.2904492	7.68659177	0.00204548	0.005117453
SPAPJ698.03c	prp12	U2 snRNP-associated protein Sap130	-0.2909436	8.10869564	0.00112194	0.00297305
SPAC22E12.14c	sck2	serine/threonine protein kinase S6K Sck2	-0.2910066	8.21065909	0.00032165	0.000969799
SPCC1281.01	ags1	cell wall alpha-1,3-glucan synthase Ags1	-0.2910356	9.77956915	1.02E-05	4.04E-05
SPBC418.02	SPBC418.02	NatA N-acetyltransferase complex subunit (predicted)	-0.2911916	6.29928926	0.08329452	0.134190151
SPNCRNA.775	#N/A	#N/A	-0.2913685	2.53200273	0.68616803	0.76110406
SPAC4D7.02c	pgc1	phosphatidylglycerol phospholipase C Pgc1 (predicted)	-0.2917273	7.30143171	0.00722582	0.015750067
SPBC713.13	#N/A	#N/A	-0.2922072	3.7986455	0.5018821	0.595208726
SPAPB2B4.02	grx5	mitochondrial [2Fe-2S] cluster assembly and transfer gluta	-0.2922499	7.02135339	0.00659597	0.014540193
SPCC622.02	SPCC622.02	Schizosaccharomyces pombe specific protein	-0.2923763	6.55684999	0.03451873	0.063026556
SPBC2F12.08c	ceg1	mRNA guanylyltransferase Ceg1	-0.2923818	4.64711027	0.2438021	0.333080365
SPAC25B8.11	SPAC25B8.11	transcription factor (predicted)	-0.2924377	7.45597272	0.00349664	0.008263492
SPCC23B6.02c	SPCC23B6.02c	pre-ribosomal factor (predicted)	-0.2926049	4.7440244	0.29659359	0.389862733
SPNCRNA.1084	#N/A	#N/A	-0.2932017	5.0430431	0.17066747	0.245953437
SPCC970.01	rad16	DNA repair endonuclease XPF	-0.2932736	7.19305017	0.0090176	0.019182493
SPBC23G7.14	SPBC23G7.14	Schizosaccharomyces specific protein	-0.2932751	6.81520844	0.0118125	0.024470026
SPBC14C8.07c	cdc18	MCM loader	-0.2936849	6.8557373	0.0215663	0.041835429
SPBC2A9.13	#N/A	#N/A	-0.2939103	4.67691175	0.25074714	0.340585685
SPAC31G5.14	gcv1	glycine decarboxylase T subunit (predicted)	-0.2941353	5.93757957	0.06897371	0.113890856
SPAC3H8.08c	SPAC3H8.08c	transcription factor (predicted)	-0.2942287	6.09627314	0.08207613	0.132446779
SPAC9.10	thi9	plasma membrane thiamine transmembrane transporter Tl	-0.2942974	6.77055101	0.01431597	0.029132822
SPBC1706.01	tea4	tip elongation aberrant protein Tea4	-0.2943503	7.34499174	0.00290167	0.006992149
SPNCRNA.1436	#N/A	#N/A	-0.2945997	6.5267862	0.02216789	0.042904566
SPNCRNA.1176	#N/A	#N/A	-0.2951446	5.8072647	0.10201125	0.159562063
SPCC330.01c	rhp16	Rad16 homolog ATP-dependent DNA helicase/ ubiquitin pro	-0.2953	8.24989803	0.00046248	0.001349558
SPBC30D10.21	mzm1	mitochondrial respiratory chain complex III assembly protei	-0.2953376	4.37126307	0.36508609	0.460587848
SPAC1751.02c	rsm19	mitochondrial ribosomal protein subunit S19 (predicted)	-0.2957483	6.7156077	0.02999218	0.055733693
SPBC14F5.10c	SPBC14F5.10c	ubiquitin-protein ligase E3, unknown biological role, implica	-0.2958514	7.01977582	0.03754091	0.067637243
SPCC306.05c	ins1	INSIG domain protein	-0.2958985	6.13714641	0.09354424	0.14845349
SPAC1565.06c	spg1	GTPase Spg1	-0.2959383	5.96945797	0.10060713	0.157858316
SPAC4F10.06	bud22	ribosome small subunit biogenesis protein, BUD22 family (|	-0.2959475	6.69520731	0.03011813	0.055921936
SPAC12G12.13c	cid14	TRAMP complex poly(A) polymerase subunit Cid14	-0.2959694	7.39703258	0.00321842	0.007676599
SPBC651.05c	dot2	ESCRT II complex subunit Dot2	-0.2960736	6.20602955	0.0324324	0.059568828
SPBC19C2.06c	mug124	Schizosaccharomyces pombe specific protein	-0.2967431	5.71827437	0.19681652	0.277352753
SPBC651.03c	gyp10	GTPase activating protein Gyp10	-0.2968785	7.54747393	0.00199814	0.00501562
SPBC8D2.16c	SPBC8D2.16c	SPOUT domain containing methyltransferase (predicted)	-0.2970562	7.34587111	0.00236587	0.005839588
SPCC1442.13c	sqs2	R3H and G-patch domain protein Sqs2	-0.2971533	5.51826573	0.09794146	0.154066491
SPCC1795.09	yps1	aspartic protease, yapsin family, unknown specificity Yps1	-0.2972805	6.58165539	0.02238281	0.043246749
SPNCRNA.654	#N/A	#N/A	-0.2978551	4.57291846	0.21977492	0.30453438
SPNCRNA.1469	#N/A	#N/A	-0.2978969	7.25561597	0.00573881	0.01286317
SPBC839.02	aly1	arrestin Aly1 related Aly1	-0.2979981	6.68813959	0.05027872	0.087171131
SPAPB21F2.03	slx9	ribosome biogenesis protein Slx9 (predicted)	-0.2983554	5.73055214	0.07372608	0.120660401
SPBC4B4.11	SPBC4B4.11	mitochondrial conserved fungal protein, implicated in mitoc	-0.2988105	7.31894547	0.01824266	0.036203343
SPAC56F8.09	rrp8	rRNA methyltransferase Rrp8	-0.2989144	5.88163005	0.05110203	0.088261248

SPAC823.13c	she9	mitochondrial inner membrane protein She9 (predicted)	-0.2989521	5.36512564	0.22296758	0.308080074
SPAC23D3.14c	aah2	alpha-amylase homolog Aah2 (predicted)	-0.2992351	6.67319164	0.01361831	0.027812903
SPAC1006.01	psp3	vacuolar serine protease Psp3 (predicted)	-0.2993155	8.85725097	0.00068587	0.001926258
SPBP16F5.02	mcs2	TFIIH complex cyclin Mcs2	-0.2995506	5.15413406	0.27748333	0.369669937
SPNCRNA.939	#N/A	#N/A	-0.2996233	5.23895951	0.14418144	0.213880876
SPAC328.04	spg4	microtubule severing ATPase Spg4 (predicted)	-0.2997168	6.53894556	0.02782358	0.052145268
SPCC1494.03	arz1	human RAP1 GTPase-GDP dissociation stimulator ortholog,	-0.2997818	6.09682452	0.05617211	0.095682409
SPBC21B10.13c	yox1	MBF complex corepressor Yox1	-0.2998226	6.66469824	0.07280264	0.119436345
SPBC887.12	SPBC887.12	P-type ATPase (predicted)	-0.2999597	8.42155371	0.00071641	0.002002125
SPNCRNA.708	#N/A	#N/A	-0.3001024	5.99274948	0.04718904	0.082381076
SPAC637.04	ypp1	phosphatidylinositol-4 kinase plasma membrane scaffold E	-0.3006036	7.37522827	0.00297993	0.007155398
SPBC3B8.08	SPBC3B8.08	Sjogren's syndrome/scleroderma autoantigen 1 family, imp	-0.3007542	5.21214896	0.21054976	0.293604738
SPAC4D7.01c	sec71	Sec7 domain protein, ARF GEF (predicted)	-0.3014206	8.78204883	0.00026951	0.000823177
SPCC1183.07	rrp5	U3 snoRNP-associated protein Rrp5 (predicted)	-0.3014397	8.33485465	0.00015851	0.000505232
SPAC750.04c	ftm3	sub-telomeric 5Tm protein family Ftm3	-0.3017785	6.0703534	0.07492293	0.122282135
SPBP8B7.24c	atg8	autophagy associated protein Atg8	-0.3020382	6.10692666	0.04016433	0.071774749
SPBC947.13	rba50	RNA polymerase II associated protein (predicted)	-0.3022311	6.22772607	0.0482763	0.083999033
SPCC663.18	SPCC663.18	GTPAse interacting protein involved in vesicle tethering at t	-0.3022739	7.56194073	0.00104506	0.002789966
SPCC290.02	rpc34	DNA-directed RNA polymerase III complex subunit Rpc34 (p	-0.3023407	6.2503701	0.03573607	0.064883657
SPNCRNA.864	#N/A	#N/A	-0.302404	5.51444619	0.07761469	0.125974909
SPAP7G5.06	per1	plasma membrane amino acid transmembrane transporter	-0.3025991	8.37618126	0.00066016	0.001860201
SPAC30D11.06c	hfl1	organic solute transmembrane transporter (predicted)	-0.3027643	7.44359217	0.00120094	0.003166372
SPNCRNA.261	#N/A	#N/A	-0.3027925	1.73057478	0.83382643	0.881358936
SPNCRNA.426	#N/A	#N/A	-0.3029347	6.04137693	0.06041946	0.101786933
SPAPB17E12.02	yip12	SMN family protein Yip12	-0.3030035	6.42220078	0.10561551	0.163977536
SPNCRNA.839	#N/A	#N/A	-0.3036661	7.27247511	0.00420899	0.009738923
SPCC4F11.04c	imt2	mannosyltransferase Imt2	-0.3039024	7.93413743	0.00055125	0.001575483
SPBC577.15c	sim3	NASP family CENP-A chaperone	-0.3040565	6.36385378	0.05516337	0.09429459
SPNCRNA.1296	#N/A	#N/A	-0.3040752	4.05306158	0.33345782	0.427743725
SPNCRNA.1105	#N/A	#N/A	-0.3046153	7.85572095	0.00097658	0.00262673
SPAC31A2.15c	dcc1	Ctf18 RFC-like complex subunit Dcc1	-0.3047487	7.21305448	0.00600649	0.013405766
SPAC22E12.06c	gmh3	alpha-1,2-galactosyltransferase Gmh3	-0.3047536	6.91750713	0.01564743	0.031475242
SPCC306.04c	set1	histone lysine methyltransferase Set1	-0.3052516	7.77218701	0.00361309	0.008490002
SPBC1773.15	dal52	dipeptide transmembrane transporter Dal5h2 (predicted)	-0.3053741	6.12710348	0.04416559	0.077747957
SPCC338.06c	SPCC338.06c	heat shock protein Hsp20 family (predicted)	-0.3055339	6.46066706	0.01676297	0.03348165
SPAC15A10.03c	rad54	DNA-dependent ATPase Rad54/Rhp54	-0.305554	6.0371156	0.10765429	0.166762449
SPNCRNA.1536	#N/A	#N/A	-0.3059617	3.46345844	0.49360666	0.588265034
SPCC1393.06c	ipi1	Rix1 complex, Armadillo-type fold Ipi1	-0.3060573	6.33242671	0.05367628	0.092053204
SPACUNK12.02c	cmk1	calcium/calmodulin-dependent protein kinase Cmk1	-0.3061613	7.22285598	0.00213196	0.005316225
SPAC23G3.02c	sib1	ferrichrome synthetase Sib1	-0.3064515	10.2349111	1.39E-06	6.29E-06
SPAC167.09	pga1	GPI-mannosyltransferase II complex subunit Pga1 (predicte	-0.3069284	6.39247016	0.03108837	0.05750376
SPAC29E6.03c	uso1	ER to Golgi tether Uso1 (predicted)	-0.3072318	8.31517057	0.00080451	0.002211092
SPAC22F3.13	tsc1	hamartin	-0.3072356	8.73751492	3.82E-05	0.000135572
SPAC20H4.10	ufd2	ubiquitin-protein ligase E4 Ufd2 (predicted)	-0.3072427	8.03824528	0.00029021	0.000879675
SPAC1002.14	itt1	ubiquitin-protein ligase E3 involved in regulation of cytoplas	-0.3073829	6.65995637	0.01112105	0.023180704
SPBC28F2.06c	mdm12	ERMES complex subunit Mdm12	-0.3076341	5.56820239	0.09657938	0.152134885
SPBC1718.04	sct1	glycerol-3-phosphate O-acyltransferase Sct1/Gpt1 (predicte	-0.3077559	7.64318918	0.00110315	0.002933538

SPBP35G2.03c	sgo1	meiotic inner centromere protein, shugoshin, Sgo1	-0.3079566	4.60867574	0.30752651	0.40084093
SPAC4G8.11c	atp10	mitochondrial F1-FO ATPase assembly protein (predicted)	-0.3079857	6.59334195	0.01089116	0.022762383
SPNCRNA.1086	#N/A	#N/A	-0.3081061	7.48504402	0.00423958	0.009796378
SPAC22E12.08	rrn10	RNA polymerase I upstream activation factor complex subu	-0.3085718	6.63558783	0.01328907	0.027222189
SPNCRNA.1146	#N/A	#N/A	-0.3087913	4.70737492	0.21828227	0.302712627
SPAC16A10.05c	dad1	DASH complex subunit Dad1	-0.3088346	3.77543643	0.41408812	0.509100214
SPCP31B10.05	tdp1	tyrosyl-DNA phosphodiesterase Tdp1	-0.3091087	6.40383083	0.02573435	0.048794158
SPNCRNA.1553	#N/A	#N/A	-0.3092697	5.56747836	0.07576698	0.123495638
SPBP23A10.03c	sdh7	mitochondrial respiratory chain complex II assembly ACN9 f	-0.309288	6.60655051	0.01142203	0.023755169
SPBC19C2.09	sre1	sterol regulatory element binding protein, transcription fact	-0.3096718	8.02999558	0.00157519	0.004040397
SPAC15A10.15	sgo2	inner centromere protein, shugoshin Sgo2	-0.3097036	6.72948191	0.007889	0.017015732
SPAC1834.08	mak1	histidine kinase Mak1	-0.3099211	8.79554594	2.13E-05	7.94E-05
SPBC27B12.03c	erg32	C-5 sterol desaturase Erg32	-0.3104245	6.57585846	0.02264832	0.043672951
SPNCRNA.667	#N/A	#N/A	-0.3105497	3.68396009	0.37895727	0.474743042
SPAC19A8.10	rfp1	SUMO-targeted ubiquitin-protein ligase subunit Rfp1	-0.3105604	6.75428866	0.01190745	0.024636733
SPAC7D4.15c	ost4	oligosaccharyltransferase subunit Ost4 (predicted)	-0.3106647	4.72586378	0.28342674	0.376335409
SPAC688.02c	mis14	NMS complex subunit Mis14/Nsl1	-0.3110264	5.6074367	0.08401845	0.135100558
SPAC17A5.18c	rec25	meiotic recombination protein Rec25	-0.3110358	6.75924108	0.00790963	0.017054833
SPNCRNA.929	#N/A	#N/A	-0.3110687	4.07087704	0.39566091	0.490968719
SPAC12B10.16c	mug157	alpha-mannosidase GH125 family Mug157 (predicted)	-0.3111705	7.93778263	0.00075754	0.002102387
SPBC21C3.20c	git1	C2 domain protein Git1	-0.3112796	8.05396944	0.00015769	0.000503079
SPAC19E9.03	pas1	cyclin Pas1	-0.3123386	8.41712403	0.04610181	0.080710781
SPAC1782.05	ypa2	protein phosphatase type 2A regulator, PTPA family Ypa2	-0.3123809	6.36899451	0.02245304	0.043357804
SPBC215.03c	csn1	COP9/signalosome complex subunit Csn1	-0.3129177	7.28115056	0.00284261	0.006869309
SPBC23E6.01c	cxr1	splicing factor Cxr1	-0.3129533	7.50246693	0.02531671	0.048109524
SPBC8D2.01	gsk31	serine/threonine protein kinase Gsk31 (predicted)	-0.3130368	7.43161097	0.00214817	0.005354682
SPNCRNA.1640	#N/A	#N/A	-0.3131692	7.11270548	0.00483566	0.011046206
SPAC2G11.14	taf111	transcription factor TFIID complex subunit Taf111	-0.3131857	8.42095238	4.23E-05	0.000148531
SPCC569.06	SPCC569.06	Schizosaccharomyces specific multicopy membrane protein	-0.3131953	7.16068056	0.00628544	0.013932386
SPCC191.08	lto1	CIA machinery involved in ribosome biogenesis protein Lto1	-0.3131971	5.100968	0.1439415	0.213636183
SPBC3B9.08c	mnh1	exon junction complex subunit, Mago-nashi homolog Mnh1	-0.3133211	6.6616379	0.01164905	0.024179339
SPBC16A3.06	tad1	tRNA specific adenosine-37 deaminase Tad1 (predicted)	-0.3134934	6.36596623	0.05672867	0.096461422
SPBP8B7.28c	stc1	CLRC ubiquitin ligase complex subunit, LIM-like Stc1	-0.3137882	4.55777734	0.35699024	0.452555623
SPNCRNA.631	#N/A	#N/A	-0.3146047	5.3484651	0.16920469	0.244362879
SPAC167.07c	hul5	HECT-type ubiquitin-protein ligase E3 (predicted)	-0.314628	7.36172153	0.00230189	0.005704425
SPBC8D2.11	SPBC8D2.11	Schizosaccharomyces specific protein	-0.3150711	6.71058172	0.0065717	0.014496072
SPCC1672.04c	cox19	mitochondrial copper chaperone for cytochrome c oxidase C	-0.315448	4.95715146	0.22886549	0.315077815
SPBC530.06c	clu1	translation initiation factor 3 complex subunit Clu1 (predicte	-0.3160927	7.57692323	0.0004753	0.001384009
SPAC8F11.07c	cdc24	DNA replication protein Cdc24	-0.3163737	5.58708173	0.10300635	0.160696512
SPNCRNA.1178	#N/A	#N/A	-0.3164056	6.82400734	0.02099674	0.040858677
SPAC31A2.16	gef2	RhoGEF Gef2	-0.3168973	7.13415205	0.00647381	0.014307973
SPNCRNA.1687	#N/A	#N/A	-0.3170348	8.99138156	0.00058644	0.001666268
SPNCRNA.1264	#N/A	#N/A	-0.317061	6.3449238	0.02952472	0.054970097
SPAC1F7.03	pkd2	plasma membrane TRP-like calcium ion channel Pkd2	-0.3171027	8.76283554	1.93E-05	7.25E-05
SPNCRNA.514	#N/A	#N/A	-0.3171078	4.55337951	0.19714539	0.277575512
SPBC30D10.10c	tor1	serine/threonine protein kinase Tor1	-0.317555	8.58167387	0.00053223	0.001530143
SPNCRNA.586	#N/A	#N/A	-0.3176564	5.73963741	0.05672756	0.096461422

SPBC17A3.10	pas4	peroxisomal ubiquitin-protein ligase E3 involved in peroxiso	-0.3178288	5.93978898	0.09391382	0.148866425
SPAC1610.04	mug99	mitochondrial conserved fungal membrane protein Mug99	-0.3178335	6.6394812	0.06328243	0.10596382
SPBP19A11.06	lid2	Lid2 complex PHD finger and jmjC subunit Lid2	-0.3179445	7.45299207	0.00077492	0.002141025
SPAC17H9.10c	ddb1	Cul4-RING E3 adaptor Ddb1	-0.3179513	8.22684722	0.00010771	0.000353241
SPNCRNA.1135	#N/A	#N/A	-0.3179633	5.8998715	0.05797842	0.09831626
SPBC16D10.10	tad2	tRNA specific adenosine deaminase subunit Tad2	-0.3182405	7.02790537	0.01175485	0.024358029
SPCC830.04c	mug128	Schizosaccharomyces specific protein	-0.3182748	3.88612485	0.46809103	0.562983286
SPNCRNA.959	#N/A	#N/A	-0.3186702	5.2751838	0.08774687	0.140465174
SPNCRNA.1404	#N/A	#N/A	-0.3186704	5.12196214	0.11705849	0.17949144
SPCC61.03	SPCC61.03	NADHX dehydratase (predicted)	-0.3187466	7.42292953	0.00245977	0.006042855
SPAC144.14	klp8	kinesin-like protein Klp8	-0.3188002	7.38860857	0.00091798	0.002487786
SPAC13G7.09c	SPAC13G7.09c	conserved fungal protein	-0.319033	4.65432384	0.22111179	0.305830693
ScpofMp09	#N/A	#N/A	-0.3193427	7.92778468	0.00102822	0.002750418
SPNCRNA.1298	#N/A	#N/A	-0.3198669	3.65946433	0.34672288	0.441686671
SPCC895.04c	ufe1	SNARE Ufe1 (predicted)	-0.3199566	6.36467674	0.01525895	0.030784799
SPAC1F7.07c	fip1	plasma membrane iron transmembrane transporter Fip1	-0.3202516	6.63884341	0.07696562	0.125131432
SPCC126.09	zip2	vacuolar zinc exporter (predicted)	-0.3203446	7.12247464	0.00932378	0.019784323
SPNCRNA.77	#N/A	#N/A	-0.3208612	5.04532194	0.12857234	0.194300418
SPBC16C6.06	vps10	sorting receptor for vacuolar proteins, Vps10	-0.3211616	9.29658516	0.00020465	0.000640583
SPBC6B1.04	mde4	microtubule-site clamp monopolin complex subunit Mde4	-0.3212196	6.7075596	0.01240211	0.02556691
SPCC16C4.20c	hap2	Ino80 complex, HMG box protein Hap2	-0.32129	5.55683008	0.12033797	0.183939778
SPCC188.08c	ubp5	ubiquitin C-terminal hydrolase Ubp5	-0.3216373	8.30540885	0.00113126	0.002996589
SPCC1620.10	cwf26	complexed with Cdc5 protein Cwf26	-0.3218871	5.98342209	0.06067108	0.102093652
SPAC1006.03c	red1	RNA elimination defective protein Red1	-0.3222922	7.43136754	0.00250735	0.006144215
SPNCRNA.656	#N/A	#N/A	-0.3226146	3.43527673	0.36551347	0.460956103
SPBC31F10.02	SPBC31F10.02	acyl-CoA hydrolase (predicted)	-0.3226764	6.72046486	0.00815003	0.017517683
SPAC630.05	gyp7	GTPase activating protein Gyp7 (predicted)	-0.3232168	8.37138911	0.00061285	0.001734071
SPAC20H4.02	dsc3	Golgi Dsc E3 ligase complex subunit Dsc3	-0.3233605	6.9987674	0.00294038	0.007072912
SPCC306.03c	cnd2	condensin complex non-SMC subunit Cnd2	-0.3239119	6.95859378	0.00736189	0.016026114
SPNCRNA.1099	#N/A	#N/A	-0.3239543	4.37881225	0.21569708	0.299799549
SPCC1739.04c	dms1	meiotic spindle pole body protein Dms1	-0.3240806	5.802459	0.06589146	0.109604343
SPNCRNA.169	#N/A	#N/A	-0.3244286	2.23569279	0.64805872	0.727249276
SPAC1002.03c	gls2	glucosidase II alpha subunit Gls2	-0.3249292	8.36393509	0.00061853	0.001748702
SPAC2F7.16c	pld1	phospholipase D, Pld1	-0.3254131	9.20935218	8.09E-06	3.27E-05
SPBP23A10.09	psf1	GINS complex subunit Psf1	-0.3255817	5.2377779	0.1052399	0.163468956
SPBC20F10.01	gar1	box H/ACA snoRNP complex subunit Gar1	-0.3256735	4.02618408	0.2885451	0.381123714
SPAC6F12.11c	sfc1	transcription factor TFIIIC complex A box associated subunit	-0.3262064	7.14094517	0.00233675	0.005778193
SPNCRNA.807	#N/A	#N/A	-0.3263109	5.39397544	0.07308347	0.119839284
SPAC144.07c	gpn2	conserved GTPase Gpn2 (predicted)	-0.3264666	6.79462896	0.00503157	0.011420895
SPBC725.07	pex5	peroxisomal targeting signal receptor Pex5 (predicted)	-0.3267208	7.1925659	0.0084121	0.018018364
SPAC17G6.12	cul1	cullin 1	-0.3267963	7.32930341	0.00333497	0.007918112
SPCC1840.10	lsm8	Lsm2-8 complex Lsm8 (predicted)	-0.3268206	5.34724103	0.08941711	0.142836478
SPBC21H7.03c	SPBC21H7.03c	multiple inositol polyphosphate phosphatase (predicted)	-0.3270773	6.78456403	0.00641411	0.014180635
SPAC1F5.03c	SPAC1F5.03c	FAD-dependent oxidoreductase involved in late endosome t	-0.3276161	7.43807498	0.00043846	0.001284982
SPAC8C9.19	SPAC8C9.19	DUF5310 family conserved fungal membrane protein	-0.3276326	3.2040057	0.44111441	0.537087768
SPBC902.02c	ctf18	Ctf18 RFC-like complex subunit Ctf18	-0.3285848	6.772149	0.02453867	0.046806722
SPAC23E2.02	lsd2	histone demethylase SWIRM2 (predicted)	-0.329121	8.00410043	0.00048568	0.00141122

SPAC3A11.04	sei1	seipin, lipid droplet associated protein Sei1 (predicted)	-0.3298462	6.32996744	0.03965128	0.07098655
SPAC1B3.10c	SPAC1B3.10c	SEL1 repeat protein, ERAD E3 ligase adaptor subunit (predic	-0.329883	6.85714036	0.00594207	0.013286398
SPAC12B10.08c	til1	mitochondrial tRNA(Ile)-lysidine synthetase Til1 (predicted)	-0.3301776	5.77903981	0.04402156	0.077622802
SPBC800.14c	SPBC800.14c	mitochondrial DUF1772 family protein, multimembrane spa	-0.3303413	6.08324763	0.03159427	0.058328539
SPAC3H5.09c	SPAC3H5.09c	mitochondrial protein, human KIAA0100 ortholog (predictec	-0.3303821	8.71300798	0.00023023	0.000713761
SPNCRNA.1544	#N/A	#N/A	-0.3305374	6.32085753	0.02358847	0.045267769
SPBC16H5.14c	SPBC16H5.14c	short chain dehydrogenase DHRS3 family, implicated in lipi	-0.3306382	6.73645816	0.01141283	0.023743301
SPBPB10D8.04c	SPBPB10D8.04c	transmembrane transporter (predicted)	-0.3307104	7.7770173	0.04338782	0.076749177
SPBC3H7.03c	kgd1	2-oxoglutarate dehydrogenase (lipoamide) (e1 component	-0.3307173	9.81666887	7.83E-05	0.000262506
SPNCRNA.1144	#N/A	#N/A	-0.3313724	6.89317285	0.00851117	0.01820847
SPAC926.02	SPAC926.02	TPR repeat protein	-0.3315559	6.47339687	0.0081793	0.017563938
SPAC24H6.03	cul3	cullin 3	-0.3316201	7.04785356	0.00704977	0.015415709
SPNCRNA.976	#N/A	#N/A	-0.3320639	2.53328561	0.64350918	0.722852727
SPAC2G11.13	atg22	vacuolar amino acid transmembrane transporter Atg22	-0.3321374	7.25301258	0.00310696	0.007440316
SPAC8F11.03	msh3	MutS protein homolog 3	-0.3321739	7.05115485	0.00699427	0.015309094
SPBC1921.07c	sgf29	SAGA complex subunit Sgf29	-0.33233	6.02977973	0.05679856	0.09655613
SPAP27G11.02	mgr3	TPR repeat protein, involved in mitochondrial protein turnov	-0.3324083	6.48447942	0.03186589	0.058685568
SPCC962.05	ast1	nuclease, XP-G family protein Ast1	-0.3325009	5.23805153	0.07493262	0.122282135
SPAP27G11.03	cdc123	translation initiation factor eIF2 assembly protein	-0.3326437	5.88628838	0.05124371	0.088483493
SPNCRNA.1550	#N/A	#N/A	-0.3332042	4.77343997	0.17682393	0.253511474
SPNCRNA.1418	#N/A	#N/A	-0.3333915	5.83118922	0.04438115	0.077999406
ScpofMt30	#N/A	#N/A	-0.33369	2.7546035	0.45326021	0.548344124
SPBC19C2.08	prp38	U4/U6 x U5 tri-snRNP complex subunit Prp38	-0.3337159	4.98317297	0.15269876	0.224479375
SPAC6G9.11	syb1	SNAP receptor, synaptobrevin family	-0.3338389	7.73397992	0.01352722	0.027643457
SPNCRNA.1293	#N/A	#N/A	-0.3338957	3.34672502	0.44976151	0.545177606
SPCP31B10.02	SPCP31B10.02	mitochondrial oxidoreductase-like protein, human OXLD1 or	-0.3339587	5.52501561	0.06911126	0.114012643
SPAC8C9.03	cgs1	cAMP-dependent protein kinase regulatory subunit Cgs1	-0.3340224	8.87087523	0.00067129	0.00188923
SPNCRNA.895	#N/A	#N/A	-0.3341351	5.72940079	0.05311163	0.091280675
SPAP14E8.03	bos1	SNARE Bos1 (predicted)	-0.3343216	6.53477558	0.01442899	0.02932774
SPBC13G1.12	did2	ESCRT III complex subunit Did2 (predicted)	-0.3345801	6.44223259	0.02368228	0.045396603
SPNCRNA.1635	#N/A	#N/A	-0.3345801	6.44223259	0.02368228	0.045396603
SPNCRNA.709	#N/A	#N/A	-0.3350714	3.58433304	0.35684714	0.452458507
SPBPJ4664.06	gpt1	UDP-glucose-glycoprotein glucosyltransferase Gpt1	-0.3365681	8.3531957	1.78E-05	6.74E-05
SPAC3A12.08	SPAC3A12.08	acyl-coenzyme A thioesterase	-0.3367364	5.76573289	0.08347766	0.134421545
SPBC28F2.07	sfr1	Swi five-dependent recombination mediator Sfr1	-0.3368104	5.17154352	0.08294443	0.133721117
SPAC589.09	csr101	sec14 cytosolic factor family, phospholipid-intermembrane	-0.3370872	6.40078634	0.01563685	0.031465549
SPBC11B10.08	SPBC11B10.08	WW domain containing conserved fungal protein	-0.3377837	5.91855177	0.02427648	0.04635281
SPAC3H8.09c	nab3	poly(A) binding protein Nab3 (predicted)	-0.3378055	6.22774445	0.02574502	0.048800805
SPCC777.17c	SPCC777.17c	mitochondrial ribosomal protein subunit L9 (predicted)	-0.3378719	5.42249095	0.05959298	0.100528072
SPNCRNA.1089	#N/A	#N/A	-0.3379271	4.13364422	0.31469414	0.408681992
SPCC622.01c	SPCC622.01c	Schizosaccharomyces pombe specific protein	-0.3383172	6.50908229	0.0109816	0.022923245
SPBC336.11	vps52	GARP complex subunit Vps52 (predicted)	-0.3383702	7.47487761	0.00675349	0.014829784
SPCC622.10c	sec5	exocyst complex subunit Sec5 (predicted)	-0.3385609	7.79193563	0.00012399	0.000402739
SPBC405.03c	SPBC405.03c	transmembrane transporter (predicted)	-0.3386579	5.4958969	0.09566721	0.151082695
SPBC19C7.10	bqt4	bouquet formation protein Bqt4	-0.3387465	7.8970494	0.00120194	0.003166555
SPBC800.02	whi5	cell cycle transcriptional repressor Whi5 (predicted)	-0.339008	5.24605155	0.11227554	0.172976007
SPCC1235.11	mpc1	mitochondrial carrier, pyruvate Mpc1 (predicted)	-0.339072	7.5967078	0.00124467	0.003267743

SPAC1039.04	SPAC1039.04	carboxylic acid transmembrane transporter (predicted)	-0.3390898	6.11246939	0.05266823	0.090621317
SPAC2G11.04	SPAC2G11.04	RNA-binding protein, G-patch type, splicing factor 45 orthol	-0.3391231	4.45999377	0.24398301	0.333260615
SPNCRNA.1150	#N/A	#N/A	-0.3393012	7.58672257	0.00028895	0.000876235
SPAC56F8.10	met9	methylenetetrahydrofolate reductase Met9	-0.3397181	7.39934823	0.0008686	0.002366216
SPNCRNA.1423	#N/A	#N/A	-0.3397369	1.53150008	1	1
SPAC1687.18c	ssl3	cohesin loading factor Ssl3	-0.3404815	7.33351976	0.00267601	0.006501335
SPCC5E4.06	smc6	Smc5-6 complex SMC P-loop ATPase subunit Smc6	-0.3409961	7.58596617	0.00026574	0.000813486
SPAC17G8.10c	dma1	mitotic spindle checkpoint ubiquitin ligase Dma1	-0.3414151	6.8125369	0.00262123	0.006377358
SPBP8B7.32	#N/A	#N/A	-0.3415271	8.00912391	0.00419776	0.009716244
SPNCRNA.670	#N/A	#N/A	-0.3416332	3.90521961	0.36145762	0.457111898
SPBC1773.08c	omh4	alpha-1,2-mannosyltransferase Omh4 (predicted)	-0.3419345	5.99254695	0.03138627	0.057975996
SPAC19B12.12c	yip11	SMN family protein Yip11	-0.3431833	5.95532333	0.05316015	0.091329156
SPAC144.04c	spe1	ornithine decarboxylase Spe1 (predicted)	-0.3434478	8.63623042	1.96E-05	7.36E-05
SPNCRNA.1556	#N/A	#N/A	-0.3437434	3.7207914	0.3224545	0.416751408
SPBC25H2.13c	cdc20	DNA polymerase epsilon catalytic subunit Pol2	-0.3437684	8.50507868	0.0015506	0.003981826
SPNCRNA.1061	#N/A	#N/A	-0.3438266	6.84880405	0.00738746	0.016061226
SPBC56F2.07c	SPBC56F2.07c	ribosome biogenesis factor recycling AAA family ATPase (p	-0.344388	6.8659767	0.00226945	0.005632252
SPNCRNA.493	#N/A	#N/A	-0.3447376	1.53158592	1	1
SPAC13F5.02c	taf7	transcription factor TFIID complex subunit Taf7	-0.3448909	7.0370222	0.00219434	0.005461776
SPNCRNA.1501	#N/A	#N/A	-0.3451317	4.23986586	0.27327612	0.364850695
SPAC323.06c	uba5	NEDD8 activating enzyme E1-type Uba5 (predicted)	-0.3457943	6.01417021	0.03347902	0.06135866
SPNCRNA.1626	#N/A	#N/A	-0.3460369	8.81655243	9.46E-06	3.76E-05
SPBC8D2.12c	tac1	mitochondrial Cox1 translational activator Tac1 (predicted)	-0.3469692	6.4044485	0.01385437	0.028261078
SPNCRNA.763	#N/A	#N/A	-0.3470185	7.39569804	0.00044987	0.001315024
SPNCRNA.254	#N/A	#N/A	-0.3470251	5.58751065	0.03786056	0.068177055
SPBC14F5.07	doa10	ER ubiquitin-protein ligase E3 Doa10 (predicted)	-0.347151	8.25762889	0.00025341	0.000778532
SPAC1751.01c	gti1	gluconate transmembrane transporter inducer Gti1	-0.3473094	7.3494739	0.00084793	0.002316393
SPAC22H10.11c	crf1	transcriptional corepressor for ribosomal proteins via TOR s	-0.347425	8.20711704	2.42E-05	8.95E-05
SPCC18B5.01c	bfr1	plasma membrane brefeldin A efflux transporter Bfr1	-0.3476966	8.46802491	2.77E-05	0.000101066
SPBC16A3.19	eaf7	histone acetyltransferase complex subunit Eaf7	-0.3477302	4.93483653	0.21195716	0.295325236
SPAC11E3.01c	swr1	SNF2 family ATP-dependent DNA helicase Swr1	-0.3480602	8.28012204	5.16E-05	0.000178739
SPNCRNA.757	#N/A	#N/A	-0.3484386	3.95603879	0.30530575	0.398849227
SPBC947.08c	hip4	histone promoter control protein Hip4	-0.3488457	6.04579233	0.01896532	0.037484463
SPAPB21F2.02	dop1	Dopey family protein Dop1	-0.3493076	8.93851354	2.59E-05	9.53E-05
SPBPB10D8.05c	SPBPB10D8.05c	transmembrane transporter (predicted)	-0.3496341	7.83256456	0.04087565	0.072854581
SPAC4F8.04	SPAC4F8.04	Brix domain protein Rpf1 (predicted)	-0.3496894	6.42522754	0.01654803	0.033110655
SPNCRNA.321	#N/A	#N/A	-0.3499107	3.95519002	0.34307746	0.437772762
SPNCRNA.1542	#N/A	#N/A	-0.3500988	5.42070985	0.16158832	0.235361408
SPCC1919.14c	bdp1	transcription factor TFIIIB complex subunit Bdp1 (predicted)	-0.3503272	6.37406809	0.03812687	0.068638443
SPNCRNA.436	#N/A	#N/A	-0.3503436	4.34817517	0.21676902	0.300982082
SPAC3H1.01c	orc3	origin recognition complex subunit Orc3	-0.3523376	7.24676893	0.00365976	0.008578939
SPBC3B9.03	srp101	signal recognition particle receptor alpha subunit Srp101 (p	-0.3528583	7.24130937	0.00474657	0.01086273
SPCC663.10	trm44	tRNA (uracil) methyltransferase Trm44 (predicted)	-0.3529258	7.98291973	8.10E-05	0.000270587
SPBPB10D8.06c	SPBPB10D8.06c	transmembrane transporter (predicted)	-0.3529341	7.84016662	0.03815127	0.06864605
SPAC6B12.13	SPAC6B12.13	protein phosphatase type I regulatory subunit (predicted)	-0.3530151	6.96115443	0.00180427	0.004567728
SPCPB1C11.03	SPCPB1C11.03	cysteine transmembrane transporter (predicted)	-0.3530848	6.86793676	0.00143395	0.003707449
SPAC323.08	rmp1	RNase MRP subunit Rmp1	-0.3533318	4.91546935	0.14823212	0.218953676

SPAC11E3.09	pyp3	protein-tyrosine phosphatase Pyp3	-0.3544501	6.17990158	0.03021743	0.056075704
SPAC630.07c	SPAC630.07c	Schizosaccharomyces specific protein	-0.3550838	5.82685363	0.0275762	0.051795765
SPAC17H9.03c	rdl1	RAD51D-like protein 1	-0.3552424	5.20986883	0.13771539	0.205832024
SPAC750.06c	SPAC750.06c	S. pombe specific DUF999 protein family 4	-0.3554212	5.62050093	0.06029233	0.101632226
SPCC126.02c	pku70	Ku domain protein Pku70	-0.3554567	6.62646235	0.00459492	0.010552962
SPAC3G9.02	oar2	3-oxoacyl-[acyl-carrier-protein] reductase Oar2 (predicted)	-0.3557289	5.74486925	0.05254043	0.090470049
SPNCRNA.783	#N/A	#N/A	-0.3557653	6.01021747	0.02858625	0.053412806
SPCC1840.12	opt3	OPT oligopeptide transmembrane transporter family protei	-0.355906	6.47506208	0.01133889	0.023611112
SPCC18B5.08c	ism1	mitochondrial isoleucine-tRNA ligase (predicted)	-0.3559174	7.43217653	0.00115418	0.003052531
SPAC17A5.01	pex6	peroxin-6 (predicted)	-0.356326	7.03259741	0.00149591	0.00385302
SPBC409.23	mim2	mitochondrial outer membrane translocase complex assem	-0.3564195	5.26036209	0.08339523	0.1343206
SPAC823.08c	rrp3	ATP-dependent RNA helicase Rrp3 (predicted)	-0.3567975	6.74332789	0.00780878	0.016858739
SPNCRNA.1088	#N/A	#N/A	-0.356837	3.59711573	0.31875093	0.413083239
SPBC1778.05c	lam2	Ragulator complex subunit, human LAMTOR2 ortholog	-0.3573833	7.07641077	0.00286958	0.006927487
SPCC24B10.12	cgi121	EKC/KEOPS complex subunit Cgi121 (predicted)	-0.3574118	5.62110026	0.04296328	0.076136755
SPBC1A4.11c	SPBC1A4.11c	Schizosaccharomyces specific protein	-0.3574974	3.98513828	0.28490286	0.377853043
SPNCRNA.1045	#N/A	#N/A	-0.3575273	4.52289702	0.25447	0.344798307
SPNCRNA.1020	#N/A	#N/A	-0.3576263	3.91882474	0.31750211	0.411621619
SPNCRNA.120	#N/A	#N/A	-0.3580728	4.09892431	0.25819793	0.348739608
SPBC16E9.07	mug100	Schizosaccharomyces pombe specific protein Mug100	-0.3581728	5.50819418	0.08247694	0.133030478
SPNCRNA.851	#N/A	#N/A	-0.3582522	5.53066933	0.04015008	0.071768128
SPNCRNA.498	#N/A	#N/A	-0.3584	5.23647777	0.07343613	0.120330945
SPBC3E7.04c	SPBC3E7.04c	Ric8 family guanine nucleotide exchange factor, human syn	-0.3588961	5.33883416	0.18286387	0.260932817
ScpofMt33	#N/A	#N/A	-0.3588983	4.15293679	0.24305779	0.332263617
SPBC17D11.02c	hrd1	Hrd1 complex ubiquitin-protein ligase E3 subunit, Hrd1	-0.359336	7.38415517	0.00014946	0.000478835
SPBC725.05c	npp1	nucleotide pyrophosphatase Npp1 (predicted)	-0.3593751	7.08170576	0.00362438	0.008505028
SPBC16H5.13	wdr7	WD repeat protein, human WDR7 ortholog	-0.3594812	7.84013323	0.00041311	0.001214958
SPAC3H1.12c	snt2	Lid2 complex PHD finger subunit Snt2	-0.3598655	7.59059046	0.00025215	0.000775363
SPAC57A7.09	SPAC57A7.09	ubiquitin-protein ligase E3, human RNF13 family homolog,	-0.3602128	6.74592775	0.00717202	0.01564281
SPNCRNA.797	#N/A	#N/A	-0.3607664	6.16202902	0.01226302	0.025295497
SPBC609.03	iqw1	WD repeat protein, Iqw1	-0.3607734	7.64746592	0.00018986	0.000596773
SPNCRNA.1671	#N/A	#N/A	-0.3609546	4.94225915	0.12546688	0.190368372
SPNCRNA.271	#N/A	#N/A	-0.361583	2.09845583	0.62441913	0.705959821
SPBC215.01	SPBC215.01	GTPase activating protein (predicted)	-0.3617589	8.29203741	6.30E-05	0.000214878
SPAC1296.05c	lcp1	cyclin L family cyclin	-0.3617857	6.57236751	0.00569107	0.012768788
SPCC1450.16c	ptl1	triacylglycerol lipase ptl1	-0.3618739	6.72143176	0.01436495	0.029223768
SPNCRNA.767	#N/A	#N/A	-0.3619191	4.30143334	0.16984275	0.24503258
SPAC24H6.01c	gup1	membrane bound O-acyltransferase, MBOAT Gup1 (predict	-0.3623594	6.22211408	0.03196409	0.058803905
SPNCRNA.1106	#N/A	#N/A	-0.3624387	4.66522221	0.24130547	0.330133434
SPNCRNA.872	#N/A	#N/A	-0.362731	7.38787794	0.00027775	0.000845691
SPBC18H10.19	vps38	phophatidylinositol 3-kinase complex subunit Vps38	-0.3637601	6.10515065	0.02449744	0.046748484
SPNCRNA.501	#N/A	#N/A	-0.363844	4.59051762	0.16244378	0.236354483
SPAC18B11.05	gpi18	pig-V, dolichyl-phosphate-mannose-glycolipid alpha-manno	-0.3640435	6.87062955	0.00478208	0.010934832
SPBC4.03c	sfb3	COPII-coated vesicle component Sfb3 (predicted)	-0.3642564	4.76520576	0.25663179	0.346916831
SPBC8D2.15	lip5	mitochondrial lipoic acid synthetase Lip5 (predicted)	-0.3645598	7.11531599	0.00035511	0.001062203
SPAC13C5.06c	mug121	Schizosaccharomyces pombe specific protein Mug121	-0.3652227	6.64984589	0.00671051	0.014744923
SPBC1685.17	SPBC1685.17	Schizosaccharomyces pombe specific protein	-0.3661003	4.99977692	0.08676663	0.138994097

SPAC6F6.13c	SPAC6F6.13c	Golgi localized Alpha/Beta hydrolase fold, DUF726 family p	-0.3665772	8.14575788	0.00207405	0.005179421
SPAC25B8.13c	isp7	2-OG-Fe(II) oxygenase superfamily protein	-0.3666659	8.63186633	9.89E-07	4.57E-06
SPBC1604.16c	SPBC1604.16c	RNA-binding protein, G-patch type, human GPANK1 ortholo	-0.3671678	6.51610548	0.00347521	0.008219961
SPAC57A7.13	SPAC57A7.13	RNA-binding protein, involved in splicing (predicted)	-0.3672961	6.53601321	0.00410708	0.009522538
SPBC2F12.15c	pfa3	palmitoyltransferase Pfa3 (predicted)	-0.367513	6.52894047	0.0052922	0.011936829
SPBC1683.06c	urh1	uridine ribohydrolase Urh1 (predicted)	-0.3685081	7.24348779	0.00019135	0.000601156
SPBC24C6.10c	dip1	WISH/DIP/SPIN90 ortholog, endocytosis protein Dip1	-0.3687532	6.33027938	0.01339669	0.02740965
SPCC1682.03c	mug174	Schizosaccharomyces specific protein Mug174	-0.3688109	5.93949195	0.04076024	0.072686956
SPNCRNA.1667	#N/A	#N/A	-0.3692991	6.87383396	0.00110583	0.002939513
SPBC23E6.03c	nta1	protein N-terminal amidase Nta1 (predicted)	-0.3693396	7.94645136	1.81E-05	6.83E-05
SPAC14C4.07	SPAC14C4.07	transmembrane transporter (predicted)	-0.369851	8.41591874	1.09E-05	4.26E-05
SPAC4F8.03	sdo1	SBDS family ribosome assembly protein Sdo1 (predicted)	-0.3698892	6.50890638	0.00590857	0.013221898
SPNCRNA.1504	#N/A	#N/A	-0.3703244	5.59875433	0.08844976	0.141490511
SPAC31G5.12c	maf1	repressor of RNA polymerase III Maf1	-0.3704771	5.68589038	0.05861389	0.099220773
SPNCRNA.907	#N/A	#N/A	-0.3708998	3.77970592	0.3209637	0.415356293
SPBC146.05c	cwf25	complexed with Cdc5 protein Cwf25	-0.3710551	6.86925448	0.01615119	0.032392832
SPCC965.13	SPCC965.13	plasma membrane pyridoxal family transmembrane transp	-0.3710955	7.66769499	2.56E-05	9.45E-05
SPNCRNA.845	#N/A	#N/A	-0.3715499	2.02502067	0.58702848	0.673193258
SPBC31F10.11c	cwf4	Prp19 complex subunit Cwf4	-0.3716922	7.17784891	0.0011369	0.003009184
SPAC1039.09	isp5	amino acid transmembrane transporter Isp5	-0.3719841	9.56004671	2.50E-08	1.45E-07
SPNCRNA.846	#N/A	#N/A	-0.3720397	6.7097749	0.00175493	0.004457736
SPRRNA.50	SPRRNA.50	5.8S ribosomal RNA	-0.3724148	7.8586579	3.93E-05	0.000139086
SPBC1718.07c	zfs1	zf-CCCH tandem zinc finger protein, human Tristetraprolin h	-0.3724278	8.2170788	0.00029638	0.00089678
SPBC17D1.03c	rrp43	exosome subunit Rrp43	-0.3733356	6.14069174	0.01137402	0.023677026
SPBC902.05c	idh2	isocitrate dehydrogenase (NAD+) subunit 2	-0.3734592	8.51253969	3.33E-06	1.43E-05
SPAC30D11.05	aps3	AP-3 adaptor complex subunit Aps3 (predicted)	-0.3735651	7.16590138	0.00018678	0.000588175
SPNCRNA.1555	#N/A	#N/A	-0.3739696	3.96774938	0.19695303	0.277430217
SPCC330.19c	SPCC330.19c	Schizosaccharomyces pombe specific protein	-0.3744273	4.67117279	0.22567145	0.311373523
SPCC11E10.02c	gpi8	pig-K	-0.3744398	6.89445253	0.00324631	0.007734995
SPAC343.04c	gid7	GID complex subunit Gid7 (predicted)	-0.374478	7.73065204	0.00236036	0.005832335
SPCC63.13	SPCC63.13	DNAJ domain protein	-0.3745933	5.57406636	0.06090424	0.102410016
SPNCRNA.1641	#N/A	#N/A	-0.3746508	6.32035523	0.01935165	0.038137268
SPBC11G11.02c	end3	actin cortical patch component End3 (predicted)	-0.37504	7.78351728	0.00022392	0.000696101
SPAC140.02	gar2	nucleolar protein required for rRNA processing	-0.3752688	8.7915337	2.31E-06	1.02E-05
SPNCRNA.682	#N/A	#N/A	-0.3753857	5.21774214	0.06175783	0.103691596
SPNCRNA.87	#N/A	#N/A	-0.3762897	4.76620793	0.09679788	0.152443779
SPBC337.02c	SPBC337.02c	mug2/mug135/meu2 family	-0.3766686	7.1328411	0.00205452	0.005134422
SPAC17H9.06c	SPAC17H9.06c	DUF4196 and DUF4211, human CCDC82 ortholog	-0.3766699	7.96605769	5.78E-06	2.40E-05
SPBC713.09	SPBC713.09	Schizosaccharomyces specific protein	-0.3769828	7.32818782	0.00215301	0.005364796
SPCC1672.06c	asp1	inositol hexakisphosphate kinase/inositol pyrophosphate sy	-0.377072	8.54325476	2.28E-05	8.46E-05
SPNCRNA.380	#N/A	#N/A	-0.3772724	2.30100398	0.63867495	0.718445649
SPAC17C9.01c	nuc2	anaphase-promoting complex TPR lobe subcomplex subunit	-0.3773352	6.53868795	0.00177504	0.004498755
SPNCRNA.1243	#N/A	#N/A	-0.3775216	4.66535797	0.18424356	0.262357918
SPCC320.12	atp23	mitochondrial inner membrane peptidase Atp23 (predicted	-0.3779831	5.55499664	0.04652652	0.081328794
SPBC660.15	msi2	mRNA cleavage factor complex subunit Msi2	-0.3785972	8.1648118	3.77E-05	0.000133965
SPBC19G7.18c	SPBC19G7.18c	Schizosaccharomyces specific protein	-0.3786476	6.58424769	0.00384436	0.008962267
SPBC21C3.07c	trm140	tRNA (cytosine) methyltransferase Trm140 (predicted)	-0.3787299	6.81992851	0.0015482	0.003977169

SPNCRNA.1429	#N/A	#N/A	-0.3787341	4.49872111	0.12271757	0.186779935
SPBC3E7.13c	syf2	Prp19 complex subunit Syf2	-0.3787986	6.04171718	0.02005821	0.039307772
SPBC16G5.07c	SPBC16G5.07c	stomatin (predicted)	-0.3789843	6.72745471	0.00125292	0.003286851
SPNCRNA.666	#N/A	#N/A	-0.3793184	5.90781903	0.014435	0.029331202
SPNCRNA.1111	#N/A	#N/A	-0.3794548	3.96897007	0.26430099	0.355331135
SPNCRNA.1234	#N/A	#N/A	-0.3797127	6.15083885	0.00907122	0.019284494
SPNCRNA.823	#N/A	#N/A	-0.3798496	3.63531233	0.27259143	0.364293932
SPCC1183.09c	pmp31	plasma membrane proteolipid Pmp31	-0.380008	7.08856527	0.00167295	0.004267031
SPBC649.05	cut12	spindle pole body protein Cut12	-0.38024	6.46117003	0.00617401	0.01371667
SPBC20F10.02c	SPBC20F10.02c	DUF1741 family protein, human C10orf76 ortholog	-0.3802826	5.63340749	0.04236567	0.075234439
SPNCRNA.1115	#N/A	#N/A	-0.3803283	8.43830212	1.01E-06	4.66E-06
SPBC713.02c	ubp15	ubiquitin C-terminal hydrolase Ubp15	-0.3806619	9.70781888	8.37E-05	0.000279027
SPNCRNA.1113	#N/A	#N/A	-0.3806707	6.72027301	0.0057218	0.012833496
SPAC1486.09	nob1	ribosome biogenesis protein Nob1 (predicted)	-0.3811504	6.58829163	0.00261838	0.00637269
SPRRNA.51	SPRRNA.51	5.8S ribosomal RNA	-0.3812325	7.86352023	2.71E-05	9.92E-05
SPNCRNA.1177	#N/A	#N/A	-0.3812549	8.36599936	9.56E-06	3.79E-05
SPNCRNA.1636	#N/A	#N/A	-0.3813777	6.5557798	0.00345527	0.008187015
SPAC4D7.04c	rer2	cis-prenyltransferase	-0.3821072	6.64730328	0.00236394	0.00583693
SPBC23E6.02	rrp2	ATP-dependent DNA helicase/ ubiquitin-protein ligase E3 (p	-0.3822309	7.95491686	1.01E-05	3.99E-05
SPAC6F12.16c	mtr4	TRAMP complex ATP-dependent RNA helicase subunit Mtr4	-0.3829402	8.37548136	4.48E-06	1.89E-05
SPNCRNA.810	#N/A	#N/A	-0.3829871	5.0993905	0.14903346	0.219946365
SPNCRNA.160	#N/A	#N/A	-0.3830099	2.39085013	0.56105684	0.650811393
SPAC12B10.04	pby1	tubulin-tyrosine ligase Pby1 (predicted)	-0.3833236	6.43492034	0.00410677	0.009522538
SPAC959.06c	SPAC959.06c	alpha/beta hydrolase fold	-0.3835974	4.17133303	0.18157844	0.259261704
SPBC776.13	cnd1	condensin complex non-SMC subunit Cnd1	-0.3840032	7.78447633	0.00012887	0.000417216
SPAC31A2.06	atp25	mitochondrial polynucleotide adenylyltransferase Atp25 (pr	-0.3843697	8.05287926	3.87E-05	0.000137166
SPNCRNA.1337	#N/A	#N/A	-0.3844683	6.05042988	0.017932	0.035617995
SPAC13G6.12c	chs1	chitin synthase I	-0.3845662	6.22528476	0.00888889	0.018938292
SPAC9G1.07	SPAC9G1.07	Schizosaccharomyces specific protein	-0.3846428	7.06263483	0.00047575	0.001384734
SPAC2F7.09c	gep3	mitochondrial GTPase related protein Gep3 (prediected)	-0.3850445	6.07880048	0.01944069	0.038268411
SPBC26H8.10	dis3	exosome 3'-5' exoribonuclease subunit Dis3	-0.3867035	8.54053438	4.04E-06	1.72E-05
SPCC550.03c	ski2	Ski complex RNA helicase Ski2 (predicted)	-0.3869032	7.74411212	0.00134209	0.003491185
SPCC126.07c	asr1	ubiquitin-protein ligase E3 Asr1 (predicted)	-0.3871261	6.73467801	0.00109212	0.00290762
SPBC1271.01c	pof13	F-box protein Pof13	-0.3873147	6.04086794	0.00883766	0.018852741
SPBC1198.11c	reb1	RNA polymerase I transcription termination factor/ RNA po	-0.3881759	6.9802522	0.00037565	0.001115803
SPBC1703.09	SPBC1703.09	Schizosaccharomyces specific protein	-0.3886929	4.18028713	0.30079912	0.393717645
SPBC21.04	med8	mediator complex subunit Med8	-0.3887704	7.53494821	0.00034487	0.001032948
SPAC4H3.16	SPAC4H3.16	Schizosaccharomyces specific protein	-0.3893074	6.85864995	0.00092576	0.002505889
SPAC22F8.12c	shf1	small histone ubiquitination factor Shf1	-0.3907466	7.87088328	1.05E-05	4.15E-05
SPNCRNA.523	#N/A	#N/A	-0.3910127	3.20295691	0.36757741	0.462958412
SPBC3B9.14c	mrpl3	mitochondrial ribosomal protein subunit L3 (predicted)	-0.3910884	6.7059148	0.00140328	0.003635647
SPNCRNA.1316	#N/A	#N/A	-0.3911873	6.04878309	0.02324431	0.04470818
SPAP8A3.05	ski7	Ski complex interacting GTPase Ski7	-0.3913777	5.77776865	0.02054822	0.040073563
SPBC29A3.07c	sap14	U2 snRNP-associated protein SF3B14 Sap14	-0.3914194	5.35373021	0.03960286	0.070956777
SPCC594.07c	bqt3	bouquet formation protein Bqt3	-0.39171	6.98781833	0.00319	0.007619506
SPBC428.16c	rhb1	Rheb GTPase Rhb1	-0.3918284	6.84376154	0.00147421	0.003798554
SPBPB10D8.07c	SPBPB10D8.07c	transmembrane transporter (predicted)	-0.3923055	7.89068101	0.01855503	0.036769651

SPBC2A9.14	SPBC2A9.14	Schizosaccharomyces pombe specific protein	-0.392397	5.01486268	0.08051398	0.130172869
SPNCRNA.663	#N/A	#N/A	-0.3932103	5.23225273	0.13454522	0.201912939
SPBC23G7.06c	nvj2	nucleus-vacuole junction protein Nvj2	-0.3935054	6.5892879	0.00197725	0.00497051
SPAC644.07	bcs1	mitochondrial Rieske ISP assembly ATPase Bcs1 (predicted)	-0.39358	5.69581741	0.03245719	0.059598257
SPNCRNA.1215	#N/A	#N/A	-0.3935914	6.02908777	0.01409871	0.028716458
SPBC2G2.01c	liz1	plasma membrane pantothenate transmembrane transport	-0.3940096	6.46300548	0.00653673	0.014437668
SPAC7D4.12c	SPAC7D4.12c	plasma membrane ThrE amino acid transmembrane transp	-0.3943888	6.87942707	0.00433913	0.009999243
SPNCRNA.1535	#N/A	#N/A	-0.3943956	4.77851471	0.12301594	0.187192196
SPBC3B9.15c	scp1	Sre1 cleavage activating protein, Scap Scp1	-0.3944517	8.38192442	6.98E-06	2.85E-05
SPCC622.13c	tti1	ASTRA complex subunit, Armadillo-type fold TTi1	-0.3947211	8.48050617	4.74E-06	1.99E-05
SPAC29E6.08	tbp1	TATA-binding protein (TBP)	-0.3951178	8.492846	1.32E-06	6.01E-06
SPCC645.03c	isa1	mitochondrial [4Fe-4S] cluster asssembly and transfer prot	-0.3952116	6.93159239	0.00069929	0.001961857
SPAC3G9.13c	msw1	mitochondrial tryptophan-tRNA ligase Msw1 (predicted)	-0.3952295	7.84692951	8.36E-06	3.36E-05
SPNCRNA.27	#N/A	#N/A	-0.3960549	2.68387686	0.5127184	0.604373591
SPAC13D1.01c	Tf2-7	retrotransposable element/transposon Tf2-type	-0.3960806	6.51994733	0.00832645	0.017851766
SPAC26H5.08c	bgl2	glucan beta-glucosidase Bgl2 (predicted)	-0.3962125	7.37367669	0.00261174	0.006361085
SPCC736.02	SPCC736.02	Schizosaccharomyces specific protein	-0.3964227	5.15749644	0.06618455	0.1100112
SPBC2D10.13	est1	telomerase regulator Est1	-0.3964619	4.77849593	0.06953794	0.114660936
SPAC227.03c	yea6	mitochondrial carrier, NAD Yea6 (predicted)	-0.3967758	6.29447259	0.00798085	0.017181163
SPAC20H4.07	rad57	RecA family ATPase Rad57/Rhp57	-0.3972361	6.6435611	0.00705665	0.015420831
SPAC16E8.16	sua7	transcription factor TFIIB	-0.3974212	7.49639063	0.0001345	0.000434402
SPBC409.04c	mis12	NMS complex subunit Mis12	-0.3980108	6.92170851	0.00059211	0.001680274
SPAC4F8.07c	hxk2	hexokinase 2	-0.3980711	9.8421206	1.78E-08	1.05E-07
SPCP1E11.06	apl4	AP-1 adaptor complex gamma subunit Apl4	-0.3981047	8.52599531	6.90E-07	3.26E-06
SPBP16F5.03c	tra1	SAGA complex phosphatidylinositol pseudokinase Tra1	-0.3981157	9.65210294	6.87E-09	4.30E-08
SPBC1348.01	SPBC1348.01	S. pombe specific DUF999 protein family 5	-0.3985365	6.07013394	0.02019009	0.039514977
SPAC24H6.02c	tim15	TIM23 translocase complex subunit Tim15 (predicted)	-0.3987376	5.65003175	0.01914652	0.037798689
SPAC110.02	pds5	mitotic and meiotic cohesin loader subunit Pds5	-0.3999312	7.4348308	0.00084003	0.002299425
SPAC17A5.06	ptr8	transcription factor TFIIH complex DNA helicase (ERCC-3) su	-0.4000687	7.90935856	2.77E-05	0.000101097
SPBC1539.05	cog3	Golgi transport complex subunit Cog3 (predicted)	-0.4000979	6.24141523	0.00825699	0.017708412
SPBC29A10.03c	pcf1	CAF assembly factor (CAF-1) complex large subunit Pcf1	-0.4005503	7.06161826	0.00861701	0.018416694
SPCC1682.01	qcr9	ubiquinol-cytochrome-c reductase complex subunit 9 (predi	-0.4008861	7.11776875	7.09E-05	0.000239769
SPCC4E9.01c	rec11	meiotic cohesin complex subunit Rec11	-0.401091	5.16105362	0.10297246	0.160680486
SPAPB2B4.03	cig2	G1/S-specific B-type cyclin Cig2	-0.401513	8.37991772	3.23E-05	0.000116062
SPBCPT2R1.01c	SPBCPT2R1.01c	S. pombe specific DUF999 protein family 9	-0.4020204	6.06129955	0.01437776	0.029241072
SPAC1805.07c	dad2	DASH complex subunit Dad2	-0.402423	4.54496732	0.23143376	0.317970272
SPBP22H7.03	sbg1	plasma membrane-actinomyosin ring linker protein Sbg1	-0.403182	6.36703955	0.00678563	0.014885954
SPBC1685.13	fhn1	eisosome assembly protein Fhn1	-0.4036265	9.07988589	6.98E-07	3.29E-06
SPAC1250.01	snf21	RSC-type complex ATP-dependent DNA helicase Snf21	-0.403908	8.50298454	1.70E-06	7.62E-06
SPCC1919.11	mug137	BAR adaptor protein, involved in endocytosis (predicted)	-0.4042042	6.93133533	0.00187906	0.004741196
SPAC22F8.11	plc1	phosphoinositide phospholipase C Plc1	-0.404231	8.04613979	4.41E-06	1.86E-05
SPAC3H1.04c	mdm31	mitochondrial inner membrane protein Mdm31	-0.404426	7.60289679	3.60E-05	0.000128307
SPAC12B10.13	gid8	GID complex subunit Gid8 (predicted)	-0.4045596	6.32053525	0.00815729	0.017522213
SPAC31A2.05c	mis4	cohesin loading factor (adherin) Mis4	-0.4049443	7.15965911	0.00172241	0.004386603
SPBC11B10.06	sws1	Srs2 interacting SWIM domain protein Srs1	-0.4049787	5.6955832	0.02258594	0.043577355
SPNCRNA.216	#N/A	#N/A	-0.4050654	4.96569297	0.11586477	0.177821323
SPNCRNA.1189	#N/A	#N/A	-0.405467	7.48036677	1.15E-05	4.50E-05

SPAC1D4.12	rad15	transcription factor TFIIH complex DNA helicase subunit Ra	-0.4061781	7.66273244	5.16E-06	2.15E-05
SPNCRNA.1386	#N/A	#N/A	-0.4073972	7.21928469	0.00048327	0.001405409
SPCC1183.05c	lig4	DNA repair ligase Lig4	-0.4076651	7.5688829	0.00068526	0.001925335
SPAC167.05	dbs2	Usp (universal stress protein) family protein	-0.4080661	8.09892785	4.10E-05	0.000144761
SPAC1F5.09c	shk2	PAK-related kinase Shk2	-0.4086532	5.23468508	0.02792218	0.052315642
SPNCRNA.558	#N/A	#N/A	-0.4088739	4.40874311	0.1155143	0.177323436
SPBC12C2.02c	ste20	Rictor homolog, Ste20	-0.4091033	8.39017132	5.08E-05	0.000176768
SPAC140.04	ctr1	telomerase regulatory factor Ctr1	-0.4097108	5.70866229	0.01617298	0.032417423
SPBC365.09c	kin17	human KIN ortholog (predicted)	-0.4097856	6.27554299	0.0027568	0.006683303
SPBC27B12.01c	mmm1	ERMES complex subunit Mmm1 (predicted)	-0.4098594	6.7754747	0.00203207	0.005087652
SPAC29E6.02	prp3	U4/U6 x U5 tri-snRNP complex subunit Prp3	-0.409985	9.00228633	3.49E-07	1.71E-06
SPBC21.05c	ral2	Ras1-Scd pathway protein Ral2	-0.4101368	7.48363809	0.00014893	0.000477591
SPAC2E1P5.01c	mns1	mannosyl-oligosaccharide 1,2-alpha-mannosidase	-0.410468	6.59918104	0.00576023	0.012906931
SPNCRNA.1140	#N/A	#N/A	-0.4104723	6.14923308	0.03480394	0.063428167
SPAPB1A10.07c	SPAPB1A10.07c	sphingolipid biosynthesis protein (predicted)	-0.4105236	6.00014422	0.0085068	0.01820847
SPNCRNA.619	#N/A	#N/A	-0.4108353	6.12721808	0.00701142	0.015341715
SPNCRNA.1095	#N/A	#N/A	-0.4119563	8.22905382	1.13E-05	4.41E-05
SPBC6B1.03c	SPBC6B1.03c	Pal1 family protein, implicated in cell wall organization	-0.41235	4.05177773	0.27325853	0.364850695
SPAC2C4.07c	dis32	3'-5'-exoribonuclease activity Dis3L2	-0.4127378	6.8609907	0.0007391	0.002059616
SPBC25H2.18	cox20	cytochrome c oxidase assembly protein Cox20 (predicted)	-0.4128784	5.73958764	0.0274506	0.051602569
SPNCRNA.975	#N/A	#N/A	-0.4130211	6.36967183	0.00549554	0.012351382
SPAPB2B4.04c	pmc1	vacuolar calcium transporting P-type ATPase P2 type, Pmc1	-0.413173	9.38858779	4.63E-07	2.24E-06
SPCC645.11c	mug117	conserved fungal protein Mug117	-0.4132516	5.47707946	0.01663578	0.033253127
SPBC4B4.05	smg1	Sm snRNP core protein Smg1	-0.4135993	5.82742782	0.01614999	0.032392832
SPNCRNA.1543	#N/A	#N/A	-0.4138415	6.93546218	0.00023761	0.000734982
SPNCRNA.634	#N/A	#N/A	-0.4139771	5.38329443	0.01917797	0.037849784
SPNCRNA.794	#N/A	#N/A	-0.4150045	4.9123082	0.09270983	0.147369871
SPAP4C9.02	emc5	ER membrane protein complex subunit Emc5 (predicted)	-0.4150758	5.0533417	0.07115014	0.116922659
SPCC162.06c	vps60	vacuolar sorting protein Vps60 (predicted)	-0.4157571	6.33059543	0.00254059	0.006214486
SPNCRNA.1027	#N/A	#N/A	-0.415878	7.51246025	0.00010283	0.000338456
SPBC26H8.03	cho2	phosphatidylethanolamine N-methyltransferase Cho2	-0.4164157	8.68853943	3.23E-07	1.59E-06
SPBC18H10.06c	swd2	Set1C complex subunit Swd2.1	-0.4167573	5.20136531	0.03768836	0.067884944
SPAC4D7.07c	csi2	mitotic chromosome segregation protein Csi2	-0.417252	6.54780452	0.02011242	0.039385609
SPCC18.19c	ost5	oligosaccharyltransferase complex zeta subunit Ost5 (predi	-0.4176054	3.25306071	0.32007875	0.414488282
SPAC4D7.13	usp104	U1 snRNP-associated protein Usp104	-0.4176992	8.07595296	3.08E-07	1.53E-06
SPNCRNA.1244	#N/A	#N/A	-0.4178246	2.6342297	0.48418752	0.579373317
SPNCRNA.836	#N/A	#N/A	-0.4180182	8.95310668	1.06E-05	4.17E-05
SPNCRNA.984	#N/A	#N/A	-0.4180267	4.68841355	0.20000633	0.280594095
SPCC364.05	vps3	CORVET complex subunit, GTPase regulator Vps3 (predicte	-0.41804	6.7341738	0.00077088	0.002131866
SPAC9G1.14	#N/A	#N/A	-0.418617	4.10929311	0.16231433	0.236267171
SPBCPT2R1.04c	SPBCPT2R1.04c	S. pombe specific DUF999 protein family 10	-0.4187774	6.20226244	0.01398844	0.02851748
SPBP19A11.07c	SPBP19A11.07c	human HID1 ortholog 2, possible Golgi protein (by similarity	-0.4188796	6.45733817	0.00803054	0.017282684
SPAC688.09	rim2	mitochondrial carrier, pyrimidine nucleotide Rim2 (predicte	-0.4189429	6.90690305	0.00280759	0.006791921
SPCC1020.13c	SPCC1020.13c	DDHD family phospholipase (predicted)	-0.419193	7.82842546	7.22E-05	0.000243912
SPAC3A12.02	SPAC3A12.02	mitochondrial inorganic diphosphatase (predicted)	-0.4197192	5.62181129	0.02257842	0.043575196
SPCC622.09	htb1	histone H2B Htb1	-0.4197451	9.23586338	2.03E-07	1.02E-06
SPBC8D2.02c	vps68	vacuolar sorting protein Vps68 (predicted)	-0.4200735	6.60193188	0.0316397	0.058372223

SPBC23E6.08	sat1	Golgi membrane exchange factor subunit Sat1 (predicted)	-0.4205387	6.83243487	0.02035536	0.039758391
SPBC1348.05	SPBC1348.05	transmembrane transporter (predicted)	-0.4206844	5.57300507	0.02746004	0.051606061
SPBC20F10.03	SPBC20F10.03	Armadillo-type fold protein, human IFRD1 ortholog, implica	-0.4207092	5.95597445	0.01258682	0.025900617
SPNCRNA.1169	#N/A	#N/A	-0.4212239	5.62574121	0.01920118	0.037884611
SPAC29B12.12	hot13	helper of TIM Hot13 (predicted)	-0.4215469	4.00759578	0.17684335	0.253511474
SPAC1565.04c	ste4	adaptor protein Ste4	-0.4219983	6.62474322	0.00173393	0.004410988
SPBC3E7.09	SPBC3E7.09	Sad1-UNC-like protein involved protein folding in the ER (pr	-0.4222914	7.29856361	2.93E-05	0.00010624
SPNCRNA.1082	#N/A	#N/A	-0.4224432	8.69784366	8.63E-06	3.45E-05
SPBC146.03c	cut3	condensin complex SMC subunit Smc4	-0.4228521	7.98381195	1.86E-06	8.30E-06
SPAP19A11.05c	mrp7	mitochondrial ribosomal protein subunit L27 (predicted)	-0.4232367	5.94395696	0.01357596	0.027734744
SPCC16C4.22	dpb3	DNA polymerase epsilon Dpb3	-0.423421	4.95407434	0.05547626	0.094758013
SPAC1565.03	SPAC1565.03	Schizosaccharomyces pombe specific protein	-0.4240686	4.21180201	0.14485331	0.214615009
SPAC25H1.03	atg101	autophagy associated HORMA domain protein Atg101	-0.4241865	5.43701902	0.0188408	0.037292517
SPCP1E11.08	nsa2	ribosome biogenesis protein Nsa2 (predicted)	-0.4243985	7.36738471	1.18E-05	4.62E-05
SPNCRNA.504	#N/A	#N/A	-0.4244795	3.49008252	0.27104494	0.362696125
SPBC24C6.02	spb4	ATP-dependent RNA helicase Spb4 (predicted)	-0.4251967	6.71117853	0.00100229	0.002686334
SPAC25B8.07c	rcf1	cytochrome c oxidase assembly protein Rcf1	-0.4258237	5.86856241	0.02945621	0.054872573
SPAPB17E12.06	sos7	NMS complex subunit Sos7	-0.4258562	6.03207264	0.00492919	0.011218447
SPBC577.14c	spa1	ornithine decarboxylase antizyme with +1 programmed ribo	-0.4261014	7.01157112	5.11E-05	0.000177413
SPBC36B7.06c	mug20	Schizosaccharomyces specific protein Mug20	-0.4262585	3.74252625	0.33215777	0.426558521
SPCC16A11.12c	ubp1	ubiquitin C-terminal hydrolase Ubp1	-0.4263863	7.08983198	0.00010144	0.000334279
SPAC24H6.12c	uba3	NEDD8 activating enzyme E1-type Uba3	-0.4264144	6.74788556	0.00097386	0.002621486
SPBC365.15	alp4	gamma tubulin complex Spc97/GCP2 subunit Alp4	-0.4265358	8.15540631	4.13E-05	0.000145661
SPAC6B12.18	gon7	EKC/KEOPS complex subunit Gon7 (predicted)	-0.4273005	5.76314788	0.00709065	0.015475258
SPNCRNA.1653	#N/A	#N/A	-0.4278408	6.63707971	0.00144476	0.003733995
SPCC4B3.04c	nte1	lysophospholipase (predicted)	-0.4279965	8.40120968	5.12E-07	2.46E-06
SPCC330.11	btb1	BTB/POZ domain protein Btb1	-0.4280433	7.98243991	9.70E-07	4.49E-06
SPNCRNA.564	#N/A	#N/A	-0.4289907	6.68486072	0.00025732	0.00078983
SPAC1952.06c	SPAC1952.06c	spliceosomal complex subunit (predicted)	-0.4292929	6.5719908	0.00132375	0.003458015
SPAC1486.03c	ntr1	RNA-binding splicing factor Ntr1 (predicted)	-0.4294546	7.49703806	6.73E-05	0.000228641
SPAC12B10.05	icp55	mitochondrial intermediate cleavage peptidase Icp55 (pred	-0.429744	6.92795211	0.00024589	0.000758858
SPNCRNA.1001	#N/A	#N/A	-0.4300152	6.46424997	0.00109413	0.002911818
SPNCRNA.1528	#N/A	#N/A	-0.4300153	4.7989492	0.07275547	0.119387746
SPCC794.01c	gcd1	glucose dehydrogenase Gcd1	-0.4304779	6.20997507	0.00488814	0.011147386
SPNCRNA.735	#N/A	#N/A	-0.4309812	7.00267366	0.00039562	0.001169503
SPAC222.16c	csn3	COP9/signalosome complex subunit Csn3 (predicted)	-0.431026	5.60027004	0.02359512	0.045267776
SPNCRNA.1453	#N/A	#N/A	-0.4311789	5.59302791	0.10232568	0.159896222
SPAC13G6.08	fzr2	meiotic fizzy-related APC coactivator Fzr2 (predicted)	-0.4312371	5.78791004	0.01521166	0.03071574
SPAC212.04c	SPAC212.04c	S. pombe specific DUF999 family protein 1	-0.4317979	5.56384367	0.02808315	0.052530463
SPNCRNA.1367	#N/A	#N/A	-0.4319451	6.07279377	0.03890126	0.069810935
SPAC17G8.11c	imt3	mannosyltransferase Imt3	-0.4322858	7.15733728	0.00294729	0.007082034
SPCC1919.01	ckk2	calmodulin-dependent kinase kinase 2	-0.4323799	6.5181146	0.00085896	0.002342772
SPBC31F10.04c	srb4	mediator complex subunit Med17	-0.4324322	6.7593735	0.00078824	0.00217165
SPAPYUK71.03c	tcb3	tricalbin, C2 domain protein (phospholipid binding) ER-plasi	-0.4327324	9.48430037	6.93E-09	4.33E-08
SPCC1919.05	ski3	Ski complex TPR repeat subunit Ski3 (predicted)	-0.4331426	8.45133239	1.61E-07	8.24E-07
SPCC895.08c	SPCC895.08c	Schizosaccharomyces specific protein	-0.4337201	6.63416328	0.00245488	0.006033038
SPAC16E8.05c	SPAC16E8.05c	Schizosaccharomyces specific protein Mde1	-0.4337284	4.58420133	0.09651291	0.152065368

SPNCRNA.914	#N/A	#N/A	-0.4337284	4.58420133	0.09651291	0.152065368
SPAC6F6.04c	SPAC6F6.04c	transmembrane transporter (predicted)	-0.4337424	6.06899114	0.00349848	0.008263492
SPBC21C3.14c	SPBC21C3.14c	chromatin binding protein ortholog	-0.4343335	8.65239679	6.61E-08	3.61E-07
SPBC15C4.02	SPBC15C4.02	ABC1 kinase family protein, implicted in mitochondrial ergc	-0.4347467	7.08210733	2.85E-05	0.000103609
SPBC3D6.03c	trz2	mitochondrial 3'-tRNA processing endonuclease tRNAse Z,	-0.4348183	6.54896332	0.00070376	0.001971626
SPBC1347.09	SPBC1347.09	hexaprenyldihydroxybenzoate methyltransferase, Coq3 varia	-0.4350765	7.09685461	0.00010286	0.000338456
SPCC74.03c	ssp2	AMP-activated protein serine/threonine kinase alpha subun	-0.4351877	8.47124908	2.18E-08	1.27E-07
SPAC22A12.11	dak1	dihydroxyacetone kinase Dak1	-0.4353412	9.15960969	1.02E-05	4.01E-05
SPAC23C11.08	php3	CCAAT-binding factor complex subunit Php3	-0.4354624	5.31103093	0.0655142	0.109083467
SPNCRNA.1645	#N/A	#N/A	-0.4356235	6.66450167	0.02048292	0.03996166
SPBPB2B2.16c	SPBPB2B2.16c	transmembrane transporter (predicted)	-0.4359795	5.55919003	0.01657535	0.03314582
SPBPB21E7.04c	SPBPB21E7.04c	O-methyltransferase, human COMT catechol homolog 2	-0.436462	8.954824	1.03E-08	6.27E-08
SPAC3F10.08c	faf1	rRNA processing protein Faf1 (predicted)	-0.4366591	5.97806352	0.00662242	0.01459376
ScpofMt16	#N/A	#N/A	-0.4370742	2.20384326	0.51472137	0.606314507
SPNCRNA.1130	#N/A	#N/A	-0.4374113	3.1517049	0.29653776	0.389862733
SPAPB1E7.11c	mpa1	mitochondrial translational activator, Mpa1	-0.4377592	6.83897375	0.00059194	0.001680274
SPAC23H4.11c	cnl2	centromere localized protein Cnl2	-0.4379956	5.49180219	0.01190603	0.024636733
SPAC17G8.13c	mst2	histone acetyltransferase Mst2	-0.4381743	8.55824087	2.68E-07	1.34E-06
SPNCRNA.105	#N/A	#N/A	-0.4384672	3.39351343	0.3561512	0.451660254
SPNCRNA.676	#N/A	#N/A	-0.4385895	4.94435953	0.04797017	0.083509087
SPCC663.08c	SPCC663.08c	short chain dehydrogenase, unknown specificity (predicted)	-0.438957	5.62806098	0.02799777	0.052399562
SPBC365.05c	slu7	splicing factor Slu7	-0.4391017	6.56060619	0.00074676	0.002077352
SPAC3G9.12	peg1	CLASP family microtubule-associated protein	-0.43933	7.6010329	8.36E-06	3.36E-05
SPBC409.14c	mrps17	mitochondrial ribosomal protein subunit S17 (predicted)	-0.4401599	6.23983666	0.00401601	0.009327282
SPBC3B9.02c	cwf28	splicing factor Cwf28	-0.440749	6.63414864	0.00123585	0.003247097
SPNCRNA.1238	#N/A	#N/A	-0.44116	4.98916442	0.04635528	0.081091956
SPCC1259.11c	gyp2	GTPase activating protein Gyp2 (predicted)	-0.4415565	7.81470712	1.57E-06	7.07E-06
SPAC6F12.15c	cut9	anaphase-promoting complex, TPR lobe subcomplex subuni	-0.4415667	7.1825889	0.00021346	0.000666323
SPAC20H4.09	SPAC20H4.09	ATP-dependent RNA helicase, spliceosomal (predicted)	-0.4419374	6.67049931	0.0004846	0.001408664
SPAC1002.08c	mtf1	mitochondrial RNA polymerase specificity factor, predicted	-0.4419944	6.28607554	0.00630367	0.013963688
SPCC1739.15	wtf21	wtf element Wtf21	-0.442022	5.54783804	0.02396952	0.04585678
SPBC1289.02c	uap2	U2 snRNP-associated protein Uap2	-0.4422445	7.0408384	0.00321561	0.007672586
SPAC17G8.12	sec3	exocyst complex subunit Sec3	-0.4426388	8.5598875	1.84E-07	9.35E-07
SPCC162.08c	nup211	nucleoporin nup211	-0.4426681	8.31729117	1.62E-07	8.28E-07
SPBC3E7.08c	rad13	DNA repair nuclease Rad13	-0.4433347	7.51290875	0.000314	0.000948808
SPBP4H10.19c	SPBP4H10.19c	calreticulin/calnexin homolog (predicted)	-0.4438085	5.78579391	0.01085082	0.02268504
SPAC15A10.01	atm1	mitochondrial iron-sulfur cluster exporter Atm1	-0.4445153	6.78191231	0.00158274	0.004055181
SPAC11D3.16c	SPAC11D3.16c	Schizosaccharomyces specific protein	-0.4447268	6.44179087	0.00531126	0.011967931
SPAPJ691.02	SPAPJ691.02	yippee-like protein	-0.4448014	6.10014613	0.01223498	0.025245314
SPBC2D10.05	exg3	cell wall glucan 1,3-beta-glucosidase Exg3	-0.4454296	7.32661131	0.00597065	0.013338884
SPNCRNA.818	#N/A	#N/A	-0.4458597	6.14438931	0.00278901	0.006749355
SPBC30B4.08	eri1	double-strand siRNA ribonuclease Eri1	-0.4460574	6.85036392	0.00036957	0.001099167
SPNCRNA.246	#N/A	#N/A	-0.4461667	4.67648728	0.16391841	0.238245365
SPCC1840.09	coq11	ubiquinone biosynthesis protein Coq11	-0.4464292	6.42892758	0.00256046	0.006249626
SPCC970.07c	raf2	CLRC ubiquitin ligase complex subunit Raf2	-0.4470109	6.2232122	0.00341747	0.008103094
SPBC3B8.10c	nem1	Nem1-Spo7 phosphatase complex catalytic subunit Nem1 (	-0.4471292	6.73525541	0.00088537	0.002407093
SPBC1734.02c	cdc27	DNA polymerase delta subunit Cdc27	-0.447135	5.50934963	0.01259785	0.025915467

SPNCRNA.1570	#N/A	#N/A	-0.4473646	7.53341641	6.05E-05	0.000207184
SPAC1805.03c	trm13	tRNA 2'-O-methyltransferase Trm13 (predicted)	-0.4477633	5.24721439	0.01518201	0.030665955
SPBC119.13c	prp31	U4/U6 x U5 tri-snRNP complex subunit Prp31	-0.4481567	6.88851675	0.00172971	0.004401892
SPNCRNA.1591	#N/A	#N/A	-0.4483367	6.32501953	0.0105643	0.022147281
SPNCRNA.624	#N/A	#N/A	-0.4493876	5.55845059	0.00935031	0.019834422
SPNCRNA.985	#N/A	#N/A	-0.4495116	4.66444411	0.15559976	0.22805435
SPCC1281.08	wtf11	wtf element Wtf11	-0.4498693	7.82641124	3.45E-06	1.48E-05
SPBC56F2.15	tam13	Schizosaccharomyces specific protein Tam13	-0.4502925	7.02037056	0.00184368	0.004655368
SPNCRNA.1637	#N/A	#N/A	-0.4503143	6.41439194	0.00163516	0.004173758
SPCC18.04	pof6	F-box protein Pof6	-0.450411	7.21446944	1.71E-05	6.51E-05
SPAC688.12c	SPAC688.12c	Schizosaccharomyces specific protein	-0.4504449	6.46223221	0.00033993	0.001019942
SPBC21B10.07	SPBC21B10.07	glycosyl hydrolase family (predicted)	-0.4510802	7.77277865	1.58E-07	8.11E-07
SPNCRNA.1638	#N/A	#N/A	-0.4526471	6.52108788	0.00102995	0.00275395
SPBC8D2.03c	hhf2	histone H4 h4.2	-0.4526548	7.53029793	1.49E-05	5.73E-05
SPAC11E3.14	SPAC11E3.14	protein with a role in clearing protein aggregates (predicte	-0.4529441	8.05527484	8.32E-06	3.35E-05
SPAC25G10.07c	cut7	kinesin-5 family plus-end directed microtubule motor, bim(	-0.453024	7.48995804	3.21E-06	1.38E-05
SPBC2D10.18	abc1	ABC1 kinase family ubiquinone biosynthesis ATPase Abc1/C	-0.453112	7.45439724	7.72E-05	0.000259251
SPBC29A10.02	spo5	meiotic RNA-binding protein 1	-0.4533049	4.43820039	0.13295497	0.200079289
SPNCRNA.1197	#N/A	#N/A	-0.4533907	6.60738541	0.00051663	0.001491061
SPNCRNA.1195	#N/A	#N/A	-0.4539195	8.21769497	7.12E-07	3.35E-06
SPBC15D4.07c	atg9	autophagy associated protein Atg9	-0.4539424	6.85132036	4.65E-05	0.000162677
SPBC26H8.05c	ppe2	serine/threonine protein phosphatase, PP4 complex subunit	-0.4545617	6.26074916	0.00123183	0.003237779
SPBC1652.01	stb3	transcription coregulator Stb3 (predicted)	-0.4549057	7.52232322	2.47E-06	1.08E-05
SPBC1685.12c	#N/A	#N/A	-0.4550809	6.57716529	0.00815517	0.017522213
SPAC7D4.03c	ahk1	scaffold protein, MAP kinase signalling pathway, Ahk1 (pre(	-0.4551298	8.55336295	1.82E-09	1.23E-08
SPCC663.03	pmd1	leptomycin transmembrane transporter Pmd1	-0.4554942	9.43455207	1.38E-09	9.47E-09
SPCC613.09	sen54	tRNA-splicing endonuclease subunit Sen54 (predicted)	-0.455749	6.70667433	0.0004509	0.001316897
SPAC16E8.02	mpo1	ER membrane sphingoid metabolism protein Mpo1 (predict	-0.4562301	4.08044993	0.16917788	0.244362879
SPNCRNA.1566	#N/A	#N/A	-0.4563027	5.59781982	0.0316572	0.058381374
SPCC1020.10	oca2	serine/threonine protein kinase Oca2	-0.4567905	7.45670607	5.41E-05	0.000186848
SPNCRNA.125	#N/A	#N/A	-0.457253	3.04345937	0.32873357	0.423439706
SPAC27E2.07	pvg2	Golgi 4,6-pyruvylated galactose (PvGal) residue biosynthesi	-0.4573559	6.96991143	2.68E-05	9.84E-05
SPAC17G6.05c	bro1	BRO1 domain protein Bro1 (predicted)	-0.4581393	7.43154559	2.82E-05	0.000102827
SPNCRNA.554	#N/A	#N/A	-0.4583489	5.0917055	0.03318654	0.060855407
SPBC1709.09	rrf1	mitochondrial translation termination factor Rrf1	-0.4585048	5.23370446	0.021239	0.041274135
SPAC750.02c	SPAC750.02c	transmembrane transporter (predicted)	-0.458613	5.57883312	0.0103191	0.021659921
SPAC16C9.07	pom2	DYRK family protein kinase Pom2	-0.4588364	7.09106172	4.50E-05	0.000157659
SPCC16C4.16c	cbc3	nuclear cap-binding complex variant subunit Cbc3	-0.4593361	6.42006021	0.00056084	0.001600217
SPCC4F11.02	ptc1	MAP kinase threonine phosphatase Ptc1	-0.4594658	7.72510234	2.47E-05	9.13E-05
SPCC1393.05	ers1	RNA-silencing factor Ers1	-0.4601646	6.04316606	0.002756	0.006683303
SPAC6C3.04	cit1	citrate synthase Cit1	-0.4604761	9.1058149	3.04E-10	2.26E-09
SPBPB2B2.19c	SPBPB2B2.19c	S. pombe specific 5Tm protein family	-0.4626047	8.0785646	8.80E-07	4.09E-06
SPBC21C3.05	sap62	U2 snRNP Sap62	-0.4632473	6.14184488	0.00229054	0.005680429
SPAC24H6.06	sld3	DNA replication pre-initiation complex subunit Sld3	-0.4635724	6.07817593	0.00407203	0.009454163
SPAC6B12.09	trm10	tRNA m(1)G methyltransferase Trm10	-0.4638911	6.43573961	0.00058887	0.001672475
SPCC895.09c	ucp12	ATP-dependent RNA helicase Ucp12, unknown location and	-0.4645188	7.62723825	5.25E-05	0.000181877
SPBC17D1.07c	npg1	GTPase regulator Rng2-like (predicted)	-0.4651179	6.48871879	0.00414669	0.009611116

SPBC30D10.08	mgm101	mitochondrial DNA repair protein (predicted)	-0.4651309	6.45747516	0.00055923	0.001596293
SPAC23A1.15c	sec20	SNARE Sec20 (predicted)	-0.4654668	6.21604902	0.00111322	0.002955705
SPBC18H10.09	SPBC18H10.09	zf-CHY type zinc finger protein	-0.4655559	6.52236209	0.00072264	0.002017879
SPCC24B10.11c	tho7	THO complex subunit Tho7 (predicted)	-0.4657215	6.66565583	0.00265838	0.006460819
SPBC29A10.04	psm1	mitotic cohesin complex ATPase subunit Psm1/Smc1	-0.4657471	7.98611348	3.07E-06	1.33E-05
SPBC947.09	hsp3103	ThiJ domain protein, implicated in cellular detoxification	-0.4660077	5.80735359	0.0034743	0.008219961
SPCC737.09c	hmt1	vacuolar phytochelatin and glutathione S-conjugate ABC far	-0.4660688	8.67221427	9.61E-07	4.45E-06
SPBC776.10c	cog6	Golgi transport complex peripheral subunit Cog6 (predicted	-0.4662803	7.66520381	1.13E-05	4.42E-05
SPNCRNA.557	#N/A	#N/A	-0.4669233	5.58264974	0.01169232	0.024250608
SPNCRNA.26	#N/A	#N/A	-0.4674359	3.97833558	0.20703534	0.289059397
SPAC144.15c	cog1	Golgi transport complex subunit Cog1 (predicted)	-0.4675913	8.16920152	1.15E-07	6.03E-07
SPCC63.11	prp28	U5 snRNP-associated ATP-dependent RNA helicase Prp28 (|	-0.4677414	7.53386435	5.85E-05	0.000200991
SPBC691.03c	apl3	AP-2 adaptor complex alpha subunit Alp3	-0.4680584	8.19960983	1.12E-07	5.86E-07
SPAC19D5.09c	Tf2-8	retrotransposable element/transposon Tf2-type	-0.468113	6.96189424	0.0002202	0.000685805
SPBC19F8.03c	yap18	ENTH/VHS domain protein (predicted)	-0.4684117	8.25453236	1.55E-08	9.21E-08
SPCC569.02c	SPCC569.02c	S. pombe specific UPF0321 family protein 2	-0.4685405	3.9276763	0.12845001	0.194158661
SPNCRNA.1025	#N/A	#N/A	-0.469501	5.45574756	0.01531071	0.030880081
SPRRNA.52	SPRRNA.52	5.8S ribosomal RNA	-0.469678	7.9444663	6.12E-08	3.35E-07
SPCC1393.11	mrpl20	mitochondrial ribosomal protein subunit L20, Mrpl20 (predi	-0.469841	6.99077309	8.29E-06	3.33E-05
SPNCRNA.573	#N/A	#N/A	-0.47026	7.13056455	0.00355919	0.008383627
SPBC337.11	SPBC337.11	mitochondrial inner membrane CH-OH group oxidoreductas	-0.4707167	5.32359887	0.01407405	0.028674822
SPAC688.10	rev3	DNA polymerase zeta catalytic subunit Rev3 (predicted)	-0.4714178	6.98788028	1.56E-05	5.96E-05
SPAC227.15	reg1	protein phosphatase regulatory subunit Reg1 (predicted)	-0.4714251	7.77584762	3.62E-07	1.77E-06
SPAC694.06c	mrc1	claspin, Mrc1	-0.4715107	7.47577324	0.00018112	0.000571147
SPBC1348.02	ftm5	sub-telomeric 5Tm protein family Ftm5	-0.4719281	8.13783062	9.04E-07	4.19E-06
SPAC4A8.05c	myp2	myosin II heavy chain Myo3	-0.4723624	9.33899419	1.38E-07	7.13E-07
SPAC1B2.06	SPAC1B2.06	Schizosaccharomyces pombe specific protein	-0.4730656	2.42195393	0.42567327	0.52070944
SPBC317.01	mbx2	MADS-box transcription factor Pvg4	-0.4734835	8.35068386	1.27E-06	5.79E-06
SPBC3D6.11c	slx8	SUMO-targeted ubiquitin-protein ligase E3 Slx8	-0.4735435	7.01748433	0.00010432	0.000342618
SPNCRNA.972	#N/A	#N/A	-0.4735924	7.47490585	0.00015934	0.000507639
SPAC23A1.06c	cmk2	MAPK-activated protein kinase Cmk2	-0.473613	6.61288748	0.00024874	0.000766268
SPAPYUG7.02c	sin1	stress activated MAP kinase interacting protein Sin1	-0.4736513	7.32609752	6.47E-06	2.66E-05
SPAC1952.15c	rec24	meiotic recombination protein Rec24	-0.4736948	5.43939473	0.01031609	0.021659921
SPNCRNA.218	#N/A	#N/A	-0.4740936	3.47719175	0.21532947	0.299460686
SPBC428.20c	alp6	gamma tubulin complex Spc98/GCP3 subunit Alp6	-0.4742426	6.58849504	0.00026446	0.000809924
SPNCRNA.266	#N/A	#N/A	-0.4747695	1.16964603	0.77294204	0.831600087
SPAC328.02	dbl4	ubiquitin-protein ligase E3 involved in sporulation Dbl4	-0.4748693	6.94831804	5.22E-05	0.000180694
SPBC1778.09	SPBC1778.09	GTPase activating protein (predicted)	-0.4753629	6.07503013	0.00759939	0.016469387
SPAC589.12	cwh43	glycosylceramide biosynthesis protein Cwh43 (predicted)	-0.4756123	7.53977372	2.31E-05	8.58E-05
SPNCRNA.1297	#N/A	#N/A	-0.4759405	4.51963872	0.05311851	0.091280675
SPAC644.06c	cdr1	NIM1 family serine/threonine protein kinase Cdr1/Nim1	-0.4765167	7.83253469	2.97E-05	0.00010763
SPAC1002.19	urg1	GTP cyclohydrolase II Urg1 (predicted)	-0.4770814	6.44877592	0.00078446	0.002162982
SPBC36B7.05c	pib1	endosomal and vacuolar ubiquitin-protein ligase E3/phosph	-0.4771772	5.6854095	0.00543151	0.012226728
SPNCRNA.1384	#N/A	#N/A	-0.477265	4.03582031	0.12067142	0.184325262
SPBC409.03	swi5	Swi5 protein	-0.4775521	6.46283476	0.00073067	0.002038632
SPBC18E5.09c	SPBC18E5.09c	Schizosaccharomyces specific protein	-0.477613	5.11250913	0.03998692	0.071514057
SPCC162.10	ppk33	serine/threonine protein kinase Ppk33 (predicted)	-0.4789482	6.54826708	0.00076605	0.002120812

SPAC18G6.10	lem2	LEM domain nuclear inner membrane protein Heh1/Lem2	-0.4794154	6.95888195	9.39E-06	3.73E-05
SPCC970.12	mis18	kinetochore protein Mis18	-0.4801105	5.55766335	0.00950009	0.020115138
SPAC1834.09	mug51	variant protein kinase 19 family protein	-0.4804654	4.72293515	0.05556989	0.094894132
SPBC21C3.01c	vps1301	chorein homolog Vps13a (predicted)	-0.4805117	8.5795419	4.38E-08	2.46E-07
SPCC188.07	ccq1	shelterin complex HEAT repeat subunit Ccq1	-0.4806456	6.60061934	0.00017899	0.00056467
SPBC31F10.13c	hip1	histone H3.3 H4 chaperone, hira family Hip1	-0.4809179	8.03042086	1.60E-07	8.20E-07
SPAC14C4.13	rad17	RFC related checkpoint protein Rad17	-0.4813212	6.57571224	0.00077105	0.002131866
SPNCRNA.901	#N/A	#N/A	-0.4817842	5.20944294	0.01512875	0.030567434
SPCC736.07c	uri1	unconventional prefoldin chaperone involved protein compl	-0.482049	6.92039695	0.00021811	0.000680212
SPAC3H1.08c	SPAC3H1.08c	mitochondrial calcium uniporter regulator (predicted)	-0.4829834	6.06813035	0.00089233	0.002425042
SPNCRNA.1328	#N/A	#N/A	-0.4831969	7.2454219	8.64E-06	3.45E-05
SPNCRNA.1037	#N/A	#N/A	-0.4837192	6.7860994	3.34E-05	0.000119595
SPCC737.08	mdn1	midasin, Mdn1	-0.484265	9.90160426	1.26E-11	1.10E-10
SPAC6F6.06c	rax2	cell polarity factor Rax2	-0.4844842	8.46030807	5.34E-08	2.95E-07
SPAC8E11.05c	SPAC8E11.05c	DUF5102 family conserved fungal protein, associated with	-0.484744	7.23364894	4.52E-06	1.90E-05
SPAC12B10.01c	SPAC12B10.01c	HECT-type ubiquitin-protein ligase E3, implicated in negativ	-0.4853547	9.2529247	1.76E-09	1.19E-08
SPBC354.08c	rsn1	Golgi to plasma membrane transport protein Rsn1 (predict	-0.4857244	7.81296154	2.25E-08	1.30E-07
SPBC660.08	SPBC660.08	Schizosaccharomyces specific protein	-0.4859926	7.37266096	1.90E-06	8.44E-06
SPCC23B6.03c	tel1	ATM checkpoint kinase	-0.4861231	7.80737818	4.09E-07	1.99E-06
SPNCRNA.1650	#N/A	#N/A	-0.4866131	4.60460316	0.15249912	0.224234337
SPBC428.17c	wpl1	cohesin loading/unloading factor (WAPL) Wpl1	-0.4874967	6.75654858	0.00180188	0.004563374
SPAC23C4.16c	atg15	autophagy associated lysophospholipase Atg15	-0.4878848	7.54671971	1.34E-07	6.93E-07
SPNCRNA.776	#N/A	#N/A	-0.4883003	6.06585878	0.00079963	0.002200368
SPAC18B11.04	ncs1	neuronal calcium sensor related protein Ncs1	-0.4884223	7.35607485	0.00024226	0.000748676
SPNCRNA.638	#N/A	#N/A	-0.4886385	5.81431886	0.00360062	0.008464167
SPBC32C12.02	ste11	transcription factor Ste11	-0.4891491	8.8841278	0.00078932	0.002173757
SPAC23C11.03	mpp10	U3 snoRNP-associated protein Mpp10 (predicted)	-0.4893958	7.02530481	4.43E-05	0.000155078
SPBC336.01	fbh1	DNA helicase I, ubiquitin ligase F-box adaptor Fbh1	-0.4896702	7.03348451	4.89E-06	2.05E-05
SPNCRNA.1428	#N/A	#N/A	-0.4896886	6.97554453	5.14E-05	0.000178523
SPCC63.14	eis1	eisosome assembly protein eis1	-0.4897181	9.14573365	8.21E-05	0.000273842
SPCC11E10.08	rik1	CLRC ubiquitin ligase complex WD repeat protein Rik1	-0.4902953	7.1305286	5.03E-06	2.10E-05
SPAC8F11.02c	dph3	diphthamide biosynthesis protein Dph3 (predicted)	-0.4906999	5.76250581	0.00432792	0.009976804
SPNCRNA.800	#N/A	#N/A	-0.4907876	4.57270716	0.04564871	0.080020464
SPAC1B3.04c	guf1	mitochondrial elongation factor GTPase Guf1 (predicted)	-0.4909524	6.80278877	2.23E-05	8.28E-05
SPAC56F8.16	esc1	transcription factor Esc1 (predicted)	-0.4917436	8.24796907	1.82E-06	8.11E-06
SPBC30B4.02c	sqs1	R3H and G-patch domain protein Sqs1 (predicted)	-0.4918135	6.97675254	4.99E-05	0.000173813
SPNCRNA.1236	#N/A	#N/A	-0.4920552	5.16400275	0.05965842	0.100613517
SPNCRNA.1213	#N/A	#N/A	-0.4921237	6.25581649	0.00097653	0.00262673
SPCC330.20	tam14	stress associated Endoplasmic Reticulum protein Tam14	-0.4923644	4.47841289	0.09019599	0.143844316
SPBC13E7.02	cwf24	ubiquitin-protein ligase E3/GCN5-related N acetyltransfera	-0.4925728	6.54476537	0.00014453	0.000464289
SPBC119.07	ppk19	serine/threonine protein kinase Ppk19	-0.4928747	7.3469935	3.60E-05	0.000128315
SPAC1952.03	otu2	ubiquitin specific cysteine protease, OTU family, Otu2	-0.4931171	5.8844567	0.0020149	0.005050246
SPBC12C2.03c	SPBC12C2.03c	methionine synthase reductase (predicted)	-0.4934322	6.5319807	0.00145509	0.003756793
SPCC1884.02	nic1	plasma membrane NiCoT heavy metal ion transmembrane	-0.4937302	7.16259317	6.39E-05	0.000217992
SPNCRNA.104	#N/A	#N/A	-0.494014	3.06094314	0.36601263	0.461329127
SPAC13G6.14	aps1	diadenosine 5',5'''-p1,p6-hexaphosphate hydrolase Aps1	-0.4947524	6.75683513	0.0005531	0.001580111
SPAC6B12.08	mug185	DNAJ domain protein Mug185 (predicted)	-0.4949688	6.8469256	9.31E-06	3.71E-05

SPNCRNA.175	#N/A	#N/A	-0.4952389	2.6843569	0.42248371	0.517738462
SPNCRNA.24	#N/A	#N/A	-0.4953631	3.21914336	0.28785931	0.380513323
SPBC1711.15c	SPBC1711.15c	Schizosaccharomyces pombe specific protein	-0.4962085	4.63633466	0.11685218	0.179215484
SPBC13E7.01	cwf22	splicing factor Cwf22	-0.4962105	7.7181717	2.22E-05	8.26E-05
SPAC4H3.05	srs2	ATP-dependent DNA helicase, UvrD subfamily	-0.4963548	6.93586919	1.05E-05	4.15E-05
SPCC1281.07c	gst4	glutathione S-transferase (predicted)	-0.4963606	7.50925713	2.06E-05	7.71E-05
SPAC664.15	caf4	CCR4-Not complex subunit Caf4/Mdv1 (predicted)	-0.4966849	7.34566393	0.00051667	0.001491061
SPBC405.05	atg16	autophagy associated protein Atg16	-0.4975174	5.09189462	0.01485519	0.03008618
SPBP35G2.10	mit1	SHREC complex ATP-dependent DNA helicase subunit Mit1	-0.4975922	7.02765725	3.06E-05	0.00011034
SPNCRNA.1206	#N/A	#N/A	-0.4976236	8.11801201	1.29E-08	7.74E-08
SPAC12G12.09	eti1	WD repeat protein associated with stress granule	-0.4979536	7.36866936	1.64E-07	8.37E-07
SPAC20G8.08c	fft1	SMARCAD1 family ATP-dependent DNA helicase Fft1 (predi	-0.498061	7.1796704	3.67E-05	0.000130694
SPNCRNA.721	#N/A	#N/A	-0.4980953	2.73200877	0.35394008	0.44944248
SPAC2F7.17	mrf1	mitochondrial translation release factor (predicted)	-0.4989041	6.41128361	0.0003378	0.001014441
SPNCRNA.05	#N/A	#N/A	-0.4989986	5.95231509	0.00206686	0.005163364
SPAC56F8.06c	alg10	dolichyl-phosphate-glucose-glycolipid alpha-glucosyltransfe	-0.4990124	5.03640585	0.02565269	0.048652891
SPAC30D11.11	izh3	ER membrane Haemolysin-III family protein involved in zinc	-0.4990826	7.88446059	9.33E-09	5.72E-08
SPAC977.03	SPAC977.03	methyltransferase (predicted)	-0.4992	4.18823326	0.08602963	0.137878386
SPNCRNA.1012	#N/A	#N/A	-0.4992342	5.22078019	0.03158327	0.058324056
SPAC2F3.14c	saf2	splicing associated factor Saf2	-0.4992847	5.81207528	0.00836098	0.017914506
SPNCRNA.696	#N/A	#N/A	-0.4994989	4.69109268	0.0405022	0.072283625
SPCC16C4.02c	SPCC16C4.02c	Armadillo-type fold protein, DUF1941 family protein, huma	-0.4997851	7.448348	1.04E-06	4.80E-06
SPCC1183.11	msy1	MS calcium ion channel protein Msy1	-0.5001987	7.97886929	1.35E-09	9.28E-09
SPAC5D6.07c	pxa1	PXA domain protein Pxa1	-0.5004775	5.49536924	0.01710364	0.034122035
SPAC22E12.18	SPAC22E12.18	human CCNDBP1 ortholog	-0.5006287	6.28281576	0.00076849	0.002126704
SPAC22F8.07c	rtf1	replication termination factor Rtf1	-0.5009635	5.80151778	0.00277815	0.00672787
SPBC29A10.05	exo1	exonuclease I Exo1	-0.5010388	8.33009778	5.51E-10	3.96E-09
SPBC29A10.14	rec8	meiotic cohesin complex subunit Rec8	-0.5012449	4.87393219	0.04846748	0.084288578
SPAC11H11.01	sst6	ESCRT I complex subunit Vps23	-0.5017394	5.33704148	0.02300833	0.044279324
SPAC1420.01c	SPAC1420.01c	GATA-like domain protein (predicted)	-0.5019824	8.10807815	4.57E-09	2.92E-08
SPCC10H11.01	prp11	ATP-dependent RNA helicase Prp11	-0.5020489	7.43581687	0.00037174	0.001105151
SPNCRNA.911	#N/A	#N/A	-0.5021293	4.074193	0.1093613	0.169175635
SPAC3C7.05c	mug191	alpha-1,6- mannanase (predicted)	-0.5025224	7.95307055	1.22E-05	4.77E-05
SPNCRNA.538	#N/A	#N/A	-0.5039393	2.23742336	0.5795262	0.66661186
SPBC30D10.09c	SPBC30D10.09c	ER membrane organization protein, HVA22/TB2/DP1 family	-0.5052549	4.81200352	0.02208891	0.04276387
SPAC630.11	vps55	vacuolar sorting protein Vps55 (predicted)	-0.5055546	5.88654157	0.00152067	0.003912356
SPAC57A10.05c	pof1	F-box/WD repeat protein Pof1	-0.5059087	6.8217224	0.00050915	0.00147337
SPNCRNA.1029	#N/A	#N/A	-0.5060075	4.8899017	0.04955172	0.085998333
SPBC31F10.09c	nut2	mediator complex subunit Med10	-0.5063418	6.21504937	0.00033871	0.001016737
SPAC23D3.11	ayr1	1-acyldihydroxyacetone phosphate reductase Ayr1 (predicte	-0.5064539	6.80291741	0.00012807	0.000414794
SPBC26H8.09c	snf59	SWI/SNF complex subunit Snf59	-0.5065168	6.50082455	0.00248039	0.006084737
SPNCRNA.1305	#N/A	#N/A	-0.506519	8.96639992	2.59E-11	2.19E-10
SPNCRNA.1142	#N/A	#N/A	-0.5068067	5.21215263	0.0123677	0.025503703
SPAC17D4.03c	cis4	Golgi cation diffusion family zinc transmembrane transport	-0.5068664	7.15370734	3.57E-05	0.000127747
SPBP8B7.23	rnf10	ubiquitin-protein ligase E3 (predicted)	-0.5074412	8.18591323	2.59E-08	1.50E-07
SPAC4H3.08	SPAC4H3.08	3-hydroxyacyl-CoA dehydrogenase (predicted)	-0.508218	7.48080208	0.00184114	0.004650696
SPBC3E7.05c	mic60	MICOS complex subunit Mic60 (predicted)	-0.5087899	6.10701644	0.00074258	0.002066762

SPBC15D4.13c	SPBC15D4.13c	activating signal cointegrator 1 complex subunit, human AS	-0.5092015	4.37786664	0.0683663	0.113030287
SPAC27E2.01	SPAC27E2.01	alpha-amylase homolog (predicted)	-0.5092136	5.62956265	0.03605062	0.065384986
SPCC320.07c	mde7	RNA-binding protein Mde7	-0.5095899	6.3292633	0.00263736	0.006414313
SPCC4F11.05	#N/A	#N/A	-0.509765	4.75999874	0.04918941	0.08544406
SPAC105.01c	kha1	plasma membrane potassium ion/proton antiporter Kha1 (p	-0.5097984	7.29622972	1.39E-06	6.29E-06
SPNCRNA.440	#N/A	#N/A	-0.5098867	5.34312198	0.00764969	0.01656784
SPAC2E1P5.04c	cwg2	geranylgeranyltransferase I beta subunit Cwg2	-0.5100465	5.91156242	0.00254955	0.006231925
SPAC56F8.15	SPAC56F8.15	Schizosaccharomyces pombe specific protein	-0.5107452	6.18244174	0.00170024	0.004335011
SPAC17G8.09	shg1	Set1C complex subunit Shg1	-0.5121167	5.22658957	0.00814011	0.017501868
SPCC320.14	sry1	serine racemase Sry1	-0.5127003	7.44107359	2.59E-07	1.29E-06
SPAC15A10.12c	tca17	TRAPP complex subunit 2-like Tca17 (predicted)	-0.5127094	5.83631968	0.00359484	0.008455879
SPBC14C8.02	tim44	TIM23 translocase complex subunit Tim44 (predicted)	-0.5128011	6.48855247	8.16E-05	0.000272518
SPAC17C9.05c	pmc3	mediator complex subunit Med27	-0.5129379	5.00169503	0.08106567	0.130940392
SPBP35G2.13c	swc2	Swr1 complex subunit Swc2	-0.513048	6.85279703	1.10E-05	4.31E-05
SPNCRNA.620	#N/A	#N/A	-0.5133748	5.90849985	0.00251955	0.006169672
SPAC1751.04	loc1	ribosome biogenesis protein Loc1 (predicted)	-0.5134382	5.84187825	0.0010562	0.00281859
SPAC3C7.04	SPAC3C7.04	transcription factor (predicted)	-0.5136709	7.9984334	5.72E-10	4.10E-09
SPAC8E11.06	SPAC8E11.06	Schizosaccharomyces pombe specific protein	-0.513699	3.09936705	0.28596401	0.378964958
SPCC970.09	sec8	exocyst complex subunit Sec8	-0.5137465	7.97478129	7.18E-07	3.38E-06
SPAC29E6.10c	nst1	conserved fungal NST1 family protein	-0.5146914	7.82436391	8.96E-07	4.16E-06
SPAC1F5.11c	tra2	NuA4 complex phosphatidylinositol pseudokinase complex s	-0.515943	10.2082488	4.55E-15	5.49E-14
SPCC548.06c	ght8	plasma membrane hexose:proton symporter, unknown spec	-0.5161838	9.90082704	5.96E-12	5.41E-11
SPAC23C11.04c	pnk1	DNA kinase/phosphatase Pnk1	-0.5164214	7.30634212	6.61E-07	3.13E-06
SPAC1952.01	gab1	Pig-U, Gab1 (predicted)	-0.5164411	5.91881701	0.00675902	0.014832348
SPAC56E4.06c	ggt2	gamma-glutamyltranspeptidase Ggt2	-0.5165075	7.78698454	1.12E-07	5.90E-07
SPAC688.08	srb8	mediator complex subunit Med12	-0.5167218	7.72677416	1.98E-08	1.16E-07
SPBC2G5.07c	rpc25	DNA-directed RNA polymerase III complex subunit Rpc25	-0.5168943	6.45356058	0.00028293	0.000860672
SPNCRNA.454	#N/A	#N/A	-0.5173654	4.79158068	0.01985562	0.038983688
SPAPB1A10.11c	mse1	mitochondrial glutamyl-tRNA ligase Mse1 (predicted)	-0.5182181	6.26851464	0.00078104	0.002154418
SPAC16.01	rho2	Rho family GTPase Rho2	-0.5187259	6.04882252	0.0009782	0.002630049
SPCC1919.15	brl1	ubiquitin-protein ligase E3 Brl1	-0.5191051	6.8333516	2.93E-05	0.0001061
SPNCRNA.1276	#N/A	#N/A	-0.5191051	6.8333516	2.93E-05	0.0001061
SPNCRNA.1425	#N/A	#N/A	-0.5191497	1.90514662	0.58905608	0.674722541
SPAC343.03	apc11	anaphase-promoting complex ubiquitin -protein ligase E3 su	-0.5194548	6.51823423	6.57E-05	0.000223714
SPBC776.16	mis20	centromere protein Mis20/Eic2	-0.519798	4.83431527	0.06402305	0.107072222
SPAC4F8.13c	rng2	RasGAP GTPase activating protein IQGAP Rng2	-0.5199381	8.81538676	2.98E-11	2.50E-10
SPAC13A11.04c	ubp8	SAGA complex ubiquitin C-terminal hydrolase Ubp8	-0.5204691	7.27865096	8.06E-07	3.76E-06
SPNCRNA.1391	#N/A	#N/A	-0.5213684	6.42508467	8.77E-05	0.000291624
SPBC8E4.02c	#N/A	#N/A	-0.5217392	7.1222645	8.73E-06	3.49E-05
SPBC25H2.14	mug16	UNC-50 family protein, implicated in vesicle-mediated tran	-0.5218809	5.51862741	0.00549334	0.012351382
SPNCRNA.889	#N/A	#N/A	-0.522677	6.37228267	4.41E-05	0.000154502
SPAPB15E9.03c	Tf2-5	retrotransposable element/transposon Tf2-type	-0.5226988	7.13715683	5.56E-05	0.000191558
SPCC1442.02	glm2	Glomulin, ubiquitin-protein transferase inhibitor Glm2	-0.5228253	6.53561124	3.27E-05	0.000117167
SPBP8B7.22	erd2	HDEL receptor (predicted)	-0.5233075	6.96065281	6.71E-06	2.74E-05
SPCC16C4.21	#N/A	#N/A	-0.5245794	3.30113352	0.19139972	0.270728565
SPCC737.07c	SPCC737.07c	DNA polymerase alpha-associated DNA helicase A (predicte	-0.5248551	5.62850766	0.00261579	0.006368682
SPNCRNA.189	#N/A	#N/A	-0.5250159	1.47743349	0.83339639	0.881178125

SPNCRNA.1684	#N/A	#N/A	-0.5253756	5.2528987	0.01240741	0.025570094
SPNCRNA.1547	#N/A	#N/A	-0.526937	3.84388547	0.15852006	0.231636032
SPBC1652.02	SPBC1652.02	APC amino acid transmembrane transporter (predicted)	-0.5269643	8.21173133	1.13E-10	8.77E-10
SPAC637.03	SPAC637.03	DUF1774 family multi-spanning conserved fungal membrar	-0.527136	8.14711136	3.62E-07	1.77E-06
SPCC895.05	for3	formin For3	-0.5279584	7.11305626	7.36E-07	3.45E-06
SPCC417.05c	cfh2	SEL1 repeat protein, chitin synthase regulatory factor-like C	-0.5281858	8.68944255	2.59E-11	2.19E-10
SPBC16E9.20	#N/A	#N/A	-0.52833	2.94684795	0.36256197	0.458082842
SPNCRNA.937	#N/A	#N/A	-0.5294935	6.13802475	0.00033665	0.001011434
SPBP8B7.04	mug45	Schizosaccharomyces specific protein Mug45	-0.529499	5.33582125	0.00842401	0.018038193
SPBC530.12c	pdf1	palmitoyl protein thioesterase/ dolichol pyrophosphate pho	-0.5299725	8.18576875	3.07E-06	1.33E-05
SPAC11G7.03	idh1	isocitrate dehydrogenase (NAD+) subunit 1 Idh1	-0.5320415	8.35004839	2.31E-09	1.52E-08
SPNCRNA.1537	#N/A	#N/A	-0.5321218	4.11599101	0.13655356	0.204499779
SPAC15E1.09	grx2	glutaredoxin Grx2	-0.5322072	6.29783174	5.99E-05	0.000205185
SPBC21C3.06	SPBC21C3.06	Schizosaccharomyces specific protein	-0.532417	6.40308988	0.00014144	0.00045464
SPCC1393.02c	spt2	non-specific DNA binding protein Spt2 (predicted)	-0.5333326	7.21340163	1.61E-07	8.24E-07
SPBC649.03	rhp14	XP-A family homolog Rhp14	-0.5333548	6.1101595	0.00024927	0.000767211
SPNCRNA.949	#N/A	#N/A	-0.5342095	7.74844358	1.32E-08	7.90E-08
SPNCRNA.577	#N/A	#N/A	-0.5352055	8.18000055	5.62E-11	4.52E-10
SPBC27B12.07	SPBC27B12.07	mitochondrial calcium uniporter regulator	-0.5357225	6.74630766	0.00022643	0.000702618
SPAC2G11.03c	vps45	vacuolar sorting protein Vps45	-0.5359238	7.60398077	2.22E-07	1.12E-06
SPNCRNA.329	#N/A	#N/A	-0.5364037	5.02075169	0.02445129	0.046673509
SPAC1556.05c	cgr1	ribosome biogenesis CGR1 family (predicted)	-0.5365817	6.53609953	0.00053591	0.001538756
SPAC1093.06c	dhc1	minus-end directed microtubule motor, dynein heavy chain	-0.5369308	8.23488861	1.38E-05	5.34E-05
SPBC1604.17c	tti2	ASTRA complex subunit Tti2	-0.5369451	5.81471863	0.00139332	0.003612015
SPAC1486.08	cox16	mitochondrial copper chaperone for cytochrome c oxidase C	-0.5370641	6.19229135	0.00041805	0.001228349
SPAC1F12.08	cab4	cytidylyltransferase (predicted)	-0.5372112	6.13762947	0.00025931	0.000795216
SPAC4G8.12c	smp3	alpha-1,2-mannosyltransferase Smp3 (predicted)	-0.5379807	5.35028898	0.05616134	0.095682409
SPAC19G12.04	dal1	ureidoglycolate hydrolase (predicted)	-0.5389135	5.28002686	0.00505818	0.011462165
SPBC1709.11c	png2	ING family histone acetyltransferase complex PHD-type zin	-0.5394775	7.13069612	2.89E-05	0.000105079
SPNCRNA.863	#N/A	#N/A	-0.5396155	6.92317924	0.01523888	0.030753442
SPAC19B12.08	atg4	Atg8 deconjugator Atg4 (predicted)	-0.5396781	6.44324097	7.61E-05	0.000255533
SPAC6F12.14	cut23	anaphase-promoting complex, TPR lobe subcomplex subuni	-0.5397099	6.75961285	0.00015665	0.000500239
SPAC1786.01c	ptl2	triacylglycerol lipase ptl2	-0.5398674	8.45375368	6.15E-08	3.36E-07
SPNCRNA.1558	#N/A	#N/A	-0.5400848	5.99634513	0.00117391	0.003099917
SPBC3B8.04c	SPBC3B8.04c	phosphate (Pi) transmembrane transporter (predicted)	-0.5409437	8.30458872	2.89E-08	1.66E-07
SPNCRNA.74	#N/A	#N/A	-0.541026	6.40839251	9.96E-05	0.000328768
SPAC1834.03c	hhf1	histone H4 h4.1	-0.5421891	7.94545672	1.03E-08	6.26E-08
SPNCRNA.1409	#N/A	#N/A	-0.5434877	3.6837232	0.30737145	0.400778443
SPCC338.08	ctp1	CtIP-related endonuclease	-0.5435091	6.93526343	1.07E-06	4.93E-06
SPBC409.12c	stn1	telomere cap complex subunit Stn1	-0.5439819	6.25252462	0.00020972	0.000655542
SPNCRNA.328	#N/A	#N/A	-0.5442116	2.39227375	0.38199077	0.477576182
SPAC2G11.15c	tgs1	rRNA methyltransferase Tgs1	-0.5446054	5.20680101	0.00358705	0.008440487
SPBC119.04	mei3	meiosis inducing protein Mei3	-0.5448797	1.47697924	0.8016669	0.855014347
SPNCRNA.1221	#N/A	#N/A	-0.5450077	2.5838234	0.34514815	0.440002469
SPCC4G3.08	psk1	ribosomal protein S6 kinase Psk1	-0.5454646	7.31863285	1.49E-06	6.72E-06
SPAC1B3.07c	vps28	ESCRT I complex subunit Vps28	-0.5455776	6.24827183	0.00027719	0.000844344
SPBP35G2.04c	SPBP35G2.04c	Schizosaccharomyces specific protein	-0.5456612	5.21970705	0.01047966	0.021983388

SPBC887.08	SPBC887.08	Schizosaccharomyces specific protein	-0.5464336	4.64262089	0.03513199	0.063889154
SPAC19B12.07c	SPAC19B12.07c	zinc finger C2H2-type, human ZNF277 ortholog, implicated	-0.5468582	5.91886956	0.00092552	0.002505889
SPNCRNA.1524	#N/A	#N/A	-0.5471045	6.44282968	0.00013276	0.000428971
SPCC10H11.02	cwf23	DNAJ domain protein Cwf23	-0.5474748	5.76745129	0.00336024	0.007970163
SPAC26F1.08c	SPAC26F1.08c	malate transmembrane transporter (predicted)	-0.5480872	6.97464982	7.03E-07	3.32E-06
SPNCRNA.1270	#N/A	#N/A	-0.5482957	5.5348948	0.00250113	0.006131183
SPAC14C4.02c	smc5	Smc5-6 complex SMC P-loop ATPase subunit Smc5	-0.5489405	7.57644027	3.57E-07	1.75E-06
SPCC777.15	dus4	tRNA dihydrouridine synthase Dus4 (predicted)	-0.5490111	5.82626719	0.00738702	0.016061226
SPCC1840.02c	bgs4	cell wall and secondary septum 1,6 branched 1,3-beta-gluca	-0.5505609	9.77782619	2.59E-12	2.45E-11
SPBC211.06	gfh1	gamma tubulin complex subunit Gfh1	-0.5511387	7.26117614	7.11E-06	2.89E-05
SPBC713.07c	SPBC713.07c	vacuolar polyphosphatase (predicted)	-0.5512105	8.45131342	1.90E-10	1.44E-09
SPBC337.07c	ecm14	carboxypeptidase Ecm14 (predicted)	-0.5513259	8.05685223	3.04E-11	2.55E-10
SPCC550.08	SPCC550.08	N-acetyltransferase (predicted)	-0.5514357	6.02585512	0.00027275	0.000832329
SPAC823.04	rrp36	rRNA processing protein Rrp36 (predicted)	-0.552378	4.92241057	0.01161492	0.024126845
SPCC622.04	#N/A	#N/A	-0.5525221	4.31651665	0.04590266	0.080403502
SPNCRNA.111	#N/A	#N/A	-0.5525532	7.64534036	1.03E-07	5.42E-07
SPAC30D11.01c	gto2	alpha-glucosidase (predicted)	-0.5527156	7.43735137	1.12E-06	5.13E-06
SPAC22H10.07	scd2	Cdc42 GTPase complex scaffold subunit Scd2	-0.5532612	7.31557521	5.10E-08	2.83E-07
SPBC26H8.04c	iml1	GTPase-activating protein subunit of SEA and Iml1 complex	-0.5535304	7.71908209	1.73E-09	1.17E-08
SPAC1834.04	hht1	histone H3 h3.1	-0.5541272	8.74424046	4.70E-11	3.83E-10
SPBC29A3.05	vps71	Swr1 complex subunit Vps71	-0.5547487	4.79505221	0.10512265	0.16339872
SPAC1A6.04c	plb1	phospholipase B homolog Plb1	-0.5547601	10.3911557	1.89E-12	1.82E-11
SPBC1711.11	atg2402	autophagy associated protein Atg24c	-0.5550153	5.51714945	0.00145373	0.003755733
SPNCRNA.861	#N/A	#N/A	-0.5553622	5.20678802	0.02081955	0.04056028
SPNCRNA.948	#N/A	#N/A	-0.5556407	5.31561943	0.00296036	0.007110926
SPNCRNA.642	#N/A	#N/A	-0.5564091	3.87618327	0.07836716	0.127003698
SPAC19G12.09	SPAC19G12.09	NADH/NADPH-dependent indole-3-acetaldehyde reductase,	-0.556534	8.54996983	9.04E-11	7.07E-10
SPAC22F8.09	rrp16	rRNA processing protein Rrp16 (predicted)	-0.5566276	6.74462416	1.80E-05	6.79E-05
SPNCRNA.1356	#N/A	#N/A	-0.5567591	4.21271478	0.11096773	0.171349085
SPCC417.11c	SPCC417.11c	glutamate-1-semialdehyde 2,1-aminomutase (predicted)	-0.5569585	6.87090987	1.53E-06	6.89E-06
SPCC4B3.10c	ipk1	inositol 1,3,4,5,6-pentakisphosphate (IP5) kinase	-0.5572692	5.20212265	0.00432632	0.009976491
SPCC1020.05	dcr2	phosphoprotein phosphatase involved in unfolded protein re	-0.5576667	8.69392076	2.84E-14	3.17E-13
SPAC922.09	#N/A	#N/A	-0.5579572	3.2855828	0.16055903	0.2340626
SPNCRNA.772	#N/A	#N/A	-0.5584317	5.8857292	0.00071065	0.001987646
SPCC663.02	wtf14	wtf element Wtf14	-0.5591581	6.97949123	0.00058174	0.001654294
SPNCRNA.1279	#N/A	#N/A	-0.5591817	6.8576051	5.55E-06	2.31E-05
SPAC589.08c	dam1	DASH complex subunit Dam1	-0.5592816	5.14515908	0.00468858	0.010742693
SPBC16C6.03c	rsa1	ribosome assembly protein Rsa1 (predicted)	-0.5595684	5.45587698	0.00197269	0.004960875
SPBC56F2.14	mrpl44	mitochondrial ribosomal protein subunit l44 (predicted)	-0.5596873	5.20349063	0.00726215	0.015819113
SPNCRNA.1326	#N/A	#N/A	-0.560514	1.82117668	0.41881226	0.513887028
SPAC2F3.10	vps54	GARP complex subunit Vps54 (predicted)	-0.5606943	7.75766505	3.12E-09	2.03E-08
SPNCRNA.945	#N/A	#N/A	-0.5606982	4.66867341	0.0186957	0.037037623
SPCC1442.15c	cox18	mitochondrial inner membrane insertase Cox18	-0.5610758	5.31149225	0.00369472	0.008648973
SPBC18E5.13	mti3	mitochondrial translation initiation factor Mti3 (predicted)	-0.5614626	5.68438223	0.00076284	0.002113653
SPAC977.10	nhe1	plasma membrane/prospore membrane sodium ion/protor	-0.5617303	7.90789972	7.13E-09	4.45E-08
SPNCRNA.970	#N/A	#N/A	-0.5619802	5.43521687	0.00538343	0.012122503
SPNCRNA.877	#N/A	#N/A	-0.5620353	2.33236165	0.40308347	0.498629408

SPBC16D10.05	mok13	alpha-1,3-glucan synthase Mok13	-0.5629229	8.82670978	4.61E-13	4.70E-12
SPBC18H10.11c	ppr2	mitochondrial PPR repeat protein Ppr2	-0.5644762	5.62545664	0.00546658	0.012297556
SPBC3D6.10	apn2	AP-endonuclease Apn2	-0.5645823	7.343144	3.68E-08	2.10E-07
SPAC12G12.10	wdr21	WD repeat protein, human DDB1 and CUL4-associated facto	-0.5648403	7.09718775	3.69E-05	0.000131364
SPAC22F8.05	SPAC22F8.05	alpha,alpha-trehalose-phosphate synthase (predicted)	-0.5657398	8.67647483	0.00028832	0.000875243
SPNCRNA.743	#N/A	#N/A	-0.565836	5.64002803	0.00220144	0.00547544
SPBC24C6.06	gpa1	G-protein alpha subunit	-0.5668855	7.7013504	6.51E-10	4.63E-09
SPAC29B12.13	SPAC29B12.13	S-(hydroxymethyl)glutathione synthase activity (predicted)	-0.56702	5.81274198	0.00119971	0.003164361
SPAC17A2.09c	csx1	RNA-binding protein Csx1	-0.5675102	8.13985788	2.16E-10	1.63E-09
SPBP22H7.04	SPBP22H7.04	mitochondrial membrane protein, human TMEM186 orthol	-0.5683846	6.80496019	9.22E-07	4.28E-06
SPNCRNA.1569	#N/A	#N/A	-0.568735	4.64789459	0.02260416	0.043600141
SPNCRNA.1600	#N/A	#N/A	-0.5690606	5.49392116	0.01624417	0.032540931
SPBC1347.11	sro1	stress responsive orphan 1	-0.5695717	6.83929524	2.37E-05	8.76E-05
SPNCRNA.1625	#N/A	#N/A	-0.5710436	6.12086987	0.00013156	0.000425343
SPNCRNA.980	#N/A	#N/A	-0.5717759	6.89245745	7.14E-06	2.91E-05
SPBP22H7.05c	abo2	ATPase with bromodomain protein (predicted)	-0.5729305	7.55462659	3.58E-08	2.04E-07
SPBC2D10.15c	pth1	mitochondrial peptidyl-tRNA hydrolase Pth1 (predicted)	-0.5732978	5.18733229	0.01020206	0.021480508
SPBP4H10.06c	cut14	condensin complex SMC subunit Smc2	-0.57498	7.86412175	4.05E-08	2.29E-07
SPAC23C4.05c	SPAC23C4.05c	LEA domain protein	-0.5756243	7.47219818	1.23E-06	5.62E-06
SPBC577.05c	rec27	meiotic recombination protein Rec27	-0.5763352	2.26841394	0.37017701	0.465620502
SPNCRNA.997	#N/A	#N/A	-0.5763774	5.34567665	0.00199799	0.00501562
SPBC17A3.06	SPBC17A3.06	phosphoprotein phosphatase (predicted)	-0.5763916	5.27125807	0.00497722	0.011305075
SPCC132.03	SPCC132.03	Schizosaccharomyces specific protein	-0.577773	6.56542637	2.72E-06	1.19E-05
SPBC713.06	adl1	DNA ligase (predicted)	-0.5781472	8.65849654	2.01E-12	1.93E-11
SPCC16A11.08	atg20	autophagy associated PX/BAR domain sorting nexin Atg20	-0.5791859	5.75647707	0.00135684	0.003522822
SPAC19A8.02	sip3	ER-plasma membrane tethering protein Sip3 (predicted)	-0.5800301	8.7003582	2.36E-10	1.78E-09
SPNCRNA.1683	#N/A	#N/A	-0.5800966	4.67382716	0.02549577	0.048382233
SPBC16E9.15	SPBC16E9.15	heat shock factor binding protein, implicated in trancription	-0.58129	6.12042103	0.00029098	0.000881608
SPAC22F3.09c	res2	MBF transcription factor complex subunit Res2	-0.5817213	6.91652995	4.15E-06	1.76E-05
SPBC354.11c	#N/A	#N/A	-0.5829751	6.35575633	3.69E-05	0.000131364
SPNCRNA.1046	#N/A	#N/A	-0.5830737	6.03325461	0.00021873	0.000681851
SPBC32F12.05c	cwf12	Prp19 complex subunit Cwf12	-0.5833827	6.54814446	7.07E-06	2.88E-05
SPBP19A11.04c	mor2	morphogenesis protein Mor2	-0.5834121	8.14397985	4.62E-08	2.59E-07
SPNCRNA.862	#N/A	#N/A	-0.5844777	7.20047225	0.0005094	0.00147337
SPAC3A11.05c	kms1	meiotic spindle pole body KASH domain protein Kms1	-0.5848525	5.9440895	0.00162943	0.004160697
SPBC1604.02c	ppr1	mitochondrial PPR repeat protein Ppr1	-0.5853275	8.27228443	1.08E-09	7.54E-09
SPNCRNA.1492	#N/A	#N/A	-0.5854078	6.29184649	8.56E-06	3.43E-05
SPNCRNA.1546	#N/A	#N/A	-0.5856181	6.89636696	4.20E-07	2.04E-06
SPCC24B10.18	SPCC24B10.18	DUF2462 family protein, human Leydig cell tumor 10 kDa p	-0.5858475	5.87744475	0.00039075	0.001156117
SPCC1020.14	Tf2-12	retrotransposable element/transposon Tf2-type	-0.5863948	5.17846162	0.00492139	0.0112065
SPBC19C2.04c	ubp11	ubiquitin C-terminal hydrolase Ubp11	-0.5864179	5.98929999	0.00216998	0.00540312
SPBC30D10.04	swi3	replication fork protection complex subunit Swi3	-0.5885861	5.38629767	0.00126742	0.003323623
SPAC23G3.01	rpb2	RNA polymerase II complex subunit Rpb2	-0.5887374	8.15966009	7.87E-12	7.08E-11
SPCC970.04c	mob2	protein kinase activator Mob2	-0.5888094	6.85880891	1.41E-06	6.37E-06
SPAC2G11.08c	smn1	SMN family protein Smn1	-0.5890902	5.26049881	0.00132959	0.003465544
SPAC11H11.03c	SPAC11H11.03c	ATP-dependent polydeoxyribonucleotide 5'-hydroxyl-kinase	-0.5898438	4.43537064	0.0402045	0.071789989
SPAC2E1P3.03c	Tf2-3	retrotransposable element/transposon Tf2-type	-0.5902515	7.6970509	6.06E-08	3.32E-07

SPAC6B12.19	rsa3	ribosome assembly protein Rsa3 (predicted)	-0.5903235	4.06225329	0.0635023	0.106305816
SPAC29A4.22	#N/A	#N/A	-0.5907985	5.45454799	0.00160888	0.004112856
SPAC11D3.08c	SPAC11D3.08c	amino acid transmembrane transporter (predicted)	-0.590806	7.74233353	1.60E-08	9.45E-08
SPNCRNA.1014	#N/A	#N/A	-0.5908845	5.79391215	0.00076211	0.00211248
SPBC1711.14	rec15	meiotic recombination protein Rec15	-0.5918372	3.59821521	0.14201405	0.211374798
SPAC20G4.05c	SPAC20G4.05c	mitochondrial protein, UPF0061 family, human SELENOO o	-0.5932661	7.04972199	3.46E-07	1.70E-06
SPCC970.10c	brl2	ubiquitin-protein ligase E3 Brl2	-0.5933765	7.42547313	4.93E-08	2.75E-07
SPAC1F3.02c	mkh1	MEK kinase (MEKK) Mkh1	-0.5937896	8.01490488	2.34E-09	1.54E-08
SPCC16C4.01	sif2	mitochondrial protein, involved in mitochondrial gene expre	-0.5945827	6.48989876	6.08E-06	2.51E-05
SPAC29B12.11c	SPAC29B12.11c	human WW domain binding protein-2 ortholog	-0.594762	6.45549399	6.13E-05	0.000209425
SPAC1952.05	gcn5	SAGA complex histone acetyltransferase catalytic subunit G	-0.5955957	6.90544401	3.19E-07	1.58E-06
SPAC3A11.06	mvp1	sorting nexin Mvp1 (predicted)	-0.595993	6.51888289	5.99E-05	0.000205349
SPBC3B8.11	rrn6	RNA polymerase I general transcription initiation factor sub	-0.5960694	7.18892648	6.11E-07	2.91E-06
SPCC736.05	wtf7	wtf element Wtf7	-0.5966988	5.285419	0.00964613	0.020404689
SPCC417.07c	mto1	gamma tubulin complex linker Mto1	-0.5969106	7.79711305	1.70E-08	1.00E-07
SPNCRNA.671	#N/A	#N/A	-0.5972497	6.72058031	0.00010312	0.000339013
SPBC1105.14	rsv2	transcription factor Rsv2	-0.5976899	7.09862012	6.79E-07	3.21E-06
SPBC216.01c	psy2	protein phosphatase PP4 complex regulatory subunit 3 Psy2	-0.5976941	7.93849664	1.62E-10	1.23E-09
SPAC2F3.16	SPAC2F3.16	ubiquitin-protein ligase E3, implicated in DNA repair (predi	-0.5987156	7.29748665	6.49E-09	4.08E-08
SPBC16C6.02c	vps1302	chorein homolog Vps1302 (predicted)	-0.5988514	9.88066816	1.82E-14	2.06E-13
SPAC23C11.10	mpn1	poly(U)-specific exoribonuclease, producing 3' uridine cyclic	-0.5991935	4.4783731	0.02608651	0.049338164
SPBC713.11c	pmp3	plasma membrane proteolipid Pmp3 (predicted)	-0.5993683	7.26414635	4.72E-08	2.64E-07
SPAC824.05	vps16	HOPS/CORVET complex subunit Vps16 (predicted)	-0.5994926	7.44448384	8.17E-09	5.06E-08
ScpofMt29	#N/A	#N/A	-0.5995669	2.77624901	0.25298749	0.34308088
SPAC20G8.02	SPAC20G8.02	mitochondrial DDHD family phospholipase (predicted)	-0.6014904	7.66144203	1.93E-09	1.29E-08
SPNCRNA.1526	#N/A	#N/A	-0.6021237	6.85886836	6.60E-07	3.13E-06
SPBC216.03	SPBC216.03	NADP binding superfamily conserved fungal protein	-0.6025898	7.17082465	9.48E-09	5.81E-08
SPNCRNA.672	#N/A	#N/A	-0.6026498	6.60228123	9.26E-05	0.000306629
SPNCRNA.1151	#N/A	#N/A	-0.6026938	5.45024956	0.00225773	0.005607248
SPBC336.07	sfc3	transcription factor TFIIIC complex B box binding subunit Sf	-0.6032595	7.04056992	8.56E-08	4.58E-07
SPBC11G11.01	fis1	mitochondrial fission protein Fis1 (predicted)	-0.6035593	7.43028133	2.02E-10	1.52E-09
SPNCRNA.108	#N/A	#N/A	-0.6047857	3.60958411	0.1278613	0.193483687
SPNCRNA.66	#N/A	#N/A	-0.6048302	3.23525313	0.24714201	0.336831844
SPAC513.02	SPAC513.02	phosphoglycerate mutase/6-phosphofructo-2-kinase family	-0.6055863	4.88800485	0.0604589	0.101786933
SPBC3B9.21	dcp1	mRNA decapping complex regulatory subunit Dcp1	-0.6056465	4.98385325	0.0080477	0.017314131
SPBC1683.03c	SPBC1683.03c	transmembrane transporter (predicted)	-0.6079072	7.6968621	9.29E-12	8.26E-11
SPAC17A5.11	rec12	meiotic recombination endonuclease Rec12	-0.6081934	4.83359944	0.01615829	0.032397512
SPBC725.02	mpr1	histidine-containing response regulator phosphotransferase	-0.6083344	7.92426004	2.53E-12	2.41E-11
SPNCRNA.1026	#N/A	#N/A	-0.6086672	6.77613186	2.52E-06	1.10E-05
SPCC188.05	#N/A	#N/A	-0.609657	1.73103709	0.40315342	0.498629408
SPAPB2B4.06	SPAPB2B4.06	acyl-coenzyme A thioesterase	-0.6099243	6.44752461	5.13E-06	2.14E-05
SPNCRNA.57	#N/A	#N/A	-0.6107365	1.36231072	0.60260688	0.685971861
SPBC17G9.05	rct1	cyclophilin family peptidyl-prolyl cis-trans isomerase, RRM-	-0.6111952	6.3478878	4.16E-06	1.76E-05
SPACUNK4.17	SPACUNK4.17	NAD binding dehydrogenase family protein, human DHDH o	-0.6115016	7.1254357	6.41E-06	2.64E-05
SPNCRNA.780	#N/A	#N/A	-0.611552	2.8854436	0.17559373	0.252251494
SPNCRNA.438	#N/A	#N/A	-0.6119315	4.02501815	0.09387765	0.148843762
SPCC24B10.14c	xlf1	XRCC4-like nonhomologous end joining factor, Cernunnon X	-0.6128644	4.27166883	0.02362787	0.045317818

SPBC27B12.14	cbp4	mitochondrial respiratory complex assembly protein Cbp4 (|	-0.6141689	5.51884051	0.00215387	0.00536496
SPNCRNA.765	#N/A	#N/A	-0.6149876	3.533649	0.19278169	0.272400206
SPBP35G2.06c	nup131	nucleoporin, WD repeat Nup131	-0.6157603	7.53096954	6.52E-09	4.09E-08
SPBC2D10.16	mhf1	CENP-S ortholog, FANCM-MHF complex subunit Mhf1	-0.6161944	4.81134554	0.01084845	0.02268504
SPCC330.08	alg11	GDP-Man:Man3GlcNAc2-PP-Dol alpha-1,2-mannosyltransfe	-0.6166666	7.64345333	8.90E-12	7.94E-11
SPAC25B8.08	SPAC25B8.08	Golgi Ras-interacting protein with DIL domain, human RAD	-0.6170599	6.94182642	2.84E-07	1.41E-06
SPAC14C4.08	mug5	dynactin complex subunit Mug5	-0.617086	4.45089418	0.01759214	0.035014489
SPAC17C9.16c	mfs1	plasma membrane spermidine transmembrane transporter	-0.6171651	8.78672909	2.60E-16	3.52E-15
SPCC1919.10c	myo52	myosin type V	-0.6178318	8.29928444	2.92E-12	2.74E-11
SPCC594.04c	SPCC594.04c	steroid oxidoreductase superfamily protein (predicted)	-0.6180591	6.3866299	1.85E-05	6.97E-05
SPCPB1C11.02	SPCPB1C11.02	amino acid transmembrane transporter (predicted)	-0.6184034	7.73777702	1.15E-10	8.92E-10
SPNCRNA.384	#N/A	#N/A	-0.6186343	3.31659885	0.11538949	0.177171808
SPCC18B5.09c	SPCC18B5.09c	Schizosaccharomyces specific protein	-0.6196319	5.68993446	0.00903157	0.019206196
SPAC1D4.14	tho2	THO complex subunit Tho2 (predicted)	-0.6196692	8.97226385	8.34E-17	1.17E-15
SPAP27G11.05c	vps41	HOPS complex subunit Vps41 (predicted)	-0.6201626	7.37738436	4.68E-10	3.40E-09
SPCC965.08c	alr1	alanine racemase Alr1	-0.6213789	6.11504774	0.00017253	0.000545317
SPBC12C2.01c	SPBC12C2.01c	Schizosaccharomyces specific protein	-0.621589	6.32817999	0.00095871	0.002584781
SPNCRNA.903	#N/A	#N/A	-0.6220122	2.9474472	0.25011074	0.339992724
SPCC594.05c	spf1	Set1C PHD Finger protein Spf1	-0.622171	6.10294021	0.00025166	0.000774204
SPCP31B10.03c	med31	mediator complex subunit Med31	-0.6229335	5.97415258	0.00088461	0.002405977
SPAC23H3.03c	npr2	SEA/Iml1/Npr2/3 complex subunit Npr2	-0.6229459	6.524568	7.57E-05	0.000254441
SPBC9B6.02c	Tf2-9	retrotransposable element/transposon Tf2-type	-0.622948	7.9508481	9.51E-09	5.81E-08
SPBC16A3.02c	SPBC16A3.02c	mitochondrial CH-OH group oxidoreductase, human RTN4IP	-0.6234835	8.76290714	7.27E-13	7.30E-12
SPAC26A3.13c	Tf2-4	retrotransposable element/transposon Tf2-type	-0.6236237	8.13250429	1.86E-09	1.25E-08
SPNCRNA.1387	#N/A	#N/A	-0.6247738	5.70697177	0.0049796	0.011306688
SPAC22F3.08c	rok1	ATP-dependent RNA helicase Rok1 (predicted)	-0.625117	6.0948312	8.78E-06	3.51E-05
SPBC6B1.05c	atg7	E1 Atg8 and Atg12 E1 activating enzyme Atg7	-0.6265054	6.75210716	1.08E-06	4.97E-06
SPBC1778.02	rap1	shelterin complex telomere binding subunit Rap1	-0.6272425	5.94257033	5.10E-05	0.000177324
SPACUNK4.20	#N/A	#N/A	-0.628182	7.48638281	2.61E-11	2.21E-10
SPAC16E8.12c	png3	ING family homolog Png3 (predicted)	-0.6297285	6.37256028	2.86E-06	1.24E-05
SPAC23H4.14	vam6	guanyl-nucleotide exchange factor, HOPS complex subunit \	-0.6299937	6.92857944	2.71E-06	1.18E-05
SPBC18H10.10c	saf4	splicing associated factor Saf4	-0.6309879	6.28207674	1.69E-05	6.42E-05
SPBC543.08	SPBC543.08	phosphoinositide biosynthesis protein (predicted)	-0.6316155	6.33003754	2.89E-05	0.000105079
SPBC15D4.12c	mug98	Schizosaccharomyces specific protein Mug98	-0.6329112	3.93905643	0.06616709	0.110009052
SPNCRNA.1013	#N/A	#N/A	-0.6336748	5.83820984	0.00028386	0.000863132
SPBC365.13c	hba1	Ran GTPase binding protein Hba1	-0.6336959	7.40370694	7.17E-10	5.08E-09
SPAC16A10.01	SPAC16A10.01	plasma membrane ThrE amino acid transmembrane transp	-0.6338951	6.26197857	8.23E-06	3.32E-05
SPCC1442.04c	SPCC1442.04c	meiotic recombination protein (predicted)	-0.6339495	7.74504392	8.27E-13	8.27E-12
SPNCRNA.1167	#N/A	#N/A	-0.6352972	5.3970898	0.0005498	0.001572015
SPAC1F3.01	rrp6	exosome 3'-5' exoribonuclease subunit Rrp6	-0.6360921	7.83285186	7.85E-09	4.88E-08
SPAC750.05c	ftm4	sub-telomeric 5Tm protein family Ftm4	-0.6366078	7.26797792	4.02E-07	1.96E-06
SPNCRNA.1362	#N/A	#N/A	-0.6367742	6.28747667	0.00038292	0.00113542
SPNCRNA.857	#N/A	#N/A	-0.6368637	7.42061916	1.31E-07	6.80E-07
SPAC27D7.11c	SPAC27D7.11c	But2 family protein, similar to cell surface molecules	-0.6369723	7.73755486	1.37E-05	5.31E-05
SPBP18G5.02	pgs1	CDP-diacylglycerol-glycerol-3-phosphate3-phosphatidyltrans	-0.6370147	6.52085876	2.71E-05	9.92E-05
SPAC823.05c	tlg2	SNARE Tlg2 (predicted)	-0.637267	6.69714019	2.87E-07	1.42E-06
SPNCRNA.1581	#N/A	#N/A	-0.6377023	3.46307741	0.17593538	0.252630021

SPNCRNA.412	#N/A	#N/A	-0.6377023	3.46307741	0.17593538	0.252630021
SPBC13E7.05	gpi14	glycosylphosphatidylinositol-mannosyltransferase I complex	-0.6382398	5.71747948	0.00026104	0.000799804
SPNCRNA.1268	#N/A	#N/A	-0.638771	4.20540389	0.03646518	0.06599615
SPNCRNA.1291	#N/A	#N/A	-0.6389553	4.71033989	0.00555684	0.012475844
SPNCRNA.1287	#N/A	#N/A	-0.6391102	1.94720645	0.35277823	0.448382828
SPBC25B2.11	pof2	F-box protein Pof2	-0.6393841	6.74654214	4.83E-08	2.69E-07
SPAC6G9.13c	bqt1	bouquet formation protein Bqt1	-0.6395206	4.71446216	0.00767679	0.016621249
SPCC622.21	wtf12	wtf element Wtf12	-0.640082	4.63188635	0.01893446	0.037445221
SPBC14C8.11c	SPBC14C8.11c	Schizosaccharomyces pombe specific protein	-0.6412504	5.13052929	0.00397417	0.009239571
SPNCRNA.1394	#N/A	#N/A	-0.6417308	5.08426597	0.00154008	0.003959282
SPAC11D3.09	SPAC11D3.09	agmatinase (predicted)	-0.6419175	6.82523893	3.49E-06	1.50E-05
SPAC22G7.08	ppk8	serine/threonine protein kinase Ppk8 (predicted)	-0.6419987	6.46605567	2.76E-07	1.37E-06
SPBC2D10.17	clr1	SHREC complex intermodule linker subunit Clr1	-0.6422644	7.72866814	1.42E-10	1.09E-09
SPAC13C5.07	mre11	Mre11 nuclease	-0.6423871	8.00181904	8.33E-15	9.81E-14
SPAC1834.07	klp3	kinesin-like protein Klp3	-0.643873	6.3026523	1.73E-06	7.73E-06
SPAC27D7.12c	but1	thioredoxin family protein But1	-0.6438768	4.36493366	0.01447081	0.029381179
SPAC227.19c	SPAC227.19c	phosphatidylinositol N-acetylglucosaminyltransferase subu	-0.6455078	4.25106184	0.16860209	0.243958586
SPNCRNA.1679	#N/A	#N/A	-0.6460352	4.04389621	0.07733715	0.125648744
SPNCRNA.996	#N/A	#N/A	-0.6464329	6.61768042	1.60E-07	8.22E-07
SPAC9E9.14	vps24	ESCRT III complex subunit Vps24	-0.6467805	6.21876493	4.12E-05	0.000145313
SPAC1D4.10	trz1	nuclear 3'-tRNA processing endonuclease tRNAse Z, Trz1	-0.6470053	7.15554633	1.12E-08	6.81E-08
SPCC777.03c	SPCC777.03c	nifs homolog, possible cysteine desulfurase	-0.6471509	6.4756176	5.80E-06	2.40E-05
SPBP35G2.17	#N/A	#N/A	-0.6474018	5.91174693	2.05E-05	7.66E-05
SPAC458.03	tel2	Tel2/Rad-5/Clk-2 family protein Tel2	-0.6476276	6.95810419	4.13E-08	2.33E-07
SPAC7D4.04	atg11	autophagy associated protein Atg11	-0.6484115	8.52924463	3.42E-17	5.02E-16
SPNCRNA.1044	#N/A	#N/A	-0.6484753	5.28068781	0.00116184	0.003070411
SPAC5D6.04	SPAC5D6.04	auxin family transmembrane transporter (predicted)	-0.6488127	8.19504344	4.56E-15	5.49E-14
SPAC13C5.03	tht1	nuclear membrane protein involved in karyogamy Tht1	-0.6494783	5.7618359	0.00031589	0.000953698
SPAC144.12	rki1	ribose 5-phosphate isomerase Rki1 (predicted)	-0.6494886	7.82160117	1.04E-10	8.12E-10
SPAC1002.06c	bqt2	bouquet formation protein Bqt2	-0.6518711	2.3003563	0.31522166	0.409210868
SPBC25H2.08c	mrs2	mitochondrial inner membrane magnesium ion transmemb	-0.6529578	5.66814984	0.00044614	0.001306346
SPCC1919.12c	erm2	multi-spanning vacuolar membrane protease (predicted)	-0.6530689	8.33964704	1.19E-11	1.05E-10
SPAC57A10.08c	SPAC57A10.08c	lipid particle hydrolase (predicted)	-0.6538386	5.83013823	0.00015755	0.000502871
SPAC1952.17c	tbc13	GTPase activating protein, involved in vesicle-mediated tra	-0.6541751	6.58466291	4.90E-06	2.05E-05
SPNCRNA.519	#N/A	#N/A	-0.654591	4.63118422	0.00573618	0.01286152
SPAC27E2.09	mak2	histidine kinase Mak2	-0.655202	8.8870319	2.26E-15	2.80E-14
SPAC7D4.13c	SPAC7D4.13c	Schizosaccharomyces specific protein	-0.6554032	5.79588675	0.0002367	0.000732479
SPBC1685.05	htr11	serine protease (predicted)	-0.6555003	7.44341976	8.41E-10	5.92E-09
SPBC19F5.01c	puc1	cyclin Puc1	-0.6559093	6.43395655	5.88E-05	0.000201886
SPAC10F6.12c	mam4	protein-S isoprenylcysteine O-methyltransferase Mam4	-0.6570124	6.71307908	0.0007756	0.002141158
SPCC16A11.04	snx12	ER-vacuole tethering sorting nexin Snx12 (predicted)	-0.6570253	7.65389939	6.89E-08	3.73E-07
SPNCRNA.1516	#N/A	#N/A	-0.6589701	4.20387837	0.03577229	0.064914785
SPCC18B5.11c	cds1	replication checkpoint kinase Cds1	-0.6598177	7.47965886	2.16E-07	1.08E-06
SPCC830.09c	pop5	RNase P and RNase MRP subunit Pop5	-0.660016	5.99883379	0.00063344	0.001788608
SPNCRNA.427	#N/A	#N/A	-0.6611994	2.13402	0.35384029	0.449419173
SPNCRNA.190	#N/A	#N/A	-0.6612488	2.77875722	0.28385898	0.376762309
SPNCRNA.1461	#N/A	#N/A	-0.6614692	6.45622999	2.59E-07	1.29E-06

SPAC25H1.02	jmj1	histone demethylase Jmj1 (predicted)	-0.6619137	5.04617126	0.00534463	0.012039122
SPAC4F10.08	mug126	Schizosaccharomyces pombe specific protein	-0.6622897	5.09315457	0.01403693	0.028607765
SPAC167.08	Tf2-2	retrotransposable element/transposon Tf2-type	-0.6627366	9.23315758	2.55E-12	2.41E-11
SPNCRNA.751	#N/A	#N/A	-0.6636267	2.63512196	0.23845952	0.326633865
SPBC1105.12	hhf3	histone H4 h4.3	-0.6643656	7.8668804	3.01E-13	3.14E-12
SPNCRNA.1248	#N/A	#N/A	-0.6646687	3.00521569	0.20275003	0.283774982
SPNCRNA.995	#N/A	#N/A	-0.6654188	6.62120635	8.67E-08	4.64E-07
SPNCRNA.561	#N/A	#N/A	-0.6655302	5.74392969	7.54E-05	0.000253653
SPNCRNA.1481	#N/A	#N/A	-0.665686	4.44379088	0.01163774	0.024166866
SPCC63.04	mok14	alpha-1,4-glucan synthase Mok14	-0.66592	7.44675111	8.58E-09	5.30E-08
SPBC660.05	SPBC660.05	WW domain containing conserved fungal protein	-0.6678054	5.24897054	0.00068228	0.001917756
SPNCRNA.647	#N/A	#N/A	-0.6707495	6.96375839	6.13E-09	3.86E-08
SPAC8E11.12	SPAC8E11.12	Schizosaccharomyces pombe specific protein	-0.6710906	2.58396417	0.27298764	0.364670456
SPAC29B12.03	spd1	ribonucleotide reductase (RNR) inhibitor	-0.671112	6.86935289	4.63E-09	2.96E-08
SPNCRNA.1274	#N/A	#N/A	-0.6716189	7.38889057	4.45E-11	3.64E-10
SPNCRNA.584	#N/A	#N/A	-0.6728106	7.38291431	4.12E-11	3.40E-10
SPNCRNA.1285	#N/A	#N/A	-0.6738141	5.90713856	5.96E-06	2.46E-05
SPBC18E5.03c	sim4	CENP-K ortholog Sim4	-0.6744187	5.47847709	0.00013533	0.000436879
SPAC24C9.11	sgd1	ribosome small subunit biogenesis protein Sgd1 (predicted)	-0.6755234	7.2354539	8.62E-09	5.32E-08
SPAC17A2.06c	vps8	CORVET complex WD repeat/ ubiquitin-protein ligase E3 su	-0.6756375	8.10249427	6.62E-11	5.26E-10
SPCC297.04c	set7	histone lysine methyltransferase Set7 (predicted)	-0.6767454	6.4266526	6.13E-06	2.53E-05
SPNCRNA.253	#N/A	#N/A	-0.6773266	2.23624907	0.29432647	0.387032196
SPNCRNA.833	#N/A	#N/A	-0.6776873	6.84963132	8.90E-08	4.74E-07
SPNCRNA.1283	#N/A	#N/A	-0.6780578	5.02785194	0.00060046	0.001701123
SPCPJ732.03	meu15	Schizosaccharomyces pombe specific protein Meu15	-0.6785087	1.86477774	0.33312797	0.427481775
SPNCRNA.931	#N/A	#N/A	-0.6785773	5.7559139	3.63E-05	0.000129199
SPBC577.13	syj2	inositol-polyphosphate 5-phosphatase, synaptojanin homol	-0.6787885	7.53570063	1.15E-12	1.13E-11
SPBC18H10.05	SPBC18H10.05	WD repeat protein, human WDR44 family	-0.6788887	6.70808544	8.07E-08	4.33E-07
SPCC757.02c	SPCC757.02c	dehydrogenase (predicted)	-0.6797738	6.30031247	2.13E-05	7.93E-05
SPNCRNA.773	#N/A	#N/A	-0.6797771	6.60508743	6.84E-07	3.23E-06
SPBC1683.01	SPBC1683.01	inorganic phosphate transmembrane transporter (predictec	-0.680832	7.66172256	1.41E-10	1.09E-09
SPAC4D7.14	new13	conserved fungal protein of unknown function	-0.6812431	5.30990527	0.00107951	0.002876301
SPBP22H7.02c	mrd1	ribosome biogenesis RNA-binding protein Mrd1 (predicted)	-0.6819439	6.33588758	8.80E-06	3.51E-05
SPBC1718.06	msp1	mitochondrial dynamin family fusion GTPase Msp1	-0.6826095	8.89839497	8.59E-21	1.80E-19
SPNCRNA.506	#N/A	#N/A	-0.6843972	1.23599102	0.76938276	0.828687822
SPAC23D3.13c	SPAC23D3.13c	guanyl-nucleotide exchange factor (predicted)	-0.6849427	8.57717436	1.95E-18	3.19E-17
SPCC965.05c	thp1	uracil DNA N-glycosylase Thp1	-0.6849591	6.71104308	3.00E-09	1.95E-08
SPAC15E1.02c	SPAC15E1.02c	DUF1761 family protein	-0.6854654	7.34574445	1.49E-11	1.30E-10
SPAC27E2.08	Tf2-6	retrotransposable element/transposon Tf2-type	-0.6855052	8.77867297	8.66E-13	8.62E-12
SPAC10F6.09c	psm3	mitotic cohesin complex ATPase subunit Psm3/Smc3	-0.6856218	8.42149622	7.66E-11	6.03E-10
SPNCRNA.725	#N/A	#N/A	-0.6856448	8.42373868	8.10E-11	6.35E-10
SPBC11C11.11c	irc3	mitochondrial DNA branch migration helicase Irc3 (predicte	-0.686403	7.01895775	3.20E-08	1.84E-07
SPAC4A8.04	isp6	vacuolar serine protease Isp6	-0.6869763	9.28395729	1.08E-12	1.07E-11
SPNCRNA.987	#N/A	#N/A	-0.6872656	3.31709354	0.08818333	0.141130657
SPAC1687.14c	SPAC1687.14c	EF hand family protein, centrin-like	-0.6876215	5.47315029	0.00207538	0.00518084
SPAC6F12.02	rst2	transcription factor Rst2	-0.6887258	7.86106749	4.83E-15	5.80E-14
SPAC27D7.03c	mei2	RNA-binding protein involved in meiosis Mei2	-0.6892267	8.67137072	2.22E-07	1.12E-06

SPAC30.02c	kti12	elongator complex associated protein Kti12 (predicted)	-0.6907131	5.86130298	0.00017228	0.000544782
SPAPB24D3.04c	mag1	DNA-3-methyladenine glycosylase Mag1	-0.6912001	5.60850083	6.05E-05	0.000207065
SPNCRNA.379	#N/A	#N/A	-0.6914805	1.23596255	0.76509305	0.825243017
SPAC607.08c	mil1	lipase/AP-1 adaptor complex binding protein Mil1 (predicte	-0.6929824	6.70754047	6.15E-08	3.36E-07
SPNCRNA.68	#N/A	#N/A	-0.6931304	1.23627586	0.76264901	0.822955581
SPAC1006.04c	mcp3	Schizosaccharomyces specific protein Mcp3	-0.6943372	5.3994089	0.00045024	0.001315532
SPAC110.01	ppk1	serine/threonine protein kinase Ppk1 (predicted)	-0.694794	7.11413621	1.16E-07	6.04E-07
SPAC16E8.08	pos1	Spa2 interacting protein Pos1	-0.6954059	3.97835671	0.02033354	0.039727171
SPNCRNA.1427	#N/A	#N/A	-0.6958033	5.96541383	0.00010132	0.000334199
SPAC3A11.14c	pkl1	kinesin-14 family minus-end directed microtubule motor Pk	-0.6961085	7.14488873	1.14E-10	8.83E-10
SPAC16A10.02	sub1	transcription coactivator PC4	-0.6963432	7.67333288	7.71E-12	6.94E-11
SPNCRNA.1368	#N/A	#N/A	-0.6966477	6.4647392	1.16E-07	6.04E-07
SPCC338.03c	#N/A	#N/A	-0.6968925	4.94522357	0.00465387	0.010673946
SPNCRNA.1008	#N/A	#N/A	-0.6971133	5.6589456	0.00015372	0.00049158
SPAC22A12.01c	pso2	DNA 5' exonuclease (predicted)	-0.6989935	6.79796432	2.37E-09	1.56E-08
SPNCRNA.1689	#N/A	#N/A	-0.7002347	4.17901561	0.01485141	0.03008618
SPNCRNA.70	#N/A	#N/A	-0.7004943	1.23658302	0.76608323	0.826049184
SPAC607.07c	SPAC607.07c	Schizosaccharomyces specific protein	-0.7024697	6.64463076	7.97E-09	4.94E-08
SPBC3B9.06c	atg3	autophagy associated protein Atg3	-0.7036645	6.16916228	1.74E-06	7.79E-06
SPBC582.06c	mcp6	horsetail movement protein Hrs1/Mcp6	-0.7037671	4.04513168	0.03702945	0.066821901
SPBC16C6.14	spo2	sporulation specific protein Spo2	-0.7045751	6.64919299	6.69E-08	3.64E-07
SPBC29A10.10c	dbl8	ATP-dependent RNA helicase Dbl8	-0.7047544	7.82774571	3.23E-13	3.35E-12
SPBC16C6.01c	SPBC16C6.01c	lysine methyltransferase, human SETD6 ortholog (predictec	-0.7052682	5.82822704	1.65E-05	6.28E-05
SPNCRNA.1458	#N/A	#N/A	-0.7055172	5.34365252	0.01545942	0.031152307
SPAC22G7.11c	cum1	Con-6 family conserved fungal protein	-0.7064823	7.04672835	8.50E-09	5.25E-08
SPNCRNA.1284	#N/A	#N/A	-0.7064894	3.34824734	0.17836654	0.255533539
SPAC3H1.14	gid4	GID complex subunit Gid4 (predicted)	-0.707181	5.32070143	0.00354167	0.008351022
SPAC9.04	Tf2-1	retrotransposable element/transposon Tf2-type	-0.7085135	8.80954668	4.54E-14	5.00E-13
SPNCRNA.1572	#N/A	#N/A	-0.7092634	5.08935404	0.00080746	0.002217417
SPAC823.16c	atg1802	autophagy associated WD repeat protein Atg18b	-0.7095731	6.46311283	3.48E-08	1.99E-07
SPAC17H9.01	cid16	poly(A/U) polymerase Cid16 (predicted)	-0.7114567	7.75647024	1.02E-12	1.01E-11
SPCC569.04	SPCC569.04	Schizosaccharomyces pombe specific protein	-0.7118646	3.36274531	0.07068237	0.116210074
SPBC27.03	meu25	Schizosaccharomyces specific protein Meu25	-0.7120884	5.72845725	1.80E-05	6.79E-05
SPAC22H10.10	alp21	tubulin specific chaperone cofactor E	-0.7122183	5.24209176	0.00060008	0.001701123
SPBC1718.01	pop1	cullin 1 adaptor protein Pop1	-0.7153876	7.11084974	1.22E-08	7.33E-08
SPNCRNA.1611	#N/A	#N/A	-0.718359	7.90791711	9.95E-16	1.28E-14
SPAC1002.10c	sgt1	SGT1 family transcriptional regulator Sgt1	-0.7184166	6.34600709	7.11E-08	3.84E-07
SPNCRNA.738	#N/A	#N/A	-0.7186468	4.4950225	0.01186371	0.024568629
SPBC4C3.08	otg2	alpha-1,3-galactosyltransferase	-0.721064	6.45458362	6.35E-06	2.61E-05
SPAC1002.20	SPAC1002.20	Schizosaccharomyces pombe specific protein	-0.7226351	3.63626026	0.03660649	0.066216686
SPCC18B5.03	wee1	M phase inhibitor protein kinase Wee1	-0.7233258	7.66617484	4.36E-13	4.46E-12
SPBC1703.04	mlh1	MutL family protein Mlh1 (predicted)	-0.7236796	6.25084055	4.69E-07	2.27E-06
SPCC417.04	#N/A	#N/A	-0.7237123	4.77471419	0.0019996	0.00501742
SPNCRNA.1579	#N/A	#N/A	-0.7243162	5.52447368	4.95E-05	0.000172355
SPAC22G7.07c	ime4	mRNA (N6-adenosine)-methyltransferase Ime4 (predicted)	-0.7258771	6.62935592	7.94E-05	0.000265617
ScpofMr11	#N/A	#N/A	-0.726162	11.1240026	1.13E-15	1.44E-14
SPNCRNA.1443	#N/A	#N/A	-0.7262687	7.11052639	1.68E-09	1.14E-08

SPBC776.05	gpc1	glycerophosphocholine acyltransferase (GPCAT) Gpc1 (pred	-0.7263994	6.15507028	7.58E-06	3.07E-05
SPBC16D10.04c	dna2	DNA replication endonuclease-helicase Dna2	-0.7267177	7.77980451	1.50E-13	1.59E-12
SPNCRNA.1509	#N/A	#N/A	-0.7276951	4.56614339	0.00918947	0.019511503
SPAC2G11.09	SPAC2G11.09	calcium ion transmembrane transporter (predicted)	-0.7280777	7.44158421	1.41E-11	1.23E-10
SPAC9G1.02	wis4	MAP kinase kinase kinase Wis4	-0.728417	8.07213243	1.34E-16	1.85E-15
SPBC1289.17	Tf2-11	retrotransposable element/transposon Tf2-type	-0.7291129	8.69123876	6.45E-15	7.66E-14
SPNCRNA.944	#N/A	#N/A	-0.7294086	3.5841637	0.05074533	0.087801159
SPBC1105.19	tam12	Schizosaccharomyces specific protein Tam12	-0.7322147	4.75151223	0.00094561	0.00255657
SPNCRNA.124	#N/A	#N/A	-0.7323669	1.53206359	0.50511003	0.598515372
SPAC4G8.09	SPAC4G8.09	mitochondrial leucine-tRNA ligase (predicted)	-0.7323845	7.44762085	1.25E-14	1.43E-13
SPAC22H10.13	zym1	metallothionein Zym1	-0.7325504	5.68104898	3.19E-05	0.000114782
SPNCRNA.257	#N/A	#N/A	-0.7327187	3.08235137	0.0944101	0.149548589
SPBC1198.06c	SPBC1198.06c	mannan endo-1,6-alpha-mannosidase (predicted)	-0.7327429	7.87373992	3.98E-17	5.76E-16
SPNCRNA.1315	#N/A	#N/A	-0.7346376	2.1695998	0.29035565	0.382920587
SPNCRNA.683	#N/A	#N/A	-0.7347724	7.36115944	1.43E-14	1.63E-13
SPAC29A4.11	rga3	RhoGAP, GTPase activating protein Rga3	-0.7352559	9.11044293	2.19E-17	3.24E-16
SPNCRNA.1580	#N/A	#N/A	-0.736815	6.43725839	4.19E-06	1.77E-05
ScpofMr12	#N/A	#N/A	-0.7382067	9.9562044	6.64E-16	8.66E-15
SPNCRNA.1493	#N/A	#N/A	-0.7388416	2.16869456	0.29747945	0.390423843
SPAC24B11.14	#N/A	#N/A	-0.7390599	3.82159942	0.02580302	0.048891616
SPBC27.05	#N/A	#N/A	-0.7391673	3.96739395	0.05591137	0.095290187
SPBPB2B2.02	say1	ER sterol deacetylase Say1 (predicted)	-0.7401031	4.98781527	0.00628125	0.013927636
SPCC1739.09c	cox13	cytochrome c oxidase subunit VIa (predicted)	-0.7404279	6.33914901	1.65E-05	6.30E-05
SPAC3A12.09c	ure4	urease accessory protein UreD	-0.7406284	5.55054237	1.53E-05	5.85E-05
SPAC1782.03	saf3	splicing associated factor Saf3	-0.7411102	5.98731698	1.07E-05	4.21E-05
SPAC1F3.06c	spo15	mitotic and mieotic spindle pole body protein Spo15	-0.7421235	9.27309938	1.78E-15	2.23E-14
SPNCRNA.1571	#N/A	#N/A	-0.7422376	5.02852948	0.00070278	0.001969682
SPNCRNA.813	#N/A	#N/A	-0.7427787	3.11855707	0.16789452	0.243089831
SPBC543.10	get1	GET complex (ER membrane insertion) subunit Get1 (predi	-0.7436309	6.65955936	1.66E-09	1.12E-08
SPAC1565.07c	knd1	Cullin-associated NEDD8-dissociated protein Knd1 (predicte	-0.7441182	7.16658283	1.39E-10	1.07E-09
SPBC1D7.01	gim6	prefoldin subunit 1 (predicted)	-0.7447575	6.65171061	4.48E-09	2.87E-08
SPAC11E3.05	sea3	SEA complex ubiquitin-protein ligase E3 subunit Sea3 (pred	-0.744898	7.42080315	1.38E-13	1.46E-12
SPAC20G4.02c	fus1	formin Fus1	-0.745932	7.339124	7.34E-13	7.35E-12
SPAP27G11.14c	SPAP27G11.14c	Schizosaccharomyces pombe specific protein	-0.7459466	6.45831681	8.89E-06	3.55E-05
SPAC13F5.05	mpd1	thioredoxin family protein Mpd1 (predicted)	-0.7464114	5.52733906	0.00025928	0.000795216
SPNCRNA.1153	#N/A	#N/A	-0.7464895	6.48126829	2.70E-06	1.18E-05
SPBC947.15c	nde1	mitochondrial NADH dehydrogenase (ubiquinone) Nde1 (pre	-0.748537	8.85276606	3.22E-13	3.35E-12
SPNCRNA.529	#N/A	#N/A	-0.7485651	3.13702194	0.12155526	0.185374252
SPNCRNA.1155	#N/A	#N/A	-0.7492713	6.69264154	6.47E-07	3.07E-06
SPCC191.01	SPCC191.01	Schizosaccharomyces specific protein	-0.7498271	8.04949723	5.35E-15	6.39E-14
SPBC29A3.03c	gid2	GID complex ubiquitin-protein ligase E3 subunit Gid2/Rmd5	-0.7500306	6.80973163	1.19E-10	9.21E-10
SPNCRNA.1164	#N/A	#N/A	-0.7502677	4.45880153	0.00486461	0.011101166
SPAC977.01	ftm1	sub-telomeric 5Tm protein family Ftm1	-0.75152	7.02986928	1.87E-05	7.05E-05
SPAC6B12.02c	mus7	DNA repair protein Mus7/Mms22	-0.7519852	8.00334821	6.18E-19	1.05E-17
SPNCRNA.35	#N/A	#N/A	-0.7550869	3.68558533	0.0224343	0.043333919
SPBC9B6.03	SPBC9B6.03	zf-FYVE type zinc finger protein, localizations and processes	-0.7556124	6.9675624	4.59E-11	3.76E-10
SPBC530.07c	SPBC530.07c	TENA/THI family protein, domain found in context with a br	-0.7558726	5.27889941	0.00013766	0.000443563

SPNCRNA.1662	#N/A	#N/A	-0.7564215	6.12350978	6.24E-07	2.97E-06
SPNCRNA.229	#N/A	#N/A	-0.7567493	3.18613821	0.08717837	0.139587952
SPCC645.05c	myo2	myosin II heavy chain	-0.7570197	8.35054459	2.39E-15	2.96E-14
SPCC970.11c	wtf9	wtf element Wtf9	-0.7583691	5.70998197	2.69E-06	1.18E-05
SPNCRNA.1336	#N/A	#N/A	-0.7602044	4.59823748	0.00950038	0.020115138
SPBC14F5.01	SPBC14F5.01	DUF4504 family protein, human C1orf74 ortholog	-0.7603926	5.6502644	1.09E-05	4.28E-05
SPAC1399.06	SPAC1399.06	Schizosaccharomyces pombe specific protein	-0.7617556	4.1773784	0.01788003	0.035525143
SPNCRNA.992	#N/A	#N/A	-0.7623696	8.12390158	3.14E-14	3.50E-13
SPNCRNA.1441	#N/A	#N/A	-0.7626313	2.68498898	0.13463992	0.201989558
SPNCRNA.1446	#N/A	#N/A	-0.7628874	5.74326972	4.02E-06	1.71E-05
SPNCRNA.1191	#N/A	#N/A	-0.7645437	6.08226839	1.22E-07	6.37E-07
SPCC1322.08	srk1	MAPK-activated protein kinase Srk1	-0.7646109	8.0611402	3.25E-08	1.87E-07
SPAC29B12.14c	SPAC29B12.14c	plasma membrane purine transmembrane transporter (pre	-0.7654407	7.49519659	3.65E-13	3.76E-12
SPAP8A3.14c	sls1	mitochondrial inner membrane protein Sls1 (predicted)	-0.7679029	6.42338602	4.38E-08	2.46E-07
SPNCRNA.186	#N/A	#N/A	-0.7685874	4.48488356	0.01145404	0.023814472
SPNCRNA.887	#N/A	#N/A	-0.7693407	5.98519634	1.29E-05	5.01E-05
SPBC1604.01	egt1	ergothioneine biosynthesis protein Egt1	-0.770232	8.22803319	6.06E-14	6.59E-13
SPNCRNA.93	#N/A	#N/A	-0.7702454	2.86380529	0.1375561	0.205684307
SPBC1683.07	mal1	maltase alpha-glucosidase Mal1	-0.7706727	9.17484701	1.01E-25	2.70E-24
ScpofMp08	#N/A	#N/A	-0.7722232	2.09883468	0.25931681	0.349782135
SPACUNK4.08	dpp2	dipeptidyl peptidase (predicted)	-0.7744703	8.22454586	1.23E-18	2.06E-17
SPCC1827.08c	pof7	F-box protein Pof7	-0.7761543	6.30100336	2.51E-07	1.26E-06
SPCC1322.07c	mug150	Schizosaccharomyces pombe specific protein Mug150	-0.7771851	6.15498266	3.09E-06	1.34E-05
SPAC688.06c	slx4	structure-specific endonuclease subunit Slx4	-0.7772996	6.00226838	1.08E-05	4.22E-05
SPCC320.06	SPCC320.06	conserved fungal protein	-0.7787783	7.89184412	3.57E-17	5.18E-16
SPNCRNA.1677	#N/A	#N/A	-0.7800363	5.07970463	0.00324364	0.007731342
SPNCRNA.581	#N/A	#N/A	-0.7809515	5.96658161	2.63E-06	1.15E-05
SPBC609.04	caf5	plasma membrane spermine family transmembrane transp	-0.7846396	6.70177537	1.92E-10	1.45E-09
SPNCRNA.1396	#N/A	#N/A	-0.7866469	6.18531901	5.16E-08	2.86E-07
SPNCRNA.700	#N/A	#N/A	-0.7867547	6.88568833	4.21E-11	3.46E-10
SPAC4D7.11	dsc4	Golgi Dsc E3 ligase complex subunit Dsc4	-0.7902837	6.96264003	2.31E-11	1.98E-10
SPNCRNA.873	#N/A	#N/A	-0.7911779	6.32099893	3.16E-06	1.37E-05
SPCPB16A4.07	SPCPB16A4.07	Schizosaccharomyces specific protein	-0.7914242	6.36936156	5.15E-08	2.85E-07
SPAC3F10.19	spd2	ribonucleotide reductase (RNR) inhibitor family	-0.7918476	5.53583321	5.52E-05	0.000190294
SPNCRNA.1623	#N/A	#N/A	-0.7923916	4.42331217	0.00707722	0.015460825
SPNCRNA.133	#N/A	#N/A	-0.7930439	7.85361068	3.44E-10	2.55E-09
SPNCRNA.960	#N/A	#N/A	-0.7965314	6.58244312	1.06E-09	7.41E-09
SPAC227.13c	isu1	mitochondrial [2Fe-2S] cluster assembly protein Isu1	-0.7968114	6.8870378	1.43E-06	6.45E-06
SPCC1281.04	akr7	pyridoxal reductase (predicted)	-0.7979266	4.92612992	0.00129264	0.003384548
SPCC24B10.03	SPCC24B10.03	Schizosaccharomyces specific protein	-0.7986297	4.9392046	0.0001962	0.000615827
SPNCRNA.1355	#N/A	#N/A	-0.7994435	2.58358749	0.15596003	0.228385621
SPAC27D7.13c	ssm4	dynactin microtubule-binding subunit, p150-Glued Ssm4	-0.7996113	5.41690224	0.0002867	0.000870981
SPAC16A10.03c	SPAC16A10.03c	ubiquitin-protein ligase E3 involved in vesicle docking Pep5/	-0.7996577	7.94774125	1.24E-16	1.72E-15
SPCC550.12	arp6	actin-like protein Arp6	-0.8005556	5.66907426	0.00016326	0.000519401
SPAC23H3.15c	ddr48	DNA damage-responsive protein ortholog DDr48	-0.8022267	8.99292302	1.11E-08	6.73E-08
SPBC20F10.10	psl1	cyclin pho85 family Psl1 (predicted)	-0.8023207	5.08846499	6.53E-05	0.000222412
SPBC27B12.02	mis19	centromere protein Mis19/Eic1	-0.8028087	5.15599731	0.00014469	0.000464443

SPNCRNA.832	#N/A	#N/A	-0.803443	8.99293836	1.12E-08	6.81E-08
SPBC3D6.16	#N/A	#N/A	-0.8054323	4.79162644	0.00056797	0.001619188
SPNCRNA.1585	#N/A	#N/A	-0.8065957	5.8434315	1.16E-06	5.32E-06
SPCC4B3.02c	got1	Golgi transport protein Got1 (predicted)	-0.80698	6.31448162	3.76E-06	1.60E-05
SPNCRNA.521	#N/A	#N/A	-0.8077459	3.4501927	0.02778889	0.052111993
SPBC887.18c	hfi1	SAGA complex subunit Hfi1/Ada1	-0.8083631	5.85112048	2.40E-06	1.06E-05
SPNCRNA.01	#N/A	#N/A	-0.8089067	6.54900057	1.86E-09	1.25E-08
SPNCRNA.517	#N/A	#N/A	-0.8089237	4.10081524	0.02640391	0.049910719
SPNCRNA.448	#N/A	#N/A	-0.8097048	5.09309445	0.00013881	0.000446911
SPNCRNA.651	#N/A	#N/A	-0.8101227	3.54770267	0.07380282	0.120727926
SPNCRNA.1071	#N/A	#N/A	-0.8119228	4.63103157	0.00069941	0.001961857
SPBC119.14	rti1	Rad22 homolog Rti1	-0.8122775	3.08232246	0.05573163	0.09513464
SPNCRNA.1168	#N/A	#N/A	-0.8129464	4.45864776	0.00361749	0.00849449
SPNCRNA.1317	#N/A	#N/A	-0.8133691	6.94899433	1.35E-12	1.33E-11
SPNCRNA.1282	#N/A	#N/A	-0.8135279	5.26144813	1.59E-05	6.07E-05
SPAC6G9.16c	xrc4	XRCC4 nonhomologous end joining factor Xrc4	-0.8137987	5.987214	2.45E-06	1.07E-05
SPAC22F3.11c	snu23	U4/U6 x U5 tri-snRNP complex subunit Snu23	-0.8147404	5.18912805	0.0001233	0.000401091
SPNCRNA.487	#N/A	#N/A	-0.8159927	3.62494567	0.02332221	0.044832671
SPAC22H12.05c	fsc1	fasciclin domain protein Fsc1	-0.8160868	7.49136971	6.39E-13	6.44E-12
SPAC17A2.07c	SPAC17A2.07c	Schizosaccharomyces specific protein	-0.8172121	5.34636935	0.00036065	0.001075478
SPAC20H4.11c	rho5	Rho family GTPase Rho5	-0.8173323	5.63327484	2.08E-05	7.74E-05
SPCC1682.06	SPCC1682.06	Schizosaccharomyces specific protein	-0.8175653	6.11210004	1.31E-07	6.80E-07
SPBC685.04c	aps2	AP-2 adaptor complex sigma subunit Aps2 (predicted)	-0.8184466	5.94106865	1.43E-06	6.47E-06
SPAC631.02	bdf2	BET family double bromodomain protein Bdf2	-0.8189392	8.65703769	1.30E-24	3.30E-23
SPAC56F8.13	#N/A	#N/A	-0.8194998	7.06515257	1.66E-13	1.75E-12
SPCC794.02	wtf5	wtf element Wtf5	-0.8204972	5.81693297	7.08E-07	3.34E-06
SPNCRNA.646	#N/A	#N/A	-0.8224865	5.66329731	6.65E-06	2.72E-05
SPCC1919.06c	wtf25	wtf element	-0.8228851	6.241025	9.48E-08	5.03E-07
SPNCRNA.966	#N/A	#N/A	-0.8235481	3.81118894	0.02060193	0.040159309
SPCC1906.04	wtf20	wtf element Wtf20	-0.8246711	4.53093041	0.00356206	0.008387479
SPAC19A8.08	upf2	nonsense-mediated decay protein Upf2	-0.8257329	7.38722549	8.95E-17	1.25E-15
SPCC645.12c	SPCC645.12c	Schizosaccharomyces specific protein	-0.8262508	4.90116274	0.00025267	0.000776621
SPCP25A2.03	tho1	THO complex subunit Tho1 (predicted)	-0.8270638	5.77691479	2.33E-06	1.03E-05
SPNCRNA.1294	#N/A	#N/A	-0.8273968	6.16032264	1.06E-07	5.60E-07
SPNCRNA.1696	#N/A	#N/A	-0.8304431	6.0097517	1.95E-06	8.67E-06
SPBC13A2.04c	ptr2	plasma membrane PTR family peptide transmembrane tra	-0.8304883	9.2235289	6.01E-19	1.03E-17
SPAC18G6.01c	SPAC18G6.01c	chalcone related protein family	-0.8314026	6.74492817	2.21E-08	1.28E-07
SPBC31E1.04	pep12	SNARE Pep12	-0.8317834	7.83483349	4.48E-20	8.71E-19
SPCC737.03c	ima1	inner nuclear membrane protein Ima1	-0.8318269	8.57190464	1.96E-24	4.93E-23
SPCC1620.01c	lsm2	Lsm2-8 complex subunit Lsm2	-0.8322579	5.2043742	0.00749797	0.016275503
SPCC825.04c	naa40	histone N-acetyltransferase Naa40 (predicted)	-0.8329259	4.80814976	0.00111051	0.002949664
SPAC2C4.17c	msy2	MS ion channel protein 2 (predicted)	-0.8333036	8.61945675	5.65E-26	1.54E-24
SPNCRNA.214	ter1	telomerase RNA	-0.8336677	7.89272794	6.47E-17	9.19E-16
SPNCRNA.882	#N/A	#N/A	-0.8340591	4.23123334	0.00516961	0.011691318
SPNCRNA.467	#N/A	#N/A	-0.8344467	1.58394586	0.54903511	0.639100912
SPNCRNA.884	#N/A	#N/A	-0.8347308	7.89339955	5.75E-17	8.21E-16
SPBC19F8.07	mcs6	TFIIH associated cyclin-dependent protein kinase Mcs6	-0.8353683	7.67420098	3.22E-19	5.68E-18

ScpofMp10	#N/A	#N/A	-0.8355017	9.20807824	1.88E-08	1.10E-07
SPNCRNA.658	#N/A	#N/A	-0.8360951	3.17320906	0.11623777	0.178353553
SPBC365.11	grp2	Golgi GRIP domain protein Grp2 (predicted)	-0.8400447	7.41212437	3.66E-13	3.77E-12
SPCC285.10c	ear1	SPRY domain membrane protein, specificity factor required	-0.842524	6.73463098	9.94E-11	7.75E-10
SPAC6F12.03c	fsv1	SNARE Fsv1	-0.8426549	6.67500662	1.50E-12	1.47E-11
SPNCRNA.155	#N/A	#N/A	-0.8438819	1.5836122	0.4956262	0.589742313
SPBC1604.09c	rex4	exoribonuclease Rex4 (predicted)	-0.8452495	5.6944475	2.00E-06	8.84E-06
SPNCRNA.417	#N/A	#N/A	-0.8454164	4.13025086	0.00898701	0.019129375
SPNCRNA.1092	#N/A	#N/A	-0.8458214	3.43639268	0.02046063	0.039929618
SPNCRNA.119	#N/A	#N/A	-0.8461589	3.27055077	0.03965289	0.07098655
SPBC21C3.02c	dep1	Sds3-like family protein Dep1	-0.8466417	4.19904492	0.00874745	0.018677873
SPBC2A9.05c	tvp23	Golgi transport protein Tvp23 (predicted)	-0.8493453	6.64198846	2.72E-11	2.29E-10
SPBC1677.02	dpm3	dolichol-phosphate mannosyltransferase subunit 3	-0.8502664	7.60252689	1.08E-19	2.03E-18
SPAC222.05c	mss1	mitochondrial tRNA wobble uridine modification GTPase M	-0.8513024	6.9457288	8.83E-14	9.47E-13
SPAC18B11.11	SPAC18B11.11	GTPase activating protein (predicted)	-0.8543156	7.42611388	1.42E-15	1.81E-14
SPNCRNA.1647	#N/A	#N/A	-0.8550776	5.70050971	1.28E-06	5.81E-06
SPNCRNA.1304	#N/A	#N/A	-0.8570091	6.66489626	4.42E-10	3.22E-09
SPAC25H1.04	mug105	K48-linkage specific deubiquitinase	-0.8576902	5.65336256	6.00E-07	2.86E-06
SPBC6B1.09c	nbs1	Mre11 complex subunit Nbs1	-0.8576995	6.49207485	6.72E-09	4.21E-08
SPBC25B2.02c	mam1	M-factor transmembrane transporter Mam1	-0.8613113	9.05153053	4.86E-26	1.34E-24
SPAC3C7.03c	rad55	RecA family ATPase Rad55/Rhp55	-0.8613247	6.2296618	2.81E-08	1.62E-07
SPAC57A7.05	SPAC57A7.05	transmembrane transporter (predicted)	-0.8648189	8.73992751	2.35E-20	4.68E-19
SPNCRNA.717	#N/A	#N/A	-0.8658291	2.60963414	0.10955982	0.169444224
SPCP1E11.07c	cwf18	complexed with Cdc5 protein Cwf18	-0.8667852	5.54361951	2.84E-05	0.00010329
SPAC3H8.04	SPAC3H8.04	DUF4210 domain protein, human FAM214A ortholog, impli	-0.867036	6.85553434	3.01E-13	3.14E-12
SPNCRNA.848	#N/A	#N/A	-0.8672337	5.97496271	5.70E-07	2.72E-06
SPAC18B11.09c	SPAC18B11.09c	serine O-acetyltransferase activity (predicted)	-0.8704309	6.64163103	6.61E-11	5.26E-10
SPNCRNA.760	#N/A	#N/A	-0.8709968	5.01340022	0.00077129	0.002131866
SPNCRNA.1257	#N/A	#N/A	-0.8711986	4.19622898	0.00388011	0.00903942
SPNCRNA.681	#N/A	#N/A	-0.8742809	4.95875786	9.26E-05	0.000306629
SPBC4F6.09	str1	plasma membrane siderophore-iron transmembrane transp	-0.8773734	7.38571471	3.10E-14	3.46E-13
SPNCRNA.58	#N/A	#N/A	-0.8782412	5.80224207	1.99E-05	7.46E-05
SPCC1672.14	#N/A	#N/A	-0.8783593	4.05648559	0.01081288	0.022626588
SPCC1827.07c	SPCC1827.07c	SPX/EXS domain protein (predicted)	-0.8795834	6.95941556	2.19E-11	1.88E-10
SPAPB17E12.09	SPAPB17E12.09	Schizosaccharomyces specific protein, expressed during me	-0.8804089	3.5207785	0.01394165	0.028430597
SPNCRNA.900	#N/A	#N/A	-0.8818091	3.08032279	0.05336019	0.091576586
SPBC15C4.06c	SPBC15C4.06c	ubiquitin-protein ligase E3 Meu34, human RNF13 family ho	-0.8818424	7.44703919	7.13E-17	1.01E-15
SPBC1A4.06c	tam41	mitochondrial phosphatidate cytidylyltransferase Tam41 (p	-0.8818493	6.29477286	1.19E-09	8.25E-09
SPAC1687.08	SPAC1687.08	Schizosaccharomyces pombe specific protein	-0.8825484	3.95769968	0.13095033	0.197498995
SPAC19G12.06c	hta2	histone H2A beta	-0.8827782	7.62957956	3.81E-15	4.61E-14
SPCPJ732.02c	xks1	xylulose kinase Xks1 (predicted)	-0.8862644	8.58625399	9.63E-25	2.45E-23
SPNCRNA.1622	#N/A	#N/A	-0.8866582	4.28708821	0.00606167	0.013506769
SPCC645.02	gep4	phosphatidylglycerol phosphate phosphatase Gep4 (predict	-0.8880132	5.23036001	7.20E-05	0.000243213
SPCC1529.01	SPCC1529.01	transmembrane transporter (predicted)	-0.8917554	6.34593611	7.54E-11	5.95E-10
SPNCRNA.1074	#N/A	#N/A	-0.891845	7.2613029	1.07E-18	1.79E-17
SPNCRNA.443	#N/A	#N/A	-0.8928174	2.55760512	0.13968621	0.208320114
SPAC1002.05c	jmj2	histone demethylase Jmj2	-0.8935762	6.488065	4.30E-12	3.97E-11

SPCC1235.13	ght6	plasma membrane glucose/fructose:proton symporter Ght6	-0.8947738	6.44506492	4.40E-12	4.04E-11
SPNCRNA.1118	#N/A	#N/A	-0.8947738	6.44506492	4.40E-12	4.04E-11
SPNCRNA.632	#N/A	#N/A	-0.8949352	7.21776863	1.82E-14	2.06E-13
SPAC12G12.16c	SPAC12G12.16c	Fen1 family nuclease, XP-G family (predicted)	-0.8952992	6.02060806	5.24E-09	3.33E-08
SPCC63.07	thg1	tRNAHis guanylyltransferase Thg1 (predicted)	-0.8957881	5.59449588	8.15E-07	3.80E-06
SPNCRNA.390	#N/A	#N/A	-0.8977853	2.92641181	0.04551021	0.079839382
SPNCRNA.1100	#N/A	#N/A	-0.8981297	6.77496684	1.18E-06	5.39E-06
SPBC1105.13c	SPBC1105.13c	Schizosaccharomyces pombe specific protein	-0.9010946	5.41914863	5.68E-06	2.36E-05
SPNCRNA.1668	#N/A	#N/A	-0.901122	7.64292779	8.15E-08	4.37E-07
ScpofMt17	#N/A	#N/A	-0.9026515	2.06287196	0.14798606	0.218637682
SPNCRNA.532	#N/A	#N/A	-0.9029507	5.63579819	1.31E-06	5.96E-06
SPNCRNA.786	#N/A	#N/A	-0.9034109	4.37857793	0.009485	0.020101337
SPNCRNA.1052	#N/A	#N/A	-0.903415	5.63364049	7.84E-08	4.22E-07
SPBC1604.18c	cmp7	ESCRT III complex subunit Cmp7 (predicted)	-0.9040691	5.13967622	7.75E-06	3.14E-05
SPBC1347.03	meu14	sporulation specific PIL domain protein Meu14	-0.9041752	5.52429	2.88E-05	0.000104589
SPBC1348.15	SPBC1348.15	Schizosaccharomyces pombe specific protein	-0.9042493	1.30013156	0.56753043	0.656586977
SPBCPT2R1.03	SPBCPT2R1.03	hypothetical protein	-0.9042493	1.30013156	0.56753043	0.656586977
SPNCRNA.43	#N/A	#N/A	-0.9047038	2.23763185	0.15673439	0.229420844
SPBC337.12	red5	zf-CCCH type zinc finger protein, NURS complex subunit Red	-0.9052832	6.68369585	2.97E-10	2.22E-09
SPBC17D11.05	tif32	translation initiation factor eIF3a	-0.9075785	7.21610502	9.97E-17	1.39E-15
SPCC757.03c	hsp3101	glyoxylase III Hsp3101	-0.9092324	6.77858402	3.38E-07	1.66E-06
SPAC20G4.03c	hri1	eIF2 alpha kinase Hri1	-0.9112388	8.37178388	5.95E-18	9.32E-17
SPBC1198.01	fmd2	glutathione-dependent formaldehyde dehydrogenase Fmd2	-0.9118237	7.05905861	9.87E-12	8.76E-11
SPAC13F5.04c	vta1	Vps20 associated protein Vta1 (predicted)	-0.913142	6.2982755	6.35E-09	4.00E-08
SPCC737.04	SPCC737.04	UPF0300 family protein 6	-0.913531	9.28611304	1.04E-38	6.20E-37
SPAC3C7.02c	pil2	meiotic eisosome BAR domain protein Pil2	-0.9142686	6.16308266	3.62E-10	2.66E-09
SPBC18H10.18c	SPBC18H10.18c	Schizosaccharomyces specific protein	-0.9144636	6.10722016	2.29E-09	1.51E-08
SPBC660.09	mug168	Schizosaccharomyces specific protein Mug168	-0.9145359	5.83690249	6.97E-08	3.77E-07
SPBC1348.04	SPBC1348.04	methyltransferase (predicted)	-0.9146828	4.31909393	0.00493984	0.011231454
SPBP8B7.27	mug30	HECT-type ubiquitin-protein ligase E3, found in association	-0.9152662	7.21282673	1.15E-14	1.33E-13
SPNCRNA.1333	#N/A	#N/A	-0.9169208	3.18643158	0.05749149	0.097612167
SPNCRNA.1320	#N/A	#N/A	-0.918689	7.0413771	1.34E-11	1.17E-10
SPNCRNA.301	#N/A	#N/A	-0.9212244	3.68490375	0.01567359	0.031518552
SPAC11D3.01c	SPAC11D3.01c	Con-6 family conserved fungal protein	-0.92254	5.91035139	2.13E-08	1.24E-07
SPBC19C7.09c	uve1	endonuclease Uve1	-0.9239916	6.84037254	4.99E-14	5.49E-13
SPAC23H3.13c	gpa2	heterotrimeric G protein alpha-2 subunit Gpa2	-0.9241484	5.8649302	6.66E-08	3.63E-07
SPBC29A3.14c	trt1	telomerase reverse transcriptase 1 protein Trt1	-0.9251827	6.7109457	3.95E-12	3.67E-11
SPNCRNA.1433	#N/A	#N/A	-0.9254977	3.81151387	0.00595557	0.013309563
SPNCRNA.1093	#N/A	#N/A	-0.9257783	4.08325143	0.00084884	0.002317972
SPBC36.10	ups2	phosphatidic acid transfer protein Ups2 (predicted)	-0.9262521	6.44661444	3.67E-12	3.42E-11
SPNCRNA.1108	#N/A	#N/A	-0.9286297	3.52082088	0.01834534	0.036396514
ScpofMp01	#N/A	#N/A	-0.9327833	10.5733703	1.18E-12	1.16E-11
SPAC9E9.01	#N/A	#N/A	-0.9332428	1.86480698	0.25396083	0.344195074
SPBC1347.01c	rev1	deoxycytidyl transferase Rev1 (predicted)	-0.9347238	6.07916863	4.23E-09	2.72E-08
SPNCRNA.740	#N/A	#N/A	-0.9348279	2.8225713	0.09561045	0.1510411
SPCC1235.12c	mug146	Schizosaccharomyces specific protein Mug46	-0.9348614	2.70789005	0.06266193	0.105105851
SPBC211.07c	ubc8	ubiquitin conjugating enzyme E2 Ubc8 (predicted)	-0.9367158	7.04358577	1.16E-13	1.24E-12

SPAC26H5.04	gid5	GID complex armadillo repeat subunit Gid5 (predicted)	-0.9367287	7.445198	3.44E-17	5.02E-16
SPAC11E3.02c	ync13	Munc family exocytic/endocytic regulator Ync13	-0.936812	8.44168184	6.85E-31	2.58E-29
SPAP8A3.13c	SPAP8A3.13c	Vid24 family protein (predicted)	-0.9378345	7.21566865	1.24E-19	2.30E-18
SPCC1919.02	pbn1	glycosylphosphatidylinositol-mannosyltransferase I complex	-0.9407534	6.39580254	1.90E-12	1.83E-11
SPNCRNA.1330	#N/A	#N/A	-0.9421275	4.74094458	3.59E-05	0.000128002
SPAP11E10.02c	mam3	cell surface adhesion protein for conjugation Mam3	-0.9428089	7.17249982	5.53E-07	2.65E-06
SPNCRNA.1630	#N/A	#N/A	-0.9431825	6.61184159	6.72E-14	7.26E-13
SPAC3C7.13c	SPAC3C7.13c	glucose-6-phosphate 1-dehydrogenase (predicted)	-0.9441152	8.61251652	3.00E-28	9.58E-27
SPNCRNA.1339	#N/A	#N/A	-0.9451247	2.26920974	0.16749596	0.242577383
SPAC11H11.05c	fta6	Mis6-Sim4 complex Fta6	-0.9459176	3.25240247	0.0374399	0.067490996
SPNCRNA.516	#N/A	#N/A	-0.9467216	2.2685595	0.12443491	0.189055497
SPAC20G8.10c	atg6	autophagy associated beclin family protein Atg6	-0.9470301	5.92856077	2.14E-09	1.42E-08
SPBC215.14c	vps20	ESCRT III complex subunit Vps20	-0.9474022	5.78170421	6.47E-08	3.53E-07
SPBC215.10	odr1	HAD superfamily hydrolase, unknown role	-0.9475914	7.91071318	2.68E-26	7.52E-25
SPAC1002.04c	taf11	transcription factor TFIID complex subunit Taf11 (predicted)	-0.9487582	5.71616566	8.61E-07	4.01E-06
SPNCRNA.650	#N/A	#N/A	-0.9493445	3.76786126	0.00409001	0.009489442
SPNCRNA.134	#N/A	#N/A	-0.9513023	6.79437632	2.43E-13	2.54E-12
SPNCRNA.319	#N/A	#N/A	-0.9514084	2.58439386	0.09015273	0.143809031
SPNCRNA.389	#N/A	#N/A	-0.9541991	2.84306892	0.04379285	0.077405278
SPAC6C3.03c	SPAC6C3.03c	Schizosaccharomyces pombe specific protein	-0.9556894	5.0560903	2.71E-05	9.92E-05
SPBC20F10.05	nrl1	RNAi-mediated silencing protein, human NRDE2 ortholog N	-0.9559175	7.10760349	1.71E-14	1.95E-13
SPACUNK4.10	gor1	glyoxylate reductase (predicted)	-0.9578274	8.89812697	4.76E-34	2.20E-32
SPAC732.02c	SPAC732.02c	fructose-2,6-bisphosphate 2-phosphatase activity (predicted	-0.9612259	7.34344659	3.50E-17	5.10E-16
SPAC11H11.02c	mug162	Schizosaccharomyces specific protein Mug162	-0.9623316	5.92070864	1.76E-07	8.97E-07
SPAC14C4.16	dad3	DASH complex subunit Dad3	-0.9624467	5.11715963	0.0001111	0.000363652
SPNCRNA.1193	#N/A	#N/A	-0.9637867	3.80142295	0.00736481	0.016027358
SPAC2F3.08	sut1	plasma membrane sucrose/maltose:proton symporter Sut1	-0.9640329	8.0434168	6.00E-20	1.14E-18
SPAC11E3.08c	nse6	Smc5-6 complex non-SMC subunit Nse6	-0.9650484	5.35741142	1.37E-05	5.29E-05
SPCC16A11.15c	SPCC16A11.15c	Schizosaccharomyces specific protein	-0.9654803	6.08057912	1.19E-08	7.17E-08
SPAC16A10.08c	mug74	Schizosaccharomyces specific protein Mug74	-0.9661655	5.22469547	1.43E-06	6.47E-06
SPNCRNA.1663	#N/A	#N/A	-0.9663922	5.66874905	1.66E-06	7.43E-06
SPNCRNA.921	#N/A	#N/A	-0.967522	4.77206084	0.00080497	0.00221146
SPCC5E4.10c	SPCC5E4.10c	human leukocyte receptor 1 ortholog	-0.9682257	5.41883878	7.27E-07	3.41E-06
SPAC1F12.03c	#N/A	#N/A	-0.9685458	2.65931919	0.12414491	0.188673552
SPBC725.03	SPBC725.03	pyridoxamine 5'-phosphate oxidase (predicted)	-0.9714269	6.15246688	1.28E-09	8.83E-09
ScpofMp05	#N/A	#N/A	-0.9715226	10.9589732	9.19E-14	9.83E-13
SPNCRNA.1576	#N/A	#N/A	-0.9718441	5.15336342	4.55E-06	1.91E-05
SPAC1071.13	#N/A	#N/A	-0.9726816	5.97811073	5.95E-10	4.26E-09
SPAC11D3.19	SPAC11D3.19	Schizosaccharomyces pombe specific protein	-0.97486	5.57072916	1.36E-07	7.04E-07
SPNCRNA.982	#N/A	#N/A	-0.9750721	5.31741091	8.37E-08	4.49E-07
SPNCRNA.1497	#N/A	#N/A	-0.9761672	2.0984728	0.12622697	0.191265755
SPBC3B9.09	vps36	ESCRT II complex subunit Vps36	-0.9771508	5.77986278	7.99E-06	3.23E-05
SPNCRNA.919	#N/A	#N/A	-0.9781222	4.77614335	1.60E-05	6.10E-05
SPNCRNA.1205	#N/A	#N/A	-0.9791541	5.26929256	1.25E-05	4.87E-05
SPAC11E3.03	csm1	microtubule-site clamp monopolin complex subunit Csm1/P	-0.9814452	5.2536888	3.03E-05	0.000109449
SPNCRNA.399	#N/A	#N/A	-0.9846782	2.60939701	0.072281	0.118666392
SPBC83.09c	lin1	U5 snRNP subunit Snu40 (predicted)	-0.9848015	6.08970533	1.56E-08	9.26E-08

SPNCRNA.451	#N/A	#N/A	-0.985368	2.44980736	0.09004441	0.143700716
SPNCRNA.1605	#N/A	#N/A	-0.9854788	4.11917848	0.00037957	0.001126461
SPNCRNA.881	#N/A	#N/A	-0.9858872	4.25645193	0.00070486	0.00197308
SPNCRNA.885	#N/A	#N/A	-0.9866737	5.41081238	3.90E-07	1.91E-06
SPAC27D7.02c	grp1	Golgi GRIP domain protein Grp1 (predicted)	-0.9868725	7.52936979	4.88E-17	7.03E-16
SPNCRNA.500	#N/A	#N/A	-0.9877636	3.47920369	0.00824506	0.017688406
SPAC4A8.09c	cwf21	complexed with Cdc5 protein Cwf21	-0.9888362	6.93628185	4.94E-19	8.55E-18
SPBC1826.01c	mot1	TATA-binding protein-associated transcription initiation rep	-0.9890518	9.04756331	5.25E-42	3.97E-40
SPAC869.09	SPAC869.09	Con-6 family conserved fungal protein	-0.990909	4.04644958	0.00050946	0.00147337
SPNCRNA.227	#N/A	#N/A	-0.9910399	4.67264603	0.00063046	0.001780924
SPAC1486.04c	alm1	nucleoporin Alm1	-0.9913204	8.09320774	3.26E-22	7.43E-21
SPBC21B10.15	SPBC21B10.15	Schizosaccharomyces specific protein	-0.9918835	5.00592511	0.00047256	0.001376603
SPBC887.17	SPBC887.17	nucleobase transmembrane transporter (predicted)	-0.9919573	8.62348964	6.41E-28	2.01E-26
SPAC23C11.06c	SPAC23C11.06c	vacuolar membrane hydrolase, implicted in protein cataboli	-0.9924436	9.11131819	7.90E-22	1.78E-20
SPAC1F7.08	fio1	plasma membrane iron transport multicopper oxidase Fio1	-0.9934008	8.37177509	2.58E-22	5.92E-21
SPCC1494.02c	taf13	transcription factor TFIID complex subunit Taf13 (predicted	-0.994012	5.87386996	7.73E-08	4.16E-07
SPBC543.05c	bor1	plasma membrane borate efflux transmembrane transporte	-0.9945018	5.93189565	1.19E-09	8.25E-09
SPNCRNA.798	#N/A	#N/A	-0.9946472	9.10966833	7.91E-22	1.78E-20
SPNCRNA.1053	#N/A	#N/A	-0.9950002	5.45446681	7.55E-08	4.07E-07
SPCC1919.04	SPCC1919.04	Schizosaccharomyces specific protein	-0.9956859	5.49248197	1.39E-07	7.19E-07
SPCC63.03	SPCC63.03	mitochondrial MIB complex subunit, human DNAJC11 ortho	-0.9960242	6.53976043	6.41E-14	6.96E-13
SPNCRNA.570	#N/A	#N/A	-0.9987078	5.69692808	1.37E-07	7.11E-07
SPNCRNA.1303	#N/A	#N/A	-1.0035223	2.92711365	0.0189542	0.037473367
SPAC1A6.11	#N/A	#N/A	-1.0062312	4.52146494	3.70E-05	0.000131605
SPNCRNA.1604	#N/A	#N/A	-1.0062594	4.18026422	0.00023087	0.000715413
SPNCRNA.1302	#N/A	#N/A	-1.0072318	8.64608148	8.37E-23	1.94E-21
SPNCRNA.306	#N/A	#N/A	-1.0076718	2.68433795	0.04795008	0.083495462
SPBC215.04	git11	heterotrimeric G protein gamma subunit Git11	-1.0084936	4.57362424	0.00080286	0.002207452
SPNCRNA.1660	#N/A	#N/A	-1.0085124	6.82727855	2.03E-18	3.30E-17
SPNCRNA.263	#N/A	#N/A	-1.0095422	1.63556724	0.29339481	0.386105529
SPAC1556.01c	rad50	DNA repair protein Rad50	-1.0099885	7.89230365	4.66E-20	8.96E-19
SPBC16C6.10	chp2	heterochromatin (HP1) family chromodomain protein Chp2	-1.0100622	5.4625318	2.28E-06	1.00E-05
SPNCRNA.1054	#N/A	#N/A	-1.0103518	2.39266697	0.1327732	0.199849957
SPBC30B4.09	SPBC30B4.09	Schizosaccharomyces specific protein	-1.0106373	5.73813445	3.40E-08	1.95E-07
SPAC869.06c	hry1	HHE domain cation binding protein (predicted)	-1.0115666	4.30315597	0.00101113	0.002708949
ScpofMp04	#N/A	#N/A	-1.0136931	11.3147181	2.58E-12	2.44E-11
SPNCRNA.173	#N/A	#N/A	-1.0150382	0.94659076	0.69611748	0.768570505
SPBPB21E7.01c	eno102	enolase (predicted)	-1.0172275	8.74166426	1.56E-23	3.77E-22
SPNCRNA.13	#N/A	#N/A	-1.0198413	3.04359894	0.04577402	0.080198812
SPAC23D3.12	SPAC23D3.12	plasma membrane inorganic phosphate transmembrane tra	-1.0206069	8.11441987	1.37E-28	4.51E-27
SPAC6C3.07	mug68	Schizosaccharomyces specific protein Mug68	-1.0247615	5.24941359	3.40E-07	1.67E-06
SPNCRNA.205	#N/A	#N/A	-1.0258352	5.59096267	4.69E-07	2.27E-06
SPAC57A10.04	mug10	meiotic Rho guanine nucleotide exchange factor (predicted	-1.0262663	4.46922061	0.00022595	0.000701787
SPNCRNA.626	#N/A	#N/A	-1.0265401	5.70206106	1.71E-09	1.16E-08
SPNCRNA.101	#N/A	#N/A	-1.0273652	4.873521	8.69E-06	3.47E-05
SPNCRNA.91	#N/A	#N/A	-1.0274212	1.90552565	0.18371282	0.261869655
SPNCRNA.1439	#N/A	#N/A	-1.0279096	5.88997617	8.77E-09	5.40E-08

SPNCRNA.62	#N/A	#N/A	-1.0289999	4.88950777	6.74E-06	2.76E-05
SPAC12B10.10	nod1	medial cortical node Gef2-related protein protein Nod1	-1.0296437	4.82228645	0.00010137	0.000334219
SPAC9E9.09c	atd1	aldehyde dehydrogenase (predicted)	-1.0301467	9.12042596	2.01E-30	7.38E-29
SPAP27G11.11c	#N/A	#N/A	-1.0308672	0.94684644	0.69620422	0.768570505
SPBC685.02	exo5	mitochondrial single stranded DNA specific 5'-3' exodeoxyri	-1.0337351	5.48780551	2.71E-09	1.77E-08
SPBPB21E7.02c	SPBPB21E7.02c	phosphoglycerate mutase/6-phosphofructo-2-kinase family	-1.0339566	4.57413638	6.81E-05	0.000231519
SPAC1952.09c	SPAC1952.09c	acetyl-CoA hydrolase (predicted)	-1.0366114	7.79842	1.07E-20	2.21E-19
SPNCRNA.936	#N/A	#N/A	-1.0375227	7.01244574	1.03E-16	1.43E-15
SPAC4F10.16c	SPAC4F10.16c	plasma membrane phospholipid-translocating ATPase com	-1.0380647	8.41994035	2.47E-32	1.04E-30
SPNCRNA.495	#N/A	#N/A	-1.0382238	5.02729828	7.36E-06	2.99E-05
SPAC22H10.09	SPAC22H10.09	Schizosaccharomyces specific protein	-1.0392101	6.39471473	2.11E-05	7.87E-05
SPBC1306.01c	mef1	mitochondrial translation elongation factor G Mef1 (predict	-1.0397367	6.70081426	3.85E-14	4.27E-13
SPNCRNA.275	#N/A	#N/A	-1.0397874	5.68301639	1.16E-08	7.03E-08
SPNCRNA.188	#N/A	#N/A	-1.0398917	2.13372661	0.13360614	0.200925915
SPNCRNA.791	#N/A	#N/A	-1.0407619	5.95912761	4.83E-10	3.50E-09
SPNCRNA.1499	#N/A	#N/A	-1.0417764	2.30110148	0.13871086	0.207047029
SPBC56F2.06	mug147	Schizosaccharomyces specific protein Mug147	-1.0422545	7.75896186	6.68E-11	5.30E-10
SPAC23E2.03c	ste7	arrestin family meiotic suppressor protein Ste7	-1.0440354	7.8222085	1.70E-17	2.54E-16
SPNCRNA.1281	#N/A	#N/A	-1.0454289	4.56035738	0.00033623	0.00101062
SPBC1A4.03c	top2	DNA topoisomerase II	-1.0455098	6.60290173	4.80E-13	4.88E-12
SPAC328.08c	tbc1	tubulin specific chaperone cofactor C, GTPase activating pro	-1.0455347	5.29668285	6.68E-07	3.16E-06
SPNCRNA.916	#N/A	#N/A	-1.0472804	3.84615707	0.00221031	0.005495478
SPNCRNA.825	#N/A	#N/A	-1.0494455	3.06345223	0.00946093	0.020056581
SPAC27F1.04c	nuf2	NMS complex subunit Nuf2	-1.0495454	5.95809518	7.87E-09	4.88E-08
SPNCRNA.920	#N/A	#N/A	-1.0500088	4.8375126	6.53E-06	2.68E-05
SPAC31G5.06	rrg8	mitochondrial conserved fungal protein Rrg8 (predicted)	-1.0507184	4.03449115	0.00226276	0.005617687
SPBC1685.04	SPBC1685.04	Schizosaccharomyces specific protein	-1.0579695	5.59753891	8.10E-09	5.01E-08
SPNCRNA.756	#N/A	#N/A	-1.0605526	3.51984302	0.00276252	0.006694771
SPAC110.05	#N/A	#N/A	-1.061276	4.11253738	0.00070095	0.001965377
SPNCRNA.274	#N/A	#N/A	-1.0614037	3.51998816	0.00256922	0.006266505
SPAC56F8.14c	mug115	Schizosaccharomyces pombe specific protein Mug115	-1.0615315	7.90569283	3.18E-32	1.32E-30
SPNCRNA.79	#N/A	#N/A	-1.0630836	3.92027005	0.00084109	0.002301422
SPCC70.04c	SPCC70.04c	Schizosaccharomyces pombe specific protein	-1.0641148	6.61219112	7.77E-15	9.16E-14
SPAC3A12.19	SPAC3A12.19	mitochondrial ribosomal protein subunit L27 (predicted)	-1.0657071	5.27478008	5.24E-07	2.52E-06
SPNCRNA.1200	#N/A	#N/A	-1.0659152	4.26271042	0.00015401	0.000492261
SPNCRNA.815	#N/A	#N/A	-1.0724164	5.33642037	6.89E-08	3.73E-07
SPBC36.13	#N/A	#N/A	-1.0738093	4.48276788	3.14E-05	0.000113033
SPAC6G10.06	tda3	FAD-dependent amino acid oxidase involved in late endosor	-1.0745491	3.90716058	0.00152831	0.003930525
SPAC25G10.04c	rec10	meiotic recombination protein Rec10	-1.08096	6.94214625	2.57E-19	4.60E-18
SPBC14C8.09c	dbl3	IMPACT domain protein, possible chaperone (predicted)	-1.0809842	4.65837633	4.76E-05	0.000166172
SPAC607.09c	btn1	battenin CLN3 family protein	-1.0839502	5.91777734	4.27E-10	3.12E-09
SPAC1006.09	win1	MAP kinase kinase kinase Win1	-1.0848438	8.03669521	9.38E-34	4.31E-32
SPAC12G12.11c	miy1	Lys48-specific deubiquitinase Mindy family, Miy1	-1.0862275	5.66498926	7.36E-09	4.58E-08
SPAC2G11.02	urb2	ribosome biogenesis protein Urb2 (predicted)	-1.0880699	6.74334527	1.21E-17	1.83E-16
SPNCRNA.1366	#N/A	#N/A	-1.0904839	4.83505763	6.10E-06	2.52E-05
SPNCRNA.898	#N/A	#N/A	-1.0928621	6.63409524	3.44E-13	3.56E-12
SPNCRNA.256	#N/A	#N/A	-1.0959532	1.36201371	0.3054826	0.399003662

SPBC13G1.16	SPBC13G1.16	Schizosaccharomyces specific protein	-1.0986673	3.46430948	0.01336966	0.027363264
SPNCRNA.38	#N/A	#N/A	-1.0990584	1.53131778	0.33464709	0.428945838
SPNCRNA.1442	#N/A	#N/A	-1.102172	4.6892931	2.80E-06	1.22E-05
SPNCRNA.341	#N/A	#N/A	-1.1069354	1.68363258	0.26495356	0.355974134
SPAC13G6.13	SPAC13G6.13	Schizosaccharomyces pombe specific protein	-1.1094703	1.68383019	0.19923981	0.279956004
SPAC22F8.03c	SPAC22F8.03c	Schizosaccharomyces pombe specific protein	-1.1099761	1.68422919	0.19471744	0.274679138
SPNCRNA.896	#N/A	#N/A	-1.1104284	3.95794023	0.00085546	0.00233417
SPAC9.13c	cwf16	splicing factor Cwf16	-1.1113036	6.48610109	8.60E-13	8.57E-12
SPAC4G9.13c	vps26	retromer complex subunit Vps26	-1.1114712	8.59337765	4.71E-38	2.69E-36
SPAC186.02c	SPAC186.02c	hydroxyacid dehydrogenase, implicated in cellular detoxifica	-1.114746	3.73402774	0.00202349	0.005068028
SPNCRNA.621	#N/A	#N/A	-1.1163948	4.0930113	0.00124012	0.003257051
SPNCRNA.100	#N/A	#N/A	-1.120804	3.34781058	0.00667568	0.01468262
SPBC16E9.17c	rem1	meiosis-specific cyclin Rem1	-1.1221265	3.43581479	0.00362448	0.008505028
SPNCRNA.1175	#N/A	#N/A	-1.1225737	4.76605219	1.90E-05	7.14E-05
SPNCRNA.1057	#N/A	#N/A	-1.1303614	2.2695032	0.05197193	0.089559131
SPCC1753.02c	git3	G-protein coupled receptor Git3	-1.1349751	8.40284311	5.18E-42	3.96E-40
SPAC23H3.04	SPAC23H3.04	conserved fungal multispanning membrane protein	-1.135991	5.33303152	3.18E-09	2.07E-08
SPAC14C4.03	mek1	Cds1/Rad53/Chk2 family protein kinase Mek1	-1.1363017	4.04000931	0.00254172	0.006215019
SPNCRNA.71	#N/A	#N/A	-1.1372954	2.53198765	0.03295919	0.060471092
SPBC1D7.02c	scr1	transcription factor Scr1	-1.1397024	7.19536422	1.62E-09	1.10E-08
SPNCRNA.644	#N/A	#N/A	-1.1401744	4.29431753	0.00052578	0.001513508
SPBC19G7.08c	art1	arrestin family protein Art1	-1.1414307	6.34707771	5.92E-14	6.45E-13
SPNCRNA.502	#N/A	#N/A	-1.1461911	3.0063648	0.00695651	0.015241151
SPNCRNA.1227	#N/A	#N/A	-1.1495211	7.6200281	9.62E-32	3.83E-30
SPNCRNA.42	#N/A	#N/A	-1.1496335	3.87778641	0.0088431	0.01885845
SPNCRNA.1463	#N/A	#N/A	-1.1498037	3.11858776	0.00606533	0.013510495
SPCC4G3.03	SPCC4G3.03	WD40/YVTN repeat-like protein	-1.1503053	6.33127374	2.92E-12	2.74E-11
SPNCRNA.1649	#N/A	#N/A	-1.1512753	5.93288624	6.15E-12	5.57E-11
SPNCRNA.212	#N/A	#N/A	-1.151835	4.16485988	0.00012527	0.00040632
SPNCRNA.268	#N/A	#N/A	-1.1526499	2.53269195	0.03633464	0.065794894
SPBC8E4.05c	SPBC8E4.05c	fumarate lyase superfamily, unknown specificity, bacterial 1	-1.1571085	5.76218648	1.19E-12	1.17E-11
SPBC31F10.08	mde2	Mde2 protein	-1.1658575	4.69314316	2.57E-06	1.12E-05
SPNCRNA.1010	#N/A	#N/A	-1.1698522	3.13599844	0.007488	0.016259033
SPNCRNA.1218	#N/A	#N/A	-1.1727093	4.25584029	0.00013654	0.000440358
SPAC6G9.06c	pcp1	gamma tubulin complex linker, pericentrin/kendrin Pcp1	-1.1755532	7.50012319	5.58E-27	1.64E-25
SPNCRNA.178	#N/A	#N/A	-1.1764438	4.71246829	7.95E-06	3.21E-05
SPNCRNA.20	#N/A	#N/A	-1.1806773	5.57121143	3.20E-10	2.37E-09
SPAC1399.01c	SPAC1399.01c	nucleobase transmembrane transporter (predicted)	-1.1810493	5.36032437	1.41E-09	9.60E-09
SPAC167.06c	mug143	Schizosaccharomyces specific protein Mug143	-1.1820978	6.44658384	4.96E-17	7.12E-16
SPNCRNA.289	#N/A	#N/A	-1.1844994	2.7082352	0.0193259	0.038097546
SPNCRNA.854	#N/A	#N/A	-1.1848899	4.13681268	7.95E-05	0.000266119
SPNCRNA.830	#N/A	#N/A	-1.1919492	3.20295056	0.0073865	0.016061226
SPCC663.17	wtf15	wtf element Wtf15	-1.1940053	5.00971154	5.21E-07	2.51E-06
SPNCRNA.260	#N/A	#N/A	-1.1950053	2.30142252	0.03848733	0.069170174
SPNCRNA.1385	#N/A	#N/A	-1.1956185	4.27272088	5.86E-06	2.43E-05
SPNCRNA.1272	#N/A	#N/A	-1.1957318	4.81910807	5.96E-07	2.84E-06
SPBC23G7.04c	nif1	protein kinase inhibitor, SEL1 repeat protein Nif1	-1.19838	6.95906258	3.09E-20	6.07E-19

SPNCRNA.247	#N/A	#N/A	-1.2013053	2.47798403	0.03907477	0.070066879
SPNCRNA.1606	#N/A	#N/A	-1.2039073	8.23393679	3.11E-39	1.92E-37
SPBC106.02c	srx1	sulfiredoxin	-1.2053981	4.09369683	5.32E-05	0.000184015
SPNCRNA.141	#N/A	#N/A	-1.2054324	2.84450081	0.03721631	0.067123512
SPNCRNA.723	#N/A	#N/A	-1.2090348	4.25637801	2.43E-05	9.00E-05
SPNCRNA.954	#N/A	#N/A	-1.2119538	7.45011708	6.29E-31	2.38E-29
SPBC18E5.08	SPBC18E5.08	N-acetyltransferase (predicted)	-1.2146673	5.35012353	1.28E-08	7.68E-08
SPBC365.12c	ish1	nuclear envelope LEA domain protein Ish1	-1.2164724	8.9011738	5.78E-13	5.85E-12
SPNCRNA.40	#N/A	#N/A	-1.2183711	1.86532895	0.12235276	0.186349709
SPNCRNA.358	#N/A	#N/A	-1.2214969	2.73164728	0.03044878	0.056443453
SPAC513.04	SPAC513.04	Schizosaccharomyces pombe specific protein	-1.2219864	1.73109507	0.14065028	0.209620053
SPAC1039.01	SPAC1039.01	amino acid transmembrane transporter (predicted)	-1.2221833	6.9223323	2.04E-28	6.57E-27
SPNCRNA.664	#N/A	#N/A	-1.2223779	1.58349576	0.25813546	0.348739608
SPAP27G11.08c	meu32	Schizosaccharomyces specific protein Meu32	-1.2229957	4.110703	2.99E-05	0.000108244
SPCC285.06c	wtf17	wtf element Wtf17	-1.2262872	5.26772304	2.09E-09	1.39E-08
SPNCRNA.1350	#N/A	#N/A	-1.2282372	3.57427545	0.00082358	0.002258036
SPNCRNA.437	#N/A	#N/A	-1.2295223	1.73130975	0.08317332	0.134026621
SPNCRNA.376	#N/A	#N/A	-1.2311919	1.58403623	0.23583268	0.323491515
SPNCRNA.962	#N/A	#N/A	-1.2341119	2.58492386	0.01775254	0.035313082
SPBC4.01	dni2	tetraspan protein, claudin Dni2	-1.2373497	4.4893511	1.17E-05	4.58E-05
SPNCRNA.159	#N/A	#N/A	-1.2381875	4.63092837	5.84E-06	2.42E-05
SPAC644.18c	bet3	TRAPP complex subunit Bet3 (predicted)	-1.2387637	3.93960849	6.98E-05	0.000236566
SPNCRNA.33	#N/A	#N/A	-1.2441653	1.42060843	0.29694548	0.38999401
SPNCRNA.177	#N/A	#N/A	-1.2460193	1.42022806	0.31626179	0.410326313
SPBC1E8.05	SPBC1E8.05	conserved fungal cell surface protein, Kre9/Knh1 family	-1.2515865	8.00203619	1.05E-25	2.79E-24
SPCC1442.01	ste6	guanyl-nucleotide exchange factor Ste6	-1.2519351	7.40126714	9.84E-32	3.89E-30
SPNCRNA.571	#N/A	#N/A	-1.252582	3.42284373	0.0009731	0.002621486
SPNCRNA.305	#N/A	#N/A	-1.2573722	2.42192183	0.01997175	0.039177782
SPNCRNA.801	#N/A	#N/A	-1.260801	6.47778736	5.17E-18	8.15E-17
SPNCRNA.1267	#N/A	#N/A	-1.2624002	3.91846167	0.00480221	0.010977163
SPAC1039.11c	gto1	alpha-glucosidase (predicted)	-1.2632606	8.26887816	4.05E-54	5.10E-52
SPNCRNA.405	#N/A	#N/A	-1.2658434	2.75459439	0.01325969	0.02717836
SPNCRNA.131	#N/A	#N/A	-1.2664746	4.31931649	2.11E-06	9.34E-06
SPNCRNA.1031	#N/A	#N/A	-1.2665789	7.24856745	3.83E-26	1.07E-24
SPBC359.05	abc3	ABC transmembrane transporter Abc3	-1.2669718	8.3350568	1.36E-51	1.50E-49
SPBC13G1.15c	SPBC13G1.15c	Schizosaccharomyces pombe specific protein	-1.2687479	2.23643034	0.04706849	0.082212799
SPNCRNA.1691	#N/A	#N/A	-1.2697492	4.08325863	8.69E-05	0.000289214
SPCC1183.10	wtf10	wtf element Wtf10	-1.2757568	4.9652279	3.95E-08	2.24E-07
SPNCRNA.1255	#N/A	#N/A	-1.277545	2.23742559	0.05759251	0.097759297
SPNCRNA.47	#N/A	#N/A	-1.277938	3.73203568	0.00036919	0.001098531
SPNCRNA.1312	#N/A	#N/A	-1.2789117	7.56597631	2.19E-35	1.07E-33
SPCC553.05c	wtf6	wtf element Wtf6	-1.2792736	5.39239276	7.19E-08	3.88E-07
SPAC4F10.15c	wsp1	WASp homolog	-1.2801388	7.57494902	6.18E-31	2.35E-29
SPCP31B10.06	tcb2	tricalbin, C2 domain protein (phospholipid binding) ER-plası	-1.2810156	7.94743262	4.28E-18	6.81E-17
SPAC1556.06.1	#N/A	#N/A	-1.2821568	5.32269297	3.79E-09	2.45E-08
SPAC1805.15c	pub2	HECT-type ubiquitin-protein ligase E3 Pub2	-1.2823704	7.05037982	8.25E-29	2.74E-27
SPNCRNA.1224	#N/A	#N/A	-1.2825146	4.09297276	1.96E-05	7.35E-05

SPAC2G11.05c	rim20	BRO1 domain protein Rim20	-1.3003862	6.15751181	3.95E-14	4.37E-13
SPNCRNA.99	#N/A	#N/A	-1.3042201	3.6619991	0.00023894	0.00073875
SPNCRNA.425	#N/A	#N/A	-1.3079686	3.50754394	0.00091653	0.002486831
SPAC4H3.03c	SPAC4H3.03c	glucan 1,4-alpha-glucosidase (predicted)	-1.3102904	8.10929549	2.83E-29	9.59E-28
SPAC513.03	mfm2	M-factor precursor Mfm2	-1.3104058	6.51045587	4.61E-08	2.59E-07
SPBC530.16	ksh1	FGolgi kish family protein Ksh1 (predicted)	-1.3121342	2.63495059	0.01728967	0.034442688
SPNCRNA.1347	#N/A	#N/A	-1.3121954	6.5315507	1.04E-17	1.58E-16
SPNCRNA.304	#N/A	#N/A	-1.3142001	6.49440869	1.68E-18	2.76E-17
SPAC2F7.06c	pol4	DNA polymerase X family	-1.3143983	6.52980338	2.74E-21	6.01E-20
SPNCRNA.774	#N/A	#N/A	-1.3188294	2.23847346	0.07411668	0.121153978
SPCC1235.14	ght5	plasma membrane high-affinity glucose/fructose:proton sy	-1.321228	9.92101954	1.50E-60	2.32E-58
SPNCRNA.1437	#N/A	#N/A	-1.3326809	3.59914573	0.00053876	0.001544989
SPNCRNA.1148	#N/A	#N/A	-1.3346537	1.77751576	0.10232888	0.159896222
SPNCRNA.821	#N/A	#N/A	-1.3363251	2.26962857	0.02454172	0.046806722
SPNCRNA.1352	#N/A	#N/A	-1.3440047	3.2367721	0.00124661	0.003271562
SPAC750.03c	SPAC750.03c	methyltransferase (predicted)	-1.3462961	3.28683111	0.00040874	0.001203583
SPNCRNA.828	#N/A	#N/A	-1.3464337	6.87471714	2.01E-28	6.51E-27
SPNCRNA.1075	#N/A	#N/A	-1.3511528	4.35178397	1.05E-06	4.85E-06
SPCC1393.12	SPCC1393.12	Schizosaccharomyces specific protein	-1.3548892	8.22593941	4.44E-34	2.07E-32
SPNCRNA.1133	#N/A	#N/A	-1.3554757	5.48802129	2.08E-12	2.00E-11
SPCC569.05c	SPCC569.05c	plasma membrane spermidine family transmembrane tran	-1.3593425	7.1989359	8.03E-37	4.17E-35
SPNCRNA.267	#N/A	#N/A	-1.3596979	1.63524073	0.1283419	0.194038353
SPAC1002.16c	SPAC1002.16c	carboxylic acid transmembrane transporter (predicted)	-1.3607225	3.87936184	0.00039393	0.001165007
SPAC16E8.18	SPAC16E8.18	Schizosaccharomyces pombe specific protein	-1.3630177	4.92715973	4.92E-09	3.14E-08
SPNCRNA.243	#N/A	#N/A	-1.3641426	2.94767	0.00302615	0.007256153
SPNCRNA.239	#N/A	#N/A	-1.3726036	2.47793221	0.02006689	0.039307772
SPNCRNA.951	#N/A	#N/A	-1.3726036	2.47793221	0.02006689	0.039307772
SPNCRNA.1318	#N/A	#N/A	-1.3797999	8.31843869	9.49E-57	1.34E-54
SPNCRNA.283	#N/A	#N/A	-1.3813065	1.63582942	0.14199707	0.211374798
SPBC25B2.08	SPBC25B2.08	Schizosaccharomyces pombe specific protein	-1.3821899	4.02862291	7.38E-06	2.99E-05
SPBC8D2.05c	sfi1	spindle pole body half bridge protein Sfi1	-1.3846977	4.94185951	9.92E-10	6.92E-09
SPNCRNA.95	#N/A	#N/A	-1.3862455	2.88645831	0.00670665	0.014741209
SPNCRNA.1062	#N/A	#N/A	-1.387556	3.89946964	0.00011104	0.000363625
SPNCRNA.04	#N/A	#N/A	-1.3880329	1.47715398	0.21172994	0.295069068
SPAC1F8.03c	str3	plasma membrane siderophore-iron transmembrane transp	-1.3902608	6.87662942	3.14E-25	8.26E-24
SPBC839.06	cta3	P-type ATPase, potassium exporting Cta3	-1.3920674	8.06291459	1.31E-39	8.49E-38
SPAC458.04c	dli1	meiotic dynein intermediate light chain Dli1/Dil1	-1.3946286	5.02052506	3.47E-10	2.56E-09
SPNCRNA.1327	#N/A	#N/A	-1.3948409	5.51652335	1.55E-14	1.77E-13
SPBC1683.09c	frp1	plasma membrane ferric-chelate reductase Frp1	-1.3950003	8.1063938	8.34E-41	5.79E-39
SPNCRNA.1598	#N/A	#N/A	-1.3952461	3.68610755	6.50E-05	0.000221428
SPNCRNA.1374	#N/A	#N/A	-1.3965199	7.95882826	2.49E-41	1.79E-39
SPNCRNA.1163	#N/A	#N/A	-1.4045881	4.77869708	4.54E-09	2.91E-08
SPNCRNA.478	#N/A	#N/A	-1.4062162	4.70257811	5.35E-08	2.95E-07
SPBC1685.14c	vid27	WD repeat protein, Vid27 family, conserved in fungi and pla	-1.4130474	7.0916868	1.10E-38	6.50E-37
SPAC26F1.11	#N/A	#N/A	-1.4131357	5.17625741	1.13E-08	6.82E-08
SPAPB15E9.02c	#N/A	#N/A	-1.4145415	7.42618965	2.25E-45	1.99E-43
SPNCRNA.520	#N/A	#N/A	-1.417478	3.10172941	0.00229487	0.005689106

SPAPB15E9.06	#N/A	#N/A	-1.4222354	5.50007974	1.70E-15	2.14E-14
SPNCRNA.69	#N/A	#N/A	-1.4234932	5.48955432	4.89E-15	5.86E-14
SPCC330.04c	mug135	mug2/mug135/meu2 family, with repeat expansion	-1.4238217	5.02362847	5.80E-09	3.66E-08
SPNCRNA.917	#N/A	#N/A	-1.4244196	1.82173372	0.06725725	0.111494661
SPNCRNA.552	#N/A	#N/A	-1.4250077	6.1470968	2.93E-19	5.20E-18
SPNCRNA.660	#N/A	#N/A	-1.4277607	6.26390682	1.27E-20	2.57E-19
SPNCRNA.1426	#N/A	#N/A	-1.4492634	3.25409306	0.00372289	0.008708928
SPNCRNA.1697	#N/A	#N/A	-1.4499075	7.17003473	5.21E-25	1.35E-23
SPNCRNA.1249	#N/A	#N/A	-1.453365	1.30023338	0.25968949	0.350215412
SPNCRNA.89	#N/A	#N/A	-1.4575934	1.30034148	0.24533326	0.334634767
SPAC4G9.12	idn1	gluconokinase	-1.4619663	8.07088528	2.12E-63	3.61E-61
SPBC18E5.14c	SPBC18E5.14c	Schizosaccharomyces specific protein	-1.4662551	4.63369111	2.10E-08	1.22E-07
SPAC32A11.02c	SPAC32A11.02c	DUF4449 family conserved fungal protein	-1.4678538	9.37640764	1.77E-27	5.41E-26
SPNCRNA.84	#N/A	#N/A	-1.4708216	2.70885052	0.00504042	0.011433342
SPAC186.04c	SPAC186.04c	N-terminal of transmembrane channel, truncated	-1.4765909	2.63587517	0.00422306	0.009764834
SPNCRNA.130	#N/A	#N/A	-1.4793029	7.6180485	4.66E-20	8.96E-19
SPNCRNA.994	#N/A	#N/A	-1.4793029	7.6180485	4.66E-20	8.96E-19
SPNCRNA.16	#N/A	#N/A	-1.4794415	1.68417413	0.1035156	0.161344946
SPAC869.08	pcm2	protein-L-isoaspartate O-methyltransferase Pcm2 (predicte	-1.4794546	4.8851858	2.95E-10	2.20E-09
SPAC869.05c	SPAC869.05c	plasma membrane sulfate transmembrane transporter (pre	-1.4803845	6.75857348	1.21E-26	3.44E-25
ScpofMp03	#N/A	#N/A	-1.4811106	7.33458541	3.74E-18	5.99E-17
SPNCRNA.45	#N/A	#N/A	-1.4839118	2.4511474	0.00610623	0.013588269
SPAPB1A10.02	scm3	CENP-A histone chaperone Scm3	-1.4864382	5.22761205	4.23E-11	3.48E-10
SPNCRNA.1616	#N/A	#N/A	-1.4873809	5.6889849	1.20E-13	1.28E-12
SPNCRNA.940	#N/A	#N/A	-1.4916759	2.75603955	0.05331611	0.091550879
SPNCRNA.106	#N/A	#N/A	-1.4938897	3.15316806	0.00036176	0.001077364
SPNCRNA.1210	#N/A	#N/A	-1.4983475	5.80679003	3.44E-16	4.59E-15
SPBC215.13	mtl3	plasma membrane-associated serine-rich cell wall sensor M	-1.5019321	5.75388947	9.60E-13	9.52E-12
SPNCRNA.10	#N/A	#N/A	-1.5039014	4.32011244	1.60E-07	8.22E-07
SPBPB2B2.11	tgd1	nucleotide-sugar 4,6-dehydratase (predicted)	-1.5056934	9.16102418	8.84E-65	1.54E-62
SPNCRNA.139	#N/A	#N/A	-1.5064594	2.96686824	0.00232737	0.00575917
SPBC15D4.11c	SPBC15D4.11c	mitochondrial Mam33 family protein (predicted)	-1.5098514	4.03876458	5.15E-05	0.000178571
SPAC9E9.17c	#N/A	#N/A	-1.5098899	1.86457817	0.05668674	0.096461422
SPNCRNA.1505	#N/A	#N/A	-1.5152413	6.16651206	3.95E-21	8.61E-20
SPBC646.17c	35764	meiotic dynein intermediate chain Dic1	-1.5167956	4.9638101	4.93E-12	4.50E-11
SPBC24C6.09c	SPBC24C6.09c	phosphoketolase family protein (predicted)	-1.5170157	6.15988058	2.28E-21	5.01E-20
SPNCRNA.1123	#N/A	#N/A	-1.5192369	2.13599294	0.01648698	0.033007917
SPNCRNA.194	#N/A	#N/A	-1.5287977	2.26974833	0.02737597	0.051504977
SPNCRNA.469	#N/A	#N/A	-1.5338741	2.58487592	0.00781945	0.016876411
SPBPB2B2.12c	gal10	UDP-glucose 4-epimerase/aldose 1-epimerase Gal10	-1.533993	9.20849537	3.45E-65	6.18E-63
SPNCRNA.604	#N/A	#N/A	-1.5373911	2.98646013	0.00201815	0.00505652
SPNCRNA.1162	#N/A	#N/A	-1.5467683	5.0047699	1.23E-11	1.08E-10
SPCC1906.03	wtf19	wtf element Wtf19	-1.549513	6.61501806	1.06E-26	3.08E-25
SPNCRNA.605	#N/A	#N/A	-1.5527996	6.95174296	3.22E-42	2.52E-40
SPNCRNA.1141	#N/A	#N/A	-1.5575361	1.09840313	0.30892589	0.402342708
SPNCRNA.168	#N/A	#N/A	-1.5616586	3.06503347	0.00043354	0.001271097
SPNCRNA.720	#N/A	#N/A	-1.5616586	3.06503347	0.00043354	0.001271097

SPCC1739.07	cti1	exosome C1D family subunit Cti1	-1.5652155	7.25691545	1.92E-29	6.64E-28
SPCC663.06c	osr1	short chain dehydrogenase, unknown specificity (predicted)	-1.5675085	5.69711438	1.44E-19	2.66E-18
SPNCRNA.1431	#N/A	#N/A	-1.5706938	4.38868808	5.18E-06	2.16E-05
SPNCRNA.1381	#N/A	#N/A	-1.5711456	5.97803536	8.41E-25	2.17E-23
SPNCRNA.1486	#N/A	#N/A	-1.5751442	3.53573274	2.20E-05	8.17E-05
SPAC1F8.05	isp3	spore wall structural constituent Isp3	-1.5774926	5.7019766	6.55E-18	1.02E-16
SPNCRNA.834	#N/A	#N/A	-1.5802014	5.32427304	3.06E-12	2.87E-11
SPNCRNA.974	#N/A	#N/A	-1.5814186	4.47089189	1.02E-07	5.38E-07
SPBPB2B2.13	gal1	galactokinase Gal1	-1.5895889	7.6150636	2.80E-33	1.26E-31
SPNCRNA.388	#N/A	#N/A	-1.5947889	3.54899043	1.57E-05	6.01E-05
SPNCRNA.398	#N/A	#N/A	-1.6015032	2.86646625	0.00129454	0.003388206
SPCC162.04c	wtf13	wtf element Wtf13	-1.6039875	6.45119425	8.69E-30	3.11E-28
SPNCRNA.993	#N/A	#N/A	-1.6129148	3.10044352	0.00016855	0.000533713
SPNCRNA.28	#N/A	#N/A	-1.6139157	5.68523876	5.16E-21	1.12E-19
SPNCRNA.1034	#N/A	#N/A	-1.6171634	2.88715427	0.00063683	0.001796696
SPNCRNA.627	#N/A	#N/A	-1.6278569	4.58866101	1.97E-09	1.31E-08
SPAC11H11.04	mam2	pheromone p-factor receptor	-1.6278618	7.87070468	2.15E-48	2.15E-46
SPNCRNA.1203	#N/A	#N/A	-1.6351576	7.39829839	3.81E-58	5.63E-56
SPNCRNA.1348	#N/A	#N/A	-1.6385087	3.88823342	8.77E-07	4.08E-06
SPNCRNA.1364	#N/A	#N/A	-1.6468066	5.57717415	1.31E-18	2.19E-17
SPAC1F8.04c	SPAC1F8.04c	hydrolase, implicated in cellular detoxification (predicted)	-1.6491118	6.78821591	1.78E-36	9.03E-35
SPNCRNA.943	#N/A	#N/A	-1.6560152	2.9067703	0.00093716	0.002535725
SPCC1442.11c	SPCC1442.11c	Schizosaccharomyces pombe specific protein	-1.6603462	2.3327587	0.00528224	0.011918316
SPAC16E8.18c	tam5	Schizosaccharomyces specific protein Tam5	-1.6669923	4.48702594	1.59E-07	8.18E-07
SPAC4D7.15	new12	Schizosaccharomyces specific protein New12	-1.6822031	4.06724943	1.17E-08	7.08E-08
SPAC4F10.17	SPAC4F10.17	conserved fungal protein	-1.6830531	5.81038028	1.47E-19	2.70E-18
SPNCRNA.237	#N/A	#N/A	-1.6872274	2.84370745	0.00087618	0.002384963
ScpofMp02	#N/A	#N/A	-1.708158	7.52410939	1.52E-17	2.28E-16
SPNCRNA.879	#N/A	#N/A	-1.7113228	2.36324944	0.00255484	0.006240661
SPAC29A4.12c	mug108	Schizosaccharomyces specific protein Mug108	-1.7154383	7.65714274	5.12E-61	8.30E-59
SPNCRNA.956	#N/A	#N/A	-1.7192209	3.30261142	2.92E-05	0.000106085
SPCC548.03c	wtf4	wtf element Wtf4	-1.7211331	6.2266616	8.47E-33	3.70E-31
SPNCRNA.184	#N/A	#N/A	-1.7212457	2.23805236	0.0059174	0.013237314
SPBC947.05c	frp2	ferric-chelate reductase Frp2 (predicted)	-1.7346418	5.36898342	1.76E-16	2.40E-15
SPNCRNA.103	sme2	meiRNA sme2	-1.7429084	4.96889406	2.13E-08	1.24E-07
SPNCRNA.869	#N/A	#N/A	-1.7457156	1.4204924	0.12130812	0.185090079
ScpofMt37	#N/A	#N/A	-1.7503063	2.80078926	0.00020182	0.000632605
SPNCRNA.1602	#N/A	#N/A	-1.7507136	4.46749534	1.90E-09	1.27E-08
SPNCRNA.156	#N/A	#N/A	-1.7594412	1.63474265	0.06308699	0.105714595
SPNCRNA.628	#N/A	#N/A	-1.7603578	3.92957328	4.26E-08	2.40E-07
SPBPB8B6.02c	SPBPB8B6.02c	plasma membrane urea transmembrane transporter (predi	-1.761606	4.22577919	3.35E-08	1.92E-07
SPNCRNA.1472	#N/A	#N/A	-1.762728	4.04705936	9.98E-06	3.95E-05
SPNCRNA.197	#N/A	#N/A	-1.7676649	5.26704686	2.13E-18	3.45E-17
SPNCRNA.327	#N/A	#N/A	-1.770444	1.16895367	0.19988982	0.2805218
SPNCRNA.296	#N/A	#N/A	-1.7756945	1.16892511	0.20204549	0.282963486
SPNCRNA.466	#N/A	#N/A	-1.7763263	3.39532964	0.000349	0.00104438
SPNCRNA.606	#N/A	#N/A	-1.7773344	5.10257483	7.53E-15	8.89E-14

SPNCRNA.512	#N/A	#N/A	-1.7805609	2.30048012	0.02269204	0.043732464
SPNCRNA.276	#N/A	#N/A	-1.7858917	4.13944002	2.54E-09	1.67E-08
SPNCRNA.338	#N/A	#N/A	-1.7865237	1.16947709	0.19642247	0.276912142
SPBC83.19c	SPBC83.19c	Schizosaccharomyces pombe specific protein	-1.7902823	3.40910839	0.00033353	0.00100295
SPNCRNA.1219	#N/A	#N/A	-1.7956223	4.82890896	1.30E-05	5.05E-05
SPCC1753.03c	rec7	meiotic recombination protein Rec7	-1.7986041	6.27706701	2.18E-34	1.03E-32
SPAC17A2.11	SPAC17A2.11	Schizosaccharomyces pombe specific protein	-1.8012203	3.22259742	1.10E-05	4.31E-05
SPBC1718.02	hop1	linear element associated protein Hop1	-1.8014573	4.50279756	5.32E-10	3.83E-09
SPNCRNA.831	#N/A	#N/A	-1.8041881	5.29366975	2.50E-19	4.47E-18
SPNCRNA.244	#N/A	#N/A	-1.8065315	4.99721793	1.38E-12	1.35E-11
SPNCRNA.587	#N/A	#N/A	-1.8148741	3.79086012	2.08E-07	1.05E-06
SPAC19A8.16	prl65	DUF2945 family protein	-1.8218593	5.36784443	5.38E-17	7.70E-16
SPAC186.05c	gdt1	Golgi calcium and manganese antiporter Gdt1	-1.8229126	2.02557386	0.01246004	0.025670765
SPCC1620.02	wtf23	wtf element Wtf23	-1.82567	6.50620158	2.73E-36	1.38E-34
SPNCRNA.1055	#N/A	#N/A	-1.8339078	3.30452094	3.61E-05	0.00012876
SPNCRNA.144	#N/A	#N/A	-1.8381489	4.18163462	1.49E-08	8.88E-08
SPNCRNA.534	#N/A	#N/A	-1.8462718	2.02631924	0.04372945	0.077313298
SPNCRNA.1128	#N/A	#N/A	-1.8468048	2.94873981	4.41E-05	0.000154502
SPNCRNA.1172	#N/A	#N/A	-1.8479004	2.02647935	0.0153664	0.030983214
SPBC16E9.16c	lsd90	Lsd90 protein	-1.8493591	9.24472421	5.47E-38	3.08E-36
SPNCRNA.145	#N/A	#N/A	-1.8532234	1.6834764	0.04683408	0.08184538
SPNCRNA.1216	#N/A	#N/A	-1.8546819	4.70330068	1.60E-12	1.55E-11
SPAC5H10.02c	hsp3102	glyoxylase III Hsp3102	-1.8581564	5.22587692	2.24E-14	2.53E-13
SPCC1235.18	#N/A	#N/A	-1.8655773	5.16876651	2.98E-16	4.01E-15
SPAC1D4.07c	#N/A	#N/A	-1.8659479	1.68441151	0.03016218	0.05598845
SPNCRNA.1109	#N/A	#N/A	-1.8670272	4.36823727	7.46E-10	5.26E-09
SPAC869.07c	mel1	alpha-galactosidase, melibiase	-1.871309	6.17547081	6.05E-32	2.44E-30
SPAC1687.23c	SPAC1687.23c	Schizosaccharomyces pombe specific protein	-1.8791095	1.68438625	0.02972643	0.055285154
SPNCRNA.505	#N/A	#N/A	-1.8839831	1.68527452	0.03344893	0.061320036
SPNCRNA.1617	#N/A	#N/A	-1.8938701	3.89950302	9.50E-08	5.04E-07
SPNCRNA.613	#N/A	#N/A	-1.9032898	4.85074344	3.23E-10	2.39E-09
SPCC338.18	SPCC338.18	Schizosaccharomyces pombe specific protein	-1.9204524	5.30963844	1.38E-18	2.29E-17
SPNCRNA.1048	#N/A	#N/A	-1.9328587	4.38238437	5.62E-09	3.56E-08
SPNCRNA.802	#N/A	#N/A	-1.9563664	5.83643154	1.50E-27	4.65E-26
SPAC4F8.08	mug114	Schizosaccharomyces pombe specific protein Mug114	-1.959771	7.15979342	8.23E-68	1.60E-65
SPNCRNA.187	#N/A	#N/A	-1.9653505	1.23639932	0.12346449	0.187832744
SPBPB2B2.10c	gal7	galactose-1-phosphate uridylyltransferase Gal7	-1.967032	6.54168563	7.98E-28	2.48E-26
SPNCRNA.1087	#N/A	#N/A	-1.9780214	7.34017832	5.96E-61	9.44E-59
SPAC13F5.07c	hpz2	zf PARP type zinc finger protein Hpz2	-1.9827018	5.85620014	8.14E-30	2.93E-28
SPNCRNA.311	#N/A	#N/A	-1.9980582	3.73403222	1.43E-05	5.49E-05
SPBC359.04c	pfl7	cell surface glycoprotein, flocculin Pfl7, DIPSY family	-2.0021931	5.76685396	1.70E-22	3.92E-21
SPNCRNA.876	#N/A	#N/A	-2.0037432	2.53394138	0.00034484	0.001032948
SPAC3G6.07	SPAC3G6.07	Schizosaccharomyces specific protein	-2.0100264	2.42159406	0.00038782	0.001148447
SPNCRNA.1186	#N/A	#N/A	-2.012092	2.42217083	0.00037632	0.001117309
SPAC1F7.06	hsp3105	ThiJ domain protein, implicated in cellular detoxification	-2.0186567	4.98986364	2.20E-18	3.55E-17
SPNCRNA.1420	#N/A	#N/A	-2.0190656	3.9990701	7.64E-08	4.12E-07
SPRRNA.13	SPRRNA.13	5S rRNA	-2.0429616	1.77707117	0.02469888	0.047053711

SPCC285.07c	wtf18	wtf element Wtf18	-2.0510156	5.22461476	3.89E-19	6.81E-18
SPBC1348.12	SPBC1348.12	transcription factor (predicted)	-2.072421	5.42915269	1.82E-28	5.95E-27
SPBC32H8.02c	nep2	NEDD8 protease Nep2	-2.0787179	8.07466661	1.68E-102	6.34E-100
SPNCRNA.1361	#N/A	#N/A	-2.1000673	3.11907034	0.0001068	0.000350413
SPNCRNA.282	#N/A	#N/A	-2.1031801	3.49511407	2.12E-08	1.24E-07
SPCPB16A4.06c	SPCPB16A4.06c	Schizosaccharomyces specific protein	-2.1147671	7.02322347	2.02E-68	4.29E-66
SPNCRNA.1370	#N/A	#N/A	-2.1358984	2.02475293	0.00190792	0.004808666
SPNCRNA.54	#N/A	#N/A	-2.1462355	2.96845876	9.38E-06	3.73E-05
SPBC1711.01c	mat3-Mi	mating type M-specific polypeptide Mi at silenced MAT3 lo	-2.1493492	1.8222487	0.00769883	0.016647768
SPBC23G7.17c	mat1-Mi	M-specific tancription factor Mi	-2.1493492	1.8222487	0.00769883	0.016647768
SPNCRNA.12	#N/A	#N/A	-2.1642238	2.20488765	0.0006634	0.001867773
SPCC417.16	SPCC417.16	mitochondrial protein (predicted)	-2.1654488	5.81478008	5.13E-26	1.41E-24
SPNCRNA.486	#N/A	#N/A	-2.1694585	2.9868634	4.75E-05	0.000166013
SPBC1348.07	SPBC1348.07	S. pombe specific DUF999 protein family 6	-2.1804778	3.005602	0.00014717	0.000472185
SPNCRNA.973	#N/A	#N/A	-2.1834524	4.78537338	1.56E-18	2.57E-17
SPNCRNA.649	#N/A	#N/A	-2.1837228	2.66020679	5.14E-05	0.000178523
SPNCRNA.1319	#N/A	#N/A	-2.1847152	6.87892009	1.01E-54	1.32E-52
SPBC336.16	#N/A	#N/A	-2.1969464	2.77932036	2.53E-05	9.32E-05
SPBPB21E7.10	SPBPB21E7.10	Schizosaccharomyces specific protein	-2.2054879	6.40834579	1.82E-53	2.21E-51
SPAC17A2.10c	SPAC17A2.10c	Schizosaccharomyces pombe specific protein	-2.212797	4.13049717	3.51E-11	2.91E-10
SPNCRNA.132	#N/A	#N/A	-2.217454	5.79856361	2.95E-35	1.43E-33
SPNCRNA.1619	#N/A	#N/A	-2.2377605	3.0266174	8.25E-06	3.32E-05
SPNCRNA.02	#N/A	#N/A	-2.2407526	2.4214671	0.00016307	0.00051902
SPNCRNA.147	#N/A	#N/A	-2.248285	1.63590483	0.01253125	0.02580967
SPNCRNA.1154	#N/A	#N/A	-2.2554662	1.36148488	0.05321437	0.091399238
SPNCRNA.323	#N/A	#N/A	-2.2567185	2.27018162	0.00060571	0.001715293
SPNCRNA.1262	#N/A	#N/A	-2.2587971	2.58521924	0.0004043	0.001191547
SPNCRNA.137	#N/A	#N/A	-2.2698174	1.36175451	0.04744076	0.082693238
SPNCRNA.1324	#N/A	#N/A	-2.2703235	9.15478903	1.01E-131	5.70E-129
SPNCRNA.208	#N/A	#N/A	-2.2707109	1.36208952	0.04779061	0.08323909
SPNCRNA.126	#N/A	#N/A	-2.273448	2.94894411	1.14E-06	5.22E-06
SPNCRNA.1621	#N/A	#N/A	-2.291966	3.94275139	4.33E-10	3.16E-09
SPCC569.09	SPCC569.09	Schizosaccharomyces specific protein	-2.293865	7.33340391	1.28E-80	3.48E-78
SPNCRNA.1631	#N/A	#N/A	-2.2958153	3.57550825	8.80E-08	4.69E-07
SPNCRNA.279	#N/A	#N/A	-2.3022703	4.32322578	4.11E-13	4.22E-12
SPAPB8E5.05	mfm1	M-factor precursor Mfm1	-2.3035009	4.95817352	2.03E-17	3.02E-16
SPNCRNA.1165	#N/A	#N/A	-2.3112448	3.17193764	1.31E-07	6.79E-07
SPNCRNA.428	#N/A	#N/A	-2.3128361	3.58840792	5.54E-08	3.05E-07
SPNCRNA.1159	#N/A	#N/A	-2.3206707	4.20633517	4.90E-08	2.73E-07
SPNCRNA.127	#N/A	#N/A	-2.3228308	3.27201299	2.33E-08	1.35E-07
SPNCRNA.181	#N/A	#N/A	-2.3265774	2.75529437	9.05E-06	3.61E-05
SPNCRNA.424	#N/A	#N/A	-2.3484888	2.33175054	0.00018291	0.000576507
SPBPJ4664.03	mfm3	M-factor precursor Mfm3	-2.3931637	5.43879018	3.18E-34	1.49E-32
SPAC5D6.10c	mug116	Schizosaccharomyces pombe specific protein Mug116	-2.4318083	2.68565601	3.89E-05	0.000137765
SPNCRNA.1039	#N/A	#N/A	-2.4364456	1.98689571	0.00544626	0.012255876
SPNCRNA.989	#N/A	#N/A	-2.4393197	5.04579181	1.31E-25	3.46E-24
SPAPB1A10.14	pof15	F-box protein (predicted)	-2.4519513	6.66036733	3.06E-67	5.79E-65

ScpofMr38	#N/A	#N/A	-2.4583805	5.62911179	5.75E-32	2.33E-30
SPBPB2B2.06c	SPBPB2B2.06c	extracellular 5'-nucleotidase, human NT5E family (predicte	-2.4870635	5.6751241	1.28E-29	4.50E-28
SPAC23C11.07	SPAC23C11.07	Schizosaccharomyces pombe specific protein	-2.5035856	2.02506387	0.00102401	0.002741196
SPBP4H10.10	rbd3	mitochondrial rhomboid family protease	-2.5202919	6.13193081	9.87E-48	9.46E-46
SPCC1183.12	spo13	sporulation specific guanyl-nucleotide exchange factor Spo1	-2.5267888	2.75614885	2.25E-06	9.92E-06
SPNCRNA.210	#N/A	#N/A	-2.5698857	2.06334564	0.0004491	0.00131332
SPBC1198.14c	fbp1	fructose-1,6-bisphosphatase Fbp1	-2.5794797	9.03693401	8.99E-144	5.56E-141
SPNCRNA.1024	#N/A	#N/A	-2.5842002	3.35162574	2.00E-07	1.01E-06
SPBC1683.08	ght4	plasma membrane hexose:proton symporter, unknown spec	-2.5975653	8.66644646	2.71E-114	1.23E-111
SPAC977.06	SPAC977.06	S. pombe specific DUF999 family protein 3	-2.6148015	2.50530948	1.26E-05	4.89E-05
SPNCRNA.431	#N/A	#N/A	-2.6251214	2.82334675	1.73E-07	8.83E-07
SPAPB1A11.01	mfc1	prospore membrane copper transmembrane transporter Mf	-2.6383565	5.51133989	6.34E-39	3.85E-37
SPNCRNA.36	#N/A	#N/A	-2.6760743	1.8651429	0.00120474	0.003172707
SPNCRNA.480	#N/A	#N/A	-2.6888946	2.13595382	0.00020256	0.000634351
SPNCRNA.326	#N/A	#N/A	-2.6973315	2.36345298	3.18E-05	0.000114299
SPBPB21E7.11	SPBPB21E7.11	Schizosaccharomyces pombe specific protein	-2.6983944	7.86392712	4.90E-150	3.71E-147
SPNCRNA.1560	#N/A	#N/A	-2.7115696	6.78345468	2.43E-63	4.03E-61
SPNCRNA.868	#N/A	#N/A	-2.7271716	5.13704534	7.72E-33	3.39E-31
SPNCRNA.439	#N/A	#N/A	-2.7392884	1.16928044	0.04561358	0.0799795
SPNCRNA.423	#N/A	#N/A	-2.7450088	1.16963333	0.04553144	0.079856038
ScpofMp06	#N/A	#N/A	-2.7469819	7.03153368	2.07E-22	4.75E-21
SPBPB21E7.05	SPBPB21E7.05	Schizosaccharomyces pombe specific protein	-2.7486827	1.58494902	0.00382112	0.008911136
SPNCRNA.1372	#N/A	#N/A	-2.7781192	3.30483405	9.73E-10	6.80E-09
SPNCRNA.316	#N/A	#N/A	-2.7977056	3.32039114	5.84E-10	4.18E-09
SPBC887.16	#N/A	#N/A	-2.8021426	2.20534182	0.0001117	0.000365249
SPNCRNA.1311	#N/A	#N/A	-2.8043766	5.37583361	1.71E-29	5.94E-28
SPNCRNA.293	#N/A	#N/A	-2.8210442	5.33336568	2.88E-29	9.70E-28
SPBP4H10.09	rsv1	transcription factor Rsv1	-2.826222	4.65826065	6.80E-22	1.54E-20
SPNCRNA.1295	#N/A	#N/A	-2.8483021	5.13520902	2.12E-29	7.24E-28
SPNCRNA.29	#N/A	#N/A	-2.8483021	5.13520902	2.12E-29	7.24E-28
SPNCRNA.182	#N/A	#N/A	-2.8852406	1.98778554	0.00024502	0.0007565
SPAC13F5.03c	gld1	mitochondrial glycerol dehydrogenase Gld1	-2.900158	8.83329575	3.93E-151	3.82E-148
SPNCRNA.312	#N/A	#N/A	-2.9678959	1.68530655	0.00449506	0.010334077
SPNCRNA.23	#N/A	#N/A	-2.9724822	3.34922149	6.47E-10	4.61E-09
SPCC1235.17	#N/A	#N/A	-2.9903882	6.29990799	9.06E-52	1.01E-49
SPCC548.07c	ght1	plasma membrane high-affinity glucose:proton symporter (	-3.013788	7.67616272	2.11E-154	2.39E-151
SPNCRNA.1549	#N/A	#N/A	-3.0554881	2.80253053	4.25E-08	2.39E-07
SPNCRNA.394	#N/A	#N/A	-3.0939947	1.77747682	0.00053826	0.001544214
SPCC1739.08c	SPCC1739.08c	short chain dehydrogenase (predicted)	-3.1405241	8.241741	3.20E-212	7.26E-209
ScpofMp07	#N/A	#N/A	-3.1671149	5.60715445	1.16E-32	5.00E-31
SPCC794.04c	SPCC794.04c	amino acid transmembrane transporter (predicted)	-3.1715326	8.73056817	3.46E-250	1.18E-246
SPNCRNA.21	#N/A	#N/A	-3.1887532	1.36267154	0.00511639	0.011578661
SPNCRNA.154	#N/A	#N/A	-3.1904392	2.70904326	4.65E-08	2.60E-07
SPAC6B12.03c	bit2	HbrB family protein involved in TOR signaling Bit2 (predicte	-3.1992406	4.72446184	7.26E-31	2.72E-29
SPNCRNA.298	#N/A	#N/A	-3.2174069	6.44555519	1.30E-86	3.86E-84
SPNCRNA.102	#N/A	#N/A	-3.2316023	2.20554211	1.96E-05	7.35E-05
SPAC2F3.07c	SPAC2F3.07c	Schizosaccharomyces pombe specific protein	-3.2525797	4.31861135	5.73E-21	1.22E-19

SPAC5D6.09c	mug86	plasma membrane acetate transmembrane transporter (pr	-3.31633	8.10346345	4.38E-147	2.98E-144
SPAC3G9.11c	pdc201	pyruvate decarboxylase (predicted)	-3.3205653	8.70514074	1.02E-71	2.24E-69
SPBPB2B2.14c	SPBPB2B2.14c	S. pombe specific DUF999 protein family 8	-3.3485412	2.82289839	2.18E-09	1.45E-08
SPNCRNA.1121	#N/A	#N/A	-3.3650973	5.99736839	5.18E-68	1.04E-65
SPNCRNA.07	#N/A	#N/A	-3.3715427	5.25004444	1.71E-37	9.17E-36
SPNCRNA.1314	#N/A	#N/A	-3.3849829	2.8461931	1.16E-09	8.09E-09
SPCC191.11	inv1	external invertase, beta-fructofuranosidase Inv1	-3.394427	7.91200419	2.66E-126	1.39E-123
SPBPB2B2.07c	SPBPB2B2.07c	S. pombe specific DUF999 protein family 7	-3.5124376	1.53181995	0.00180544	0.00456788
SPNCRNA.473	#N/A	#N/A	-3.6710462	2.13567407	4.04E-06	1.72E-05
SPNCRNA.1098	#N/A	#N/A	-3.7306254	5.07030263	2.85E-38	1.66E-36
SPNCRNA.1120	#N/A	#N/A	-3.7440056	4.41607548	1.21E-29	4.30E-28
SPCC417.15	#N/A	#N/A	-3.7492354	2.20357143	2.36E-05	8.74E-05
SPAPB24D3.10c	agl1	maltose alpha-glucosidase Agl1	-3.7559431	10.2519919	3.35E-40	2.22E-38
SPNCRNA.1313	#N/A	#N/A	-3.8920071	2.98974174	1.53E-12	1.49E-11
SPNCRNA.1256	#N/A	#N/A	-3.9671783	3.52404357	5.84E-18	9.16E-17
SPNCRNA.1223	#N/A	#N/A	-3.9857609	4.04403194	1.01E-20	2.10E-19
SPNCRNA.549	#N/A	#N/A	-4.0457667	3.59024479	9.79E-19	1.64E-17
SPCC191.10	SPCC191.10	Schizosaccharomyces pombe specific protein	-4.1943415	2.91059612	3.48E-11	2.89E-10
SPNCRNA.32	#N/A	#N/A	-4.2446057	2.55929871	6.58E-07	3.12E-06
SPNCRNA.217	#N/A	#N/A	-4.3646637	3.06442292	3.94E-12	3.66E-11
SPBC359.06	mug14	adducin, involved in actin cytoskeleton organization	-4.41263	8.69486144	2.59E-17	3.80E-16
SPNCRNA.393	#N/A	#N/A	-4.5615878	1.09920238	0.00953619	0.020184689
SPNCRNA.513	#N/A	#N/A	-4.6347392	2.84551955	2.26E-13	2.37E-12
SPAC589.11	pth4	mitochondrial translation release factor	-4.6623818	3.29045077	1.49E-16	2.05E-15
SPNCRNA.1460	#N/A	#N/A	-4.6950636	2.88905799	5.20E-14	5.68E-13
SPAC1F8.01	ght3	plasma membrane gluconate:proton symporter Ght3	-4.9772568	8.91307991	2.59E-28	8.31E-27
SPAC1F8.02c	shu1	cell-surface heme aquisition protein Shu1	-5.1496523	4.95191346	3.37E-51	3.64E-49
SPNCRNA.1559	#N/A	#N/A	-5.2672471	2.70955697	7.46E-12	6.72E-11
SPNCRNA.352	#N/A	#N/A	-5.3380081	1.47783518	0.00022477	0.000698421
SPNCRNA.37	#N/A	#N/A	-5.3402188	1.47798839	0.00013577	0.000438083
SPCC794.16	#N/A	#N/A	-5.435937	5.88486551	1.37E-91	4.23E-89
SPNCRNA.209	#N/A	#N/A	-5.6068816	1.63535113	4.77E-05	0.000166463
SPBC1348.14c	ght7	plasma membrane hexose transmembrane transporter Ght	-5.6345645	7.94113774	0	0
SPNCRNA.22	#N/A	#N/A	-5.8435967	1.77795487	5.11E-06	2.13E-05
SPNCRNA.603	#N/A	#N/A	-6.1610185	3.44260486	6.16E-19	1.05E-17
SPNCRNA.410	#N/A	#N/A	-6.5511707	2.27015158	1.78E-09	1.20E-08
SPNCRNA.30	#N/A	#N/A	-7.5305158	3.04822762	5.02E-18	7.92E-17
SPBC32H8.15	#N/A	#N/A	-7.5537538	3.06698216	7.10E-17	1.00E-15
